# Educational Software Applied in Teaching Electrocardiogram: A Systematic Review

**DOI:** 10.1155/2018/8203875

**Published:** 2018-03-15

**Authors:** Paulo A. I. Pontes, Rafael O. Chaves, Roberto C. Castro, Érica F. de Souza, Marcos C. R. Seruffo, Carlos R. L. Francês

**Affiliations:** ^1^Federal University of Para (UFPA), Belém, PA, Brazil; ^2^Federal Institute of Education, Science and Technology of Para (IFPA), Belém, PA, Brazil; ^3^State University of Para (UEPA), Belém, PA, Brazil; ^4^Federal Technological University of Paraná (UTFPR), Cornélio Procópio, PR, Brazil

## Abstract

**Background:**

The electrocardiogram (ECG) is the most used diagnostic tool in medicine; in this sense, it is essential that medical undergraduates learn how to interpret it correctly while they are still on training. Naturally, they go through classic learning (e.g., lectures and speeches). However, they are not often efficiently trained in analyzing ECG results. In this regard, methodologies such as other educational support tools in medical practice, such as educational software, should be considered a valuable approach for medical training purposes.

**Methods:**

We performed a literature review in six electronic databases, considering studies published before April 2017. The resulting set comprises 2,467 studies. From this collection, 12 studies have been selected, initially, whereby we carried out a snowballing process to identify other relevant studies through the reference lists of these studies, resulting in five relevant studies, making up a total of 17 articles that passed all stages and criteria.

**Results:**

The results show that 52.9% of software types were tutorial and 58.8% were designed to be run locally on a computer. The subjects were discussed together with a greater focus on the teaching of electrophysiology and/or cardiac physiology, identifying patterns of ECG and/or arrhythmias.

**Conclusions:**

We found positive results with the introduction of educational software for ECG teaching. However, there is a clear need for using higher quality research methodologies and the inclusion of appropriate controls, in order to obtain more precise conclusions about how beneficial the inclusion of such tools can be for the practices of ECG interpretation.

## 1. Introduction

ECG is one of the most useful complementary medical examinations [1] whose aim is to detect and assess cardiac abnormalities through waveform recording, which are changes in electrical potential between two points generated during the electrical activity of the heart [2]. ECG is a low cost, easy, and fast execution and risk-free examination that provides valuable information for the healthcare professional, thus being an important diagnostic tool of the patient's health [3]. It is possible to find in the literature the relevance of learning ECG given the importance in the practice of health professionals and in making clinical decisions [4–8]. As it is one of the most used exams and it provides important information about the cardiac activity, it is essential that medical undergraduate students learn how to properly interpret this exam while still attending university classes and activities. However, despite the importance of the ECG, the educational paradigms presently used or the most frequently used educational formats for teaching the interpretation of this examination during university training of the physician are usually traditional methodologies (e.g., through readings, lectures, and study) [5, 8, 9]. The possibility of verification through the result of this systematic literature review (SLR) shows an increasing number of researches in the area of educational software for ECG teaching since 2000, with 12 of the 17 studies.

New methodologies and techniques have been performed in the quest for improving the teaching and learning process. In this context, educational software can fit in very well. The demand for educational software is growing exponentially with the increase of interest in the Internet, educational reform, and distance learning [10]. It is already being applied in several areas of knowledge, such as musical training [11], teaching physics [12], environmental education through educational games and educational models [13, 14], how to act or behave in terrorist attacks [15], physiotherapy through the use of games with Microsoft Kinect [16, 17], and public health by raising awareness about the difficulties of blood donation through a serious game [18].

The process of making sense of the direct experience is called experiential learning [19] and is being recognized as a powerful instructional strategy [20]. Among the many types of experiential learning, we may use an educational software such as simulation and serious games. Educational software can be an alternative in the quest for improvement of the teaching and learning process of theoretical and practical aspects of electrocardiogram (ECG).

The use of educational software has some significant advantages; for example, (1) it allows practice in virtual scenarios, avoiding possible consequences of errors committed in real scenarios [21]; (2) it provides “learning by doing” in realistic situations [22], because it allows a singular practical experience; (3) students can learn at their own pace, allowing them to handle divergences during the learning process, so they will not be restricted to interact with their instructor and/or peers [23].

The objective of this study is to provide an overview, based on a systematic literature review (SLR), on the use of educational software for ECG teaching. We seek to identify (1) which software systems have been designed with educational objective of ECG teaching or those which, even if they have not been designed for this purpose, have been used for that end; (2) how their access is made available; (3) what are the subjects covered by them; (4) their learning task of teaching, referring to the tasks to be performed during their learning experience [24]; and (5) how they have been evaluated regarding training and/or learning.

The rest of the article is structured as follows: Section 2 presents a brief background on ECG.  Section 3 shows the search of related works; Section 4 explains the research method used to perform SRL; Section 5 presents the results of the SRL in ECG teaching; Section 6 presents the discussions and finally Section 7 concludes the paper.

## 2. Background

### 2.1. ECG

The electrical currents of the heart were first measured more than 100 years ago, but the ECG, as known today, was developed only at the beginning of the 20th century, by the Dutch scientist Willem Einthoven, which granted him, in 1924, the Nobel Prize in physiology or medicine for that discovery [25].

The ECG is one of the most useful complementary medical examinations [1], carried out by means of the cardiograph, whose aim is to detect and assess cardiac abnormalities through waveform recording, which are changes in electrical potential between two points generated during the electrical activity of the heart [2, 26]. The graphs are represented by means of traces called leads, in a total of 12 [27], from which there are three bipolar leads (DI, DII, and DIII), recorded through two electrodes at the ends of the members; six unipolar leads (V1, V2, V3, V4, V5, and V6), obtained through electrodes placed on the anterior surface of the precordium, plus three extended leads (aVL, aVR, and aVL), obtained by means of mathematical calculations [26].

**Figure 1:**
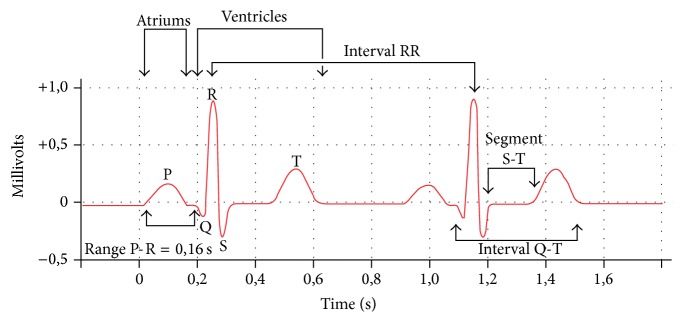
A normal electrocardiogram [2].

An ECG tracing, for a single cardiac cycle, in each one of these leads, consists physiologically of five waves: P, Q, R, S, and T. The ECG devices are designed to present these waves with high accuracy [28]. The P-wave corresponds to atrial depolarization, the Q, R, and S waves represent the ventricular depolarization, and the T wave is the graphical representation of ventricular repolarization [29], as shown in Figure 1.

It should be stressed that, before being able to understand how the cardiac abnormalities affect the ECG traces, a physician should be familiar with the concepts of vector and vector analysis applied to the electrical potentials of the heart, responsible for its contraction and relaxation, which make the heart function as a pump [2], considering that the ECG in itself is the representation of the electrical impulses generated by the heart.

Therefore, understanding the pathway of these impulses, translated by electrical vector amplitude and directions, enables the physician not only to comprehend the functionality of the normal heart, but also to define abnormalities of most of the heart diagnosis.

## 3. Related Work

Before performing the study presented in this work, a study was conducted with the purpose of seeking secondary studies that have performed the same research in the same topic proposed by our study. We applied in this work the same research principles from this SLR, following the procedures for performing systematic reviews in accordance with [30–33].  Table 1 presents an overview about the search area and the search terms used in this research.

The search string presented in Table 1 was applied in all six electronic databases used in our proposed study: ACM, PubMed, Cochrane, Scopus, Science Direct, and IEEE Xplore. No secondary studies that have carried out a literature review on the teaching of ECG through educational software were found.  Table 2 presents the search results.

## 4. Methods

The research method presented in this article was defined on the guidelines given by [33] and involves three main stages: planning, conduction, and publishing the results. During the planning stage, the research objectives are listed and a revising protocol, steps to be taken for the SLR, is defined. The protocol specifies the research questions, the strategy to be used to perform the SLR, the criteria for inclusion and exclusion, the sources of study, and the search string. In the stage of conducting the review, primary studies were selected. We performed the selection and assessment of studies in accordance with the inclusion and exclusion criteria presented in the evaluation protocol. In the results stage, after the studies have been selected, the data of the articles was extracted, synthesized, and used to answer the research questions.

It is important to emphasize that this whole process is interactive and it must be ensured that the planning is appropriate. The protocol should be evaluated and if problems are found, the researcher must return to the planning phase and review the protocol. In the conduction stage, in addition to the searches in databases, we also carried out the snowballing process [32], from reference lists of selected studies in order to identify relevant additional studies from the reference lists.

In this section, we will discuss the main steps performed for the study of systematic review.  Section 4.1 presents the research questions, Section 4.2 discusses the selection of studies, and Section 4.3 shows the classification scheme, when the classification scheme adopted is presented.

### 4.1. Research Questions

SLR, for our purposes, is focused on finding and identifying software that has been designed with the educational aim of teaching of ECG or those which, even if they have not been designed for this purpose, have been used for that objective. It is also intended to verify the subjects covered by them, how they were made available for access, and what their learning task is (detailed in Section 4.3.4) and to identify how they have been evaluated regarding training and/or learning.  Table 3 presents the research questions which this study intends to answer, as well as the justification for considering them.

### 4.2. Selection of Studies

Regarding searching for appropriate studies, we performed a selection process in which, among others, the following issues were addressed: (i) selection of source for research; (ii) terms and definition of the search string; and (iii) definition of inclusion and exclusion criteria.

#### 4.2.1. Search String

The articles used in this study have been retrieved from the use of search terms that were divided into three topics, software, electrocardiogram, and education (Table 4). It was defined that the search should occur through the definition of demand in the fields abstract and title and throughout the text.

#### 4.2.2. Database

The research was performed in six electronic databases:

- IEEE Xplore (http://ieeexplore.ieee.org)
- ACM Digital Library (http://dl.acm.org)
- Scopus (https://www.scopus.com)
- Science Direct (https://www.sciencedirect.com)
- PubMed (https://www.ncbi.nlm.nih.gov/pubmed)
- Cochrane (http://www.cochranelibrary.com).

The research team examined articles in English and Portuguese languages, from all years in the databases until April 2017.  Table 5 shows the number of search results by electronic database. In order to manage the large number of references we used the EndNote [34], a management tool, to remove eventual duplicates.

#### 4.2.3. Inclusion and Exclusion Criteria

The eligibility criteria for our review were as follows:

- The studies should be published in Portuguese or English.
- The study presents an educational software with the primary goal of teaching ECG.
- The software was not developed with educational aim but was used for this purpose, especially the teaching of ECG.
- Any type of training and/or learning evaluation was performed.

Articles that do not have software with the educational purpose were excluded, as well as those which had educational software, but their goal was not the teaching of ECG directly or in background, the studies that did not assess training and/or learning, the articles without abstract, and those that were deemed irrelevant or which were inaccessible.

#### 4.2.4. Data Extraction and Synthesis

**Figure 2:**
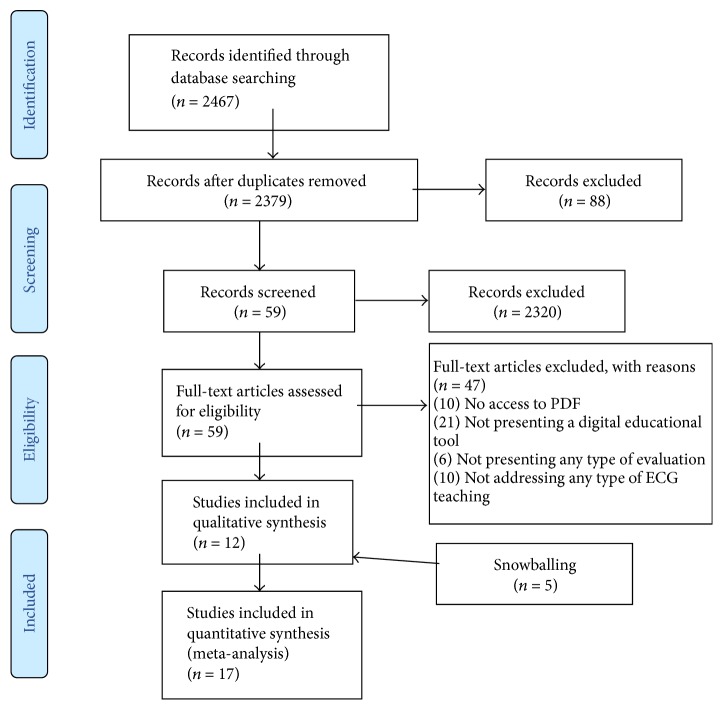
Identification and study selection process.

The process of selection of study was divided into four (4) steps, which were started after completing the execution of searches in databases, which returned 2,467 articles (S1 Appendix). For an initial screening, all search results were imported into a reference management software (EndNote). The process of identification and selection of the study and its four (4) phases are shown in Figure 2.

In the first stage, 88 articles were excluded due to being duplicated, which resulted in 2,379 (S2 Appendix) articles to make the screening. In the second stage, the articles were evaluated by means of the reviewing titles, abstracts, and keywords, in order to exclude clearly irrelevant records, considering the exclusion and inclusion criteria. This resulted in the exclusion of 2,320 articles. After applying these procedures, only 59 publications were considered relevant to the research questions (S3 Appendix). In the third stage, the articles remaining were evaluated thoroughly by reading the entire article, following the inclusion and exclusion criteria shown in Tables 5 and 6. During this stage, 47 studies were again removed: 21 not presenting educational software; 10 with no access to PDF reading; 10 by the described software not having as main objective the teaching ECG; and 6 not presenting any type of evaluation. Eventually, 12 articles were deemed relevant.

All 12 relevant articles were submitted to the fourth stage in which the snowballing process (described in Section 3) was implemented; at this stage 19 articles were found for verification. The 19 articles selected in the snowballing process were submitted to the selection of studies based on step 2, and then the articles were submitted to the inclusion and exclusion criteria, whereby only 5 articles were regarded as relevant to the research questions. Therefore, after passing all stages and criteria, a total of 17 articles were considered relevant to the research questions.

The main reason for exclusion of articles was that they only addressed the development of software that either did not have educational style, did not have the teaching of ECG as main objective, or did not make any training and/or learning evaluation.  Table 6 presents the list of selected studies. We have added a unique identifier (ID) to each article, which will be used throughout this article, to refer to the corresponding publication.

### 4.3. Classification Scheme

To perform a systematic review, it is necessary to define a classification scheme [35]. We analyzed the main characteristics and the form of application software, and so we followed two approaches: (i) based on categories already considered in the literature and (ii) taking into account the selected studies.

#### 4.3.1. Types of Educational Software (RQ1)

For the classification of software presented in the studies we decided to adopt the five categories outlined by [36], described below. We consider the classification criterion of software according to the main objective of this SRL: the teaching of ECG.

- *Drill and Practice Software. *It provides exercises in which students work out items, usually one at a time, and receive feedback on their correctness. Programs vary considerably in the kind of feedback they provide in response to student input. Feedback can range from a simple display like “OK” or “No, try again” to elaborate animated displays or verbal explanations. Some programs simply present the next item if the student answers correctly. It is different from the tutorial because it does not provide instructions; its goal is the practice.
- *Tutorial Software. *It is a result of instructions, providing all information and activities that a student needs to master a subject: summaries of information, explanation, practice routines, feedback, and evaluation. This type of software is similar to the classroom. It is normally expected to be a unit of self-contained instructions instead of an add-in for other instructions.
- *Simulator Software. *This is a computer model of real or abstract systems designed to teach how a system works [36]. It allows students to choose the tasks to be performed and the order in which they should do them, allowing the users to see the impact of their actions. It is useful when the real situation is very time consuming, dangerous, expensive, or unrealistic for a presentation in the classroom.
- *Instructional Game. *It increases the motivation by adding rules similar to games to learning activities. The common characteristics that distinguish the instruction games from other types of software are game rules, elements of competition or challenge, and entertainment or fun driven formats. Teachers often mix games with other activities to draw the attention of students or as a reward for doing other activities, even though the games themselves can be teaching tools. When students know that they will be playing a game, they expect a fun activity because of the challenge of competition and the potential expectation to win.
- *Problem Solving Software. *A mean of thinking about the solution of problems is through three of its most important components: recognition of a target (an opportunity to solve a problem), a process (a sequence of physical activities or operations), and mental activity (cognitive operations to seek a solution). Although simulations and instruction games are often used to help teaching skills of problem solving, the problem solver is specially designed for this purpose. The software features for resolving problems can concentrate on the promotion of competences of components or approaches to the overall ability of solving problems or may provide opportunities to practise solving various types of problems of content area.

On the basis of selected studies, it was possible to see that a study may encompass characteristics of more than one type of category. However, in order to classify them we considered only their characteristics towards the teaching of ECG. For example, a study that shows characteristics of a tutorial but used a simulator for the teaching of ECG was then finally classified as simulator. Another relevant aspect is that the terms used in the studies were not considered; for example, some studies claimed dealing with a simulator; however there was no discussion about the simulator itself as the concept of simulation presented in this section.

#### 4.3.2. How the Software Is Made Available (RQ2)

On the basis of selected studies, we define three categories to classify the availability of access to the software presented in this study.

- Availability for local use on PC: the software for teaching of ECG was installed locally on a computer.
- Availability for use through the Internet through a website: the software for teaching of ECG was available for use from any computer via a web page.
- Access by mobile devices: the software for ECG teaching was made available to be used on any mobile device, through the installation of an App.

#### 4.3.3. Subjects Covered (RQ3)

In this case, we applied a classification with reference to the division of subjects proposed in the summary of [2], and we created 4 (four) categories where the identified issues were classified, so that studies could teach more than one category:

- Teaching of electrophysiology and/or physiology of the heart: the electrophysiology is a science responsible for the study and understanding of electrical activity of the heart, the automatism, and the conduction of electrical stimuli between cells and tissues. In general, it studies differences in potential and conduction of electrical stimuli, as well as the properties of excitable membranes. Finally, it covers the most important aspects of the bioelectrical phenomena.
- Preparing the ECG test: the preparation is the process prior to performing the test. The test begins with the setting of 6 (six) electrodes on the skin of the patient's chest, in the region of the breasts, with the help of adhesives, and four additional pads with electrodes, which are placed on the wrists and ankles of patients.
- Normal ECG: the normal ECG is that one which does not present irregularities with respect to cardiac rhythm and frequency. The information contained in the normal ECG are heart rate of 60 to 100 beats per minute; the sinus rhythm being identified with the presence of a P-wave, which, to be normal, must present duration shorter than 0.12 seconds; PR interval with length of 0.12 to 0.20 seconds; QRS complex with duration of 0.06 to 0.10 seconds; and a normal representation in the electrical axis in the values of −30° and +90°.
- Arrhythmias: the cardiac arrhythmias occur when an electrical or mechanical abnormality interferes with the proper functioning of the heart, so it is critical to understand the physiology of arrhythmias. Arrhythmias are generated, basically, by changes in five items: rhythmicity, sinus node, blockades, normal ways of transmission of the heart, and spontaneous generation of false impulses in any part of the heart.

#### 4.3.4. Learning Task (RQ4)

In a classroom the instructor and the students have specific tasks that they must perform in order to complete the teaching/learning process in an effective way. Therefore, the instructor must provide the ideal learning environment and the proper materials, besides passing on practical knowledge acquired from work experience in order to motivate students to learn in a significant way and to successfully perform the learning tasks [37, 38]. The learning task may follow certain pattern due to the type of educational software used. However, it is likely that some significant differences exist in learning task even within the same type of educational software. The learning task refers to the tasks to be performed during the learning experience. These tasks will determine, based on the accomplishment of specific steps, the things students will need to go through in order to learn the content and reach the goals outlined by [38].

#### 4.3.5. Evaluation of Training and/or Learning (RQ5)

This RQ aims to assess the study designs used to evaluate learning experience referred to in the studies. To classify the types of evaluations that were used for training and learning, the team classified the evaluations with respect to the level and type of evaluation of studies. We classified the level of evaluation of studies according to Kirkpatrick's four levels for evaluation model [21, 39], a popular and widely used model for evaluation of training and learning:

- Level 1: evaluation of reaction: it evaluates how participants felt about the training or learning experience, normally performed by means of questionnaires distributed at the end of a learning experience: feedback forms; verbal reactions; posttraining surveys.
- Level 2: evaluation of learning: it evaluates the increase of knowledge or skills. Evaluations and tests occur before and after the training, interviews, or observation.
- Level 3: evaluation of behavior: it evaluates the degree to which the new learning gained during the training actually transfers to the job, measuring actual performance in the work environment. It is performed through observation and interviews over time to assess changes, relevance of change and sustainability of change, observation of work performance, and review of administrative data.
- Level 4: evaluation of results: it is evaluation of the effect in today's business environment by the student: posttraining investigations over the long term; observation as part of an ongoing, sequenced training and coaching for a period of time; metrics, such as rework and errors, to measure whether participants have achieved the training objectives; interviews with trainees and their managers or groups of customers.

Regarding the type of evaluation, we classified the study design used in educational contexts as follows; in this case we will apply the classification used by [21] where the evaluations were divided into three groups.

- Nonexperimental: no control groups were used or no comparisons were made with pretest to assess the capabilities of the students.
- Quasi-experimental: there are a pretest and posttest without the presence of a control group.
- Experimental: a precise comparison (pre/posttests) was included for a randomly selected control group.

## 5. Results

In this section, we present the results for each of the research questions defined in Section  4.1.

### 5.1. Types of Software (RQ1)

**Figure 3:**
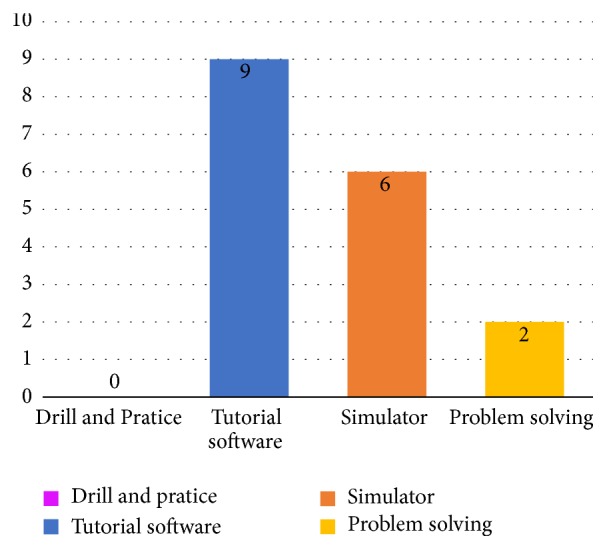
Types of software.

This RQ refers to types of software that has been found in the studies. A study may cover more than one feature; however we considered the classification criterion of software according to the main objective of this SRL: the teaching of ECG. Most of the software types found in the studies (52.9% or 9 of 17) are tutorials; 35.3% (or 6 of 17) are simulators and 11.8% (2 of 17) of the software types are for problem solving.  Figure 3 shows the classification of the software found in each study.

### 5.2. Availability (RQ2)

**Figure 4:**
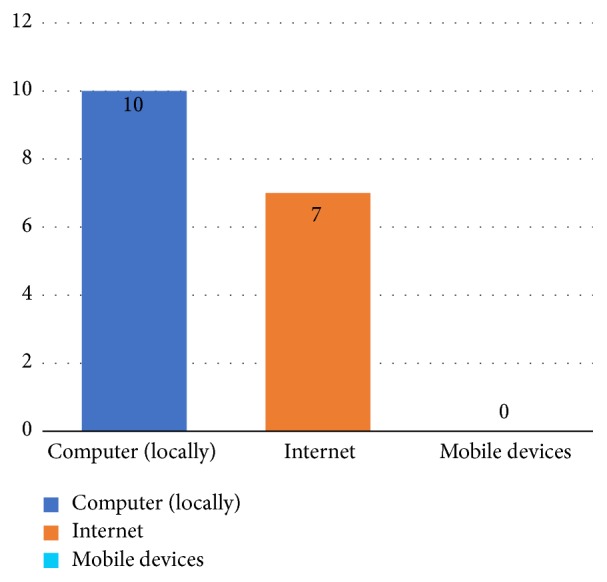
Availability of access of the software.

This RQ refers to form of availability for access of the software found in the studies. In the majority of the studies, 58.8% (10 of 17) of the software types are installed locally on a computer and 41.2% (7 of 17) of the software types are made available to be accessed from any computer, as they are available on the Internet through a website.  Figure 4 shows the forms of availability of access of the software.

### 5.3. Content Knowledge (RQ3)

**Figure 5:**
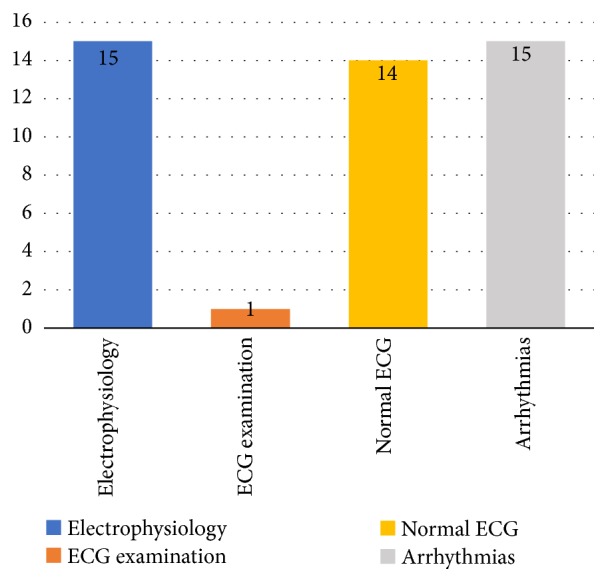
Subjects and details of learning.

This RQ refers to the major subjects that were addressed in the studies. The teaching of electrophysiology and/or physiology of the heart was addressed in 88.2% (15 of 17) of the selected studies. The identification of ECG patterns, through the provision of standards, information, characteristics, or explanation, was addressed in a larger number for the teaching of arrhythmias, 88.2% (15 of 17), and for the teaching of normal ECG, 82.4% (14 of 17). In only 5.9% (1 of 17) the preparation for ECG testing was covered.  Figure 5 shows the subjects addressed in each study.

### 5.4. Learning Task (RQ4)

This RQ refers to the learning tasks performed during the learning experience in the studies. The learning task may follow a certain pattern due to the type of educational software used (shown in Section 5.1). Despite having the same classification, students may perform different learning tasks even within the same educational software, according to the teaching/learning needs desired by the teachers. In 52.9% (9 of 17) of the studies, represented by the tutorials, the students would read a textual material on the subjects covered and view images, diagrams of ECG, videos, and/or animations. Then, the students would answer questionnaires (exercises) on the subjects studied, made available at the end of a chapter or by the user's request at any time. It is worth noticing that only in S5 the students could both watch the arrhythmias drawing in real time and control the speed of the animation through commands provided by the software.

In 35.3% (6 of 17) the learning tasks were represented by simulators, in which participants could change parameters and thereby allow observing immediate effects on ECG. In two studies (S2 and S17) students could, in addition to changing parameters, read a textual content on the matters proposed and thus complement the process of teaching/learning. Only S12 addressed the positioning of the electrodes of the ECG. In this study, the participant had to position the electrodes and see the effects on ECG.

For 11.8%, represented by problem solving, the proposal for a learning task was based on the availability of problems encountered in the real world. In S7 students used tools (rulers, calipers, and magnification) for measuring and comparing ECG intervals and thus identify the rhythms (normal ECG and arrhythmias). While in S15 the students observed scenarios where ECG rhythms were presented, they should identify the arrhythmias and suggest the appropriate interventions, including the prescription of drugs or the use of a defibrillator or pacemaker.

### 5.5. Evaluation and Outcomes of Training and/or Learning (RQ5)

**Figure 6:**
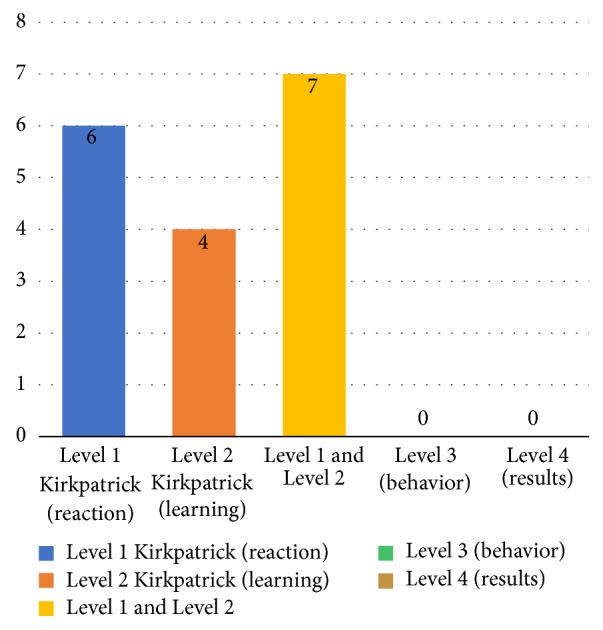
Level educational evaluation of studies.

This RQ refers to the evaluation of training and/or learning and respective outcomes of the studies. Most of the studies (76.5% or 13 of 17) assessed the participants' reaction (Kirkpatrick level 1), characterized by the way in which the participants feel about the training or learning experience. And 64.7% (11 of 17) assessed the level of learning (Kirkpatrick level 2) through the increase of knowledge or skills (Figure 6).

**Figure 7:**
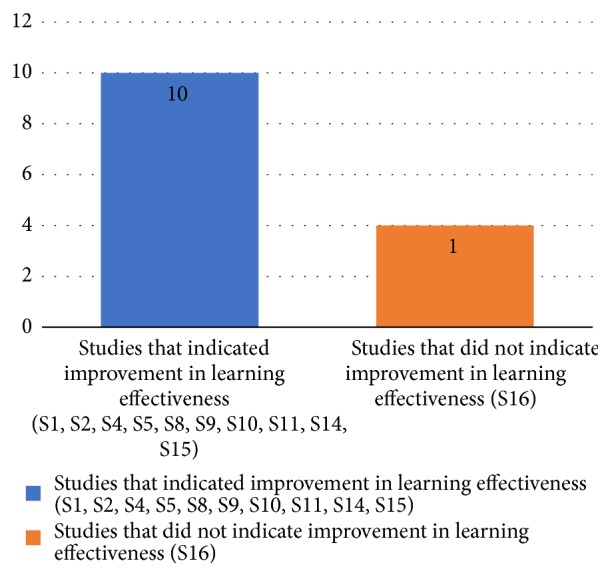
Studies that indicated and did not indicate improvement in learning effectiveness.

In 90.9% (10 of 11) of the studies using Kirkpatrick's level 2, the assessments indicated improvement in learning effectiveness, knowledge about electrocardiogram, and/or the ability to interpret ECGs.  Figure 7 details the studies that indicated and did not indicate improvement in learning effectiveness.

**Figure 8:**
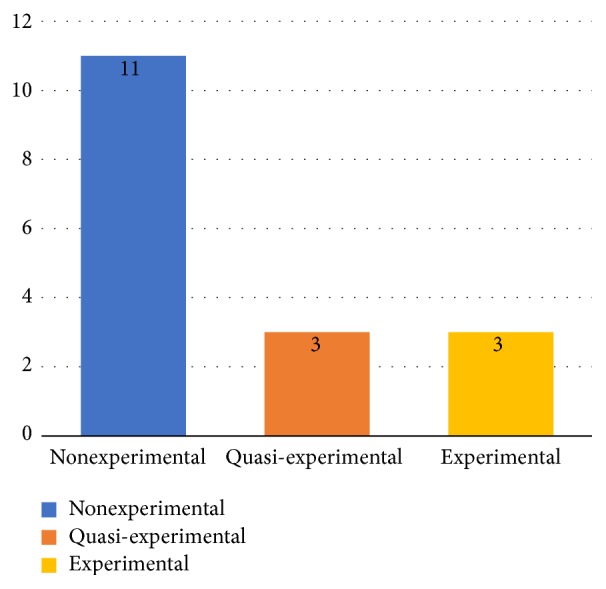
Information of the studies according to the characteristics of the assessments performed.

The type of training evaluation and learning of selected articles followed a study design considered nonexperimental in 64.7% (11 of 17), while in 17.6% (3 of 17) a study design was considered quasi-experimental and 17.6% (3 of 17) proposed an experimental design.  Figure 8 shows the information of the studies according to type of the assessments performed. A complete overview of the extracted data is available in S4 Appendix.

## 6. Discussion

The ECG is an important diagnostic tool and one of the most widely used complementary tests in medical practice. It is generally used in order to detect and assess cardiac abnormalities through ultrasound waves recording. Thus, a proper understanding and interpretation of this test are mandatory. However, several studies have addressed the struggles in teaching ECG using traditional methodologies, such as reading sessions, lectures, and study groups [4, 5, 9, 40]. Therefore, educational software might be an alternative as a learning support approach, which can bring significant results to ECG teaching.

The results obtained in this SLR show how the educational software for ECG teaching is being used. To that end, our study selected 17 articles published in the last 34 years, three of which were carried out in the 1980s and two in the 1990s. Our results indicate a peak of publication, for this kind of studies, during the 2000s, when the publication of 12 out of the 17 studies took place.

In regard to the types of software (RQ1), the majority is composed of tutorials (52.9% or 9 out of 17). This predominance may be due to the fact that this is one of the oldest paradigms of educational software. Nevertheless, this type of software shows positive educational effects (e.g., 7 out of the 9 tutorial type studies have had positive results on learning effectiveness (level 2)). In addition, this model is the one that most closely resembles the classical teaching activities [36], allowing self-stimulated instruction assessments and coming up as a learning and instruction alternative when teachers are not available. However, it is worth mentioning that this type of software presents some difficulties concerning its development, addressed by [36] as follows: (a) difficulty in creating a didactic tutorial able to properly teach a certain subject with the learning tasks that this subject requires; (b) knowing the best sequence to follow; (c) how to properly explain and demonstrate essential concepts; (d) knowing the most common mistakes students are likely to make; (e) how to provide instruction and feedback to correct such mistakes; and (f) the way of teaching a particular subject which is often in disagreement with teachers, especially regarding how to teach more effectively and in what order to present the tasks.

Considering the availability of educational software (RQ2), in other words, in which platform the user can access the software, the results indicated that most of them (58.8% or 10 of 17) were developed to be available for local access, as in a desktop or laptop type of personal computer. It was also possible to verify that there is a tendency for web software to be available in the studies found between 2006 and 2016. During this period, 41% of the studies were developed to be available for Internet based use, allowing the user to access it on any computer. This trend may be related to the increase in the number of users with Internet access [41], the influence of the Internet on human life [42], or the general advantages of the Internet such as availability, geographic independence, flexibility, improved visualization, and interaction opportunities [43]. Another advantage of using the software on the Internet, addressed by S13, is the possibility of accessing it using only a browser.

Regarding the subjects addressed (RQ3), the majority of the studies focused on the teaching of electrophysiology and/or heart physiology, identification of ECG patterns, information and characteristics of arrhythmias, and normal ECG pattern. This result shows that the studies follow the same model applied by the main textbooks of human physiology and electrophysiology [2, 44, 45], which are used as a standard in universities, where the teaching of ECG must begin with the basic concepts of electrophysiology. Our findings showed that only one study addressed the ECG test preparation, which may reveal a topic with little teaching focus. In addition, it is also in agreement with [46], which claims that despite the relevance of the test preparation, ECG textbooks contain little or no information on the effects of incorrect electrode positioning and also that current pedagogical tools that include training manikins do not allow free positioning of the electrodes to demonstrate these effects. In our study, it is evidenced that some studies claim that between 0.4% and 4% of all 12-lead electrocardiograms (ECG) are recorded using incorrect electrode positions, and this incorrect positioning can cause a misdiagnosis, hide a pathology, or even emulate a pathology.

Most of the studies that we found followed the same learning task design (RQ4) in order to achieve proper learning. The tasks performed by the majority of the students included the following: (a) reading of textual material on the subjects covered by the software; (b) visualization of images, ECG diagrams, videos, and/or animations; (c) questionnaires (exercises) on the subjects presented, made available at the end of a chapter or by the user's request at any time. This type of learning task design may have been predominant due to the fact that it is a methodological format similar to the instructions given in the classroom by a teacher, which include training and practical activities with immediate feedback, and the fact that it has achieved positive results in learning improvement, as showed in this SLR in 8 out of the 9 studies that carried out this type of learning task.

Regarding the type of training evaluation and/or learning of educational software (RQ5), we found that only a few studies (17% or 3 out of 17) apply an experimental research design. In our results, two (S4, S15) of the three studies that performed an experimental study design proved that there was a significant increase in performance on electrocardiogram knowledge and in ECG's interpretation skills. Although the use of experimental design is arguably the best practice for evaluating learning effectiveness [47], the fact that we have found few experimental studies may be related to the complexity of performing adequate experiments that indicate reliable levels of evidence [48]. This complexity is related to three factors addressed by [49]: (a) the effort required to run this type of experiment, which can cause disruption in the flow of the course and may not be well accepted by the students themselves; (b) requiring control groups that may be harmed by the use of alternative teaching methods which are considered inferior; and (c) a considerable sample size which is required in order to obtain statistically significant results from such experiments, especially due to the need for an experimental and a control group.

The studies were only evaluated at levels 1 and 2 according to Kirkpatrick's Four Levels of Evaluation [39]. Participants' learning outcomes were mainly assessed through level 1, probably due to the ease of obtaining information through feedback, verbal reactions, and survey. However, this may not be enough to adequately assess or capture the participants' actual learning effect. Further studies using the second level of assessment need to be carried out in order to determine whether the learning objectives have been met.

These results are in agreement with other studies which indicated that the inclusion of educational software might result in several benefits, such as increasing the effectiveness of learning, increasing interest and motivation, and reducing teaching time [21, 50]. However, it is necessary to perform investigation methodologies in studies with experiments of greater level of evidence and inclusion of adequate controls, in order to draw more accurate conclusions, and thus to check whether the inclusion of educational software as an instructive strategy, which is effective and efficient way for teaching and learning, is truly beneficial.

### 6.1. Threats to Validity

As in any systematic review, there are some threats to the validity of the results. For this reason, we identified potential threats and apply mitigation strategies to minimize their impact.

#### 6.1.1. Publication Bias

Our results have been against what has been said by [49, 51], which indicates that the systematic reviews suffer from the common bias that positive results are more likely to be published than negative ones. This was evident in this review, in which few studies have negative results of evaluation of software, only 2 out of 17. However, we do not believe that this was a critical threat to our research; instead of focusing on the impact of educational software, our goal was to identify how they were evaluated.

#### 6.1.2. Identification of Studies

Another risk is the omission of relevant studies. To mitigate this risk, we have carefully built the search sequence to be as comprehensive as possible, considering not only the basics, but also synonyms. The risk of exclusion of primary studies has yet been mitigated through the use of multiple databases that cover the majority of scientific publications in the field. It was defined that the search string should go in the fields abstract and title and throughout the text. The additional mitigation was achieved through the inclusion of studies that were identified in the process of snowballing.

#### 6.1.3. Study Selection and Data Extraction

The threats to the selection of studies and extraction of data were mitigated through a detailed definition of inclusion and exclusion criteria. We have defined and documented a strict protocol for the selection of the study; the data obtained from the selected articles would then be examined independently by two authors. The discrepancies and disagreements, as well as any differences established, should be resolved in discussion with a third author.

## 7. Conclusion

According to [32] SLR is a way to identify, evaluate, and interpret all available research relevant to a particular research question, topic area, or phenomenon of interest. Thus, in order to obtain the state of the art on the implementation of educational software for the teaching of ECG, we carried out the first systematic literature review (SLR) exploring this theme. The main contributions of this work are as follows: (1) the identification of software with the educational purpose of ECG; (2) a vision of how such software is available for free; (3) which subjects are addressed by them; (4) strategies for learning task; and (5) the assessments of training and/or learning.

We identified 17 articles that assessed different software. The most found type of software was tutorial, with 52.9% (9 of 17) of the total number of studies. Most of software types, 58.8% (10 of 17), are designed to be run locally on a computer. The subjects were discussed together with a greater focus on the teaching of electrophysiology and/or cardiac physiology and identifying patterns of ECG and/or arrhythmias. The results showed the insertion of educational software for ECG teaching to be positive; however the evidence base on which these inferences were made shows low evidence level and thus these benefits cannot be confirmed or claimed. There is a clear need to use higher quality research methodologies and the inclusion of appropriate controls, in order to obtain more precise conclusions about how beneficial the inclusion of these tools can be for the practices of ECG interpretation. As this is one of the most used diagnostic tests in medicine and, thus, it is essential for medical students to effectively learn ECG during their university training.

## Figures and Tables

**Table 1:** The search area and search keywords in the study.

Search area	Search terms
*Software*	game OR simulator OR tutorial OR “educational software” OR “Instructional Software” OR APP
*Education *	education OR teaching OR learning OR training OR instruction OR interpretation
*Electrocardiogram*	electrocardiogram OR electrocardiography OR ECG OR EKG
*Review*	“Systematic Literature Review” OR “Systematic Review” OR “Systematic Mapping” OR “Mapping Study” OR “Mapping Studies” OR “Systematic Literature Mapping” OR “Literature Review”

**Table 2:** Results of the searches in the databases.

*ACM*	*7*
*PubMed*	1
*Cochrane*	1
*Scopus*	542
*Science Direct*	0
*IEEE Xplore*	0

*Total*	551

*Relevant articles*	None

**Table 3:** Research questions (RQs).

Number	Research question	Rationale
RQ1	What types of software are used with educational aim for the teaching of ECG?	This research question aims to investigate which categories of software (Drill and Practice, Simulator, instructional games, Problem Solving) were used for the teaching and interpretation of ECG.

RQ2	How are the software made available for access?	Check in what platforms software is available.

RQ3	What are the issues addressed by software?	Identify which subtopics have been further explored by these software for the teaching of ECG.

RQ4	What are the learning tasks?	Identify the learning tasks used by such software (referring to the tasks to be performed during their learning experience).

RQ5	What are the levels, types, and respective results of the study evaluations regarding training and/or learning?	Identify the level, the type, and its respective results. To classify the level, we used Kirkpatrick's four levels.

**Table 4:** Search terms on educational software for learning of ECG.

Identification	Keywords
*Type of software*	Game, tutorial, simulator, “educational software”, “Instructional Software”, APP

*Electrocardiogram*	“electrocardiogram”, “electrocardiography”, “ECG”, “EKG”

*Education*	Education, teaching, learning, training, instruction, interpretation

*Search terms*	(game OR tutorial OR simulator OR “educational software” OR “Instructional Software” OR APP) AND (education OR teaching OR learning OR training OR instruction OR interpretation) AND (electrocardiogram OR electrocardiography OR ECG OR EKG)

**Table 5:** Number of studies per database.

Database	Number of studies
*ACM*	18
*PubMed*	91
*Cochrane*	13
*Scopus*	1837
*Science Direct*	510
*IEEE Xplore*	0

*Total*	2469

*Number of duplicated studies*	88

**Table 6:** List of selected studies.

ID	Author	Title	Sample size	Year	Country	Publication type	Reference
S1	Shahein	“Computers in Health-Sciences Education. An Application to Electrocardiography”	n	1983	Saudi Arabia	Journal	[52]

S2	Fukushima et al.	“Computer-Assisted Education System for Arrhythmia (CAESAR)”	13	1984	Japan	Journal	[53]

S3	Golding	“Cardiac Rhythms and Arrhythmias: A Teaching Program”	50	1986	EUA	Journal	[54]

S4	Devitt et al.	“Evaluation of a Computer Based Package on Electrocardiography”	61	1998	Australia	Journal	[55]

S5	Takeuchi et al.	“WinArrhythmia: A Windows Based Application for Studying Cardiac Arrhythmias”	n	1998	Japan	Journal	[56]

S6	Takeuchi et al.	“Simulation System of Arrhythmia Using ActiveX Control”	n	2005	Japan	Journal	[57]

S7	Criley and Nelson	“Virtual Tools for Teaching Electrocardiographic Rhythm Analysis”	21	2006	EUA	Journal	[58]

S8	Patuwo et al.	“Comparison of Teaching Basic Electrocardiographic Concepts with and without ECGSIM, an Interactive Program for Electrocardiography”	35	2007	EUA	Journal	[9]

S9	Burke et al.	“Critical Analysis of a Computer-Assisted Tutorial on ECG Interpretation and Its Ability to Determine Competency”	50	2008	EUA	Journal	[59]

S10	Nilsson et al.	“Evaluation of a Web-Based ECG-Interpretation Programme for Undergraduate Medical Students”	32	2008	Sweden	Journal	[60]

S11	Lessard et al.	“An ECG Analysis Interactive Training System for Understanding Arrhythmias”	18	2009	France	Journal	[43]

S12	Bond et al.	“A Simulation Tool for Visualizing and Studying the Effects of Electrode Misplacement on the 12-Lead Electrocardiogram”	17	2011	United Kingdom	Journal	[61]

S13	Çakırog	“WebECG: A Novel ECG Simulator Based on MATLAB Web Figure”	40	2012	Turkey	Journal	[62]

S14	Bojsen et al.	“The Acquisition and Retention of ECG Interpretation Skills after a Standardized Web-Based ECG Tutorial-A Randomised Study”		2015	Denmark	Journal	[63]

S15	Tubaishat and Tawalbeh	“Effect of Cardiac Arrhythmia Simulation on Nursing Students' Knowledge Acquisition and Retention”	100	2015	Jordan	Journal	[64]

S16	Davies et al.	“E-Learning and Near-Peer Teaching in Electrocardiogram Education: A Randomised Trial”	39	2016	United Kingdom	Journal	[65]

S17	Fent et al.	“A Randomized Control Trial Comparing Use of a Novel Electrocardiogram Simulator with Traditional Teaching in the Acquisition of Electrocardiogram Interpretation Skill”	168	2016	United Kingdom	Journal	[40]

## Supplementary Materials

Supplementary 1 S1 Appendix: identification—records identified through database searching (DOCX).

Supplementary 2 S2 Appendix: screening—records after duplicates removed (DOCX).

Supplementary 3 S3 Appendix: eligibility—full-text articles assessed for eligibility (DOCX).

Supplementary 4 S4 Appendix: complete overview of the extracted data (DOCX).
