# Supplementary material for: Educational Software Applied in Teaching Electrocardiogram: A Systematic Review
Source: Biomed Res Int. 2018 Mar 15;2018:8203875. doi: 10.1155/2018/8203875 (PMC5875041; doi:10.1155/2018/8203875)
Supplement: Supplementary 1 — S1 Appendix: identification—records identified through database searching (DOCX). [file 8203875.f1.docx]

**S1 Appendix -IDENTIFICATION - Records identified through database searching**

The following 2467 papers identified through searching.

1. Cipryan L. The effect of fitness level on cardiac autonomic regulation, IL-6, total antioxidant capacity, and muscle damage responses to a single bout of high-intensity interval training. Journal of Sport and Health Science. doi: http://doi.org/10.1016/j.jshs.2016.11.001.

2. Duhig SJ, Williams MD, Minett GM, Opar D, Shield AJ. Drop punt kicking induces eccentric knee flexor weakness associated with reductions in hamstring electromyographic activity. Journal of Science and Medicine in Sport. doi: http://doi.org/10.1016/j.jsams.2016.09.006.

3. Mamede S, Schmidt HG. Reflection in Medical Diagnosis: A Literature Review. Health Professions Education. doi: http://doi.org/10.1016/j.hpe.2017.01.003.

4. Montayre J, Sparks T. Important yet unnecessary: Nursing students' perceptions of anatomy and physiology laboratory sessions. Teaching and Learning in Nursing. doi: http://doi.org/10.1016/j.teln.2017.03.009.

5. Nelson K, Nicholls C, Muckler VC. Pediatric Review and Perioperative Considerations. Journal of PeriAnesthesia Nursing. doi: http://doi.org/10.1016/j.jopan.2016.03.011.

6. Park H-R, Park J-W, Kim C-J, Song J-E. Development and validation of simulation teaching strategies in an integrated nursing practicum. Collegian. doi: http://doi.org/10.1016/j.colegn.2016.10.007.

7. Patey O, Gatzoulis MA, Thilaganathan B, Carvalho JS. Perinatal Changes in Fetal Ventricular Geometry, Myocardial Performance, and Cardiac Function in Normal Term Pregnancies. Journal of the American Society of Echocardiography. doi: http://doi.org/10.1016/j.echo.2017.01.011.

8. Neubert FR. An allopath at sea. British Homoeopathic journal. 1945;35(1):47-51. doi: http://doi.org/10.1016/S0007-0785(45)80030-3.

9. Gliebe PA, Kerr WJ. Recognition of emotional factors in allergic manifestations. The American Journal of Medicine. 1947;3(5):607-13. doi: http://doi.org/10.1016/0002-9343(47)90205-2.

10. Bogdonoff MD, Woods AH, White JE, Engel FL. Hyperparathyroidism. The American Journal of Medicine. 1956;21(4):583-95. doi: http://doi.org/10.1016/0002-9343(56)90074-2.

11. Complications of diabetes mellitus. The American Journal of Medicine. 1958;25(4):627-37. doi: http://doi.org/10.1016/0002-9343(58)90051-2.

12. Acute renal failure of obscure etiology. The American Journal of Medicine. 1961;30(3):464-71. doi: http://doi.org/10.1016/0002-9343(61)90054-7.

13. Procter WI. Subacute bacterial endocarditis due to Erysipelothrix rhusiopathiae: Report of a case and review of the literature. The American Journal of Medicine. 1965;38(5):820-4. doi: http://doi.org/10.1016/0002-9343(65)90203-2.

14. Gilroy J, Meyer JS, Bauer RB, Vulpe M, Greenwood D. Clinical, biochemical and neurophysiological studies of chronic interstitial hypertrophic polyneuropathy. The American Journal of Medicine. 1966;40(3):368-83. doi: http://doi.org/10.1016/0002-9343(66)90132-X.

15. News in ORbit. AORN Journal. 1967;5(3):42-55. doi: http://doi.org/10.1016/S0001-2092(08)71142-6.

16. AORN Proceedings. AORN Journal. 1967;5(1):82-91. doi: http://doi.org/10.1016/S0001-2092(08)71360-7.

17. Kuramoto K, Ikai M, Asahina K, Ktjroda Y, Ogawa S, Miyashita M. Strenuous Exercise Electrocardiogram of Top Class Swimmers in Mexico City. Jpn Heart J. 1967;8(3):291-300. doi: 10.1536/ihj.8.291.

18. Sohar E, Gafni J, Pras M, Heller H. Familial Mediterranean fever: A survey of 470 cases and review of the literature. The American Journal of Medicine. 1967;43(2):227-53. doi: http://doi.org/10.1016/0002-9343(67)90167-2.

19. Sapira JD. The narcotic addict as a medical patient. The American Journal of Medicine. 1968;45(4):555-88. doi: http://doi.org/10.1016/0002-9343(68)90172-1.

20. News in ORbit. AORN Journal. 1969;9(6):121-9. doi: http://doi.org/10.1016/S0001-2092(08)70383-1.

21. Noteworthy references. AORN Journal. 1970;12(3):131-6. doi: http://doi.org/10.1016/S0001-2092(07)60228-2.

22. Bennett LR. THIS I BELIEVE … THAT NURSES MAY BECOME EXTINCT. AORN Journal. 1970;11(4):57-63. doi: http://doi.org/10.1016/S0001-2092(07)62418-1.

23. Northrup FC. Involvement with children's surgery. AORN Journal. 1970;12(6):69-76. doi: http://doi.org/10.1016/S0001-2092(07)60695-4.

24. Technical exhibits. AORN Journal. 1971;13(1):132-40. doi: http://doi.org/10.1016/S0001-2092(07)67081-1.

25. Hanne-Paparo N, Wendkos MH, Brunner D. T wave abnormalities in the electrocardiograms of top-ranking athletes without demonstrable organic heart disease. Am Heart J. 1971;81(6):743-7. doi: 10.1016/0002-8703(71)90078-0.

26. Maddy JA, Winternitz WW. Hypothalamic syndrome with hypernatremia and muscular paralysis. The American Journal of Medicine. 1971;51(3):394-402. doi: http://doi.org/10.1016/0002-9343(71)90275-0.

27. McArdle WD, Magel JR, Kyvallos LC. Aerobic capacity, heart rate and estimated energy cost during women’s competitive basketball. Res Q Am Assoc Health, Phys Educ Recreat. 1971;42(2):178-86. doi: 10.1080/10671188.1971.10615055.

28. AORN proceedings. AORN Journal. 1972;16(1):129-36. doi: http://doi.org/10.1016/S0001-2092(07)61974-7.

29. A thirty-eight year old woman with overwhelming sepsis. The American Journal of Medicine. 1972;53(2):233-41. doi: http://doi.org/10.1016/0002-9343(72)90132-5.

30. Kanal LN. Interactive pattern analysis and classification systems: A survey and commentary. Proc IEEE. 1972;60(10):1200-15. doi: 10.1109/PROC.1972.8880.

31. Crossword puzzles. AORN Journal. 1973;17(6):141-50. doi: http://doi.org/10.1016/S0001-2092(07)60312-3.

32. Blanksby BA, Elliott BC, Bloomfield J. Telemetered heart rate responses of middle aged sedentary males, middle aged active males and 'A' grade male squash players. MED J AUST. 1973;2(10):477-81.

33. Duff WR, Mannes JH, Breitmeyer MO. Clinical device note: a versatile ECG simulator for laboratory and teaching applications. Med Instrum. 1973;7(4):235-6. Epub 1973/09/01. PubMed PMID: 4746838.

34. Koerner DR. Cardiovascular benefits from an industrial physical fitness program. J Occup Med. 1973;15(9):700-7.

35. Lichtman J, O'Rourke RA, Klein A, Karliner JS. Electrocardiogram of the Athlete: Alterations Simulating Those of Organic Heart Disease. Arch Intern Med. 1973;132(5):763-70. doi: 10.1001/archinte.1973.03650110095021.

36. Schwartz WB, Gorry GA, Kassirer JP, Essig A. Decision analysis and clinical judgment. The American Journal of Medicine. 1973;55(4):459-72. doi: http://doi.org/10.1016/0002-9343(73)90203-9.

37. Diabetes mellitus. The American Journal of Medicine. 1974;57(6):940-8. doi: http://doi.org/10.1016/0002-9343(74)90172-7.

38. Noteworthy references. AORN Journal. 1974;20(1):152-6. doi: http://doi.org/10.1016/S0001-2092(07)67111-7.

39. Gordon MS. Cardiology patient simulator. Development of an animated manikin to teach cardiovascular disease. Am J Cardiol. 1974;34(3):350-5. doi: 10.1016/0002-9149(74)90038-1.

40. Gordon MS. Cardiology patient simulator. Development of an animated manikin to teach cardiovascular disease. Am J Cardiol. 1974;34(3):350-5. Epub 1974/09/01. PubMed PMID: 4136577.

41. Ogirimah A, Cunningham D, Rechnitzer P, Yuhasz M. Comparison of effects of two types of exercise programs on work capacity and electrocardiogram of patients with previous myocardial infarction. Jsport Med (Torino) [Internet]. 1974; 14(1):[1-7 pp.]. Available from: http://onlinelibrary.wiley.com/o/cochrane/clcentral/articles/817/CN-00193817/frame.html.

42. Ogirimah AM, Cunningham DA, Rechnitzer PA, Yuhasz MS. Comparison of effects of two types of exercise programs on work capacity and electrocardiogram of patients with previous myocardial infarction. J SPORTS MED PHYS FITNESS. 1974;14(1):1-7.

43. Patrick EA, Patrick EA, Stelmack FP, Shen LYL. Review of Pattern Recognition in Medical Diagnosis and Consulting Relative to a New System Model. IEEE Trans Syst Man Cybern. 1974;SMC-4(1):1-16. doi: 10.1109/TSMC.1974.5408515.

44. Read RC, Hall WH. Objective assessment of gastric function after vagotomy. Curr Probl Surg. 1974;11(7):1,3-63. doi: 10.1016/S0011-3840(74)80009-2.

45. Rottet SM. Gaming as a learning strategy. J Contin Educ Nurs. 1974;5(6):22-5.

46. Rottet SM. Gaming as a learning strategy. J Contin Educ Nurs. 1974;5(6):22-5. Epub 1974/11/01. PubMed PMID: 4497850.

47. Selected bibliography. PAIN. 1975;1(2):195-211. doi: http://doi.org/10.1016/0304-3959(75)90128-1.

48. Pfeiffer EA. A simulator for teaching electrical safety procedures in the hospital. Med Instrum. 1975;9(2):103-5. Epub 1975/03/01. PubMed PMID: 1128310.

49. Widrow B, Williams CS, Glover JR, Jr., McCool JM, Hearn RH, Zeidler JR, et al. Adaptive Noise Cancelling: Principles and Applications. Proc IEEE. 1975;63(12):1692-716. doi: 10.1109/PROC.1975.10036.

50. Corfield JR. Computers in medical physics. BRITJCLINEQUIP. 1976;1(5):278-82.

51. Fisher KD. Reaction to “Perspective”. Journal of Nutrition Education. 1976;8(4):186-7. doi: http://doi.org/10.1016/S0022-3182(76)80099-4.

52. Jarmon RG, Yesalis Iii CE. Provider performance in the recognition and treatment of telemetered electrocardiogram patterns. Journal of the American College of Emergency Physicians. 1976;5(12):971-4. doi: 10.1016/S0361-1124(76)80124-4.

53. Mustacchi P. Arterial hypertension and the work environment: Some considerations affecting its compensability. J Occup Med. 1976;18(8):561-6.

54. Jarmon RG. Cardiac telemetry exercise program. Journal of the American College of Emergency Physicians. 1977;6(2):50-2. doi: 10.1016/S0361-1124(77)80032-4.

55. Jordan CH. Awareness for action. AORN Journal. 1977;25(7):1317-36. doi: http://doi.org/10.1016/S0001-2092(07)67797-7.

56. Howlett PJ, Pearson SA. Simple e.c.g. arrhythmia simulator. Med Biol Eng Comput. 1978;16(2):217-8. doi: 10.1007/BF02451926.

57. Lie H, Erikssen J. ECG Aberrations, Latent Coronary Heart Disease and Cardiopulmonary Fitness in Various Age Groups of Norwegian Cross‐Country Skiers. Acta Med Scand. 1978;203(1-6):503-7. doi: 10.1111/j.0954-6820.1978.tb14916.x.

58. Maron MB, Horvath SM. The marathon: A history and review of the literature. Med Sci Sports. 1978;10(2):137-50.

59. Sears DA. The morbidity of sickle cell trait: A review of the literature. The American Journal of Medicine. 1978;64(6):1021-36. doi: http://doi.org/10.1016/0002-9343(78)90458-8.

60. Barlow JS. Computerized Clinical Electroencephalography in Perspective. IEEE TRANS BIOMED ENG. 1979;BME-26(7):377-91. doi: 10.1109/TBME.1979.326416.

61. Hutchison JC. Hypertension Detection and Compliance: Permanent Site Hypertensive Evaluation—A New Method of Increasing Patient Compliance. Angiology. 1979;30(8):568-76. doi: 10.1177/000331977903000807.

62. Maun PM. Primary OR nursing in outpatient surgery. AORN Journal. 1979;29(7):1231-49. doi: http://doi.org/10.1016/S0001-2092(07)64251-3.

63. Tornello JD. When I build my OR: Dream and reality. AORN Journal. 1979;30(1):44-50. doi: http://doi.org/10.1016/S0001-2092(07)61988-7.

64. Exhibitors describe Congress booths. AORN Journal. 1980;31(3):508-45. doi: http://doi.org/10.1016/S0001-2092(07)61443-4.

65. Johnson F, Sawle GV, Tomlinson DR. An e.c.g. vector simulator to facilitate learning of the basic principles of electrocardiography [proceedings]. J Physiol. 1980;298:1p. Epub 1980/01/01. PubMed PMID: 7359376; PubMed Central PMCID: PMCPMC1279024.

66. Llewelyn DEH, Anderson J. The historical development of the concepts of diagnosis and prognosis and their relationship to probabilistic inference. Informatics Health Soc Care. 1980;5(4):267-80. doi: 10.3109/14639238009001409.

67. Murayama M, Kuroda Y. Cardiovascular future of athletes. Jpn J Phys Fitness Sports Med. 1980;29(2):117-23. doi: 10.7600/jspfsm1949.29.117.

68. Picklo KK. Infection Control in the Hospital. AORN Journal. 1980;31(2):278-9. doi: http://doi.org/10.1016/S0001-2092(07)62370-9.

69. Exhibitors describe Congress booths. AORN Journal. 1981;33(3):538-74. doi: http://doi.org/10.1016/S0001-2092(07)69493-9.

70. Gleim GW, Witman PA, Nicholas JA. Indirect assessment of cardiovascular 'demands' using telemetry on professional football players. AM J SPORTS MED. 1981;9(3):178-83.

71. Gleim GW, Witman PA, Nicholas JA. Indirect assessment of cardiovascular "demands" using telemetry on professional football players. Am J Sports Med. 1981;9(3):178-83. Epub 1981/05/01. doi: 10.1177/036354658100900309. PubMed PMID: 7235115.

72. McManus BM, Graboys TB, Mitchell JH, Siegel RJ, Miller Jr HS, Froelicher VF, et al. Exercise and sudden death- part I. Curr Probl Cardiol. 1981;6(9):6-89. doi: 10.1016/0146-2806(81)90002-5.

73. Wetchler BV. Anesthesia for outpatient surgery. AORN Journal. 1981;34(2):282-96. doi: http://doi.org/10.1016/S0001-2092(07)62243-1.

74. Yen PK. Fractures and diet—What's the relationship? Geriatric Nursing. 1981;2(5):327-78. doi: http://doi.org/10.1016/S0197-4572(81)80002-X.

75. Deregulation of nursing home industry proposed in administration report that calls health rules “wasteful”. Geriatric Nursing. 1982;3(2):72-127. doi: http://doi.org/10.1016/S0197-4572(82)80001-3.

76. Burk J. Simon Farber, a man on a complex regimen. Geriatric Nursing. 1982;3(1):41-3. doi: http://doi.org/10.1016/S0197-4572(82)80079-7.

77. Calvo Aguilar JL, Mahedero Balsera B, Peña Bernal JJ. Simulation of the variable compliance of living systems. Comput Biol Med. 1982;12(2):133-41. doi: 10.1016/0010-4825(82)90021-X.

78. Gatch G. Caring for children needing anesthesia. AORN Journal. 1982;35(2):218-26. doi: http://doi.org/10.1016/S0001-2092(07)68783-3.

79. Heyman S. Correcting transposition of the great arteries. AORN Journal. 1982;36(1):35-44. doi: http://doi.org/10.1016/S0001-2092(07)62043-2.

80. Jelkmann CHM. My heart transplant: A second chance to live. AORN Journal. 1982;36(1):53-6. doi: http://doi.org/10.1016/S0001-2092(07)62046-8.

81. Jung K. Physical exercise therapy in juvenile diabetes mellitus. J SPORTS MED PHYS FITNESS. 1982;22(1):23-31.

82. Lipschultz A. Computerized arrhythmia monitoring systems: A review. J Clin Eng. 1982;7(3):229-34.

83. McManus BM, Waller BF, Graboys TB, Mitchell JH, Siegel RJ, Miller Jr HS, et al. Exercise and sudden death-Part II. Curr Probl Cardiol. 1982;6(10):3-57. doi: 10.1016/0146-2806(82)90014-7.

84. Pratt GT, Larmer P. SPORTS INJURIES AND THEIR MANAGEMENT: WHAT ARE THE RUGBY LEAGUE CLUBS PROVIDING? Australian Journal of Physiotherapy. 1982;28(6):3-7. doi: http://doi.org/10.1016/S0004-9514(14)60778-9.

85. Ricci G, Lajoie D, Petitclerc R, Peronnet F, Ferguson RJ, Fournier M, et al. Left ventricular size following endurance, sprint, and strength training. Med Sci Sports Exerc. 1982;14(5):344-7.

86. Bates RHT, Garden KL, Peters TM. Overview of Computerized Tomography with Emphasis on Future Developments. Proc IEEE. 1983;71(3):356-72. doi: 10.1109/PROC.1983.12594.

87. Eichner ER. Exercise and heart disease: Epidemiology of the “exercise hypothesis”. The American Journal of Medicine. 1983;75(6):1008-23. doi: http://doi.org/10.1016/0002-9343(83)90882-3.

88. Furno GS, Tompkins WJ. A Learning Filter for Removing Noise Interference. IEEE TRANS BIOMED ENG. 1983;BME-30(4):234-5. doi: 10.1109/TBME.1983.325225.

89. Ho KW, Roy RR, Taylor JF, Heusner WW, Van Huss WD. Differential effects of running and weight-lifting on the rat coronary arterial tree. Med Sci Sports Exerc. 1983;15(6):472-7.

90. Little J, Seeger B. The Treatment and Management of Duchenne Muscular Dystrophy at Regency Park Centre, South Australia. Australian Journal of Physiotherapy. 1983;29(5):167-74. doi: http://doi.org/10.1016/S0004-9514(14)60688-7.

91. Pilcher GF, Cook AJ, Johnston BL, Fletcher GF. Twenty-four-hour continuous electrocardiography during exercise and free activity in 80 apparently healthy runners. Am J Cardiol. 1983;52(7):859-61. doi: 10.1016/0002-9149(83)90428-9.

92. Shahein HI. Computers in health-sciences education An application to electrocardiography. Comput Programs Biomed. 1983;17(3):213-23. doi: 10.1016/0010-468X(83)90042-9.

93. Adams RH, Fry DE. Surgical Suite Reconstruction: Infection Control. AORN Journal. 1984;39(5):868-76. doi: http://doi.org/10.1016/S0001-2092(07)64026-5.

94. Balady GJ, Cadigan JB, Ryan TJ. Electrocardiogram of the athlete: An analysis of 289 professional football players. Am J Cardiol. 1984;53(9):1339-43. doi: 10.1016/0002-9149(84)90090-0.

95. Bouchard C, Lortie G. Heredity and Endurance Performance. Sports Med. 1984;1(1):38-64. doi: 10.2165/00007256-198401010-00004.

96. Ferst JA, Chaitman BR. The Electrocardiogram and the Athlete. SPORTS MED. 1984;1(5):390-403. doi: 10.2165/00007256-198401050-00004.

97. Fukushima M, Inoue M, Fukunami M, Ishikawa K, Inada H, Abe H. Computer-assisted education system for arrhythmia (CAESAR). Comput Biomed Res. 1984;17(4):376-88. doi: 10.1016/0010-4809(84)90047-8.

98. Fukushima M, Inoue M, Fukunami M, Ishikawa K, Inada H, Abe H. Computer-assisted education system for arrhythmia (CAESAR). Comput Biomed Res. 1984;17(4):376-88. Epub 1984/08/01. PubMed PMID: 6383704.

99. Gevins AS. Analysis of the Electromagnetic Signals of the Human Brain: Milestones, Obstacles, and Goals. IEEE TRANS BIOMED ENG. 1984;BME-31(12):833-50. doi: 10.1109/TBME.1984.325246.

100. Inbar GF, Noujaim AE. On Surface EMG Spectral Characterization and Its Application to Diagnostic Classification. IEEE TRANS BIOMED ENG. 1984;BME-31(9):597-604. doi: 10.1109/TBME.1984.325303.

101. Jaffe AS, Garfinkel BT, Ritter CS, Sobel BE. Plasma mb creatine kinase after vigorous exercise in professional athletes. Am J Cardiol. 1984;53(6):856-8. doi: 10.1016/0002-9149(84)90419-3.

102. Lie H, Erikssen J. Five‐year Follow‐up of ECG Aberrations, Latent Coronary Heart Disease and Cardiopulmonary Fitness in Various Age Groups of Norwegian Cross‐country Skiers. Acta Med Scand. 1984;216(4):377-83. doi: 10.1111/j.0954-6820.1984.tb03821.x.

103. Magner JA, Rogol AD, Gorden P. Reversible growth hormone deficiency and delayed puberty triggered by a stressful experience in a young adult. The American Journal of Medicine. 1984;76(4):737-42. doi: http://doi.org/10.1016/0002-9343(84)90307-3.

104. Northcote R, Evans AB, Ballantyne D. SUDDEN DEATH IN SQUASH PLAYERS. Lancet. 1984;323(8369):148-51. doi: 10.1016/S0140-6736(84)90073-4.

105. Soni Cassani J, Garcia Moreira C, Kabela E, Gonzalez Beltran C, Mondragon J, Valenzuela F, et al. [Policy guidelines and practical considerations for the development of technology at the Instituto Nacional de Cardiologia de Mexico]. Arch Inst Cardiol Mex. 1984;54(6):527-33. Epub 1984/11/01. PubMed PMID: 6241457.

106. Superko HR, Adams WC, Daly PW. Effects of ozone inhalation during exercise in selected patients with heart disease. The American Journal of Medicine. 1984;77(3):463-70. doi: http://doi.org/10.1016/0002-9343(84)90105-0.

107. Higgs JOY, Wettenhall AP. Designing a Pulmonary Rehabilitation Programme. Australian Journal of Physiotherapy. 1985;31(2):46-56. doi: http://doi.org/10.1016/S0004-9514(14)60618-8.

108. Kurihara S, Konishi M, Kobayashi K, Tomizawa N, Matsune Y, Kawamura T, et al. Effects of swimming on cardiac functions of rats at growing stage. Jpn J Phys Fitness Sports Med. 1985;34:113-20. doi: 10.7600/jspfsm1949.34.113.

109. Palatini P, Maraglino G, Sperti G, Calzavara A, Libardoni M, Pessina AC, et al. Prevalence and possible mechanisms of ventricular arrhythmias in athletes. Am Heart J. 1985;110(3):560-7. doi: 10.1016/0002-8703(85)90075-4.

110. Sharma OP. Sarcoidosis: Clinical, laboratory, and immunologic aspects. Seminars in Roentgenology. 1985;20(4):340-55. doi: http://doi.org/10.1016/0037-198X(85)90042-2.

111. Recommendations for Preventing Transmission of Infection With Human T-Lymphotropic Virus Type III/Lymphadenopathy-Associated Virus in the Workplace. AORN Journal. 1986;43(2):528-43. doi: http://doi.org/10.1016/S0001-2092(07)64177-5.

112. Alford MM. A guide for nutrition educators at the worksite. Journal of Nutrition Education. 1986;18(2, Supplement 1):S19-S21. doi: http://doi.org/10.1016/S0022-3182(86)80087-5.

113. Bullock MI. Overseas Observations of the Physiotherapist in the Work Place. Australian Journal of Physiotherapy. 1986;32(3):151-6. doi: http://doi.org/10.1016/S0004-9514(14)60650-4.

114. Cohen DG, Klopovich P. Growing up differently: An adolescent's perspective. Seminars in Oncology Nursing. 1986;2(2):84-9. doi: http://doi.org/10.1016/0749-2081(86)90015-X.

115. Goodman T. Marfan's Syndrome: A Personal Perspective. AORN Journal. 1986;43(2):452-61. doi: http://doi.org/10.1016/S0001-2092(07)64167-2.

116. Larnard DJ. Simulation of cardiac arrhythmia haemodynamics with a real-time computer model. Med Biol Eng Comput. 1986;24(5):465-70. doi: 10.1007/BF02443960.

117. Lowenstein SR, Crescenzi CA, Kern DC, Steel K. Care of the elderly in the emergency department. Ann Emerg Med. 1986;15(5):528-35. doi: 10.1016/S0196-0644(86)80987-8.

118. Murphy EK. Performance of routine OR nursing activities can be the deciding factor in determining negligence. AORN Journal. 1986;44(5):850-4. doi: http://doi.org/10.1016/S0001-2092(07)65451-9.

119. Posner JD, Gorman KM, Klein HS, Woldow A. Exercise capacity in the elderly. Am J Cardiol. 1986;57(5):C52-C8. doi: 10.1016/0002-9149(86)91027-1.

120. News watch. Geriatric Nursing. 1987;8(4):166-212. doi: http://doi.org/10.1016/S0197-4572(87)80209-4.

121. Adams RH, Applegeet CJ. Managing Conflict: Techniques Managers Can Use. AORN Journal. 1987;46(6):1116-20. doi: http://doi.org/10.1016/S0001-2092(07)69722-1.

122. Cramer C, Renz VR. Preoperative Care Unit: An Alternative to the Holding Room. AORN Journal. 1987;45(2):464-72. doi: http://doi.org/10.1016/S0001-2092(07)68359-8.

123. De Groot G, Hollander AP, Sargeant AJ, Van Ingen Schenau GJ, De Boer RW. Applied physiology of speed skating. J SPORTS SCI. 1987;5(3):249-59. doi: 10.1080/02640418708729780.

124. Duncan HW, Barnard RJ, Grimditch GK, Vinten-Johansen J, Buckberg GD. Cardiovascular response to sudden strenuous exercise. Basic Res Cardiol. 1987;82(3):226-32. doi: 10.1007/BF01906853.

125. Elliot DL, Hickam DH. Evaluation of Physical Examination Skills: Reliability of Faculty Observers and Patient Instructors. JAMA. 1987;258(23):3405-8. doi: 10.1001/jama.1987.03400230065033.

126. Le-Huy P, Yvroud E, Dion J-L, editors. VERSATILE CARDIAC ARRHYTHMIA SIMULATOR. Conference Proceedings - IEEE Instrumentation and Measurement Technology Conference; 1987; New York, NY, USA

Boston, MA, USA: IEEE.

127. Miller G. Exposure Guidelines for Magnetic Fields. American Industrial Hygiene Association Journal. 1987;48(12):957-68. doi: 10.1080/15298668791385903.

128. Nikias CL, Raghuveer MR. Bispectrum Estimation: A Digital Signal Processing Framework. Proc IEEE. 1987;75(7):869-91. doi: 10.1109/PROC.1987.13824.

129. Paiss O, Inbar GF. Autoregressive Modeling of Surface EMG and Its Spectrum with Application to Fatigue. IEEE TRANS BIOMED ENG. 1987;BME-34(10):761-70. doi: 10.1109/TBME.1987.325918.

130. Rao MS. An interpretation of holistic health. British Homoeopathic journal. 1987;76(1):30-41. doi: http://doi.org/10.1016/S0007-0785(87)80037-6.

131. Reisman SS, Friedman KJ, editors. EKG CHALLENGER - A SELF TEACHING EKG COMPUTER PROGRAM. Proceedings of the Ninth Annual Conference of the IEEE Engineering in Medicine and Biology Conference; 1987; New York, NY, USA

Boston, MA, USA: IEEE.

132. Winickoff RN, Murphy PK. The Persistent Problem of Poor Blood Pressure Control. Arch Intern Med. 1987;147(8):1393-6. doi: 10.1001/archinte.1987.00370080029007.

133. Index to volume 9. Geriatric Nursing. 1988;9(6):366-8. doi: http://doi.org/10.1016/S0197-4572(88)80074-0.

134. Calendar of events. The American Journal of Medicine. 1988;85(6):A19-A48. doi: http://doi.org/10.1016/S0002-9343(88)80015-9.

135. Proposed Recommended Practices: Traffic Patterns in the Surgical Suite. AORN Journal. 1988;47(4):1002-5. doi: http://doi.org/10.1016/S0001-2092(07)66555-7.

136. Courses and Conferences. Physiotherapy. 1988;74(12):626-33. doi: http://doi.org/10.1016/S0031-9406(10)62904-5.

137. Courses and Conferences. Physiotherapy. 1988;74(10):510-9. doi: http://doi.org/10.1016/S0031-9406(10)63395-0.

138. Eaton SB, Konner M, Shostak M. Stone agers in the fast lane: Chronic degenerative diseases in evolutionary perspective. The American Journal of Medicine. 1988;84(4):739-49. doi: http://doi.org/10.1016/0002-9343(88)90113-1.

139. Fogg DM. Proper surgical scrub technique; need for staff development coordinator; laundering surgical attire at home. AORN Journal. 1988;48(1):18-24. doi: http://doi.org/10.1016/S0001-2092(07)67435-3.

140. Gaba DM, DeAnda A. A comprehensive anesthesia simulation environment: re-creating the operating room for research and training. ANESTHESIOLOGY. 1988;69(3):387-94.

141. Gaba DM, DeAnda A. A comprehensive anesthesia simulation environment: re-creating the operating room for research and training. ANESTHESIOLOGY. 1988;69(3):387-94. Epub 1988/09/01. PubMed PMID: 3415018.

142. Meyer RA, Hagler D, Huhta J, Smallhorn J, Snider R, Williams R. Guidelines for Physician Training in Pediatric Echocardiography: Recommendations of the Society of Pediatric Echocardiography Committee on Physician Training. Journal of the American Society of Echocardiography. 1988;1(4):285-6. doi: http://doi.org/10.1016/S0894-7317(88)80047-6.

143. Schwid HA. Electrocardiogram simulation using a personal computer. Comput Biomed Res. 1988;21(6):562-9. doi: 10.1016/0010-4809(88)90012-2.

144. Courses and conferences. Physiotherapy. 1989;75(8):465-78. doi: http://doi.org/10.1016/S0031-9406(10)62645-4.

145. Haskell WL, Brachfeld N, Bruce RA, Davis PO, Dennis CA, Fox Iii SM, et al. Task force II: Determination of occupational working capacity in patients with ischemic heart disease. J Am Coll Cardiol. 1989;14(4):1025-34. doi: 10.1016/0735-1097(89)90485-3.

146. Kapoor WN. Syncope with abrupt termination of exercise. The American Journal of Medicine. 1989;87(5):597-9. doi: http://doi.org/10.1016/S0002-9343(89)80626-6.

147. Lin KP, Chang WH. QRS Feature Extraction Using Linear Prediction. IEEE TRANS BIOMED ENG. 1989;36(10):1050-5. doi: 10.1109/10.40806.

148. McIlvaine WB. Perioperative pain management in children: A review. Journal of Pain and Symptom Management. 1989;4(4):215-29. doi: http://doi.org/10.1016/0885-3924(89)90045-6.

149. Morokhoev VI. [Play olfactometry for children]. Vestn Otorinolaringol. 1989;(5):43-5. Epub 1989/09/01. PubMed PMID: 2588414.

150. Paulanka BJ, Dunnington SF. From life care to professional care. Geriatric Nursing. 1989;10(6):288-9. doi: http://doi.org/10.1016/S0197-4572(89)80103-X.

151. Schiel U. Abstractions in semantic networks: axiom schemata for generalization, aggregation and grouping. SIGART Bull. 1989;(107):25-6. doi: 10.1145/65751.65752.

152. Spitzer K, Stein V, Thie A, Kunze K. NERVTRACK-A neuroanatomical data bank. Comput Biomed Res. 1989;22(2):181-9. doi: 10.1016/0010-4809(89)90024-4.

153. Walz MJ. Opening an outpatient facility. Conducting a mock run. Aorn j. 1989;50(3):583-5. Epub 1989/09/01. PubMed PMID: 2774543.

154. Walz MJA. Opening an Outpatient Facility: Conducting a Mock Run. AORN Journal. 1989;50(3):583-5. doi: http://doi.org/10.1016/S0001-2092(07)62124-3.

155. Yen PK. Tummy trouble. Geriatric Nursing. 1989;10(6):301. doi: http://doi.org/10.1016/S0197-4572(89)80110-7.

156. Zonneveld FW, Lobregt S, van der Meulen JCH, Vaandrager JM. Three-dimensional imaging in craniofacial surgery. World J Surg. 1989;13(4):328-42. doi: 10.1007/BF01660745.

157. Notices. Intensive Care Nursing. 1990;6(4):214. doi: http://doi.org/10.1016/0266-612X(90)90034-5.

158. Index to Subjects. Journal of the American Society of Echocardiography. 1990;3(6):511-8. doi: http://doi.org/10.1016/S0894-7317(14)80371-4.

159. Baxter R. 12 lead ECG interpretation: The self-assessment Approach: W. B. Saunders &amp; Company, Philadelphia 1989, ISBN 7216 2846X 304 pages. Price £13.50. Intensive Care Nursing. 1990;6(4):213-4. doi: http://doi.org/10.1016/0266-612X(90)90033-4.

160. Coast DA, Cano GG, Briller SA. Use of hidden markov models for electrocardiographic signal analysis. J Electrocardiol. 1990;23(SUPPL.):184-91. doi: 10.1016/0022-0736(90)90099-N.

161. Coast DA, Stern RM, Cano GG, Briller SA. An Approach to Cardiac Arrhythmia Analysis using Hidden Markov Models. IEEE TRANS BIOMED ENG. 1990;37(9):826-36. doi: 10.1109/10.58593.

162. Corrado D, Thiene G, Nava A, Rossi L, Pennelli N. Sudden death in young competitive athletes: clinicopathologic correlations in 22 cases. The American Journal of Medicine. 1990;89(5):588-96. doi: http://doi.org/10.1016/0002-9343(90)90176-E.

163. Ferdjallah M, Barr RE. Frequency-domain digital filtering techniques for the removal of powerline noise with application to the electrocardiogram. Comput Biomed Res. 1990;23(5):473-89. doi: 10.1016/0010-4809(90)90035-B.

164. Hackett PH, Roach RC. High altitude pulmonary edema. Journal of Wilderness Medicine. 1990;1(1):3-26. doi: http://doi.org/10.1580/0953-9859-1.1.3.

165. Jalaleddine SMS, Hutchens CG, Strattan RD, Coberly WA. ECG Data Compression Techniques—A Unified Approach. IEEE TRANS BIOMED ENG. 1990;37(4):329-43. doi: 10.1109/10.52340.

166. Montpetit RR. Applied Physiology of Squash. SPORTS MED. 1990;10(1):31-41. doi: 10.2165/00007256-199010010-00004.

167. Murphy EK. Precounted cesarean section kits; private tissue procurement; surgery without nurses. AORN Journal. 1990;52(3):622-6. doi: http://doi.org/10.1016/S0001-2092(07)69890-1.

168. Ness TJ, Gebhart GF. Visceral pain: a review of experimental studies. Pain. 1990;41(2):167-234. doi: http://doi.org/10.1016/0304-3959(90)90021-5.

169. Shannon B, Mullis RM, Pirie PL, Pheley AM. Promoting better nutrition in the grocery store using a game format: The shop smart game project. Journal of Nutrition Education. 1990;22(4):183-8. doi: http://doi.org/10.1016/S0022-3182(12)80923-X.

170. Zehender M, Meinertz T, Keul J, Just H. ECG variants and cardiac arrhythmias in athletes: Clinical relevance and prognostic importance. Am Heart J. 1990;119(6):1378-91. doi: 10.1016/S0002-8703(05)80189-9.

171. It's whether you play the game. Geriatric Nursing. 1991;12(1):9. doi: http://doi.org/10.1016/S0197-4572(06)80308-3.

172. Courses and conferences. Physiotherapy. 1991;77(10):686-96. doi: http://doi.org/10.1016/S0031-9406(10)60436-1.

173. Ali A, Farrally M. Recording soccer players’ heart rates during matches. J SPORTS SCI. 1991;9(2):183-9. doi: 10.1080/02640419108729879.

174. Ali A, Farrally M. Recording soccer players' heart rates during matches. J Sports Sci. 1991;9(2):183-9. Epub 1991/01/01. doi: 10.1080/02640419108729879. PubMed PMID: 1895354.

175. Bjørnstad H, Slorstein L, Meen HD, Hals O. Electrocardiographic findings in athletic students and sedentary controls. Cardiology. 1991;79(4):290-305. doi: 10.1159/000174893.

176. Jack RAF. Seven successful clinical cases. British Homoeopathic journal. 1991;80(2):101-7. doi: http://doi.org/10.1016/S0007-0785(05)80393-X.

177. Moens HJB, van der Korst JK. Computer-assisted diagnosis of rheumatic disorders. Semin Arthritis Rheum. 1991;21(3):156-69. doi: 10.1016/0049-0172(91)90004-J.

178. Oosterom AV. Mathematical aspects of source modeling. Acta Oto-Laryngol. 1991;111(S491):70-9. doi: 10.3109/00016489109136783.

179. Pollak MH. Heart rate reactivity to laboratory tasks and ambulatory heart rate in daily life. Psychosom Med. 1991;53(1):25-35. Epub 1991/01/01. PubMed PMID: 2011648.

180. Schels HF, Haberl R, Jilge G, Steinbigler P, Steinbeck G. Frequency Analysis of the Electrocardiogram with Maximum Entropy Method for Identification of Patients with Sustained Ventricular Tachycardia. IEEE TRANS BIOMED ENG. 1991;38(9):821-6. doi: 10.1109/10.83601.

181. Schmaus D. Evaluating computer-assisted instructional software for the OR. AORN Journal. 1991;54(6):1296-301. doi: http://doi.org/10.1016/S0001-2092(07)66882-3.

182. Squires RW, Miller TD, Harn T, Micheels TA, Palma TA. Transtelephonic electrocardiographic monitoring of cardiac rehabilitation exercise sessions in coronary artery disease. Am J Cardiol. 1991;67(11):962-4. doi: 10.1016/0002-9149(91)90168-K.

183. Storstein L, Bjørnstad H, Hals O, Meen ID. Electrocardiographic findings according to sex in athletes and controls. Cardiology. 1991;79(3):227-36. doi: 10.1159/000174882.

184. Thomsen CE, Rosenfalck A, Christensen KN. Assessment of anaesthetic depth by clustering analysis and autoregressive modelling of electroencephalograms. COMPUT METHODS PROGRAMS BIOMED. 1991;34(2-3):125-38. doi: 10.1016/0169-2607(91)90038-U.

185. Volpicello C. Bone Cyst of the Acetabulum: A Case Study. AORN Journal. 1991;54(2):291-9. doi: http://doi.org/10.1016/S0001-2092(07)69291-6.

186. Windyga P, Almeida D, Passariello G, Mora-Ciangherotti FA, Coatrieux JL. Knowledge-based approach to the management of serious arrhythmia in the CCU. Med Biol Eng Comput. 1991;29(3):254-60. doi: 10.1007/BF02446707.

187. Courses and Conferences. Physiotherapy. 1992;78(9):698-716. doi: http://doi.org/10.1016/S0031-9406(10)61594-5.

188. Courses and Conferences. Physiotherapy. 1992;78(1):53-67. doi: http://doi.org/10.1016/S0031-9406(10)61355-7.

189. Courses and Conferences. Physiotherapy. 1992;78(7):530-47. doi: http://doi.org/10.1016/S0031-9406(10)61174-1.

190. Subject index volume 93. The American Journal of Medicine. 1992;93(6):711-21. doi: http://doi.org/10.1016/0002-9343(92)90221-V.

191. Résumés des communications et des posters de la réunion scientifique Paris, 16–18 janvier 1992. Réanimation Urgences. 1992;1(1):87-152. doi: http://doi.org/10.1016/S1164-6756(05)80427-X.

192. Bernhardt JH. Non-ionizing radiation safety: Radiofrequency radiation, electric and magnetic fields. Physics in Medicine and Biology. 1992;37(4):807-44. doi: 10.1088/0031-9155/37/4/001.

193. Bresnitz EA, Gracely EJ, Rubenstein HL. A randomized trial to evaluate a computer-based learning program in occupational lung disease. J Occup Med. 1992;34(4):422-7.

194. Hashimoto F, Appenzeller O, Abrams J, Qualls C. Ambulatory electrocardiographic monitoring at high altitude. Journal of Wilderness Medicine. 1992;3(4):358-66. doi: http://doi.org/10.1580/0953-9859-3.4.358.

195. Iezzoni LI, Daley J. A Description and Clinical Assessment of the Computerized Severity Index™. QRB - Quality Review Bulletin. 1992;18(2):44-52. doi: http://doi.org/10.1016/S0097-5990(16)30506-1.

196. Larkin KT, Zayfert C, Abel JL, Veltum LG. Reducing heart rate reactivity to stress with feedback. Generalization across task and time. Behav Modif. 1992;16(1):118-31. Epub 1992/01/01. doi: 10.1177/01454455920161006. PubMed PMID: 1540120.

197. Pearlman AS, Gardin JM, Martin RP, Parisi AF, Popp RL, Quinones MA, et al. Guidelines for Physician Training in Transesophag. eal Echocardiography: Recommendations of the American Society of Echocardiography Committee for Physician Training in Echocardiography. Journal of the American Society of Echocardiography. 1992;5(2):187-94. doi: http://doi.org/10.1016/S0894-7317(14)80552-X.

198. Sandige RS, Ferris CD, Bhaskaran A. Electronic ECG simulator. BIOMED SCI INSTRUM. 1992;28:021-5.

199. Sedgwick ML, Watson J, Dalziel K, Carrington DJ, Cobbe SM. Efficacy of out of hospital defibrillation by ambulance technicians using automated external defibrillators. The Heartstart Scotland Project. Resuscitation. 1992;24(1):73-87. doi: 10.1016/0300-9572(92)90175-C.

200. Stratos C, Stefanadis C, Kallikazaros I, Boudoulas H, Toutouzas P. Ascending aorta distensibility abnormalities in hypertensive patients and response to nifedipine administration. The American Journal of Medicine. 1992;93(5):505-12. doi: http://doi.org/10.1016/0002-9343(92)90577-X.

201. Continuing Education and Meeting Calendar. Journal of the American Society of Echocardiography. 1993;6(6):21A-31A. doi: http://doi.org/10.1016/S0894-7317(14)80170-3.

202. Continuing Education and Meeting Calendar. Journal of the American Society of Echocardiography. 1993;6(4):19A-31A. doi: http://doi.org/10.1016/S0894-7317(14)80232-0.

203. Poster session. Air Medical Journal. 1993;12(9):344-51. doi: http://doi.org/10.1016/S1067-991X(05)80082-3.

204. Product &amp; service guide. Air Medical Journal. 1993;12(5):125-50. doi: http://doi.org/10.1016/S1067-991X(05)80251-2.

205. Abstract Session A ASE Young Investigator Research Award. Journal of the American Society of Echocardiography. 1993;6(3, Part 2):S7-S40. doi: http://doi.org/10.1016/S0894-7317(14)80408-2.

206. Booth Descriptions. AORN Journal. 1993;57(1):290-308. doi: http://doi.org/10.1016/S0001-2092(07)68426-9.

207. Résumés des communications orales et des posters de la réunion scientifique Paris, 20–22 janvier 1994. Réanimation Urgences. 1993;2(6):641-732. doi: http://doi.org/10.1016/S1164-6756(05)80347-0.

208. Annobil SH. Scorpion stings in children in the Asir Province of Saudi Arabia. Journal of Wilderness Medicine. 1993;4(3):241-51. doi: http://doi.org/10.1580/0953-9859-4.3.241.

209. Bjørnstad H, Storstein L, Meen HD, Hals O. Electrocardiographic findings of heart rate and conduction times in athletic students and sedentary control subjects. Cardiology. 1993;83(4):258-67. doi: 10.1159/000175979.

210. Burg MM, Jain D, Soufer R, Kerns RD, Zaret BL. Role of behavioral and psychological factors in mental stress-induced silent left ventricular dysfunction in coronary artery disease. J Am Coll Cardiol. 1993;22(2):440-8. doi: 10.1016/0735-1097(93)90048-6.

211. Çetin AE, Köymen H, Aydin MC. Multichannel ECG Data Compression by Multirate Signal Processing and Transform Domain Coding Techniques. IEEE TRANS BIOMED ENG. 1993;40(5):495-9. doi: 10.1109/10.243411.

212. Ciaccio EJ, Dunn SM, Akay M. Biosignal Pattern Recognition And Interpretation Systems. IEEE Eng Med Biol Mag. 1993;12(4):106-13. doi: 10.1109/51.248173.

213. Fontes CK, Miles PC. New Technology Goes to Taiwan: Perioperative Nurses Make International Journey with New Technology. AORN Journal. 1993;58(5):913-26. doi: http://doi.org/10.1016/S0001-2092(07)65629-4.

214. Frayne R, Rutt BK. Frequency response of retrospectively gated phase‐contrast MR imaging: Effect of interpolation. J Magn Reson Imaging. 1993;3(6):907-17. doi: 10.1002/jmri.1880030619.

215. Grappa E, Bonvecchio A, Zucco P, editors. Functional and technical evaluation of the skier by a specific ergometer. 9th International Symposium on Skiing and Safety; 1993; Philadelphia, PA, United States

Thredbo, Aust: Publ by ASTM.

216. Häggman H. Accident and Emergency in a shipping line. Accident and Emergency Nursing. 1993;1(2):104-7. doi: http://doi.org/10.1016/0965-2302(93)90057-7.

217. Hecht HS, DeBord L, Sotomayor N, Shaw R, Dunlap R, Ryan C. Supine Bicycle Stress Echocardiography: Peak Exercise Imaging is Superior to Postexercise Imaging. Journal of the American Society of Echocardiography. 1993;6(3, Part 1):265-71. doi: http://doi.org/10.1016/S0894-7317(14)80062-X.

218. Johnson DA, Roethig-Johnston K, Richards D. Biochemical and physiological parameters of recovery in acute severe head injury: Responses to multisensory stimulation. Brain Inj. 1993;7(6):491-9. doi: 10.3109/02699059309008176.

219. Knox AM. performing endotracheal suction on children: a literature review and implications for nursing practice. Intensive and Critical Care Nursing. 1993;9(1):48-54. doi: http://doi.org/10.1016/0964-3397(93)90009-M.

220. Mora FA, Passariello G. Intelligent Patient Monitoring and Management ystems: A Review. IEEE Eng Med Biol Mag. 1993;12(4):23-33. doi: 10.1109/51.248164.

221. Parenti C, Lurie N. Are things different in the light of day? A time study of internal medicine house staff days. The American Journal of Medicine. 1993;94(6):654-8. doi: http://doi.org/10.1016/0002-9343(93)90220-J.

222. Secher NH. Physiological and Biomechanical Aspects of Rowing: Implications for Training. Sports Med. 1993;15(1):24-42. doi: 10.2165/00007256-199315010-00004.

223. Speers AT. Games in nursing staff development. J Nurs Staff Dev. 1993;9(6):274-7.

224. Calendar. Air Medical Journal. 1994;13(10):449-53. doi: http://doi.org/10.1016/S1067-991X(05)80067-7.

225. 1994 Buyer's guide. Air Medical Journal. 1994;13(5):167-200. doi: http://doi.org/10.1016/S1067-991X(05)80108-7.

226. Belgrade M. Two decades after ping-pong diplomacy: Is there a role for acupuncture in American pain medicine? APS Journal. 1994;3(2):73-83. doi: http://doi.org/10.1016/S1058-9139(05)80329-8.

227. Bentley PM, McDonnell JTE. Wavelet tranforms: An introduction. Electron Commun Eng J. 1994;6(4):175-86.

228. Bjørnstad H, Storstein L, Dyre Meen H, Hals O. Electrocardiographic findings of repolarization in athletic students and control subjects. Cardiology. 1994;84(1):51-60. doi: 10.1159/000176328.

229. Chopra V, Engbers FH, Geerts MJ, Filet WR, Bovill JG, Spierdijk J. The Leiden anaesthesia simulator. Br J Anaesth. 1994;73(3):287-92. Epub 1994/09/01. PubMed PMID: 7946850.

230. Chopra V, Engbers FHM, Geerts MJ, Filet WR, Bovill JG, Spierdijk J. The Leiden anaesthesia simulator. BR J ANAESTH. 1994;73(3):287-92.

231. Coia LR. Chemoradiation: A superior alternative for the primary management of esophageal carcinoma. Seminars in Radiation Oncology. 1994;4(3):157-64. doi: http://doi.org/10.1016/S1053-4296(05)80063-3.

232. Cullum N. Medication teaching aids: Springhouse 1993 378pp illus ISBN: 0-8743-4512-X (pb) £26.95. Nurse Education Today. 1994;14(3):251-2. doi: http://doi.org/10.1016/0260-6917(94)90101-5.

233. Derkay CS, Lefebvre SM, George MRST. Retrieving Foreign Bodies from Upper Aerodigestive Tracts of Children. AORN Journal. 1994;60(1):53-66. doi: http://doi.org/10.1016/S0001-2092(07)69664-1.

234. Dubin S, Butler A, Hanes D. EKGenius: a computer-interactive electrophysiology learning tool. Biomed Sci Instrum. 1994;30:133-40. Epub 1994/01/01. PubMed PMID: 7948625.

235. Dubin S, Butler A, Hans D. EKGenius: A computer interactive electrophysiology learning tool. BIOMED SCI INSTRUM. 1994;30:133-40.

236. Johnson KF. Does an On-site Satellite Laboratory Reduce Surgical Delays?: A Study of Delays in a Same Day Surgical Center. AORN Journal. 1994;59(6):1275-90. doi: http://doi.org/10.1016/S0001-2092(07)64893-5.

237. Lafferty FW, Fiske ME. Postmenopausal estrogen replacement: A long-term cohort study. The American Journal of Medicine. 1994;97(1):66-77. doi: http://doi.org/10.1016/0002-9343(94)90050-7.

238. Miller RA. Medical diagnostic decision support systems- Past, present, and future: A threaded bibliography and brief commentary. J Am Med Informatics Assoc. 1994;1(1):8-27.

239. Murphy EK. Are perioperative nurses ‘borrowed servants’? Are surgeons ‘captains of the ship’? AORN Journal. 1994;60(3):474-7. doi: http://doi.org/10.1016/S0001-2092(07)62783-5.

240. Rotstein A, Sagiv M, Ben-Sira D, Werber G, Hutzler J, Annenburg H. Aerobic capacity and anaerobic threshold of wheelchair basketball players. Paraplegia. 1994;32(3):196-201. doi: 10.1038/sc.1994.36.

241. Santiano N, Daffurn K, Lee A. The Basic Knowledge Assessment Tool: Is It Useful? Australian Critical Care. 1994;7(4):18-23. doi: http://doi.org/10.1016/S1036-7314(94)70698-6.

242. Smith CJ. Preparing Nurses to Monitor Patients Receiving Local Anesthesia: Using the Decision-Making Process. AORN Journal. 1994;59(5):1033-41. doi: http://doi.org/10.1016/S0001-2092(07)65513-6.

243. Speers AT. Crossword puzzles: A teaching strategy for critical care nursing. Dimensions Crit Care Nurs. 1994;13(1):52-5.

244. Studdy SJ, Nicol MJ, Fox-Hiley A. Teaching and learning clinical skills, Part 1 — Development of a multidisciplinary skills centre. Nurse Education Today. 1994;14(3):177-85. doi: http://doi.org/10.1016/0260-6917(94)90079-5.

245. Timpka T, Rauch E, Nyce JM. Towards productive Knowledge-Based Systems in clinical organizations: A methods perspective. Artif Intell Med. 1994;6(6):501-19. doi: 10.1016/0933-3657(94)90027-2.

246. Tuttle MS, Nelson SJ. The role of the UMLS in 'storing' and 'sharing' across systems. Int J Bio-Med Comput. 1994;34(1-4):207-37. doi: 10.1016/0020-7101(94)90023-X.

247. Webber WB, Summers AN, Rinehart GC. Computer-based multimedia in plastic surgery education. Plast Reconstr Surg. 1994;93(6):1290-300.

248. Whitworth J. The direction of medical research in Australia. Collegian. 1994;1(1):26-8. doi: http://doi.org/10.1016/S1322-7696(08)60575-2.

249. Zelcer J, Scott DA. Simulators for training in anaesthesia. Bailliere's Clin Anaesthesiol. 1994;8(3):625-35. doi: 10.1016/S0950-3501(05)80125-4.

250. Congress registration, AORN services, planning calendar, posters, pre-Congress and Congress sessions: Sunday, March 3, to Friday, March 8, 1996. AORN Journal. 1995;62(4):612-35. doi: http://doi.org/10.1016/S0001-2092(06)63503-5.

251. Résumés Des Communications Orales Et Des Posters De La Réunion Scientifique Paris, 17–19 janvier 1996. Réanimation Urgences. 1995;4(6):673-767. doi: http://doi.org/10.1016/S1164-6756(05)80136-7.

252. Clavario P, Copello F, Giugliano M, Martinengo E, Biagini A, Damanti D, et al., editors. Use of an arrhythmia simulator for the evaluation of nurses' knowledge and as a teaching tool. Proceedings of the 1995 Conference on Computers in Cardiology; 1995; Los Alamitos, CA, United States

Vienna, Austria: IEEE.

253. Green JS, Crouse SF. The effects of endurance training on functional capacity in the elderly: A meta-analysis. Med Sci Sports Exerc. 1995;27(6):920-6.

254. Hunter TB. Tubes, lines, catheters, and other interesting devices. Curr Probl Diagn Radiol. 1995;24(2):55-108. doi: 10.1016/S0363-0188(95)80002-6.

255. LaCombe MA. Innocent bystander. The American Journal of Medicine. 1995;98(5):507-8. doi: http://doi.org/10.1016/S0002-9343(99)80353-2.

256. LaCombe MA. What is it patients want? The American Journal of Medicine. 1995;99(6):588-9. doi: http://doi.org/10.1016/S0002-9343(99)80242-3.

257. London MJ, Ahlstrom LD. Validation testing of the SpaceLabs PC2 ST-segment analyzer. J Cardiothorac Vasc Anesth. 1995;9(6):684-93. doi: 10.1016/S1053-0770(05)80230-7.

258. Mackenzie CF, Hu PF, Horst RL, Group L. An audio-video system for automated data acquisition in the clinical environment. J Clin Monitor Comput. 1995;11(5):335-41. doi: 10.1007/BF01616993.

259. Nerlich S. Computer-Assisted Learning (CAL) for General and Specialist Nursing Education. Australian Critical Care. 1995;8(3):23-7. doi: http://doi.org/10.1016/S1036-7314(95)70286-7.

260. Siregar P, Chahine M, Lemoulec F, Le Beux P. An interactive qualitative model in cardiology. Comput Biomed Res. 1995;28(6):443-78. doi: 10.1006/cbmr.1995.1029.

261. Stephens C, Beehan S, Hillman K. Preparation of the Critically III for Transfer in Metropolitan Sydney. Australian Critical Care. 1995;8(4):14-8. doi: http://doi.org/10.1016/S1036-7314(95)70293-4.

262. Tattersall AJ, Hockey GR. Level of operator control and changes in heart rate variability during simulated flight maintenance. Hum Factors. 1995;37(4):682-98. Epub 1995/12/01. doi: 10.1518/001872095778995517. PubMed PMID: 8851773.

263. Weisenberg M, Tepper I, Schwarzwald J. Humor as a cognitive technique for increasing pain tolerance. PAIN®. 1995;63(2):207-12. doi: http://doi.org/10.1016/0304-3959(95)00046-U.

264. Recent literature on A &amp; E nursing. Accident and Emergency Nursing. 1996;4(1):B1-B5. doi: http://doi.org/10.1016/S0965-2302(96)90048-X.

265. Résumés des communications orales et des posters de la réunion scientifique Paris, 22–24 janvier 1997. Réanimation Urgences. 1996;5(6):741-837. doi: http://doi.org/10.1016/S1164-6756(05)80607-3.

266. Aitkenhead AR. Risk reduction in anaesthesia: is improved monitoring the answer? Current Anaesthesia & Critical Care. 1996;7(6):302-8. doi: http://doi.org/10.1016/S0953-7112(96)80051-2.

267. Arne R, Stale F, Ragna K, Petter L. PatSim--simulator for practising anaesthesia and intensive care. Development and observations. Int J Clin Monit Comput. 1996;13(3):147-52. Epub 1996/08/01. PubMed PMID: 8912028.

268. Arne R, Ståle F, Ragna K, Petter L. PatSim-Simulator for practising anaesthesia and intensive care - Development and observations. J Clin Monit Comput. 1996;13(3):147-52. doi: 10.1023/A:1016964810485.

269. Bradie B. Wavelet packet-based compression of single lead ECG. IEEE TRANS BIOMED ENG. 1996;43(5):493-501. doi: 10.1109/10.488797.

270. Burke LJ. Securing life through technology acceptance: The first six months after transvenous internal cardioverter defibrillator implantation. Heart & Lung: The Journal of Acute and Critical Care. 1996;25(5):352-66. doi: http://doi.org/10.1016/S0147-9563(96)80077-X.

271. Cerutti S, Carrault G, Cluitmans PJM, Kinie A, Lipping T, Nikolaidis N, et al. Non-linear algorithms for processing biological signals. COMPUT METHODS PROGRAMS BIOMED. 1996;51(1-2):51-73. doi: 10.1016/0169-2607(96)01762-2.

272. Clarke T, Abbenbroek B, Hardy L. The impact of a high dependency unit continuing education program on nursing practice and patient outcomes. Australian Critical Care. 1996;9(4):138-49. doi: http://doi.org/10.1016/S1036-7314(96)70371-5.

273. Dyson RJ, Buchanan M, Farrington TA, Hurrion PD. Electromyographic activity during windsurfing on water. J SPORTS SCI. 1996;14(2):125-30.

274. Keravnou ET. Temporal diagnostic reasoning based on time-objects. Artif Intell Med. 1996;8(3):235-65. doi: 10.1016/0933-3657(95)00035-6.

275. King PH, Blanks ST, Rummel DM, Patterson D. Simulator training in anesthesiology: An answer? BIOMED INSTRUM TECHNOL. 1996;30(4):341-5.

276. Laguna P, Jané R, Masgrau E, Caminal P. The adaptive linear combiner with a periodic-impulse reference input as a linear comb filter. Signal Process. 1996;48(3):193-203. doi: 10.1016/0165-1684(95)00135-2.

277. Larbig W, Montoya P, Flor H, Bilow H, Weller S, Birbaumer N. Evidence for a change in neural processing in phantom limb pain patients. Pain. 1996;67(2–3):275-83. doi: http://doi.org/10.1016/0304-3959(96)03107-7.

278. LeFebvre SM. Take Our Daughters to Work Day is an excellent way to promote perioperative nursing. AORN Journal. 1996;64(5):800-3. doi: http://doi.org/10.1016/S0001-2092(06)63268-7.

279. Lewis CK. The clinical nurse specialist's role as coach in a clinical practice development model. Journal of Vascular Nursing. 1996;14(2):48-52. doi: http://doi.org/10.1016/S1062-0303(96)80016-1.

280. McBee MJ. Pocket guide to ED orientation. Journal of Emergency Nursing. 1996;22(5):446-50. doi: http://doi.org/10.1016/S0099-1767(96)80174-7.

281. Sheedy Ii PF, Johnson CM, Welch TJ, Stanson AW, Breen JF, Maus TP. Fast CT for pulmonary embolus. Seminars in Ultrasound, CT and MRI. 1996;17(4):324-38. doi: http://doi.org/10.1016/S0887-2171(96)90020-7.

282. Tiller WA, McCraty R, Atkinson M. Cardiac coherence: A new, noninvasive measure of autonomic nervous system order. Altern Ther Health Med. 1996;2(1):52-65.

283. Wang C, Ohe K, Sakurai T, Nagase T, Kaihara S. Object-oriented analysis and design of an ECG storage and retrieval system integrated with an HIS. METHODS INF MED. 1996;35(1):35-40.

284. Session one: The challenges and opportunities of providing maternity care through collaborative practice. Women's Health Issues. 1997;7(5):309-42. doi: http://doi.org/10.1016/S1049-3867(97)00053-4.

285. Xes journées d'enseignement des infirmiers et infirmières de l'urgence 2es journées des assistantes sociales. Réanimation Urgences. 1997;6(2):119-54. doi: http://doi.org/10.1016/S1164-6756(97)80161-2.

286. Résumés des communications orales et affichées de la réunion scientifique 21–23 janvier 1998 cnit, place de la défense, 92090 Paris-La Défense cedex 26. Réanimation Urgences. 1997;6(6):709-820. doi: http://doi.org/10.1016/S1164-6756(97)80074-6.

287. Badgett RG, Lucey CR, Mulrow CD. Can the clinical examination diagnose left-sided heart failure in adults? J AM MED ASSOC. 1997;277(21):1712-9.

288. Ball C. Planning for the future: advanced nursing practice in critical care. Intensive and Critical Care Nursing. 1997;13(1):17-25. doi: http://doi.org/10.1016/S0964-3397(97)80673-8.

289. Bower JO. Using patient simulators to train surgical team members. AORN Journal. 1997;65(4):805-8. doi: http://doi.org/10.1016/S0001-2092(06)63003-2.

290. Brender J. Methodology for assessment of medical IT-based systems - In an organisational context. Stud Health Technol Informatics. 1997;42:1-307. doi: 10.3233/978-1-60750-886-1-i.

291. Brown LH, Gough JE, Hawley CR. Accuracy of rural EMS provider interpretation of three-lead ECG rhythm strips. Prehospital Emergency Care. 1997;1(4):259-62. doi: 10.1080/10903129708958820.

292. Brown LH, Gough JE, Hawley CR. Accuracy of rural EMS provider interpretation of three-lead ECG rhythm strips. Prehosp Emerg Care. 1997;1(4):259-62. Epub 1998/08/26. PubMed PMID: 9709367.

293. Castiglia PT. Attention deficit/hyperactivity disorder. Journal of Pediatric Health Care. 1997;11(3):130-3. doi: http://doi.org/10.1016/S0891-5245(97)90065-1.

294. Chimiak WJ, Rainer RO, Chimiak JM, Martinez R. An architecture for naval telemedicine. IEEE Trans Inf Technol Biomed. 1997;1(1):73-9.

295. Collins D. The prehospital 12-lead EKG: Starting outside the emergency department. Journal of Emergency Nursing. 1997;23(1):48-50. doi: http://doi.org/10.1016/S0099-1767(97)90062-3.

296. Damian F, Atkinson CC, Bouchard A, Harrington S, Powers T. Disaster relief efforts after hurricane Marilyn: A pediatric team's experience in St. Thomas. Journal of Emergency Nursing. 1997;23(6):545-9. doi: http://doi.org/10.1016/S0099-1767(97)90267-1.

297. Delprado A. The lessons from Atlanta. Australian Emergency Nursing Journal. 1997;1(2):48-51. doi: http://doi.org/10.1016/S1328-2743(97)80034-X.

298. Drury T, Zacharias S. Integrating nursing education into a trauma outreach program. International Journal of Trauma Nursing. 1997;3(3):83-7. doi: http://doi.org/10.1016/S1075-4210(97)90034-1.

299. Ferrara-Love R. The product page. Journal of PeriAnesthesia Nursing. 1997;12(2):127-33. doi: http://doi.org/10.1016/S1089-9472(97)80028-7.

300. Forsström J. Why certification of medical software would be useful? International Journal of Medical Informatics. 1997;47(3):143-51. doi: http://doi.org/10.1016/S1386-5056(97)00098-1.

301. Geissbühler A, Miller RA. Desiderata for product labeling of medical expert systems. International Journal of Medical Informatics. 1997;47(3):153-63. doi: http://doi.org/10.1016/S1386-5056(97)00099-3.

302. Hauber C, Sharp RL, Franke WD. Heart rate response to submaximal and maximal workloads during running and swimming. INT J SPORTS MED. 1997;18(5):347-53. doi: 10.1055/s-2007-972644.

303. Johnson CR. Computational and numerical methods for bioelectric field problems. CRIT REV BIOMED ENG. 1997;25(1):1-81.

304. Kim WY, Poulsen JK, Terp K, Sloth E, Paulsen PK. New semiautomated Doppler method for quantification of volumetric flow: Intraoperative validation with multiplane transesophageal color Doppler imaging. Journal of the American Society of Echocardiography. 1997;10(4):330-6. doi: http://doi.org/10.1016/S0894-7317(97)70069-5.

305. Lin Z, De Chen JZ. Advances in time-frequency analysis of biomedical signals. CRIT REV BIOMED ENG. 1997;24(1):1-72.

306. Locke S, Colquhoun D, Briner M, Ellis L, O'Brien M, Wollstein J, et al. Squash racquets: A review of physiology and medicine. SPORTS MED. 1997;23(2):130-8.

307. Long WJ, Fraser H, Naimi S. Reasoning requirements for diagnosis of heart disease. Artif Intell Med. 1997;10(1):5-24. doi: 10.1016/S0933-3657(97)00381-3.

308. McGinty P. Helpful websites. International Journal of Trauma Nursing. 1997;3(3):99-100. doi: http://doi.org/10.1016/S1075-4210(97)90038-9.

309. Nathwani BN, Clarke K, Lincoln T, Berard C, Taylor C, Patil R, et al. Evaluation of an expert system on lymph node pathology. HUM PATHOL. 1997;28(9):1097-110. doi: 10.1016/S0046-8177(97)90065-4.

310. Newsham KR. Exertional chest pain in an intercollegiate athlete. J Athl Train. 1997;32(1):59-62.

311. Öhrn MAK, Van Oostrom JH, Van Meurs WL. A comparison of traditional textbook and interactive computer learning of neuromuscular block. ANESTH ANALG. 1997;84(3):657-61. doi: 10.1097/00000539-199703000-00035.

312. Papadimitriou S, Gatzounas D, Papadopoulos V, Tzigounis V, Bezerianos A. Denoising of the fetal heart rate signal with non-linear filtering of the wavelet transform maxima. International Journal of Medical Informatics. 1997;44(3):177-92. doi: http://doi.org/10.1016/S1386-5056(97)00019-1.

313. Payton RG, Gardner R, Reynolds D. Pharmacologic considerations and management of common endocrine disorders in women. Journal of Nurse-Midwifery. 1997;42(3):186-206. doi: http://doi.org/10.1016/S0091-2182(97)00024-4.

314. Ramakrishnan AG, Saha S. ECG coding by wavelet-based linear prediction. IEEE TRANS BIOMED ENG. 1997;44(12):1253-61. doi: 10.1109/10.649997.

315. Reiffel Md JA. The Sicilian Gambit: Does It Help the Clinician? ACC Current Journal Review. 1997;6(5):32-4. doi: http://doi.org/10.1016/S1062-1458(97)00080-9.

316. Secola R. Pediatric blood cell transplantation. Seminars in Oncology Nursing. 1997;13(3):184-93. doi: http://doi.org/10.1016/S0749-2081(97)80034-4.

317. Swami A, Giannakis GB, Zhou G. Bibliography on higher-order statistics. Signal Process. 1997;60(1):65-126. doi: 10.1016/S0165-1684(97)00065-0.

318. Takashina T, Shimizu M, Katayama H. A new cardiology patient simulator. CARDIOLOGY. 1997;88(5):408-13.

319. Takashina T, Shimizu M, Katayama H. A new cardiology patient simulator. CARDIOLOGY. 1997;88(5):408-13. Epub 1997/09/01. PubMed PMID: 9286501.

320. Tougas G, Kamath M, Watteel G, Fitzpatrick D, Fallen EL, Hunt RH, et al. Modulation of neurocardiac function by oesophageal stimulation in humans. CLIN SCI. 1997;92(2):167-74.

321. Ward R. Implications ofcomputer networking and the internet for nurse education. Nurse Education Today. 1997;17(3):178-83. doi: http://doi.org/10.1016/S0260-6917(97)80130-X.

322. Weinberger MW, Carnes M. Diagnosis and management of delirium. Primary Care Update for OB/GYNS. 1997;4(3):80-6. doi: http://doi.org/10.1016/S1068-607X(97)00005-X.

323. Sentinel Events: Approaches to Error Reduction and Prevention. The Joint Commission Journal on Quality Improvement. 1998;24(4):175-86. doi: http://doi.org/10.1016/S1070-3241(16)30370-4.

324. Subject index. Current Anaesthesia & Critical Care. 1998;9(6):332-9. doi: http://doi.org/10.1016/S0953-7112(98)80024-0.

325. Subject index. Journal of PeriAnesthesia Nursing. 1998;13(6):428-42. doi: http://doi.org/10.1016/S1089-9472(98)80016-6.

326. Treatment trends. The Case Manager. 1998;9(6):18-21. doi: http://doi.org/10.1016/S1061-9259(98)80131-8.

327. 17th National ASPANConference Abstracts. Journal of PeriAnesthesia Nursing. 1998;13(3):207-14. doi: http://doi.org/10.1016/S1089-9472(98)80052-X.

328. Résumés des communications orales et affichées de la réunion scientifique. Réanimation Urgences. 1998;7, Supplement 1:s49-s158. doi: http://doi.org/10.1016/S1164-6756(99)80082-6.

329. Abt G, Zhou S, Weatherby R. The Effect of a High-Carbohydrate Diet on the Skill Performance of Midfield Soccer Players after Intermittent Treadmill Exercise. Journal of Science and Medicine in Sport. 1998;1(4):203-12. doi: http://doi.org/10.1016/S1440-2440(09)60003-7.

330. Althoff KD, Bergmann R, Wess S, Manago M, Auriol E, Larichev OI, et al. Case-based reasoning for medical decision support tasks: The Inreca approach. Artif Intell Med. 1998;12(1):25-41. doi: 10.1016/S0933-3657(97)00038-9.

331. Bauer MD, Huynh MV. Nursing students' blood pressure measurement following CD-ROM and conventional classroom instruction: A pilot study. Int J Med Inform. 1998;50(1-3):103-9. doi: 10.1016/S1386-5056(98)00072-0.

332. Bell MDD, Bodenham AR. Problems and pitfalls of practical procedures: a medico-legal perspective. Current Anaesthesia & Critical Care. 1998;9(6):278-89. doi: http://doi.org/10.1016/S0953-7112(98)80015-X.

333. Carver J. The perceptions of registered nurses on role expansion. Intensive and Critical Care Nursing. 1998;14(2):82-90. doi: http://doi.org/10.1016/S0964-3397(98)80226-7.

334. Chopra V. Simulators in anaesthesia. Current Anaesthesia & Critical Care. 1998;9(4):187-92. doi: http://doi.org/10.1016/S0953-7112(98)80053-7.

335. Christensen UJ, Heffernan D, Andersen SF, Jensen PF. ResusSim 98 - A PC advanced life support trainer. Resuscitation. 1998;39(1-2):81-4. doi: 10.1016/S0300-9572(98)00107-5.

336. Daponte P, Grimaldi D. Artificial neural networks in measurements. Meas J Int Meas Confed. 1998;23(2):93-115.

337. Devitt P, Worthley S, Palmer E, Cehic D. Evaluation of a computer based package on electrocardiography. Aust New Zealand J Med. 1998;28(4):432-5.

338. Devitt P, Worthley S, Palmer E, Cehic D. Evaluation of a computer based package on electrocardiography. Aust New Zealand J Med [Internet]. 1998; 28(4):[432-5 pp.]. Available from: http://onlinelibrary.wiley.com/o/cochrane/clcentral/articles/792/CN-00155792/frame.html.

339. Devitt P, Worthley S, Palmer E, Cehic D. Evaluation of a computer based package on electrocardiography. Aust N Z J Med. 1998;28(4):432-5. Epub 1998/10/20. PubMed PMID: 9777109.

340. Fürnkranz J, Pfahringer B. Guest editorial: first-order knowledge discovery in databases. Appl Artif Intell. 1998;12(5):345-61. doi: 10.1080/088395198117677.

341. Gamrath B, Del Monte L, Richards K. Noninvasive pacing: What you should know. Journal of Emergency Nursing. 1998;24(3):223-32. doi: http://doi.org/10.1016/S0099-1767(98)90062-9.

342. Gardner MB, Holden MK, Leikauskas JM, Richard RL. Partial body weight support with treadmill locomotion to improve gait after incomplete spinal cord injury: A single-subject experimental design. Phys Ther. 1998;78(4):361-74.

343. Hewitt-Taylor J. Paediatric intensive care nursing education by distance learning: an example from Canada. Intensive and Critical Care Nursing. 1998;14(3):137-43. doi: http://doi.org/10.1016/S0964-3397(98)80387-X.

344. Horio H, Murakami M, Chiba Y, Inada H. Fetal monitor for non-stress-test screening at home. BIOMED INSTRUM TECHNOL. 1998;32(1):39-47.

345. Jones S, Elliott PM, Sharma S, McKenna WJ, Whipp BJ. Cardiopulmonary responses to exercise in patients with hypertrophic cardiomyopathy. Heart. 1998;80(1):60-7.

346. Konstantaki M, Trowbridge EA, Swaine IL. The relationship between blood lactate and heart rate responses to swim bench exercise and women's competitive water polo. J SPORTS SCI. 1998;16(3):251-6. doi: 10.1080/026404198366777.

347. Kontodimopoulos N, Pallikarakis N, Christov I, Daskalov I. In-house development of test equipment for quality control and training. Case study: a prototype ECG simulator-tester. Med Eng Phys. 1998;20(10):717-21. Epub 1999/05/01. PubMed PMID: 10223639.

348. Laguna P, Moody GB, Mark RG. Power spectral density of unevenly sampled data by least-square analysis: Performance and application to heart rate signals. IEEE TRANS BIOMED ENG. 1998;45(6):698-715. doi: 10.1109/10.678605.

349. Leven FJ, Haux R. Twenty five years of medical informatics education at Heidelberg/Heilbronn: discussion of a specialized curriculum for medical informatics. International Journal of Medical Informatics. 1998;50(1–3):31-42. doi: http://doi.org/10.1016/S1386-5056(98)00048-3.

350. Lindberg E. Snoring and Sleep Apnea: A Study of Evolution and Consequences in a Male Population - Minireview based on a doctoral thesis. Upsala J Med Sci. 1998;103(3):155-202.

351. Nicol S, Narkowicz C. Learning physiology from cardiac surgery patients. Am J Physiol. 1998;274(6 PART 2):S74-S83.

352. Rainwater JA, Romano PS, Antonius DM. The California Hospital Outcomes Project: How Useful Is California’s Report Card for Quality Improvement? The Joint Commission Journal on Quality Improvement. 1998;24(1):31-9. doi: http://doi.org/10.1016/S1070-3241(16)30357-1.

353. Roelandt JRTC. Three-dimensional echocardiography: The future today! Acta Cardiol. 1998;53(6):323-36.

354. Roelandt JRTC, Yao J, Kasprzak JD. Three-dimensional echocardiography. Curr Opin Cardiol. 1998;13(6):386-96.

355. Rohde MM, Bement SL, Lupa RS. ECG Boy: Low-cost medical instrumentation using mass-produced, hand- held entertainment computers: A preliminary report. BIOMED INSTRUM TECHNOL. 1998;32(5):497-508.

356. Rontoyannis GP, Stalikas A, Sarros G, Vlastaris A. Medical, morphological and functional aspects of Greek football referees. J SPORTS MED PHYS FITNESS. 1998;38(3):208-14.

357. Rose K. Deterministic annealing for clustering, compression, classification, regression, and related optimization problems. Proc IEEE. 1998;86(11):2210-39. doi: 10.1109/5.726788.

358. Smith HK. Applied physiology of water polo. SPORTS MED. 1998;26(5):317-34.

359. Standen P. Managing triage with uniformity and individualised care — is it possible? Australian Emergency Nursing Journal. 1998;1(5):8-10. doi: http://doi.org/10.1016/S1328-2743(98)80005-9.

360. Steinhagen MR, Meyers MC, Erickson HH, Noble L, Richardson MT. Physiological profile of college club-sport lacrosse athletes. J Strength Cond Res. 1998;12(4):226-31.

361. Tillinghast SJ. Can Western Quality Improvement Methods Transform the Russian Health Care System? The Joint Commission Journal on Quality Improvement. 1998;24(5):280-98. doi: http://doi.org/10.1016/S1070-3241(16)30381-9.

362. van Bemmel JH, Duisterhout JS. Education and training of medical informatics in the medical curriculum. International Journal of Medical Informatics. 1998;50(1–3):49-58. doi: http://doi.org/10.1016/S1386-5056(98)00050-1.

363. Wilson EM. The weakest link in the chain of survival? Journal of Emergency Nursing. 1998;24(1):69-70. doi: http://doi.org/10.1016/S0099-1767(98)90177-5.

364. CEU Test. Journal of Emergency Nursing. 1999;25(5):432-6. doi: http://doi.org/10.1016/S0099-1767(99)70108-X.

365. XXIIIe Congrès de la Société de réanimation de la langue franç. Réanimation Urgences. 1999;8, Supplement 3:125s-214s. doi: http://doi.org/10.1016/S1164-6756(00)87592-1.

366. Adelmann HG. Design of a PC-based system for time-domain and spectral analysis of heart rate variability. Comput Biomed Res. 1999;32(1):77-92. doi: 10.1006/cbmr.1998.1502.

367. Balogh D. Enhancing medical students’ clinical skills. Australian Critical Care. 1999;12(2):80. doi: http://doi.org/10.1016/S1036-7314(99)70570-9.

368. Bar-Mor G, Zeevi B, Yaaron M, Falk B. Use of the heart rate monitor to modulate physical activity in adolescents with congenital aortic stenosis: An innovative approach. Journal of Pediatric Nursing. 1999;14(4):273-7. doi: http://doi.org/10.1016/S0882-5963(99)80026-8.

369. Bradley V. WWW: Something for everyone. Journal of Emergency Nursing. 1999;25(2):139-41. doi: http://doi.org/10.1016/S0099-1767(99)70162-5.

370. Calvelo D, Chambrin MC, Pomorski D, Vilhelm C, editors. Decision support using machine learning: Towards intensive care unit patient state characterization. 1999 European Control Conference, ECC 1999; 1999: Institute of Electrical and Electronics Engineers Inc.

371. Felici F, Rodio A, Madaffari A, Ercolani L, Marchetti M. The cardiovascular work of competitive dinghy sailing. J SPORTS MED PHYS FITNESS. 1999;39(4):309-14.

372. Ferriell KM. Invasive Lines in the Emergency Department. Journal of Emergency Nursing. 1999;25(5):403-14. doi: http://doi.org/10.1016/S0099-1767(99)90000-4.

373. Forsström JJ, Rigby M. Considerations on the quality of medical software and information services. International Journal of Medical Informatics. 1999;56(1–3):169-76. doi: http://doi.org/10.1016/S1386-5056(99)00044-1.

374. Gopalsamy C, Park S, Rajamanickam R, Jayaraman S. The wearable motherboard™: The first generation of adaptive and responsive textile structures (ARTS) for medical applications. Virtual Reality. 1999;4(3):152-68.

375. Haag M, Maylein L, Leven FJ, Tönshoff B, Haux R. Web-based training: A new paradigm in computer-assisted instruction in medicine. International Journal of Medical Informatics. 1999;53(1):79-90. doi: 10.1016/S1386-5056(98)00118-X.

376. Henriksen E, Landelius J, Kangro T, Jonason T, Hedberg P, Wesslén L, et al. An echocardiographic study of right and left ventricular adaptation to physical exercise in elite female orienteers. Eur Heart J. 1999;20(4):309-16. doi: 10.1053/euhj.1998.1197.

377. Heron RJ. Partnerships and educational benefits in postgraduate education. Australian Critical Care. 1999;12(2):80-1. doi: http://doi.org/10.1016/S1036-7314(99)70571-0.

378. Karlsson S, Yu J, Akay M. Enhancement of spectral analysis of myoelectric signals during static contractions using wavelet methods. IEEE TRANS BIOMED ENG. 1999;46(6):670-84. doi: 10.1109/10.764944.

379. Lean T. Cardiothoracic intensive care introductory program. Australian Critical Care. 1999;12(2):80. doi: http://doi.org/10.1016/S1036-7314(99)70569-2.

380. Lilly KT, Langley VL. The Perioperative Nurse and the Organ Donation Experience. AORN Journal. 1999;69(4):779-91. doi: http://doi.org/10.1016/S0001-2092(06)62351-X.

381. Price RR. The AAPM/RSNA physics tutorial for residents: MR imaging safety considerations. Radiographics. 1999;19(6):1641-51.

382. Price RR. The AAPM/RSNA physics tutorial for residents. MR imaging safety considerations. Radiological Society of North America. Radiographics. 1999;19(6):1641-51. Epub 1999/11/11. doi: 10.1148/radiographics.19.6.g99no331641. PubMed PMID: 10555679.

383. Reddy S, Young B, Xue Q, Taha B, Brodnick D, Steinberg J. Review of methods to predict and detect atrial fibrillation in post- cardiac surgery patients. J Electrocardiol. 1999;32(SUPPL.):23-8.

384. Sabry-Rizk M, Zgallai W. Higher order statistics are indispensable tools in the analysis of electrocardiogram signals. IEE Colloq Dig. 1999;(2):39-45.

385. Salzbach R. Pediatric Septic Arthritis. AORN Journal. 1999;70(6):986-1002. doi: http://doi.org/10.1016/S0001-2092(06)62205-9.

386. Sharma S, Whyte G, Elliott P, Padula M, Kaushal R, Mahon N, et al. Electrocardiographic changes in 1000 highly trained junior elite athletes. Br J Sports Med. 1999;33(5):319-24.

387. Signorini MG, Cerutti S. Nonlinear properties of cardiovascular time series. Stud Health Technol Informatics. 1999;60:73-89. doi: 10.3233/978-1-60750-904-2-73.

388. Takeda H, Minato K, Takahasi T. High quality image oriented telemedicine with multimedia technology. International Journal of Medical Informatics. 1999;55(1):23-31. doi: http://doi.org/10.1016/S1386-5056(99)00017-9.

389. Thomas JJ, Vander Wyk S, Boyer J. Contrasting occupational forms: Effects on performance and affect in patients undergoing phase II cardiac rehabilitation. Occup Ther J Res. 1999;19(3):187-202.

390. Whyte G, Sharma S, George K, McKenna WJ. Alterations in cardiac morphology and function in elite multi-disciplinary athletes. INT J SPORTS MED. 1999;20(4):222-6. doi: 10.1055/s-2007-971121.

391. Wieben O, Afonso VX, Tompkins WJ. Classification of premature ventricular complexes using filter bank features, induction of decision trees and a fuzzy rule-based system. Med Biol Eng Comput. 1999;37(5):560-5.

392. Yao X. Evolving artificial neural networks. Proc IEEE. 1999;87(9):1423-47. doi: 10.1109/5.784219.

393. Continuing Education Test. Journal of Emergency Nursing. 2000;26(1):88-92. doi: http://doi.org/10.1016/S0099-1767(00)90028-X.

394. 19th National ASPAN Conference Abstracts. Journal of PeriAnesthesia Nursing. 2000;15(3):202-13. doi: http://doi.org/10.1016/S1089-9472(00)80024-6.

395. Ballora M, Pennycook B, Ivanov PC, Goldberger A, Glass L, editors. Detection of obstructive sleep apnea through auditory display of heart rate variability. Computers in Cardiology 2000; 2000; Los Alamitos, CA, United States

Cambridge, MA, USA: IEEE.

396. Banja J. State and federal regulation of health care: Friend or foe? The Case Manager. 2000;11(2):34-9. doi: http://doi.org/10.1016/S1061-9259(00)80038-7.

397. Brohet C. Clinical value of vectorcardiography, Holter monitoring and quantitative electrocardiology. Acta Cardiol. 2000;55(3):157-62.

398. Cotin S, Dawson SL, Meglan D, Shaffer DW, Ferrell MA, Bardsley RS, et al., editors. ICTS, an interventional cardiology training system. 8th Annual Meeting of Medicine Meets Virtual Reality, MMVR 2000; 2000; Newport Beach, CA2000137400.

399. Cotin S, Dawson SL, Meglan D, Shaffer DW, Ferrell MA, Bardsley RS, et al. ICTS, an interventional cardiology training system. Stud Health Technol Inform. 2000;70:59-65. Epub 2000/09/08. PubMed PMID: 10977584.

400. Haji-Michael PG, Vincent JL, Degaute JP, Van De Borne P. Power spectral analysis of cardiovascular variability in critically ill neurosurgical patients. Crit Care Med. 2000;28(7):2578-83.

401. Hauer K, Niebauer J, Weiss C, Marburger C, Hambrecht R, Schlierf G, et al. Myocardial ischemia during physical exercise in patients with stable coronary artery disease: Predictability and prevention. Int J Cardiol. 2000;75(2-3):179-86. doi: 10.1016/S0167-5273(00)00321-1.

402. Jones NB, Spurgeon SK, Pont MJ, Twiddle JA, Lim CL, Parikh CR, et al. Aspects of diagnostic schemes for biomedical and engineering systems. IEE Proc Sci Meas Technol. 2000;147(6):357-62. doi: 10.1049/ip-smt:20000859.

403. Kundu M, Nasipuri M, Basu DK. Knowledge-based ECG interpretation: A critical review. Pattern Recogn. 2000;33(3):351-73.

404. Lyttle V. The computer: An educational adjunct. Journal of Emergency Nursing. 2000;26(4):374-6. doi: http://doi.org/10.1067/men.2000.108155.

405. Millet-Roig J, Ventura-Galiano R, Chorro-Gascó FJ, Cebrián A, editors. Support vector machine for arrhythmia discrimination with wavelet-transform-based feature selection. Computers in Cardiology 2000; 2000; Los Alamitos, CA, United States

Cambridge, MA, USA: IEEE.

406. Montgomery H. Cardiac reserve: Linking physiology and genetics. Intensive Care Med Suppl. 2000;26(1):S137-S44.

407. Mulvagh SL, DeMaria AN, Feinstein SB, Burns PN, Kaul S, Miller JG, et al. Contrast Echocardiography: Current and Future Applications. Journal of the American Society of Echocardiography. 2000;13(4):331-42. doi: http://doi.org/10.1067/mje.2000.105462.

408. Parkkari J, Natri A, Kannus P, Mänttäri A, Laukkanen R, Haapasalo H, et al. A controlled trial of the health benefits of regular walking on a golf course∗. The American Journal of Medicine. 2000;109(2):102-8. doi: http://doi.org/10.1016/S0002-9343(00)00455-1.

409. Peña-Reyes CA, Sipper M. Evolutionary computation in medicine: An overview. Artif Intell Med. 2000;19(1):1-23. doi: 10.1016/S0933-3657(99)00047-0.

410. Pien LC. Appropriate use of second-generation antihistamines. Clevel Clin J Med. 2000;67(5):372-80.

411. Popek C, Schaeffer R. An adult male with facial swelling, erythema, and sensation of arm tightness. Journal of Emergency Nursing. 2000;26(6):633-4. doi: http://doi.org/10.1067/men.2000.110197.

412. Rechichi C, Dawson B, Lawrence SR. A multistage shuttle swim test to assess aerobic fitness in competitive water polo players. Journal of Science and Medicine in Sport. 2000;3(1):55-64. doi: http://doi.org/10.1016/S1440-2440(00)80048-1.

413. Rechichi C, Dawson B, Lawrence SR. A multistage shuttle swim test to assess aerobic fitness in competitive water polo players. Journal of Science and Medicine in Sport. 2000;3(1):55-64.

414. Revill SM, Morgan MDL. Biological quality control for exercise testing. Thorax. 2000;55(1):63-6. doi: 10.1136/thorax.55.1.63.

415. Smith AM, Stuart MJ, Fish KN, editors. Psychological, physiological, and performance variables in goalies during hockey games. 3rd Symposium on Ice Hockey 'Safety in Ice Hockey'; 2000; Conshohocken, PA, United States

St. Louis, MO, USA: ASTM.

416. Snyder O. A 71-year-old man with syncope, respiratory distress, and hypotension. Journal of Emergency Nursing. 2000;26(5):522-3. doi: http://doi.org/10.1067/men.2000.110010.

417. Stamkopoulos T, Maglaveras N, Bamidis PD, Pappas C, editors. Wave segmentation using nonstationary properties of ECG. Computers in Cardiology 2000; 2000; Los Alamitos, CA, United States

Cambridge, MA, USA: IEEE.

418. Terenzi C. The triage game. Journal of Emergency Nursing. 2000;26(1):66-9. doi: http://doi.org/10.1016/S0099-1767(00)90021-7.

419. Wang GJ, Volkow ND, Fowler JS, Franceschi D, Logan J, Pappas NR, et al. PET studies of the effects of aerobic exercise on human striatal dopamine release. J Nucl Med. 2000;41(8):1352-6.

420. Continuing education test. Journal of Emergency Nursing. 2001;27(4):406-11. doi: http://doi.org/10.1016/S0099-1767(01)70022-0.

421. 2001 Congress information and highlights: Sunday, March 11, to Thursday, March 15, 2001. AORN Journal. 2001;73(2):320-34. doi: http://doi.org/10.1016/S0001-2092(06)61976-5.

422. Airmed conference abstract 2000. Air Medical Journal. 2001;20(2):23-31. doi: http://doi.org/10.1016/S1067-991X(01)70090-9.

423. Allen TL, Delbridge TR, Stevens MH, Nicholas D. Intubation success rates by air ambulance personnel during 12- versus 24-hour shifts: Does fatigue make a difference? Prehospital Emergency Care. 2001;5(4):340-3. doi: http://doi.org/.

424. Barold SS, Cantens F. Characterization of the 16 blanking periods of the Medtronic GEM DR dual chamber defibrillators. J Intervent Card Electrophysiol. 2001;5(3):319-25. doi: 10.1023/A:1011477002235.

425. Barold SS, Cantens F. Characterization of the 16 blanking periods of the Medtronic GEM DR dual chamber defibrillators. J Interv Card Electrophysiol. 2001;5(3):319-25. Epub 2001/08/14. PubMed PMID: 11500587.

426. Chizner MA, O'Rourke RA. Foreword: Current Problems in Cardiology. Curr Probl Cardiol. 2001;26(5):288-379.

427. Clutter P. An effective, fun annual emergency competency day/skills fair: St John's 2-year experience. Journal of Emergency Nursing. 2001;27(5):500-2. doi: http://doi.org/10.1067/men.2001.118679.

428. Cooper RA, Fitzgerald SG, Boninger ML, Brienza DM, Shapcott N, Cooper R, et al. Telerehabilitation: Expanding access to rehabilitation expertise. Proc IEEE. 2001;89(8):1174-90. doi: 10.1109/5.940286.

429. Coutinho BD, Fry ME, Pollard JK. Web-controlled instrumentation for educational applications. IEE Colloq Dig. 2001;(46 I):31-5.

430. Fletcher GF, Balady GJ, Amsterdam EA, Chaitman B, Eckel R, Fleg J, et al. Exercise standards for testing and training: A statement for healthcare professionals from the American Heart Association. Circulation. 2001;104(14):1694-740.

431. Gerig HJ, Heidegger T, Ulrich B, Grossenbacher R, Kreienbuehl G. Fiberoptically-guided insertion of transtracheal catheters. ANESTH ANALG. 2001;93(3):663-6.

432. Goldman L. Key challenges confronting internal medicine in the early twenty-first century. The American Journal of Medicine. 2001;110(6):463-70. doi: http://doi.org/10.1016/S0002-9343(01)00649-0.

433. Hajdukiewicz JR, Vicente KJ, Doyle DJ, Milgram P, Burns CM. Modeling a medical environment: an ontology for integrated medical informatics design. International Journal of Medical Informatics. 2001;62(1):79-99. doi: http://doi.org/10.1016/S1386-5056(01)00128-9.

434. Karczmarewicz S, Janusek D, Buczkowski T, Gutkowski R, Kulakowski P. Influence of mobile phones on accuracy of ECG interpretation algorithm in automated external defibrillator. Resuscitation. 2001;51(2):173-7. doi: 10.1016/S0300-9572(01)00406-3.

435. Karczmarewicz S, Janusek D, Buczkowski T, Gutkowski R, Kulakowski P. Influence of mobile phones on accuracy of ECG interpretation algorithm in automated external defibrillator. Resuscitation. 2001;51(2):173-7. Epub 2001/11/24. PubMed PMID: 11718973.

436. Kohn MA, Newman TB. What white blood cell count should prompt antibiotic treatment in a febrile child? Tutorial on the importance of disease likelihood to the interpretation of diagnostic tests. Med Decis Mak. 2001;21(6):479-89. doi: 10.1177/02729890122062839.

437. Langdeau JB, Blier L, Turcotte H, O'Hara G, Boulet LP. Electrocardiographic findings in athletes: The prevalence of left ventricular hypertrophy and conduction defects. Canadian Journal of Cardiology. 2001;17(6):655-9.

438. Lees PJ, Chiarugi F, Lombardi D, Chronaki CE, Tsiknakis M, Orphanoudakis SC. Simulator of patient traffic in a cardiology department for testing the integration of an ECG management system with an existing clinical database. Comput Cardiol. 2001:197-200. doi: 10.1109/CIC.2001.977625.

439. Mangum SS. Learning perioperative nursing in Guatemala. AORN Journal. 2001;74(5):706-11. doi: http://doi.org/10.1016/S0001-2092(06)61770-5.

440. Nakao M, Komori M, Matsuda T, Takahashi T, editors. 4D visible and palpable simulation using dynamic pressure model based on cardiac morphology. 2001 Medicine Meets Virtual Reality Conference: Outer Space, Inner Space, Virtual Space, MMVR 2001; 2001; Newport Beach, CA2001138103.

441. Nathanson LA, Safran C, McClennen S, Goldberger AL. ECG Wave-Maven: a self-assessment program for students and clinicians. Proc AMIA Symp. 2001:488-92. Epub 2002/02/05. PubMed PMID: 11825236; PubMed Central PMCID: PMCPMC2243537.

442. Popeseu S, editor Training the children wavelets to recognise waveforms within non-stationary signals. 6th International Symposium on Signal Processing and Its Applications, ISSPA 2001; 2001; Kuala Lumpur: IEEE Computer Society.

443. Redfern J, Ellis E, Holmes W. The use of a pressure manometer enhances student physiotherapists' performance during manual hyperinflation. Australian Journal of Physiotherapy. 2001;47(2):121-31. doi: http://doi.org/10.1016/S0004-9514(14)60303-2.

444. Reims H, Fossum E, Kjeldsen SE, Julius S. Home blood pressure monitoring. Current knowledge and directions for future research. Blood Press. 2001;10(5-6):271-87. doi: 10.1080/080370501753400584.

445. Silipo R, Vergassola R, Zong W, Berthold MR. Knowledge-based and data-driven models in arrhythmia fuzzy classification. METHODS INF MED. 2001;40(5):397-402.

446. Smith NT, Greenberg JE, editors. Design of a module for teaching/learning spectral analysis. 2001 ASEE Annual Conference and Exposition: Peppers, Papers, Pueblos and Professors; 2001; Albuquerque, NM.

447. Stasiu RK, De Britto J, Dias JDS, Scalabrin E. Teaching of electrocardiogram interpretation guided by a tutorial expert. Proc IEEE Symp Comput Based Med Syst. 2001:487-92. doi: 10.1109/CBMS.2001.941766.

448. Sternberg WF, Boka C, Kas L, Alboyadjia A, Gracely RH. Sex-Dependent Components of the Analgesia Produced by Athletic Competition. The Journal of Pain. 2001;2(1):65-74. doi: http://doi.org/10.1054/jpai.2001.18236.

449. Sternberger C, Meyer L. Hypermedia-assisted instruction: Authoring with learning guidelines. Comput Nurs. 2001;19(2):69-74.

450. Sutton AJ, Abrams KR. Bayesian methods in meta-analysis and evidence synthesis. Stat Methods Med Res. 2001;10(4):277-303. doi: 10.1191/096228001678227794.

451. Swoveland B, Medvick C, Kirsh M, Thompson GK, Nuss D. The Nuss Procedure for Pectus Excavatum Correction. AORN Journal. 2001;74(6):827-41. doi: http://doi.org/10.1016/S0001-2092(06)61501-9.

452. Walsh GM, Annunziato L, Frossard N, Knol K, Levander S, Nicolas JM, et al. New insights into the second generation antihistamines. Drugs. 2001;61(2):207-36.

453. Young-McCaughan S, Miaskowski C. Measurement of opioid-induced sedation. Pain Management Nursing. 2001;2(4):132-49. doi: http://doi.org/10.1053/jpmn.2001.25169.

454. Earn 6.0 contact hours by reading the journal and taking this post test. Journal of Emergency Nursing. 2002;28(6):599-604. doi: http://doi.org/10.1016/S0099-1767(02)70014-7.

455. Abbod MF, Linkens DA, Mahfouf M, Dounias G. Survey on the use of smart and adaptive engineering systems in medicine. Artif Intell Med. 2002;26(3):179-209. doi: 10.1016/S0933-3657(02)00083-0.

456. Atwal S, Porter J, MacDonald P. Cardiovascular effects of strenuous exercise in adult recreational hockey: The hockey heart study. CMAJ. 2002;166(3):303-7.

457. Atwal S, Porter J, MacDonald P. Cardiovascular effects of strenuous exercise in adult recreational hockey: the Hockey Heart Study. CMAJ. 2002;166(3):303-7. Epub 2002/03/01. PubMed PMID: 11868637; PubMed Central PMCID: PMCPMC99308.

458. Baselli G, Caiani E, Porta A, Montano N, Signorini MG, Cerutti S. Biomedical signal processing and modeling in cardiovascular systems. CRIT REV BIOMED ENG. 2002;30(1-3):55-84. doi: 10.1615/CritRevBiomedEng.v30.i123.40.

459. Biffi A, Pelliccia A, Verdile L, Fernando F, Spataro A, Caselli S, et al. Long-term clinical significance of frequent and complex ventricular tachyarrhythmias in trained athletes. J Am Coll Cardiol. 2002;40(3):446-52. doi: 10.1016/S0735-1097(02)01977-0.

460. Bilsborough W, O'Driscoll G, Stanton K, Weerasooriya R, Dembo L, Taylor R, et al. Effect of lowering tumour necrosis factor-α on vascular endothelial function in Type II diabetes. CLIN SCI. 2002;103(2):163-9.

461. Bond WF, Spillane L. The use of simulation for emergency medicine resident assessment. Acad Emerg Med. 2002;9(11):1295-9. doi: 10.1197/aemj.9.11.1295.

462. Bowers EJ, Langley P, Drinnan MJ, Allen J, Smith FE, Murray A, editors. Simulation of cardiac RR interval time series. Computers in Cardiology 2002; 2002; Memphis, TN.

463. Chiarugi F, Lombardi D, Lees PJ, Chronaki CE, Tsiknakis M, Orphanoudakis SC. Support of daily ECG procedures in a cardiology department via the integration of an existing clinical database and a commercial ECG management system. Ann Noninvasive Electrocardiol. 2002;7(3):263-70.

464. Chizner MA. The diagnosis of heart disease by clinical assessment alone. Dis Mon. 2002;48(1):5-98.

465. Coatrieux JL. Signal processing and physiological modeling - Part II: Depth model-driven analysis. CRIT REV BIOMED ENG. 2002;30(1-3):37-54. doi: 10.1615/CritRevBiomedEng.v30.i123.30.

466. Corlin RF. The secrets of gun violence in America: What we don't know is killing us. International Journal of Trauma Nursing. 2002;8(2):42-7. doi: http://doi.org/10.1067/mtn.2002.123415.

467. Cosman PH, Cregan PC, Martin CJ, Cartmill JA. Virtual reality simulators: Current status in acquisition and assessment of surgical skills. ANZ J Surg. 2002;72(1):30-4. doi: 10.1046/j.1445-2197.2002.02293.x.

468. Duenas R. United States Chiropractic Practice Acts and Institute of Medicine defined primary care practice. Journal of Chiropractic Medicine. 2002;1(4):155-70. doi: http://doi.org/10.1016/S0899-3467(07)60030-5.

469. Grunkemeier GL, Payne N. Bayesian analysis: A new statistical paradigm for new technology. Ann Thorac Surg. 2002;74(6):1901-8. doi: 10.1016/S0003-4975(02)04535-6.

470. Halberg F, Cornélissen G, Wall D, Otsuka K, Halberg J, Katinas G, et al. Engineering and governmental challenge: 7-Day/24-hour chronobiologic blood pressure and heart rate screening: Part I. BIOMED INSTRUM TECHNOL. 2002;36(2):89-122.

471. Halberg F, Cornélissen G, Wall D, Otsuka K, Halberg J, Katinas G, et al. Engineering and governmental challenge: 7-Day/24-hour chronobiologic blood pressure and heart rate screening: Part II. BIOMED INSTRUM TECHNOL. 2002;36(3):183-97.

472. Haraldsson H, Ohlsson M, Edenbrandt L. Value of exercise data for the interpretation of myocardial perfusion SPECT. J Nucl Cardiol. 2002;9(2):169-73. doi: 10.1067/mnc.2002.120161.

473. Hightower S, Carmon M, Minick P. A qualitative descriptive study of the lived experiences of school-aged children with epilepsy. Journal of Pediatric Health Care. 2002;16(3):131-7. doi: http://doi.org/10.1067/mph.2002.119599.

474. Jacobs LG, Bonuck K, Burton W, Mulvihill M. Hospital Care at the End of Life: An Institutional Assessment. Journal of Pain and Symptom Management. 2002;24(3):291-8. doi: http://doi.org/10.1016/S0885-3924(02)00494-3.

475. Joy S, Benrubi G. The personal digital assistant in an obstetrics and gynecology residency program. Primary Care Update for OB/GYNS. 2002;9(6):238-42. doi: http://doi.org/10.1016/S1068-607X(02)00125-7.

476. Joy S, Benrubi G. The personal digital assistant in an obstetrics and gynecology residency program. Primary Care Update for Ob/Gyns. 2002;9(6):238-42. doi: 10.1016/S1068-607X(02)00125-7.

477. Kim JH, Kim WO, Min KT, Yang JY, Nam YT. Learning by computer simulation does not lead to better test performance than textbook study in the diagnosis and treatment of dysrhythmias. J Clin Anesth. 2002;14(5):395-400. doi: 10.1016/S0952-8180(02)00384-7.

478. Köhler BU, Hennig C, Orglmeister R. The principles of software QRS detection. IEEE Eng Med Biol Mag. 2002;21(1):42-57. doi: 10.1109/51.993193.

479. Lalande A, Van Kien PK, Salvé N, Salem DB, Legrand L, Walker PM, et al. Automatic determination of aortic compliance with cinemagnetic resonance Imaging: An application of fuzzy logic theory. Invest Radiol. 2002;37(12):685-91. doi: 10.1097/00004424-200212000-00008.

480. Liu CL, Nakashima K, Sako H, Fujisawa H, editors. Handwritten digit recognition using state-of-the-art techniques. 8th International Workshop on Frontiers in Handwriting Recognition, IWFHR 2002; 2002; Ontario, ON.

481. Maglaveras N, Koutkias V, Chouvarda I, Goulis DG, Avramides A, Adamidis D, et al. Home care delivery through the mobile telecommunications platform: the Citizen Health System (CHS) perspective. International Journal of Medical Informatics. 2002;68(1–3):99-111. doi: http://doi.org/10.1016/S1386-5056(02)00069-2.

482. Nakao M, Oyama H, Komori M, Matsuda T, Sakaguchi G, Komeda M, et al. Haptic reproduction and interactive visualization of a beating heart for cardiovascular surgery simulation. International Journal of Medical Informatics. 2002;68(1–3):155-63. doi: http://doi.org/10.1016/S1386-5056(02)00073-4.

483. Nakao M, Oyama H, Komori M, Matsuda T, Sakaguchi G, Komeda M, et al. Haptic reproduction and interactive visualization of a beating heart for cardiovascular surgery simulation. International Journal of Medical Informatics. 2002;68(1-3):155-63. doi: 10.1016/S1386-5056(02)00073-4.

484. Rojo-Álvarez JL, Arenal-Maíz Á, Artés-Rodríguez A. Discriminating between supraventricular and ventricular tachycardias from EGM onset analysis. IEEE Eng Med Biol Mag. 2002;21(1):16-26. doi: 10.1109/51.993190.

485. Rojo-Álvarez JL, Arenal-Maíz Á, Artés-Rodríguez A. Support vector black-box interpretation in ventricular arrhythmia discrimination. IEEE Eng Med Biol Mag. 2002;21(1):27-35. doi: 10.1109/51.993191.

486. Schlindwein M, von Wagner G, Kirst M, Rajewicz M, Karl F, Schochlin J, et al. Mobile patient simulator for resuscitation training with automatic external defibrillators. Biomed Tech (Berl). 2002;47 Suppl 1 Pt 2:559-60. Epub 2002/12/06. PubMed PMID: 12465236.

487. Schlindwein M, von Wagner G, Kirst M, Rajewicz M, Karl F, Schöchlin J, et al. Mobile patient simulator for resuscitation training with automatic external defibrillators. Biomed Tech (Berl). 2002;47 Suppl 1 Pt 2:559-60.

488. Shneyder Y. Personal Digital Assistants (PDA) for the nurse practitioner. Journal of Pediatric Health Care. 2002;16(6):317-20. doi: http://doi.org/10.1067/mph.2002.130153.

489. Vargas F, Lettnin D, Brum D, Prestes D. A new learning approach to design fault tolerant ANNs: Finally a zero HW-SW overhead. Proc Asian Test Symp. 2002;2002-January:218-23. doi: 10.1109/ATS.2002.1181714.

490. Vargas F, Lettnin D, De Castro MCF, Macarthy M. Electrocardiogram pattern recognition by means of MLP network and PCA: A case study on equal amount of input signal types. Proc - Brazilian Symp Neural Netw SBRN. 2002;2002-January:200-5. doi: 10.1109/SBRN.2002.1181474.

491. Veasey S, Rosen R, Barzansky B, Rosen I, Owens J. Sleep loss and fatigue in residency training: A reappraisal. J AM MED ASSOC. 2002;288(9):1116-24.

492. Walsh GM. Emerging safety issues regarding long-term usage of H 1-receptor antagonists. Expert Opin Drug Saf. 2002;1(3):225-35. doi: 10.1517/14740338.1.3.225.

493. Zhang XS, Huang JW, Roy RJ. Modeling for neuromonitoring depth of anesthesia. CRIT REV BIOMED ENG. 2002;30(1-3):131-73. doi: 10.1615/CritRevBiomedEng.v30.i123.70.

494. Zimmermann PG. Cutting-edge answers on management, policy, and program issues in emergency care. Journal of Emergency Nursing. 2002;28(6):562-71. doi: http://doi.org/10.1067/men.2002.127206.

495. Research Abstracts. Journal of Emergency Nursing. 2003;29(2):98-105. doi: http://doi.org/10.1067/men.2003.38-51.

496. Abtracts Diagnosis, assessment, and reviews. The Journal of Pain. 2003;4(2, Supplement):1-104. doi: http://doi.org/.

497. Alliche A, Mokrani K, editors. Higher order statistics and ECG arrhythmia classification. 3rd IEEE International Symposium on Signal Processing and Information Technology, ISSPIT 2003; 2003: Institute of Electrical and Electronics Engineers Inc.

498. Armstrong WF. Heart disease and myocardial infarction in 1957: a patient's perspective. ACC Current Journal Review. 2003;12(6):17-8. doi: http://doi.org/10.1016/j.accreview.2003.09.065.

499. Ayache N. Epidaure: A Research Project in Medical Image Analysis, Simulation, and Robotics at INRIA. IEEE Trans Med Imaging. 2003;22(10):1185-201. doi: 10.1109/TMI.2003.812863.

500. Castellanos A, Myerburg RJ. The Chicago school of arrhythmology: Revisited. Card Electrophysiol Rev. 2003;7(1):96-8. doi: 10.1023/A:1023615828855.

501. Craven M, Newman S, Fletcher M, Silvera B, Coore D, Forbes N, et al., editors. Cardiac training simulator using pump with electronic pressure sensor to trigger ventricular fibrillation. Proceedings IEEE SoutheastCon 2003 "Bridging the Digital Divide"; 2003; Ocho Rios, St. Ann.

502. De Carvalho MRP, Tebexreni AS, Barros Neto TL, Sato EI. Oxygen consumption and aerobic conditioning in patients with systemic lupus erythematosus. Rev Bras Reumatol. 2003;43(1):32-7.

503. Decker K, Bauer M. Ergonomics in the operating room - From the anesthesiologist's point of view. Minimally Invasive Ther Allied Technol. 2003;12(6):268-77. doi: 10.1080/13645700310018795.

504. Delaluz V, Kandemir M, Sivasubramaniam A, Irwin MJ, Vijaykrishnan N, editors. Reducing dTLB energy through dynamic resizing. Proceedings: 21st International Conference on Computer Design ICCD 2003; 2003; San Jose, CA.

505. Grace SL, Abbey SE, Bisaillon S, Shnek ZM, Irvine J, Stewart DE. Presentation, delay, and contraindication to thrombolytic treatment in females and males with myocardial infarction. Women's Health Issues. 2003;13(6):214-21. doi: http://doi.org/10.1016/j.whi.2003.09.002.

506. Khriesat I, Najada AH. Acute rheumatic fever without early carditis: An atypical clinical presentation. Eur J Pediatr. 2003;162(12):868-71. doi: 10.1007/s00431-003-1320-x.

507. Kitajima I. [The ideal curriculum for undergraduate clinical practice training in clinical laboratory medical education]. Rinsho Byori. 2003;51(10):983-94. Epub 2003/12/05. PubMed PMID: 14653198.

508. Kurnaz MN, Dokur Z, Ölmez T, editors. Segmentation of Ultrasound Images by Using Wavelet Transform. A New Beginning for Human Health: Proceedings of the 25th Annual International Conference of the IEEE Engineering in Medicine and Biology Society; 2003; Cancun.

509. Ladouceur R, Sevigny S, Blaszczynski A, O'Connor K, Lavoie ME. Video lottery: winning expectancies and arousal. Addiction. 2003;98(6):733-8. Epub 2003/06/05. PubMed PMID: 12780361.

510. Ladouceur R, Sévigny S, Blaszczynski A, O'Connor K, Lavoie ME. Video lottery: Winning expectancies and arousal. Addiction. 2003;98(6):733-8. doi: 10.1046/j.1360-0443.2003.00412.x.

511. Lee YH, Liu BS. Inflight workload assessment: comparison of subjective and physiological measurements. Aviat Space Environ Med. 2003;74(10):1078-84. Epub 2003/10/15. PubMed PMID: 14556571.

512. Mackenzie CF, Hu FM, Xiao Y, Seagull FJ. Video acquisition and audio system network (VAASNET®) for analysis of workplace safety performance. BIOMED INSTRUM TECHNOL. 2003;37(4):285-91.

513. Menzies T. 21st-century al: Proud, not smug. IEEE Intell Syst. 2003;18(3):18-24. doi: 10.1109/MIS.2003.1200723.

514. Morin RL, Gerber TC, McCollough CH. Physics and dosimetry in computed tomography. Cardiol Clin. 2003;21(4):515-20. doi: 10.1016/S0733-8651(03)00108-5.

515. Nielsen R. Electrocardiograms. BIOMED INSTRUM TECHNOL. 2003;37(4):281-4.

516. Riva G. Applications of Virtual Environments in Medicine. METHODS INF MED. 2003;42(5):524-34.

517. Sandlin D. Perianesthesia nursing care considerations for laparoscopic adjustable gastric banding minimally invasive surgical patients. Journal of PeriAnesthesia Nursing. 2003;18(4):272-6. doi: http://doi.org/10.1016/S1089-9472(03)00134-5.

518. Shah AN, Frush K, Luo X, Wears RL. Effect of an intervention standardization system on pediatric dosing and equipment size determination: A crossover trial involving simulated resuscitation events. Arch Pediatr Adolesc Med. 2003;157(3):229-36.

519. Sheridan S, Pignone M, Mulrow C. Framingham-based Tools to Calculate the Global Risk of Coronary Heart Disease: A Systematic Review of Tools for Clinicians. J Gen Intern Med. 2003;18(12):1039-52. doi: 10.1111/j.1525-1497.2003.30107.x.

520. Shub C. Echocardiography or auscultation? How to evaluate systolic murmurs. Can Fam Phys. 2003;49(FEB.):163-7.

521. Stasis AC, Loukis EN, Pavlopoulos SA, Koutsouris D, editors. Using decision tree algorithms as a basis for a heart sound diagnosis decision support system. 4th International IEEE EMBS Special Topic Conference on Information Technology Applications in Biomedicine 2003, ITAB 2003; 2003: Institute of Electrical and Electronics Engineers Inc.

522. Tümer MB, Belfore Ii LA, Ropella KM. A Syntactic Methodology for Automatic Diagnosis by Analysis of Continuous Time Measurements Using Hierarchical Signal Representations. IEEE Trans Syst Man Cybern Part B Cybern. 2003;33(6):951-65. doi: 10.1109/TSMCB.2002.804365.

523. Vallverdú M, Palacios M, Caminal P, editors. Modeling the dynamics of the heart rate variability by Hidden Markov models. Computers in Cardiology 2003; 2003; Thessaloniki Chalkidiki.

524. Van Klei WA, Grobbee DE, Rutten CLG, Hennis PJ, Knape JTA, Kalkman CJ, et al. Role of history and physical examination in preoperative evaluation. Eur J Anaesthesiol. 2003;20(8):612-8. doi: 10.1017/S026502150300098X.

525. Walsh R. Online CEUs. Journal of Emergency Nursing. 2003;29(6):568-9. doi: http://doi.org/10.1016/j.jen.2003.09.005.

526. Zimmerman MW, Povinelli RJ, Johnson MT, Ropella KM, editors. A reconstructed phase space approach for distinguishing ischemic from non-ischemic ST changes using Holter ECG data. Computers in Cardiology 2003; 2003; Thessaloniki Chalkidiki.

527. Subject index. Journal of PeriAnesthesia Nursing. 2004;19(6):444-57. doi: http://doi.org/10.1016/S1089-9472(04)00371-5.

528. Abstracts of the 26th congress of ESPEN, the European society for clinical nutrition ; metabolism. Clinical Nutrition. 2004;23(4):757-944. doi: http://doi.org/10.1016/j.clnu.2004.06.003.

529. Abo-Zahhad M, Al-Smadi A, Ahmed SM. High-quality low-complexity wavelet-based compression algorithm for audio signals. Electr Eng. 2004;86(4):219-27.

530. Alexander M. Mosby's ECGs online: St. Louis, MO: MosbyJems, 2003, $36.95. Prehospital Emergency Care. 2004;8(1):73-4. doi: http://doi.org/10.1197/S1090-3127(03)00284-3.

531. Alinier G, Hunt WB, Gordon R. Determining the value of simulation in nurse education: study design and initial results. Nurse Education in Practice. 2004;4(3):200-7. doi: http://doi.org/10.1016/S1471-5953(03)00066-0.

532. Andre AD, Jorgenson DB, Froman JA, Snyder DE, Poole JE. Automated external defibrillator use by untrained bystanders:: Can the public-use model work? Prehospital Emergency Care. 2004;8(3):284-91. doi: http://doi.org/10.1016/j.prehos.2004.02.004.

533. Baker CS. Learning on the Web. Case 3: acute chest pain. Heart. 2004;90(1):112. Epub 2007/12/12. PubMed PMID: 18069146; PubMed Central PMCID: PMCPMC1768022.

534. Botsis T, Halkiotis SC, Kourlaba G. Computer simulation of the human respiratory system for educational purposes. CIN Comput Informatics Nurs. 2004;22(3):162-70.

535. Cohen MS. Fetal and childhood onset of adult cardiovascular diseases. Pediatr Clin North Am. 2004;51(6 SPEC. ISS.):1697-719. doi: 10.1016/j.pcl.2004.08.001.

536. de Chillou C, Magnin-Poull I, Andronache M, Abdelaal A, State S, Blangy H, et al. [The electrocardiogram in ventricular tachycardias]. Arch Mal Coeur Vaiss. 2004;97 Spec No 4(4):13-24. Epub 2005/02/18. PubMed PMID: 15714886.

537. de Waard D, Steyvers FJJM, Brookhuis KA. How much visual road information is needed to drive safely and comfortably? Safety Science. 2004;42(7):639-55. doi: http://doi.org/10.1016/j.ssci.2003.09.002.

538. Deschamps A, Kaufman I, Backman SB, Plourde G. Autonomic nervous system response to epidural analgesia in laboring patients by wavelet transform of heart rate and blood pressure variability. ANESTHESIOLOGY. 2004;101(1):21-7. doi: 10.1097/00000542-200407000-00006.

539. Fairbanks RJ, Caplan S. Poor Interface Design and Lack of Usability Testing Facilitate Medical Error. The Joint Commission Journal on Quality and Safety. 2004;30(10):579-84. doi: http://doi.org/10.1016/S1549-3741(04)30068-7.

540. Finder JD, Birnkrant D, Carl J, Farber HJ, Gozal D, Iannaccone ST, et al. Respiratory care of the patient with duchenne muscular dystrophy: ATS consensus statement. Am J Respir Crit Care Med. 2004;170(4):456-65. doi: 10.1164/rccm.200307-885ST.

541. Friedman WA, Laws Jr ER, Burchiel KJ, Berger MS, Grossman RG, Hodge Jr CJ. Resident Duty Hours in American Neurosurgery. Neurosurgery. 2004;54(4):925-33.

542. Geggel RL. Conditions leading to pediatric cardiology consultation in a tertiary academic hospital. Pediatrics. 2004;114(4):e409-e17. doi: 10.1542/peds.2003-0898-L.

543. Greenleaf JE, Rehrer NJ, Mohler SR, Quach DT, Evans DG. Airline chair-rest deconditioning: Induction of immobilisation thromboemboli? SPORTS MED. 2004;34(11):705-25. doi: 10.2165/00007256-200434110-00002.

544. Hall L, Gordon A, Newall L, James R. A development environment for intelligent applications on mobile devices. Expert Sys Appl. 2004;27(3):481-92. doi: 10.1016/j.eswa.2004.05.010.

545. Haraldsson H, Edenbrandt L, Ohlsson M. Detecting acute myocardial infarction in the 12-lead ECG using Hermite expansions and neural networks. Artif Intell Med. 2004;32(2):127-36. doi: 10.1016/j.artmed.2004.01.003.

546. Hughes NP, Tarassenko L, Roberts SJ, editors. Markov models for automated ECG interval analysis. 17th Annual Conference on Neural Information Processing Systems, NIPS 2003; 2004; Vancouver, BC: Neural information processing systems foundation.

547. James B, Lindstrom J. Construct validity of the professional behavior evaluation instrument from the National Standard Paramedic Curriculum. Prehospital Emergency Care. 2004;8(4):434-5. doi: http://doi.org/10.1016/j.prehos.2004.06.002.

548. Kanz KG, Kay MV, Biberthaler P, Russ W, Lackner CK, Mutschler W. Effect of digital cellular phones on tachyarrhythmia analysis of automated external defibrillators. Eur J Emerg Med. 2004;11(2):75-80.

549. Kanz KG, Kay MV, Biberthaler P, Russ W, Lackner CK, Mutschler W. Effect of digital cellular phones on tachyarrhythmia analysis of automated external defibrillators. Eur J Emerg Med. 2004;11(2):75-80. Epub 2004/03/19. PubMed PMID: 15028895.

550. Kanz KG, Kay MV, Biberthaler P, Russ W, Wessel S, Lackner CK, et al. Susceptibility of automated external defibrillators to train overhead lines and metro third rails. Resuscitation. 2004;62(2):189-98. doi: 10.1016/j.resuscitation.2004.02.018.

551. Kim KH, Bang SW, Kim SR. Emotion recognition system using short-term monitoring of physiological signals. Med Biol Eng Comput. 2004;42(3):419-27. doi: 10.1007/BF02344719.

552. Kim S, André E, editors. A generate and sense approach to automated music composition. IUI 04: 2004 International Conference on Intelligent User Interfaces; 2004; Madeira.

553. Koutedakis Y, Jamurtas A. The dancer as a performing athlete: Physiological considerations. SPORTS MED. 2004;34(10):651-61. doi: 10.2165/00007256-200434100-00003.

554. Kumari M, Head J, Marmot M. Prospective study of social and other risk factors for incidence of type 2 diabetes in the Whitehall II study. Arch Intern Med. 2004;164(17):1873-80. doi: 10.1001/archinte.164.17.1873.

555. Leung GM, Yeung RYT, Lai TYY, Johnston JM, Tin KYK, Wong IOL, et al. Physicians’ perceptions towards the impact of and willingness to pay for clinical computerization in Hong Kong. International Journal of Medical Informatics. 2004;73(5):403-14. doi: http://doi.org/10.1016/j.ijmedinf.2004.03.003.

556. Marenzi B. Body piercing: a patient safety issue. Journal of PeriAnesthesia Nursing. 2004;19(1):4-10. doi: http://doi.org/10.1016/j.jopan.2003.11.002.

557. Miaou SG, Chen ST. Automatic quality control for wavelet-based compression of volumetric medical images using distortion-constrained adaptive vector quantization. IEEE Trans Med Imaging. 2004;23(11):1417-29. doi: 10.1109/TMI.2004.835312.

558. Montas HJ, Tipton AJ, editors. Dimensionality effects in cellular automaton modeling of cardiac dynamics. ASAE Annual International Meeting 2004; 2004; Ottawa, ON.

559. Morton JM, Baker CC, Farrell TM, Yohe ME, Kimple RJ, Herman DC, et al. What do surgery residents do on their call nights? Am J Surg. 2004;188(3):225-9. doi: 10.1016/j.amjsurg.2004.06.011.

560. Novák D, Lhotská L, Cuesta-Frau D, Micó P, Al-Ani T, Hamam Y, et al., editors. Morphology analysis of physiological signals using Hidden Markov Models. Proceedings of the 17th International Conference on Pattern Recognition, ICPR 2004; 2004; Cambridge.

561. Ohlsson M. WeAidU - A decision support system for myocardial perfusion images using artificial neural networks. Artif Intell Med. 2004;30(1):49-60. doi: 10.1016/S0933-3657(03)00050-2.

562. Oinuma M, Hirayanagi K, Yajima K, Igarashi M, Arakawa Y. Changes in cardio-respiratory function, heart rate variability, and electrogastrogram preceding motion sickness-like symptoms induced by virtual reality stimulus. Jpn J Aerosp Environ Med. 2004;41(3):99-109.

563. Osowski S, Hoai LT, Markiewicz T. Support Vector Machine-Based Expert System for Reliable Heartbeat Recognition. IEEE TRANS BIOMED ENG. 2004;51(4):582-9. doi: 10.1109/TBME.2004.824138.

564. Page D, Breiter M, Kokx G, Manson J. Online Summative Paramedic Exam (OSPE)–Phase 1: feasibility of using web-based testing tools to develop and administer a final summative exam to paramedic students. Prehospital Emergency Care. 2004;8(4):436-7. doi: http://doi.org/10.1016/j.prehos.2004.06.008.

565. Parshuram CS, Dhanani S, Kirsh JA, Cox PN. Fellowship training, workload, fatigue and physical stress: A prospective observational study. CMAJ. 2004;170(6):965-70. doi: 10.1503/cmaj.1030442.

566. Rani P, Sarkar N, Smith CA, Kirby LD. Anxiety detecting robotic system - Towards implicit human-robot collaboration. Robotica. 2004;22(1):85-95. doi: 10.1017/S0263574703005319.

567. Rix H, Meste O, Muhammad W. Averaging Signals with Random Time Shift and Time Scale Fluctuations. METHODS INF MED. 2004;43(1):13-6.

568. Rosset A, Spadola L, Ratib O. OsiriX: An open-source software for navigating in multidimensional DICOM images. J Digit Imaging. 2004;17(3):205-16. doi: 10.1007/s10278-004-1014-6.

569. Schertler T, Wildermuth S, Willmann JK, Crook DW, Marincek B, Boehm T. Retrospectively ECG-gated multi-detector row CT of the chest: Does ECG-gating improve three-dimensional visualization of the bronchial tree? RoFo Fortschr Geb Rontgenstr Bildgebenden Verfahren. 2004;176(4):513-21. doi: 10.1055/s-2004-812777.

570. Smith AM, Finnie SB, Stuart MJ, Meis J, Beaver KM, Laskowski ER, et al. Psychophysiologic factors and performance in ice hockey goalies during competition. ASTM Spec Tech Publ. 2004;(1446):244-64.

571. Sussman MS, Robert N, Wright GA. Adaptive averaging for improved SNR in real-time coronary artery MRI. IEEE Trans Med Imaging. 2004;23(8):1034-45. doi: 10.1109/TMI.2004.828677.

572. Switt JT. In this issue. Journal of the American Dietetic Association. 2004;104(10):1614-23. doi: http://doi.org/10.1016/j.jada.2004.08.024.

573. Tsipouras MG, Fotiadis DI. Automatic arrhythmia detection based on time and time-frequency analysis of heart rate variability. COMPUT METHODS PROGRAMS BIOMED. 2004;74(2):95-108. doi: 10.1016/S0169-2607(03)00079-8.

574. Vainoras A, Marozas V, Korsakas S, Gargasas L, Siupsinskas L, Miskinis V. Cardiological telemonitoring in rehabilitation and sports medicine. Stud Health Technol Informatics. 2004;105:121-30.

575. Vallverdú M, Palacios M, Hoyer D, Clarià F, Baranowski R, Caminal P, editors. Evaluation of different rhythms by hidden Markov models in heart rate variability of hypertrophic cardiomyopathy patients. Conference Proceedings - 26th Annual International Conference of the IEEE Engineering in Medicine and Biology Society, EMBC 2004; 2004; San Francisco, CA.

576. Wahle A, Olszewski ME, Sonka M. Interactive virtual endoscopy in coronary arteries based on multimodality fusion. IEEE Trans Med Imaging. 2004;23(11):1391-403. doi: 10.1109/TMI.2004.837109.

577. Wang HE, Davis DP, Wayne MA, Delbridge T. Prehospital Rapid-sequence Intubation—What Does the Evidence Show?: Proceedings from the 2004 national association of EMS physicians annual meeting. Prehospital Emergency Care. 2004;8(4):366-77. doi: http://doi.org/10.1016/j.prehos.2004.06.011.

578. Woywodt A, Herrmann A, Kielstein JT, Haller H, Haubitz M, Purnhagen H. A novel multimedia tool to improve bedside teaching of cardiac auscultation. Postgrad Med J. 2004;80(944):355-7. doi: 10.1136/pgmj.2003.014944.

579. Zimmermann PG. Cutting-edge Discussions of Management, Policy, and Program Issues in Emergency Care. Journal of Emergency Nursing. 2004;30(6):559-64. doi: http://doi.org/10.1016/j.jen.2004.09.004.

580. Advance Program for 2005 Emergency Nurses. Journal of Emergency Nursing. 2005;31(3):A27-A54. doi: http://doi.org/10.1016/j.jen.2005.05.002.

581. Abstracts of the 5th International Conference for Emergency Nurses, 13-15 October 2005, Coogee Beach, Sydney, Australia. Australasian Emergency Nursing Journal. 2005;8(3):85-130. doi: http://doi.org/10.1016/j.aenj.2005.08.004.

582. APhA2005 Abstracts of Contributed Papers. Journal of the American Pharmacists Association. 2005;45(2):219-94. doi: http://doi.org/10.1331/1544345053623500.

583. Index to Subjects. Journal of the American Society of Echocardiography. 2005;18(12):1486-513. doi: http://doi.org/10.1016/S0894-7317(05)01033-3.

584. Abacherli R, Pasquier C, Odille F, Kraemer M, Schmid JJ, Felblinger J. Suppression of MR gradient artefacts on electrophysiological signals based on an adaptive real-time filter with LMS coefficient updates. Magma. 2005;18(1):41-50. Epub 2005/02/09. doi: 10.1007/s10334-004-0093-1. PubMed PMID: 15700133.

585. Anthony D. Diagnosis and screening of coronary artery disease. Prim Care Clin Off Pract. 2005;32(4):931-46. doi: 10.1016/j.pop.2005.09.014.

586. Bagnall A, Janacek G. Clustering time series with clipped data. Mach Learn. 2005;58(2-3):151-78. doi: 10.1007/s10994-005-5825-6.

587. Bearnson CS, Wiker KM. Human patient simulators: A new face in baccalaureate nursing education at Brigham Young University. J Nurs Educ. 2005;44(9):421-5.

588. Blackburn GL, Mun EC. Therapy insight: Weight-loss surgery and major cardiovascular risk factors. Nat Clin Pract Cardiovasc Med. 2005;2(11):585-91. doi: 10.1038/ncpcardio0349

10.1007/s00464-004-8825-x]; Korner, J., Effects of Roux-en-Y gastric bypass surgery on fasting and postprandial concentrations of plasma ghrelin, peptide YY, and insulin (2005) J. Clin. Endocrinol. Metab., 90, pp. 359-365; Fruhbeck, G., Fasting plasma ghrelin concentrations 6 months after gastric bypass are not determined by weight loss or changes in insulinemia (2004) Obes. Surg., 14, pp. 1208-1215; Meier, J.J., Nauck, M.A., Glucagon-like peptide 1 (GLP-1) in biology and pathology (2005) Diabetes Metab. Res. Rev., 21, pp. 91-117; Kelly, J., Best practice recommendations for surgical care in weight loss surgery (2005) Obes. Res., 13, pp. 227-233; Klein, S., American Heart Association Scientific Statement. Clinical implications of obesity with specific focus on cardiovascular disease: A statement for professionals from the American Heart Association Council on Nutrition, Physical Activity, and Metabolism: Endorsed by the American College of Cardiology Foundation (2004) Circulation, 110, pp. 2952-2967; Buchwald, H., Bariatric surgery for morbid obesity: Health implications for patients, health professionals, and third-party payers (2005) J. Am. Coll. Surg., 200, pp. 593-604; Nanni, G., Biliopancreatic diversion: Clinical experience (1997) Obes. Surg., 7, pp. 26-29; Marceau, P., Biliopancreatic diversion (duodenal switch procedure) (1999) Eur. J. Gastroenterol. Hepatol., 11, pp. 99-103; Commonwealth of Massachusetts Betsy Lehman Center for Patient Safety and Medical Error Reduction Expert Panel on Weight Loss Surgery: Executive report (2005) Obes. Res., 13, pp. 205-226. , Lehman Center Weight Loss Surgery Expert Panel; Schauer, P., The learning curve for laparoscopic Roux-en-Y gastric bypass is 100 cases (2003) Surg. Endos., 17, pp. 212-215; Nguyen, N.T., A comparison study of laparoscopic versus open gastric bypass for morbid obesity (2000) J. Am. Coll. Surg., 191, pp. 149-155; Dixon, J.B., O'Brien, P.E., Gastroesophageal reflux in obesity: The effect of lap-band placement (1999) Obes. Surg., 9, pp. 527-531; Dixon, J.B., Improvements in insulin sensitivity and β-cell function (HOMA) with weight loss in the severely obese (2003) Diabet. Med., 20, pp. 127-134; Dixon, J.B., Sustained weight loss in obese subjects has benefits that are independent of attained weight (2004) Obes. Res., 12, pp. 1895-1902; Dixon, J.B., Polysomnography before and after weight loss in obese patients with severe sleep apnea (2005) Int. J. Obes. Relat. Metab. Disord., , [doi:10.1038/sj.ijo.0802960]; Dixon, J.B., Surgery as an effective early intervention for diabesity: Why the reluctance? (2005) Diabetes Care, 28, pp. 472-474; Dixon, A.F., Laparoscopic adjustable gastric banding induces prolonged satiety: A randomized blind crossover study (2005) J. Clin. Endocrinol. Metab., 90, pp. 813-819; Hall, J.E., Mechanisms of obesity-associated cardiovascular and renal disease (2002) Am. J. Med. Sci., 324, pp. 127-137; Hackam, D.G., Anand, S.S., Emerging risk factors for atherosclerotic vascular disease: A critical review of the evidence (2003) JAMA, 290, pp. 932-940; Eyre, H., Preventing cancer, cardiovascular disease, and diabetes: A common agenda for the American Cancer Society, the American Diabetes Association, and the American Heart Association (2004) CA Cancer J. Clin., 54, pp. 190-207; Droyvold, W.B., Change in body mass index and its impact on blood pressure: A prospective population study (2005) Int. J. Obes. Relat. Metab. Disord., 29, pp. 650-655; Tanne, D., Body fat distribution and long-term risk of stroke mortality (2005) Stroke, 36, pp. 1021-1025; Gavrila, A., Serum adiponectin levels are inversely associated with overall and central fat distribution but are not directly regulated by acute fasting or leptin administration in humans: Cross-sectional and interventional studies (2003) J. Clin. Endocrinol. Metab., 88, pp. 4823-4831; Sjöstrom, C.D., Reduction in incidence of diabetes, hypertension and lipid disturbances after intentional weight loss induced by bariatric surgery: The SOS Intervention Study (1999) Obes. Res., 7, pp. 477-484; Blackburn, G.L., Solutions in weight control: Lessons from gastric surgery (2005) Am. J. Clin. Nutr., 82 (SUPPL. 1), pp. 248S-252S; Danias, P.G., Cardiac structure and function in the obese: A cardiovascular magnetic resonance imaging study J. Cardiovasc. Magn. Reson., 5, pp. 431-438; Friberg, P., Increased left ventricular mass in obese adolescents (2004) Eur. Heart J., 25, pp. 987-992; Danias, P.G., Comparison of aortic elasticity determined by cardiovascular magnetic resonance imaging in obese versus lean adults (2003) Am. J. Cardiol., 91, pp. 195-199; Karason, K., Effects of obesity and weight loss on cardiac function and valvular performance (1998) Obes. Res., 6, pp. 422-429; Alpert, M.A., Effect of weight loss on the ECG of normotensive morbidly obese patients (2001) Chest, 119, pp. 507-510; Saltzman, E., Criteria for patient selection and multidisciplinary evaluation and treatment of the weight loss surgery patient (2005) Obes. Res., 13, pp. 234-243; Eilat-Adar, S., Association of intentional changes in body weight with coronary heart disease event rates in overweight subjects who have an additional coronary risk factor (2005) Am. J. Epidemiol., 161, pp. 352-358; Pories, W.J., Surgical treatment of obesity and its effect on diabetes: 10-y follow-up (1992) Am. J. Clin. Nutr., 55 (SUPPL. 2), pp. 582S-585S; Choban, P.S., Bariatric surgery for morbid obesity: Why, who, when, how, where, and then what? (2002) Cleve Clin. J. Med., 69, pp. 897-903; Buchwald, H., Bariatric surgery: A systematic review and meta-analysis (2004) JAMA, 292, pp. 1724-1737; Kopp, H.P., Impact of weight loss on inflammatory proteins and their association with the insulin resistance syndrome in morbidly obese patients (2003) Arterioscler. Thromb. Vasc. Biol., 23, pp. 1042-1047; Pontiroli, A.E., Left ventricular hypertrophy and QT interval in obesity and in hypertension: Effects of weight loss and of normalisation of blood pressure (2004) Int. J. Obes. Relat. Metab. Disord., 28, pp. 1118-1123; New ACP guidelines target obesity management (2005), http://www.acponline.org/journals/news/apr05/obesity.htm#care, ACP Observer (online April) American College of Physicians (accessed 2 August 2005)Chapman, A.E., Laparoscopic adjustable gastric banding in the treatment of obesity: A systematic literature review (2004) Surgery, 135, pp. 326-351; Fernandez Jr., A.Z., Experience with over 3,000 open and laparoscopic bariatric procedures: Multivariate analysis of factors related to leak and resultant mortality (2004) Surg. Endosc., 18, pp. 193-197; Sugerman, H.J., Risks and benefits of gastric bypass in morbidly obese patients with severe venous stasis disease (2001) Ann. Surg., 234, pp. 41-46; Flum, D.R., Dellinger, E.P., Impact of gastric bypass operation on survival: A population-based analysis (2004) J. Am. Coll. Surg., 199, pp. 543-551; Chapman, A.E., Laparoscopic adjustable gastric banding in the treatment of obesity: A systematic literature review (2004) Surgery, 135, pp. 326-351; Fielding, G.A., Ren, C.J., Laparoscopic adjustable gastric band (2005) Surg. Clin. North Am., 85, pp. 129-140; Pratt, J.S., Case records of the Massachusetts General Hospital. Weekly clinicopathological exercises. Case 25-2004. A 49-year-old woman with severe obesity, diabetes, and hypertension (2004) N. Engl. J. Med., 35, pp. 696-705.

589. Carvajal R, Wessel N, Vallverdú M, Caminal P, Voss A. Correlation dimension analysis of heart rate variability in patients with dilated cardiomyopathy. COMPUT METHODS PROGRAMS BIOMED. 2005;78(2):133-40. doi: 10.1016/j.cmpb.2005.01.004.

590. Cowperthwaite L, Holm R, Kostka J, Reno D. Attendees earn contact hours and take home valuable information from Congress education sessions: Saturday, April 2, to Thursday, April 7, 2005. AORN Journal. 2005;81(6):1209-22. doi: http://doi.org/10.1016/S0001-2092(06)60384-0.

591. Eason MP. Simulation devices in cardiothoracic and vascular anesthesia. Semin Cardiothorac Vasc Anesth. 2005;9(4):309-23. doi: 10.1177/108925320500900404.

592. Faisan S, Thoraval L, Armspach JP, Foucher JR, Metz-Lutz MN, Heitz F. Hidden Markov event sequence models: Toward unsupervised functional MRI brain mapping. Acad Radiol. 2005;12(1):25-36. doi: 10.1016/j.acra.2004.09.012.

593. Faisan S, Thoraval L, Armspach JP, Metz-Lutz MN, Heitz F. Unsupervised Learning and Mapping of Active Brain Functional MRI Signals Based on Hidden Semi-Markov Event Sequence Models. IEEE Trans Med Imaging. 2005;24(2):263-76. doi: 10.1109/TMI.2004.841225.

594. Gabbott D, Smith G, Mitchell S, Colquhoun M, Nolan J, Soar J, et al. Cardiopulmonary resuscitation standards for clinical practice and training in the UK. Accident and Emergency Nursing. 2005;13(3):171-9. doi: http://doi.org/10.1016/j.aaen.2005.04.004.

595. Gill D, Gavrieli N, Intrator N, editors. Detection and identification of heart sounds using homomorphic envelogram and self-organizing probabilistic model. Computers in Cardiology, 2005; 2005; Lyon.

596. Hayashi K, Ishihara K, Hashimoto H, Oguri K, editors. Individualized drowsiness detection during driving by pulse wave analysis with neural network. 8th International IEEE Conference on Intelligent Transportation Systems; 2005; Vienna.

597. Jafari MG, Chambers JA. Fetal electrocardiogram extraction by sequential source separation in the wavelet domain. IEEE TRANS BIOMED ENG. 2005;52(3):390-400. doi: 10.1109/TBME.2004.842958.

598. Jeffries PR. A framework for designing, implementing, and evaluating: Simulations used as teaching strategies in nursing. Nurs Educ Persp. 2005;26(2):96-103.

599. Jeharon H, Seagar A, Seagar N, editors. Feature extraction from phonocardiogram for diagnosis based on expert system. 2005 27th Annual International Conference of the Engineering in Medicine and Biology Society, IEEE-EMBS 2005; 2005; Shanghai.

600. Kaafarani HMA, Itani KMF, Petersen LA, Thornby J, Berger DH. Does resident hours reduction have an impact on surgical outcomes? J Surg Res. 2005;126(2):167-71. doi: 10.1016/j.jss.2004.12.024.

601. Kadous MW, Sammut C. Classification of multivariate time series and structured data using constructive induction. Mach Learn. 2005;58(2-3):179-216. doi: 10.1007/s10994-005-5826-5.

602. Larin JT, Eagle KA. The Heart Attack Chronicles of a Marathon Runner: Or, Women Will Take the Ambulance but Men Would Rather Drive Themselves. ACC Current Journal Review. 2005;14(5):12-4. doi: http://doi.org/10.1016/j.accreview.2005.04.069.

603. Leong FT, Hughes LO. Learning on the Web. Case 9: a mother's heartache. Heart. 2005;91(4):552. Epub 2006/12/16. PubMed PMID: 17171828; PubMed Central PMCID: PMCPMC1768797.

604. Levin D, Aladl U, Germano G, Slomka P. Techniques for efficient, real-time, 3D visualization of multi-modality cardiac data using consumer graphics hardware. Comput Med Imaging Graph. 2005;29(6):463-75. doi: 10.1016/j.compmedimag.2005.02.007.

605. Li Q, Lee KM, editors. Effects of color characterization on computational efficiency of feature detection with live-object handling applications. Proceedings of the 2005 IEEE/ASME International Conference on Advanced Intelligent Mechatronics, AIM 2005; 2005; Monterey, CA.

606. Liao W, Ajwa IA, editors. On the introduction of Grid computing to undergraduate education. 2005 International Conference on Grid Computing and Applications, GCA'05; 2005; Las Vegas, NV.

607. Manzke R, Koken P, Hawkes D, Grass M. Helical cardiac cone beam CT reconstruction with large area detectors: A simulation study. Physics in Medicine and Biology. 2005;50(7):1547-68. doi: 10.1088/0031-9155/50/7/016.

608. McBride AB. Nursing and the informatics revolution. Nurs Outlook. 2005;53(4):183-91. doi: 10.1016/j.outlook.2005.02.006.

609. Mueller M, Christ T, Dobrev D, Nitsche I, Stehr S, Ravens U, et al. Teaching antiarrhythmic therapy and ECG in simulator-based interdisciplinary undergraduate medical education. BR J ANAESTH [Internet]. 2005; 95(3):[300-4 pp.]. Available from: http://onlinelibrary.wiley.com/o/cochrane/clcentral/articles/615/CN-00522615/frame.html.

610. Mueller MP, Christ T, Dobrev D, Nitsche I, Stehr SN, Ravens U, et al. Teaching antiarrhythmic therapy and ECG in simulator-based interdisciplinary undergraduate medical education. BR J ANAESTH. 2005;95(3):300-4. doi: 10.1093/bja/aei174.

611. Mueller MP, Christ T, Dobrev D, Nitsche I, Stehr SN, Ravens U, et al. Teaching antiarrhythmic therapy and ECG in simulator-based interdisciplinary undergraduate medical education. Br J Anaesth. 2005;95(3):300-4. Epub 2005/06/21. doi: 10.1093/bja/aei174. PubMed PMID: 15964889.

612. Pavlik G, Kemény D, Kneffel Z, Petrekanits M, Horváth P, Sidó Z. Echocardiographic data in Hungarian top-level water polo players. Med Sci Sports Exerc. 2005;37(2):323-8. doi: 10.1249/01.MSS.0000152805.34215.97.

613. Pickering TG, Hall JE, Appel LJ, Falkner BE, Graves J, Hill MN, et al. Recommendations for blood pressure measurement in humans and experimental animals. Part 1: Blood pressure measurement in humans: A statement for professionals from the subcommittee of professional and public education of the American Heart Association council on high blood pressure research. Hypertension. 2005;45(1):142-61. doi: 10.1161/01.HYP.0000150859.47929.8e.

614. Sauerland S, Angrisani L, Belachew M, Chevallier JM, Favretti F, Finer N, et al. Obesity surgery: Evidence-based guidelines of the European Association for Endoscopic Surgery (E.A.E.S.). Surg Endosc Interv Tech. 2005;19(2):200-21. doi: 10.1007/s00464-004-9194-1.

615. Song MH, Lee J, Cho SP, Lee KJ, Yoo SK. Support vector machine based arrhythmia classification using reduced features. Int J Control Autom Syst. 2005;3(4):571-9.

616. Spurney CF, Sable CA, Berger JT, Martin GR. Use of a hand-carried ultrasound device by critical care physicians for the diagnosis of pericardial effusions, decreased cardiac function, and left ventricular enlargement in pediatric patients. Journal of the American Society of Echocardiography. 2005;18(4):313-9. doi: http://doi.org/10.1016/j.echo.2004.10.016.

617. Subasi A, Erçelebi E. Classification of EEG signals using neural network and logistic regression. COMPUT METHODS PROGRAMS BIOMED. 2005;78(2):87-99. doi: 10.1016/j.cmpb.2004.10.009.

618. Tabesh H, Shahghadami R, editors. Computerized heart sound analysis. 3rd IASTED International Conference on Medical Engineering 2005; 2005; Innsbruck.

619. Takeuchi A, Hirose M, Hamada A, Ikeda N. Simulation system of arrhythmia using ActiveX control. COMPUT METHODS PROGRAMS BIOMED. 2005;79(1):49-57. doi: 10.1016/j.cmpb.2005.03.011.

620. Taşar MA, Bostanci I, Atli Ö, Dallar Y. Effect of short-acting inhaler β2-agonists on serum cardiac troponin in wheezy infant. Allergy Asthma Proc. 2005;26(6):477-82.

621. Tri JL, Severson RP, Firl AR, Hayes DL, Abenstein JP. Cellular telephone interference with medical equipment. Mayo Clin Proc. 2005;80(10):1286-90.

622. von Lubitz DKJE, Levine H. Distributed, multiplatform high fidelity human patient simulation environment: A global-range simulation-based medical learning and training network. Int J Healthc Technol Manage. 2005;6(4-6):500-28. doi: 10.1504/IJHTM.2005.006993.

623. Wong KK, Barker AP, Warren AE. Paediatricians' validation of learning objectives in paediatric cardiology. Paediatr Child Health. 2005;10(2):95-9.

624. Company Listings. AORN Journal. 2006;84(6, Supplement 2):S13-S44. doi: http://doi.org/10.1016/S0001-2092(06)63983-5.

625. Al-Assaf Y. Surface myoelectric signal analysis: Dynamic approaches for change detection and classification. IEEE TRANS BIOMED ENG. 2006;53(11):2248-56. doi: 10.1109/TBME.2006.883628.

626. Alinier G, Gordon R, Harwood C, Hunt WB. 12-Lead ECG training: The way forward. Nurse Education Today. 2006;26(1):87-92. doi: http://doi.org/10.1016/j.nedt.2005.08.004.

627. Anne MacNeil C. A Northern Experience in Nunavut. Canadian Journal of Medical Radiation Technology. 2006;37(3):18-23. doi: http://doi.org/10.1016/S0820-5930(09)60097-9.

628. Baier V, Baumert M, Caminal P, Vallverdú M, Faber R, Voss A. Hidden Markov models based on symbolic dynamics for statistical modeling of cardiovascular control in hypertensive pregnancy disorders. IEEE TRANS BIOMED ENG. 2006;53(1):140-3. doi: 10.1109/TBME.2005.859812.

629. Burkett LN, Todd MA, Adams T. Yoga and distractibility. Journal of Bodywork and Movement Therapies. 2006;10(4):276-86. doi: 10.1016/j.jbmt.2005.08.007.

630. Childs JC, Sepples S. Clinical teaching by simulation: Lessons learned from a complex patient care scenario. Nurs Educ Persp. 2006;27(3):154-8.

631. de Virgilio C, Yaghoubian A, Lewis RJ, Stabile BE, Putnam BA. The 80-Hour Resident Workweek Does Not Adversely Affect Patient Outcomes or Resident Education. Curr Surg. 2006;63(6):435-9. doi: 10.1016/j.cursur.2006.03.006.

632. Detling N, Smith A, Nishimura R, Keller S, Martinez M, Young W, et al. Psychophysiologic responses of invasive cardiologists in an academic catheterization laboratory. Am Heart J. 2006;151(2):522-8. doi: 10.1016/j.ahj.2005.03.044.

633. Fulton J, Popovetsky G, Jacoby J, Heller M, Reed J. The effect of IM droperidol on driving performance. J Med Toxicol [Internet]. 2006; 2(3):[93-6 pp.]. Available from: http://onlinelibrary.wiley.com/o/cochrane/clcentral/articles/687/CN-00621687/frame.html.

634. Fulton J, Popovetsky G, Jacoby JL, Heller MB, Reed J. The effect of IM droperidol on driving performance. J Med Toxicol. 2006;2(3):93-6.

635. Fulton J, Popovetsky G, Jacoby JL, Heller MB, Reed J. The effect of IM droperidol on driving performance. J Med Toxicol. 2006;2(3):93-6. Epub 2007/12/12. PubMed PMID: 18072125; PubMed Central PMCID: PMCPMC3550152.

636. Galarraga M, Serrano L, Martínez I, De Toledo P, editors. Standards for medical device communication: X73 PoC-MDC. 3rd Annual International Conference on Medical and Care Compunetics, ICMCC 2006; 2006; The Hague17095823.

637. Green M, Björk J, Forberg J, Ekelund U, Edenbrandt L, Ohlsson M. Comparison between neural networks and multiple logistic regression to predict acute coronary syndrome in the emergency room. Artif Intell Med. 2006;38(3):305-18. doi: 10.1016/j.artmed.2006.07.006.

638. Greenly MA. Helping Hippocrates: A Cross-Functional Approach to Patient Identification. The Joint Commission Journal on Quality and Patient Safety. 2006;32(8):463-9. doi: http://doi.org/10.1016/S1553-7250(06)32061-2.

639. Hadhoud MMA, Eladawy MI, Farag A, editors. Computer aided diagnosis of cardiac arrhythmias. 2006 International Conference on Computer Engineering and Systems, ICCES'06; 2006; Cairo.

640. Harbison J. Clinical judgement in the interpretation of evidence: A Bayesian approach. J Clin Nurs. 2006;15(12):1489-97. doi: 10.1111/j.1365-2702.2005.01487.x.

641. Henderson G, Ifeachor E, Hudson N, Goh C, Outram N, Wimalaratna S, et al. Development and assessment of methods for detecting dementia using the human electroencephalogram. IEEE TRANS BIOMED ENG. 2006;53(8):1557-68. doi: 10.1109/TBME.2006.878067.

642. Hoznek A, Salomon L, De La Taille A, Yiou R, Vordos D, Larre S, et al. Simulation training in video-assisted urologic surgery. Curr Urol Rep. 2006;7(2):107-13. doi: 10.1007/s11934-006-0068-z.

643. Iellamo F, Pigozzi F, Spataro A, Di Salvo V, Fagnani F, Roselli A, et al. Autonomic and psychological adaptations in Olympic rowers. J SPORTS MED PHYS FITNESS. 2006;46(4):598-604.

644. King P, editor Electrocardiogram instruction, capture and analysis. 113th Annual ASEE Conference and Exposition, 2006; 2006; Chicago, IL.

645. Kishnani PS, Steiner RD, Bali D, Berger K, Byrne BJ, Case L, et al. Pompe disease diagnosis and management guideline. Gen Med. 2006;8(5):267-88. doi: 10.1097/01.gim.0000218152.87434.f3.

646. Kitchiner R. The role of the personal digital assistant (PDA) in chiropractic practice. Clinical Chiropractic. 2006;9(3):119-28. doi: http://doi.org/10.1016/j.clch.2006.04.003.

647. Labinaz M, Swabey T, Watson R, Natarajan M, Fucile W, Lubelsky B, et al. Delivery of primary percutaneous coronary intervention for the management of acute ST segment elevation myocardial infarction: Summary of the Cardiac Care Network of Ontario Consensus Report. Canadian Journal of Cardiology. 2006;22(3):243-50. doi: http://doi.org/10.1016/S0828-282X(06)70904-7.

648. Lai WW, Geva T, Shirali GS, Frommelt PC, Humes RA, Brook MM, et al. Guidelines and Standards for Performance of a Pediatric Echocardiogram: A Report from the Task Force of the Pediatric Council of the American Society of Echocardiography. Journal of the American Society of Echocardiography. 2006;19(12):1413-30. doi: http://doi.org/10.1016/j.echo.2006.09.001.

649. Lessard Y, Siregar P, Julen N, Sinteff JP, Le Beux P, editors. Multimedia and physiology: A new way to ensure the quality of medical education and medical knowledge. 20th International Congress of the European Federation for Medical Informatics, MIE 2006; 2006; Maastricht17108626.

650. Maag M. An interactive self-paced electrocardiography electrode placement tutorial. Comput Inform Nurs. 2006;24(3):131-3. Epub 2006/05/19. PubMed PMID: 16707940.

651. Mayur P. Ictal electroencephalographic characteristics during electroconvulsive therapy: A review of determination and clinical relevance. J ECT. 2006;22(3):213-7. doi: 10.1097/01.yct.0000235922.14623.39.

652. McMenamin JP, Tiglio AD. Not the next tobacco: Defenses to obesity claims. Food Drug Law J. 2006;61(3):445-518+i.

653. Medina R, Garreau M, Toro J, Breton HL, Coatrieux JL, Jugo D. Markov random field modeling for three-dimensional reconstruction of the left ventricle in cardiac angiography. IEEE Trans Med Imaging. 2006;25(8):1087-100. doi: 10.1109/TMI.2006.877444.

654. Milosavljević N, Petrović A, editors. ST segment change detection by means of wavelets. 8th Seminar on Neural Network Applications in Electrical Engineering, Neurel-2006; 2006; Belgrade.

655. Morgan MA, Reuter CWM. Molecularly targeted therapies in myelodysplastic syndromes and acute myeloid leukemias. Ann Hematol. 2006;85(3):139-63. doi: 10.1007/s00277-005-0051-7.

656. Paley J. Evidence and expertise. Nurs Inq. 2006;13(2):82-93. doi: 10.1111/j.1440-1800.2006.00307.x.

657. Pines JM, Hollander JE, Datner EM, Metlay JP. Pay for Performance for Antibiotic Timing in Pneumonia: Caveat Emptor. The Joint Commission Journal on Quality and Patient Safety. 2006;32(9):531-5. doi: http://doi.org/10.1016/S1553-7250(06)32069-7.

658. Porsteinsson AP, Cosman KM. Memantine in the treatment of Alzheimer's disease. Aging Health. 2006;2(6):891-904. doi: 10.2217/1745509X.2.6.891.

659. Postolache O, Postolache G, Girão PS, editors. Non-invasive mobile homeostasis instrument. IEEE International Workshop on Medical Measurement and Applications, MeMeA 2006; 2006; Benevento.

660. Riphaus A, Gstettenbauer T, Frenz MB, Wehrmann T. Quality of psychomotor recovery after propofol sedation for routine endoscopy: A randomized and controlled study. Endoscopy. 2006;38(7):677-83. doi: 10.1055/s-2006-925244.

661. Rochitte CE, Pinto IMF, Fernandes JL, Azevedo Filho CF, Jatene A, Carvalho ACDC, et al. The Brazilian Society of Cardiology (SBC) guidelines for resonance and cardiovascular tomography: Study group in resonance and cardiovascular tomography (GERT). Arq Bras Cardiol. 2006;87(3):e60-e100.

662. Rohoman L, Kirilova A, Lee JWK. MR Patient Care, Safety and Contrast Administration. Canadian Journal of Medical Radiation Technology. 2006;37(4):26-33. doi: http://doi.org/10.1016/S0820-5930(09)60195-X.

663. Takla G, Petre JH, Doyle DJ, Horibe M, Gopakumaran B. The problem of artifacts in patient monitor data during surgery: A clinical and methodological review. ANESTH ANALG. 2006;103(5):1196-204. doi: 10.1213/01.ane.0000247964.47706.5d.

664. Thomas J, Rose C, Charpillet F, editors. A multi-HMM approach to ECG segmentation. 18th IEEE International Conference on Tools with Artificial Intelligence, ICTAI 2006; 2006; Arlington, VA.

665. Vemulakonda VM, Jones EA. Primer: Diagnosis and management of uncomplicated daytime wetting in children. Nat Clin Pract Urol. 2006;3(10):551-9. doi: 10.1038/ncpuro0584.

666. Zanzonico P, Rothenberg LN, Strauss HW. Radiation Exposure of Computed Tomography and Direct Intracoronary Angiography. Risk Has its Reward. J Am Coll Cardiol. 2006;47(9):1846-9. doi: 10.1016/j.jacc.2005.10.075.

667. Zgibor JC, Piatt GA, Ruppert K, Orchard TJ, Roberts MS. Deficiencies of cardiovascular risk prediction models for type 1 diabetes. Diabetes Care. 2006;29(8):1860-5. doi: 10.2337/dc06-0290.

668. Subject Index. Journal of Emergency Nursing. 2007;33(6):e23-e30. doi: http://doi.org/10.1016/S0099-1767(07)00638-1.

669. World Physical Therapy 2007 - Abstracts. Physiotherapy. 2007;93, Supplement 1:S1-S802. doi: http://doi.org/10.1016/S0031-9406(07)60001-7.

670. February 2007 New in Review. Journal of the American Dietetic Association. 2007;107(2):336-45. doi: http://doi.org/10.1016/j.jada.2006.12.021.

671. Author Index. Journal of Emergency Nursing. 2007;33(6):e19-e22. doi: http://doi.org/10.1016/S0099-1767(07)00637-X.

672. 2007 AMTC Scientific Assembly Monday, September 17, 2007. Air Medical Journal. 2007;26(5):230-9. doi: http://doi.org/10.1016/j.amj.2007.06.002.

673. Poster Session III 2007. Journal of the American Society of Echocardiography. 2007;20(5):603-27. doi: http://doi.org/10.1016/j.echo.2007.04.006.

674. Poster Session II 2007. Journal of the American Society of Echocardiography. 2007;20(5):578-603. doi: http://doi.org/10.1016/j.echo.2007.04.005.

675. Al Khatib I, Bertozzi D, Jantsch A, Benini L, editors. Performance analysis and design space exploration for high-end biomedical applications: Challenges and solutions. CODES+ISSS 2007: 5th International Conference on Hardware/Software Codesign and System Synthesis; 2007; Salzburg.

676. Al-ani T, Le Ba QT, Monacelli E, editors. On-line automatic detection of human activity in home using Wavelet and Hidden Markov Models Scilab Toolkits. 16th IEEE International Conference on Control Applications, CCA 2007 Part of IEEE Multi-conference on Systems and Control; 2007.

677. Balasubramanian V, Adalarasu K. EMG-based analysis of change in muscle activity during simulated driving. Journal of Bodywork and Movement Therapies. 2007;11(2):151-8. doi: http://doi.org/10.1016/j.jbmt.2006.12.005.

678. Balasubramanian V, Adalarasu K. EMG-based analysis of change in muscle activity during simulated driving. Journal of Bodywork and Movement Therapies. 2007;11(2):151-8. doi: 10.1016/j.jbmt.2006.12.005.

679. Bhavsar J, Montgomery D, Li J, Kline-Rogers E, Saab F, Motivala A, et al. Impact of Duty Hours Restrictions on Quality of Care and Clinical Outcomes. The American Journal of Medicine. 2007;120(11):968-74. doi: http://doi.org/10.1016/j.amjmed.2007.07.026.

680. Bhavsar J, Montgomery D, Li J, Kline-Rogers E, Saab F, Motivala A, et al. Impact of Duty Hours Restrictions on Quality of Care and Clinical Outcomes. Am J Med. 2007;120(11):968-74. doi: 10.1016/j.amjmed.2007.07.026.

681. Blackstone EH, Rice TW. From Trees to Wood and Back: Perspective on Clinical Data Analysis in Thoracic Surgery. Thorac Surg Clin. 2007;17(3):309-27. doi: 10.1016/j.thorsurg.2007.07.005.

682. Boey RA, Wuyts FL, Van de Heyning PH, De Bodt MS, Heylen L. Characteristics of stuttering-like disfluencies in Dutch-speaking children. Journal of Fluency Disorders. 2007;32(4):310-29. doi: http://doi.org/10.1016/j.jfludis.2007.07.003.

683. Bouder F. A case study of long QT regulation: A regulatory tennis game across the Atlantic. J Risk Res. 2007;10(3):385-412. doi: 10.1080/13669870701270903.

684. Brown PP, Houser F, Kugelmass AD, Anderson AL, Tarkington LG, Simon AW, et al. Cardiovascular Centers of Excellence Program: A System Approach for Improving the Care and Outcomes of Cardiovascular Patients at HCA Hospitals. The Joint Commission Journal on Quality and Patient Safety. 2007;33(11):647-59. doi: http://doi.org/10.1016/S1553-7250(07)33074-2.

685. Califf RM, Mehta RH, Peterson ED. Clinical Quality in Non–ST-Elevation Acute Coronary Syndromes. The American Journal of Medicine. 2007;120(11):930-5. doi: http://doi.org/10.1016/j.amjmed.2006.10.016.

686. Calkins H, Brugada J, Packer DL, Cappato R, Chen SA, Crijns HJG, et al. HRS/EHRA/ECAS Expert Consensus Statement on Catheter and Surgical Ablation of Atrial Fibrillation: Recommendations for Personnel, Policy, Procedures and Follow-Up. A report of the Heart Rhythm Society (HRS) Task Force on Catheter and Surgical Ablation of Atrial Fibrillation. Developed in partnership with the European Heart Rhythm Association (EHRA). Heart Rhythm. 2007;4(6):816-61. doi: 10.1016/j.hrthm.2007.04.005.

687. Calkins H, Brugada J, Packer DL, Cappato R, Chen SA, Crijns HJG, et al. HRS/EHRA/ECAS expert consensus statement on catheter and surgical ablation of atrial fibrillation: Recommendations for personnel, policy, procedures and follow-up. A report of the Heart Rhythm Society (HRS) Task Force on catheter and surgical ablation of atrial fibrillation. Europace. 2007;9(6):335-79. doi: 10.1093/europace/eum120.

688. Casajus JA, Castagna C. Aerobic fitness and field test performance in elite Spanish soccer referees of different ages. Journal of Science and Medicine in Sport. 2007;10(6):382-9. doi: http://doi.org/10.1016/j.jsams.2006.08.004.

689. Chen X, Ho CT, Lim ET, Kyaw TZ, editors. Cellular phone based online ECG processing for ambulatory and continuous detection. Computers in Cardiology 2007, CAR 2007; 2007; Durham, NC.

690. Chudáček V, Petrík M, Georgoulas G, Čepek M, Lhotská L, Stylios C, editors. Comparison of seven approaches for holter ECG clustering and classification. 29th Annual International Conference of IEEE-EMBS, Engineering in Medicine and Biology Society, EMBC'07; 2007; Lyon.

691. Cody DD, Mahesh M. AAPM/RSNA physics tutorial for residents: Technologic advances in multidetector CT with a focus on cardiac imaging. Radiographics. 2007;27(6):1829-37. doi: 10.1148/rg.276075120.

692. Cohen LB, DeLegge MH, Aisenberg J, Brill JV, Inadomi JM, Kochman ML, et al. AGA Institute Review of Endoscopic Sedation. Gastroenterology. 2007;133(2):675-701. doi: 10.1053/j.gastro.2007.06.002.

693. Cumin D, Merry AF. Simulators for use in anaesthesia. Anaesthesia. 2007;62(2):151-62. doi: 10.1111/j.1365-2044.2006.04902.x.

694. Dossey L. The Undead: Botched Burials, Safety Coffins, and the Fear of the Grave. EXPLORE: The Journal of Science and Healing. 2007;3(4):347-54. doi: http://doi.org/10.1016/j.explore.2007.05.001.

695. Febvre M, Trosini-Desert V, Atassi K, Hermant C, Colchen A, Raspaud C, et al. Les bonnes pratiques de la bronchoscopie souple diagnostique, en 2007. Revue des Maladies Respiratoires. 2007;24(10):1363-92. doi: http://doi.org/10.1016/S0761-8425(07)78513-3.

696. Green M, Ohlsson M, Lundager Forberg J, Björk J, Edenbrandt L, Ekelund U. Best leads in the standard electrocardiogram for the emergency detection of acute coronary syndrome. J Electrocardiol. 2007;40(3):251-6. doi: 10.1016/j.jelectrocard.2006.12.011.

697. Hallez H, Vanrumste B, Grech R, Muscat J, De Clercq W, Vergult A, et al. Review on solving the forward problem in EEG source analysis. J NeuroEng Rehabil. 2007;4. doi: 10.1186/1743-0003-4-46.

698. Høilund-Carlsen PF, Johansen A, Vach W, Christensen HW, Møldrup M, Haghfelt T. High probability of disease in angina pectoris patients: Is clinical estimation reliable? Canadian Journal of Cardiology. 2007;23(8):641-7. doi: http://doi.org/10.1016/S0828-282X(07)70226-X.

699. Hongzong S, Tao W, Xiaojun Y, Huanxiang L, Zhide H, Mancang L, et al. Support vector machines classification for discriminating coronary heart disease patients from non-coronary heart disease. West Indian Med J. 2007;56(5):451-7.

700. Honos G, Amyot R, Choy J, Leong-Poi H, Schnell G, Yu E. Contrast echocardiography in Canada: Canadian Cardiovascular Society/Canadian Society of Echocardiography position paper. Canadian Journal of Cardiology. 2007;23(5):351-6. doi: http://doi.org/10.1016/S0828-282X(07)70767-5.

701. Hoyle RJ, Walker KJ, Thomson G, Bailey M. Accuracy of electrocardiogram interpretation improves with emergency medicine training. EMA Emerg Med Australas. 2007;19(2):143-50. doi: 10.1111/j.1742-6723.2007.00946.x.

702. Huaming L, Jindong T, editors. Body sensor network based ECG segmentation and analysis. 29th Annual International Conference of IEEE-EMBS, Engineering in Medicine and Biology Society, EMBC'07; 2007; Lyon.

703. Jamšek J, Stefanovska A, editors. The cardiorespiratory couplings observed in the LDF signal using wavelet bispectrum. 29th Annual International Conference of IEEE-EMBS, Engineering in Medicine and Biology Society, EMBC'07; 2007; Lyon.

704. Juvé Udina ME, Muñoz SF, Calvo CM, Prat DM, Barrabés GF, Serra RM, et al. ¿Cómo definen los profesionales de enfermería hospitalarios sus competencias asistenciales? Nursing (Ed española). 2007;25(7):50-61. doi: http://doi.org/10.1016/S0212-5382(07)70957-3.

705. Kelley FJ, Kopac CA, Rosselli J. Advanced Health Assessment in Nurse Practitioner Programs: Follow-Up Study. Journal of Professional Nursing. 2007;23(3):137-43. doi: http://doi.org/10.1016/j.profnurs.2006.12.005.

706. Khan N, Rajput AQK, Chowdhry BS, Arain AA, editors. Design of quality ensuing, real-time WLAN tele-monitoring healthcare system. 8th World Wireless Congress, WWC 2007; 2007; San Francisco, CA.

707. Khawaja A, Dössel O. Predicting the QRS complex and detecting small changes using principal component analysis. Biomed Tech (Berl). 2007;52(1):11-7. doi: 10.1515/BMT.2007.004.

708. Kiryu T, Iijima A, Bando T. Relationships between sensory stimuli and autonomic nervous regulation during real and virtual exercises. J NeuroEng Rehabil. 2007;4. doi: 10.1186/1743-0003-4-38.

709. Kostic MN, Fakhar S, Foxall T, Drakulic BS, Krucoff MW. Evaluation of novel ECG signal processing on quantification of transient ischemia and baseline wander suppression. Conf Proc IEEE Eng Med Biol Soc. 2007:2199-202.

710. Kostic MN, Fakhar S, Foxall T, Drakulic BS, Krucoff MW. Evaluation of novel ECG signal processing on quantification of transient ischemia and baseline wander suppression. Conf Proc IEEE Eng Med Biol Soc. 2007;2007:2199-202. Epub 2007/11/16. doi: 10.1109/iembs.2007.4352760. PubMed PMID: 18002426.

711. Kramer-Johansen J, Edelson DP, Abella BS, Becker LB, Wik L, Steen PA. Pauses in chest compression and inappropriate shocks: A comparison of manual and semi-automatic defibrillation attempts. Resuscitation. 2007;73(2):212-20. doi: 10.1016/j.resuscitation.2006.09.006.

712. Kremser AK, Lyneham J. Can Australian Nurses Safely Assess for Thrombolysis on EKG Criteria? Journal of Emergency Nursing. 2007;33(2):102-9. doi: http://doi.org/10.1016/j.jen.2006.10.015.

713. Lahtinen TM, Koskelo JP, Laitinen T, Leino TK. Heart rate and performance during combat missions in a flight simulator. Aviat Space Environ Med. 2007;78(4):387-91. Epub 2007/05/09. PubMed PMID: 17484341.

714. Lee KM, Li Q, Daley W. Effects of classification methods on color-based feature detection with food processing applications. IEEE Trans Autom Sci Eng. 2007;4(1):40-51. doi: 10.1109/TASE.2006.874972.

715. Lima CS, Cardoso MJ, editors. Cardiac arrhythmia detection by parameters sharing and MMIE training of hidden markov models. 29th Annual International Conference of IEEE-EMBS, Engineering in Medicine and Biology Society, EMBC'07; 2007; Lyon.

716. Lloyd G, Kendall J, Meek S, Younge P. High-level simulators in emergency department education: Thoughts from the trainers' perspective. Emerg Med J. 2007;24(4):288-91. doi: 10.1136/emj.2006.033779.

717. Löwbeer C, Seeberger A, Gustafsson SA, Bouvier F, Hulting J. Serum cardiac troponin T, troponin I, plasma BNP and left ventricular mass index in professional football players. Journal of Science and Medicine in Sport. 2007;10(5):291-6. doi: http://doi.org/10.1016/j.jsams.2006.10.002.

718. Mahesh M, Cody DD. AAPM/RSNA physics tutorial for residents: Physics of cardiac imaging with multiple-row detector CT. Radiographics. 2007;27(5):1495-509. doi: 10.1148/rg.275075045.

719. Martinez-Möller A, Zikic D, Botnar RM, Bundschuh RA, Howe W, Ziegler SI, et al. Dual cardiac-respiratory gated PET: Implementation and results from a feasibility study. Eur J Nucl Med Mol Imaging. 2007;34(9):1447-54. doi: 10.1007/s00259-007-0374-9.

720. Mehta SS, Lingayat NS. Development of entropy based algorithm for cardiac beat detection in 12-lead electrocardiogram. Signal Process. 2007;87(12):3190-201. doi: 10.1016/j.sigpro.2007.06.009.

721. Mehta SS, Lingayat NS, editors. Biomedical signal processing using SVM. IET-UK International Conference on Information and Communication Technology in Electrical Sciences, ICTES 2007; 2007; Tamil Nadu.

722. Montgomery VL. Effect of fatigue, workload, and environment on patient safety in the pediatric intensive care unit. Pediatr Crit Care Med. 2007;8(2 SUPPL.):S11-S6. doi: 10.1097/01.PCC.0000257735.49562.8F.

723. Morad Y, Azaria B, Avni I, Barkana Y, Zadok D, Kohen-Raz R, et al. Posturography as an indicator of fatigue due to sleep deprivation. Aviat Space Environ Med. 2007;78(9):859-63.

724. Morris RW, Pybus DA. "Orpheus" cardiopulmonary bypass simulation system. J Extra-Corpor Technol. 2007;39(4):228-33.

725. Morris RW, Pybus DA. "Orpheus" cardiopulmonary bypass simulation system. J Extra Corpor Technol. 2007;39(4):228-33. Epub 2008/02/26. PubMed PMID: 18293807; PubMed Central PMCID: PMCPMC4680687.

726. Moylan KC, Binder EF. Falls in Older Adults: Risk Assessment, Management and Prevention. The American Journal of Medicine. 2007;120(6):493.e1-.e6. doi: http://doi.org/10.1016/j.amjmed.2006.07.022.

727. Muehlsteff J, Thijs J, Pinter R, Morren G, Muesch G, editors. A handheld device for simultaneous detection of electrical and mechanical cardio-vascular activities with synchronized ECG and CW-Doppler Radar. 29th Annual International Conference of IEEE-EMBS, Engineering in Medicine and Biology Society, EMBC'07; 2007; Lyon.

728. Muhs BE, Verhagen HJM, Huddle MG, Pai VM, Hecht EM, Dardik A. Theory, technique, and practice of magnetic resonance angiography. Vascular. 2007;15(6):376-83. doi: 10.2310/6670.2007.00052.

729. Ogden PE, Cobbs LS, Howell MR, Sibbitt SJB, DiPette DJ. Clinical Simulation: Importance to the Internal Medicine Educational Mission. The American Journal of Medicine. 2007;120(9):820-4. doi: http://doi.org/10.1016/j.amjmed.2007.06.017.

730. Parrish FJ. Volume CT: State-of-the-art reporting. Am J Roentgenol. 2007;189(3):528-34. doi: 10.2214/AJR.07.2426.

731. Pfeifer B, Seger M, Hintermüller C, Fischer G, Mühlthaler H, Modre-Osprian R, et al. AAM-based segmentation for imaging cardiac electrophysiology. METHODS INF MED. 2007;46(1):36-42.

732. Pytte M, Pedersen TE, Ottem J, Rokvam AS, Sunde K. Comparison of hands-off time during CPR with manual and semi-automatic defibrillation in a manikin model. Resuscitation. 2007;73(1):131-6. doi: 10.1016/j.resuscitation.2006.08.025.

733. Rahmati A, Zhong L. Context-for-wireless: context-sensitive energy-efficient wireless data transfer. Proceedings of the 5th international conference on Mobile systems, applications and services; San Juan, Puerto Rico. 1247681: ACM; 2007. p. 165-78.

734. Rasanathan K, Tukuitonga CF. Tobacco smoking prevalence in Pacific Island countries and territories: A review. New Zealand Med J. 2007;120(1263).

735. Rezek I, Roberts SJ, Conradt R. Increasing the depth of anesthesia assessment. IEEE Eng Med Biol Mag. 2007;26(2):64-73. doi: 10.1109/MEMB.2007.335582.

736. Schertler T, Wildermuth S, Teodorovic N, Mayer D, Marincek B, Boehm T. Visualization of congenital thoracic vascular anomalies using multi-detector row computed tomography and two- and three-dimensional post-processing. Eur J Radiol. 2007;61(1):97-119. doi: 10.1016/j.ejrad.2006.08.015.

737. Sousa JP, Cabri J, Donaghy M. Case research in sports physiotherapy: A review of studies. Phys Ther Sport. 2007;8(4):197-206. doi: 10.1016/j.ptsp.2007.02.003.

738. Su SW, Wang L, Celler BG, Savkin AV, Guo Y. Identification and control for heart rate regulation during treadmill exercise. IEEE TRANS BIOMED ENG. 2007;54(7):1238-46. doi: 10.1109/TBME.2007.890738.

739. Syeda-Mahmood T, Wang F, Beymer D, Amir A, Richmond M, Hashmi SN, editors. AALIM: Multimodal mining for cardiac decision support. Computers in Cardiology 2007, CAR 2007; 2007; Durham, NC.

740. Tadejko P, Rakowski W, editors. Hybrid wavelet-mathematical morphology feature extraction for heartbeat classification. EUROCON 2007 - The International Conference on Computer as a Tool; 2007; Warsaw.

741. Tay SC, Primak AN, Fletcher JG, Schmidt B, Amrami KK, Berger RA, et al. Four-dimensional computed tomographic imaging in the wrist: Proof of feasibility in a cadaveric model. Skelet Radiol. 2007;36(12):1163-9. doi: 10.1007/s00256-007-0374-7.

742. Tay SC, Primak AN, Fletcher JG, Schmidt B, Amrami KK, Berger RA, et al. Four-dimensional computed tomographic imaging in the wrist: proof of feasibility in a cadaveric model. Skeletal Radiol. 2007;36(12):1163-9. Epub 2007/09/07. doi: 10.1007/s00256-007-0374-7. PubMed PMID: 17805530.

743. Telles S, Naveen KV, Dash M. Yoga reduces symptoms of distress in tsunami survivors in the Andaman Islands. Evid-Based Complement Altern Med. 2007;4(4):503-9. doi: 10.1093/ecam/nem069

doi:10.1093/ecam/nel114; Swami Gambhirananda. Mandukya Upanisad. Calcutta: Advaita Ashram, 2000Monro, R., Nagarathna, R., Nagendra, H.R., Ford-Kohne, N., (1991) Yoga for Common Ailments, , New York: Simon & Schuster; Tran, M.D., Holly, R.G., Lashbrook, J., Amsterdam, E.A., Effects of hatha yoga practice on the health-related aspects of physical fitness (2001) Prev Cardio, 4, pp. 165-170; Vempati, R.P., Telles, S., Yoga based guided relaxation reduces sympathetic activity judged from baseline levels (2002) Psychol Rep, 90, pp. 487-494; Brown, R.P., Gerbarg, P.L., Sudarshan Kriya Yogic breathing in the treatment of stress, anxiety, and depression, Part II-clinical applications and guidelines (2005) J Altern Complement Med, 11, pp. 711-717; Shannahoff-Khalsa, D.S., Patient perspectives: Kundalini yoga meditation techniques for psycho-oncology and as potential therapies for cancer (2005) Integr Cancer Ther, 4, pp. 87-100; Manjunath, N.K., Telles, S., Influence of yoga and ayurveda on self rated sleep in a geriatric population (2005) Indian J Med Res, 121, pp. 683-690; Khalsa, S.B., Treatment of chronic insomnia with yoga: A preliminary study with sleep-wake diaries (2004) Appl Psychophysiol Biofeedback, 29, pp. 269-278; Telles, S., Nagarathna, R., Nagendra, H.R., Desiraju, T., Physiological changes in sports teachers following 3 months of training in yoga (1993) Indian J Med Sci, 47, pp. 235-238; Telles, S., Narendran, S., Raghuraj, P., Nagarathna, R., Nagendra, H.R., Comparison of changes in autonomic and respiratory parameters of girls after yoga and games at a community home (1997) Percept Mot Skills, 84, pp. 251-257; Telles, S., Srinivas, R.B., Autonomic and respiratory measures in children with impaired vision following yoga and physical activity programs (1999) Int J Rehab Health, 4, pp. 117-122; Ax, A.F., The physiologic differentiation between fear and anger in humans (1953) Psychosomatic Med, 15, pp. 433-442.

744. Thomas J, Rose C, Charpillet F, editors. A support system for ECG segmentation based on hidden Markov models. 29th Annual International Conference of IEEE-EMBS, Engineering in Medicine and Biology Society, EMBC'07; 2007; Lyon.

745. Thomas RE, Crutcher R, Lorenzetti D. A systematic review of the methodological quality and outcomes of RCTs to teach medical undergraduates surgical and emergency procedures. Can J Surg. 2007;50(4):278-90.

746. Vayssière C, David E, Meyer N, Haberstich R, Sebahoun V, Roth E, et al. A French randomized controlled trial of ST-segment analysis in a population with abnormal cardiotocograms during labor. Am J Obstet Gynecol. 2007;197(3):299.e1-.e6. doi: 10.1016/j.ajog.2007.07.007.

747. Wahed MA. Characterization of ECG signals based on Zernike moments and moment invariants. J Eng Appl Sci. 2007;54(2):205-21.

748. Welch SJ, Jones SS, Allen T. Mapping the 24-Hour Emergency Department Cycle to Improve Patient Flow. The Joint Commission Journal on Quality and Patient Safety. 2007;33(5):247-55. doi: http://doi.org/10.1016/S1553-7250(07)33029-8.

749. Wu WH, Batalin MA, Kaiser WJ, Sarrafzadeh M, Bui AAT, editors. A novel method and testbed for sensor management and patient diagnosis. 2007 Joint Workshop on High Confidence Medical Devices, Software, and Systems and Medical Device Plug-and-Play Interoperability, HCMDSS/MDPnP 2007; 2007; Cambridge, MA.

750. Yamamoto K, Izumi H, Kumashiro M. The effect of the balance between operators' processing abilities and required operating speed on operators' task performance and psycho-physiological state during simple repetitive work under time constraints. J UOEH. 2007;29(1):1-26.

751. Zhang J. Effects of exercise and custom-made orthotics on blood pressure and heart rate variability: a randomized controlled pilot study. Journal of Chiropractic Medicine. 2007;6(2):56-65. doi: http://doi.org/10.1016/j.jcme.2007.04.002.

752. 2008 AMTC Scientific Assembly Monday, October 20, 2008. Air Medical Journal. 2008;27(5):230-7. doi: http://doi.org/10.1016/j.amj.2008.07.008.

753. Poster Session III 2008. Journal of the American Society of Echocardiography. 2008;21(5):582-609. doi: http://doi.org/10.1016/j.echo.2008.02.020.

754. Index to Abstract Subjects. Journal of the American Society of Echocardiography. 2008;21(5):636-74. doi: http://doi.org/10.1016/S0894-7317(08)00234-4.

755. Index to Abstract Authors. Journal of the American Society of Echocardiography. 2008;21(5):610-35. doi: http://doi.org/10.1016/S0894-7317(08)00233-2.

756. Sessions Posters. Réanimation. 2008;17, Supplement 1:S61-S229. doi: http://doi.org/10.1016/j.reaurg.2007.11.004.

757. Al-Ani T, Karmakar CK, Khandoker AH, Palaniswami M, editors. Automatic recognition of obstructive sleep apnoea syndrome using power spectral analysis of electrocardiogram and hidden markov models. 2008 International Conference on Intelligent Sensors, Sensor Networks and Information Processing, ISSNIP 2008; 2008; Sydney, NSW.

758. Anderson M, Leflore J. Playing It Safe: Simulated Team Training in the OR. AORN Journal. 2008;87(4):772-9. doi: http://doi.org/10.1016/j.aorn.2007.12.027.

759. Andreão RV, Muller SMT, Boudy J, Dorizzi B, Bastos-Filho TF, Sarcinelli-Filho M. Incremental HMM training applied to ECG signal analysis. Comput Biol Med. 2008;38(6):659-67. doi: 10.1016/j.compbiomed.2008.03.006.

760. Arends LR, Hamza TH, Van Houwelingen JC, Heijenbrok-Kal MH, Hunink MGM, Stijnen T. Bivariate random effects meta-analysis of ROC curves. Med Decis Mak. 2008;28(5):621-38. doi: 10.1177/0272989X08319957.

761. Asl BM, Setarehdan SK, Mohebbi M. Support vector machine-based arrhythmia classification using reduced features of heart rate variability signal. Artif Intell Med. 2008;44(1):51-64. doi: 10.1016/j.artmed.2008.04.007.

762. Bardo DME, Brown P. Cardiac multidetector computed tomography: Basic physics of image acquisition and clinical applications. Curr Cardiol Rev. 2008;4(3):231-43. doi: 10.2174/157340308785160615.

763. Baskin C, Seetharamu N, Mazure B, Vassallo L, Steinberg H, Kerpen H, et al. Effect of a CD-ROM-based educational intervention on resident knowledge and adherence to deep venous thrombosis prophylaxis guidelines. J Hosp Med. 2008;3(1):42-7. doi: 10.1002/jhm.266.

764. Benitez R, Nenadic Z. Robust unsupervised detection of action potentials with probabilistic models. IEEE TRANS BIOMED ENG. 2008;55(4):1344-54. doi: 10.1109/TBME.2007.912433.

765. Berner ES, Graber ML. Overconfidence as a Cause of Diagnostic Error in Medicine. The American Journal of Medicine. 2008;121(5, Supplement):S2-S23. doi: http://doi.org/10.1016/j.amjmed.2008.01.001.

766. Bohn A, Gude P. Feedback during cardiopulmonary resuscitation. Curr Opin Anaesthesiol. 2008;21(2):200-3. doi: 10.1097/ACO.0b013e3282f63f12.

767. Bohn A, Gude P. Feedback during cardiopulmonary resuscitation. Curr Opin Anaesthesiol. 2008;21(2):200-3. Epub 2008/04/30. doi: 10.1097/ACO.0b013e3282f63f12. PubMed PMID: 18443489.

768. Bosquet L, Camelin FX, Berthoin S. Reliability of postexercise heart rate recovery. INT J SPORTS MED. 2008;29(3):238-43. doi: 10.1055/s-2007-965162.

769. Botchen RP, Bachthaler S, Schick F, Chen M, Mori G, Weiskopf D, et al. Action-based multifield video visualization. IEEE Trans Visual Comput Graphics. 2008;14(4):885-99. doi: 10.1109/TVCG.2008.40.

770. Burke JF, Gnall E, Umrudden Z, Kyaw M, Schick PK. Critical analysis of a computer-assisted tutorial on ECG interpretation and its ability to determine competency. Med Teach. 2008;30(2):e41-8. Epub 2008/05/09. doi: 10.1080/01421590801972471. PubMed PMID: 18464131.

771. Bursa M, Huptych M, Lhotska L, editors. Ant colony inspired metaheuristics in biological signal processing - Hybrid ant colony and evolutionary approach. BIOSIGNALS 2008 - 1st International Conference on Bio-inspired Systems and Signal Processing; 2008; Funchal, Madeira.

772. Calder S. Clinical Pearls and Pitfalls of Electrocardiogram Interpretation in Acute Myocardial Infarction. Journal of Emergency Nursing. 2008;34(4):324-9. doi: 10.1016/j.jen.2007.08.003.

773. Caner C, Engin M, Engin EZ. The programmable ECG simulator. J Med Syst. 2008;32(4):355-9. doi: 10.1007/s10916-008-9140-1.

774. Caner C, Engin M, Engin EZ. The programmable ECG simulator. J Med Syst. 2008;32(4):355-9. Epub 2008/07/16. PubMed PMID: 18619099.

775. Cheah TCS, Kumar A, editors. A simple pulse analyzer for device with limited computational power. 5th International Workshop on Wearable and Implantable Body Sensor Networks, BSN 2008, in conjunction with the 5th International Summer School and Symposium on Medical Devices and Biosensors, ISSS-MDBS 2008; 2008; Hong Kong.

776. Chizner MA. Cardiac Auscultation: Rediscovering the Lost Art. Curr Probl Cardiol. 2008;33(7):326-408. doi: 10.1016/j.cpcardiol.2008.03.003.

777. Chou KT, Yu SN, editors. Categorizing heartbeats by independent component analysis and support vector machines. 8th International Conference on Intelligent Systems Design and Applications, ISDA 2008; 2008; Kaohsiung.

778. Chudacek V, Spilka J, Rubackova B, Koucky M, Georgoulas G, Lhotska L, et al., editors. Evaluation of feature subsets for classification of cardiotocographic recordings. Computers in Cardiology 2008, CAR; 2008; Bologna.

779. Cormier E. Attention Deficit/Hyperactivity Disorder: A Review and Update. Journal of Pediatric Nursing. 2008;23(5):345-57. doi: http://doi.org/10.1016/j.pedn.2008.01.003.

780. Coussement K, Van den Poel D. Churn prediction in subscription services: An application of support vector machines while comparing two parameter-selection techniques. Expert Sys Appl. 2008;34(1):313-27. doi: 10.1016/j.eswa.2006.09.038.

781. Dan C, He W, Zhou J, Li X, editors. Playing and acquiring heart sounds and electrocardiogram simultaneously based on LabVIEW. 2008 World Automation Congress, WAC 2008; 2008; Waikoloa, HI.

782. Dimoulas C, Kalliris G, Papanikolaou G, Petridis V, Kalampakas A. Bowel-sound pattern analysis using wavelets and neural networks with application to long-term, unsupervised, gastrointestinal motility monitoring. Expert Sys Appl. 2008;34(1):26-41. doi: 10.1016/j.eswa.2006.08.014.

783. Dong J, Zhang S, Wan Y, editors. A hybrid framework for ECG interpretation by computer and its evaluation platform. BioMedical Engineering and Informatics: New Development and the Future - 1st International Conference on BioMedical Engineering and Informatics, BMEI 2008; 2008; Sanya, Hainan.

784. Dumont J, Hernández AI, Fleureau J, Carrault G, editors. Modelling temporal evolution of cardiac electrophysiological features using Hidden Semi-Markov Models. 30th Annual International Conference of the IEEE Engineering in Medicine and Biology Society, EMBS'08; 2008; Vancouver, BC.

785. Emoto M, Sugawara M, Nojiri Y. Viewing angle dependency of visually-induced motion sickness in viewing wide-field images by subjective and autonomic nervous indices. Disp. 2008;29(2):90-9. doi: 10.1016/j.displa.2007.09.010.

786. Epstein AE, DiMarco JP, Ellenbogen KA, Estes Iii NAM, Freedman RA, Gettes LS, et al. ACC/AHA/HRS 2008 Guidelines for Device-Based Therapy of Cardiac Rhythm Abnormalities. A Report of the American College of Cardiology/American Heart Association Task Force on Practice Guidelines (Writing Committee to Revise the ACC/AHA/NASPE 2002 Guideline Update for Implantation of Cardiac Pacemakers and Antiarrhythmia Devices). J Am Coll Cardiol. 2008;51(21):e1-e62. doi: 10.1016/j.jacc.2008.02.032.

787. Epstein AE, DiMarco JP, Ellenbogen KA, Estes Iii NAM, Freedman RA, Gettes LS, et al. ACC/AHA/HRS 2008 Guidelines for Device-Based Therapy of Cardiac Rhythm Abnormalities. Heart Rhythm. 2008;5(6):e1-e62. doi: 10.1016/j.hrthm.2008.04.014.

788. Epstein RH, Dexter F, Piotrowski E. Automated correction of room location errors in anesthesia information management systems. ANESTH ANALG. 2008;107(3):965-71. doi: 10.1213/ane.0b013e31817e7b99.

789. Freeman K, Denham SA. Improving Patient Satisfaction by Addressing Same Day Surgery Wait Times. Journal of PeriAnesthesia Nursing. 2008;23(6):387-93. doi: http://doi.org/10.1016/j.jopan.2008.08.003.

790. Galanti G, Pizzi A, Lucarelli M, Stefani L, Gianassi M, Di Tante V, et al. The cardiovascular profile of soccer referees: An echocardiographic study. Cardiovasc Ultrasound. 2008;6. doi: 10.1186/1476-7120-6-8.

791. Galanti G, Pizzi A, Lucarelli M, Stefani L, Gianassi M, Di Tante V, et al. The cardiovascular profile of soccer referees: an echocardiographic study. Cardiovasc Ultrasound. 2008;6:8. Epub 2008/02/14. doi: 10.1186/1476-7120-6-8. PubMed PMID: 18269755; PubMed Central PMCID: PMCPMC2259300.

792. Gharaviri A, Dehghan F, Teshnelab M, Moghaddam HA, editors. Comparison of neural network, ANFIS, and SVM classifiers for PVC arrhythmia detection. 7th International Conference on Machine Learning and Cybernetics, ICMLC; 2008; Kunming.

793. Gon B, #231, alves, Jos, #233, Filho GP, et al. A service architecture for sensor data provisioning for context-aware mobile applications. Proceedings of the 2008 ACM symposium on Applied computing; Fortaleza, Ceara, Brazil. 1364155: ACM; 2008. p. 1946-52.

794. Gon\ B, \#231, alves, Zamborlini V, Guizzardi G, Jos\, et al. Using a lightweight ontology of heart electrophysiology in an interactive web application. Companion Proceedings of the XIV Brazilian Symposium on Multimedia and the Web; Vila Velha, Esp\&iacute;rito Santo, Brazil. 1810001: ACM; 2008. p. 77-80.

795. Gore T, Hunt CW, Raines KH. Mock Hospital Unit Simulation: A Teaching Strategy to Promote Safe Patient Care. Clinical Simulation in Nursing. 2008;4(3):e57-e64. doi: http://doi.org/10.1016/j.ecns.2008.08.006.

796. Gören JL, Parks JJ, Ghinassi FA, Milton CG, Oldham JM, Hernandez P, et al. When Is Antipsychotic Polypharmacy Supported by Research Evidence? Implications for QI. The Joint Commission Journal on Quality and Patient Safety. 2008;34(10):571-82. doi: http://doi.org/10.1016/S1553-7250(08)34072-0.

797. Görges M, Staggers N. Evaluations of physiological monitoring displays: A systematic review. J Clin Monit Comput. 2008;22(1):45-66. doi: 10.1007/s10877-007-9106-8.

798. Gregg RE, Zhou SH, Lindauer JM, Helfenbein ED, Giuliano KK. What is inside the electrocardiograph? J Electrocardiol. 2008;41(1):8-14. doi: 10.1016/j.jelectrocard.2007.08.059.

799. Gregg RE, Zhou SH, Lindauer JM, Helfenbein ED, Giuliano KK. What is inside the electrocardiograph? J Electrocardiol. 2008;41(1):8-14. Epub 2008/01/15. doi: 10.1016/j.jelectrocard.2007.08.059. PubMed PMID: 18191652.

800. Hall EJ, Brenner DJ. Cancer risks from diagnostic radiology. Br J Radiol. 2008;81(965):362-78. doi: 10.1259/bjr/01948454.

801. Jin Z, Cheng AC. ImplantBench: Characterizing and projecting representative benchmarks for emerging bioimplantable computing. IEEE Micro. 2008;28(4):71-91. doi: 10.1109/MM.2008.55.

802. Kasamatsu T, Hashimoto J, Iyatomi H, Nakahara T, Bai J, Kitamura N, et al. Application of support vector machine classifiers to preoperative risk stratification with myocardial perfusion scintigraphy. Circ J. 2008;72(11):1829-35. doi: 10.1253/circj.CJ-08-0236.

803. Lannfelt L, Blennow K, Zetterberg H, Batsman S, Ames D, Harrison J, et al. Safety, efficacy, and biomarker findings of PBT2 in targeting Aβ as a modifying therapy for Alzheimer's disease: a phase IIa, double-blind, randomised, placebo-controlled trial. Lancet Neurol. 2008;7(9):779-86. doi: 10.1016/S1474-4422(08)70167-4.

804. Laschinger S, Medves J, Pulling C, McGraw R, Waytuck B, Harrison MB, et al. Effectiveness of simulation on health profession students' knowledge, skills, confidence and satisfaction. Int J Evid-Based Healthc. 2008;6(3):278-302. doi: 10.1111/j.1479-6988.2008.00108.x.

805. Lee JS, Cho BH, Chee YJ, Kim IY, Kim SI. A new approach for personal identification based on dVCG. IEICE Trans Inf Syst. 2008;E91-D(4):1201-5. doi: 10.1093/ietisy/e91-d.4.1201.

806. Lei WK, Dong MC, Shi J, Fu BB, editors. Automatic ECG interpretation via morphological feature extraction and SVM inference nets. APCCAS 2008 - 2008 IEEE Asia Pacific Conference on Circuits and Systems; 2008; Macao.

807. Lessard Y, Sinteff JP, Siregar P, Julen N, Hannouche F, Rio S, et al., editors. Oaat, a new, simple and powerfull interactive tool for ecg analysis learning. e-Learning 2008, MCCSIS'08 - IADIS Multi Conference on Computer Science and Information Systems; 2008; Amsterdam.

808. Maier C, Khalil M, Ulmer H, Dickhaus H. Precursors of syncope in linear and non-linear parameters of heart rate variability during pediatric head-up tilt test. Biomed Tech (Berl). 2008;53(3):145-55. doi: 10.1515/BMT.2008.014.

809. Malarvili MB, Mesbah M, editors. Combining newborn EEG and HRV information for automatic seizure detection. 30th Annual International Conference of the IEEE Engineering in Medicine and Biology Society, EMBS'08; 2008; Vancouver, BC.

810. Mansor S, Noble JA, editors. Local wall motion classification of stress echocardiography using a hidden Markov model approach. 2008 5th IEEE International Symposium on Biomedical Imaging: From Nano to Macro, ISBI; 2008; Paris.

811. Meek T. Anaesthetic simulators: Making the most of your purchase. Current Anaesthesia & Critical Care. 2008;19(5–6):354-60. doi: http://doi.org/10.1016/j.cacc.2008.07.010.

812. Mehta SS, Lingayat NS. Development of SVM based classification techniques for the delineation of wave components in 12-lead electrocardiogram. Biomed Signal Process Control. 2008;3(4):341-9. doi: 10.1016/j.bspc.2008.04.002.

813. Merzougui R, Feham M, editors. Algorithm of remote monitoring ECG using mobile phone: Conception and implementation. 3rd International Conference on Broadband Communications, Informatics and Biomedical Applications, BroadCom 2008; 2008; Pretoria, Gauteng.

814. Milanesi M, Martini N, Vanello N, Positano V, Santarelli MF, Landini L. Independent component analysis applied to the removal of motion artifacts from electrocardiographic signals. Med Biol Eng Comput. 2008;46(3):251-61. doi: 10.1007/s11517-007-0293-8.

815. Min JK, Lin FY. What makes a coronary CT angiogram nondiagnostic? J Cardiovasc Comput Tomogr. 2008;2(6):351-9. doi: 10.1016/j.jcct.2008.10.011.

816. Mulvagh SL, Rakowski H, Vannan MA, Abdelmoneim SS, Becher H, Bierig SM, et al. American Society of Echocardiography Consensus Statement on the Clinical Applications of Ultrasonic Contrast Agents in Echocardiography. Journal of the American Society of Echocardiography. 2008;21(11):1179-201. doi: http://doi.org/10.1016/j.echo.2008.09.009.

817. Myers J, Arena R, Dewey F, Bensimhon D, Abella J, Hsu L, et al. A cardiopulmonary exercise testing score for predicting outcomes in patients with heart failure. Am Heart J. 2008;156(6):1177-83. doi: 10.1016/j.ahj.2008.07.010.

818. Nacke L, Lindley CA. Flow and immersion in first-person shooters: measuring the player's gameplay experience. Proceedings of the 2008 Conference on Future Play: Research, Play, Share; Toronto, Ontario, Canada. 1496998: ACM; 2008. p. 81-8.

819. Nacke L, Lindley CA, editors. Flow and immersion in first-person shooters: Measuring the player's gameplay experience. 2008 Conference on Future Play: Research, Play, Share, Future Play 2008; 2008; Toronto, ON.

820. Odom-Forren J. Perioperative Patient Safety and Procedural Sedation. Perioperative Nursing Clinics. 2008;3(4):355-66. doi: http://doi.org/10.1016/j.cpen.2008.08.010.

821. Phua K, Chen J, Dat TH, Shue L. Heart sound as a biometric. Pattern Recogn. 2008;41(3):906-19. doi: 10.1016/j.patcog.2007.07.018.

822. Posada-Gomez R, Enriquez-Rodriguez JJ, Alor-Hernandez G, Martinez-Sibaja A, editors. USB bulk transfers between a PC and a PIC microcontroller for embedded applications. Proceedings - 5th Meeting of the Electronics, Robotics and Automotive Mechanics Conference 2008, CERMA 2008; 2008; Cuernavaca, Morelos.

823. Pradhan GN, Prabhakaran B. Storage, retrieval, and communication of body sensor network data. Proceedings of the 16th ACM international conference on Multimedia; Vancouver, British Columbia, Canada. 1459617: ACM; 2008. p. 1161-2.

824. Rabe-Hesketh S, Skrondal A. Classical latent variable models for medical research. Stat Methods Med Res. 2008;17(1):5-32. doi: 10.1177/0962280207081236.

825. Raines DA. A competency-based approach to the nursing research. Nurse Education in Practice. 2008;8(6):373-81. doi: http://doi.org/10.1016/j.nepr.2008.03.004.

826. Rasku J, Juhola M, Tossavainen T, Pyykkö I, Toppila E. Modelling stabilograms with hidden Markov models. J Med Eng Technol. 2008;32(4):273-83. doi: 10.1080/03091900600968908.

827. Rybicki FJ, Otero HJ, Steigner ML, Vorobiof G, Nallamshetty L, Mitsouras D, et al. Initial evaluation of coronary images from 320-detector row computed tomography. Int J Card Imaging. 2008;24(5):535-46. doi: 10.1007/s10554-008-9308-2.

828. Samanta B, Nataraj C, editors. Prognostics using morphological signal processing and computational intelligence. 2008 International Conference on Prognostics and Health Management, PHM 2008; 2008; Denver.

829. Saremi F, Grizzard JD, Kim RJ. Optimizing cardiac MR imaging: Practical remedies for artifacts. Radiographics. 2008;28(4):1161-87. doi: 10.1148/rg.284065718.

830. Schuetz M, Moenk S, Vollmer J, Kurz S, Mollnau H, Post F, et al. High degree of realism in teaching percutaneous coronary interventions by combining a virtual reality trainer with a full scale patient simulator. Simul Healthc. 2008;3(4):242-6. Epub 2008/12/18. doi: 10.1097/SIH.0b013e3181871b58. PubMed PMID: 19088669.

831. Sharma T, Bhardwaj S, Maringanti HB, editors. Emotion Estimation using Physiological Signals. 2008 IEEE Region 10 Conference, TENCON 2008; 2008; Hyderabad.

832. Shilkofski NA, Nelson KL, Hunt EA. Recognition and treatment of unstable supraventricular tachycardia by pediatric residents in a simulation scenario. Simul Healthc. 2008;3(1):4-9. Epub 2008/12/18. doi: 10.1097/SIH.0b013e31815bfa4e. PubMed PMID: 19088636.

833. Smith HM, Jacob AK, Segura LG, Dilger JA, Torsher LC. Simulation education in anesthesia training: A case report of successful resuscitation of bupivacaine-induced cardiac arrest linked to recent simulation training. ANESTH ANALG. 2008;106(5):1581-4. doi: 10.1213/ane.0b013e31816b9478.

834. Snider S, Morse D, Chen G, Apte SV, Liburdy JA, Zhang E, editors. Detection and analysis of separated flow induced vortical structures. 46th AIAA Aerospace Sciences Meeting and Exhibit; 2008; Reno, NV.

835. Sprick C, Ruthenbeck GS, Owen H, Reynolds KJ, editors. Virtual patient monitors for new user familiarization. Medicine Meets Virtual Reality 16 - Parallel, Combinatorial, Convergent: NextMed by Design, MMVR 2008; 2008; Long Beach, CA18391350.

836. Staats C, Austin D, Aboy M. A statistical model and simulator for cardiovascular pressure signals. Proc Inst Mech Eng Part H J Eng Med. 2008;222(6):991-8. doi: 10.1243/09544119JEIM348.

837. Sun G, Wang Z, Wang M, editors. A new multi-classification method based on binary tree support vector machine. 3rd International Conference on Innovative Computing Information and Control, ICICIC'08; 2008; Dalian, Liaoning.

838. Tobon-Gomez C, Butakoff C, Aguade S, Sukno F, Moragas G, Frangi AF. Automatic construction of 3D-ASM intensity models by simulating image acquisition: Application to myocardial gated SPECT studies. IEEE Trans Med Imaging. 2008;27(11):1655-67. doi: 10.1109/TMI.2008.2004819.

839. Wahi A, Thirumurugan E, editors. Recognition of objects by supervised neural network using wavelet features. 1st International Conference on Emerging Trends in Engineering and Technology, ICETET 2008; 2008; Nagpur, Maharashtra.

840. Wai KL, Bing NL, Ming CD, Bin BF, editors. An application of morphological feature extraction and support vector machines in computerized ECG interpretation. 2007 6th Mexican International Conference on Artificial Intelligence, Special Session, MICAI 2007; 2008; Aguascalientes.

841. Weghorst S, Seibel E, Oppenheimer P, Hoffman H, Schowengerdt B, Furness TA. Medical interface research at the HIT Lab. Virtual Reality. 2008;12(4):201-14. doi: 10.1007/s10055-008-0107-9.

842. Wiggins M, Saad A, Litt B, Vachtsevanos G. Evolving a Bayesian classifier for ECG-based age classification in medical applications. Appl Soft Comput J. 2008;8(1):599-608. doi: 10.1016/j.asoc.2007.03.009.

843. Wong EM-L, Lau PF. Evolving towards professionalism in emergency nursing in Hong Kong. International Emergency Nursing. 2008;16(1):53-8. doi: http://doi.org/10.1016/j.ienj.2007.11.008.

844. Wu WH, Bui AAT, Batalin MA, Au LK, Binney JD, Kaiser WJ. MEDIC: Medical embedded device for individualized care. Artif Intell Med. 2008;42(2):137-52. doi: 10.1016/j.artmed.2007.11.006.

845. Yadollahi A, Moussavi Z. Respiratory sounds compression. IEEE TRANS BIOMED ENG. 2008;55(4):1336-43. doi: 10.1109/TBME.2007.912421.

846. Yaghoubian A, Saltmarsh G, Rosing DK, Lewis RJ, Stabile BE, De Virgilio C. Decreased bile duct injury rate during laparoscopic cholecystectomy in the era of the 80-hour resident workweek. Arch Surg. 2008;143(9):847-51. doi: 10.1001/archsurg.143.9.847

10.1016/S1072-7515 (02)01242-5; de Virgilio, C., Yaghoubian, A., Lewis, R.J., Stabile, B.E., Putnam, B.A., The 80-hour resident workweek does not adversely affect patient outcomes or resident education (2006) Curr Surg, 63 (6), pp. 435-439; Naylor, R.A., Rege, R.V., Valentine, R.J., Do resident duty hour restrictions reduce technical complications of emergency lapa roscopic cholecystectomy? (2005) J Am Coll Surg, 201 (5), pp. 724-731; Schenarts, P., Bowen, J., Bard, M., The effect of a rotating night-float coverage scheme on preventable and potentially preventable morbidity at a level 1 trauma center (2005) Am J Surg, 190 (1), pp. 147-152; Kaafarani, H.M., Itani, K.M., Petersen, L.A., Thornby, J., Berger, D.H., Does resident hours reduction have an Impact on surgical outcomes? (2005) J Surg Res, 126 (2), pp. 167-171; Goldman, L.I., McDonough, M.T., Rosemond, G.P., Stresses affecting surgical performance and learning, I: Correlation of heart rate, electrocardiogram and operation simultaneously recorded on videotapes (1972) J Surg Res, 12 (2), pp. 83-86; Taffinder, N.J., McManus, I.C., Gul, Y., Russell, R.C., Darzi, A., Effect of sleep deprivation on surgeons' dexterity on laparoscopy simulator [letter] (1998) Lancet, 352 (9135), p. 1191; Grantcharov, T.P., Bardram, L., Funch-Jensen, P., Rosenberg, J., Laparoscopic performance after one night on call In a surgical department: Prospective study (2001) BMJ, 323 (7323), pp. 1222-1223; Fletcher, D.R., Hobbs, M.S., Tan, P., Complications of cholecystectomy: Risks of the laparoscopic approach and protective effects of operative cholangiography: a population-based study (1999) Ann Surg, 229 (4), pp. 449-457; Giger, U.F., Michel, J.M., Opitz l, T., Inderbitzin, D., Kocher, T., Krähenbühl, L., Swiss Association of Laparoscopic and Thoracoscopic Surgery (SALTS) Study Group. Risk factors for perioperative complications In patients undergoing laparoscopic cholecystectomy: Analysis of 22 953 consecutive cases from the Swiss Association of Laparoscopic and Thoracoscopic Surgery database (2006) J Am Coll Surg, 203 (5), pp. 723-728; Krähenbühl, L., Sclabas, G., Wente, M.N., Schäfer, M., Schlumpf, R., Büchler, M.W., Incidence, risk factors, and prevention of biliary tract Injuries during laparoscopic cholecystectomy In Switzerland (2001) World J Surg, 25 (10), pp. 1325-1330; Schol, F.P., Go, P.M., Gouma, D.J., Risk factors for bile duct Injury in laparoscopic cholecystectomy: Analysis of 49 cases (1994) Br J Surg, 81 (12), pp. 1786-1788; Grönroos, J.M., Hämäläinen, M.T., Karvonen, J., Gullichsen, R., Laine, S., Is male gender a risk factor for bile duct Injury during laparoscopic cholecystectomy? (2003) Lan-genbecks Arch Surg, 388 (4), pp. 261-264; Way, L.W., Stewart, L., Gantert, W., Causes and prevention of laparoscopic bile duct injuries: Analysis of 252 cases from a human factors and cognitive psychology perspective (2003) Ann Surg, 237 (4), pp. 460-469; GigotJF, Bile duct injury during laparoscopic cholecystectomy: Risk factors, mechanisms, type, severity and Immediate detection (2003) Acta Chir Belg, 103 (2), pp. 154-160; Andrén-Sandberg, A., Alinder, G., Beng mark, S., Accidental lesions of the common bile duct at cholecystectomy: Pre- and perioperative factors of importance (1985) Ann Surg, 201 (3), pp. 328-332; Kum, C.K., Eypasch, E., Lefering, R., Paul, A., Neugebauer, E., Troidl, H., Laparoscopic cholecystectomy for acute cholecystitis: Is it really safe? (1996) World J Surg, 20 (1), pp. 43-49; Deziel, D.J., Millikan, K.W., Economou, S.G., Doolas, A., Ko, S.T., Airan, M.C., Complications of laparoscopic cholecystectomy: A national survey of 4292 hospitals and an analysis of 77 604 cases (1993) Am J Surg, 165 (1), pp. 9-14; Firth-Cozens, J., Greenhalgh, J., Doctors' perceptions of the links between stress and lowered clinical care (1997) Soc Sci Med, 44 (7), pp. 1017-1022; Ellman, P.I., Kron, I.L., Alvis, J.S., Acute sleep deprivation In the thoracic surgical resident does not affect operative outcomes (2005) Ann Thorac Surg, 80 (1), pp. 60-64.

847. Yang G, Lin Y, Bhattacharya P. Multimodality inferring of human cognitive states based on integration of neuro-fuzzy network and information fusion techniques. Eurasip J Adv Sign Process. 2008;2008. doi: 10.1155/2008/371621.

848. Zhang G, Dilling TJ, Stevens CW, Forster KM. Functional lung imaging in thoracic cancer radiotherapy. Cancer Control. 2008;15(2):112-9.

849. Zhang Y, Zhang Q, Wu S, editors. Biomedical signal detection based on fractional fourier transform. 5th International Conference on Information Technology and Applications in Biomedicine, ITAB 2008 in conjunction with 2nd International Symposium and Summer School on Biomedical and Health Engineering, IS3BHE 2008; 2008; Shenzhen.

850. 23rd Annual Conference. Journal of the Association for Vascular Access. 2009;14(4):171-86. doi: http://doi.org/10.1016/S1552-8855(09)70071-6.

851. The International Olympic Committee (IOC) consensus statement on periodic health evaluation of elite athletes: March 2009. J Athl Train. 2009;44(5):538-57. doi: 10.4085/1062-6050-44.5.538.

852. Web scan. Emerg Nurse. 2009;16(10):4. Epub 2009/03/10. doi: 10.7748/en.16.10.4.s9. PubMed PMID: 27644355.

853. Al-Sawalmeh W, Daqrouq K, Al-Qawasmi AR, Hilal TA. The use of wavelets in speaker feature tracking identification system using neural network. WSEAS Trans Signal Process. 2009;5(5):167-77.

854. Anuar SHB, Elamvazuthi I, Hanif NHHBM, editors. Blood pressure measuring device embedded with SMS capabilities. 2009 IEEE Student Conference on Research and Development, SCOReD2009; 2009; Serdang.

855. Biederman RWW. Cardiovascular magnetic resonance imaging as applied to patients with pulmonary arterial hypertension. Int J Clin Pract. 2009;63(SUPPL. 162):20-35. doi: 10.1111/j.1742-1241.2009.02109.x.

856. Boumbarov O, Velchev Y, Sokolov S, editors. ECG personal identification in subspaces using radial basis neural networks. 5th IEEE International Workshop on Intelligent Data Acquisition and Advanced Computing Systems: Technology and Applications, IDAACS'2009; 2009; Rende.

857. Brook RD, Urch B, Dvonch JT, Bard RL, Speck M, Keeler G, et al. Insights into the mechanisms and mediators of the effects of air pollution exposure on blood pressure and vascular function in healthy humans. Hypertension. 2009;54(3):659-67. doi: 10.1161/HYPERTENSIONAHA.109.130237.

858. Brown D, Chronister C. The Effect of Simulation Learning on Critical Thinking and Self-confidence When Incorporated Into an Electrocardiogram Nursing Course. Clinical Simulation in Nursing. 2009;5(1):e45-e52. doi: http://doi.org/10.1016/j.ecns.2008.11.001.

859. Brown SJ, Brown JA. Heart rate variability and ventilatory efficiency. INT J SPORTS MED. 2009;30(7):496-502. doi: 10.1055/s-0028-1112146.

860. Carabalona R, Castiglioni P, Gramatica F, editors. Brain-computer interfaces and neurorehabilitation. Stud Health Technol Informatics; 2009 19592793.

861. Chalaye P, Goffaux P, Lafrenaye S, Marchand S. Respiratory effects on experimental heat pain and cardiac activity. Pain Med (USA). 2009;10(8):1334-40. doi: 10.1111/j.1526-4637.2009.00681.x.

862. Chang PC, Hsieh JC, Lin JJ, Chou YH, Liu CH, editors. A Hybrid System with Hidden Markov Models and Gaussian Mixture Models for Myocardial Infarction Classification with 12-Lead ECGs. 11th IEEE International Conference on High Performance Computing and Communications, HPCC 2009; 2009; Seoul.

863. Chiu SC, Cheng KY, Sun TK, Chang KC, Tan TY, Lin TK, et al. The effectiveness of interactive computer assisted instruction compared to videotaped instruction for teaching nurses to assess neurological function of stroke patients: A randomized controlled trial. International Journal of Nursing Studies. 2009;46(12):1548-56. doi: 10.1016/j.ijnurstu.2009.05.008.

864. Chu CM, Chien WC, Lai CH, Bludau HB, Tschai HJ, Pai L, et al. A Bayesian expert system for clinical detecting coronary artery disease. J Med Sci(Taiwan). 2009;29(4):187-94.

865. Clark SL. Sleep deprivation: implications for obstetric practice in the United States. Am J Obstet Gynecol. 2009;201(2):136.e1-.e4. doi: 10.1016/j.ajog.2009.01.013.

866. Colloca L, Benedetti F. Placebo analgesia induced by social observational learning. PAIN®. 2009;144(1–2):28-34. doi: http://doi.org/10.1016/j.pain.2009.01.033.

867. Colloca L, Benedetti F. Placebo analgesia induced by social observational learning. Pain [Internet]. 2009; 144(1-2):[28-34 pp.]. Available from: http://onlinelibrary.wiley.com/o/cochrane/clcentral/articles/290/CN-00697290/frame.html.

868. Colloca L, Benedetti F. Placebo analgesia induced by social observational learning. Pain. 2009;144(1-2):28-34. Epub 2009/03/13. doi: 10.1016/j.pain.2009.01.033. PubMed PMID: 19278785.

869. Czum JM. Evidence-based Methodology for Evaluation of Coronary Computed Tomographic Angiography: Reflecting on a Self-Directed Learning Experience. Seminars in Roentgenology. 2009;44(3):201-8. doi: http://doi.org/10.1053/j.ro.2009.03.017.

870. Devi G, Sarma KK, Datta P, Mahanta AK. ANN based multi classifier system for prediction of high energy shower primary energy and core location. World Acad Sci Eng Technol. 2009;39:890-9.

871. Du BX, Liu HJ. The application of recurrence plot in DC tracking test of gamma-ray irradiated polycarbonate. IEEE Trans Dielectr Electr Insul. 2009;16(1):17-23. doi: 10.1109/TDEI.2009.4784547.

872. Dussault C, Lely L, Langrume C, Sauvet F, Jouanin JC. Heart rate and autonomic balance during stand tests before and after fighter combat missions. Aviat Space Environ Med. 2009;80(9):796-802. doi: 10.3357/ASEM.2494.2009.

873. ElHelw M, Pansiot J, McIlwraith D, Ali R, Lo B, Atallah L, editors. An integrated multi-sensing framework for pervasive healthcare monitoring. 2009 3rd International Conference on Pervasive Computing Technologies for Healthcare - Pervasive Health 2009, PCTHealth 2009; 2009; London.

874. Elmenhorst EM, Vejvoda M, Maass H, Wenzel J, Plath G, Schubert E, et al. Workload during approaches: Comparison of simulated standard and noise-abatement profiles. Aviat Space Environ Med. 2009;80(4):364-70. doi: 10.3357/ASEM.2382.2009.

875. Figueredo VM. The Time Has Come for Physicians to Take Notice: The Impact of Psychosocial Stressors on the Heart. The American Journal of Medicine. 2009;122(8):704-12. doi: http://doi.org/10.1016/j.amjmed.2009.05.001.

876. Flavell EM, Stacey MR, Hall JE. The clinical management of airway obstruction. Current Anaesthesia & Critical Care. 2009;20(3):102-12. doi: http://doi.org/10.1016/j.cacc.2009.02.004.

877. Frénay B, de Lannoy G, Verleysen M, editors. Improving the transition modelling in hidden markov models for ECG segmentation. 17th European Symposium on Artificial Neural Networks - Advances in Computational Intelligence and Learning, ESANN 2009; 2009; Bruges.

878. Gade L, Krishna S, Panchanathan S, editors. Person localization using a wearable camera towards enhancing social interactions for individuals with visual impairment. 1st ACM SIGMM International Workshop on Media Studies and Implementations that Help Improving Access to Disabled Users, MSIADU'09, Co-located with the 2009 ACM International Conference on Multimedia, MM'09; 2009; Beijing.

879. Gomes MM, Higgins AL, Butler R, Farzaneh JR. Anatomy of a Staged Orientation Process. Journal of Emergency Nursing. 2009;35(6):575-9. doi: http://doi.org/10.1016/j.jen.2009.09.014.

880. Gomez BT. Assessing Competency With the Use of Human Patient Simulation in the Emergency Department. Journal of Emergency Nursing. 2009;35(5):476-8. doi: http://doi.org/10.1016/j.jen.2009.06.012.

881. Graveling AJ, Frier BM. Hypoglycaemia: An overview. Primary Care Diabetes. 2009;3(3):131-9. doi: http://doi.org/10.1016/j.pcd.2009.08.007.

882. Gubbi J, Khandoker A, Palaniswami M, editors. Classification of obstructive and central sleep apnea using wavelet packet analysis of ECG signals. 36th Annual Conference of Computers in Cardiology, CinC 2009; 2009; Park City, UT.

883. Guo Yy, Kang Gx, Cao Y, Zhang P. Emergency access mechanism in IEEE 802.15.4 for wireless body area sensor networks. J China Univ Post Telecom. 2009;16(6):24-31. doi: 10.1016/S1005-8885(08)60284-8.

884. Ho HJ, Chen TC. Motorized CPM/CAM physiotherapy device with sliding-mode Fuzzy Neural Network control loop. COMPUT METHODS PROGRAMS BIOMED. 2009;96(2):96-107. doi: 10.1016/j.cmpb.2009.04.007.

885. Hu F, Xiao Y, Hao Q. Congestion-aware, loss-resilient bio-monitoring sensor networking for mobile health applications. IEEE J Sel Areas Commun. 2009;27(4):450-65. doi: 10.1109/JSAC.2009.090509.

886. Hwang S-L, Liang G-F, Lin J-T, Yau Y-J, Yenn T-C, Hsu C-C, et al. A real-time warning model for teamwork performance and system safety in nuclear power plants. Safety Science. 2009;47(3):425-35. doi: http://doi.org/10.1016/j.ssci.2008.07.011.

887. Ironside PM, Jeffries PR, Martin A. Fostering patient safety competencies using multiple-patient simulation experiences. Nurs Outlook. 2009;57(6):332-7. doi: 10.1016/j.outlook.2009.07.010.

888. Ivarsson M, Anderson M, Åkerstedt T, Lindblad F. Playing a violent television game affects heart rate variability. Acta Paediatr Int J Paediatr. 2009;98(1):166-72. doi: 10.1111/j.1651-2227.2008.01096.x.

889. Jeong K, Jung EY, Park DK, editors. Trend of wireless u-Health. 2009 9th International Symposium on Communications and Information Technology, ISCIT 2009; 2009; Icheon.

890. Joshi AJ, Chandran S, Jayaraman VK, Kulkarni BD, editors. Hybrid SVM for multiclass arrhythmia classification. 2009 IEEE International Conference on Bioinformatics and Biomedicine, BIBM 2009; 2009; Washington, D.C.

891. Jung C, Ferrari M, Goebel B, Figulla HR. The patient's motivation during bicycle stress ECG test is dependent on the investigator's sex in male patients. Int J Cardiol. 2009;136(3):348-51. doi: 10.1016/j.ijcard.2008.04.074.

892. Justus Hofmeyr G, Haws RA, Bergström S, Lee ACC, Okong P, Darmstadt GL, et al. Obstetric care in low-resource settings: What, who, and how to overcome challenges to scale up? Int J Gynecol Obstet. 2009;107(SUPPL.):S21-S45. doi: 10.1016/j.ijgo.2009.07.017.

893. Katerndahl DA. Lessons from Jurassic Park: Patients as complex adaptive systems. J Eval Clin Pract. 2009;15(4):755-60. doi: 10.1111/j.1365-2753.2009.01228.x.

894. Khandoker AH, Karmakar CK, Palaniswami M. Automated recognition of patients with obstructive sleep apnoea using wavelet-based features of electrocardiogram recordings. Comput Biol Med. 2009;39(1):88-96. doi: 10.1016/j.compbiomed.2008.11.003.

895. Kim D-J, Prabhakaran B. Multimedia aspects in health care. Proceedings of the 17th ACM international conference on Multimedia; Beijing, China. 1631453: ACM; 2009. p. 921-2.

896. Kors JA, Van Herpen G. Methodology of QT-interval measurement in the modular ECG analysis system (MEANS). Ann Noninvasive Electrocardiol. 2009;14(SUPPL. 1):S48-S53. doi: 10.1111/j.1542-474X.2008.00261.x.

897. Ladysz R, editor Nonlinear dynamical and entropic complexity measures as indicators of nonstationarities in short-term ECG signals. 2009 IEEE International Conference on Bioinformatics and Biomedicine, BIBM 2009; 2009; Washington, D.C.

898. Lee HJ, Lee SH, Ha K-S, Jang HC, Chung W-Y, Kim JY, et al. Ubiquitous healthcare service using Zigbee and mobile phone for elderly patients. International Journal of Medical Informatics. 2009;78(3):193-8. doi: http://doi.org/10.1016/j.ijmedinf.2008.07.005.

899. Lessard Y, Sinteff JP, Siregar P, Julen N, Hannouche F, Rio S, et al., editors. An ECG analysis interactive training system for understanding arrhythmias. 22nd International Conference on Medical Informatics Europe, MIE 2009; 2009; Sarajevo19745450.

900. Ljungqvist A, Jenoure P, Engebretsen L, Alonso JM, Bahr R, Clough A, et al. The International Olympic Committee (IOC) consensus statement on periodic health evaluation of elite athletes. Int SportMed J. 2009;10(3).

901. Ljungqvist A, Jenoure P, Engebretsen L, Alonso JM, Bahr R, Clough A, et al. The International Olympic Committee (IOC) Consensus Statement on periodic health evaluation of elite athletes March 2009. Br J Sports Med. 2009;43(9):631-43. doi: 10.1136/bjsm.2009.064394.

902. Ljungqvist A, Jenoure PJ, Engebretsen L, Alonso JM, Bahr R, Clough AF, et al. The International Olympic Committee (IOC) consensus statement on periodic health evaluation of elite Athletes, March 2009. Clin J Sport Med. 2009;19(5):347-65. doi: 10.1097/JSM.0b013e3181b7332c.

903. Maglogiannis I, Loukis E, Zafiropoulos E, Stasis A. Support Vectors Machine-based identification of heart valve diseases using heart sounds. COMPUT METHODS PROGRAMS BIOMED. 2009;95(1):47-61. doi: 10.1016/j.cmpb.2009.01.003.

904. Marcelloni F, Vecchio M. An efficient lossless compression algorithm for tiny nodes of monitoring wireless sensor networks. Comput J. 2009;52(8):969-87. doi: 10.1093/comjnl/bxp035.

905. Martis RJ, Chakraborty C, Ray AK. A two-stage mechanism for registration and classification of ECG using Gaussian mixture model. Pattern Recogn. 2009;42(11):2979-88. doi: 10.1016/j.patcog.2009.02.008.

906. McGillivray B, Considine J. Implementation of evidence into practice: Development of a tool to improve emergency nursing care of acute stroke. Australasian Emergency Nursing Journal. 2009;12(3):110-9. doi: http://doi.org/10.1016/j.aenj.2009.03.005.

907. Mehta SS, Lingayat NS. Identification of QRS complexes in 12-lead electrocardiogram. Expert Sys Appl. 2009;36(1):820-8. doi: 10.1016/j.eswa.2007.10.007.

908. Mehta SS, Lingayat NS. Application of support vector machine for the detection of P- and T-waves in 12-lead electrocardiogram. COMPUT METHODS PROGRAMS BIOMED. 2009;93(1):46-60. doi: 10.1016/j.cmpb.2008.07.014.

909. Meng X, Wang J, Pi Y, Yuan Q. A novel Artificial Neural Network training method combined with Quantum Computational Multi-Agent System theory. Int J Intell Syst Technol Appl. 2009;6(1-2):50-60. doi: 10.1504/IJISTA.2009.022687.

910. Miyaji M, Kawanaka H, Oguri K, editors. Driver's cognitive distraction detection using physiological features by the AdaBoost. 2009 12th International IEEE Conference on Intelligent Transportation Systems, ITSC '09; 2009; St. Louis, MO.

911. Mogil JS, Simmonds K, Simmonds MJ. Pain research from 1975 to 2007: A categorical and bibliometric meta-trend analysis of every Research Paper published in the journal, Pain. Pain. 2009;142(1–2):48-58. doi: http://doi.org/10.1016/j.pain.2008.11.012.

912. Neil JA. Simulation in Nursing Education. Perioperative Nursing Clinics. 2009;4(2):97-112. doi: http://doi.org/10.1016/j.cpen.2009.02.002.

913. Nikendei C, Andreesen S, Hoffmann K, Junger J. Cross-year peer tutoring on internal medicine wards: effects on self-assessed clinical competencies--a group control design study. Medical teacher [Internet]. 2009; 31(2):[e32-5 pp.]. Available from: http://onlinelibrary.wiley.com/o/cochrane/clcentral/articles/920/CN-00702920/frame.html.

914. Niyato D, Hossain E, Camorlinga S. Remote patient monitoring service using heterogeneous wireless access networks: Architecture and optimization. IEEE J Sel Areas Commun. 2009;27(4):412-23. doi: 10.1109/JSAC.2009.090506.

915. Otero HJ, Steigner ML, Rybicki FJ. The "Post-64" Era of Coronary CT Angiography: Understanding New Technology from Physical Principles. Radiol Clin North Am. 2009;47(1):79-90. doi: 10.1016/j.rcl.2008.11.001.

916. Pachauri A, Bhuyan M. Robust detection of R-wave using wavelet technique. World Acad Sci Eng Technol. 2009;32:901-5.

917. Pachauri A, Bhuyan M, editors. Wavelet and energy based approach for PVC detection. 2009 International Conference on Emerging Trends in Electronic and Photonic Devices and Systems, ELECTRO '09; 2009; Varanasi.

918. Patel K, Chua CP, Faul S, Bleakley CJ, editors. Low power real-time seizure detection for ambulatory EEG. 2009 3rd International Conference on Pervasive Computing Technologies for Healthcare - Pervasive Health 2009, PCTHealth 2009; 2009; London.

919. Pittiruti M, Scoppettuolo G, LaGreca A. The PICC Project: The Development of a Nationwide Program for the Diffusion of PICC in Italy 2005–2009. Journal of the Association for Vascular Access. 2009;14(4):191-8. doi: http://doi.org/10.2309/java.14-4-4.

920. Pittiruti M, Scoppettuolo G, LaGreca A. The PICC project: The development of a nationwide program for the diffusion of PICC in Italy 2005-2009. JAVA J Assoc Vasc Access. 2009;14(4):191-8. doi: 10.2309/java.14-4-4.

921. Potse M, Dubé B, Vinet A. Cardiac anisotropy in boundary-element models for the electrocardiogram. Med Biol Eng Comput. 2009;47(7):719-29. doi: 10.1007/s11517-009-0472-x.

922. Poungponsri S, Yu XH, editors. Electrocardiogram (ECG) signal modeling and noise reduction using wavelet neural networks. 2009 IEEE International Conference on Automation and Logistics, ICAL 2009; 2009; Shenyang.

923. Rengier F, Weber TF, Giesel FL, Böckler D, Kauczor HU, Von Tengg-Kobligk H. Centerline analysis of aortic CT angiographic examinations: Benefits and limitations. Am J Roentgenol. 2009;192(5):W255-W63. doi: 10.2214/AJR.08.1488.

924. Rogal Jr SR, Neto AB, Figueredo MVM, Paraiso EC, Kaestner CAA, editors. Automatic detection of arrhythmias using wavelets and self-organized artificial neural networks. 9th International Conference on Intelligent Systems Design and Applications, ISDA 2009; 2009; Pisa.

925. Rosales M, Radeva P, Rodriguez-Leor O, Gil D. Modelling of image-catheter motion for 3-D IVUS. Med Image Anal. 2009;13(1):91-104. doi: 10.1016/j.media.2008.06.012.

926. Rosero SZ. Implantable electrocardiographic monitoring devices. Cardiol J. 2009;16(1):86-7.

927. Sahlén A, Rubulis A, Winter R, Jacobsen PH, Ståhlberg M, Tornvall P, et al. Cardiac fatigue in long-distance runners is associated with ventricular repolarization abnormalities. Heart Rhythm. 2009;6(4):512-9. doi: 10.1016/j.hrthm.2008.12.020.

928. Samanta B, Nataraj C. Morphological signal processing and computational intelligence for engineering system prognostics. Proc Inst Mech Eng Part I J Syst Control Eng. 2009;223(8):1095-109. doi: 10.1243/09596518JSCE722.

929. Sanchez LD, Pereira J, Berkoff DJ. The Evaluation of Cardiac Complaints in Marathon Runners. J Emerg Med. 2009;36(4):369-76. doi: 10.1016/j.jemermed.2007.09.029.

930. Sargolzaei A, Faez K, Sargolzaei S, editors. A new robust wavelet based algorithm for baseline wandering cancellation in ECG signals. 2009 IEEE International Conference on Signal and Image Processing Applications, ICSIPA09; 2009; Kuala Lumpur.

931. Sauvet F, Jouanin JC, Langrume C, Van Beers P, Papelier Y, Dussault C. Heart rate variability in novice pilots during and after a multi-leg cross-country flight. Aviat Space Environ Med. 2009;80(10):862-9. doi: 10.3357/ASEM.2531.2009.

932. Sayyed SH, Cassidy MM, Hadi MA. Use of multidetector computed tomography for evaluation of global and regional left ventricular function. J Cardiovasc Comput Tomogr. 2009;3(1 SUPPL.):S23-S34. doi: 10.1016/j.jcct.2008.10.016.

933. Scolaro GR, Azevedo FM, Rathke JE, Possa PRC, Andrighetto E, Adur R, et al., editors. Development of a didactic platform for acquisition and processing of biomedical signals for the practice in biomedical engineering. 9th International Conference on Electronic Measurement and Instruments, ICEMI 2009; 2009; Beijing.

934. Sriram JC, Shin M, Choudhury T, Kotz D, editors. Activity-aware ECG-based patient authentication for remote health monitoring. International Conference on Multimodal Interfaces and the Workshop on Machine Learning for Multimodal Interfaces, ICMI-MLMI'09; 2009; Cambridge, MA.

935. Stolzmann P, Leschka S, Betschart T, Desbiolles L, Flohr TG, Marincek B, et al. Radiation dose values for various coronary calcium scoring protocols in dual-source CT. Int J Card Imaging. 2009;25(4):443-51. doi: 10.1007/s10554-008-9397-y

10.1016/S0735-1097(00)00872-X; Raggi, P., Callister, T.Q., Cooil, B., He, Z.X., Lippolis, N.J., Russo, D.J., Zelinger, A., Mahmarian, J.J., Identification of patients at increased risk of first unheralded acute myocardial infarction by electron-beam computed tomography (2000) Circulation, 101, pp. 850-855; Carr, J.J., Nelson, J.C., Wong, N.D., McNitt-Gray, M., Arad, Y., Jacobs Jr., D.R., Sidney, S., Detrano, R.C., Calcified coronary artery plaque measurement with cardiac CT in population-based studies: Standardized protocol of multi-ethnic study of atherosclerosis (MESA) and coronary artery risk development in young adults (CARDIA) study (2005) Radiology, 234, pp. 35-43. , doi: 10.1148/radiol.2341040439; Shareghi, S., Ahmadi, N., Young, E., Gopal, A., Liu, S.T., Budoff, M.J., Prognostic significance of zero coronary calcium scores on cardiac computed tomography (2007) JCCT, 1, pp. 155-159; de Vos, A.M., Rutten, A., van der Zaag-Loonen, H.J., Bots, M.L., Dikkers, R., Buiskool, R.A., Mali, W.P., Oudkerk, M., Non-invasive cardiac assessment in high risk patients (the ground study): Rationale, objectives and design of a multi-center randomized controlled clinical trial (2008) Trials, 9, p. 49. , doi: 10.1186/1745-6215-9-49; Budoff, M.J., Achenbach, S., Blumenthal, R.S., Carr, J.J., Goldin, J.G., Greenland, P., Guerci, A.D., Wiegers, S.E., Assessment of coronary artery disease by cardiac computed tomography: A scientific statement from the American Heart Association Committee on Cardiovascular Imaging and Intervention, Council on Cardiovascular Radiology and Intervention, and Committee on Cardiac Imaging, Council on Clinical Cardiology (2006) Circulation, 114, pp. 1761-1791. , doi: 10.1161/CIRCULATIONAHA.106.178458; Raggi, P., Too many options for computed tomography for coronary calcium screening can we strike a balance between accuracy and radiation exposure? (2008) Acad Radiol, 15, pp. 955-957. , doi: 10.1016/j.acra.2008.06.001; Horiguchi, J., Yamamoto, H., Hirai, N., Akiyama, Y., Fujioka, C., Marukawa, K., Fukuda, H., Ito, K., Variability of repeated coronary artery calcium measurements on low-dose ECG-gated 16-MDCT (2006) AJR Am J Roentgenol, 187, pp. W1-W6. , doi: 10.2214/AJR.05.0052; McCollough, C.H., Ulzheimer, S., Halliburton, S.S., Shanneik, K., White, R.D., Kalender, W.A., Coronary artery calcium: A multi-institutional, multi-manufacturer international standard for quantification at cardiac CT (2007) Radiology, 243, pp. 527-538. , doi: 10.1148/radiol.2432050808; Muhlenbruch, G., Hohl, C., Das, M., Wildberger, J.E., Suess, C., Klotz, E., Flohr, T., Mahnken, A.H., Evaluation of automated attenuation-based tube current adaptation for coronary calcium scoring in MDCT in a cohort of 262 patients (2007) Eur Radiol, 17, pp. 1850-1857. , doi: 10.1007/s00330-006-0543-4; Abada, H.T., Larchez, C., Daoud, B., Sigal-Cinqualbre, A., Paul, J.F., MDCT of the coronary arteries: Feasibility of low-dose CT with ECG-pulsed tube current modulation to reduce radiation dose (2006) AJR Am J Roentgenol, 186, pp. S387-S390. , doi: 10.2214/AJR.05.0216; Jakobs, T.F., Wintersperger, B.J., Herzog, P., Flohr, T., Suess, C., Knez, A., Reiser, M.F., Becker, C.R., Ultra-low-dose coronary artery calcium screening using multislice CT with retrospective ECG gating (2003) Eur Radiol, 13, pp. 1923-1930. , doi: 10.1007/s00330-003-1895-7; Kopp, A.F., Ohnesorge, B., Becker, C., Schroder, S., Heuschmid, M., Kuttner, A., Kuzo, R., Claussen, C.D., Reproducibility and accuracy of coronary calcium measurements with multi-detector row versus electron-beam CT (2002) Radiology, 225, pp. 113-119. , doi: 10.1148/radiol.2251010173; Ulzheimer, S., Kalender, W.A., Assessment of calcium scoring performance in cardiac computed tomography (2003) Eur Radiol, 13, pp. 484-497; Horiguchi, J., Kiguchi, M., Fujioka, C., Arie, R., Shen, Y., Sunasaka, K., Kitagawa, T., Ito, K., Variability of repeated coronary artery calcium scoring and radiation dose on 64- and 16-slice computed tomography by prospective electrocardiographically triggered axial and retrospective electrocardiographically gated spiral computed tomography: A phantom study (2008) Acad Radiol, 15, pp. 958-965. , doi: 10.1016/j.acra.2008.03.007; Scheffel, H., Alkadhi, H., Leschka, S., Plass, A., Desbiolles, L., Guber, I., Krauss, T., Stolzmann, P., Low-dose CT coronary angiography in the step-and-shoot mode: Diagnostic performance (2008) Heart, 94 (9), pp. 1132-1137; Jakobs, T.F., Becker, C.R., Ohnesorge, B., Flohr, T., Suess, C., Schoepf, U.J., Reiser, M.F., Multislice helical CT of the heart with retrospective ECG gating: Reduction of radiation exposure by ECG-controlled tube current modulation (2002) Eur Radiol, 12, pp. 1081-1086. , doi: 10.1007/s00330-001-1278-x; Flohr, T.G., McCollough, C.H., Bruder, H., Petersilka, M., Gruber, K., Suss, C., Grasruck, M., Ohnesorge, B.M., First performance evaluation of a dual-source CT (DSCT) system (2006) Eur Radiol, 16, pp. 256-268. , doi: 10.1007/s00330-005-2919-2; Matt, D., Scheffel, H., Leschka, S., Flohr, T.G., Marincek, B., Kaufmann, P.A., Alkadhi, H., Dual-source CT coronary angiography: Image quality, mean heart rate, and heart rate variability (2007) AJR Am J Roentgenol, 189, pp. 567-573. , doi: 10.2214/AJR.07.2078; Leschka, S., Scheffel, H., Desbiolles, L., Plass, A., Gaemperli, O., Valenta, I., Husmann, L., Alkadhi, H., Image quality and reconstruction intervals of dual-source CT coronary angiography: Recommendations for ECG-pulsing windowing (2007) Invest Radiol, 42, pp. 543-549. , doi: 10.1097/RLI.0b013e31803b93cf; McCollough, C.H., Primak, A.N., Saba, O., Bruder, H., Stierstorfer, K., Raupach, R., Suess, C., Flohr, T.G., Dose performance of a 64-channel dual-source CT scanner (2007) Radiology, 243, pp. 775-784. , doi: 10.1148/radiol.2433061165; Stolzmann, P., Scheffel, H., Schertler, T., Frauenfelder, T., Leschka, S., Husmann, L., Flohr, T.G., Alkadhi, H., Radiation dose estimates in dual-source computed tomography coronary angiography (2008) Eur Radiol, 18, pp. 592-599. , doi: 10.1007/s00330-007-0786-8; Horiguchi, J., Yamamoto, H., Akiyama, Y., Marukawa, K., Hirai, N., Ito, K., Coronary artery calcium scoring using 16-MDCT and a retrospective ECG-gating reconstruction algorithm (2004) AJR Am J Roentgenol, 183, pp. 103-108; Kalender, W.A., Wolf, H., Suess, C., Gies, M., Greess, H., Bautz, W.A., Dose reduction in CT by on-line tube current control: Principles and validation on phantoms and cadavers (1999) Eur Radiol, 9, pp. 323-328. , doi: 10.1007/s003300050674; (2000) European Guidelines on Quality Criteria for Computed Tomography, , Menzel H, Schibilla H, Teunen D (eds) Luxembourg: European Commission; Publication No. EUR 16262 EN; Gerber, T.C., Kuzo, R.S., Morin, R.L., Techniques and parameters for estimating radiation exposure and dose in cardiac computed tomography (2005) Int J Cardiovasc Imaging, 21, pp. 165-176. , doi: 10.1007/s10554-004-5338-6; McNitt-Gray, M.F., AAPM/RSNA physics tutorial for residents: Topics in CT. Radiation dose in CT (2002) Radiographics, 22, pp. 1541-1553. , doi: 10.1148/rg.226025128; Hausleiter, J., Meyer, T., Hadamitzky, M., Huber, E., Zankl, M., Martinoff, S., Kastrati, A., Schomig, A., Radiation dose estimates from cardiac multislice computed tomography in daily practice: Impact of different scanning protocols on effective dose estimates (2006) Circulation, 113, pp. 1305-1310. , doi: 10.1161/CIRCULATIONAHA.105.602490; Ohnesorge, B., Flohr, T., Fischbach, R., Kopp, A.F., Knez, A., Schroder, S., Schopf, U.J., Becker, C.R., Reproducibility of coronary calcium quantification in repeat examinations with retrospectively ECG-gated multisection spiral CT (2002) Eur Radiol, 12, pp. 1532-1540. , doi: 10.1007/s00330-002-1394-2; Ropers, D., Baum, U., Pohle, K., Anders, K., Ulzheimer, S., Ohnesorge, B., Schlundt, C., Achenbach, S., Detection of coronary artery stenoses with thin-slice multi-detector row spiral computed tomography and multiplanar reconstruction (2003) Circulation, 107, pp. 664-666. , doi: 10.1161/01.CIR.0000055738.31551.A9; van Ooijen, P.M., Vliegenthart, R., Witteman, J.C., Oudkerk, M., Influence of scoring parameter settings on Agatston and volume scores for coronary calcification (2005) Eur Radiol, 15, pp. 102-110. , doi: 10.1007/s00330-004-2479-x; Van Hoe, L.R., De Meerleer, K.G., Leyman, P.P., Vanhoenacker, P.K., Coronary artery calcium scoring using ECG-gated multidetector CT: Effect of individually optimized image-reconstruction windows on image quality and measurement reproducibility (2003) AJR Am J Roentgenol, 181, pp. 1093-1100; Groen, J.M., Greuter, M.J., Schmidt, B., Suess, C., Vliegenthart, R., Oudkerk, M., The influence of heart rate, slice thickness, and calcification density on calcium scores using 64-slice multidetector computed tomography: A systematic phantom study (2007) Invest Radiol, 42, pp. 848-855; Kalra, M.K., Maher, M.M., Toth, T.L., Hamberg, L.M., Blake, M.A., Shepard, J.A., Saini, S., Strategies for CT radiation dose optimization (2004) Radiology, 230, pp. 619-628. , doi: 10.1148/radiol.2303021726; Gerber, T.C., Stratmann, B.P., Kuzo, R.S., Kantor, B., Morin, R.L., Effect of acquisition technique on radiation dose and image quality in multidetector row computed tomography coronary angiography with submillimeter collimation (2005) Invest Radiol, 40, pp. 556-563. , doi: 10.1097/01.rli.0000170628.69792.cb; Sigal-Cinqualbre, A.B., Hennequin, R., Abada, H.T., Chen, X., Paul, J.F., Low-kilovoltage multi-detector row chest CT in adults: Feasibility and effect on image quality and iodine dose (2004) Radiology, 231, pp. 169-174. , doi: 10.1148/radiol.2311030191; Thomas, C.K., Muhlenbruch, G., Wildberger, J.E., Hohl, C., Das, M., Gunther, R.W., Mahnken, A.H., Coronary artery calcium scoring with multislice computed tomography: In vitro assessment of a low tube voltage protocol (2006) Invest Radiol, 41, pp. 668-673. , doi: 10.1097/01.rli.0000233324.09603.dd; Mahnken, A.H., Wildberger, J.E., Simon, J., Koos, R., Flohr, T.G., Schaller, S., Gunther, R.W., Detection of coronary calcifications: Feasibility of dose reduction with a body weight-adapted examination protocol (2003) AJR Am J Roentgenol, 181, pp. 533-538.

936. Strachan IGD, editor Novel probabilistic algorithms for dynamic monitoring of electrocardiogram waveforms. 6th International Conference on Condition Monitoring and Machinery Failure Prevention Technologies 2009; 2009; Dublin: British Institute of Non-Destructive Testing.

937. Strachan IGD, Clifton DA, Sage C, Veselovac D, editors. A hidden Markov model for condition monitoring of a manufacturing drilling process. 6th International Conference on Condition Monitoring and Machinery Failure Prevention Technologies 2009; 2009; Dublin: British Institute of Non-Destructive Testing.

938. Strachan IGD, Hughes NP, Poonawala MH, Mason JW, Tarassenko L. Automated QT analysis that learns from cardiologist annotations. Ann Noninvasive Electrocardiol. 2009;14(SUPPL. 1):S9-S21. doi: 10.1111/j.1542-474X.2008.00259.x.

939. Summer L, Gonzalez L, Jimeno M, Christensen K. Development of a nasogastric tube insertion simulator: A collaborative interdisciplinary effort. CIN Comput Informatics Nurs. 2009;27(2):105-13. doi: 10.1097/NCN.0b013e31819753b3.

940. Sun RR, Wang YY. Predicting spontaneous termination of atrial fibrillation based on the RR interval. Proc Inst Mech Eng Part H J Eng Med. 2009;223(6):713-26. doi: 10.1243/09544119JEIM576.

941. Światowiec A, Król W, Kuch M, Braksator W, Krysztofiak H, Dłuzniewski M, et al. Analysis of 12-lead electrocardiogram in top competitive professional athletes in the light of recent guidelines. Kardiol Pol. 2009;67(10):1095-102.

942. Tan F, Polglaze T, Dawson B. Activity profiles and physical demands of elite women's water polo match play. J SPORTS SCI. 2009;27(10):1095-104. doi: 10.1080/02640410903207416.

943. Thi N, Lee GB, Wheeler P, Peterson J, editors. GA-SVM based framework for time series forecasting. 5th International Conference on Natural Computation, ICNC 2009; 2009; Tianjian.

944. Tormene P, Giorgino T, Quaglini S, Stefanelli M. Matching incomplete time series with dynamic time warping: an algorithm and an application to post-stroke rehabilitation. Artif Intell Med. 2009;45(1):11-34. doi: 10.1016/j.artmed.2008.11.007.

945. Tsai WL, Tsai IC, Chen MC, Lin PC, Chan SW, Ho HC, et al. The image quality and feasibility of delayed phase in cardiac CT: Prospective ECG-triggering sequential scan versus retrospective ECG-gating spiral scan using the same radiation dose. Chin J Radiol. 2009;34(2):67-78.

946. Turdi S, Guo R, Huff AF, Wolf EM, Culver B, Ren J. Cardiomyocyte contractile dysfunction in the APPswe/PS1dE9 mouse model of Alzheimer's disease. PLoS One. 2009;4(6):e6033. Epub 2009/06/25. doi: 10.1371/journal.pone.0006033. PubMed PMID: 19551139; PubMed Central PMCID: PMCPMC2696039.

947. Van Noord C, Straus SMJM, Sturkenboom MCJM, Hofman A, Aarnoudse AJLHJ, Bagnardi V, et al. Psychotropic drugs associated with corrected QT interval prolongation. J Clin Psychopharmacol. 2009;29(1):9-15. doi: 10.1097/JCP.0b013e318191c6a8.

948. Vassiliadis V, Dounias G. Nature-inspired intelligence: A review of selected methods and applications. Int J on Artif Intell Tools. 2009;18(4):487-516. doi: 10.1142/S021821300900024X.

949. Vierhile A, Robb A, Ryan-Krause P. Attention-Deficit/Hyperactivity Disorder in Children and Adolescents: Closing Diagnostic, Communication, and Treatment Gaps. Journal of Pediatric Health Care. 2009;23(1, Supplement):S5-S21. doi: http://doi.org/10.1016/j.pedhc.2008.10.009.

950. Wang CH, Dong TP, Kuo W. A hybrid approach for identification of concurrent control chart patterns. J Intell Manuf. 2009;20(4):409-19. doi: 10.1007/s10845-008-0115-3.

951. Willick S, Akau CK, Harrast MA, Storm SA, Finnoff JT. Sports and Performing Arts Medicine: 5. Special Populations. PM&R. 2009;1(3, Supplement):S78-S82. doi: http://doi.org/10.1016/j.pmrj.2009.01.023.

952. Xie SY, Guo R, Li NF, Wang G, Zhao HT, editors. Brain fMRI processing and classification based on combination of PCA and SVM. 2009 International Joint Conference on Neural Networks, IJCNN 2009; 2009; Atlanta, GA.

953. Yu SN, Chou KT. Selection of significant independent components for ECG beat classification. Expert Sys Appl. 2009;36(2 PART 1):2088-96. doi: 10.1016/j.eswa.2007.12.016.

954. Zhang Q, Eagleson R, Peters TM. Dynamic real-time 4D cardiac MDCT image display using GPU-accelerated volume rendering. Comput Med Imaging Graph. 2009;33(6):461-76. doi: 10.1016/j.compmedimag.2009.04.002.

955. Zheng X, Li X, Liu J, Chen W, Hao Y, editors. A portable wireless eye movement-controlled Human-Computer Interface for the Disabled. 2009 ICME International Conference on Complex Medical Engineering, CME 2009; 2009; Tempe, AZ.

956. Canadian Cardiovascular Society (CCS) CCS825 Oral: Treatment and Outcomes in Acute Coronary Syndromes (ACS) Tuesday, October 26, 2010. Canadian Journal of Cardiology. 2010;26, Supplement D:110D-1D. doi: http://doi.org/10.1016/S0828-282X(10)71139-9.

957. Discover new research, new colleagues and old Montréal at the 2010 Canadian Cardiovascular Congress! Canadian Journal of Cardiology. 2010;26(7):347-8. doi: http://doi.org/10.1016/S0828-282X(10)70432-3.

958. Subject Index. Journal of Emergency Nursing. 2010;36(6):e8-e20. doi: http://doi.org/10.1016/S0099-1767(10)00524-6.

959. News. The Journal for Nurse Practitioners. 2010;6(10):747-50. doi: http://doi.org/10.1016/j.nurpra.2010.09.011.

960. Author Index. Journal of Emergency Nursing. 2010;36(6):e3-e7. doi: http://doi.org/10.1016/S0099-1767(10)00523-4.

961. Sixième Congrès de Pneumologie et de Chirurgie Thoracique de l'Association Franco-Vietnamienne de Pneumologie, 10 au 12 novembre, Dalat, Vietnam. Revue des Maladies Respiratoires. 2010;27(8):971-81. doi: http://doi.org/10.1016/j.rmr.2010.09.006.

962. Sessions Posters. Réanimation. 2010;19, Supplement 1:S36-S207. doi: http://doi.org/10.1016/j.reaurg.2009.11.001.

963. Electrical stimulation. Physiother Can. 2010;62(5):26-38. doi: 10.3138/ptc.2009-09-s4

10.1016/S0140-6736 78 91312-0; Glotzer, T.V., Gordon, M., Sparta, M., Radoslovich, G., Zimmerman, J., Electromagnetic interference from a muscle stimulation device causing discharge of an implantable cardioverter defibrillator: Epicardial bipolar and endocardial bipolar sensing circuits are compared (1998) PACE - Pacing and Clinical Electrophysiology, 21 (10), pp. 1996-1998. , DOI 10.1111/j.1540-8159.1998.tb00021.x; Glotzer, T.V., Reply to the editor (1999) Pacing Clin. Electrophysiol, 22, p. 693. , doi:10.1111/j.1540-8159.1999.tb00522.x; Jones, S.L., Electromagnetic field interference and cardiac pacemakers (1976) Phys. Ther., 56, pp. 1013-1018; La Ban, M., Petty, D., Hauser, A., Taylor, R., Peripheral nerve conduction stimulation: Its effect on cardiac pacemakers (1988) Arch. Phys. Med. Rehabil., 69, pp. 358-362; Philbin, D.M., Schabrun, S., Inappropriate shocks delivered by an ICD as a result of sensed potentials from a trancutaneous electrical nerve stimulation unit (1998) Pacing Clin. Electrophysiol, 10, pp. 2010-2011; Nagele, H., Azizi, M., Inappropriate ICD discharge induced by electrical interference from a physio-therapeutic muscle stimulation device (2006) Herzschrittmachertherapie und Elektrophysiologie, 17 (3), pp. 137-139. , DOI 10.1007/s00399-006-0527-8; Weitz, S.H., Tunick, P.A., McElhinney, L., Mitchell, T., Kronzon, I., Pseudoatrial flutter: Artifact simulating atrial flutter caused by a transcutaneous electrical nerve stimulator (TENS) (1997) PACE - Pacing and Clinical Electrophysiology, 20 (12), pp. 3010-3011. , DOI 10.1111/j.1540-8159.1997.tb05478.x; Hauptman, P.J., Raza, M., Electrocardiographic artifact with a transcutaneous electrical nerve stimulation unit (1992) Int. J. Cardiol, 34, pp. 110-112. , doi:10.1016/0167-5273 92 90093-I; Kimberley, A.P., Soni, N., Williams, T.R., Transcutaneous nerve stimulation and the electrocardiograph (1987) Anaesth. Intens Care, 15, pp. 358-359; Marples, I.L., Transcutaneous electrical nerve stimulation (TENS): An unusual source of electrocardiogram artifact (2000) Anaesth., 55, pp. 719-720. , doi:10.1046/j.1365-2044.2000.01557-39x; Sliwa, J.A., Marinko, M.S., Transcutaneous electrical nerve stimulation-induced electrocardiogram artifact: A brief report (1996) Am. J. Phys. Med. Rehabil., 75, pp. 307-309; Rasmussen, M.J., Hayes, D.L., Vlietstra, R.E., Thorsteinsson, G., Can transcutaneous electrical nerve stimulation be safely used in patients with permanent cardiac pacemakers? (1988) Mayo Clin. Proc., 63, pp. 443-445; Dunn, P., Rogers, D., Halford, K., Transcutaneous electrical nerve stimulation at acupuncture points in the induction of uterine contractions (1989) Obstet. Gynecol, 73, pp. 286-290; Belanger, A.Y., Physiological evidence for an endogenous opiaterelated pain-modulating system and its relevance to TENS: A review (1985) Physiother. Can., 37, pp. 163-168; Chapman, C.R., Benedetti, C., Analgesia following transcutaneous electrical stimulation and its partial reversal by a narcotic antagonist (1977) Life Sci., 21, pp. 1645-1648. , doi:10.1016/0024-3205 77 90243-0; Gabis, L., Shklar, B., Geva, D., Immediate influence of transcranial electrostimulation of pain and ß-endorphin blood levels: An active placebo-controlled study (2003) Am. J. Phys. Med. Rehabil., 82, pp. 81-85; Coldron, Y., Crothers, E., Haslam, J., Notcutt, W., Sidney, D., Thomas, R., (2007) ACPWH Guidance on the Safe use of Transcutaneous Electrical Nerve Stimulation (TENS) for Musculosketal Pain During Pregnancy, , London, UK: Edited/written by the ACPWH and published by the Chartered Society of Physiotherapists; Schaefer, N., Schafer, H., Maintz, D., Wagner, M., Overhaus, M., Hoelscher, A.H., Turler, A., Efficacy of Direct Electrical Current Therapy and Laser-Induced Interstitial Thermotherapy in Local Treatment of Hepatic Colorectal Metastases: An Experimental Model in the Rat (2008) Journal of Surgical Research, 146 (2), pp. 230-240. , DOI 10.1016/j.jss.2007.03.084, PII S0022480407002454; Bauer, W., Electrical treatment of severe head and neck cancer pain (1983) Arch. Otolaryngol, 109, pp. 382-383; Bausewein, C., Booth, S., Gysels, M., Higginson, I., Non-pharmacological interventions for breathlessness in advanced stages of malignant and non-malignant diseases (2008) Cochrane Db Syst. Rev., p. 2. , doi:10.1002/14651858. CD005623.pub2; Crevenna, R., Marosi, C., Schmidinger, M., Fialka-Moser, V., Neuromuscular electrical stimulation for a patient with metastatic lung cancer-a case report (2006) Support Care Cancer, 14, pp. 970-973. , doi:10.1007/s00520-006-0033-x; Reuss, R., Meyer, S.C., The use of TENS in the management of cancer pain (1985) Clin. Manag. Phys. Ther., 5, pp. 26-28; Kaada, B., Vasodilatation induced by transcutaneous nerve stimulation in peripheral ischemia (Raynaud's phenomenon and diabetic polyneuropathy) (1982) Eur. Heart J., 3, pp. 303-314; Lamb, S., Mani, R., Does interferential therapy affect blood flow? (1994) Clin. Rehabil., 8, pp. 213-218. , doi:10.1177/026921559400800305; Nussbaum, E.L., Rush, P., Disenhaus, L., The effects of interferential therapy on peripheral blood flow (1990) Physiotherapy, 76, pp. 803-807. , doi:10.1016/S0031-9406 10 63186-0; Levine, S.P., Kett, R.L., Gross, M.D., Wilson, B.A., Cederna, P.S., Juni, J.E., Blood flow in the gluteus maximus of seated individuals during electrical muscle stimulation (1990) Arch. Phys. Med. Rehabil., 71, pp. 682-686; Liu, H.I., Currier, D.P., Threlkeld, A.J., Circulatory response of digital arteries associated with electrical stimulation of calf muscle in healthy subjects (1987) Phys. Ther., 67, pp. 340-345; Mohr, T., Akers, T.K., Wessman, H.C., Effect of high voltage stimulation on blood flow in the rat hind limb (1987) Physical Therapy, 67 (4), pp. 526-533; Lindstrom, B., Korsan-Bengtsen, K., Jonsson, O., Petrusson, B., Pettersson, S., Wikstrand, J., Electrically induced short-lasting tetanus of the calf muscles for prevention of deep vein thrombus (1982) Brit J. Surg., 69, pp. 203-206; Aquejouf, O., Doutremepuich, F., Doutremepuich, C., Effects of electrical stimulation on laser beam induced experimental thrombus (2006) Pathophysiol Haemost. Thromb., 35, pp. 364-369; Querol, F., Gallach, J.E., Toca-Herrera, J.L., Gomis, M., Gonzalez, L.-M., Surface electrical stimulation of the quadriceps femoris in patients affected by haemophilia A (2006) Haemophilia, 12 (6), pp. 629-632. , DOI 10.1111/j.1365-2516.2006.01356.x; Roche, P.A., Gijsbers, K., Belch, J.J., Forbes, C.D., Modification of haemophiliac haemorrhage pain by transcutaneous electrical nerve stimulation (1985) Pain, 21, pp. 43-48. , doi:10.1016/0304-3959 85 90075-2; Kincaid, C.B., Lavoie, K.H., Inhibition of bacterial growth in vitro following stimulation with high voltage, monophasic, pulsed current (1989) Phys. Ther., 69, pp. 651-655; Rowley, B.A., Electrical current effects on E. coli growth rates (1972) Proc. Soc. Exp. Biol. Med., 139, pp. 929-934; Szuminsky, N., Albers, A.C., Unger, P., Eddy, J.G., Effect of narrow, pulsed high voltages on bacterial viability (1994) Phys. Ther., 74, pp. 660-667; Gilcreast, D., Stotts, N.A., Froelicher, E., Baker, L., Moss, K., Effect of electrical stimulation on foot skin perfusion in persons with or at risk for diabetic foot ulcers (1998) Wound Repair Regen, 6, pp. 434-441. , doi:10.1046/j.1524-475X.1998.60505.x; Anderson, S.I., Whatling, P., Hudlicka, O., Gosling, P., Simms, M., Brown, M.D., Chronic transcutaneous electrical stimulation of calf muscles improves functional capacity without inducing systemic inflammation in claudicants (2004) European Journal of Vascular and Endovascular Surgery, 27 (2), pp. 201-209. , DOI 10.1016/j.ejvs.2003.10.003; Im, M.J., Lee, W.P.A., Hoopes, J.E., Effect of electrical stimulation on survival of skin flaps in pigs (1990) Phys. Ther., 70, pp. 37-40; Peters, E.J.G., Armstrong, D.G., Wunderlich, R.P., Bosma, J., Stacpoole-Shea, S., Lavery, L.A., The benefit of electrical stimulation to enhance perfusion in persons with diabetes mellitus (1998) Journal of Foot and Ankle Surgery, 37 (5), pp. 396-400; Goldman, R., Brewley, B., Zhou, L., Golden, M., Electrotherapy reverses inframalleolar ischemia: A retrospective, observational study (2003) Adv. Skin Wound Care, 16, pp. 79-89. , doi:10.1097/00129334-200303000-00009; Lambert, I., Tebbs, S.E., Hill, D., Moss, H.A., Davies, A.J., Elliot, T.S., Interferential therapy machines as a possible vehicle for crossinfection (2000) J. Hosp. Infect., 44, pp. 59-64. , doi:10.1053/jhin. 1999.0647; Al'Ajlan, A., Thestrup-Pedersen, K., Al'Eisa, A., Contact leukoderma following nickel dermatitis elicited by TENS electrode plates (2000) Contact Dermatitis, 42, pp. 172-173; Dwyer, C.M., Chapman, R.S., Forsyth, A., Allergic contact dermatitis from TENS gel (1994) Contact Dermatitis, 30 (5), p. 305. , DOI 10.1111/j.1600-0536.1994.tb00608.x; Oyibo, S.O., Breislin, K., Boulton, A.J.M., Electrical stimulation therapy through stocking electrodes for painful diabetic neuropathy: A double blind, controlled crossover study (2004) Diabetic Medicine, 21 (8), pp. 940-944. , DOI 10.1111/j.1464-5491.2004.01243.x; Stecker, M.M., Patterson, T., Netherton, B.L., Mechanisms of electrode induced injury, part 1: Theory (2006) Am. J. Electroneurodiagnostic Technol., 46, pp. 315-342; Weber-Muller, F., Reichert-Penetrat, S., Schmutz, J.L., Barbaud, A., Contact dermatitis from polyacrylate in TENS electrode (2004) Ann. Dermatol. Vener, 131, pp. 478-480; Ford, K.S., Shrader, M.W., Smith, J., McLean, T.J., Dahm, D.L., Full thickness burn formation after the use of electrical stimulation therapy for rehabilitation of unicompartmental knee arthroplasty (2005) J. Arthroplasty, 20, pp. 950-953; Tsang, K.K.W., Morris, L.M., Hand, J.W., Ice bag application may negate the effects of interferential electrical stimulation (2008) J. Athl Training, 43 (3 SUPP.), pp. S84; Dudley-Javoroski, S., Shields, R.K., Muscle and bone plasticity after spinal cord injury: Review of adaptations to disuse and to electrical muscle stimulation (2008) J. Rehabil. Res. Dev., 45, pp. 283-296. , doi:10.1682/JRRD.2007.02.0031; Richardson, R.R., Meyer Jr., P.R., Cerullo, L.J., Transcutaneous electrical neurostimulation in musculoskeletal pain of acute spinal cord injuries (1980) Spine, 5, pp. 42-45. , doi:10.1097/00007632-198001000-00008; Loeser, J.D., Black, R.G., Christman, A., Relief of pain by transcutaneous stimulation (1975) J. Neurosurg., 42, pp. 308-314. , doi:10.3171/jns.1975.42.3.0308; Stevens, J.E., Mizner, R.L., Snyder-Mackler, L., Quadriceps strength and volitional activation before and after total knee arthroplasty for osteoarthritis (2003) Journal of Orthopaedic Research, 21 (5), pp. 775-779. , DOI 10.1016/S0736-0266(03)00052-4; Stevens, J.E., Mizner, R.L., Snyder-Mackler, L., Neuromuscular Electrical Stimulation for Quadriceps Muscle Strengthening after Bilateral Total Knee Arthroplasty: A Case Series (2004) Journal of Orthopaedic and Sports Physical Therapy, 34 (1), pp. 21-29. , DOI 10.2519/jospt.2004.0947; Avramidis, K., Strike, P.W., Taylor, P.N., Swain, I.D., Effectiveness of electrical stimulation of the vastus medialis muscle in the rehabilitation of patients after total knee arthroplasty (2003) Arch. Phys. Med. Rehabil., 84, pp. 1850-1853. , doi:10.1016/S0003-9993 03 00429-5; Ansari, A., Ramsey, K.W.D.R., Floyd, D.C., Rupture of a flexor pollicis longus repair in a body builder through the use of an electronic muscle stimulator (2006) British Journal of Sports Medicine, 40 (12), pp. 1009-1010. , DOI 10.1136/bjsm.2006.026591; Rosted, P., Repetitive epileptic fits-a possible adverse effect after TENS (2001) Acupuncture Med., 19, pp. 46-49. , doi:10.1136/aim.19.1.46; Scherder, E., Someren, E.J.V., Swaab, D., Epilepsy: A possible contraindication for transcutaneous electrical nerve stimulation (1999) J. Pain Symptom Manage, 17, pp. 152-153; Guo, J., Lui, J., Fu, W., Ma, W., Xu, Z., Yuan, M., Effect of electroacupuncture stimulation of hindlimb on seizure incidence and supragranular mossy fibre sprouting in a rat model of epilepsy (2008) J. Physiol. Sci., 58, pp. 309-315; Mann, J.C., Respiratory compromise: A rare complication of transcutaneous electrical nerve stimulation for angina pectoris (1996) J. Accid Emerg. Med., 13, p. 68. , doi:10.1136/emj.13.1.68; Benedetti, F., Amanzio, M., Casadio, C., Cavallo, A., Cianci, R., Giobbe, R., Postthoracoscopy pain: Is TENS the answer? (1997) Ann. Thorac. Surg., 64, pp. 608-610; Bourjeily-Habr, G., Rochester, C.L., Palermo, F., Snyder, P., Mohsenin, V., Randomised controlled trial of transcutaneous electrical muscle stimulation of the lower extremities in patients with chronic obstructive pulmonary disease (2002) Thorax, 57 (12), pp. 1045-1049. , DOI 10.1136/thorax.57.12.1045; Dal Corso, S., Napolis, L., Malaguti, C., Gimenes, A.C., Albuquerque, A., Nogueira, C.R., De Fuccio, M.B., Neder, J.A., Skeletal muscle structure and function in response to electrical stimulation in moderately impaired COPD patients (2007) Respiratory Medicine, 101 (6), pp. 1236-1243. , DOI 10.1016/j.rmed.2006.10.023, PII S0954611106005439; Neder, J.A., Sword, D., Ward, S.A., Mackay, E., Cochrane, L.M., Clark, C.J., Home based neuromuscular electrical stimulation as a new rehabilitative strategy for severely disabled patients with chronic obstructive pulmonary disease (COPD) (2002) Thorax, 57 (4), pp. 333-337. , DOI 10.1136/thorax.57.4.333; Quittan, M., Wiesinger, G.F., Sturm, B., Puig, S., Mayr, W., Sochor, A., Paternostro, T., Fialka-Moser, V., Improvement of thigh muscles by neuromuscular electrical stimulation in patients with refractory heart failure: A single-blind, randomized, controlled trial (2001) American Journal of Physical Medicine and Rehabilitation, 80 (3), pp. 206-214. , DOI 10.1097/00002060-200103000-00011; Vivodtzev, I., Lacasse, Y., Maltais, F., Neuromuscular electrical stimulation of the lower limbs in patients with chronic obstructive pulmonary disease (2008) J. Cardiopulm Rehabil., 28, pp. 79-91; Zanotti, E., Felicetti, G., Maini, M., Fracchia, C., Peripheral muscle strength training in bed-bound patients with COPD receiving mechanical ventilation: Effect of electrical stimulation (2003) Chest, 124 (1), pp. 292-296. , DOI 10.1378/chest.124.1.292; Solomon, S., Elkind, A., Freitag, F., Gallagher, R.M., Moore, K., Swerdlow, B., Safety and effectiveness of cranial electrotherapy in the treatment of tension headache (1989) Headache, 29, pp. 445-450. , doi:10.1111/j.1526-4610.1989.hed2907445.x; Engin-Ustun, Y., Korkmaz, C., Duru, N., Baser, I., Comparison of three sperm retrieval techniques in spinal cord-injured men: Pregnancy outcome (2006) Gynecological Endocrinology, 22 (5), pp. 252-255. , DOI 10.1080/09513590600647326, PII V402203500281717; Mariotti, G., Sciarra, A., Salciccia, S., Alfarone, A., Pierro, G.D., Gentile, V., Early recovery of urinary incontinence after prostatectomy using early pelvic floor electrical stimulation and biofeedback associated treatment (2009) J. Urol, 181, pp. 1788-1789; Gilling, P.J., Wilson, L.C., Westenberg, A.M., McAllister, W.J., Kennett, K.M., Frampton, C.M., A double blind randomized controlled trial of electromagnetic stimulation of the pelvic floor versus sham therapy in the treatment of women with stress urinary incontinence (2009) Brit J. Urol, 103, pp. 1386-1390; Tsujimoto, T., Takano, M., Ishikawa, M., Tsuruzono, T., Matsumura, Y., Kitano, H., Yoneda, S., Fukui, H., Onset of ischemic colitis following use of electrical muscle stimulation (EMS) exercise equipment (2004) Internal Medicine, 43 (8), pp. 693-695. , DOI 10.2169/internalmedicine.43.693; Bolton, L., TENS electrode irritation (1983) J. Am. Acad. Dermatol., 8, pp. 134-135. , doi:10.1016/S0190-9622 83 80304-1; Castelain, P.Y., Chabeau, G., Contact dermatitis after transcutaneous electric analgesia (1986) Contact Dermatitis, 15, pp. 32-35. , doi:10.1111/j.1600-0536.1986.tb01258.x; Fisher, A.A., Dermatitis associated with transcutaneous electrical stimulation current (1978) Cutis, 21, pp. 24-47; Fisher, A.A., Brancaccio, R.R., Allergic contact sensitivity to propylene glycol in a lubricant jelly (1979) Archives of Dermatology, 115 (12), p. 1451. , DOI 10.1001/archderm.115.12.1451; Marren, P., De Berker, D., Powell, S., Methacrylate sensitivity and transcutaneous electrical nerve stimulation (TENS) (1991) Contact Dermatitis, 25, pp. 190-191. , doi:10.1111/j.1600-0536.1991.tb01828.x; Morris, S.D., McGibbon, D.H., Rycroft, R.J.G., Letters to the editor: Dermatitis caused by electromagnetic radiation [1] (2001) Contact Dermatitis, 45 (3), p. 188. , DOI 10.1034/j.1600-0536.2001.045003188.x; Zugerman, C., Dermatitis from transcutaneous electrical nerve stimulation (1982) J. Am. Acad. Dermatol., 6, pp. 936-939. , doi:10.1016/S0190-9622 82 70082-9; Frasson, E., Priori, A., Ruzzante, B., Didone, G., Bertolasi, L., Nerve stimulation boosts botulinum toxin action in spasticity (2005) Movement Disorders, 20 (5), pp. 624-629. , DOI 10.1002/mds.20395; Marchand, S., Li, J., Charest, J., Effects of caffeine on analgesia from transcutaneous electric nerve stimulation (1995) N Engl. J. Med., 333, pp. 325-326. , doi:10.1056/NEJM199508033330521; Sluka, K.A., Walsh, D., Transcutaneous electrical nerve stimulation: Basic science mechanisms and clinical effectiveness (2003) Journal of Pain, 4 (3), pp. 109-121. , DOI 10.1054/jpai.2003.434; Kitchen, S., Audit of the unexpected effects of electrophysical agents (2000) Physiotherapy, 86, pp. 152-155; Partridge, C.J., Kitchen, S.S., Adverse effects of electrotherapy used by physiotherapists (1999) Physiotherapy, 85, pp. 298-303. , doi:10.1016/S0031-9406 05 67133-7; Guarascio, P., Lusi, E.A., Soccorsi, F., Electronic muscle stimulators: A novel unsuspected cause of rhabdomyolysis (2004) Brit J. Sport Med., 38, pp. 505-507; Kalinowski, D., Brogan, M., Sleeper, M., A practical technique for disinfecting electrical stimulation apparatuses used in wound treatment (1996) Phys. Ther., 76, pp. 1340-1347; Shields, N., O'Hare, N., Boyle, G., Gormley, J., Development and application of a quality control procedure for short-wave diathermy units (2003) Medical and Biological Engineering and Computing, 41 (1), pp. 62-68. , DOI 10.1007/BF02343540.

964. Afsar FA, editor Prediction of acute hypotension episodes in patients taking pressor medication using modeling of arterial blood pressure waveforms. 4th International Conference on Bioinformatics and Biomedical Engineering, iCBBE 2010; 2010; Chengdu.

965. Al-Naima F, Ali Al-Timemy AH, editors. A neural network based algorithm for assessing risk priority of medical equipments. 2010 7th International Multi-Conference on Systems, Signals and Devices, SSD-10; 2010; Amman.

966. Ammer K. Thermology 2009 - A computer- assisted literature survey. Thermology Int. 2010;20(1):5-27.

967. Andreatta PB, Maslowski E, Petty S, Shim W, Marsh M, Hall T, et al. Virtual reality triage training provides a viable solution for disaster-preparedness. Acad Emerg Med. 2010;17(8):870-6. doi: 10.1111/j.1553-2712.2010.00728.x.

968. Arnrich B, Mayora O, Bardram J, Tröster G. Pervasive healthcare paving the way for a pervasive, user-centered and preventive healthcare model. METHODS INF MED. 2010;49(1):67-73. doi: 10.3414/ME09-02-0044.

969. Artikis A, Paliouras G, Portet F, Skarlatidis A, editors. Logic-based representation, reasoning and machine learning for event recognition. 4th ACM International Conference on Distributed Event-Based Systems, DEBS 2010; 2010; Cambridge.

970. Balady GJ, Arena R, Sietsema K, Myers J, Coke L, Fletcher GF, et al. Clinician's guide to cardiopulmonary exercise testing in adults: A scientific statement from the American heart association. Circulation. 2010;122(2):191-225. doi: 10.1161/CIR.0b013e3181e52e69

10.1016/j.ijcard.2008. 12.143 Accessed May 27; Guazzi, M., Myers, J., Arena, R., Cardiopulmonary exercise testing in the clinical and prognostic assessment of diastolic heart failure (2005) Journal of the American College of Cardiology, 46 (10), pp. 1883-1890. , DOI 10.1016/j.jacc.2005.07.051, PII S0735109705019984; Moore, B., Brubaker, P.H., Stewart, K.P., Kitzman, D.W., VE/VCO2 slope in older heart failure patients with normal versus reduced ejection fraction compared with age-matched healthy controls (2007) J Card Fail, 13, pp. 259-262; Arena, R., Owens, D.S., Arevalo, J., Smith, K., Mohiddin, S.A., McAreavey, D., Ulisney, K.L., Plehn, J.F., Ventilatory efficiency and resting hemodynamics in hypertrophic cardiomyopathy (2008) Med Sci Sports Exerc, 40, pp. 799-805; Guazzi, M., Myers, J., Peberdy, M.A., Bensimhon, D., Chase, P., Arena, R., Exercise oscillatory breathing in diastolic heart failure: Prevalence and prognostic insights (2008) Eur Heart J, 29, pp. 2751-2759; Waraich, S., Sietsema, K.E., Clinical cardiopulmonary exercise testing: Patient and referral characteristics (2007) J Cardiopulm Rehabil Prev, 27, pp. 400-406; Janicki, J.S., Weber, K.T., Likoff, M.J., Fishman, A.P., Exercise testing to evaluate patients with pulmonary vascular disease (1984) Am Rev Respir Dis, 129, pp. S93-S95; Martinez, F.J., Stanopoulos, I., Acero, R., Becker, F.S., Pickering, R., Beamis, J.F., Graded comprehensive cardiopulmonary exercise testing in the evaluation of dyspnea unexplained by routine evaluation (1994) Chest, 105, pp. 168-174; Depaso, W.J., Winterbauer, R.H., Lusk, J.A., Dreis, D.F., Springmeyer, S.C., Chronic dyspnea unexplained by history, physical examination, chest roentgenogram, and spirometry: Analysis of a seven-year experience (1991) Chest, 100, pp. 1293-1299; Pratter, M.R., Curley, F.J., Dubois, J., Irwin, R.S., Cause and evaluation of chronic dyspnea in a pulmonary disease clinic (1989) Arch Intern Med, 149, pp. 2277-2282; Wasserman, K., Hansen, J.E., Sue, D.Y., Stringer, W.W., Whipp, B.J., (2004) Principles of Exercise Testing and Interpretation: Including Pathophysiology and Clinical Applications, , 4th ed. Philadelphia Pa: Lippincott Williams & Wilkins; Weisman, I.M., Zeballos, R.J., An integrated approach to the interpretation of cardiopulmonary exercise testing (1994) Clin Chest Med, 15, pp. 421-445; Palange, P., Carlone, S., Forte, S., Galassetti, P., Serra, P., Cardiopulmonary exercise testing in the evaluation of patients with ventilatory vs circulatory causes of reduced exercise tolerance (1994) Chest, 105, pp. 1122-1126; Palange, P., Ward, S.A., Carlsen, K.-H., Casaburi, R., Gallagher, C.G., Gosselink, R., O'Donnell, D.E., Whipp, B.J., Recommendations on the use of exercise testing in clinical practice (2007) European Respiratory Journal, 29 (1), pp. 185-209. , DOI 10.1183/09031936.00046906; Hansen, J.E., Sue, D.Y., Oren, A., Wasserman, K., Relation of oxygen uptake to work rate in normal men and men with circulatory disorders (1987) Am J Cardiol, 59, pp. 669-674; Haller, R.G., Lewis, S.F., Pathophysiology of exercise performance in muscle disease (1984) Med Sci Sports Exerc, 16, pp. 456-459; Flaherty, K.R., Wald, J., Weisman, I.M., Zeballos, R.J., Schork, M.A., Blaivas, M., Rubenfire, M., Martinez, F.J., Unexplained exertional limitation: Characterization of patients with a mitochondrial myopathy (2001) Am J Respir Crit Care Med, 164, pp. 425-432; Tanabe, Y., Nakagawa, I., Ito, E., Suzuki, K., Hemodynamic basis of the reduced oxygen uptake relative to work rate during incremental exercise in patients with chronic heart failure (2002) Int J Cardiol, 83, pp. 57-62; Duscha, B.D., Kraus, W.E., Keteyian, S.J., Sullivan, M.J., Green, H.J., Schachat, F.H., Pippen, A.M., Annex, B.H., Capillary density of skeletal muscle: A contributing mechanism for exercise intolerance in class II-III chronic heart failure independent of other peripheral alterations (1999) J Am Coll Cardiol, 33, pp. 1956-1963; Hambrecht, R., Fiehn, E., Yu, J., Niebauer, J., Weigl, C., Hilbrich, L., Adams, V., Schuler, G., Effects of endurance training on mitochondrial ultra-structure and fiber type distribution in skeletal muscle of patients with stable chronic heart failure (1997) J Am Coll Cardiol, 29, pp. 1067-1073; Mettauer, B., Zoll, J., Garnier, A., Ventura-Clapier, R., Heart failure: A model of cardiac and skeletal muscle energetic failure (2006) Pflugers Arch, 452, pp. 653-666; Sullivan, M.J., Knight, J.D., Higginbotham, M.B., Cobb, F.R., Relation between central and peripheral hemodynamics during exercise in patients with chronic heart failure: Muscle blood flow is reduced with maintenance of arterial perfusion pressure (1989) Circulation, 80, pp. 769-781; Papazachou, O., Anastasiou-Nana, M., Sakellariou, D., Tassiou, A., Dimopoulos, S., Venetsanakos, J., Maroulidis, G., Nanas, S., Pulmonary function at peak exercise in patients with chronic heart failure (2007) International Journal of Cardiology, 118 (1), pp. 28-35. , DOI 10.1016/j.ijcard.2006.04.091, PII S0167527306006103; Agostoni, P., Bussotti, M., Cattadori, G., Margutti, E., Contini, M., Muratori, M., Marenzi, G., Fiorentini, C., Gas diffusion and alveolar-capillary unit in chronic heart failure (2006) Eur Heart J, 27, pp. 2538-2543; Marin-García, J., Goldenthal, M.J., Moe, G.W., Abnormal cardiac and skeletal muscle mitochondrial function in pacing-induced cardiac failure (2001) Cardiovasc Res, 52, pp. 103-110; Duscha, B.D., Schulze, P.C., Robbins, J.L., Forman, D.E., Implications of chronic heart failure on peripheral vasculature and skeletal muscle before and after exercise training (2008) Heart Fail Rev, 13, pp. 21-37; Gielen, S., Adams, V., Möbius-Winkler, S., Linke, A., Erbs, S., Yu, J., Kempf, W., Hambrecht, R., Anti-inflammatory effects of exercise training in the skeletal muscle of patients with chronic heart failure (2003) J Am Coll Cardiol, 42, pp. 861-868; Siciliano, G., Volpi, L., Piazza, S., Ricci, G., Mancuso, M., Murri, L., Functional diagnostics in mitochondrial diseases (2007) Biosci Rep, 27, pp. 53-67; Jeppesen, T.D., Schwartz, M., Olsen, D.B., Vissing, J., Oxidative capacity correlates with muscle mutation load in mitochondrial myopathy (2003) Ann Neurol, 54, pp. 86-92; Taivassalo, T., Jensen, T.D., Kennaway, N., Dimauro, S., Vissing, J., Haller, R.G., The spectrum of exercise tolerance in mitochondrial myopathies: A study of 40 patients (2003) Brain, 126, pp. 413-423; Taivassalo, T., Haller, R.G., Exercise and training in mitochondrial myop-athies (2005) Med Sci Sports Exerc, 37, pp. 2094-2101; Arena, R., Myers, J., Guazzi, M., The clinical significance of aerobic exercise testing and prescription: From apparently healthy to confirmed cardiovascular disease (2008) Am J Lifestyle Med, 2, pp. 519-536; MacKo, R.F., Ivey, F.M., Forrester, L.W., Task-oriented aerobic exercise in chronic hemiparetic stroke: Training protocols and treatment effects (2005) Top Stroke Rehabil, 12, pp. 45-57; MacKo, R.F., Benvenuti, F., Stanhope, S., MacEllari, V., Taviani, A., Nesi, B., Weinrich, M., Stuart, M., Adaptive physical activity improves mobility function and quality of life in chronic hemiparesis (2008) J Rehabil Res Dev, 45, pp. 323-328; Ivey, F.M., MacKo, R.F., Prevention of deconditioning after stroke (2009) Stroke Recovery and Rehabilitation Textbook, pp. 387-404. , Stein J, Harvey RL, Macko RF, Winstein CJ, Zorowitz RD, eds New York, NY: Demos Medical; Fletcher, B.J., Dunbar, S.B., Felner, J.M., Jensen, B.E., Almon, L., Cotsonis, G., Fletcher, G.F., Exercise testing and training in physically disabled men with clinical evidence of coronary artery disease (1994) Am J Cardiol, 73, pp. 170-174; Luft, A.R., MacKo, R.F., Forrester, L.W., Villagra, F., Ivey, F., Sorkin, J.D., Whitall, J., Hanley, D.F., Treadmill exercise activates subcortical neural networks and improves walking after stroke: A randomized controlled trial (2008) Stroke, 39, pp. 3341-3350; Ivey, F.M., Ryan, A.S., Hafer-Macko, C.E., Goldberg, A.P., MacKo, R.F., Treadmill aerobic training improves glucose tolerance and indices of insulin sensitivity in disabled stroke survivors: A preliminary report (2007) Stroke, 38, pp. 2752-2758; The pulmonary system (2007) Guides to the Evaluation of Permanent Impairment, , American Medical Association. 6th ed. Chicago Ill: American Medical Association; Oren, A., Sue, D.Y., Hansen, J.E., Torrance, D.J., Wasserman, K., The role of exercise testing in impairment evaluation (1987) Am Rev Respir Dis, 135, pp. 230-235; Agostoni, P., Smith, D.D., Schoene, R.B., Robertson, H.T., Butler, J., Evaluation of breathlessness in asbestos workers: Results of exercise testing (1987) Am Rev Respir Dis, 135, pp. 812-816; Fredriksen, P.M., Veldtman, G., Hechter, S., Therrien, J., Chen, A., Warsi, M.A., Freeman, M., Webb, G., Aerobic capacity in adults with various congenital heart diseases (2001) Am J Cardiol, 87, pp. 310-314; Dimopoulos, K., Okonko, D.O., Diller, G.-P., Broberg, C.S., Salukhe, T.V., Babu-Narayan, S.V., Li, W., Gatzoulis, M.A., Abnormal ventilatory response to exercise in adults with congenital heart disease relates to cyanosis and predicts survival (2006) Circulation, 113 (24), pp. 2796-2802. , DOI 10.1161/CIRCULATIONAHA.105.594218, PII 0000301720060620000006; Diller, G.P., Dimopoulos, K., Okonko, D., Li, W., Babu-Narayan, S.V., Broberg, C.S., Johansson, B., Gatzoulis, M.A., Exercise intolerance in adult congenital heart disease: Comparative severity, correlates, and prognostic implication (2005) Circulation, 112, pp. 828-835; Gratz, A., Hess, J., Hager, A., Self-estimated physical functioning poorly predicts actual exercise capacity in adolescents and adults with congenital heart disease (2009) Eur Heart J, 30, pp. 497-504; Giardini, A., Specchia, S., Berton, E., Sangiorgi, D., Coutsoumbas, G., Gargiulo, G., Oppido, G., Picchio, F.M., Strong and independent prognostic value of peak circulatory power in adults with congenital heart disease (2007) Am Heart J, 154, pp. 441-447; Colice, G.L., Shafazand, S., Griffin, J.P., Keenan, R., Bolliger, C.T., Physiologic evaluation of the patient with lung cancer being considered for resectional surgery: ACCP evidenced-based clinical practice guidelines (2nd edition) (2007) Chest, 132 (3 SUPPL.), pp. 161S-177S. , DOI 10.1378/chest.07-1359; Loewen, G.M., Watson, D., Kohman, L., Herndon Je, I.I., Shennib, H., Kernstine, K., Olak, J., Green, M., Preoperative exercise VO2 measurement for lung resection candidates: Results of Cancer and Leukemia Group B Protocol 9238 (2007) J Thorac Oncol, 2, pp. 619-625. , Cancer and Leukemia Group B; DeCamp Jr., M.M., Lipson, D., Krasna, M., Minai, O.A., McKenna Jr., R.J., Thomashow, B.M., The evaluation and preparation of the patient for lung volume reduction surgery (2008) Proceedings of the American Thoracic Society, 5 (4), pp. 427-431. , http://pats.atsjournals.org/cgi/reprint/5/4/427, DOI 10.1513/pats.200707-087ET; Fishman, A., Martinez, F., Naunheim, K., Piantadosi, S., Wise, R., Ries, A., Weinmann, G., Wood, D.E., A randomized trial comparing lung-volume-reduction surgery with medical therapy for severe emphysema (2003) N Engl J Med, 348, pp. 2059-2073. , National Emphysema Treatment Trial Research Group; Rich, S., Rabinovitch, M., Diagnosis and treatment of secondary (non-category 1) pulmonary hypertension (2008) Circulation, 118, pp. 2190-2199; Sun, X.G., Hansen, J.E., Oudiz, R.J., Wasserman, K., Gas exchange detection of exercise-induced right-to-left shunt in patients with primary pulmonary hypertension (2002) Circulation, 105, pp. 54-60; Ting, H., Sun, X.G., Chuang, M.L., Lewis, D.A., Hansen, J.E., Wasserman, K., A noninvasive assessment of pulmonary perfusion abnormality in patients with primary pulmonary hypertension (2001) Chest, 119, pp. 824-832; Miyamoto, S., Nagaya, N., Satoh, T., Kyotani, S., Sakamaki, F., Fujita, M., Nakanishi, N., Miyatake, K., Clinical correlates and prognostic significance of six-minute walk test in patients with primary pulmonary hypertension: Comparison with cardiopulmonary exercise testing (2000) Am J Respir Crit Care Med, 161, pp. 487-492; Kavanagh, T., Mertens, D.J., Hamm, L.F., Beyene, J., Kennedy, J., Corey, P., Shephard, R.J., Prediction of long-term prognosis in 12 169 men referred for cardiac rehabilitation (2002) Circulation, 106, pp. 666-671; Kavanagh, T., Mertens, D.J., Hamm, L.F., Beyene, J., Kennedy, J., Corey, P., Shephard, R.J., Peak oxygen intake and cardiac mortality in women referred for cardiac rehabilitation (2003) J Am Coll Cardiol, 42, pp. 2139-2143; Chaundhry, S., Arena, R., Wasserman, K., Hansen, J.E., Lewis, G.D., Myers, J., Chronos, N., Boden, W.E., Exercise-induced myocardial ischemia detected by cardiopulmonary exercise testing (2009) Am Heart J, 103, pp. 615-619; Belardinelli, R., Lacalaprice, F., Carle, F., Minnucci, A., Cianci, G., Perna, G., D'Eusanio, G., Exercise-induced myocardial ischaemia detected by car-diopulmonary exercise testing (2003) Eur Heart J, 24, pp. 1304-1313; Bussotti, M., Apostolo, A., Andreini, D., Palermo, P., Contini, M., Agostoni, P., Cardiopulmonary evidence of exercise-induced silent ischaemia (2006) Eur J Cardiovasc Prev Rehabil, 13, pp. 249-253; Greco, E.M., Guardini, S., Ferrario, M., Romano, S., How to program rate responsive pacemakers (2000) Pacing Clin Electrophysiol, 23, pp. 165-173; Duru, F., Cho, Y., Wilkoff, B.L., Cole, C.R., Adler, S., Jensen, D.N., Strobel, U., Candinas, R., Rate responsive pacing using transthoracic impedance minute ventilation sensors: A multicenter study on calibration stability (2002) Pacing Clin Electrophysiol, 25, pp. 1679-1684; Capucci, A., Boriani, G., Specchia, S., Marinelli, M., Santarelli, A., Magnani, B., Evaluation by cardiopulmonary exercise test of DDDR versus DDD pacing (1992) Pacing Clin Electrophysiol, 15, pp. 1908-1913; Lemke, B., Dryander, S.V., Jäger, D., MacHraoui, A., MacCarter, D., Barmeyer, J., Aerobic capacity in rate modulated pacing (1992) Pacing Clin Electrophysiol, 15, pp. 1914-1918; Lewalter, T., Rickli, H., MacCarter, D., Schwartze, P., Schimpf, R., Schumacher, B., Jung, W., Lüderitz, B., Oxygen uptake to work rate relation throughout peak exercise in normal subjects: Relevance for rate adaptive pacemaker programming (1999) Pacing Clin Electrophysiol, 22, pp. 769-775; Mathony, U., Schmidt, H., Gröger, C., Francis, D.P., Konzag, I., Müller-Werdan, U., Werdan, K., Syska, J., Optimal maximum tracking rate of dual-chamber pacemakers required by children and young adults for a maximal cardiorespiratory performance (2005) Pacing Clin Electrophysiol, 28, pp. 378-383; Alt, E.U., Schlegl, M.J., Matula, M.M., Intrinsic heart rate response as a predictor of rate-adaptive pacing benefit (1995) Chest, 107, pp. 925-930; Meine, M., Achtelik, M., Hexamer, M., Kloppe, A., Werner, J., Trappe, H.J., Assessment of the chronotropic response at the anaerobic threshold: An objective measure of chronotropic function (2000) Pacing Clin Electrophysiol, 23, pp. 1457-1467; Page, E., Defaye, P., Bonnet, J.L., Durand, C., Amblard, A., Comparison of the cardiopulmonary response to exercise in recipients of dual sensor DDDR pacemakers versus a healthy control group (2003) Pacing Clin Electrophysiol, 26, pp. 239-243; Madaric, J., Vanderheyden, M., Van Laethem, C., Verhamme, K., Feys, A., Goethals, M., Verstreken, S., Bartunek, J., Early and late effects of cardiac resynchronization therapy on exercise-induced mitral regurgitation: Relationship with left ventricular dyssynchrony, remodelling and cardiopulmonary performance (2007) Eur Heart J, 28, pp. 2134-2141; Strickberger, S.A., Conti, J., Daoud, E.G., Havranek, E., Mehra, M.R., Piña, I.L., Young, J., Patient selection for cardiac resynchronization therapy: From the Council on Clinical Cardiology Subcommittee on Electrocardiogra-phy and Arrhythmias and the Quality of Care and Outcomes Research Interdisciplinary Working Group, in collaboration with the Heart Rhythm Society (2005) Circulation, 111, pp. 2146-2150; Pardaens, K., Van Cleemput, J., Vanhaecke, J., Fagard, R.H., Atrial fibrillation is associated with a lower exercise capacity in male chronic heart failure patients (1997) Heart, 78, pp. 564-568; Agostoni, P., Emdin, M., Corrà, U., Veglia, F., Magrì, D., Tedesco, C.C., Berton, E., Guazzi, M., Permanent atrial fibrillation affects exercise capacity in chronic heart failure patients (2008) Eur Heart J, 29, pp. 2367-2372; Guazzi, M., Belletti, S., Bianco, E., Lenatti, L., Guazzi, M.D., Endothelial dysfunction and exercise performance in lone atrial fibrillation or associated with hypertension or diabetes: Different results with cardio-version (2006) Am J Physiol Heart Circ Physiol, 291, pp. H921-H928; Guazzi, M., Belletti, S., Tumminello, G., Fiorentini, C., Guazzi, M.D., Exercise hyperventilation, dyspnea sensation, and ergoreflex activation in lone atrial fibrillation (2004) Am J Physiol Heart Circ Physiol, 287, pp. H2899-H2905; Wozakowska-Kaplon, B., Opolski, G., Effects of sinus rhythm restoration in patients with persistent atrial fibrillation: A clinical, echocardiographic and hormonal study (2004) Int J Cardiol, 96, pp. 171-176; Lok, N.S., Lau, C.P., Oxygen uptake kinetics and cardiopulmonary performance in lone atrial fibrillation and the effects of sotalol (1997) Chest, 111, pp. 934-940; McCullough, P.A., Gallagher, M.J., Dejong, A.T., Sandberg, K.R., Trivax, J.E., Alexander, D., Kasturi, G., Franklin, B.A., Cardiorespiratory fitness and short-term complications after bariatric surgery (2006) Chest, 130, pp. 517-525; Santry, H.P., Gillen, D.L., Lauderdale, D.S., Trends in bariatric surgical procedures (2005) JAMA, 294, pp. 1909-1917; Eagle, K.A., Berger, P.B., Calkins, H., Chaitman, B.R., Ewy, G.A., Fleischmann, K.E., Fleisher, L.A., Smith Jr., S.C., ACC/AHA guideline update for perioperative cardiovascular evaluation for noncardiac surgery: Executive summary: A report of the American College of Cardiology/American Heart Association Task Force on Practice Guidelines (2002) Circulation, 105, pp. 1257-1267. , Committee to Update the 1996 Guidelines on Perioperative Cardiovascular Evaluation for Noncardiac Surgery [published correction appears in Circulation. 2006;113:e846]; Ainsworth, B.E., Haskell, W.L., Whitt, M.C., Irwin, M.L., Swartz, A.M., Strath, S.J., O'Brien, W.L., Leon, A.S., Compendium of physical activities: An update of activity codes and MET intensities (2000) Med Sci Sports Exerc, 32, pp. S498-S504; Jones, N., (1997) Clinical Exercise Testing, , Philadelphia Pa: Saunders; Morris, C.K., Myers, J., Froelicher, V.F., Kawaguchi, T., Ueshima, K., Hideg, A., Nomogram based on metabolic equivalents and age for assessing aerobic exercise capacity in men (1993) J Am Coll Cardiol, 22, pp. 175-182; Hansen, J.E., Sue, D.Y., Wasserman, K., Predicted values for clinical exercise testing (1984) Am Rev Respir Dis, 129, pp. S49-S55; Hsich, E., Gorodeski, E.Z., Starling, R.C., Blackstone, E.H., Ishwaran, H., Lauer, M.S., Importance of treadmill exercise time as an initial prognostic screening tool in patients with systolic left ventricular dysfunction (2009) Circulation, 119, pp. 3189-3197; Baba, R., Nagashima, M., Goto, M., Nagano, Y., Yokota, M., Tauchi, N., Nishibata, K., Oxygen intake efficiency slope: A new index of cardiore-spiratory functional reserve derived from the relationship between oxygen consumption and minute ventilation during incremental exercise (1996) Nagoya J Med Sci, 59, pp. 55-62; Van Laethem, C., Bartunek, J., Goethals, M., Nellens, P., Andries, E., Vander-Heyden, M., Oxygen uptake efficiency slope, a new submaximal parameter in evaluating exercise capacity in chronic heart failure patients (2005) Am Heart J, 149, pp. 175-180; Hollenberg, M., Tager, I.B., Oxygen uptake efficiency slope: An index of exercise performance and cardiopulmonary reserve requiring only sub-maximal exercise (2000) J Am Coll Cardiol, 36, pp. 194-201; Davies, L.C., Wensel, R., Georgiadou, P., Cicoira, M., Coats, A.J., Piepoli, M.F., Francis, D.P., Enhanced prognostic value from cardiopulmonary exercise testing in chronic heart failure by non-linear analysis: Oxygen uptake efficiency slope (2006) Eur Heart J, 27, pp. 684-690; Baba, R., Kubo, N., Morotome, Y., Iwagaki, S., Reproducibility of the oxygen uptake efficiency slope in normal healthy subjects (1999) Journal of Sports Medicine and Physical Fitness, 39 (3), pp. 202-206; Pogliaghi, S., Dussin, E., Tarperi, C., Cevese, A., Schena, F., Calculation of oxygen uptake efficiency slope based on heart rate reserve end-points in healthy elderly subjects (2007) Eur J Appl Physiol, 101, pp. 691-696; Arena, R., Myers, J., Hsu, L., Peberdy, M.A., Pinkstaff, S., Bensimhon, D., Chase, P., Guazzi, M., The minute ventilation/carbon dioxide production slope is prognostically superior to the oxygen uptake efficiency slope (2007) J Card Fail, 13, pp. 462-469; Myers, J., Arena, R., Dewey, F., Bensimhon, D., Abella, J., Hsu, L., Chase, P., Peberdy, M.A., A cardiopulmonary exercise testing score for predicting outcomes in patients with heart failure (2008) Am Heart J, 156, pp. 1177-1183; Arena, R., Peberdy, M.A., Reliability of resting end-tidal carbon dioxide in chronic heart failure (2005) J Cardiopulm Rehabil, 25, pp. 177-180; Jin, X., Weil, M.H., Tang, W., Povoas, H., Pernat, A., Xie, J., Bisera, J., End-tidal carbon dioxide as a noninvasive indicator of cardiac index during circulatory shock (2000) Crit Care Med, 28, pp. 2415-2419; Isserles, S.A., Breen, P.H., Can changes in end-tidal PCO2 measure changes in cardiac output? (1991) Anesth Analg, 73, pp. 808-814; Matsumoto, A., Itoh, H., Eto, Y., Kobayashi, T., Kato, M., Omata, M., Watanabe, H., Momomura, S., End-tidal CO2 pressure decreases during exercise in cardiac patients: Association with severity of heart failure and cardiac output reserve (2000) J Am Coll Cardiol, 36, pp. 242-249; Arena, R., Guazzi, M., Myers, J., Prognostic value of end-tidal carbon dioxide during exercise testing in heart failure (2007) International Journal of Cardiology, 117 (1), pp. 103-108. , DOI 10.1016/j.ijcard.2006.04.058, PII S0167527306005092; Arena, R., Myers, J., Abella, J., Pinkstaff, S., Brubaker, P., Moore, B., Kitzman, D., Guazzi, M., The partial pressure of resting end-tidal carbon dioxide predicts major cardiac events in patients with systolic heart failure (2008) Am Heart J, 156, pp. 982-988; Bradley, T.D., The ups and downs of periodic breathing: Implications for mortality in heart failure (2003) J Am Coll Cardiol, 41, pp. 2182-2184; Leite, J.J., Mansur, A.J., De Freitas, H.F., Chizola, P.R., Bocchi, E.A., Terra-Filho, M., Neder, J.A., Lorenzi-Filho, G., Periodic breathing during incremental exercise predicts mortality in patients with chronic heart failure evaluated for cardiac transplantation (2003) J Am Coll Cardiol, 41, pp. 2175-2181; Guazzi, M., Arena, R., Ascione, A., Piepoli, M., Guazzi, M.D., Exercise oscillatory breathing and increased ventilation to carbon dioxide production slope in heart failure: An unfavorable combination with high prognostic value (2007) Am Heart J, 153, pp. 859-867; Ribeiro, J.P., Periodic breathing in heart failure: Bridging the gap between the sleep laboratory and the exercise laboratory (2006) Circulation, 113, pp. 9-10; Somers, V.K., Sleep: A new cardiovascular frontier (2005) N Engl J Med., 353, pp. 2070-2073. , [published correction appears in N Engl J Med 2005;353:2523]; Hanly, P., Zuberi, N., Gray, R., Pathogenesis of Cheyne-Stokes respiration in patients with congestive heart failure: Relationship to arterial PCO2 (1993) Chest, 104, pp. 1079-1084; Ben-Dov, I., Sietsema, K.E., Casaburi, R., Wasserman, K., Evidence that circulatory oscillations accompany ventilatory oscillations during exercise in patients with heart failure (1992) Am Rev Respir Dis, 145, pp. 776-781; Francis, D.P., Willson, K., Davies, L.C., Coats, A.J., Piepoli, M., Quantitative general theory for periodic breathing in chronic heart failure and its clinical implications (2000) Circulation, 102, pp. 2214-2221; Ponikowski, P., Anker, S.D., Chua, T.P., Francis, D., Banasiak, W., Poole-Wilson, P.A., Coats, A.J., Piepoli, M., Oscillatory breathing patterns during wakefulness in patients with chronic heart failure: Clinical implications and role of augmented peripheral chemosensitivity (1999) Circulation, 100, pp. 2418-2424; Guazzi, M., Raimondo, R., Vicenzi, M., Arena, R., Proserpio, C., Sarzi Braga, S., Pedretti, R., Exercise oscillatory ventilation may predict sudden cardiac death in heart failure patients (2007) J Am Coll Cardiol, 50, pp. 299-308; Myers, J., Salleh, A., Buchanan, N., Smith, D., Neutel, J., Bowes, E., Froelicher, V.F., Ventilatory mechanisms of exercise intolerance in chronic heart failure (1992) Am Heart J, 124, pp. 710-719; Guazzi, M., Marenzi, G., Assanelli, E., Perego, G.B., Cattadori, G., Doria, E., Agostoni, P.G., Evaluation of the dead space/tidal volume ratio in patients with chronic congestive heart failure (1995) J Card Fail, 1, pp. 401-408; Jones, N.L., Robertson, D.G., Kane, J.W., Difference between end-tidal and arterial PCO2 in exercise (1979) J Appl Physiol, 47, pp. 954-960; Lewis, D.A., Sietsema, K.E., Casaburi, R., Sue, D.Y., Inaccuracy of nonin-vasive estimates of VD/VT in clinical exercise testing (1994) Chest, 106, pp. 1476-1480; Koike, A., Itoh, H., Kato, M., Sawada, H., Aizawa, T., Fu, L.T., Watanabe, H., Prognostic power of ventilatory responses during submaximal exercise in patients with chronic heart disease (2002) Chest, 121, pp. 1581-1588; Barstow, T.J., Casaburi, R., Wasserman, K., O2 uptake kinetics and the O2 deficit as related to exercise intensity and blood lactate (1993) J Appl Physiol, 75, pp. 755-762; Hickson, R.C., Bomze, H.A., Hollozy, J.O., Faster adjustment of O2 uptake to the energy requirement of exercise in the trained state (1978) J Appl Physiol, 44, pp. 877-881; Powers, S.K., Dodd, S., Beadle, R.E., Oxygen uptake kinetics in trained athletes differing in V? O 2max (1985) Eur J Appl Physiol Occup Physiol, 54, pp. 306-308; Sietsema, K.E., Ben-Dov, I., Zhang, Y.Y., Sullivan, C., Wasserman, K., Dynamics of oxygen uptake for submaximal exercise and recovery in patients with chronic heart failure (1994) Chest, 105, pp. 1693-1700; Sietsema, K.E., Cooper, D.M., Perloff, J.K., Rosove, M.H., Child, J.S., Canobbio, M.M., Whipp, B.J., Wasserman, K., Dynamics of oxygen uptake during exercise in adults with cyanotic congenital heart disease (1986) Circulation, 73, pp. 1137-1144; Koike, A., Yajima, T., Adachi, H., Shimizu, N., Kano, H., Sugimoto, K., Niwa, A., Hiroe, M., Evaluation of exercise capacity using submaximal exercise at a constant work rate in patients with cardiovascular disease (1995) Circulation, 91, pp. 1719-1724; Harris, R.C., Edwards, R.H., Hultman, E., Nordesjö, L.O., Nylind, B., Sahlin, K., The time course of phosphorylcreatine resynthesis during recovery of the quadriceps muscle in man (1976) Pflugers Arch, 367, pp. 137-142; Barstow, T.J., Lamarra, N., Whipp, B.J., Modulation of muscle and pulmonary O2 uptakes by circulatory dynamics during exercise (1990) J Appl Physiol, 68, pp. 979-989; Guazzi, M., Tumminello, G., Di Marco, F., Fiorentini, C., Guazzi, M.D., The effects of phosphodiesterase-5 inhibition with sildenafil on pulmonary hemodynamics and diffusion capacity, exercise ventilatory efficiency, and oxygen uptake kinetics in chronic heart failure (2004) J Am Coll Cardiol, 44, pp. 2339-2348; De Groote, P., Millaire, A., Decoulx, E., Nugue, O., Guimier, P., Ducloux, G., Kinetics of oxygen consumption during and after exercise in patients with dilated cardiomyopathy: New markers of exercise intolerance with clinical implications (1996) J Am Coll Cardiol, 28, pp. 168-175; Wilson, J.R., Rayos, G., Yeoh, T.K., Gothard, P., Dissociation between peak exercise oxygen consumption and hemodynamic dysfunction in potential heart transplant candidates (1995) J Am Coll Cardiol, 26, pp. 429-435; Chomsky, D.B., Lang, C.C., Rayos, G.H., Shyr, Y., Yeoh, T.K., Pierson III, R.N., Davis, S.F., Wilson, J.R., Hemodynamic exercise testing: A valuable tool in the selection of cardiac transplantation candidates (1996) Circulation, 94, pp. 3176-3183; Metra, M., Faggiano, P., D'Aloia, A., Nodari, S., Gualeni, A., Raccagni, D., Dei Cas, L., Use of cardiopulmonary exercise testing with hemodynamic monitoring in the prognostic assessment of ambulatory patients with chronic heart failure (1999) J Am Coll Cardiol, 33, pp. 943-950; Lang, C.C., Agostoni, P., Mancini, D.M., Prognostic significance and measurement of exercise-derived hemodynamic variables in patients with heart failure (2007) J Card Fail, 13, pp. 672-679; Stringer, W.W., Hansen, J.E., Wasserman, K., Cardiac output estimated noninvasively from oxygen uptake during exercise (1997) J Appl Physiol, 82, pp. 908-912; Lang, C.C., Karlin, P., Haythe, J., Tsao, L., Mancini, D.M., Ease of noninvasive measurement of cardiac output coupled with peak V? O2 determination at rest and during exercise in patients with heart failure (2007) Am J Cardiol, 99, pp. 404-405; Grossman, W., Blood flow measurement: Cardiac output and vascular resistance (2006) Grossman's Cardiac Catheterization, Angiography, and Intervention, pp. 148-162. , Baim D, ed Philadelphia, Pa: Lippincott Williams & Wilkins; Sun, X.-G., Hansen, J.E., Stringer, W.W., Ting, H., Wasserman, K., Carbon dioxide pressure-concentration relationship in arterial and mixed venous blood during exercise (2001) Journal of Applied Physiology, 90 (5), pp. 1798-1810; Agostoni, P., Cattadori, G., Apostolo, A., Contini, M., Palermo, P., Marenzi, G., Wasserman, K., Noninvasive measurement of cardiac output during exercise by inert gas rebreathing technique: A new tool for heart failure evaluation (2005) J Am Coll Cardiol, 46, pp. 1779-1781; Baum, M.M., Moss, J.A., Kumar, S., Wagner, P.D., Non-invasive measurement of cardiac output: Evaluation of new infrared absorption spectrometer (2006) Respir Physiol Neurobiol, 153, pp. 191-201; Johnson, B.D., Weisman, I.M., Zeballos, R.J., Beck, K.C., Emerging concepts in the evaluation of ventilatory limitation during exercise: The exercise tidal flow-volume loop (1999) Chest, 116, pp. 488-503; O'Donnell, D.E., Hyperinflation, dyspnea, and exercise intolerance in chronic obstructive pulmonary disease (2006) Proc Am Thorac Soc, 3, pp. 180-184; Johnson, B.D., Beck, K.C., Olson, L.J., O'Malley, K.A., Allison, T.G., Squires, R.W., Gau, G.T., Ventilatory constraints during exercise in patients with chronic heart failure (2000) Chest, 117, pp. 321-332; Dempsey, J.A., McKenzie, D.C., Haverkamp, H.C., Eldridge, M.W., Update in the understanding of respiratory limitations to exercise performance in fit, active adults (2008) Chest, 134, pp. 613-622.

971. Ben Morrison T, Rea RF, Hodge DO, Crusan D, Koestler C, Asirvatham SJ, et al. Risk factors for implantable defibrillator lead fracture in a recalled and a nonrecalled lead. J Cardiovasc Electrophysiol. 2010;21(6):671-7. doi: 10.1111/j.1540-8167.2009.01683.x.

972. Bernardes J, Ayres-De-Campos D. The persistent challenge of foetal heart rate monitoring. Curr Opin Obstet Gynecol. 2010;22(2):104-9. doi: 10.1097/GCO.0b013e328337233c.

973. Bhanji F, Mancini ME, Sinz E, Rodgers DL, McNeil MA, Hoadley TA, et al. Part 16: Education, implementation, and teams: 2010 American Heart Association Guidelines for Cardiopulmonary Resuscitation and Emergency Cardiovascular Care. Circulation. 2010;122(SUPPL. 3):S920-S33. doi: 10.1161/CIRCULATIONAHA.110.971135

10.1007/s10459-009-9207-x; (2008) Program Administration Manual, , 4th ed. Dallas Tex: American Heart Association; Weiss, K.B., Future of board certification in a new era of public accountability (2010) J Am Board Fam Med, 23 (SUPPL. 1), pp. S32-S39; Miles, P.V., Maintenance of certification: The role of the American Board of Pediatrics in improving children's health care (2009) Pediatr Clin North Am, 56, pp. 987-994; Smith, K.K., Gilcreast, D., Pierce, K., Evaluation of staff's retention of ACLS and BLS skills (2008) Resuscitation, 78, pp. 59-65; Woollard, M., Whitfeild, R., Smith, A., Colquhoun, M., Newcombe, R.G., Vetteer, N., Chamberlain, D., Skill acquisition and retention in automated external defibrillator (AED) use and CPR by lay responders: A prospective study (2004) Resuscitation, 60, pp. 17-28; Spooner, B.B., Fallaha, J.F., Kocierz, L., Smith, C.M., Smith, S.C., Perkins, G.D., An evaluation of objective feedback in basic life support (BLS) training (2007) Resuscitation, 73, pp. 417-424; Berden, H.J., Willems, F.F., Hendrick, J.M., Pijls, N.H., Knape, J.T., (1993) How Frequently Should Basic Cardiopulmonary Resuscitation Training Be Repeated to Maintain Adequate Skills? BMJ, 306, pp. 1576-1577; Woollard, M., Whitfield, R., Newcombe, R.G., Colquhoun, M., Vetter, N., Chamberlain, D., Optimal refresher training intervals for AED and CPR skills: A randomised controlled trial (2006) Resuscitation, 71, pp. 237-247; Duran, R., Aladag, N., Vatansever, U., Kucukugurluoglu, Y., Sut, N., Acunas, B., Proficiency and knowledge gained and retained by pediatric residents after neonatal resuscitation course (2008) Pediatr Int, 50, pp. 644-647; Anthonypillai, F., Retention of advanced cardiopulmonary resuscitation knowledge by intensive care trained nurses (1992) Intensive Crit Care Nurs, 8, pp. 180-184; Boonmak, P., Boonmak, S., Srichaipanha, S., Poomsawat, S., Knowledge and skill after brief ACLS training (2004) J Med Assoc Thai, 87, pp. 1311-1314; Kaye, W., Wynne, G., Marteau, T., Dubin, H.G., Rallis, S.F., Simons, R.S., Evans, T.R., An advanced resuscitation training course for preregistration house officers (1990) J R Coll Physicians Lond, 24, pp. 51-54; Skidmore, M.B., Urquhart, H., Retention of skills in neonatal resuscitation (2001) Paediatr Child Health, 6, pp. 31-35; Semeraro, F., Signore, L., Cerchiari, E.L., Retention of CPR performance in anaesthetists (2006) Resuscitation, 68, pp. 101-108; Trevisanuto, D., Ferrarese, P., Cavicchioli, P., Fasson, A., Zanardo, V., Zacchello, F., Knowledge gained by pediatric residents after neonatal resuscitation program courses (2005) Paediatr Anaesth, 15, pp. 944-947; Young, R., King, L., An evaluation of knowledge and skill retention following an in-house advanced life support course (2000) Nurs Crit Care, 5, pp. 7-14; Duran, R., Sen, F., Na, Vatansever, U., Acunaçs, B., Knowledge gained and retained by neonatal nurses following neonatal resuscitation program course (2007) Turk Pediatr Ars, 42, pp. 153-155; Grant, E.C., Marczinski, C.A., Menon, K., Using pediatric advanced life support in pediatric residency training: Does the curriculum need resuscitation? (2007) Pediatr Crit Care Med, 8, pp. 433-439; O'Steen, D.S., Kee, C.C., Minick, M.P., The retention of advanced cardiac life support knowledge among registered nurses (1996) J Nurs Staff Dev, 12, pp. 66-72; Hammond, F., Saba, M., Simes, T., Cross, R., Advanced life support: Retention of registered nurses' knowledge 18 months after initial training (2000) Aust Crit Care, 13, pp. 99-104; Andresen, D., Arntz, H.R., Grafling, W., Hoffmann, S., Hofmann, D., Kraemer, R., Krause-Dietering, B., Wegscheider, K., Public access resuscitation program including defibrillator training for laypersons: A randomized trial to evaluate the impact of training course duration (2008) Resuscitation, 76, pp. 419-424; Wik, L., Myklebust, H., Auestad, B.H., Steen, P.A., Twelve-month retention of CPR skills with automatic correcting verbal feedback (2005) Resuscitation, 66, pp. 27-30; Christenson, J., Nafziger, S., Compton, S., Vijayaraghavan, K., Slater, B., Ledingham, R., Powell, J., McBurnie, M.A., The effect of time on CPR and automated external defibrillator skills in the Public Access Defibrillation Trial (2007) Resuscitation, 74, pp. 52-62; Riegel, B., Nafziger, S.D., McBurnie, M.A., Powell, J., Ledingham, R., Sehra, R., Mango, L., Henry, M.C., How well are cardiopulmonary resuscitation and automated external defibrillator skills retained over time? Results from the Public Access Defibrillation (PAD) Trial (2006) Acad Emerg Med, 13, pp. 254-263; Choa, M., Park, I., Chung, H.S., Yoo, S.K., Shim, H., Kim, S., The effectiveness of cardiopulmonary resuscitation instruction: Animation versus dispatcher through a cellular phone (2008) Resuscitation, 77, pp. 87-94; Choa, M., Cho, J., Choi, Y.H., Kim, S., Sung, J.M., Chung, H.S., Animation-assisted CPRII program as a reminder tool in achieving effective one-person-CPR performance (2009) Resuscitation, 80, pp. 680-684; Ertl, L., Christ, F., Significant improvement of the quality of bystander first aid using an expert system with a mobile multimedia device (2007) Resuscitation, 74, pp. 286-295; Ward, P., Johnson, L.A., Mulligan, N.W., Ward, M.C., Jones, D.L., Improving cardiopulmonary resuscitation skills retention: Effect of two checklists designed to prompt correct performance (1997) Resuscitation, 34, pp. 221-225; Merchant, R.M., Abella, B.S., Abotsi, E.J., Smith, T.M., Long, J.A., Trudeau, M.E., Leary, M., Asch, D.A., Cell telephone car-diopulmonary resuscitation: Audio instructions when needed by lay rescuers: A randomized, controlled trial (2010) Ann Emerg Med., 55, pp. 538e1-543e1; Lerner, C., Gaca, A.M., Frush, D.P., Hohenhaus, S., Ancarana, A., Seelinger, T.A., Frush, K., Enhancing pediatric safety: Assessing and improving resident competency in life-threatening events with a computer-based interactive resuscitation tool (2009) Pediatr Radiol, 39, pp. 703-709; Schneider, A.J., Murray, W.B., Mentzer, S.C., Miranda, F., Vaduva, S., "Helper": A critical events prompter for unexpected emergencies (1995) J Clin Monit, 11, pp. 358-364; Berkenstadt, H., Yusim, Y., Ziv, A., Ezri, T., Perel, A., An assessment of a point-of-care information system for the anesthesia provider in simulated malignant hyperthermia crisis (2006) Anesth Analg, 102, pp. 530-532; Harrison, T.K., Manser, T., Howard, S.K., Gaba, D.M., Use of cognitive aids in a simulated anesthetic crisis (2006) Anesth Analg, 103, pp. 551-556; Zanner, R., Wilhelm, D., Feussner, H., Schneider, G., Evaluation of M-AID, a first aid application for mobile phones (2007) Resuscitation, 74, pp. 487-494; Mills, P.D., Derosier, J.M., Neily, J., McKnight, S.D., Weeks, W.B., Bagian, J.P., A cognitive aid for cardiac arrest: You can't use it if you don't know about it (2004) Jt Comm J Qual Saf, 30, pp. 488-496; Neily, J., Derosier, J.M., Mills, P.D., Bishop, M.J., Weeks, W.B., Bagian, J.P., Awareness and use of a cognitive aid for anesthesiology (2007) Jt Comm J Qual Patient Saf, 33, pp. 502-511; Beckers, S.K., Skorning, M.H., Fries, M., Bickenbach, J., Beuerlein, S., Derwall, M., Kuhlen, R., Rossaint, R., CPREzy improves performance of external chest compressions in simulated cardiac arrest (2007) Resuscitation, 72, pp. 100-107; Monsieurs, K.G., De Regge, M., Vogels, C., Calle, P.A., Improved basic life support performance by ward nurses using the CAREvent Public Access Resuscitator (PAR) in a simulated setting (2005) Resuscitation, 67, pp. 45-50; Sutton, R.M., Donoghue, A., Myklebust, H., Srikantan, S., Byrne, A., Priest, M., Zoltani, Z., Nadkarni, V., The voice advisory manikin (VAM): An innovative approach to pediatric lay provider basic life support skill education (2007) Resuscitation, 75, pp. 161-168; Wik, L., Thowsen, J., Steen, P.A., An automated voice advisory manikin system for training in basic life support without an instructor: A novel approach to CPR training (2001) Resuscitation, 50, pp. 167-172; Wik, L., Myklebust, H., Auestad, B.H., Steen, P.A., Retention of basic life support skills 6 months after training with an automated voice advisory manikin system without instructor involvement (2002) Resuscitation, 52, pp. 273-279; Dine, C.J., Gersh, R.E., Leary, M., Riegel, B.J., Bellini, L.M., Abella, B.S., Improving cardiopulmonary resuscitation quality and resuscitation training by combining audiovisual feedback and debriefing (2008) Crit Care Med, 36, pp. 2817-2822; Boyle, A.J., Wilson, A.M., Connelly, K., McGuigan, L., Wilson, J., Whitbourn, R., Improvement in timing and effectiveness of external cardiac compressions with a new non-invasive device: The CPR-Ezy (2002) Resuscitation, 54, pp. 63-67; Elding, C., Baskett, P., Hughes, A., The study of the effectiveness of chest compressions using the CPR-plus (1998) Resuscitation, 36, pp. 169-173; Handley, A.J., Handley, S.A., Improving CPR performance using an audible feedback system suitable for incorporation into an automated external defibrillator (2003) Resuscitation, 57, pp. 57-62; Jantti, H., Silfvast, T., Turpeinen, A., Kiviniemi, V., Uusaro, A., Influence of chest compression rate guidance on the quality of cardiopulmonary resuscitation performed on manikins (2009) Resuscitation, 80, pp. 453-457; Noordergraaf, G.J., Drinkwaard, B.W., Van Berkom, P.F., Van Hemert, H.P., Venema, A., Scheffer, G.J., Noordergraaf, A., The quality of chest compressions by trained personnel: The effect of feedback, via the CPREzy, in a randomized controlled trial using a manikin model (2006) Resuscitation, 69, pp. 241-252; Oh, J.H., Lee, S.J., Kim, S.E., Lee, K.J., Choe, J.W., Kim, C.W., Effects of audio tone guidance on performance of CPR in simulated cardiac arrest with an advanced airway (2008) Resuscitation, 79, pp. 273-277; Perkins, G.D., Augre, C., Rogers, H., Allan, M., Thickett, D.R., CPREzy: An evaluation during simulated cardiac arrest on a hospital bed (2005) Resuscitation, 64, pp. 103-108; Thomas, S.H., Stone, C.K., Austin, P.E., March, J.A., Brinkley, S., Utilization of a pressure-sensing monitor to improve in-flight chest compressions (1995) Am J Emerg Med, 13, pp. 155-157; Williamson, L.J., Larsen, P.D., Tzeng, Y.C., Galletly, D.C., Effect of automatic external defibrillator audio prompts on cardiopulmonary resuscitation performance (2005) Emerg Med J, 22, pp. 140-143; Kern, K.B., Stickney, R.E., Gallison, L., Smith, R.E., Metronome improves compression and ventilation rates during CPR on a manikin in a randomized trial (2010) Resuscitation, 81, pp. 206-210; Peberdy, M.A., Silver, A., Ornato, J.P., Effect of caregiver gender, age, and feedback prompts on chest compression rate and depth (2009) Resuscitation, 80, pp. 1169-1174; Rawlins, L., Woollard, M., Williams, J., Hallam, P., Effect of listening to Nellie the Elephant during CPR training on performance of chest compressions by lay people: Randomised crossover trial (2009) BMJ, 339, pp. b4707; Abella, B.S., Edelson, D.P., Kim, S., Retzer, E., Myklebust, H., Barry, A.M., O'Hearn, N., Becker, L.B., CPR quality improvement during in-hospital cardiac arrest using a real-time audiovisual feedback system (2007) Resuscitation, 73, pp. 54-61; Chiang, W.C., Chen, W.J., Chen, S.Y., Ko, P.C., Lin, C.H., Tsai, M.S., Chang, W.T., Ma, M.H., Better adherence to the guidelines during cardiopulmonary resuscitation through the provision of audio-prompts (2005) Resuscitation, 64, pp. 297-301; Fletcher, D., Galloway, R., Chamberlain, D., Pateman, J., Bryant, G., Newcombe, R.G., Basics in advanced life support: A role for download audit and metronomes (2008) Resuscitation, 78, pp. 127-134; Kramer-Johansen, J., Myklebust, H., Wik, L., Fellows, B., Svensson, L., Sorebo, H., Steen, P.A., Quality of out-of-hospital cardiopulmonary resuscitation with real time automated feedback: A prospective interventional study (2006) Resuscitation, 71, pp. 283-292; Niles, D., Nysaether, J., Sutton, R., Nishisaki, A., Abella, B.S., Arbogast, K., Maltese, M.R., Nadkarni, V., Leaning is common during in-hospital pediatric CPR, and decreased with automated corrective feedback (2009) Resuscitation, 80, pp. 553-557; Nishisaki, A., Nysaether, J., Sutton, R., Maltese, M., Niles, D., Donoghue, A., Bishnoi, R., Nadkarni, V., Effect of mattress deflection on CPR quality assessment for older children and adolescents (2009) Resuscitation, 80, pp. 540-545; Perkins, G.D., Boyle, W., Bridgestock, H., Davies, S., Oliver, Z., Bradburn, S., Green, C., Cooke, M.W., Quality of CPR during advanced resuscitation training (2008) Resuscitation, 77, pp. 69-74; O'Donnell, J., Rodgers, D., Lee, W., Edelson, D., Haag, J., Hamilton, M., Hoadley, T., Meeks, R., (2009) Structured and Supported Debriefing, , Dallas Tex: American Heart Association; Savoldelli, G.L., Naik, V.N., Park, J., Joo, H.S., Chow, R., Hamstra, S.J., Value of debriefing during simulated crisis management: Oral versus video-assisted oral feedback (2006) Anesthesiology, 105, pp. 279-285; Morgan, P.J., Tarshis, J., Leblanc, V., Cleave-Hogg, D., Desousa, S., Haley, M.F., Herold-Mcilroy, J., Law, J.A., Efficacy of high-fidelity simulation debriefing on the performance of practicing anaesthetists in simulated scenarios (2009) Br J Anaesth, 103, pp. 531-537; Falcone Jr., R.A., Daugherty, M., Schweer, L., Patterson, M., Brown, R.L., Garcia, V.F., Multidisciplinary pediatric trauma team training using high-fidelity trauma simulation (2008) J Pediatr Surg, 43, pp. 1065-1071; Weng, T.I., Huang, C.H., Ma, M.H., Chang, W.T., Liu, S.C., Wang, T.D., Chen, W.J., Improving the rate of return of spontaneous circulation for out-of-hospital cardiac arrests with a formal, structured emergency resuscitation team (2004) Resuscitation, 60, pp. 137-142; Nichol, G., Thomas, E., Callaway, C.W., Hedges, J., Powell, J.L., Aufderheide, T.P., Rea, T., Stiell, I., Regional variation in out-of-hospital cardiac arrest incidence and outcome (2008) JAMA, 300, pp. 1423-1431; White, R.D., Bunch, T.J., Hankins, D.G., Evolution of a community-wide early defibrillation programme experience over 13 years using police/fire personnel and paramedics as responders (2005) Resuscitation, 65, pp. 279-283; Neumar, R.W., Nolan, J.P., Adrie, C., Aibiki, M., Berg, R.A., Bottiger, B.W., Callaway, C., Vanden Hoek, T., Post-cardiac arrest syndrome: Epidemiology, pathophysiology, treatment, and prognostication: A consensus statement from the International Liaison Committee on Resuscitation (2008) Circulation, 118, pp. 2452-2483. , (American Heart Association, Australian and New Zealand Council on Resuscitation, European Resuscitation Council, Heart and Stroke Foundation of Canada, InterAmerican Heart Foundation, Resuscitation Council of Asia, and the Resuscitation Council of Southern Africa); the American Heart Association Emergency Cardiovascular Care Committee; the Council on Cardiovascular Surgery and Anesthesia; the Council on Cardiopulmonary, Perioperative, and Critical Care; the Council on Clinical Cardiology; and the Stroke Council; Devita, M.A., Bellomo, R., Hillman, K., Kellum, J., Rotondi, A., Teres, D., Auerbach, A., Galhotra, S., Findings of the first consensus conference on medical emergency teams (2006) Crit Care Med, 34, pp. 2463-2478; Baxter, A.D., Cardinal, P., Hooper, J., Patel, R., Medical emergency teams at the Ottawa Hospital: The first two years (2008) Can J Anaesth, 55, pp. 223-231; Bellomo, R., Goldsmith, D., Uchino, S., Buckmaster, J., Hart, G.K., Opdam, H., Silvester, W., Gutteridge, G., A prospective before-and-after trial of a medical emergency team (2003) Med J Aust, 179, pp. 283-287; Benson, L., Mitchell, C., Link, M., Carlson, G., Fisher, J., Using an advanced practice nursing model for a rapid response team (2008) Jt Comm J Qual Patient Saf, 34, pp. 743-747; Bertaut, Y., Campbell, A., Goodlett, D., Implementing a rapid-response team using a nurse-to-nurse consult approach (2008) J Vasc Nurs, 26, pp. 37-42; Buist, M.D., Moore, G.E., Bernard, S.A., Waxman, B.P., Anderson, J.N., Nguyen, T.V., Effects of a medical emergency team on reduction of incidence of and mortality from unexpected cardiac arrests in hospital: Preliminary study (2002) BMJ, 324, pp. 387-390; Buist, M., Harrison, J., Abaloz, E., Van Dyke, S., Six year audit of cardiac arrests and medical emergency team calls in an Australian outer metropolitan teaching hospital (2007) BMJ, 335, pp. 1210-1212; Chamberlain, B., Donley, K., Maddison, J., Patient outcomes using a rapid response team (2009) Clin Nurse Spec, 23, pp. 11-12; Dacey, M.J., Mirza, E.R., Wilcox, V., Doherty, M., Mello, J., Boyer, A., Gates, J., Baute, R., The effect of a rapid response team on major clinical outcome measures in a community hospital (2007) Crit Care Med, 35, pp. 2076-2082; DeVita, M.A., Braithwaite, R.S., Mahidhara, R., Stuart, S., Foraida, M., Simmons, R.L., Use of medical emergency team responses to reduce hospital cardiopulmonary arrests (2004) Quality and Safety in Health Care, 13 (4), pp. 251-254. , DOI 10.1136/qshc.2003.006585; Gould, D., Promoting patient safety: The rapid medical response team (2007) Perm J, 11, pp. 26-34; Hatler, C., Mast, D., Bedker, D., Johnson, R., Corderella, J., Torres, J., King, D., Plueger, M., Implementing a rapid response team to decrease emergencies outside the ICU: One hospital's experience (2009) Medsurg Nurs, 18, pp. 84-90. , 126; Jolley, J., Bendyk, H., Holaday, B., Lombardozzi, K.A., Harmon, C., Rapid response teams: Do they make a difference? (2007) Dimens Crit Care Nurs, 26, pp. 253-260; Jones, D., Bellomo, R., Bates, S., Warrillow, S., Goldsmith, D., Hart, G., Opdam, H., Gutteridge, G., Long term effect of a medical emergency team on cardiac arrests in a teaching hospital (2005) Crit Care, 9, pp. R808-R815; Jones, D., Bellomo, R., Bates, S., Warrillow, S., Goldsmith, D., Hart, G., Opdam, H., Patient monitoring and the timing of cardiac arrests and medical emergency team calls in a teaching hospital (2006) Intensive Care Med, 32, pp. 1352-1356; Moldenhauer, K., Sabel, A., Chu, E.S., Mehler, P.S., Clinical triggers: An alternative to a rapid response team (2009) Jt Comm J Qual Patient Saf, 35, pp. 164-174; Offner, P.J., Heit, J., Roberts, R., Implementation of a rapid response team decreases cardiac arrest outside of the intensive care unit (2007) J Trauma, 62, pp. 1223-1227; Chan, P.S., Khalid, A., Longmore, L.S., Berg, R.A., Kosiborod, M., Spertus, J.A., Hospital-wide code rates and mortality before and after implementation of a rapid response team. (2008) JAMA, 300, pp. 2506-2513; Hillman, K., Chen, J., Cretikos, M., Bellomo, R., Brown, D., Doig, G., Finfer, S., Flabouris, A., Introduction of the medical emergency team (MET) system: A cluster-randomised controlled trial (2005) Lancet, 365, pp. 2091-2097; Kenward, G., Castle, N., Hodgetts, T., Shaikh, L., Evaluation of a medical emergency team one year after implementation (2004) Resuscitation, 61, pp. 257-263; King, E., Horvath, R., Shulkin, D.J., Establishing a rapid response team (RRT) in an academic hospital: One year's experience (2006) J Hosp Med, 1, pp. 296-305; McFarlan, S.J., Hensley, S., Implementation and outcomes of a rapid response team (2007) J Nurs Care Qual, 22, pp. 307-313; Rothschild, J.M., Woolf, S., Finn, K.M., Friedberg, M.W., Lemay, C., Furbush, K.A., Williams, D.H., Bates, D.W., A controlled trial of a rapid response system in an academic medical center (2008) Jt Comm J Qual Patient Saf, 34, pp. 417-425. , 365; Hunt, E.A., Zimmer, K.P., Rinke, M.L., Shilkofski, N.A., Matlin, C., Garger, C., Dickson, C., Miller, M.R., Transition from a traditional code team to a medical emergency team and categorization of cardiopulmonary arrests in a children's center (2008) Arch Pediatr Adolesc Med, 162, pp. 117-122; Brilli, R.J., Gibson, R., Luria, J.W., Wheeler, T.A., Shaw, J., Linam, M., Kheir, J., McBride, M., Implementation of a medical emergency team in a large pediatric teaching hospital prevents respiratory and cardiopulmonary arrests outside the intensive care unit (2007) Pediatr Crit Care Med, 8, pp. 236-246; Sharek, P.J., Parast, L.M., Leong, K., Coombs, J., Earnest, K., Sullivan, J., Frankel, L.R., Roth, S.J., Effect of a rapid response team on hospital-wide mortality and code rates outside the ICU in a children's hospital (2007) JAMA, 298, pp. 2267-2274; Tibballs, J., Kinney, S., Reduction of hospital mortality and of preventable cardiac arrest and death on introduction of a pediatric medical emergency team (2009) Pediatr Crit Care Med, 10, pp. 306-312; Tibballs, J., Kinney, S., Duke, T., Oakley, E., Hennessy, M., Reduction of paediatric in-patient cardiac arrest and death with a medical emergency team: Preliminary results (2005) Arch Dis Child, 90, pp. 1148-1152; Chan, P.S., Jain, R., Nallmothu, B.K., Berg, R.A., Sasson, C., Rapid response teams: A systematic review and meta-analysis. (2010) Arch Intern Med, 170, pp. 18-26; Devita, M.A., Smith, G.B., Adam, S.K., Adams-Pizarro, I., Buist, M., Bellomo, R., Bonello, R., Winters, B., "Identifying the hospitalised patient in crisis": A consensus conference on the afferent limb of rapid response systems (2010) Resuscitation, 81, pp. 375-382; Engdahl, J., Abrahamsson, P., Bang, A., Lindqvist, J., Karlsson, T., Herlitz, J., Is hospital care of major importance for outcome after out-of-hospital cardiac arrest? Experience acquired from patients with out-of-hospital cardiac arrest resuscitated by the same Emergency Medical Service and admitted to one of two hospitals over a 16-year period in the municipality of Goteborg (2000) Resuscitation, 43, pp. 201-211; Langhelle, A., Tyvold, S.S., Lexow, K., Hapnes, S.A., Sunde, K., Steen, P.A., In-hospital factors associated with improved outcome after out-of-hospital cardiac arrest: A comparison between four regions in Norway (2003) Resuscitation, 56, pp. 247-263; Carr, B.G., Goyal, M., Band, R.A., Gaieski, D.F., Abella, B.S., Merchant, R.M., Branas, C.C., Neumar, R.W., A national analysis of the relationship between hospital factors and post-cardiac arrest mortality (2009) Intensive Care Med, 35, pp. 505-511; Liu, J.M., Yang, Q., Pirrallo, R.G., Klein, J.P., Aufderheide, T.P., Hospital variability of out-of-hospital cardiac arrest survival (2008) Prehosp Emerg Care, 12, pp. 339-346; Carr, B.G., Kahn, J.M., Merchant, R.M., Kramer, A.A., Neumar, R.W., Inter-hospital variability in post-cardiac arrest mortality (2009) Resuscitation, 80, pp. 30-34; Herlitz, J., Engdahl, J., Svensson, L., Angquist, K.A., Silfverstolpe, J., Holmberg, S., Major differences in 1-month survival between hospitals in Sweden among initial survivors of out-of-hospital cardiac arrest (2006) Resuscitation, 70, pp. 404-409; Keenan, S.P., Dodek, P., Martin, C., Priestap, F., Norena, M., Wong, H., Variation in length of intensive care unit stay after cardiac arrest: Where you are is as important as who you are (2007) Crit Care Med, 35, pp. 836-841; Oddo, M., Schaller, M.D., Feihl, F., Ribordy, V., Liaudet, L., From evidence to clinical practice: Effective implementation of therapeutic hypothermia to improve patient outcome after cardiac arrest (2006) Crit Care Med, 34, pp. 1865-1873; Sunde, K., Pytte, M., Jacobsen, D., Mangschau, A., Jensen, L.P., Smedsrud, C., Draegni, T., Steen, P.A., Implementation of a standardised treatment protocol for post resuscitation care after out-of-hospital cardiac arrest (2007) Resuscitation, 73, pp. 29-39; Knafelj, R., Radsel, P., Ploj, T., Noc, M., Primary percutaneous coronary intervention and mild induced hypothermia in comatose survivors of ventricular fibrillation with ST-elevation acute myocardial infarction (2007) Resuscitation, 74, pp. 227-234; Wolfrum, S., Pierau, C., Radke, P.W., Schunkert, H., Kurowski, V., Mild therapeutic hypothermia in patients after out-of-hospital cardiac arrest due to acute ST-segment elevation myocardial infarction undergoing immediate percutaneous coronary intervention (2008) Crit Care Med, 36, pp. 1780-1786; Gaieski, D.F., Band, R.A., Abella, B.S., Neumar, R.W., Fuchs, B.D., Kolansky, D.M., Merchant, R.M., Goyal, M., Early goal-directed hemodynamic optimization combined with therapeutic hypothermia in comatose survivors of out-of-hospital cardiac arrest (2009) Resuscitation, 80, pp. 418-424; Bradley, E.H., Herrin, J., Wang, Y., Barton, B.A., Webster, T.R., Mattera, J.A., Roumanis, S.A., Krumholz, H.M., Strategies for reducing the door-to-balloon time in acute myocardial infarction (2006) N Engl J Med, 355, pp. 2308-2320; Lamonte, M.P., Bahouth, M.N., Magder, L.S., Alcorta, R.L., Bass, R.R., Browne, B.J., Floccare, D.J., Gaasch, W.R., A regional system of stroke care provides thrombolytic outcomes comparable with the NINDS stroke trial (2009) Ann Emerg Med, 54, pp. 319-327; Organised inpatient (stroke unit) care for stroke (2007) Cochrane Database Syst Rev, pp. CD000197. , Oct 17; MacKenzie, E.J., Rivara, F.P., Jurkovich, G.J., Nathens, A.B., Frey, K.P., Egleston, B.L., Salkever, D.S., Scharfstein, D.O., A national evaluation of the effect of trauma-center care on mortality (2006) N Engl J Med, 354, pp. 366-378; Nichol, G., Aufderheide, T.P., Eigel, B., Neumar, R.W., Lurie, K.G., Bufalino, V.J., Callaway, C.W., Peterson, E., Regional systems of care for out-of-hospital cardiac arrest: A policy statement from the American Heart Association (2010) Circulation, 121, pp. 709-729; Ali, J., Adam, R., Stedman, M., Howard, M., Williams, J., Cognitive and attitudinal impact of the Advanced Trauma Life Support program in a developing country (1994) J Trauma, 36, pp. 695-702; Bergman, S., Deckelbaum, D., Lett, R., Haas, B., Demyttenaere, S., Munthali, V., Mbembati, N., Razek, T., Assessing the impact of the trauma team training program in Tanzania (2008) J Trauma, 65, pp. 879-883; Bhat, B.V., Biswal, N., Bhatia, B.D., Nalini, P., Undergraduate training in neonatal resuscitation: A modified approach (1993) Indian J Matern Child Health, 4, pp. 87-88; Carlo, W.A., Wright, L.L., Chomba, E., McClure, E.M., Carlo, M.E., Bann, C.M., Collins, M., Harris, H., Educational impact of the neonatal resuscitation program in low-risk delivery centers in a developing country (2009) J Pediatr, 154, pp. 504e5-508e5; Couper, I.D., Thurley, J.D., Hugo, J.F., The neonatal resuscitation training project in rural South Africa (2005) Rural Remote Health, 5, p. 459; Ergenekon, E., Koc, E., Atalay, Y., Soysal, S., Neonatal resuscitation course experience in Turkey (2000) Resuscitation, 45, pp. 225-227; Husum, H., Gilbert, M., Wisborg, T., Training pre-hospital trauma care in low-income countries: The "village University" experience (2003) Med Teach, 25, pp. 142-148; Jabir, M.M., Doglioni, N., Fadhil, T., Zanardo, V., Trevisanuto, D., Knowledge and practical performance gained by Iraqi residents after participation to a neonatal resuscitation program course (2009) Acta Paediatr, 98, pp. 1265-1268; Kimura, A., Okada, K., Kobayashi, K., Inaka, A., Hagiwara, Y., Sakamoto, T., Sugimoto, N., Emoto, M., Introductory adult cardiac life support course for Vietnamese healthcare workers (2008) Resuscitation, 79, pp. 511-512; McClure, E.M., Carlo, W.A., Wright, L.L., Chomba, E., Uxa, F., Lincetto, O., Bann, C., Evaluation of the educational impact of the WHO Essential Newborn Care course in Zambia (2007) Acta Paediatr, 96, pp. 1135-1138; Trevisanuto, D., Ibrahim, S.A., Doglioni, N., Salvadori, S., Ferrarese, P., Zanardo, V., Neonatal resuscitation courses for pediatric residents: Comparison between Khartoum (Sudan) and Padova (Italy) (2007) Paediatr Anaesth, 17, pp. 28-31; Urbano, J., Matamoros, M.M., Lopez-Herce, J., Carrillo, A.P., Ordonez, F., Moral, R., Mencia, S., A paediatric cardiopulmonary resuscitation training project in Honduras (2010) Resuscitation, 81, pp. 472-476; Zaeemul, H., Qureshi, F., Hafeez, A., Zafar, S., Mohamud, B.K., Southal, D.P., Evidence for improvement in the quality of care given during emergencies in pregnancy, infancy and childhood following training in life-saving skills: A postal survey (2009) J Pak Med Assoc, 59, pp. 22-26; Ali, J., Adam, R., Butler, A.K., Chang, H., Howard, M., Gonsalves, D., Pitt-Miller, P., Williams, J.I., Trauma outcome improves following the advanced trauma life support program in a developing country (1993) J Trauma, 34, pp. 890-898; Ali, J., Adam, R.U., Gana, T.J., Williams, J.I., Trauma patient outcome after the Prehospital Trauma Life Support program (1997) J Trauma, 42, pp. 1018-1021; Arreola-Risa, C., Mock, C., Herrera-Escamilla, A.J., Contreras, I., Vargas, J., Cost-effectiveness and benefit of alternatives to improve training for prehospital trauma care in Mexico (2004) Prehosp Disaster Med, 19, pp. 318-325; Husum, H., Gilbert, M., Wisborg, T., Van Heng, Y., Murad, M., Rural prehospital trauma systems improve trauma outcome in low-income countries: A prospective study from North Iraq and Cambodia (2003) J Trauma, 54, pp. 1188-1196; Chomba, E., McClure, E.M., Wright, L.L., Carlo, W.A., Chakraborty, H., Harris, H., Effect of WHO newborn care training on neonatal mortality by education (2008) Ambul Pediatr, 8, pp. 300-304; Zhu, X.Y., Fang, H.Q., Zeng, S.P., Li, Y.M., Lin, H.L., Shi, S.Z., The impact of the Neonatal Resuscitation Program Guidelines (NRPG) on the neonatal mortality in a hospital in Zhuhai China (1997) Singapore Med J, 38, pp. 485-487; Moretti, M.A., Cesar, L.A., Nusbacher, A., Kern, K.B., Timerman, S., Ramires, J.A., Advanced cardiac life support training improves long-term survival from in-hospital cardiac arrest (2007) Resuscitation, 72, pp. 458-465; Carlo, W.A., Goudar, S.S., Jehan, I., Chomba, E., Tshefu, A., Garces, A., Parida, S., Wright, L.L., Newborn-care training and perinatal mortality in developing countries (2010) N Engl J Med, 362, pp. 614-623; Smith, M.K., Ross, C., Teaching cardiopulmonary resuscitation in a developing country: Using Nicaragua as a model (1997) Crit Care Nurs Q, 20, pp. 15-21; Tennant, C., Resuscitation training in Uganda (2000) Emerg Nurse, 8, pp. 10-14; Young, S., Hutchinson, A., Nguyen, V.T., Le, T.H., Nguyen, D.V., Vo, T.K., Teaching paediatric resuscitation skills in a developing country: Introduction of the Advanced Paediatric Life Support course into Vietnam (2008) Emerg Med Australas, 20, pp. 271-275; Zafar, S., Hafeez, A., Qureshi, F., Arshad, N., Southall, D., Structured training in the management of emergencies in mothers, babies and children in a poorly resourced health system: Logbooks to document skill use (2009) Resuscitation, 80, pp. 449-452.

974. Blanke P, Bulla S, Baumann T, Siepe M, Winterer JT, Euringer W, et al. Thoracic aorta: Prospective electrocardiographically triggered CT angiography with dual-source CT - Feasibility, image quality, and dose reduction. Radiology. 2010;255(1):207-17. doi: 10.1148/radiol.09090860.

975. Bloomfield J, Roberts J, While A. The effect of computer-assisted learning versus conventional teaching methods on the acquisition and retention of handwashing theory and skills in pre-qualification nursing students: A randomised controlled trial. International Journal of Nursing Studies. 2010;47(3):287-94. doi: http://doi.org/10.1016/j.ijnurstu.2009.08.003.

976. Burunkaya M. Design and construction of a low cost dsPIC controller based repetitive transcranial magnetic stimulator (rTMS). J Med Syst. 2010;34(1):15-24. doi: 10.1007/s10916-008-9211-3

10.1007/s10916-008-9140-1; Davey, K.R., Riehl, M., (2004) Suppressing the Surface Field during Transcranial Magnetic Stimulation, , http://www.utexas.edu/research/cem/images/suppressing_surface_field.pdf, TBME-00442-2004.R2, March 18; Kammer, T., Beck, S., Thielscher, A., Laubis-Herrmann, U., Topka, H., Motor thresholds in humans: A transcranial magnetic stimulation study comparing different pulse waveforms, current directions and stimulator types (2001) Clinical Neurophysiology, 112 (2), pp. 250-258. , DOI 10.1016/S1388-2457(00)00513-7, PII S1388245700005137; Jali-Nous, R., Guide to magnetic stimulation (1998) The Magnetism Comp. U.K, pp. 1-2. , 5-6, 11; Wolf, E.W., Walker, C.F., Design and practical considerations in the construction of magnetic induction stimulators (1991) Annual Int. Conf. of the IEEE. Eng. in Med. Biol. Soc., 13 (2), pp. 857-858. , Neuromuscular Systems 25.2-7; Erickson, R.W., (2001) Fundamentals of Power Electronics, pp. 44-48. , Kluwer Academic Publisher, ISBN 0-7923-7270-0, University of Colarado Boulder 50-75, 88, 91, 95-97; Clarke, R., (2007) An Introduction to the Air Cored Coil, , http://info.ee.surrey.ac.uk/Workshop/advice/coils/air_coils.html. Accessed2June2008; Malmivuo, J., Plonsey, R., (1995) Bioelectromagnetism; Principles and Applications of Bioelectric and Biomagnetic Fields, Chapter 22: Magnetic Stimulation of Neural Tissue, pp. 375-380. , Oxford University Press New York; Sack, A.T., Linden, D.E.J., Combining transcranial magnetic stimulation and functional imaging in cognitive brain research: Possibilities and limitations (2003) Brain Research Reviews, 43 (1), pp. 41-56. , DOI 10.1016/S0165-0173(03)00191-7; Iramina, K., Maeno, T., Kowatari, Y., Ueno, S., Effects of transcranial magnetic stimulation on EEG activity (2002) IEEE Transactions on Magnetics, 38, pp. 3347-3349. , DOI 10.1109/TMAG.2002.802309; Microchip Tech.Inc., dsPIC30F3014/4013, 3-15, 57-62, 2006MicroC for dsPIC IDE C Compiler Manual (2007) Mikro Elektronika, pp. 1-2. , 161-162; NTE Electronics Inc: NTE5351 Silicon Controlled Rectifier (SCR) for High Speed Switching. NTE Electronics Inc, Bloomfield, 2008SGS-THOMSON Microelectronics., TXN/TYN 058(G)-TXN/TYN 1008 (G) SCR. pp:5, Italy, April 1995Silicon Power, S.D.M.: 170HK2 MTO Thyristor, , Malvern, USA, Sdm170v2.xls, 5/15/2001; Song, S.-H., Current control of 12-pulse regenerative converter for high current magnetic power supply (2006) Electric Power Components and Systems, 34 (8), pp. 917-926. , DOI 10.1080/15325000600561621, PII T31170817276370; Chan, T.K., Morcos, M.M., Switching performance of semiconductor devices in a cascode switch (2002) Electric Power Components and Systems, 30 (2), pp. 167-198. , DOI 10.1080/153250002753427842; Microchip, dsPIC30F Digital Signal Controllers: Blending A 16-Bit Flash MCU with the Power of DSP, p. 6. , 2004 Microchip Tech.Inc., USA (1-20); Burunkaya, M., Guler, I., Design and construction of a microcontroller based transcranial magnetic stimulator (2008) Instrumentation Science and Technology, 36 (1), pp. 32-42. , DOI 10.1080/10739140701749831, PII 788588900; Davey, K., Riehl, M., Designing transcranial magnetic stimulation systems (2005) IEEE Transactions on Magnetics, 41 (3), pp. 1142-1148. , DOI 10.1109/TMAG.2004.843326; Al-Mutawaly, N., Bruin, H., Designing and constructing a magnetic stimulator: Theoretical and practical consideration Proceedings of 23rd Annual EMBS Int. Conf, , Istanbul, Turkey, October 25-28.

977. Cant RP, Cooper SJ. Simulation-based learning in nurse education: Systematic review. J Adv Nurs. 2010;66(1):3-15. doi: 10.1111/j.1365-2648.2009.05240.x.

978. Cardoso Martins A, Dias Costa P, Miguel Marques J, Cruz Correia R, editors. Electrocardiogram rhythm simulation in open source environment - A contribution to training in biomedical sciences. 3rd International Conference on Health Informatics, HEALTHINF 2010; 2010; Valencia.

979. Chang T, Xiao N, editors. Improved RCE neural network and its application in human-robot interaction based on hand gesture recognition. 2nd International Conference on Information Science and Engineering, ICISE2010; 2010; Hangzhou.

980. Cheung SS, Petersen SR, McLellan TM. Physiological strain and countermeasures with firefighting. Scand J Med Sci Sports. 2010;20(SUPPL. 3):103-16. doi: 10.1111/j.1600-0838.2010.01215.x.

981. Chiu CC, Hwang SY, Cook DF, Luh YP. Process disturbance identification through integration of spatiotemporal ICA and CART approach. Neural Comput Appl. 2010;19(5):677-89. doi: 10.1007/s00521-009-0324-5.

982. Cooper S, Cant R, Porter J, Sellick K, Somers G, Kinsman L, et al. Rating medical emergency teamwork performance: Development of the Team Emergency Assessment Measure (TEAM). Resuscitation. 2010;81(4):446-52. doi: 10.1016/j.resuscitation.2009.11.027.

983. Costa Jr JD, Ferreira DD, Nadal J, Miranda De Sá AMFL, editors. Reducing electrocardiographic artifacts from electromyogram signals with independent component analysis. 2010 32nd Annual International Conference of the IEEE Engineering in Medicine and Biology Society, EMBC'10; 2010; Buenos Aires.

984. Creighton DW, Shrier I, Shultz R, Meeuwisse WH, Matheson GO. Return-to-play in sport: A decision-based model. Clin J Sport Med. 2010;20(5):379-85. doi: 10.1097/JSM.0b013e3181f3c0fe.

985. Deakin CD, Morrison LJ, Morley PT, Callaway CW, Kerber RE, Kronick SL, et al. Part 8: Advanced life support: 2010 International consensus on cardiopulmonary resuscitation and emergency cardiovascular care science with treatment recommendations. Resuscitation. 2010;81(1 SUPPL.1):e93-e174. doi: 10.1016/j.resuscitation.2010.08.027.

986. Deakin CD, Nolan JP, Soar J, Sunde K, Koster RW, Smith GB, et al. European Resuscitation Council Guidelines for Resuscitation 2010 Section 4. Adult advanced life support. Resuscitation. 2010;81(10):1305-52. doi: 10.1016/j.resuscitation.2010.08.017

10.1016/j.resuscitation.2010.08.027Manz, M., Pfeiffer, D., Jung, W., Lueritz, B., Intravenous treatment with magnesium in recurrent persistent ventricular tachycardia (1991) New Trends Arrhythmias, 7, pp. 437-442; Tzivoni, D., Banai, S., Schuger, C., Treatment of torsade de pointes with magnesium sulfate (1988) Circulation, 77, pp. 392-397; Delacretaz, E., Clinical practice. Supraventricular tachycardia (2006) N Engl J Med, 354, pp. 1039-1051; DiMarco, J.P., Miles, W., Akhtar, M., Adenosine for paroxysmal supraventricular tachycardia: dose ranging and comparison with verapamil: assessment in placebo-controlled, multicenter trials. The Adenosine for PSVT Study Group [published correction appears in Ann Intern Med. 1990; 113:996] (1990) Ann Intern Med, 113, pp. 104-110; Fuster, V., Ryden, L.E., Cannom, D.S., ACC/AHA/ESC 2006 guidelines for the management of patients with atrial fibrillation: a report of the American College of Cardiology/American Heart Association Task Force on Practice Guidelines and the European Society of Cardiology Committee for Practice Guidelines (Writing Committee to Revise the 2001 Guidelines for the Management of Patients With Atrial Fibrillation): developed in collaboration (2006) Circulation, 114, pp. e257-e354; Sticherling, C., Tada, H., Hsu, W., Effects of diltiazem and esmolol on cycle length and spontaneous conversion of atrial fibrillation (2002) J Cardiovasc Pharmacol Ther, 7, pp. 81-88; Shettigar, U.R., Toole, J.G., Appunn, D.O., Combined use of esmolol and digoxin in the acute treatment of atrial fibrillation or flutter (1993) Am Heart J, 126, pp. 368-374; Demircan, C., Cikriklar, H.I., Engindeniz, Z., Comparison of the effectiveness of intravenous diltiazem and metoprolol in the management of rapid ventricular rate in atrial fibrillation (2005) Emerg Med J, 22, pp. 411-414; Wattanasuwan, N., Khan, I.A., Mehta, N.J., Acute ventricular rate control in atrial fibrillation: IV combination of diltiazem and digoxin vs. IV diltiazem alone (2001) Chest, 119, pp. 502-506; Davey, M.J., Teubner, D., A randomized controlled trial of magnesium sulfate, in addition to usual care, for rate control in atrial fibrillation (2005) Ann Emerg Med, 45, pp. 347-353; Chiladakis, J.A., Stathopoulos, C., Davlouros, P., Manolis, A.S., Intravenous magnesium sulfate versus diltiazem in paroxysmal atrial fibrillation (2001) Int J Cardiol, 79, pp. 287-291; Dauchot, P., Gravenstein, J.S., Effects of atropine on the electrocardiogram in different age groups (1971) Clin Pharmacol Ther, 12, pp. 274-280; Chamberlain, D.A., Turner, P., Sneddon, J.M., Effects of atropine on heart-rate in healthy man (1967) Lancet, 2, pp. 12-15; Bernheim, A., Fatio, R., Kiowski, W., Weilenmann, D., Rickli, H., Rocca, H.P., Atropine often results in complete atrioventricular block or sinus arrest after cardiac transplantation: an unpredictable and dose-independent phenomenon (2004) Transplantation, 77, pp. 1181-1185; Klumbies, A., Paliege, R., Volkmann, H., Mechanical emergency stimulation in asystole and extreme bradycardia (1988) Z Gesamte Inn Med, 43, pp. 348-352; Zeh, E., Rahner, E., The manual extrathoracal stimulation of the heart. Technique and effect of the precordial thump (author's transl) (1978) Z Kardiol, 67, pp. 299-304; Chan, L., Reid, C., Taylor, B., Effect of three emergency pacing modalities on cardiac output in cardiac arrest due to ventricular asystole (2002) Resuscitation, 52, pp. 117-119; Camm, A.J., Garratt, C.J., Adenosine and supraventricular tachycardia (1991) N Engl J Med, 325, pp. 1621-1629; Wang, H.E., O'Connor, R.E., Megargel, R.E., The use of diltiazem for treating rapid atrial fibrillation in the out-of-hospital setting (2001) Ann Emerg Med, 37, pp. 38-45; Martinez-Marcos, F.J., Garcia-Garmendia, J.L., Ortega-Carpio, A., Fernandez-Gomez, J.M., Santos, J.M., Camacho, C., Comparison of intravenous flecainide, propafenone, and amiodarone for conversion of acute atrial fibrillation to sinus rhythm (2000) Am J Cardiol, 86, pp. 950-953; Kalus, J.S., Spencer, A.P., Tsikouris, J.P., Impact of prophylactic i.v. magnesium on the efficacy of ibutilide for conversion of atrial fibrillation or flutter (2003) Am J Health Syst Pharm, 60, pp. 2308-2312; Nolan, J.P., Neumar, R.W., Adrie, C., Post-cardiac arrest syndrome: epidemiology, pathophysiology, treatment, and prognostication. A Scientific Statement from the International Liaison Committee on Resuscitation; the American Heart Association Emergency Cardiovascular Care Committee; the Council on Cardiovascular Surgery and Anesthesia; the Council on Cardiopulmonary, Perioperative, and Critical Care; the Council on Clinical Cardiolog (2008) Resuscitation, 79, pp. 350-379; Sunde, K., Pytte, M., Jacobsen, D., Implementation of a standardised treatment protocol for post resuscitation care after out-of-hospital cardiac arrest (2007) Resuscitation, 73, pp. 29-39; Gaieski, D.F., Band, R.A., Abella, B.S., Early goal-directed hemodynamic optimization combined with therapeutic hypothermia in comatose survivors of out-of-hospital cardiac arrest (2009) Resuscitation, 80, pp. 418-424; Carr, B.G., Goyal, M., Band, R.A., A national analysis of the relationship between hospital factors and post-cardiac arrest mortality (2009) Intensive Care Med, 35, pp. 505-511; Oddo, M., Schaller, M.D., Feihl, F., Ribordy, V., Liaudet, L., From evidence to clinical practice: effective implementation of therapeutic hypothermia to improve patient outcome after cardiac arrest (2006) Crit Care Med, 34, pp. 1865-1873; Knafelj, R., Radsel, P., Ploj, T., Noc, M., Primary percutaneous coronary intervention and mild induced hypothermia in comatose survivors of ventricular fibrillation with ST-elevation acute myocardial infarction (2007) Resuscitation, 74, pp. 227-234; Nolan, J.P., Laver, S.R., Welch, C.A., Harrison, D.A., Gupta, V., Rowan, K., Outcome following admission to UK intensive care units after cardiac arrest: a secondary analysis of the ICNARC Case Mix Programme Database (2007) Anaesthesia, 62, pp. 1207-1216; Keenan, S.P., Dodek, P., Martin, C., Priestap, F., Norena, M., Wong, H., Variation in length of intensive care unit stay after cardiac arrest: where you are is as important as who you are (2007) Crit Care Med, 35, pp. 836-841; Carr, B.G., Kahn, J.M., Merchant, R.M., Kramer, A.A., Neumar, R.W., Inter-hospital variability in post-cardiac arrest mortality (2009) Resuscitation, 80, pp. 30-34; Niskanen, M., Reinikainen, M., Kurola, J., Outcome from intensive care after cardiac arrest: comparison between two patient samples treated in 1986-87 and 1999-2001 in Finnish ICUs (2007) Acta Anaesthesiol Scand, 51, pp. 151-157; Hovdenes, J., Laake, J.H., Aaberge, L., Haugaa, H., Bugge, J.F., Therapeutic hypothermia after out-of-hospital cardiac arrest: experiences with patients treated with percutaneous coronary intervention and cardiogenic shock (2007) Acta Anaesthesiol Scand, 51, pp. 137-142; Soar, J., Mancini, M.E., Bhanji, F., (2010), International consensus on cardiopulmonary resuscitation and emergency cardiovascular care science with treatment recommendations. Part 12: education, implementation, and teams. Resuscitation; , in press. doi:10.1016/j.resuscitation.2010.08.030Laver, S., Farrow, C., Turner, D., Nolan, J., Mode of death after admission to an intensive care unit following cardiac arrest (2004) Intensive Care Med, 30, pp. 2126-2128; Laurent, I., Monchi, M., Chiche, J.D., Reversible myocardial dysfunction in survivors of out-of-hospital cardiac arrest (2002) J Am Coll Cardiol, 40, pp. 2110-2116; Ruiz-Bailen, M., Aguayo de Hoyos, E., Ruiz-Navarro, S., Reversible myocardial dysfunction after cardiopulmonary resuscitation (2005) Resuscitation, 66, pp. 175-181; Cerchiari, E.L., Safar, P., Klein, E., Diven, W., Visceral, hematologic and bacteriologic changes and neurologic outcome after cardiac arrest in dogs. The visceral post-resuscitation syndrome (1993) Resuscitation, 25, pp. 119-136; Adrie, C., Monchi, M., Laurent, I., Coagulopathy after successful cardiopulmonary resuscitation following cardiac arrest: implication of the protein C anticoagulant pathway (2005) J Am Coll Cardiol, 46, pp. 21-28; Adrie, C., Adib-Conquy, M., Laurent, I., Successful cardiopulmonary resuscitation after cardiac arrest as a " sepsis-like" syndrome (2002) Circulation, 106, pp. 562-568; Adrie, C., Laurent, I., Monchi, M., Cariou, A., Dhainaou, J.F., Spaulding, C., Postresuscitation disease after cardiac arrest: a sepsis-like syndrome? (2004) Curr Opin Crit Care, 10, pp. 208-212; Zwemer, C.F., Whitesall, S.E., D'Alecy, L.G., Cardiopulmonary-cerebral resuscitation with 100% oxygen exacerbates neurological dysfunction following nine minutes of normothermic cardiac arrest in dogs (1994) Resuscitation, 27, pp. 159-170; Richards, E.M., Fiskum, G., Rosenthal, R.E., Hopkins, I., McKenna, M.C., Hyperoxic reperfusion after global ischemia decreases hippocampal energy metabolism (2007) Stroke, 38, pp. 1578-1584; Vereczki, V., Martin, E., Rosenthal, R.E., Hof, P.R., Hoffman, G.E., Fiskum, G., Normoxic resuscitation after cardiac arrest protects against hippocampal oxidative stress, metabolic dysfunction, and neuronal death (2006) J Cereb Blood Flow Metab, 26, pp. 821-835; Liu, Y., Rosenthal, R.E., Haywood, Y., Miljkovic-Lolic, M., Vanderhoek, J.Y., Fiskum, G., Normoxic ventilation after cardiac arrest reduces oxidation of brain lipids and improves neurological outcome (1998) Stroke, 29, pp. 1679-1686; Menon, D.K., Coles, J.P., Gupta, A.K., Diffusion limited oxygen delivery following head injury (2004) Crit Care Med, 32, pp. 1384-1390; Buunk, G., van der Hoeven, J.G., Meinders, A.E., Cerebrovascular reactivity in comatose patients resuscitated from a cardiac arrest (1997) Stroke, 28, pp. 1569-1573; Buunk, G., van der Hoeven, J.G., Meinders, A.E., A comparison of near-infrared spectroscopy and jugular bulb oximetry in comatose patients resuscitated from a cardiac arrest (1998) Anaesthesia, 53, pp. 13-19; Roine, R.O., Launes, J., Nikkinen, P., Lindroth, L., Kaste, M., Regional cerebral blood flow after human cardiac arrest. A hexamethylpropyleneamine oxime single photon emission computed tomographic study (1991) Arch Neurol, 48, pp. 625-629; Beckstead, J.E., Tweed, W.A., Lee, J., MacKeen, W.L., Cerebral blood flow and metabolism in man following cardiac arrest (1978) Stroke, 9, pp. 569-573; Zheng, Z.J., Croft, J.B., Giles, W.H., Mensah, G.A., Sudden cardiac death in the United States, 1989 to 1998 (2001) Circulation, 104, pp. 2158-2163; Pell, J.P., Sirel, J.M., Marsden, A.K., Ford, I., Walker, N.L., Cobbe, S.M., Presentation, management, and outcome of out of hospital cardiopulmonary arrest: comparison by underlying aetiology (2003) Heart, 89, pp. 839-842; Zipes, D.P., Wellens, H.J., Sudden cardiac death (1998) Circulation, 98, pp. 2334-2351; Spaulding, C.M., Joly, L.M., Rosenberg, A., Immediate coronary angiography in survivors of out-of-hospital cardiac arrest (1997) N Engl J Med, 336, pp. 1629-1633; Bendz, B., Eritsland, J., Nakstad, A.R., Long-term prognosis after out-of-hospital cardiac arrest and primary percutaneous coronary intervention (2004) Resuscitation, 63, pp. 49-53; Keelan, P.C., Bunch, T.J., White, R.D., Packer, D.L., Holmes, D.R., Early direct coronary angioplasty in survivors of out-of-hospital cardiac arrest (2003) Am J Cardiol, 91, pp. 1461-1463. , A6; Quintero-Moran, B., Moreno, R., Villarreal, S., Percutaneous coronary intervention for cardiac arrest secondary to ST-elevation acute myocardial infarction. Influence of immediate paramedical/medical assistance on clinical outcome (2006) J Invasive Cardiol, 18, pp. 269-272; Garot, P., Lefevre, T., Eltchaninoff, H., Six-month outcome of emergency percutaneous coronary intervention in resuscitated patients after cardiac arrest complicating ST-elevation myocardial infarction (2007) Circulation, 115, pp. 1354-1362; Nagao, K., Hayashi, N., Kanmatsuse, K., Cardiopulmonary cerebral resuscitation using emergency cardiopulmonary bypass, coronary reperfusion therapy and mild hypothermia in patients with cardiac arrest outside the hospital (2000) J Am Coll Cardiol, 36, pp. 776-783; Nielsen, N., Hovdenes, J., Nilsson, F., Outcome, timing and adverse events in therapeutic hypothermia after out-of-hospital cardiac arrest (2009) Acta Anaesthesiol Scand, 53, pp. 926-934; Wolfrum, S., Pierau, C., Radke, P.W., Schunkert, H., Kurowski, V., Mild therapeutic hypothermia in patients after out-of-hospital cardiac arrest due to acute ST-segment elevation myocardial infarction undergoing immediate percutaneous coronary intervention (2008) Crit Care Med, 36, pp. 1780-1786; Rivers, E., Nguyen, B., Havstad, S., Early goal-directed therapy in the treatment of severe sepsis and septic shock (2001) N Engl J Med, 345, pp. 1368-1377; Mullner, M., Sterz, F., Binder, M., Arterial blood pressure after human cardiac arrest and neurological recovery (1996) Stroke, 27, pp. 59-62; Trzeciak, S., Jones, A.E., Kilgannon, J.H., Significance of arterial hypotension after resuscitation from cardiac arrest (2009) Crit Care Med, 37, pp. 2895-2903; Bernard, S.A., Gray, T.W., Buist, M.D., Treatment of comatose survivors of out-of-hospital cardiac arrest with induced hypothermia (2002) N Engl J Med, 346, pp. 557-563; Angelos, M.G., Ward, K.R., Hobson, J., Beckley, P.D., Organ blood flow following cardiac arrest in a swine low-flow cardiopulmonary bypass model (1994) Resuscitation, 27, pp. 245-254; Sakabe, T., Tateishi, A., Miyauchi, Y., Intracranial pressure following cardiopulmonary resuscitation (1987) Intensive Care Med, 13, pp. 256-259; Morimoto, Y., Kemmotsu, O., Kitami, K., Matsubara, I., Tedo, I., Acute brain swelling after out-of-hospital cardiac arrest: pathogenesis and outcome (1993) Crit Care Med, 21, pp. 104-110; Nishizawa, H., Kudoh, I., Cerebral autoregulation is impaired in patients resuscitated after cardiac arrest (1996) Acta Anaesthesiol Scand, 40, pp. 1149-1153; Sundgreen, C., Larsen, F.S., Herzog, T.M., Knudsen, G.M., Boesgaard, S., Aldershvile, J., Autoregulation of cerebral blood flow in patients resuscitated from cardiac arrest (2001) Stroke, 32, pp. 128-132; Ely, E.W., Truman, B., Shintani, A., Monitoring sedation status over time in ICU patients: reliability and validity of the Richmond Agitation-Sedation Scale (RASS) (2003) JAMA, 289, pp. 2983-2991; De Jonghe, B., Cook, D., Appere-De-Vecchi, C., Guyatt, G., Meade, M., Outin, H., Using and understanding sedation scoring systems: a systematic review (2000) Intensive Care Med, 26, pp. 275-285; Snyder, B.D., Hauser, W.A., Loewenson, R.B., Leppik, I.E., Ramirez-Lassepas, M., Gumnit, R.J., Neurologic prognosis after cardiopulmonary arrest. III: seizure activity (1980) Neurology, 30, pp. 1292-1297; Levy, D.E., Caronna, J.J., Singer, B.H., Lapinski, R.H., Frydman, H., Plum, F., Predicting outcome from hypoxic-ischemic coma (1985) JAMA, 253, pp. 1420-1426; Krumholz, A., Stern, B.J., Weiss, H.D., Outcome from coma after cardiopulmonary resuscitation: relation to seizures and myoclonus (1988) Neurology, 38, pp. 401-405; Zandbergen, E.G., Hijdra, A., Koelman, J.H., Prediction of poor outcome within the first 3 days of postanoxic coma (2006) Neurology, 66, pp. 62-68; Ingvar, M., Cerebral blood flow and metabolic rate during seizures. Relationship to epileptic brain damage (1986) Ann NY Acad Sci, 462, pp. 194-206; Caviness, J.N., Brown, P., Myoclonus: current concepts and recent advances (2004) Lancet Neurol, 3, pp. 598-607; Losert, H., Sterz, F., Roine, R.O., Strict normoglycaemic blood glucose levels in the therapeutic management of patients within 12h after cardiac arrest might not be necessary (2007) Resuscitation; Skrifvars, M.B., Saarinen, K., Ikola, K., Kuisma, M., Improved survival after in-hospital cardiac arrest outside critical care areas (2005) Acta Anaesthesiol Scand, 49, pp. 1534-1539; van den Berghe, G., Wouters, P., Weekers, F., Intensive insulin therapy in the critically ill patients (2001) N Engl J Med, 345, pp. 1359-1367; Van den Berghe, G., Wilmer, A., Hermans, G., Intensive insulin therapy in the medical ICU (2006) N Engl J Med, 354, pp. 449-461; Oksanen, T., Skrifvars, M.B., Varpula, T., Strict versus moderate glucose control after resuscitation from ventricular fibrillation (2007) Intensive Care Med, 33, pp. 2093-2100; Finfer, S., Chittock, D.R., Su, S.Y., Intensive versus conventional glucose control in critically ill patients (2009) N Engl J Med, 360, pp. 1283-1297; Preiser, J.C., Devos, P., Ruiz-Santana, S., A prospective randomised multi-centre controlled trial on tight glucose control by intensive insulin therapy in adult intensive care units: the Glucontrol study (2009) Intensive Care Med, 35, pp. 1738-1748; Griesdale, D.E., de Souza, R.J., van Dam, R.M., Intensive insulin therapy and mortality among critically ill patients: a meta-analysis including NICE-SUGAR study data (2009) CMAJ, 180, pp. 821-827; Wiener, R.S., Wiener, D.C., Larson, R.J., Benefits and risks of tight glucose control in critically ill adults: a meta-analysis (2008) JAMA, 300, pp. 933-944; Krinsley, J.S., Grover, A., Severe hypoglycemia in critically ill patients: risk factors and outcomes (2007) Crit Care Med, 35, pp. 2262-2267; Meyfroidt, G., Keenan, D.M., Wang, X., Wouters, P.J., Veldhuis, J.D., Van den Berghe, G., Dynamic characteristics of blood glucose time series during the course of critical illness: effects of intensive insulin therapy and relative association with mortality (2010) Crit Care Med, 38, pp. 1021-1029; Padkin, A., Glucose control after cardiac arrest (2009) Resuscitation, 80, pp. 611-612; Takino, M., Okada, Y., Hyperthermia following cardiopulmonary resuscitation (1991) Intensive Care Med, 17, pp. 419-420; Hickey, R.W., Kochanek, P.M., Ferimer, H., Alexander, H.L., Garman, R.H., Graham, S.H., Induced hyperthermia exacerbates neurologic neuronal histologic damage after asphyxial cardiac arrest in rats (2003) Crit Care Med, 31, pp. 531-535; Takasu, A., Saitoh, D., Kaneko, N., Sakamoto, T., Okada, Y., Hyperthermia: is it an ominous sign after cardiac arrest? (2001) Resuscitation, 49, pp. 273-277; Zeiner, A., Holzer, M., Sterz, F., Hyperthermia after cardiac arrest is associated with an unfavorable neurologic outcome (2001) Arch Intern Med, 161, pp. 2007-2012; Hickey, R.W., Kochanek, P.M., Ferimer, H., Graham, S.H., Safar, P., Hypothermia and hyperthermia in children after resuscitation from cardiac arrest (2000) Pediatrics, 106, pp. 118-122; Diringer, M.N., Reaven, N.L., Funk, S.E., Uman, G.C., Elevated body temperature independently contributes to increased length of stay in neurologic intensive care unit patients (2004) Crit Care Med, 32, pp. 1489-1495; Gunn, A.J., Thoresen, M., Hypothermic neuroprotection (2006) NeuroRx, 3, pp. 154-169; Froehler, M.T., Geocadin, R.G., Hypothermia for neuroprotection after cardiac arrest: mechanisms, clinical trials and patient care (2007) J Neurol Sci, 261, pp. 118-126; McCullough, J.N., Zhang, N., Reich, D.L., Cerebral metabolic suppression during hypothermic circulatory arrest in humans (1999) Ann Thorac Surg, 67, pp. 1895-1899. , [discussion 919-21]; Mild therapeutic hypothermia to improve the neurologic outcome after cardiac arrest (2002) N Engl J Med, 346, pp. 549-556; Belliard, G., Catez, E., Charron, C., Efficacy of therapeutic hypothermia after out-of-hospital cardiac arrest due to ventricular fibrillation (2007) Resuscitation, 75, pp. 252-259; Castrejon, S., Cortes, M., Salto, M.L., Improved prognosis after using mild hypothermia to treat cardiorespiratory arrest due to a cardiac cause: comparison with a control group (2009) Rev Esp Cardiol, 62, pp. 733-741; Bro-Jeppesen, J., Kjaergaard, J., Horsted, T.I., The impact of therapeutic hypothermia on neurological function and quality of life after cardiac arrest (2009) Resuscitation, 80, pp. 171-176; Hachimi-Idrissi, S., Corne, L., Ebinger, G., Michotte, Y., Huyghens, L., Mild hypothermia induced by a helmet device: a clinical feasibility study (2001) Resuscitation, 51, pp. 275-281; Bernard, S.A., Jones, B.M., Horne, M.K., Clinical trial of induced hypothermia in comatose survivors of out-of-hospital cardiac arrest (1997) Ann Emerg Med, 30, pp. 146-153; Busch, M., Soreide, E., Lossius, H.M., Lexow, K., Dickstein, K., Rapid implementation of therapeutic hypothermia in comatose out-of-hospital cardiac arrest survivors (2006) Acta Anaesthesiol Scand, 50, pp. 1277-1283; Storm, C., Steffen, I., Schefold, J.C., Mild therapeutic hypothermia shortens intensive care unit stay of survivors after out-of-hospital cardiac arrest compared to historical controls (2008) Crit Care, 12, pp. R78; Don, C.W., Longstreth, W.T., Maynard, C., Active surface cooling protocol to induce mild therapeutic hypothermia after out-of-hospital cardiac arrest: a retrospective before-and-after comparison in a single hospital (2009) Crit Care Med, 37, pp. 3062-3069; Arrich, J., Clinical application of mild therapeutic hypothermia after cardiac arrest (2007) Crit Care Med, 35, pp. 1041-1047; Holzer, M., Mullner, M., Sterz, F., Efficacy and safety of endovascular cooling after cardiac arrest: cohort study and Bayesian approach (2006) Stroke, 37, pp. 1792-1797; Polderman, K.H., Herold, I., Therapeutic hypothermia and controlled normothermia in the intensive care unit: practical considerations, side effects, and cooling methods (2009) Crit Care Med, 37, pp. 1101-1120; Bernard, S., Buist, M., Monteiro, O., Smith, K., Induced hypothermia using large volume, ice-cold intravenous fluid in comatose survivors of out-of-hospital cardiac arrest: a preliminary report (2003) Resuscitation, 56, pp. 9-13; Virkkunen, I., Yli-Hankala, A., Silfvast, T., Induction of therapeutic hypothermia after cardiac arrest in prehospital patients using ice-cold Ringer's solution: a pilot study (2004) Resuscitation, 62, pp. 299-302; Kliegel, A., Losert, H., Sterz, F., Cold simple intravenous infusions preceding special endovascular cooling for faster induction of mild hypothermia after cardiac arrest-a feasibility study (2005) Resuscitation, 64, pp. 347-351; Kliegel, A., Janata, A., Wandaller, C., Cold infusions alone are effective for induction of therapeutic hypothermia but do not keep patients cool after cardiac arrest (2007) Resuscitation, 73, pp. 46-53; Kilgannon, J.H., Roberts, B.W., Stauss, M., Use of a standardized order set for achieving target temperature in the implementation of therapeutic hypothermia after cardiac arrest: a feasibility study (2008) Acad Emerg Med, 15, pp. 499-505; Scott, B.D., Hogue, T., Fixley, M.S., Adamson, P.B., Induced hypothermia following out-of-hospital cardiac arrest; initial experience in a community hospital (2006) Clin Cardiol, 29, pp. 525-529; Kim, F., Olsufka, M., Carlbom, D., Pilot study of rapid infusion of 2 L of 4 degrees C normal saline for induction of mild hypothermia in hospitalized, comatose survivors of out-of-hospital cardiac arrest (2005) Circulation, 112, pp. 715-719; Jacobshagen, C., Pax, A., Unsold, B.W., Effects of large volume, ice-cold intravenous fluid infusion on respiratory function in cardiac arrest survivors (2009) Resuscitation, 80, pp. 1223-1228; Spiel, A.O., Kliegel, A., Janata, A., Hemostasis in cardiac arrest patients treated with mild hypothermia initiated by cold fluids (2009) Resuscitation, 80, pp. 762-765; Larsson, I.M., Wallin, E., Rubertsson, S., Cold saline infusion and ice packs alone are effective in inducing and maintaining therapeutic hypothermia after cardiac arrest (2010) Resuscitation, 81, pp. 15-19; Skulec, R., Kovarnik, T., Dostalova, G., Kolar, J., Linhart, A., Induction of mild hypothermia in cardiac arrest survivors presenting with cardiogenic shock syndrome (2008) Acta Anaesthesiol Scand, 52, pp. 188-194; Hoedemaekers, C.W., Ezzahti, M., Gerritsen, A., van der Hoeven, J.G., Comparison of cooling methods to induce and maintain normo- and hypothermia in intensive care unit patients: a prospective intervention study (2007) Crit Care, 11, pp. R91; Kim, F., Olsufka, M., Longstreth, W.T., Pilot randomized clinical trial of prehospital induction of mild hypothermia in out-of-hospital cardiac arrest patients with a rapid infusion of 4 degrees C normal saline (2007) Circulation, 115, pp. 3064-3070; Kamarainen, A., Virkkunen, I., Tenhunen, J., Yli-Hankala, A., Silfvast, T., Prehospital therapeutic hypothermia for comatose survivors of cardiac arrest: a randomized controlled trial (2009) Acta Anaesthesiol Scand, 53, pp. 900-907; Kamarainen, A., Virkkunen, I., Tenhunen, J., Yli-Hankala, A., Silfvast, T., Induction of therapeutic hypothermia during prehospital CPR using ice-cold intravenous fluid (2008) Resuscitation, 79, pp. 205-211; Hammer, L., Vitrat, F., Savary, D., Immediate prehospital hypothermia protocol in comatose survivors of out-of-hospital cardiac arrest (2009) Am J Emerg Med, 27, pp. 570-573; Aberle, J., Kluge, S., Prohl, J., Hypothermia after CPR through conduction and convection-initial experience on an ICU (2006) Intensivmed Notfallmed, 43, pp. 37-43; Feuchtl, A., Gockel, B., Lawrenz, T., Bartelsmeier, M., Stellbrink, C., Endovascular cooling improves neurological short-term outcome after prehospital cardiac arrest (2007) Intensivmedizin, 44, pp. 37-42; Fries, M., Stoppe, C., Brucken, D., Rossaint, R., Kuhlen, R., Influence of mild therapeutic hypothermia on the inflammatory response after successful resuscitation from cardiac arrest (2009) J Crit Care, 24, pp. 453-457; Benson, D.W., Williams, G.R., Spencer, F.C., Yates, A.J., The use of hypothermia after cardiac arrest (1959) Anesth Analg, 38, pp. 423-428; Yanagawa, Y., Ishihara, S., Norio, H., Preliminary clinical outcome study of mild resuscitative hypothermia after out-of-hospital cardiopulmonary arrest (1998) Resuscitation, 39, pp. 61-66; Damian, M.S., Ellenberg, D., Gildemeister, R., Coenzyme Q10 combined with mild hypothermia after cardiac arrest: a preliminary study (2004) Circulation, 110, pp. 3011-3016; Hay, A.W., Swann, D.G., Bell, K., Walsh, T.S., Cook, B., Therapeutic hypothermia in comatose patients after out-of-hospital cardiac arrest (2008) Anaesthesia, 63, pp. 15-19; Zeiner, A., Holzer, M., Sterz, F., Mild resuscitative hypothermia to improve neurological outcome after cardiac arrest. A clinical feasibility trial. Hypothermia After Cardiac Arrest (HACA) Study Group (2000) Stroke, 31, pp. 86-94; Uray, T., Malzer, R., Out-of-hospital surface cooling to induce mild hypothermia in human cardiac arrest: a feasibility trial (2008) Resuscitation, 77, pp. 331-338; Castren, M., Nordberg, P., Svensson, L., Intra-arrest transnasal evaporative cooling: a randomized, prehospital, multicenter study (PRINCE: Pre-ROSC IntraNasal Cooling Effectiveness) (2010) Circulation, 122, pp. 729-736; Felberg, R.A., Krieger, D.W., Chuang, R., Hypothermia after cardiac arrest: feasibility and safety of an external cooling protocol (2001) Circulation, 104, pp. 1799-1804; Flint, A.C., Hemphill, J.C., Bonovich, D.C., Therapeutic hypothermia after cardiac arrest: performance characteristics and safety of surface cooling with or without endovascular cooling (2007) Neurocrit Care, 7, pp. 109-118; Heard, K.J., Peberdy, M.A., Sayre, M.R., (2010), 81, pp. 9-14. , A randomized controlled trial comparing the Arctic Sun to standard cooling for induction of hypothermia after cardiac arrest. ResuscitationMerchant, R.M., Abella, B.S., Peberdy, M.A., Therapeutic hypothermia after cardiac arrest: unintentional overcooling is common using ice packs and conventional cooling blankets (2006) Crit Care Med, 34, pp. S490-S494; Haugk, M., Sterz, F., Grassberger, M., Feasibility and efficacy of a new non-invasive surface cooling device in post-resuscitation intensive care medicine (2007) Resuscitation, 75, pp. 76-81; Al-Senani, F.M., Graffagnino, C., Grotta, J.C., A prospective, multicenter pilot study to evaluate the feasibility and safety of using the CoolGard System and Icy catheter following cardiac arrest (2004) Resuscitation, 62, pp. 143-150; Pichon, N., Amiel, J.B., Francois, B., Dugard, A., Etchecopar, C., Vignon, P., Efficacy of and tolerance to mild induced hypothermia after out-of-hospital cardiac arrest using an endovascular cooling system (2007) Crit Care, 11, pp. R71; Wolff, B., Machill, K., Schumacher, D., Schulzki, I., Werner, D., Early achievement of mild therapeutic hypothermia and the neurologic outcome after cardiac arrest (2009) Int J Cardiol, 133, pp. 223-228; Nagao, K., Kikushima, K., Watanabe, K., Early induction of hypothermia during cardiac arrest improves neurological outcomes in patients with out-of-hospital cardiac arrest who undergo emergency cardiopulmonary bypass and percutaneous coronary intervention (2010) Circ J, 74, pp. 77-85; Mahmood, M.A., Zweifler, R.M., Progress in shivering control (2007) J Neurol Sci, 261, pp. 47-54; Wadhwa, A., Sengupta, P., Durrani, J., Magnesium sulphate only slightly reduces the shivering threshold in humans (2005) Br J Anaesth, 94, pp. 756-762; Kuboyama, K., Safar, P., Radovsky, A., Delay in cooling negates the beneficial effect of mild resuscitative cerebral hypothermia after cardia arrest in dogs: a prospective, randomized study (1993) Crit Care Med, 21, pp. 1348-1358; Riter, H.G., Brooks, L.A., Pretorius, A.M., Ackermann, L.W., Kerber, R.E., Intra-arrest hypothermia: both cold liquid ventilation with perfluorocarbons and cold intravenous saline rapidly achieve hypothermia, but only cold liquid ventilation improves resumption of spontaneous circulation (2009) Resuscitation, 80, pp. 561-566; Staffey, K.S., Dendi, R., Brooks, L.A., Liquid ventilation with perfluorocarbons facilitates resumption of spontaneous circulation in a swine cardiac arrest model (2008) Resuscitation, 78, pp. 77-84; Polderman, K.H., Peerdeman, S.M., Girbes, A.R., Hypophosphatemia and hypomagnesemia induced by cooling in patients with severe head injury (2001) J Neurosurg, 94, pp. 697-705; Tortorici, M.A., Kochanek, P.M., Poloyac, S.M., Effects of hypothermia on drug disposition, metabolism, and response: a focus of hypothermia-mediated alterations on the cytochrome P450 enzyme system (2007) Crit Care Med, 35, pp. 2196-2204; Randomized clinical study of thiopental loading in comatose survivors of cardiac arrest. Brain Resuscitation Clinical Trial I Study Group (1986) N Engl J Med, 314, pp. 397-403; Grafton, S.T., Longstreth, W.T., Steroids after cardiac arrest: a retrospective study with concurrent, nonrandomized controls (1988) Neurology, 38, pp. 1315-1316; Mentzelopoulos, S.D., Zakynthinos, S.G., Tzoufi, M., Vasopressin, epinephrine, and corticosteroids for in-hospital cardiac arrest (2009) Arch Intern Med, 169, pp. 15-24; Gueugniaud, P.Y., Gaussorgues, P., Garcia-Darennes, F., Early effects of nimodipine on intracranial and cerebral perfusion pressures in cerebral anoxia after out-of-hospital cardiac arrest (1990) Resuscitation, 20, pp. 203-212; Roine, R.O., Kaste, M., Kinnunen, A., Nikki, P., Sarna, S., Kajaste, S., Nimodipine after resuscitation from out-of-hospital ventricular fibrillation: a placebo-controlled, double-blind, randomized trial (1990) JAMA, 264, pp. 3171-3177; A randomized clinical study of a calcium-entry blocker (lidoflazine) in the treatment of comatose survivors of cardiac arrest. Brain Resuscitation Clinical Trial II Study Group (1991) N Engl J Med, 324, pp. 1225-1231; Laurent, I., Adrie, C., Vinsonneau, C., High-volume hemofiltration after out-of-hospital cardiac arrest: a randomized study (2005) J Am Coll Cardiol, 46, pp. 432-437; Edgren, E., Hedstrand, U., Nordin, M., Rydin, E., Ronquist, G., Prediction of outcome after cardiac arrest (1987) Crit Care Med, 15, pp. 820-825; Young, G.B., Doig, G., Ragazzoni, A., Anoxic-ischemic encephalopathy: clinical and electrophysiological associations with outcome (2005) Neurocrit Care, 2, pp. 159-164; Al Thenayan, E., Savard, M., Sharpe, M., Norton, L., Young, B., Predictors of poor neurologic outcome after induced mild hypothermia following cardiac arrest (2008) Neurology, 71, pp. 1535-1537; Wijdicks, E.F., Parisi, J.E., Sharbrough, F.W., Prognostic value of myoclonus status in comatose survivors of cardiac arrest (1994) Ann Neurol, 35, pp. 239-243; Thomke, F., Marx, J.J., Sauer, O., Observations on comatose survivors of cardiopulmonary resuscitation with generalized myoclonus (2005) BMC Neurol, 5, p. 14; Arnoldus, E.P., Lammers, G.J., Postanoxic coma: good recovery despite myoclonus status (1995) Ann Neurol, 38, pp. 697-698; Celesia, G.G., Grigg, M.M., Ross, E., Generalized status myoclonicus in acute anoxic and toxic-metabolic encephalopathies (1988) Arch Neurol, 45, pp. 781-784; Morris, H.R., Howard, R.S., Brown, P., Early myoclonic status and outcome after cardiorespiratory arrest (1998) J Neurol Neurosurg Psychiatry, 64, pp. 267-268; Datta, S., Hart, G.K., Opdam, H., Gutteridge, G., Archer, J., Post-hypoxic myoclonic status: the prognosis is not always hopeless (2009) Crit Care Resusc, 11, pp. 39-41; English, W.A., Giffin, N.J., Nolan, J.P., Myoclonus after cardiac arrest: pitfalls in diagnosis and prognosis (2009) Anaesthesia, 64, pp. 908-911; Wijdicks, E.F., Hijdra, A., Young, G.B., Bassetti, C.L., Wiebe, S., Practice parameter: prediction of outcome in comatose survivors after cardiopulmonary resuscitation (an evidence-based review): report of the Quality Standards Subcommittee of the American Academy of Neurology (2006) Neurology, 67, pp. 203-210; Zandbergen, E.G., de Haan, R.J., Hijdra, A., Systematic review of prediction of poor outcome in anoxic-ischaemic coma with biochemical markers of brain damage (2001) Intensive Care Med, 27, pp. 1661-1667; Grubb, N.R., Simpson, C., Sherwood, R., Prediction of cognitive dysfunction after resuscitation from out-of-hospital cardiac arrest using serum neuron-specific enolase and protein S-100 (2007) Heart; Martens, P., Serum neuron-specific enolase as a prognostic marker for irreversible brain damage in comatose cardiac arrest survivors (1996) Acad Emerg Med, 3, pp. 126-131; Meynaar, I.A., Straaten, H.M., van der Wetering, J., Serum neuron-specific enolase predicts outcome in post-anoxic coma: a prospective cohort study (2003) Intensive Care Med, 29, pp. 189-195; Rech, T.H., Vieira, S.R., Nagel, F., Brauner, J.S., Scalco, R., Serum neuron-specific enolase as early predictor of outcome after in-hospital cardiac arrest: a cohort study (2006) Crit Care, 10, pp. R133; Reisinger, J., Hollinger, K., Lang, W., Prediction of neurological outcome after cardiopulmonary resuscitation by serial determination of serum neuron-specific enolase (2007) Eur Heart J, 28, pp. 52-58; Schoerkhuber, W., Kittler, H., Sterz, F., Time course of serum neuron-specific enolase. A predictor of neurological outcome in patients resuscitated from cardiac arrest (1999) Stroke, 30, pp. 1598-1603; Bottiger, B.W., Mobes, S., Glatzer, R., Astroglial protein S-100 is an early and sensitive marker of hypoxic brain damage and outcome after cardiac arrest in humans (2001) Circulation, 103, pp. 2694-2698; Fogel, W., Krieger, D., Veith, M., Serum neuron-specific enolase as early predictor of outcome after cardiac arrest (1997) Crit Care Med, 25, pp. 1133-1138; Martens, P., Raabe, A., Johnsson, P., Serum S-100 and neuron-specific enolase for prediction of regaining consciousness after global cerebral ischemia (1998) Stroke, 29, pp. 2363-2366; Prohl, J., Rother, J., Kluge, S., Prediction of short-term and long-term outcomes after cardiac arrest: a prospective multivariate approach combining biochemical, clinical, electrophysiological, and neuropsychological investigations (2007) Crit Care Med, 35, pp. 1230-1237; Stelzl, T., von Bose, M.J., Hogl, B., Fuchs, H.H., Flugel, K.A., A comparison of the prognostic value of neuron-specific enolase serum levels and somatosensory evoked potentials in 13 reanimated patients (1995) Eur J Emerg Med, 2, pp. 24-27; Tiainen, M., Roine, R.O., Pettila, V., Takkunen, O., Serum neuron-specific enolase and S-100B protein in cardiac arrest patients treated with hypothermia (2003) Stroke, 34, pp. 2881-2886; Pfeifer, R., Borner, A., Krack, A., Sigusch, H.H., Surber, R., Figulla, H.R., Outcome after cardiac arrest: predictive values and limitations of the neuroproteins neuron-specific enolase and protein S-100 and the Glasgow Coma Scale (2005) Resuscitation, 65, pp. 49-55; Roine, R.O., Somer, H., Kaste, M., Viinikka, L., Karonen, S.L., Neurological outcome after out-of-hospital cardiac arrest. Prediction by cerebrospinal fluid enzyme analysis (1989) Arch Neurol, 46, pp. 753-756; Zingler, V.C., Krumm, B., Bertsch, T., Fassbender, K., Pohlmann-Eden, B., Early prediction of neurological outcome after cardiopulmonary resuscitation: a multimodal approach combining neurobiochemical and electrophysiological investigations may provide high prognostic certainty in patients after cardiac arrest (2003) Eur Neurol, 49, pp. 79-84; Rosen, H., Sunnerhagen, K.S., Herlitz, J., Blomstrand, C., Rosengren, L., Serum levels of the brain-derived proteins S-100 and NSE predict long-term outcome after cardiac arrest (2001) Resuscitation, 49, pp. 183-191; Dauberschmidt, R., Zinsmeyer, J., Mrochen, H., Meyer, M., Changes of neuron-specific enolase concentration in plasma after cardiac arrest and resuscitation (1991) Mol Chem Neuropathol, 14, pp. 237-245; Mussack, T., Biberthaler, P., Kanz, K.G., Serum S-100B and interleukin-8 as predictive markers for comparative neurologic outcome analysis of patients after cardiac arrest and severe traumatic brain injury (2002) Crit Care Med, 30, pp. 2669-2674; Fries, M., Kunz, D., Gressner, A.M., Rossaint, R., Kuhlen, R., Procalcitonin serum levels after out-of-hospital cardiac arrest (2003) Resuscitation, 59, pp. 105-109; Hachimi-Idrissi, S., Van der Auwera, M., Schiettecatte, J., Ebinger, G., Michotte, Y., Huyghens, L., S-100 protein as early predictor of regaining consciousness after out of hospital cardiac arrest (2002) Resuscitation, 53, pp. 251-257; Piazza, O., Cotena, S., Esposito, G., De Robertis, E., Tufano, R., S100B is a sensitive but not specific prognostic index in comatose patients after cardiac arrest (2005) Minerva Chir, 60, pp. 477-480; Rosen, H., Rosengren, L., Herlitz, J., Blomstrand, C., Increased serum levels of the S-100 protein are associated with hypoxic brain damage after cardiac arrest (1998) Stroke, 29, pp. 473-477; Mussack, T., Biberthaler, P., Kanz, K.G., Wiedemann, E., Gippner-Steppert, C., Jochum, M., S-100b, sE-selectin, and sP-selectin for evaluation of hypoxic brain damage in patients after cardiopulmonary resuscitation: pilot study (2001) World J Surg, 25, pp. 539-543. , [discussion 44]; Sodeck, G.H., Domanovits, H., Sterz, F., Can brain natriuretic peptide predict outcome after cardiac arrest? An observational study (2007) Resuscitation, 74, pp. 439-445; Geppert, A., Zorn, G., Delle-Karth, G., Plasma concentrations of von Willebrand factor and intracellular adhesion molecule-1 for prediction of outcome after successful cardiopulmonary resuscitation (2003) Crit Care Med, 31, pp. 805-811; Adib-Conquy, M., Monchi, M., Goulenok, C., Increased plasma levels of soluble triggering receptor expressed on myeloid cells 1 and procalcitonin after cardiac surgery and cardiac arrest without infection (2007) Shock, 28, pp. 406-410; Longstreth, W.T., Clayson, K.J., Chandler, W.L., Sumi, S.M., Cerebrospinal fluid creatine kinase activity and neurologic recovery after cardiac arrest (1984) Neurology, 34, pp. 834-837; Karkela, J., Pasanen, M., Kaukinen, S., Morsky, P., Harmoinen, A., Evaluation of hypoxic brain injury with spinal fluid enzymes, lactate, and pyruvate (1992) Crit Care Med, 20, pp. 378-386; Rothstein, T., Thomas, E., Sumi, S., Predicting outcome in hypoxic-ischemic coma. A prospective clinical and electrophysiological study (1991) Electroencephalogr Clin Neurophysiol, 79, pp. 101-107; Sherman, A.L., Tirschwell, D.L., Micklesen, P.J., Longstreth, W.T., Robinson, L.R., Somatosensory potentials. CSF creatine kinase BB activity, and awakening after cardiac arrest (2000) Neurology, 54, pp. 889-894; Longstreth, W.T., Clayson, K.J., Sumi, S.M., Cerebrospinal fluid and serum creatine kinase BB activity after out-of-hospital cardiac arrest (1981) Neurology, 31, pp. 455-458; Tirschwell, D.L., Longstreth, W.T., Rauch-Matthews, M.E., Cerebrospinal fluid creatine kinase BB isoenzyme activity and neurologic prognosis after cardiac arrest (1997) Neurology, 48, pp. 352-357; Clemmensen, P., Strandgaard, S., Rasmussen, S., Grande, P., Cerebrospinal fluid creatine kinase isoenzyme BB levels do not predict the clinical outcome in patients unconscious following cardiac resuscitation (1987) Clin Cardiol, 10, pp. 235-236; Rosen, H., Karlsson, J.E., Rosengren, L., CSF levels of neurofilament is a valuable predictor of long-term outcome after cardiac arrest (2004) J Neurol Sci, 221, pp. 19-24; Tiainen, M., Kovala, T.T., Takkunen, O.S., Roine, R.O., Somatosensory and brainstem auditory evoked potentials in cardiac arrest patients treated with hypothermia (2005) Crit Care Med, 33, pp. 1736-1740; Rossetti, A.O., Oddo, M., Liaudet, L., Kaplan, P.W., Predictors of awakening from postanoxic status epilepticus after therapeutic hypothermia (2009) Neurology, 72, pp. 744-749; Rossetti, A.O., Logroscino, G., Liaudet, L., Status epilepticus: an independent outcome predictor after cerebral anoxia (2007) Neurology, 69, pp. 255-260; Rossetti, A.O., Oddo, M., Logroscino, G., Kaplan, P.W., Prognostication after cardiac arrest and hypothermia: a prospective study (2010) Ann Neurol, 67, pp. 301-307; Oksanen, T., Tiainen, M., Skrifvars, M.B., Predictive power of serum NSE and OHCA score regarding 6-month neurologic outcome after out-of-hospital ventricular fibrillation and therapeutic hypothermia (2009) Resuscitation, 80, pp. 165-170; Rundgren, M., Karlsson, T., Nielsen, N., Cronberg, T., Johnsson, P., Friberg, H., Neuron specific enolase and S-100B as predictors of outcome after cardiac arrest and induced hypothermia (2009) Resuscitation, 80, pp. 784-789; Fieux, F., Losser, M.R., Bourgeois, E., Kidney retrieval after sudden out of hospital refractory cardiac arrest: a cohort of uncontrolled non heart beating donors (2009) Crit Care, 13, pp. R141; Kootstra, G., Statement on non-heart-beating donor programs (1995) Transplant Proc, 27, p. 2965; Fondevila, C., Hessheimer, A.J., Ruiz, A., Liver transplant using donors after unexpected cardiac death: novel preservation protocol and acceptance criteria (2007) Am J Transplant, 7, pp. 1849-1855; Morozumi, J., Sakurai, E., Matsuno, N., Successful kidney transplantation from donation after cardiac death using a load-distributing-band chest compression device during long warm ischemic time (2009) Resuscitation, 80, pp. 278-280; Perkins, G.D., Brace, S., Gates, S., Mechanical chest-compression devices: current and future roles (2010) Curr Opin Crit Care, 16, pp. 203-210; Engdahl, J., Abrahamsson, P., Bang, A., Lindqvist, J., Karlsson, T., Herlitz, J., Is hospital care of major importance for outcome after out-of-hospital cardiac arrest? Experience acquired from patients with out-of-hospital cardiac arrest resuscitated by the same Emergency Medical Service and admitted to one of two hospitals over a 16-year period in the municipality of Goteborg (2000) Resuscitation, 43, pp. 201-211; Liu, J.M., Yang, Q., Pirrallo, R.G., Klein, J.P., Aufderheide, T.P., Hospital variability of out-of-hospital cardiac arrest survival (2008) Prehosp Emerg Care, 12, pp. 339-346; Herlitz, J., Engdahl, J., Svensson, L., Angquist, K.A., Silfverstolpe, J., Holmberg, S., Major differences in 1-month survival between hospitals in Sweden among initial survivors of out-of-hospital cardiac arrest (2006) Resuscitation, 70, pp. 404-409; Callaway, C.W., Schmicker, R., Kampmeyer, M., Receiving hospital characteristics associated with survival after out-of-hospital cardiac arrest (2010) Resuscitation; Davis, D.P., Fisher, R., Aguilar, S., The feasibility of a regional cardiac arrest receiving system (2007) Resuscitation, 74, pp. 44-51; Spaite, D.W., Bobrow, B.J., Vadeboncoeur, T.F., The impact of prehospital transport interval on survival in out-of-hospital cardiac arrest: implications for regionalization of post-resuscitation care (2008) Resuscitation, 79, pp. 61-66; Spaite, D.W., Stiell, I.G., Bobrow, B.J., Effect of transport interval on out-of-hospital cardiac arrest survival in the OPALS Study: implications for triaging patients to specialized cardiac arrest centers (2009) Ann Emerg Med; Vermeer, F., Oude Ophuis, A.J., vd Berg, E.J., Prospective randomised comparison between thrombolysis, rescue PTCA, and primary PTCA in patients with extensive myocardial infarction admitted to a hospital without PTCA facilities: a safety and feasibility study (1999) Heart, 82, pp. 426-431; Widimsky, P., Groch, L., Zelizko, M., Aschermann, M., Bednar, F., Suryapranata, H., Multicentre randomized trial comparing transport to primary angioplasty vs immediate thrombolysis vs combined strategy for patients with acute myocardial infarction presenting to a community hospital without a catheterization laboratory The PRAGUE study (2000) Eur Heart J, 21, pp. 823-831; Widimsky, P., Budesinsky, T., Vorac, D., Long distance transport for primary angioplasty vs immediate thrombolysis in acute myocardial infarction. Final results of the randomized national multicentre trial--PRAGUE-2 (2003) Eur Heart J, 24, pp. 94-104; Le May, M.R., So, D.Y., Dionne, R., A citywide protocol for primary PCI in ST-segment elevation myocardial infarction (2008) N Engl J Med, 358, pp. 231-240; Abernathy, J.H., McGwin, G., Acker, J.E., Rue, L.W., Impact of a voluntary trauma system on mortality, length of stay, and cost at a level I trauma center (2002) Am Surg, 68, pp. 182-192; Clemmer, T.P., Orme, J.F., Thomas, F.O., Brooks, K.A., Outcome of critically injured patients treated at Level I trauma centers versus full-service community hospitals (1985) Crit Care Med, 13, pp. 861-863; Culica, D., Aday, L.A., Rohrer, J.E., Regionalized trauma care system in Texas: implications for redesigning trauma systems (2007) Med Sci Monit, 13, pp. SR9-18; Hannan, E.L., Farrell, L.S., Cooper, A., Henry, M., Simon, B., Simon, R., Physiologic trauma triage criteria in adult trauma patients: are they effective in saving lives by transporting patients to trauma centers? (2005) J Am Coll Surg, 200, pp. 584-592; Harrington, D.T., Connolly, M., Biffl, W.L., Majercik, S.D., Cioffi, W.G., Transfer times to definitive care facilities are too long: a consequence of an immature trauma system (2005) Ann Surg, 241, pp. 961-966. , [discussion 6-8]; Liberman, M., Mulder, D.S., Lavoie, A., Sampalis, J.S., Implementation of a trauma care system: evolution through evaluation (2004) J Trauma, 56, pp. 1330-1335; MacKenzie, E.J., Rivara, F.P., Jurkovich, G.J., A national evaluation of the effect of trauma-center care on mortality (2006) N Engl J Med, 354, pp. 366-378; Mann, N.C., Cahn, R.M., Mullins, R.J., Brand, D.M., Jurkovich, G.J., Survival among injured geriatric patients during construction of a statewide trauma system (2001) J Trauma, 50, pp. 1111-1116; Mullins, R.J., Veum-Stone, J., Hedges, J.R., Influence of a statewide trauma system on location of hospitalization and outcome of injured patients (1996) J Trauma, 40, pp. 536-545. , [discussion 45-6]; Mullins, R.J., Mann, N.C., Hedges, J.R., Worrall, W., Jurkovich, G.J., Preferential benefit of implementation of a statewide trauma system in one of two adjacent states (1998) J Trauma, 44, pp. 609-616. , [discussion 17]; Mullins, R.J., Veum-Stone, J., Helfand, M., Outcome of hospitalized injured patients after institution of a trauma system in an urban area (1994) JAMA, 271, pp. 1919-1924; Mullner, R., Goldberg, J., An evaluation of the Illinois trauma system (1978) Med Care, 16, pp. 140-151; Mullner, R., Goldberg, J., Toward an outcome-oriented medical geography: an evaluation of the Illinois trauma/emergency medical services system (1978) Soc Sci Med, 12, pp. 103-110; Nathens, A.B., Jurkovich, G.J., Rivara, F.P., Maier, R.V., Effectiveness of state trauma systems in reducing injury-related mortality: a national evaluation (2000) J Trauma, 48, pp. 25-30. , [discussion 1]; Nathens, A.B., Maier, R.V., Brundage, S.I., Jurkovich, G.J., Grossman, D.C., The effect of interfacility transfer on outcome in an urban trauma system (2003) J Trauma, 55, pp. 444-449; Nicholl, J., Turner, J., Effectiveness of a regional trauma system in reducing mortality from major trauma: before and after study (1997) BMJ, 315, pp. 1349-1354; Potoka, D.A., Schall, L.C., Gardner, M.J., Stafford, P.W., Peitzman, A.B., Ford, H.R., Impact of pediatric trauma centers on mortality in a statewide system (2000) J Trauma, 49, pp. 237-245; Sampalis, J.S., Lavoie, A., Boukas, S., Trauma center designation: initial impact on trauma-related mortality (1995) J Trauma, 39, pp. 232-237. , [discussion 7-9]; Sampalis, J.S., Denis, R., Frechette, P., Brown, R., Fleiszer, D., Mulder, D., Direct transport to tertiary trauma centers versus transfer from lower level facilities: impact on mortality and morbidity among patients with major trauma (1997) J Trauma, 43, pp. 288-95. , [discussion 95-6]; Nichol, G., Aufderheide, T.P., Eigel, B., Regional systems of care for out-of-hospital cardiac arrest: a policy statement from the American Heart Association (2010) Circulation, 121, pp. 709-729; Nichol, G., Soar, J., Regional cardiac resuscitation systems of care (2010) Curr Opin Crit Care, 16, pp. 223-230; Soar, J., Packham, S., Cardiac arrest centres make sense (2010) Resuscitation, 81, pp. 507-508.

987. Dlugosz R, Talaska T, Pedrycz W, Wojtyna R. Realization of the conscience mechanism in CMOS implementation of winner-takes-all self-organizing neural networks. IEEE Trans Neural Netw. 2010;21(6):961-71. Epub 2010/04/28. doi: 10.1109/tnn.2010.2046497. PubMed PMID: 20421180.

988. Dlugosz R, Talaśka T, Pedrycz W, Wojtyna R. Realization of the conscience mechanism in cmos implementation of winner-takes-all self-organizing neural networks. IEEE Trans Neural Networks. 2010;21(6):961-71. doi: 10.1109/TNN.2010.2046497.

989. Doyle OM, Temko A, Murray DM, Lightbody G, Marnane W, Boylan GB, editors. Predicting the neurodevelopmental outcome in newborns with hypoxic-ischaemic injury. 2010 32nd Annual International Conference of the IEEE Engineering in Medicine and Biology Society, EMBC'10; 2010; Buenos Aires.

990. Eagle TF, Gurm R, Goldberg CS, Durussel-Weston J, Kline-Rogers E, Palma-Davis L, et al. Health status and behavior among middle-school children in a midwest community: What are the underpinnings of childhood obesity? Am Heart J. 2010;160(6):1185-9. doi: 10.1016/j.ahj.2010.09.019.

991. Earls JP, Leipsic J. Cardiac Computed Tomography Technology and Dose-reduction Strategies. Radiol Clin North Am. 2010;48(4):657-74. doi: 10.1016/j.rcl.2010.04.003.

992. Evans SM, Murray A, Patrick I, Fitzgerald M, Smith S, Cameron P. Clinical handover in the trauma setting: A qualitative study of paramedics and trauma team members. Qual Saf Health Care. 2010;19(6). doi: 10.1136/qshc.2009.039073.

993. Farahabadi E, Farahabadi A, Rabbani H, Dehnavi AM, Mahjoob MP, editors. An entropy-based method for ischemia diagnosis using ECG signal in wavelet domain. 2010 IEEE 10th International Conference on Signal Processing, ICSP2010; 2010; Beijing.

994. Field JM, Hazinski MF, Sayre MR, Chameides L, Schexnayder SM, Hemphill R, et al. Part 1: Executive summary: 2010 American Heart Association Guidelines for Cardiopulmonary Resuscitation and Emergency Cardiovascular Care. Circulation. 2010;122(SUPPL. 3):S640-S56. doi: 10.1161/CIRCULATIONAHA.110.970889

10.1016/j.ajem.2010.07.001. Available at; Paradis, N., Young, G., Lemeshow, S., Brewer, J., Halperin, H., Inhomo-geneity and temporal effects in AutoPulse Assisted Prehospital International Resuscitation-an exception from consent trial terminated early (2010) Am J Emerg Med, 28, pp. 391-398; Tomte, O., Sunde, K., Lorem, T., Auestad, B., Souders, C., Jensen, J., Wik, L., Advanced life support performance with manual and mechanical chest compressions in a randomized, multicentre manikin study (2009) Resuscitation, 80, pp. 1152-1157; Axelsson, C., Nestin, J., Svensson, L., Axelsson, A.B., Herlitz, J., Clinical consequences of the introduction of mechanical chest compression in the EMS system for treatment of out-of-hospital cardiac arrest-a pilot study (2006) Resuscitation, 71, pp. 47-55; Larsen, A.I., Hjornevik, A.S., Ellingsen, C.L., Nilsen, D.W., Cardiac arrest with continuous mechanical chest compression during percutaneous coronary intervention. A report on the use of the LUCAS device (2007) Resuscitation, 75, pp. 454-459; Deakin, C.D., O'Neill, J.F., Tabor, T., Does compression-only cardiopulmo-nary resuscitation generate adequate passive ventilation during cardiac arrest? (2007) Resuscitation, 75, pp. 53-59; Bonnemeier, H., Olivecrona, G., Simonis, G., Gotberg, M., Weitz, G., Iblher, P., Gerling, I., Schunkert, H., Automated continuous chest compression for in-hospital cardiopulmonary resuscitation of patients with pulseless electrical activity: A report of five cases (2009) Int J Cardiol, 136, pp. e39-e50; Wagner, H., Terkelsen, C.J., Friberg, H., Harnek, J., Kern, K., Lassen, J.F., Olivecrona, G.K., Cardiac arrest in the catheterisation laboratory: A 5-year experience of using mechanical chest compressions to facilitate PCI during prolonged resuscitation efforts (2010) Resuscitation, 81, pp. 383-387; Larsen, M.P., Eisenberg, M.S., Cummins, R.O., Hallstrom, A.P., Predicting survival from out-of-hospital cardiac arrest: A graphic model (1993) Ann Emerg Med, 22, pp. 1652-1658; Valenzuela, T.D., Roe, D.J., Cretin, S., Spaite, D.W., Larsen, M.P., Estimating effectiveness of cardiac arrest interventions: A logistic regression survival model (1997) Circulation, 96, pp. 3308-3313; Swor, R.A., Jackson, R.E., Cynar, M., Sadler, E., Basse, E., Boji, B., Rivera-Rivera, E.J., Jacobson, R., Bystander CPR, ventricular fibrillation, and survival in witnessed, unmonitored out-of-hospital cardiac arrest (1995) Ann Emerg Med, 25, pp. 780-784; Cobb, L.A., Fahrenbruch, C.E., Walsh, T.R., Copass, M.K., Olsufka, M., Breskin, M., Hallstrom, A.P., Influence of cardiopulmonary resuscitation prior to defibrillation in patients with out-of-hospital ventricular fibrillation (1999) JAMA, 281, pp. 1182-1188; Wik, L., Hansen, T.B., Fylling, F., Steen, T., Vaagenes, P., Auestad, B.H., Steen, P.A., Delaying defibrillation to give basic cardiopulmonary resuscitation to patients with out-of-hospital ventricular fibrillation: A randomized trial (2003) JAMA, 289, pp. 1389-1395; Baker, P.W., Conway, J., Cotton, C., Ashby, D.T., Smyth, J., Woodman, R.J., Grantham, H., Defibrillation or cardiopulmonary resuscitation first for patients with out-of-hospital cardiac arrests found by paramedics to be in ventricular fibrillation? A randomised control trial (2008) Resuscitation, 79, pp. 424-431; Jacobs, I.G., Finn, J.C., Oxer, H.F., Jelinek, G.A., CPR before defibrillation in out-of-hospital cardiac arrest: A randomized trial (2005) Emerg Med Australas, 17, pp. 39-45; Morrison, L.J., Dorian, P., Long, J., Vermeulen, M., Schwartz, B., Sawadsky, B., Frank, J., Lerman, B.B., Out-of-hospital cardiac arrest rectilinear biphasic to monophasic damped sine defibrillation waveforms with advanced life support intervention trial (ORBIT) (2005) Resuscitation, 66, pp. 149-157; Schneider, T., Martens, P.R., Paschen, H., Kuisma, M., Wolcke, B., Gliner, B.E., Russell, J.K., Chamberlain, D., Multicenter, randomized, controlled trial of 150-J biphasic shocks compared with 200-to 360-J monophasic shocks in the resuscitation of out-of-hospital cardiac arrest victims. Optimized Response to Cardiac Arrest (ORCA) Investigators (2000) Circulation, 102, pp. 1780-1787; Van Alem, A.P., Chapman, F.W., Lank, P., Hart, A.A., Koster, R.W., A prospective, randomised and blinded comparison of first shock success of monophasic and biphasic waveforms in out-of-hospital cardiac arrest (2003) Resuscitation, 58, pp. 17-24; Carpenter, J., Rea, T.D., Murray, J.A., Kudenchuk, P.J., Eisenberg, M.S., Defi-brillation waveform and post-shock rhythm in out-of-hospital ventricular fibrillation cardiac arrest (2003) Resuscitation, 59, pp. 189-196; Freeman, K., Hendey, G.W., Shalit, M., Stroh, G., Biphasic defibrillation does not improve outcomes compared to monophasic defibrillation in out-of-hospital cardiac arrest (2008) Prehosp Emerg Care, 12, pp. 152-156; Gliner, B.E., White, R.D., Electrocardiographic evaluation of defibril-lation shocks delivered to out-of-hospital sudden cardiac arrest patients (1999) Resuscitation, 41, pp. 133-144; White, R.D., Hankins, D.G., Bugliosi, T.F., Seven years' experience with early defibrillation by police and paramedics in an emergency medical services system (1998) Resuscitation, 39, pp. 145-151; Cummins, R.O., Eisenberg, M.S., Bergner, L., Hallstrom, A., Hearne, T., Murray, J.A., Automatic external defibrillation: Evaluations of its role in the home and in emergency medical services (1984) Ann Emerg Med, 13 (PART2), pp. 798-801; White, R.D., Vukov, L.F., Bugliosi, T.F., Early defibrillation by police: Initial experience with measurement of critical time intervals and patient outcome (1994) Ann Emerg Med, 23, pp. 1009-1013; Mittal, S., Ayati, S., Stein, K.M., Schwartzman, D., Cavlovich, D., Tchou, P.J., Markowitz, S.M., Lerman, B.B., Transthoracic cardioversion of atrial fibrillation: Comparison of rectilinear biphasic versus damped sine wave monophasic shocks (2000) Circulation, 101, pp. 1282-1287; Page, R.L., Kerber, R.E., Russell, J.K., Trouton, T., Waktare, J., Gallik, D., Olgin, J.E., Bardy, G.H., Biphasic versus monophasic shock waveform for conversion of atrial fibrillation: The results of an international randomized, double-blind multicenter trial (2002) J Am Coll Cardiol, 39, pp. 1956-1963; Scholten, M., Szili-Torok, T., Klootwijk, P., Jordaens, L., Comparison of monophasic and biphasic shocks for transthoracic cardioversion of atrial fibrillation (2003) Heart, 89, pp. 1032-1034; Glover, B.M., Walsh, S.J., McCann, C.J., Moore, M.J., Manoharan, G., Dalzell, G.W., McAllister, A., Adgey, A.A., Biphasic energy selection for transthoracic cardioversion of atrial fibrillation. The BEST AF Trial (2008) Heart, 94, pp. 884-887; Reisinger, J., Gstrein, C., Winter, T., Zeindlhofer, E., Hollinger, K., Mori, M., Schiller, A., Siostrzonek, P., Optimization of initial energy for cardioversion of atrial tachyarrhythmias with biphasic shocks (2010) Am J Emerg Med, 28, pp. 159-165; Kerber, R.E., Martins, J.B., Kienzle, M.G., Constantin, L., Olshansky, B., Hopson, R., Charbonnier, F., Energy, current, and success in defibrillation and cardioversion: Clinical studies using an automated impedance-based method of energy adjustment (1988) Circulation, 77, pp. 1038-1046; Hedges, J.R., Syverud, S.A., Dalsey, W.C., Feero, S., Easter, R., Shultz, B., Prehospital trial of emergency transcutaneous cardiac pacing (1987) Circulation, 76, pp. 1337-1343; Barthell, E., Troiano, P., Olson, D., Stueven, H.A., Hendley, G., Prehospital external cardiac pacing: A prospective, controlled clinical trial (1988) Ann Emerg Med, 17, pp. 1221-1226; Cummins, R.O., Graves, J.R., Larsen, M.P., Hallstrom, A.P., Hearne, T.R., Ciliberti, J., Nicola, R.M., Horan, S., Out-of-hospital transcutaneous pacing by emergency medical technicians in patients with asystolic cardiac arrest (1993) N Engl J Med, 328, pp. 1377-1382; Smith, I., Monk, T.G., White, P.F., Comparison of transesophageal atrial pacing with anticholinergic drugs for the treatment of intraoperative bradycardia (1994) Anesth Analg, 78, pp. 245-252; Morrison, L.J., Long, J., Vermeulen, M., Schwartz, B., Sawadsky, B., Frank, J., Cameron, B., Dorian, P., A randomized controlled feasibility trial comparing safety and effectiveness of prehospital pacing versus conventional treatment: 'PrePACE' (2008) Resuscitation, 76, pp. 341-349; Karagounis, L., Ipsen, S.K., Jessop, M.R., Gilmore, K.M., Valenti, D.A., Clawson, J.J., Teichman, S., Anderson, J.L., Impact of field-transmitted electrocardiography on time to in-hospital thrombolytic therapy in acute myocardial infarction (1990) Am J Cardiol, 66, pp. 786-791; Kereiakes, D.J., Gibler, W.B., Martin, L.H., Pieper, K.S., Anderson, L.C., Relative importance of emergency medical system transport and the prehospital electrocardiogram on reducing hospital time delay to therapy for acute myocardial infarction: A preliminary report from the Cincinnati Heart Project (1992) Am Heart J, 123 (4 PART1), pp. 835-840; Banerjee, S., Rhoden, W.E., Fast-tracking of myocardial infarction by paramedics (1998) J R Coll Physicians Lond, 32, pp. 36-38; Melville, M.R., Gray, D., Hinchley, M., The potential impact of prehospital electrocardiography and telemetry on time to thrombolysis in a United Kingdom center (1998) Ann Noninvasive Electrocardiol, 3, pp. 327-332; Millar-Craig, M.W., Joy, A.V., Adamowicz, M., Furber, R., Thomas, B., Reduction in treatment delay by paramedic ECG diagnosis of myo-cardial infarction with direct CCU admission (1997) Heart, 78, pp. 456-461; Brainard, A.H., Raynovich, W., Tandberg, D., Bedrick, E.J., The prehospital 12-lead electrocardiogram's effect on time to initiation of reperfusion therapy: A systematic review and meta-analysis of existing literature (2005) Am J Emerg Med, 23, pp. 351-356; Morrison, L.J., Brooks, S., Sawadsky, B., McDonald, A., Verbeek, P.R., Pre-hospital 12-lead electrocardiography impact on acute myocardial infarction treatment times and mortality: A systematic review (2006) Acad Emerg Med, 13, pp. 84-89; Adams, G.L., Campbell, P.T., Adams, J.M., Strauss, D.G., Wall, K., Patterson, J., Shuping, K.B., Wagner, G.S., Effectiveness of prehospital wireless transmission of electrocardiograms to a cardiologist via hand-held device for patients with acute myocardial infarction (from the Timely Intervention in Myocardial Emergency, NorthEast Experience [TIME-NE]) (2006) Am J Cardiol, 98, pp. 1160-1164; Afolabi, B.A., Novaro, G.M., Pinski, S.L., Fromkin, K.R., Bush, H.S., Use of the prehospital ECG improves door-to-balloon times in ST segment elevation myocardial infarction irrespective of time of day or day of week (2007) Emerg Med J, 24, pp. 588-591; Terkelsen, C.J., Lassen, J.F., Norgaard, B.L., Gerdes, J.C., Poulsen, S.H., Bendix, K., Ankersen, J.P., Andersen, H.R., Reduction of treatment delay in patients with ST-elevation myocardial infarction: Impact of pre-hospital diagnosis and direct referral to primary percutanous coronary intervention (2005) Eur Heart J, 26, pp. 770-777; Wall, T., Albright, J., Livingston, B., Isley, L., Young, D., Nanny, M., Jaco-Bowitz, S., Wagner, G., Prehospital ECG transmission speeds reperfusion for patients with acute myocardial infarction (2000) N C Med J, 61, pp. 104-108; Dhruva, V.N., Abdelhadi, S.I., Anis, A., Gluckman, W., Hom, D., Dougan, W., Kaluski, E., Klapholz, M., ST-Segment Analysis Using Wireless Technology in Acute Myocardial Infarction (STAT-MI) trial (2007) J Am Coll Cardiol, 50, pp. 509-513; Sekulic, M., Hassunizadeh, B., McGraw, S., David, S., Feasibility of early emergency room notification to improve door-to-balloon times for patients with acute ST segment elevation myocardial infarction (2005) Catheter Cardiovasc Interv, 66, pp. 316-319; Swor, R., Hegerberg, S., McHugh-Mcnally, A., Goldstein, M., McEachin, C.C., Prehospital 12-lead ECG: Efficacy or effectiveness? (2006) Prehosp Emerg Care, 10, pp. 374-377; Campbell, P.T., Patterson, J., Cromer, D., Wall, K., Adams, G.L., Albano, A., Corey, C., Wagner, G., Prehospital triage of acute myocardial infarction: Wireless transmission of electrocardiograms to the on-call cardiologist via a handheld computer (2005) J Electrocardiol, 38, pp. 300-309; Lopez-Herce, J., Garcia, C., Dominguez, P., Carrillo, A., Rodriguez-Nunez, A., Calvo, C., Ma, D., Characteristics and outcome of cardiorespi-ratory arrest in children (2004) Resuscitation, 63, pp. 311-320; Rodriguez-Nunez, A., Lopez-Herce, J., Garcia, C., Dominguez, P., Carrillo, A., Bellon, J.M., Pediatric defibrillation after cardiac arrest: Initial response and outcome (2006) Crit Care, 10, pp. R113; Berg, R.A., Hilwig, R.W., Kern, K.B., Babar, I., Ewy, G.A., Simulated mouth-to-mouth ventilation and chest compressions (bystander cardiopulmo-nary resuscitation) improves outcome in a swine model of prehospital pediatric asphyxial cardiac arrest (1999) Crit Care Med, 27, pp. 1893-1899; Berg, R.A., Hilwig, R.W., Kern, K.B., Ewy, G.A., "Bystander" chest compressions and assisted ventilation independently improve outcome from piglet asphyxial pulseless "cardiac arrest." (2000) Circulation, 101, pp. 1743-1748; Iglesias, J.M., Lopez-Herce, J., Urbano, J., Solana, M.J., Mencia, S., Del Castillo, J., Chest compressions versus ventilation plus chest compressions in a pediatric asphyxial cardiac arrest animal model (2010) Intensive Care Med, 36, pp. 712-716; Tibballs, J., Weeranatna, C., The influence of time on the accuracy of healthcare personnel to diagnose paediatric cardiac arrest by pulse palpation (2010) Resuscitation, 81, pp. 671-675; Billi, J.E., Zideman, D.A., Eigel, B., Nolan, J.P., Montgomery, W.H., Nadkarni, V.M., Conflict of interest management before, during, and after the 2005 International Consensus Conference on Car-diopulmonary Resuscitation and Emergency Cardiovascular Care Science with Treatment Recommendations (2005) Circulation, 112 (22 SUPPL.), pp. 131-132. , from the International Liaison Committee on Resuscitation and the American Heart Association III; Billi, J.E., Eigel, B., Montgomery, W.H., Nadkarni, V.M., Hazinski, M.F., Management of conflict of interest issues in the activities of the American Heart Association Emergency Cardiovascular Care Committee, 2000-2005 (2005) Circulation, 112 (24 SUPPL.), pp. IV204-IV205; Billi, J.E., Shuster, M., Bossaert, L., De Caen, A.R., Deakin, C.D., Eigel, B., Hazinski, M.F., Zideman, D., Part 4: Conflict of interest management before, during, and after the 2010 International Consensus on Cardiopulmonary Resuscitation and Emergency Cardiovascular Care Science with Treatment Recommendations (2010) Circulation, 122 (SUPPL. 2), pp. S291-S297. , for the International Liaison Committee on Resuscitation and the American Heart Association.

995. Fisher L, Ormonde DG, Riley RH, Laurence BH. Endoscopic skills training in a simulated clinical setting. Simul Healthc. 2010;5(4):232-7. Epub 2011/02/19. doi: 10.1097/SIH.0b013e3181d2a7af. PubMed PMID: 21330802.

996. Fogel DB. Revisiting overlooked foundations of evolutionary computation: PART II. Cybern Syst. 2010;41(6):407-15. doi: 10.1080/01969722.2010.497993.

997. Fordham Z, Devereaux R, Edwards A. Teaching emergency medicine residents transvenous cardiac pacing: Simulation technology versus traditional methods. Acad Emerg Med [Internet]. 2010; 17:[S196 p.]. Available from: http://onlinelibrary.wiley.com/o/cochrane/clcentral/articles/712/CN-01055712/frame.html.

998. Fruscione R, Hyland D. Collaborative efforts of nursing students and surgical technology students in the simulation laboratory. Teaching and Learning in Nursing. 2010;5(2):78-84. doi: http://doi.org/10.1016/j.teln.2010.01.007.

999. Gaitan BD, Ramakrishna H, DiNardo JA, Cannesson M. Case 1-2010 Pulmonary Thrombectomy in an Adult With Fontan Circulation. J Cardiothorac Vasc Anesth. 2010;24(1):173-82. doi: 10.1053/j.jvca.2009.11.003.

1000. Garg MK, Kim DJ, Turaga DS, Prabhakaran B, editors. Multimodal analysis of body sensor network data streams for real-time healthcare. 2010 ACM SIGMM International Conference on Multimedia Information Retrieval, MIR 2010; 2010; Philadelphia, PA.

1001. Gaskell L, Beaton S. Inter-professional work based learning within an MSc in Advanced Practice: Lessons from one UK higher education programme. Nurse Education in Practice. 2010;10(5):274-8. doi: http://doi.org/10.1016/j.nepr.2009.11.017.

1002. Georgiopoulou VV, Kalogeropoulos AP, Raggi P, Butler J. Prevention, Diagnosis, and Treatment of Hypertensive Heart Disease. Cardiol Clin. 2010;28(4):675-91. doi: 10.1016/j.ccl.2010.07.005.

1003. Gerdprasert S, Pruksacheva T, Panijpan B, Ruenwongsa P. Development of a web-based learning medium on mechanism of labour for nursing students. Nurse Education Today. 2010;30(5):464-9. doi: http://doi.org/10.1016/j.nedt.2009.10.007.

1004. Geria V, Djordjevic V, Lhotska L, Krajca V, editors. PSGLab Matlab toolbox for polysomnographic data processing: Development and practical application. 10th International Conference on Information Technology and Applications in Biomedicine: Emerging Technologies for Patient Specific Healthcare, ITAB 2010; 2010; Corfu.

1005. Giakoumis D, Vogiannou A, Kosunen I, Moustakas K, Tzovaras D, Hassapis G, editors. Identifying psychophysiological correlates of boredom and negative mood induced during HCI. 1st International Wokshop on Bio-inspired Human-Machine Interfaces and Healthcare Applications, B-INTERFACE 2010, in Conjunction with BIOSTEC 2010; 2010; Valencia.

1006. Guglielmi M, Urbaz L, Tedesco C, Pusceddu A, Sogni A, Ronzoni G. A structured training program for awake fiber optic intubation: Teaching the complete package. Minerva Anestesiol. 2010;76(9):699-706.

1007. Hue O, Antoine-Jonville S, Galy O, Blonc S. Maximal oxygen uptake, ventilatory thresholds and mechanical power during cycling in Tropical climate in Guadeloupean elite cyclists. Journal of Science and Medicine in Sport. 2010;13(6):607-12. doi: http://doi.org/10.1016/j.jsams.2009.11.004.

1008. Iliopoulos CS, Michalakopoulos S. Combinatorial ECG analysis for mobile devices. Proceedings of the international conference on Multimedia information retrieval; Philadelphia, Pennsylvania, USA. 1743454: ACM; 2010. p. 409-12.

1009. Iliopoulos CS, Michalakopoulos S, editors. Combinatorial ECG analysis for mobile devices. 2010 ACM SIGMM International Conference on Multimedia Information Retrieval, MIR 2010; 2010; Philadelphia, PA.

1010. Jia D, Li N, Liu S, Li S, editors. Decision level fusion for pulse signal classification using multiple features. 3rd International Conference on BioMedical Engineering and Informatics, BMEI 2010; 2010; Yantai.

1011. Joshi AJ, Chandran S, Jayaraman VK, Kulkarni BD. Hybrid Support Vector Machine for imbalanced data in multiclass arrhythmia classification. Int J Funct Informatics Pers Med. 2010;3(1):29-47. doi: 10.1504/IJFIPM.2010.033244.

1012. Kempny A, Piorkowski A. CT2TEE--a novel, internet-based simulator of transoesophageal echocardiography in congenital heart disease. Kardiol Pol. 2010;68(3):374-9. Epub 2010/04/23. PubMed PMID: 20411468.

1013. Kempny A, Piórkowski A. CT2TEE - A novel, internet-based simulator of transoesophageal echocardiography in congenital heart disease. Kardiol Pol. 2010;68(3):374-9.

1014. Khazaee A, Ebrahimzadeh A. Classification of electrocardiogram signals with support vector machines and genetic algorithms using power spectral features. Biomed Signal Process Control. 2010;5(4):252-63. doi: 10.1016/j.bspc.2010.07.006.

1015. Klein R, Beanlands RSB, DeKemp RA. Quantification of myocardial blood flow and flow reserve: Technical aspects. J Nucl Cardiol. 2010;17(4):555-70. doi: 10.1007/s12350-010-9256-9

10.1007/s12350-010-9225-3; Wyss, C.A., Koepfli, P., Mikolajczyk, K., Burger, C., Von Schulthess, G.K., Kaufmann, P.A., Bicycle exercise stress in PET for assessment of coronary flow reserve-Repeatability and comparison with adenosine stress (2003) J. Nucl. Med., 44, pp. 146-154; (2010) Performance Measurements of Positron Emission Tomographs (PETs) by the Association of Electrical and Medical Equipment Manufacturers, , www.nema.org; DeKemp, R.A., Klein, R., Renaud, J., Alghamdi, A., Lortie, M., DaSilva, J.N., Beanlands, R.S., 3D list-mode cardiac PET for simultaneous quantification of myocardial blood flow and ventricular function (2008) Nucl. Sci. Symp. and Med. Imag. Conf. Record, pp. 5215-5218; Hsu, B., Casey, M.E., Watson, C.C., Bateman, T.M., Case, J.A., Validation of prompt gamma correction for 3D Rb-82 myocardial perfusion PET/CT imaging (2008) J. Nucl. Card., 15 (S), p. 4; Esteves, F.P., Nye, J.A., Khan, A., Folks, R.D., Halkar, R.K., Garcia, E.V., Schuster, D.M., Votaw, J.R., Prompt-gamma compensation in Rb-82 myocardial perfusion 3D PET/CT (2009) J. Nucl. Cardiol., 17, pp. 247-253; Boellaard, R., Van Lingen, A., Lammertsma, A.A., Experimental and clinical evaluation of iterative reconstruction (OSEM) in dynamic PET: Quantitative characteristics and effects on kinetic modeling (2001) J. Nucl. Med., 42, pp. 808-817; Cook, R.A.H., Carnes, G., Lee, T.-Y., Wells, R.G., Respiration-averaged CT for attenuation correction in canine cardiac PET/CT (2007) J. Nucl. Med., 48, pp. 811-818; Dilsizian, V., Bacharach, S.L., Beanlands, R.S., Bergmann, S.R., Delbeke, D., Gropler, R.J., Knuuti, J., Travin, M.I., ASNC imaging guidelines for nuclear cardiology procedures: PET myocardial perfusion and metabolism clinical imaging J. Nucl. Cardiol., , doi: 10.1007/s12350-009-9094-9; DeKemp, R.A., Klein, R., Lortie, M., Beanlands, R., Constant-activityrate infusions for myocardial blood flow quantification with 82Rb and 3D PET (2006) Nucl. Sci. Symp. and Med. Imag. Conf. Record, pp. 3519-3521; Renkin, E.M., Transport of potassium-42 from blood to tissue isolated mammalian skeletal muscles (1959) Am. J. Physiol., 197, pp. 1205-1210; Crone, C., Permeability of capillaries in various organs as determined by use of the indicator diffusion method (1963) Acta Physiol. Scand., 58, pp. 292-305; Katoh, C., Morita, K., Shiga, T., Kubo, N., Nakada, K., Tamaki, N., Improvement of algorithm for quantification of regional myocardial blood flow using 15O-water with PET (2004) J. Nucl. Med., 45, pp. 1908-1916; Iida, H., Kanno, I., Takahashi, A., Miura, S., Murakami, M., Takahashi, K., Ono, Y., Tomura, N., Measurement of absolute myocardial blood flow with H21 5O and dynamic positronemission tomography. Strategy for quantification in relation to the partial-volume effect (1988) Circulation, 78, pp. 104-115; Iida, H., Yokoyama, I., Agostini, D., Banno, T., Kato, T., Ito, K., Kuwabara, Y., Tamaki, N., Quantitative assessment of regional myocardial blood flow using oxygen-15-labelled water and positron emission tomography: A multicentre evaluation in Japan (2000) Eur. J. Nucl. Med., 27, pp. 192-201; Manabe, O., Yoshinaga, K., Katoh, C., Naya, M., De Kemp, R.A., Tamaki, N., Repeatability of rest and hyperemic myocardial blood flow measurements with 82Rb dynamic PET (2009) J. Nucl. Med., 50, pp. 68-71; El Fahkri, G., Sitek, A., Guerin, B., Kijewski, M.F., Di Carli, M.F., Moore, S.C., Quantitative dynamic cardiac 82Rb PET using generalized factor and compartment analyses (2005) J. Nucl. Med., 8, pp. 1264-1271; El Fakhri, G., Kardan, A., Sitek, A., Dorbala, S., Abi-Hatem, N., Lahoud, Y., Fischman, A., Di Carli, M.F., Reproducibility and accuracy of quantitative myocardial blood flow assessment with 82Rb PET: Comparison with 13N-ammonia PET (2009) J. Nucl. Med., 50, pp. 1062-1071; Wu, H.-M., Hoh, C.K., Choi, Y., Schelbert, H.R., Hawkins, R.A., Phelps, M.E., Huang, S.-C., Factor analysis for extraction of blood timeactivity curves in dynamic FDG-PET studies (1995) J. Nucl. Med., 36, pp. 1714-1722; Wu, H.-M., Hoh, C.K., Buxton, D.B., Kuhle, W.G., Schelbert, H.R., Choi, Y., Hawkins, R.A., Huang, S.-C., Quantification of myocardial blood flow using dynamic nitrogen-13-ammonia PET studies and factor analysis of dynamic structures (1995) J. Nucl. Med., 36, pp. 2087-2093; Klein, R., Beanlands, R.S., Wassenaar, R.W., Thorn, S., Lamoureux, M., DaSilva, J.N., Adler, A., DeKemp, R.A., Model based factor analysis of dynamic sequences of cardiac positron emission tomography (2010) Med. Phys., , in press; Gould, K.L., Pan, T., Loghin, C., Johnson, N.P., Guha, A., Sdringola, S., Frequent diagnostic errors in cardiac PET/CT due to misregistration of CT attenuation and emission PET images: A definitive analysis of causes, consequences, and corrections (2007) J. Nucl. Med., 48, pp. 1112-1121; Patterson, P.E., Eisner, R.L., Horowitz, S.F., Comparison of cost-effectiveness and utility of exercise ECG, single photon emission computer tomography, positron emission tomography, and coronary angiography for diagnosis of coronary artery disease (1995) Circulation, 91, pp. 54-65; Merhige, M.E., Breen, W.J., Shelton, V., Houston, T., D'Arcy, B.J., Perna, A.F., Impact of myocardial perfusion imaging with PET and (82) Rb on downstream invasive procedure utilization, costs, and outcomes in coronary disease management (2007) J. Nucl. Med., 48, pp. 1069-1076; Cherry, S., Dahlbom, M., PET: Physics, instrumentation, and scanners (2004) PET Molecular Imaging and its Biological, pp. 1-124. , Phelps M, editor, New York: Springer-Verlag; Schelbert, H.R., Phelps, M.E., Huang, S.C., MacDonald, N.S., Hansen, H., Selin, C., Kuhl, D.E., N-13 Ammonia as an indicator of myocardial blood flow (1981) Circulation, 63, pp. 1259-1272; Beanlands, R.S.B., Muzik, O., Mintun, M., Mangner, T., Lee, K., Petry, N., Hutchins, G.D., Schwaiger, M., The kinetics of copper-62-PTSM in the normal human heart (1992) J. Nucl. Med., 33, pp. 684-690; Lautamaki, R., George, R.T., Kitagawa, K., Rubidium-82 PET-CT for quantitative assessment of myocardial blood flow: Validation in a canine model of coronary artery stenosis (2009) Eur. J. Nucl. Med. Mol. Imaging, 36, pp. 576-586; Glatting, G., Bergmann, K.P., Stollfuss, J.C., Weismueller, P., Kochs, M., Hombach, V., Reske, S.N., Myocardial Rb extraction fraction: Determination in humans (1995) J. Am. Coll. Cardiol., 25, pp. 364A-5A; Schelbert, H.R., Positron emission tomography of the heart: Methodology, findings in the normal and the disease heart, and clinical applications PET: Molecular Imaging and its Biological Applications, , Phelps ME, editor, New York: Springer; Lecomte, R., Technology challenges in small animal PET imaging (2004) Nucl. Instrum. Meth. Phys. Res. A, 527, pp. 157-165; Bergmann, S.R., Fox, K.A., Rand, A.L., McElvany, K.D., Welch, M.J., Markham, J., Sobel, B.E., Quantification of regional myocardial blood flow in vivo with H215O (1984) Circulation, 70, pp. 724-733; Yoshida, K., Mullani, N., Gould, K.L., Coronary flow and flow reserve by PET simplified for clinical applications using rubidium-82 or nitrogen-13-ammonia (1996) J. Nucl. Med., 37, pp. 1701-1712.

1016. Kobayashi L, Lindquist DG, Jenouri IM, Dushay KM, Haze D, Sutton EM, et al. Comparison of sudden cardiac arrest resuscitation performance data obtained from in-hospital incident chart review and in situ high-fidelity medical simulation. Resuscitation. 2010;81(4):463-71. doi: 10.1016/j.resuscitation.2010.01.003.

1017. Lamounier Jr E, Bucioli A, Cardoso A, Andrade A, Soares A, editors. On the use of Augmented Reality techniques in learning and interpretation of cardiologic data. 2010 32nd Annual International Conference of the IEEE Engineering in Medicine and Biology Society, EMBC'10; 2010; Buenos Aires.

1018. Lanatà A, Valenza G, Scilingo EP, editors. The contribution of the phase spectrum in automatic multiple cardiac arrhythmias recognition in wearable systems. 2010 3rd International Symposium on Applied Sciences in Biomedical and Communication Technologies, ISABEL 2010; 2010; Roma.

1019. Lang W, Morse M, Patel JM. Dictionary-based compression for long time-series similarity. IEEE Trans Knowl Data Eng. 2010;22(11):1609-22. doi: 10.1109/TKDE.2009.201.

1020. Lapkin S, Levett-Jones T, Bellchambers H, Fernandez R. Effectiveness of Patient Simulation Manikins in Teaching Clinical Reasoning Skills to Undergraduate Nursing Students: A Systematic Review. Clinical Simulation in Nursing. 2010;6(6):e207-e22. doi: http://doi.org/10.1016/j.ecns.2010.05.005.

1021. Lee TY, Chhem RK. Impact of new technologies on dose reduction in CT. Eur J Radiol. 2010;76(1):28-35. doi: 10.1016/j.ejrad.2010.06.036.

1022. Li J, Tamminedi T, Yosiphon G, Ganguli A, Zhang L, Stankovic JA, et al., editors. Remote physiological monitoring of first responders with intermittent network connectivity. 1st Wireless Health Conference, WH'10; 2010; San Diego, CA.

1023. Li X, Porikli F, editors. Human state classification and predication for critical care monitoring by real-time bio-signal analysis. 2010 20th International Conference on Pattern Recognition, ICPR 2010; 2010; Istanbul.

1024. Li Y, Poon CCY, Zhang YT. Analog integrated circuits design for processing physiological signals. IEEE Rev Biomed Eng. 2010;3:93-105. doi: 10.1109/RBME.2010.2082521.

1025. Lian S, Zheng G, editors. Similarity measurement study for bio-signal waveform based on tunnel morph. 3rd International Conference on BioMedical Engineering and Informatics, BMEI 2010; 2010; Yantai.

1026. Lighthall GK, Poon T, Harrison TK. Using In Situ Simulation to Improve In-Hospital Cardiopulmonary Resuscitation. The Joint Commission Journal on Quality and Patient Safety. 2010;36(5):209-16. doi: http://doi.org/10.1016/S1553-7250(10)36034-X.

1027. Lin FX, Rahmati A, Zhong L. Dandelion: a framework for transparently programming phone-centered wireless body sensor applications for health. Wireless Health 2010; San Diego, California. 1921091: ACM; 2010. p. 74-83.

1028. Lin J, Sun Y, Wang W, editors. Violence detection in movies with auditory and visual cues. 2010 International Conference on Computational Intelligence and Security, CIS 2010; 2010; Nanning.

1029. Linte CA, White J, Eagleson R, Guiraudon GM, Peters TM. Virtual and augmented medical imaging environments: Enabling technology for minimally invasive cardiac interventional guidance. IEEE Rev Biomed Eng. 2010;3:25-47. doi: 10.1109/RBME.2010.2082522.

1030. Liu L, Ge Y, Liu M, Cao H, Liu Y, Zhang G, et al., editors. The application of an apparatus of motion measurement and analysis in freestyle aerials. 2010 2nd WRI Global Congress on Intelligent Systems, GCIS 2010; 2010; Wuhan.

1031. Mancini ME, Soar J, Bhanji F, Billi JE, Dennett J, Finn J, et al. Part 12: Education, implementation, and teams: 2010 International Consensus on Cardiopulmonary Resuscitation and Emergency Cardiovascular Care Science with Treatment Recommendations. Circulation. 2010;122(16 SUPPL. 2):S539-S81. doi: 10.1161/CIRCULATIONAHA.110.971143.

1032. Mendez MO, Matteucci M, Castronovo V, Ferini-Strambi L, Cerutti S, Bianchi AM. Sleep staging from heart rate variability: Time-varying spectral features and hidden markov models. Int J Biomed Eng Technol. 2010;3(3-4):246-63. doi: 10.1504/IJBET.2010.032695.

1033. Meschia JF, McNeil RB, Barrett KM, Brott TG, Graff-Radford NR, Brown Jr RD. Mayo Acute Stroke Trial for Enhancing Recovery (MASTER) Protocol. Journal of Stroke and Cerebrovascular Diseases. 2010;19(4):299-310. doi: http://doi.org/10.1016/j.jstrokecerebrovasdis.2009.05.005.

1034. Micó P, Mora M, Cuesta-Frau D, Aboy M. Automatic segmentation of long-term ECG signals corrupted with broadband noise based on sample entropy. COMPUT METHODS PROGRAMS BIOMED. 2010;98(2):118-29. doi: 10.1016/j.cmpb.2009.08.010

http://doi.ieeecomputersociety.org/10.1109/SSAP.1996.534872; Raskinis, A., Raskinis, G., Application of symbolic machine learning to audio signal segmentatin (2005) Nonlinear Speech Modeling and Applications, 3445, pp. 397-403; Richman, J.S., Moorman, J.R., Physiological time-series analysis using approximate entropy and sample entropy (2000) American Journal of Physiology-Heart and Circulatory Physiology, 278 (6), pp. H2039-H2049; Clifford, G., Zapanta, L., Janz, B., Mietus, J., Youn, C., Mark, R., (2005), pp. 595-598. , doi:10.1109/CIC.2005.1588171, Segmentation of 24-hour cardiovascular activity using ecg-based sleep/sedation and noise metrics, in: Computers in CardiologyMahmoodi, S., Sharif, B.S., Signal segmentation and denoising algorithm based on energy optimisation (2005) Signal Processing, 85 (9), pp. 1845-1851; Morales, R.O., Sanchez, M.A.P., Ginori, J.V.L., Abalo, R.G., Ramirez, R.R., Evaluation of qrs morphological classifiers in the presence of noise (1997) Computers and Biomedical Research, 30 (3), pp. 200-210; Aboy, M., Cuesta-Frau, D., Austin, D., Mico-Tormos, P., Characterization of sample entropy in the context of biomedical signal analysis (2007) IEEE 29th Annual International Engineering in Medicine and Biology Society Conference (EMBC-07), pp. 5942-5945; Goldberger, A.L., Amaral, L.A.N., Glass, L., Hausdorff, J.M., Ivanov, P.C., Mark, R.G., Mietus, J.E., Stanley, H.E., PhysioBank, PhysioToolkit, and PhysioNet: components of a new research resource for complex physiologic signals (2000) Circulation, 101 (23), pp. e215-e220; Moody, G., Muldrow, W., Mark, R., A noise stress test for arrhythmia detectors (1984) Computers in Cardiology, vol. 11, pp. 381-384; Lake, D., Richman, J.S., Griffin, M.P., Moorman, J.R., Sample entropy analysis of neonatal heart rate variability (2002) American Journal of Physiology. Regulatory, Integrative and Comparative Physiology, 283 (3), pp. 789-797; Laurent, H., Doncarli, C., Stationarity index for abrupt changes detection in the time-frequency plane (1998) IEEE Signal Processing Letters, 5 (2), pp. 43-45.

1035. Miyaji M, Kawanaka H, Oguri K, editors. Study on effect of adding pupil diameter as recognition features for driver's cognitive distraction detection. 2010 7th International Symposium on Communication Systems, Networks and Digital Signal Processing, CSNDSP 2010; 2010; Newcastle upon Tyne.

1036. Morrison LJ, Deakin CD, Morley PT, Callaway CW, Kerber RE, Kronick SL, et al. Part 8: Advanced life support: 2010 International Consensus on Cardiopulmonary Resuscitation and Emergency Cardiovascular Care Science with Treatment Recommendations. Circulation. 2010;122(16 SUPPL. 2):S345-S421. doi: 10.1161/CIRCULATIONAHA.110.971051
[truncated: 1,155,745 more chars]
