# Supplementary material for: Educational Software Applied in Teaching Electrocardiogram: A Systematic Review
Source: Biomed Res Int. 2018 Mar 15;2018:8203875. doi: 10.1155/2018/8203875 (PMC5875041; doi:10.1155/2018/8203875)
Supplement: Supplementary 2 — S2 Appendix: screening—records after duplicates removed (DOCX). [file 8203875.f2.docx]

**S2 Appendix -SCREENING -Records after duplicates removed**

The following 2467 after duplicates removed.

1.Nakao M, Komori M, Matsuda T, Takahashi T, editors. 4D visible and palpable simulation using dynamic pressure model based on cardiac morphology. 2001 Medicine Meets Virtual Reality Conference: Outer Space, Inner Space, Virtual Space, MMVR 2001; 2001; Newport Beach, CA2001138103.

2. Baxter R. 12 lead ECG interpretation: The self-assessment Approach: W. B. Saunders &amp; Company, Philadelphia 1989, ISBN 7216 2846X 304 pages. Price £13.50. Intensive Care Nursing. 1990;6(4):213-4. doi: http://doi.org/10.1016/0266-612X(90)90033-4.

3. Chen DG, Tang F, Law MK, Bermak A. A 12 pJ/Pixel analog-to-information converter based 816 × 640 pixel CMOS image sensor. IEEE J Solid State Circuits. 2014;49(5):1210-22. doi: 10.1109/JSSC.2014.2307063.

4. Alinier G, Gordon R, Harwood C, Hunt WB. 12-Lead ECG training: The way forward. Nurse Education Today. 2006;26(1):87-92. doi: http://doi.org/10.1016/j.nedt.2005.08.004.

5. 17th National ASPANConference Abstracts. Journal of PeriAnesthesia Nursing. 1998;13(3):207-14. doi: http://doi.org/10.1016/S1089-9472(98)80052-X.

6. 19th National ASPAN Conference Abstracts. Journal of PeriAnesthesia Nursing. 2000;15(3):202-13. doi: http://doi.org/10.1016/S1089-9472(00)80024-6.

7. Menzies T. 21st-century al: Proud, not smug. IEEE Intell Syst. 2003;18(3):18-24. doi: 10.1109/MIS.2003.1200723.

8. 23rd Annual Conference. Journal of the Association for Vascular Access. 2009;14(4):171-86. doi: http://doi.org/10.1016/S1552-8855(09)70071-6.

9. Snyder O. A 71-year-old man with syncope, respiratory distress, and hypotension. Journal of Emergency Nursing. 2000;26(5):522-3. doi: http://doi.org/10.1067/men.2000.110010.

10. de Virgilio C, Yaghoubian A, Lewis RJ, Stabile BE, Putnam BA. The 80-Hour Resident Workweek Does Not Adversely Affect Patient Outcomes or Resident Education. Curr Surg. 2006;63(6):435-9. doi: 10.1016/j.cursur.2006.03.006.

11. 1994 Buyer's guide. Air Medical Journal. 1994;13(5):167-200. doi: http://doi.org/10.1016/S1067-991X(05)80108-7.

12. 2001 Congress information and highlights: Sunday, March 11, to Thursday, March 15, 2001. AORN Journal. 2001;73(2):320-34. doi: http://doi.org/10.1016/S0001-2092(06)61976-5.

13. 2007 AMTC Scientific Assembly Monday, September 17, 2007. Air Medical Journal. 2007;26(5):230-9. doi: http://doi.org/10.1016/j.amj.2007.06.002.

14. 2008 AMTC Scientific Assembly Monday, October 20, 2008. Air Medical Journal. 2008;27(5):230-7. doi: http://doi.org/10.1016/j.amj.2008.07.008.

15. David Hillis L, Smith PK, Bittl JA, Bridges CR, Byrne JG, Cigarroa JE, et al. 2011 ACCF/AHA guideline for coronary artery bypass graft surgery a report of the American College of Cardiology Foundation/American Heart Association Task Force on Practice Guidelines. Circulation. 2011;124(23):e652-e735. doi: 10.1161/CIR.0b013e31823c074e

10.1161/CIR. 0b013e318235eb4d. Accessed November 3, 2011; Drew, B.J., Califf, R.M., Funk, M., Kaufman, E.S., Krucoff, M.W., Laks, M.M., Macfarlane, P.W., Van Hare, G.F., Practice standards for electrocardiographic monitoring in hospital settings: An American Heart Association scientific statement from the councils on cardiovascular nursing, clinical cardiology, and cardiovascular disease in the young (2004) Circulation, 110 (17), pp. 2721-2746. , DOI 10.1161/01.CIR.0000145144.56673.59; Echahidi, N., Pibarot, P., O'Hara, G., Mathieu, P., Mechanisms, Prevention, and Treatment of Atrial Fibrillation After Cardiac Surgery (2008) Journal of the American College of Cardiology, 51 (8), pp. 793-801. , DOI 10.1016/j.jacc.2007.10.043, PII S0735109707037813; Gordon, M.A., Urban, M.K., O'Connor, T., Is the pressure rate quotient a predictor or indicator of myocardial ischemia as measured by ST-segment changes in patients undergoing coronary artery bypass surgery? (1991) Anesthesiology, 74, pp. 848-853; Jain, U., Laflamme, C.J.A., Aggarwal, A., Ramsay, J.G., Comunale, M.E., Ghoshal, S., Ngo, L., Mangano, D.T., Electrocardiographic and hemodynamic changes and their association with myocardial infarction during coronary artery bypass surgery: A multicenter study (1997) Anesthesiology, 86 (3), pp. 576-591. , DOI 10.1097/00000542-199703000-00009; Knight, A.A., Hollenberg, M., London, M.J., Perioperative myocardial ischemia: Importance of the preoperative ischemic pattern (1988) Anesthesiology, 68, pp. 681-688; Mangano, D.T., Siliciano, D., Hollenberg, M., The Study of Perioperative Ischemia (SPI) Research Group. Postoperative myocardial ischemia. Therapeutic trials using intensive analgesia following surgery (1992) Anesthesiology, 76, pp. 342-353; Zvara, D.A., Groban, L., Rogers, A.T., Prophylactic nitroglycerin did not reduce myocardial ischemia during accelerated recovery management of coronary artery bypass graft surgery patients (2000) J Cardiothorac Vasc Anesth, 14, pp. 571-575; Berry, P.D., Thomas, S.D., Mahon, S.P., Jackson, M., Fox, M.A., Fabri, B., Weir, W.I., Russell, G.N., Myocardial ischaemia after coronary artery bypass grafting: Early vs late extubation (1998) British Journal of Anaesthesia, 80 (1), pp. 20-25; Cheng, D.C.H., Karski, J., Peniston, C., Asokumar, B., Raveendran, G., Carroll, J., Nierenberg, H., Sandler, A., Morbidity outcome in early versus conventional tracheal extubation after coronary artery bypass grafting: A prospective randomized controlled trial (1996) Journal of Thoracic and Cardiovascular Surgery, 112 (3), pp. 755-764. , DOI 10.1016/S0022-5223(96)70062-4; Practice guidelines for pulmonary artery catheterization: An updated report by the American Society of anesthesiologists task force on pulmonary artery catheterization (2003) Anesthesiology, 99, pp. 988-1014; Pearson, K.S., Gomez, M.N., Moyers, J.R., Carter, J.G., Tinker, J.H., A cost/benefit analysis of randomized invasive monitoring for patients undergoing cardiac surgery (1989) Anesthesia and Analgesia, 69 (3), pp. 336-341; Resano, F.G., Kapetanakis, E.I., Hill, P.C., Haile, E., Corso, P.J., Clinical Outcomes of Low-Risk Patients Undergoing Beating-Heart Surgery With or Without Pulmonary Artery Catheterization (2006) Journal of Cardiothoracic and Vascular Anesthesia, 20 (3), pp. 300-306. , DOI 10.1053/j.jvca.2006.01.017, PII S1053077006000024; Schwann, T.A., Zacharias, A., Riordan, C.J., Durham, S.J., Engoren, M., Habib, R.H., Safe, highly selective use of pulmonary artery catheters in coronary artery bypass grafting: An objective patient selection method (2002) Annals of Thoracic Surgery, 73 (5), pp. 1394-1401. , DOI 10.1016/S0003-4975(02)03422-7, PII S0003497502034227; Stewart, R.D., Psyhojos, T., Lahey, S.J., Levitsky, S., Campos, C.T., Central venous catheter use in low-risk coronary artery bypass grafting (1998) Annals of Thoracic Surgery, 66 (4), pp. 1306-1311. , DOI 10.1016/S0003-4975(98)00760-7, PII S0003497598007607; Tuman, K.J., McCarthy, R.J., Spiess, B.D., DaValle, M., Hompland, S.J., Dabir, R., Ivankovich, A.D., Effect of pulmonary artery catheterization on outcome in patients undergoing coronary artery surgery (1989) Anesthesiology, 70 (2), pp. 199-206; Avidan, M.S., Zhang, L., Burnside, B.A., Finkel, K.J., Searleman, A.C., Selvidge, J.A., Saager, L., Evers, A.S., Anesthesia awareness and the bispectral index (2008) New England Journal of Medicine, 358 (11), pp. 1097-1108. , http://content.nejm.org/cgi/reprint/358/11/1097.pdf, DOI 10.1056/NEJMoa0707361; Hemmerling, T.M., Olivier, J.-F., Basile, F., Le, N., Prieto, I., Bispectral index as an indicator of cerebral hypoperfusion during off-pump coronary artery bypass grafting (2005) Anesthesia and Analgesia, 100 (2), pp. 354-356. , DOI 10.1213/01.ANE.0000140245.44494.12; Myles, P.S., Leslie, K., McNeil, J., Forbes, A., Chan, M.T.V., Bispectral index monitoring to prevent awareness during anaesthesia: The B-Aware randomised controlled trial (2004) Lancet, 363 (9423), pp. 1757-1763. , DOI 10.1016/S0140-6736(04)16300-9, PII S0140673604163009; Brady, K., Joshi, B., Zweifel, C., Real-time continuous monitoring of cerebral blood flow autoregulation using near-infrared spectroscopy in patients undergoing cardiopulmonary bypass (2010) Stroke, 41; Murkin, J.M., Adams, S.J., Novick, R.J., Quantz, M., Bainbridge, D., Iglesias, I., Cleland, A., Fox, S., Monitoring brain oxygen saturation during coronary bypass surgery: A randomized, prospective study (2007) Anesthesia and Analgesia, 104 (1), pp. 51-58. , DOI 10.1213/01.ane.0000246814.29362.f4, PII 0000053920070100000011; Slater, J.P., Guarino, T., Stack, J., Cerebral oxygen desaturation predicts cognitive decline and longer hospital stay after cardiac surgery (2009) Ann Thorac Surg, 87, pp. 36-44; American society of anesthesiologists Standards for Basic Anesthetic Monitoring, , http://www.asahq.org/For-Members/Clinical-Information/_/media/ For%20Members/documents/Standards%20Guidelines%20Stmts/ Basic%20Anesthetic%20Monitoring%202011.ashx, Committee of Origin: Standards and Practice Parameters Last amended October 20, 2010. Accessed July 1, 2011; Chatterjee, K., The Swan-Ganz catheters: Past, present, and future. A viewpoint (2009) Circulation, 119, pp. 147-152; London, M.J., Moritz, T.E., Henderson, W.G., Sethi, G.K., O'Brien, M.M., Grunwald, G.K., Beckman, C.B., Grover, F.L., Standard versus fiberoptic pulmonary artery catheterization for cardiac surgery in the Department of Veterans Affairs: A prospective, observational, multicenter analysis (2002) Anesthesiology, 96 (4), pp. 860-870. , DOI 10.1097/00000542-200204000-00013; Vincent, J.L., Pinsky, M.R., Sprung, C.L., The pulmonary artery catheter: In medio virtus (2008) Crit Care Med, 36, pp. 3093-3096; Bernard, G.R., Sopko, G., Cerra, F., Demling, R., Edmunds, H., Kaplan, S., Kessler, L., Williams, D., Pulmonary artery catheterization and clinical outcomes: National Heart, Lung, and Blood Institute and Food and Drug Administration workshop report (2000) Journal of the American Medical Association, 283 (19), pp. 2568-2572; Fleisher, L.A., Beckman, J.A., Brown, K.A., 2009 ACCF/AHA focused update on perioperative beta blockade incorporated into the ACC/AHA 2007 guidelines on perioperative cardiovascular evaluation and care for noncardiac surgery (2009) Circulation, 120, pp. e169-e276; Isley, M.R., Edmonds Jr., H.L., Stecker, M., Guidelines for intraoperative neuromonitoring using raw (analog or digital waveforms) and quantitative electroencephalography: A position statement by the American Society of Neurophysiological Monitoring (2009) J Clin Monit Comput, 23, pp. 369-390; Edmonds Jr., H.L., Isley, M.R., Sloan, T.B., American society of neurophysiologic monitoring and American society of neuroim-aging joint guidelines for transcranial doppler ultrasonic monitoring (2011) J Neuroimaging, 21, pp. 177-183; Practice advisory for intraoperative awareness and brain function monitoring: A report by the American society of anesthesiologists task force on intraoperative awareness (2006) Anesthesiology, 104, p. 864; Edmonds Jr., H.L., 2010 standard of care for central nervous system monitoring during cardiac surgery (2010) J Cardiothorac Vasc Anesth, 24, pp. 541-543; Kertai, M.D., Pal, N., Palanca, B.J., Association of perioperative risk factors and cumulative duration of low bispectral index with intermediate-term mortality after cardiac surgery in the B-Unaware Trial (2010) Anesthesiology, 112, pp. 1116-1127; Monk, T.G., Saini, V., Weldon, B.C., Sigl, J.C., Anesthetic management and one-year mortality after noncardiac surgery (2005) Anesthesia and Analgesia, 100 (1), pp. 4-10. , DOI 10.1213/01.ANE.0000147519.82841.5E; Murkin, J., Arango, M., Near-infrared spectroscopy as an index of brain and tissue oxygenation (2009) Br J Anaesth, 103, pp. i3-i13; Heringlake, M., Garbers, C., Kabler, J., Preoperative cerebral oxygen saturation and clinical outcomes in cardiac surgery (2010) Anesthesiology, 114, pp. 12-13; Clark, R.E., The development of the Society of Thoracic Surgeons voluntary national database system: Genesis, issues, growth, and status (1996) Best Pract Benchmarking Healthc, 1, pp. 62-69; Kouchoukos, N.T., Ebert, P.A., Grover, F.L., Lindesmith, G.G., Report of the Ad Hoc committee on risk factors for coronary artery bypass surgery (1988) Annals of Thoracic Surgery, 45 (3), pp. 348-349; Geraci, J.M., Johnson, M.L., Gordon, H.S., Petersen, N.J., Shroyer, A.L., Grover, F.L., Wray, N.P., Mortality after cardiac bypass surgery: Prediction from administrative versus clinical data (2005) Medical Care, 43 (2), pp. 149-158. , DOI 10.1097/00005650-200502000-00008; Hannan, E.L., Kilburn Jr., H., Lindsey, M.L., Clinical versus administrative data bases for CABG surgery. Does it matter? (1992) Med Care, 30, pp. 892-907; Hannan, E.L., Racz, M.J., Jollis, J.G., Peterson, E.D., Using medicare claims data to assess provider quality for CABG surgery: Does it work well enough? (1997) Health Services Research, 31 (6), pp. 659-678; Hartz, A.J., Kuhn, E.M., Comparing hospitals that perform coronary artery bypass surgery: The effect of outcome measures and data sources (1994) American Journal of Public Health, 84 (10), pp. 1609-1614; Jones, R.H., Hannan, E.L., Hammermeister, K.E., DeLong, E.R., O'Connor, G.T., Luepker, R.V., Parsonnet, V., Pryor, D.B., Identification of preoperative variables needed for risk adjustment of short-term mortality after coronary artery bypass graft surgery (1996) Journal of the American College of Cardiology, 28 (6), pp. 1478-1487. , DOI 10.1016/S0735-1097(96)00359-2, PII S0735109796003592; Mack, M.J., Herbert, M., Prince, S., Dewey, T.M., Magee, M.J., Edgerton, J.R., Does reporting of coronary artery bypass grafting from administrative databases accurately reflect actual clinical outcomes? (2005) Journal of Thoracic and Cardiovascular Surgery, 129 (6), pp. 1309-1317. , DOI 10.1016/j.jtcvs.2004.10.036, PII S0022522304016861; Shahian, D.M., Silverstein, T., Lovett, A.F., Wolf, R.E., Normand, S.-L.T., Comparison of clinical and administrative data sources for hospital coronary artery bypass graft surgery report cards (2007) Circulation, 115 (12), pp. 1518-1527. , DOI 10.1161/CIRCULATIONAHA.106.633008; Tu, J.V., Sykora, K., Naylor, C.D., Assessing the outcomes of coronary artery bypass graft surgery: How many risk factors are enough? (1997) Journal of the American College of Cardiology, 30 (5), pp. 1317-1323. , DOI 10.1016/S0735-1097(97)00295-7, PII S0735109797002957; Grover, F.L., Johnson, R.R., Marshall, G., Factors predictive of operative mortality among coronary artery bypass subsets (1993) Ann Thorac Surg, 56, pp. 1296-1306; Grover, F.L., Johnson, R.R., Shroyer, A.L.W., Marshall, G., Hammermeister, K.E., The Veterans Affairs continuous improvement in cardiac surgery study (1994) Annals of Thoracic Surgery, 58 (6), pp. 1845-1851; Grover, F.L., Shroyer, A.L.W., Hammermeister, K.E., Calculating risk and outcome: The Veterans Affairs database (1996) Annals of Thoracic Surgery, 62 (SUPPL.), pp. S6-S11. , DOI 10.1016/0003-4975(96)00821-1, PII S0003497596008211; O'Connor, G.T., Plume, S.K., Olmstead, E.M., A regional prospective study of in-hospital mortality associated with coronary artery bypass grafting (1991) JAMA, 266, pp. 803-809. , the Northern New England Cardiovascular Disease Study Group; O'Connor, G.T., Plume, S.K., Olmstead, E.M., Multivariate prediction of in-hospital mortality associated with coronary artery bypass graft surgery (1992) Circulation, 85, pp. 2110-2118. , Northern New England Cardiovascular Disease Study Group; Hannan, E.L., Kilburn Jr., H., O'Donnell, J.F., Adult open heart surgery in New York State. An analysis of risk factors and hospital mortality rates (1990) JAMA, 264, pp. 2768-2774; Hannan, E.L., Kumar, D., Racz, M., Siu, A.L., Chassin, M.R., New York State's Cardiac Surgery Reporting System: Four years later (1994) Annals of Thoracic Surgery, 58 (6), pp. 1852-1857; Shahian, D.M., Normand, S.-L., Torchiana, D.F., Lewis, S.M., Pastore, J.O., Kuntz, R.E., Dreyer, P.I., Cardiac surgery report cards: Comprehensive review and statistical critique (2001) Annals of Thoracic Surgery, 72 (6), pp. 2155-2168. , DOI 10.1016/S0003-4975(01)03222-2, PII S0003497501032222; Shahian, D.M., Torchiana, D.F., Normand, S.-L.T., Implementation of a cardiac surgery report card: Lessons from the Massachusetts experience (2005) Annals of Thoracic Surgery, 80 (3), pp. 1146-1150. , DOI 10.1016/j.athoracsur.2004.10.046, PII S0003497504021484; Hammermeister, K.E., Daley, J., Grover, F.L., Using outcomes data to improve clinical practice: What we have learned (1994) Annals of Thoracic Surgery, 58 (6), pp. 1809-1811; Hammermeister, K.E., Johnson, R., Marshall, G., Grover, F.L., Continuous assessment and improvement in quality of care: A model from the Department of Veterans Affairs Cardiac Surgery (1994) Annals of Surgery, 219 (3), pp. 281-290; Ferguson Jr., T.B., Dziuban Jr, S.W., Edwards, F.H., Eiken, M.C., Shroyer, A.L.W., Pairolero, P.C., Anderson, R.P., Grover, F.L., The STS national database: Current changes and challenges for the new millennium (2000) Annals of Thoracic Surgery, 69 (3), pp. 680-691. , DOI 10.1016/S0003-4975(99)01538-6, PII S0003497599015386; Ferguson Jr., T.B., Peterson, E.D., Coombs, L.P., Eiken, M.C., Carey, M.L., Grover, F.L., DeLong, E.R., Use of Continuous Quality Improvement to Increase Use of Process Measures in Patients Undergoing Coronary Artery Bypass Graft Surgery: A Randomized Controlled Trial (2003) Journal of the American Medical Association, 290 (1), pp. 49-56. , DOI 10.1001/jama.290.1.49; O'Connor, G.T., Plume, S.K., Olmstead, E.M., Morton, J.R., Maloney, C.T., Nugent, W.C., Hernandez Jr., F., Kasper, J.F., A regional intervention to improve the hospital mortality associated with coronary artery bypass graft surgery (1996) Journal of the American Medical Association, 275 (11), pp. 841-846. , DOI 10.1001/jama.275.11.841; Hannan, E.L., Kilburn Jr., H., Racz, M., Shields, E., Chassin, M.R., Improving the outcomes of coronary artery bypass surgery in New York State (1994) Journal of the American Medical Association, 271 (10), pp. 761-766. , DOI 10.1001/jama.271.10.761; Hannan, E.L., Siu, A.L., Kumar, D., The decline in coronary artery bypass graft surgery mortality in New York State. The role of surgeon volume (1995) JAMA, 273, pp. 209-213; Peterson, E.D., Delong, E.R., Jollis, J.G., Muhlbaier, L.H., Mark, D.B., The effects of New York's bypass surgery provider profiling on access to care and patient outcomes in the elderly (1998) Journal of the American College of Cardiology, 32 (4), pp. 993-999. , DOI 10.1016/S0735-1097(98)00332-5, PII S0735109798003325; Ghali, W.A., Ash, A.S., Hall, R.E., Moskowitz, M.A., Statewide quality improvement initiatives and mortality after cardiac surgery (1997) Journal of the American Medical Association, 277 (5), pp. 379-382; Guru, V., Fremes, S.E., Naylor, C.D., Austin, P.C., Shrive, F.M., Ghali, W.A., Tu, J.V., Public versus private institutional performance reporting: What is mandatory for quality improvement? (2006) American Heart Journal, 152 (3), pp. 573-578. , DOI 10.1016/j.ahj.2005.10.026, PII S0002870306001219; Hannan, E.L., Sarrazin, M.S., Doran, D.R., Provider profiling and quality improvement efforts in coronary artery bypass graft surgery: The effect on short-term mortality among Medicare beneficiaries (2003) Med Care, 41, pp. 1164-1172; Chassin, M.R., Achieving and sustaining improved quality: Lessons from New York State and cardiac surgery (2002) Health Aff (Millwood), 21, pp. 40-51; Erickson, L.C., Torchiana, D.F., Schneider, E.C., Newburger, J.W., Hannan, E.L., The relationship between managed care insurance and use of lower- mortality hospitals for CABG surgery (2000) Journal of the American Medical Association, 283 (15), pp. 1976-1982; Hannah, E.L., Stone, C.C., Biddie, T.L., DeBuono, B.A., Public release of cardiac surgery outcomes data in New York: What do New York State cardiologists think of it? (1997) American Heart Journal, 134 (1), pp. 55-61. , DOI 10.1016/S0002-8703(97)70106-6; Jha, A.K., Epstein, A.M., The predictive accuracy of the New York State coronary artery bypass surgery report-card system (2006) Health Affairs, 25 (3), pp. 844-855. , http://content.healthaffairs.org/cgi/reprint/25/3/844.pdf, DOI 10.1377/hlthaff.25.3.844; Mukamel, D.B., Mushlin, A.I., Quality of Care Information Makes a Difference: An Analysis of Market Share and Price Changes after Publication of the New York State Cardiac Surgery Mortality Reports (1998) Medical Care, 36 (7), pp. 945-954; Mukamel, D.B., Mushlin, A.I., The impact of quality report cards on choice of physicians, hospitals, and HMOs: A midcourse evaluation (2001) Jt Comm J Qual Improv, 27, pp. 20-27; Mukamel, D.B., Weimer, D.L., Mushlin, A.I., Interpreting market share changes as evidence for effectiveness of quality report cards (2007) Med Care, 45, pp. 1227-1232; Schauffler, H.H., Mordavsky, J.K., Consumer reports in health care: Do they make a difference? (2001) Annual Review of Public Health, 22, pp. 69-89. , DOI 10.1146/annurev.publhealth.22.1.69; Shahian, D.M., Yip, W., Westcott, G., Selection of a cardiac surgery provider in the managed care era (2000) J Thorac Cardiovasc Surg, 120, pp. 978-987; Werner, R.M., Asch, D.A., The unintended consequences of publicly reporting quality information (2005) Journal of the American Medical Association, 293 (10), pp. 1239-1244. , DOI 10.1001/jama.293.10.1239; Romano, P.S., Zhou, H., Do well-publicized risk-adjusted outcomes reports affect hospital volume? (2004) Med Care, 42, pp. 367-377; Hibbard, J.H., Sofaer, S., Jewett, J.J., Condition-specific performance information: Assessing salience, comprehension, and approaches for communicating quality (1996) Health Care Financing Review, 18 (1), pp. 95-109; Hibbard, J.H., Jowett, J.J., Will Quality Report Cards Help Consumers? (1997) Health Affairs, 16 (3), pp. 218-228; Hibbard, J.H., Slovic, P., Jewett, J.J., Informing Consumer Decisions in Health Care: Implications from Decision-Making Research (1997) Milbank Quarterly, 75 (3), pp. 395-414; Hibbard, J.H., Peters, E., Slovic, P., Making health care quality reports easier to use (2001) Jt Comm J Qual Improv, 27, pp. 591-604; Hibbard, J.H., Peters, E., Supporting informed consumer health care decisions: Data presentation approaches that facilitate the use of information in choice (2003) Annual Review of Public Health, 24, pp. 413-433. , DOI 10.1146/annurev.publhealth.24.100901.141005; Green, J., Wintfeld, N., Report cards on cardiac surgeons. Assessing New York State's approach (1995) N Engl J Med, 332, pp. 1229-1232; Dranove, D., Kessler, D., McClellan, M., Satterthwaite, M., Is more information better? The effects of "Report Cards" on health care providers (2003) Journal of Political Economy, 111 (3), pp. 555-588. , DOI 10.1086/374180; Jones, R.H., In search of the optimal surgical mortality (1989) Circulation, 79, pp. I132-I136; Omoigui, N.A., Miller, D.P., Brown, K.J., Annan, K., Cosgrove III, D., Lytle, B., Loop, F., Topol, E.J., Outmigration for coronary bypass surgery in an era of public dissemination of clinical outcomes (1996) Circulation, 93 (1), pp. 27-33; Schneider, E.C., Epstein, A.M., Influence of cardiac-surgery performance reports on referral practices and access to care: A survey of cardiovascular specialists (1996) New England Journal of Medicine, 335 (4), pp. 251-256. , DOI 10.1056/NEJM199607253350406; Iezzoni, L.I., (2003) Risk Adjustment for Measuring Health Care Outcomes, , Chicago Ill: Health Administration Press; Kassirer, J.P., The use and abuse of practice profiles (1994) New England Journal of Medicine, 330 (9), pp. 634-636. , DOI 10.1056/NEJM199403033300910; Krumholz, H.M., Brindis, R.G., Brush, J.E., Cohen, D.J., Epstein, A.J., Furie, K., Howard, G., Normand, S.-L.T., Standards for statistical models used for public reporting of health outcomes: An American Heart Association scientific statement from the Quality of Care and Outcomes Research Interdisciplinary Writing Group (2006) Circulation, 113 (3), pp. 456-462. , DOI 10.1161/CIRCULATIONAHA.105.170769, PII 0000301720060124000019; Selker, H.P., Systems for comparing actual and predicted mortality rates: Characteristics to promote cooperation in improving hospital care (1993) Annals of Internal Medicine, 118 (10), pp. 820-822; Austin, P.C., Alter, D.A., Tu, J.V., The Use of Fixed- and Random-Effects Models for Classifying Hospitals as Mortality Outliers: A Monte Carlo Assessment (2003) Medical Decision Making, 23 (6), pp. 526-539. , DOI 10.1177/0272989X03258443; Christiansen, C.L., Morris, C.N., Improving the statistical approach to health care provider profiling (1997) Annals of Internal Medicine, 127 (SUPPL.), pp. 764-768; Goldstein, H., Spiegelhalter, D.J., League tables and their limitations: Statistical issues in comparisons of institutional performance (1996) Journal of the Royal Statistical Society. Series A: Statistics in Society, 159 (3), pp. 385-443; Localio, A.R., Hamory, B.H., Fisher, A.C., Tenhave, T.R., The Public Release of Hospital and Physician Mortality Data in Pennsylvania: A Case Study (1997) Medical Care, 35 (3), pp. 272-286; Normand, S.-L., Glickman, M., Gatsonis, C., Statistical methods for profiling providers of medical care: Issues and applications (1997) J Am Stat Assoc, 92, pp. 803-814; Normand, S.-L., Shahian, D., Statistical and clinical aspects of hospital outcomes profiling (2007) Statistical Science, 22, pp. 206-226; Shahian, D.M., Blackstone, E.H., Edwards, F.H., Grover, F.L., Grunkemeier, G.L., Naftel, D.C., Nashef, S.A.M., Peterson, E.D., Cardiac surgery risk models: A position article (2004) Annals of Thoracic Surgery, 78 (5), pp. 1868-1877. , DOI 10.1016/j.athoracsur.2004.05.054, PII S0003497504011968; Shahian, D.M., Normand, S.L., Comparison of "risk-adjusted" hospital outcomes (2008) Circulation, 117, pp. 1955-1963; (2006) Performance Measure: Accelerating Improvement, , Institute of Medicine Washington, DC: The National Academies Press; O'Brien, S.M., Shahian, D.M., DeLong, E.R., Normand, S.-L.T., Edwards, F.H., Ferraris, V.A., Haan, C.K., Peterson, E.D., Quality Measurement in Adult Cardiac Surgery: Part 2-Statistical Considerations in Composite Measure Scoring and Provider Rating (2007) Annals of Thoracic Surgery, 83 (SUPPL.), pp. S13-S26. , DOI 10.1016/j.athoracsur.2007.01.055, PII S0003497507002251; Clark, R.E., Outcome as a function of annual coronary artery bypass graft volume (1996) Annals of Thoracic Surgery, 61 (1), pp. 21-26. , DOI 10.1016/0003-4975(95)00734-2; Grumbach, K., Anderson, G.M., Luft, H.S., Regionalization of cardiac surgery in the United States and Canada. Geographic access, choice, and outcomes (1995) JAMA, 274, pp. 1282-1288; Hannan, E.L., Kilburn Jr., H., Bernard, H., Coronary artery bypass surgery: The relationship between inhospital mortality rate and surgical volume after controlling for clinical risk factors (1991) Med Care, 29, pp. 1094-1107; Hannan, E.L., Wu, C., Ryan, T.J., Bennett, E., Culliford, A.T., Gold, J.P., Hartman, A., Subramanian, V.A., Do hospitals and surgeons with higher coronary artery bypass graft surgery volumes still have lower risk-adjusted mortality rates? (2003) Circulation, 108 (7), pp. 795-801. , DOI 10.1161/01.CIR.0000084551.52010.3B; Kalant, N., Shrier, I., Volume and outcome of coronary artery bypass graft surgery: Are more and less the same? (2004) Canadian Journal of Cardiology, 20 (1), pp. 81-86; Nallamothu, B.K., Saint, S., Ramsey, S.D., Hofer, T.P., Vijan, S., Eagle, K.A., The role of hospital volume in coronary artery bypass grafting: Is more always better? (2001) Journal of the American College of Cardiology, 38 (7), pp. 1923-1930. , DOI 10.1016/S0735-1097(01)01647-3, PII S0735109701016473; Peterson, E.D., Coombs, L.P., DeLong, E.R., Haan, C.K., Ferguson, T.B., Procedural Volume as a Marker of Quality for CABG Surgery (2004) Journal of the American Medical Association, 291 (2), pp. 195-201. , DOI 10.1001/jama.291.2.195; Rathore, S.S., Epstein, A.J., Volpp, K.G.M., Krumholz, H.M., Hospital Coronary Artery Bypass Graft Surgery Volume and Patient Mortality, 1998-2000 (2004) Annals of Surgery, 239 (1), pp. 110-117. , DOI 10.1097/01.sla.0000103066.22732.b8; Showstack, J.A., Rosenfeld, K.E., Garnick, D.W., Association of volume with outcome of coronary artery bypass graft surgery. Scheduled vs nonscheduled operations (1987) Journal of the American Medical Association, 257 (6), pp. 785-789. , DOI 10.1001/jama.257.6.785; Shroyer, A.L.W., Marshall, G., Warner, B.A., Johnson, R.R., Guo, W., Grover, F.L., Hammermeister, K.E., No continuous relationship between veterans affairs hospital coronary artery bypass grafting surgical volume and operative mortality (1996) Annals of Thoracic Surgery, 61 (1), pp. 17-20. , DOI 10.1016/0003-4975(95)00830-6; Sowden, A.J., Deeks, J.J., Sheldon, T.A., Volume and outcome in coronary artery bypass graft surgery: True association or artefact? (1995) BMJ, 311, pp. 151-155; Welke, K.F., Barnett, M.J., Vaughan Sarrazin, M.S., Rosenthal, G.E., Limitations of hospital volume as a measure of quality of care for coronary artery bypass graft surgery (2005) Annals of Thoracic Surgery, 80 (6), pp. 2114-2119. , DOI 10.1016/j.athoracsur.2005.05.017, PII S0003497505009100; Wu, C., Hannan, E.L., Ryan, T.J., Bennett, E., Culliford, A.T., Gold, J.P., Isom, O.W., Subramanian, V.A., Is the impact of hospital and surgeon volumes on the in-hospital mortality rate for coronary artery bypass graft surgery limited to patients at high risk? (2004) Circulation, 110 (7), pp. 784-789. , DOI 10.1161/01.CIR.0000138744.13516.B5; Flood, A.B., Scott, W.R., Ewy, W., Does practice make perfect? Part I: The relation between hospital volume and outcomes for selected diagnostic categories (1984) Medical Care, 22 (2), pp. 98-114; Hannan, E.L., Kilburn Jr., H., O'Donnell, J.F., A longitudinal analysis of the relationship between in-hospital mortality in New York State and the volume of abdominal aortic aneurysm surgeries performed (1992) Health Serv Res, 27, pp. 517-542; Luft, H.S., Bunker, J.P., Enthoven, A.C., Should operations be regionalized? The empirical relation between surgical volume and mortality (1979) New England Journal of Medicine, 301 (25), pp. 1364-1369; Halm, E.A., Lee, C., Chassin, M.R., Is volume related to outcome in health care? A systematic review and methodologic critique of the literature (2002) Annals of Internal Medicine, 137 (6), pp. 511-520; Hughes, C.M., Influence of hospital volume on mortality following major cancer surgery (1999) JAMA, 281, p. 1375; Hughes, R.G., Hunt, S.S., Luft, H.S., Effects of surgeon volume and hospital volume on quality of care in hospitals (1987) Medical Care, 25 (6), pp. 489-503. , DOI 10.1097/00005650-198706000-00004; Dudley, R.A., Johansen, K.L., Brand, R., Rennie, D.J., Milstein, A., Selective referral to high-volume hospitals: Estimating potentially avoidable deaths (2000) Journal of the American Medical Association, 283 (9), pp. 1159-1166; Birkmeyer, J.D., Sun, Y., Goldfaden, A., Birkmeyer, N.J.O., Stukel, T.A., Volume and process of care in high-risk cancer surgery (2006) Cancer, 106 (11), pp. 2476-2481. , DOI 10.1002/cncr.21888; Birkmeyer, J.D., Finlayson, S.R.G., Tosteson, A.N.A., Sharp, S.M., Warshaw, A.L., Fisher, E.S., Effect of hospital volume on in-hospital mortality with pancreaticoduodenectomy (1999) Surgery, 125 (3), pp. 250-256; Birkmeyer, J.D., Warshaw, A.L., Finlayson, S.R.G., Grove, M.R., Tosteson, A.N.A., Relationship between hospital volume and late survival after pancreaticoduodenectomy (1999) Surgery, 126 (2), pp. 178-183. , DOI 10.1016/S0039-6060(99)70152-2; Birkmeyer, J.D., High-risk surgery-follow the crowd (2000) JAMA, 283, pp. 1191-1193; Birkmeyer, J.D., Lucas, F.L., De, W., Potential benefits of regionalizing major surgery in Medicare patients (1999) Eff Clin Pract, 2, pp. 277-283; Birkmeyer, J.D., Should we regionalize major surgery? Potential benefits and policy considerations (2000) Journal of the American College of Surgeons, 190 (3), pp. 341-349. , DOI 10.1016/S1072-7515(99)00270-7, PII S1072751599002707; Birkmeyer, J.D., Siewers, A.E., Finlayson, E.V.A., Stukel, T.A., Lucas, F.L., Batista, I., Welch, H.G., Wennberg, D.E., Hospital volume and surgical mortality in the United States (2002) New England Journal of Medicine, 346 (15), pp. 1128-1137. , DOI 10.1056/NEJMsa012337; Birkmeyer, J.D., Stukel, T.A., Siewers, A.E., Goodney, P.P., Wennberg, D.E., Lucas, F.L., Surgeon Volume and Operative Mortality in the United States (2003) New England Journal of Medicine, 349 (22), pp. 2117-2127. , DOI 10.1056/NEJMsa035205; Chang, A.C., Birkmeyer, J.D., The volume-performance relationship in esophagectomy (2006) Thorac Surg Clin, 16, pp. 87-94; Hollenbeck, B.K., Wei, Y., Birkmeyer, J.D., Volume, Process of Care, and Operative Mortality for Cystectomy for Bladder Cancer (2007) Urology, 69 (5), pp. 871-875. , DOI 10.1016/j.urology.2007.01.040, PII S0090429507001033; Hannan, E.L., The relation between volume and outcome in health care (1999) New England Journal of Medicine, 340 (21), pp. 1677-1679. , DOI 10.1056/NEJM199905273402112; Hannan, E.L., Radzyner, M., Rubin, D., Dougherty, J., Brennan, M.F., The influence of hospital and surgeon volume on in-hospital mortality for colectomy, gastrectomy, and lung lobectomy in patients with cancer (2002) Surgery, 131 (1), pp. 6-15. , DOI 10.1067/msy.2002.120238; Hannan, E.L., Wu, C., Walford, G., King III, S.B., Holmes Jr., D.R., Ambrose, J.A., Sharma, S., Jones, R.H., Volume-outcome relationships for percutaneous coronary interventions in the stent era (2005) Circulation, 112 (8), pp. 1171-1179. , DOI 10.1161/CIRCULATIONAHA.104.528455; Hannan, E.L., Racz, M., Ryan, T.J., McCallister, B.D., Johnson, L.W., Arani, D.T., Guerci, A.D., Topol, E.J., Coronary angioplasty volume-outcome relationships for hospitals and cardiologists (1997) Journal of the American Medical Association, 277 (11), pp. 892-898; Hannan, E.L., Popp, A.J., Tranmer, B., Fuestel, P., Waldman, J., Shah, D., Relationship between provider volume and mortality for carotid endarterectomies in New York State (1998) Stroke, 29 (11), pp. 2292-2297; Shahian, D.M., Normand, S.-L.T., The volume-outcome relationship: From luft to leapfrog (2003) Annals of Thoracic Surgery, 75 (3), pp. 1048-1058. , DOI 10.1016/S0003-4975(02)04308-4, PII S0003497502043084; Birkmeyer, J.D., Finlayson, E.V.A., Birkmeyer, C.M., Volume standards for high-risk surgical procedures: Potential benefits of the Leapfrog initiative (2001) Surgery, 130 (3), pp. 415-422. , DOI 10.1067/msy.2001.117139; Halm, E., Lee, C., Chassin, M., How is volume related to quality in health care? A systematic review of the research literature (2000) Interpreting the Volume-outcome Relationship in the Context of Health Care Quality: Workshop Summary, , Hewitt M, editor Washington, DC: National Academy Press; Luft, H., Garnick, D., Mark, D., (1990) Hospital Volume Physician Volume and Patient Outcomes: Assessing the Evidence, , Ann Harbor MI: Health Administration Press; Hewitt, M., (2000) Interpreting the Volume-outcome Relationship in the Context of Health Care Quality: Workshop Summary, , Washington DC: National Academy Press; Urbach, D.R., Austin, P.C., Conventional models overestimate the statistical significance of volume-outcome associations, compared with multilevel models (2005) Journal of Clinical Epidemiology, 58 (4), pp. 391-400. , DOI 10.1016/j.jclinepi.2004.12.001; Shahian, D.M., Improving Cardiac Surgery Quality - Volume, Outcome, Process? (2004) Journal of the American Medical Association, 291 (2), pp. 246-248. , DOI 10.1001/jama.291.2.246; Shahian, D.M., Normand, S.-L.T., Low-volume coronary artery bypass surgery: Measuring and optimizing performance (2008) Journal of Thoracic and Cardiovascular Surgery, 135 (6), pp. 1202-1209. , DOI 10.1016/j.jtcvs.2007.12.037, PII S0022522308002171; Spiegelhalter, D.J., Funnel plots for comparing institutional performance (2005) Statistics in Medicine, 24 (8), pp. 1185-1202. , DOI 10.1002/sim.1970; De Leval, M.R., Francois, K., Bull, C., Analysis of a cluster of surgical failures. Application to a series of neonatal arterial switch operations (1994) J Thorac Cardiovasc Surg, 107, pp. 914-923; Grigg, O.A., Farewell, V.T., Spiegelhalter, D.J., Use of risk-adjusted CUSUM and RSPRT charts for monitoring in medical contexts (2003) Statistical Methods in Medical Research, 12 (2), pp. 147-170; Spiegelhalter, D., Grigg, O., Kinsman, R., Treasure, T., Risk-adjusted sequential probability ratio tests: Applications to Bristol, Shipman and adult cardiac surgery (2003) International Journal for Quality in Health Care, 15 (1), pp. 7-13. , DOI 10.1093/intqhc/15.1.7; Luft, H.S., Better for whom? Policy implications of acting on the relation between volume and outcome in coronary artery bypass grafting (2001) Journal of the American College of Cardiology, 38 (7), pp. 1931-1933. , DOI 10.1016/S0735-1097(01)01636-9, PII S0735109701016369; Selim, M., Perioperative stroke (2007) N Engl J Med, 356, pp. 706-713; McKhann, G.M., Goldsborough, M.A., Borowicz Jr., L.M., Mellits, E.D., Brookmeyer, R., Quaskey, S.A., Baumgartner, W.A., Gardner, T.J., Predictors of stroke risk in coronary artery bypass patients (1997) Annals of Thoracic Surgery, 63 (2), pp. 516-521. , DOI 10.1016/S0003-4975(97)83384-X, PII S0003497596010570; Filsoufi, F., Rahmanian, P.B., Castillo, J.G., Bronster, D., Adams, D.H., Incidence, Topography, Predictors and Long-Term Survival After Stroke in Patients Undergoing Coronary Artery Bypass Grafting (2008) Annals of Thoracic Surgery, 85 (3), pp. 862-870. , DOI 10.1016/j.athoracsur.2007.10.060, PII S000349750702190X; Tarakji, K.G., Sabik Iii, J.F., Bhudia, S.K., Temporal onset, risk factors, and outcomes associated with stroke after coronary artery bypass grafting (2011) JAMA, 305, pp. 381-390; Gottesman, R.F., Sherman, P.M., Grega, M.A., Yousem, D.M., Borowicz Jr., L.M., Selnes, O.A., Baumgartner, W.A., McKhann, G.M., Watershed strokes after cardiac surgery: Diagnosis, etiology, and outcome (2006) Stroke, 37 (9), pp. 2306-2311. , DOI 10.1161/01.STR.0000236024.68020.3a, PII 0000767020060900000033; Caplan, L.R., Hennerici, M., Impaired clearance of emboli (washout) is an important link between hypoperfusion, embolism, and ischemic stroke (1998) Archives of Neurology, 55 (11), pp. 1475-1482; Roach, G.W., Kanchuger, M., Mangano, C.M., Multicenter Study of Perioperative Ischemia Research Group and the Ischemia Research and Education Foundation Investigators. Adverse cerebral outcomes after coronary bypass surgery (1996) N Engl J Med, 335, pp. 1857-1863; Legare, J.-F., Buth, K.J., King, S., Wood, J., Sullivan, J.A., Friesen, C.H., Lee, J., Hirsch, G.M., Coronary Bypass Surgery Performed off Pump Does Not Result in Lower In-Hospital Morbidity Than Coronary Artery Bypass Grafting Performed on Pump (2004) Circulation, 109 (7), pp. 887-892. , DOI 10.1161/01.CIR.0000115943.41814.7D; Muneretto, C., Bisleri, G., Negri, A., Off-pump coronary artery bypass surgery technique for total arterial myocardial revascularization: A prospective randomized study (2003) Ann Thorac Surg, 76, pp. 778-782; Nakamura, M., Okamoto, F., Nakanishi, K., Does intensive management of cerebral hemodynamics and atheromatous aorta reduce stroke after coronary artery surgery? (2008) Ann Thorac Surg, 85, pp. 513-519; Rosenberger, P., Shernan, S.K., Loffler, M., The influence of epiaortic ultrasonography on intraoperative surgical management in 6051 cardiac surgical patients (2008) Ann Thorac Surg, 85, pp. 548-553; Yamaguchi, A., Adachi, H., Tanaka, M., Efficacy of intraoperative epiaortic ultrasound scanning for preventing stroke after coronary artery bypass surgery (2009) Ann Thorac Cardiovasc Surg, 15, pp. 98-104; Van Linden, J.D., Hadjinikolaou, L., Bergman, P., Lindblom, D., Postoperative stroke in cardiac surgery is related to the location and extent of atherosclerotic disease in the ascending aorta (2001) Journal of the American College of Cardiology, 38 (1), pp. 131-135. , DOI 10.1016/S0735-1097(01)01328-6, PII S0735109701013286; Suvarna, S., Smith, A., Stygall, J., Kolvecar, S., Walesby, R., Harrison, M., Newman, S., An Intraoperative Assessment of the Ascending Aorta: A Comparison of Digital Palpation, Transesophageal Echocardiography, and Epiaortic Ultrasonography (2007) Journal of Cardiothoracic and Vascular Anesthesia, 21 (6), pp. 805-809. , DOI 10.1053/j.jvca.2007.05.014, PII S1053077007001693; Sylivris, S., Calafiore, P., Matalanis, G., Rosalion, A., Yuen, H.P., Buxton, B.F., Tonkin, A.M., The intraoperative assessment of ascending aortic atheroma: Epiaortic imaging is superior to both transesophageal echocardiography and direct palpation (1997) Journal of Cardiothoracic and Vascular Anesthesia, 11 (6), pp. 704-707. , DOI 10.1016/S1053-0770(97)90161-0; Goto, T., Baba, T., Matsuyama, K., Honma, K., Ura, M., Koshiji, T., Aortic atherosclerosis and postoperative neurological dysfunction in elderly coronary surgical patients (2003) Annals of Thoracic Surgery, 75 (6), pp. 1912-1918. , DOI 10.1016/S0003-4975(03)00029-8, PII S0003497503000298; Hangler, H.B., Nagele, G., Danzmayr, M., Mueller, L., Ruttmann, E., Laufer, G., Bonatti, J., Modification of surgical technique for ascending aortic atherosclerosis: Impact on stroke reduction in coronary artery bypass grafting (2003) Journal of Thoracic and Cardiovascular Surgery, 126 (2), pp. 391-400. , DOI 10.1016/S0022-5223(03)00395-7; Schachner, T., Nagele, G., Kacani, A., Laufer, G., Bonatti, J., Factors associated with presence of ascending aortic atherosclerosis in CABG patients (2004) Annals of Thoracic Surgery, 78 (6), pp. 2028-2032. , DOI 10.1016/j.athoracsur.2004.04.078, PII S0003497504010215; Gold, J.P., Torres, K.E., Maldarelli, W., Zhuravlev, I., Condit, D., Wasnick, J., Improving outcomes in coronary surgery: The impact of echo-directed aortic cannulation and perioperative hemodynamic management in 500 patients (2004) Annals of Thoracic Surgery, 78 (5), pp. 1579-1585. , DOI 10.1016/j.athoracsur.2004.05.051, PII S0003497504011932; Zingone, B., Rauber, E., Gatti, G., Pappalardo, A., Benussi, B., Dreas, L., Lattuada, L., The impact of epiaortic ultrasonographic scanning on the risk of perioperative stroke (2006) European Journal of Cardio-thoracic Surgery, 29 (5), pp. 720-728. , DOI 10.1016/j.ejcts.2006.02.001, PII S101079400600145X; Durand, D.J., Perler, B.A., Roseborough, G.S., Grega, M.A., Borowicz Jr., L.M., Baumgartner, W.A., Yuh, D.D., Mandatory versus selective preoperative carotid screening: A retrospective analysis (2004) Annals of Thoracic Surgery, 78 (1), pp. 159-166. , DOI 10.1016/j.athoracsur.2004.02.024, PII S0003497504004011; Sheiman, R.G., Janne D'Othee, B., Screening carotid sonography before elective coronary artery bypass graft surgery: Who needs it [published correction appears in Am J Roentgenol 2007;189:512] (2007) Am J Roentgenol, 188, pp. W475-W79; Naylor, A.R., Mehta, Z., Rothwell, P.M., Bell, P.R.F., Carotid artery disease and stroke during coronary artery bypass: A critical review of the literature (2002) European Journal of Vascular and Endovascular Surgery, 23 (4), pp. 283-294. , DOI 10.1053/ejvs.2002.1609; Endarterectomy for asymptomatic carotid artery stenosis (1995) JAMA, 273, pp. 1421-1428. , Executive Committee for the Asymptomatic Carotid Atherosclerosis Study; Evans, B.A., Wijdicks, E.F.M., High-grade carotid stenosis detected before general surgery: Is endarterectomy indicated? (2001) Neurology, 57 (7), pp. 1328-1330; Mortaz, H., Mostafazadeh, D., Sahraian, M., Carotid endarterectomy for carotid stenosis in patients selected for coronary artery bypass graft surgery (Review) (2009) Cochrane Database Syst Rev, pp. CD006074; Wijns, W., Kolh, P., Danchin, N., Guidelines on myocardial revascularization: The task force on myocardial revascularization of the european society of cardiology (ESC) and the European association for cardio-thoracic surgery (EACTS) (2010) Eur Heart J, 31, pp. 2501-2555; Cywinski, J.B., Koch, C.G., Krajewski, L.P., Smedira, N., Li, L., Starr, N.J., Increased Risk Associated With Combined Carotid Endarterectomy and Coronary Artery Bypass Graft Surgery: A Propensity-Matched Comparison With Isolated Coronary Artery Bypass Graft Surgery (2006) Journal of Cardiothoracic and Vascular Anesthesia, 20 (6), pp. 796-802. , DOI 10.1053/j.jvca.2006.01.022, PII S1053077006000565; Bucerius, J., Gummert, J.F., Borger, M.A., Walther, T., Doll, N., Falk, V., Schmitt, D.V., Mohr, F.W., Predictors of delirium after cardiac surgery delirium: Effect of beating-heart (off-pump) surgery (2004) Journal of Thoracic and Cardiovascular Surgery, 127 (1), pp. 57-64. , DOI 10.1016/S0022-5223(03)01281-9; Gottesman, R.F., Ma, G., Bailey, M.M., Delirium after coronary artery bypass graft surgery and late mortality (2010) Ann Neurol, 67, pp. 338-344; Rudolph, J.L., Jones, R.N., Rasmussen, L.S., Silverstein, J.H., Inouye, S.K., Marcantonio, E.R., Independent Vascular and Cognitive Risk Factors for Postoperative Delirium (2007) American Journal of Medicine, 120 (9), pp. 807-813. , DOI 10.1016/j.amjmed.2007.02.026, PII S0002934307004494; Veliz-Reissmuller, G., Torres, H.A., Van Der Linden, J., Lindblom, D., Jonhagen, M.E., Pre-operative mild cognitive dysfunction predicts risk for post-operative delirium after elective cardiac surgery (2007) Aging - Clinical and Experimental Research, 19 (3), pp. 172-177; Rudolph, J.L., Babikian, V.L., Treanor, P., Microemboli are not associated with delirium after coronary artery bypass graft surgery (2009) Perfusion, 24, pp. 409-415; Hudetz, J.A., Iqbal, Z., Gandhi, S.D., Postoperative delirium and short-term cognitive dysfunction occur more frequently in patients undergoing valve surgery with or without coronary artery bypass graft surgery compared with coronary artery bypass graft surgery alone: Results of a pilot study (2011) J Cardiothorac Vasc Anesth, 25, pp. 811-816; Rudolph, J.L., Inouye, S.K., Jones, R.N., Delirium: An independent predictor of functional decline after cardiac surgery (2010) J Am Geriatr Soc, 58, pp. 643-649; Andrew, M.J., Baker, R.A., Bennetts, J., Kneebone, A.C., Knight, J.L., A comparison of neuropsychologic deficits after extracardiac and intracardiac surgery (2001) Journal of Cardiothoracic and Vascular Anesthesia, 15 (1), pp. 9-14. , DOI 10.1053/jcan.2001.20210; Fearn, S.J., Pole, R., Wesnes, K., Faragher, E.B., Hooper, T.L., McCollum, C.N., Cerebral injury during cardiopulmonary bypass: Emboli impair memory (2001) Journal of Thoracic and Cardiovascular Surgery, 121 (6), pp. 1150-1160. , DOI 10.1067/mtc.2001.114099; Raymond, P.D., Hinton-Bayre, A.D., Radel, M., Ray, M.J., Marsh, N.A., Assessment of statistical change criteria used to define significant change in neuropsychological test performance following cardiac surgery (2006) European Journal of Cardio-thoracic Surgery, 29 (1), pp. 82-88. , DOI 10.1016/j.ejcts.2005.10.016, PII S101079400500775X; Selnes, O.A., Goldsborough, M.A., Borowicz Jr., L.M., Enger, C., Quaskey, S.A., McKhann, G.M., Determinants of cognitive change after coronary artery bypass surgery: A multifactorial problem (1999) Annals of Thoracic Surgery, 67 (6), pp. 1669-1676. , DOI 10.1016/S0003-4975(99)00258-1, PII S0003497599002581; Selnes, O.A., Pham, L., Zeger, S., McKhann, G.M., Defining Cognitive Change After CABG: Decline Versus Normal Variability (2006) Annals of Thoracic Surgery, 82 (2), pp. 388-390. , DOI 10.1016/j.athoracsur.2006.02.060, PII S0003497506004024; Johnson, T., Monk, T., Rasmussen, L.S., Abildstrom, H., Houx, P., Korttila, K., Kuipers, H.M., Moller, J.T., Postoperative cognitive dysfunction in middle-aged patients (2002) Anesthesiology, 96 (6), pp. 1351-1357. , DOI 10.1097/00000542-200206000-00014; Monk, T.G., Weldon, B.C., Garvan, C.W., Predictors of cognitive dysfunction after major noncardiac surgery (2008) Anesthesiology, 108, pp. 18-30; Rasmussen, L.S., Moller, J.T., Central nervous system dysfunction after anesthesia in the geriatric patient (2000) Anesthesiology Clinics of North America, 18 (1), pp. 59-70; Ho, P.M., Arciniegas, D.B., Grigsby, J., McCarthy Jr., M., McDonald, G.O., Moritz, T.E., Shroyer, A.L., Hammermeister, K.E., Predictors of cognitive decline following coronary artery bypass graft surgery (2004) Annals of Thoracic Surgery, 77 (2), pp. 597-603. , DOI 10.1016/S0003-4975(03)01358-4; Goto, T., Baba, T., Honma, K., Shibata, Y., Arai, Y., Uozumi, H., Okuda, T., Magnetic resonance imaging findings and postoperative neurologic dysfunction in elderly patients undergoing coronary artery bypass grafting (2001) Annals of Thoracic Surgery, 72 (1), pp. 137-142. , DOI 10.1016/S0003-4975(01)02676-5, PII S0003497501026765; Takagi, H., Tanabashi, T., Kawai, N., A meta-analysis of minimally invasive coronary artery bypass versus percutaneous coronary intervention with stenting for isolated left anterior descending artery disease is indispensable (2007) J Thorac Cardiovasc Surg, 134, pp. 548-549; Marasco, S.F., Sharwood, L.N., Abramson, M.J., No improvement in neurocognitive outcomes after off-pump versus on-pump coronary revascularisation: A meta-analysis (2008) Eur J Cardiothorac Surg, 33, pp. 961-970; Rosengart, T.K., Sweet, J.J., Finnin, E., Wolfe, P., Cashy, J., Hahn, E., Marymont, J., Sanborn, T., Stable Cognition After Coronary Artery Bypass Grafting: Comparisons With Percutaneous Intervention and Normal Controls (2006) Annals of Thoracic Surgery, 82 (2), pp. 597-607. , DOI 10.1016/j.athoracsur.2006.03.026, PII S0003497506005339; Sweet, J.J., Finnin, E., Wolfe, P.L., Beaumont, J.L., Hahn, E., Marymont, J., Sanborn, T., Rosengart, T.K., Absence of Cognitive Decline One Year After Coronary Bypass Surgery: Comparison to Nonsurgical and Healthy Controls (2008) Annals of Thoracic Surgery, 85 (5), pp. 1571-1578. , DOI 10.1016/j.athoracsur.2008.01.090, PII S0003497508002373; Selnes, O.A., Ma, G., Borowicz Jr., L.M., Cognitive changes with coronary artery disease: A prospective study of coronary artery bypass graft patients and nonsurgical controls (2003) Ann Thorac Surg, 75, pp. 1377-1384; Newman, M.F., Kirchner, J.L., Phillips-Bute, B., Gaver, V., Grocott, H., Jones, R.H., Mark, D.B., Blumenthal, J.A., Longitudinal assessment of neurocognitive function after coronary-artery bypass surgery (2001) New England Journal of Medicine, 344 (6), pp. 395-402. , DOI 10.1056/NEJM200102083440601; Stygall, J., Newman, S.P., Fitzgerald, G., Steed, L., Mulligan, K., Arrowsmith, J.E., Pugsley, W., Harrison, M.J., Cognitive Change 5 Years after Coronary Artery Bypass Surgery (2003) Health Psychology, 22 (6), pp. 579-586. , DOI 10.1037/0278-6133.22.6.579; Selnes, O.A., Ma, G., Bailey, M.M., Cognition 6 years after surgical or medical therapy for coronary artery disease (2008) Ann Neurol, 63, pp. 581-590; Van Dijk, D., Spoor, M., Hijman, R., Nathoe, H.M., Borst, C., Jansen, E.W.L., Grobbee, D.E., Kalkman, C.J., Cognitive and cardiac outcomes 5 years after off-pump vs on-pump coronary artery bypass graft surgery (2007) Journal of the American Medical Association, 297 (7), pp. 701-708. , http://jama.ama-assn.org/cgi/reprint/297/7/701, DOI 10.1001/jama.297.7.701; Kreter, B., Woods, M., Antibiotic prophylaxis for cardiothoracic operations. Meta-analysis of thirty years of clinical trials (1992) J Thorac Cardiovasc Surg, 104, pp. 590-599; Goodman, J.S., Schaffner, W., Collins, H.A., Infection after cardiovascular surgery. Clinical study including examination of antimicrobial prophylaxis (1968) N Engl J Med, 278, pp. 117-123; Fong, I.W., Baker, C.B., McKee, D.C., The value of prophylactic antibiotics in aorta-coronary bypass operations. A double-blind randomized trial (1979) Journal of Thoracic and Cardiovascular Surgery, 78 (6), pp. 908-913; Fekety Jr., F.R., Cluff, L.E., Sabiston Jr., D.C., A study of antibiotic prophylaxis in cardiac surgery (1969) J Thorac Cardiovasc Surg, 57, pp. 757-763; Austin, T.W., Coles, J.C., Burnett, R., Goldbach, M., Aortocoronary bypass procedures and sternotomy infections: A study of antistaphylococcal prophylaxis (1980) Canadian Journal of Surgery, 23 (5), pp. 483-485; Kaiser, A.B., Petracek, M.R., Lea, J.W., Efficacy of cefazolin, cefamandole, and gentamicin as prophylactic agents in cardiac surgery. Results of a prospective, randomized, double-blind trial in 1030 patients (1987) Ann Surg, 206, pp. 791-797; Bolon, M.K., Morlote, M., Weber, S.G., Koplan, B., Carmeli, Y., Wright, S.B., Glycopeptides are no more effective than β-lactam agents for prevention of surgical site infection after cardiac surgery: A meta-analysis (2004) Clinical Infectious Diseases, 38 (10), pp. 1357-1363. , DOI 10.1086/383318; Finkelstein, R., Rabino, G., Mashiah, T., Bar-El, Y., Adler, Z., Kertzman, V., Cohen, O., Milo, S., Vancomycin versus cefazolin prophylaxis for cardiac surgery in the setting of a high prevalence of methicillin-resistant staphylococcal infections (2002) Journal of Thoracic and Cardiovascular Surgery, 123 (2), pp. 326-332. , DOI 10.1067/mtc.2002.119698; Maki, D.G., Bohn, M.J., Stolz, S.M., Comparative study of cefazolin, cefamandole, and vancomycin for surgical prophylaxis in cardiac and vascular operations. A double-blind randomized trial (1992) J Thorac Cardiovasc Surg, 104, pp. 1423-1434; Saginur, R., Croteau, D., Bergeron, M.G., Comparative efficacy of teicoplanin and cefazolin for cardiac operation prophylaxis in 3027 patients (2000) J Thorac Cardiovasc Surg, 120, pp. 1120-1130. , the ESPRIT Group; Salminen, U.-S., Viljanen, T.U.T., Valtonen, V.V., Ikonen, T.E.H., Sahlman, A.E., Harjula, A.L.J., Ceftriaxone versus vancomycin prophylaxis in cardiovascular surgery (1999) Journal of Antimicrobial Chemotherapy, 44 (2), pp. 287-290. , DOI 10.1093/jac/44.2.287; Townsend, T.R., Reitz, B.A., Bilker, W.B., Bartlett, J.G., Clinical trial of cefamandole, cefazolin, and cefuroxime for antibiotic prophylaxis in cardiac operations (1993) Journal of Thoracic and Cardiovascular Surgery, 106 (4), pp. 664-670; Vuorisalo, S., Pokela, R., Syrjala, H., Comparison of vancomycin and cefuroxime for infection prophylaxis in coronary artery bypass surgery (1998) Infection Control and Hospital Epidemiology, 19 (4), pp. 234-239; Wilson, A.P., Treasure, T., Gruneberg, R.N., Antibiotic prophylaxis in cardiac surgery: A prospective comparison of two dosage regimens of teicoplanin with a combination of flucloxacillin and tobramycin (1988) J Antimicrob Chemother, 21, pp. 213-223; Centers for diseases control and prevention. Recommendations for preventing the spread of vancomycin resistance. Recommendations of the hospital infection control practices advisory committee (2010) MMWR Morb Mortal Wkly Rep, 44, pp. 1-13; Spelman, D., Harrington, G., Russo, P., Wesselingh, S., Clinical, microbiological, and economic benefit of a change in antibiotic prophylaxis for cardiac surgery (2002) Infection Control and Hospital Epidemiology, 23 (7), pp. 402-404; Walsh, E.E., Greene, L., Kirshner, R., Sustained Reduction in Methicillin-Resistant Staphylococcus aureus Wound Infections after Cardiothoracic Surgery (2010) Arch Intern Med, 171, pp. 68-73; Jurkiewicz, M.J., Bostwick III, J., Hester, T.R., Infected median sternotomy wound. Successful treatment by muscle flaps (1980) Annals of Surgery, 191 (6), pp. 738-744; Rand, R.P., Cochran, R.P., Aziz, S., Prospective trial of catheter irrigation and muscle flaps for sternal wound infection (1998) Ann Thorac Surg, 65, pp. 1046-1049; Wong, C.H.K., Senewiratne, S., Garlick, B., Mullany, D., Two-stage management of sternal wound infection using bilateral pectoralis major advancement flap (2006) European Journal of Cardio-thoracic Surgery, 30 (1), pp. 148-152. , DOI 10.1016/j.ejcts.2006.03.049, PII S1010794006003599; Argenta, L.C., Morykwas, M.J., Vacuum-assisted closure: A new method for wound control and treatment: Clinical experience (1997) Ann Plast Surg, 38, pp. 563-576; Baillot, R., Cloutier, D., Montalin, L., Impact of deep sternal wound infection management with vacuum-assisted closure therapy followed by sternal osteosynthesis: A 15-year review of 23 499 sternotomies (2010) Eur J Cardiothorac Surg, 37, pp. 880-887; Cowan, K.N., Teague, L., Sue, S.C., Mahoney, J.L., Vacuum-assisted wound closure of deep sternal infections in high-risk patients after cardiac surgery (2005) Annals of Thoracic Surgery, 80 (6), pp. 2205-2212. , DOI 10.1016/j.athoracsur.2005.04.005, PII S0003497505006028; Doss, M., Martens, S., Wood, J.P., Wolff, J.D., Baier, C., Moritz, A., Vacuum-assisted suction drainage versus conventional treatment in the management of poststernotomy osteomyelitis (2002) European Journal of Cardio-thoracic Surgery, 22 (6), pp. 934-938. , DOI 10.1016/S1010-7940(02)00594-8, PII S1010794002005948; Ennker, I.C., Malkoc, A., Pietrowski, D., The concept of negative pressure wound therapy (NPWT) after poststernotomy mediastinitis-a single center experience with 54 patients (2009) J Cardiothorac Surg, 4, p. 5; Fleck, T., Moidl, R., Giovanoli, P., A conclusion from the first 125 patients treated with the vacuum assisted closure system for postoperative sternal wound infection (2006) Interact Cardiovasc Thorac Surg, 5, pp. 145-148; Fleck, T.M., Fleck, M., Moidl, R., Czerny, M., Koller, R., Giovanoli, P., Hiesmayer, M.J., Grabenwoger, M., The vacuum-assisted closure system for the treatment of deep sternal wound infections after cardiac surgery (2002) Annals of Thoracic Surgery, 74 (5), pp. 1596-1600. , DOI 10.1016/S0003-4975(02)03948-6, PII S0003497502039486; Luckraz, H., Murphy, F., Bryant, S., Charman, S.C., Ritchie, A.J., Vacuum-assisted closure as a treatment modality for infections after cardiac surgery (2003) Journal of Thoracic and Cardiovascular Surgery, 125 (2), pp. 301-305. , DOI 10.1067/mtc.2003.74; Sjogren, J., Gustafsson, R., Nilsson, J., Malmsjo, M., Ingemansson, R., Clinical outcome after poststernotomy mediastinitis: Vacuum-assisted closure versus conventional treatment (2005) Annals of Thoracic Surgery, 79 (6), pp. 2049-2055. , DOI 10.1016/j.athoracsur.2004.12.048, PII S0003497505000068; Sjogren, J., Nilsson, J., Gustafsson, R., Malmsjo, M., Ingemansson, R., The impact of vacuum-assisted closure on long-term survival after post-sternotomy mediastinitis (2005) Annals of Thoracic Surgery, 80 (4), pp. 1270-1275. , DOI 10.1016/j.athoracsur.2005.04.010, PII S0003497505006077; Furnary, A.P., Wu, Y., Eliminating the Diabetic Disadvantage: The Portland Diabetic Project (2006) Seminars in Thoracic and Cardiovascular Surgery, 18 (4), pp. 302-308. , DOI 10.1053/j.semtcvs.2006.04.005, PII S1043067906000165, Diabetes, Hyperglycemia, and Cardiac Surgery Patient; Kirdemir, P., Yildirim, V., Kiris, I., Does continuous insulin therapy reduce postoperative supraventricular tachycardia incidence after coronary artery bypass operations in diabetic patients? (2008) J Cardiothorac Vasc Anesth, 22, pp. 383-387; Bilgin, Y.M., Van De Watering, L.M.G., Eijsman, L., Versteegh, M.I.M., Brand, R., Van Oers, M.H.J., Brand, A., Double-blind, randomized controlled trial on the effect of leukocyte-depleted erythrocyte transfusions in cardiac valve surgery (2004) Circulation, 109 (22), pp. 2755-2760. , DOI 10.1161/01.CIR.0000130162.11925.21; Blumberg, N., Heal, J.M., Cowles, J.W., Hicks Jr., G.L., Risher, W.H., Samuel, P.K., Kirkley, S.A., Leukocyte-reduced transfusions in cardiac surgery: Results of an implementation trial (2002) American Journal of Clinical Pathology, 118 (3), pp. 376-381. , DOI 10.1309/79B7-2QWN-AG8W-HBHD; Romano, G., Mastroianni, C., Bancone, C., Leukoreduction program for red blood cell transfusions in coronary surgery: Association with reduced acute kidney injury and in-hospital mortality (2010) J Thorac Cardiovasc Surg, 140, pp. 188-195; Van De Watering, L.M.G., Hermans, J., Houbiers, J.G.A., Van den Broek, P.J., Bouter, H., Boer, F., Harvey, M.S., Brand, A., Beneficial effects of leukocyte depletion of transfused blood on postoperative complications in patients undergoing cardiac surgery: A randomized clinical trial (1998) Circulation, 97 (6), pp. 562-568; Konvalinka, A., Errett, L., Fong, I.W., Impact of treating Staphylococcus aureus nasal carriers on wound infections in cardiac surgery (2006) Journal of Hospital Infection, 64 (2), pp. 162-168. , DOI 10.1016/j.jhin.2006.06.010, PII S0195670106002982; Van Rijen, M., Bonten, M., Wenzel, R., Mupirocin ointment for preventing Staphylococcus aureus infections in nasal carriers (2008) Cochrane Database Syst Rev, pp. CD006216; Fletcher, N., Sofianos, D., Berkes, M.B., Obremskey, W.T., Prevention of perioperative infection (2007) Journal of Bone and Joint Surgery - Series A, 89 (7), pp. 1605-1618. , DOI 10.2106/JBJS.F.00901; Geelhoed, G.W., Sharpe, K., Simon, G.L., A comparative study of surgical skin preparation method (1983) Surgery Gynecology and Obstetrics, 157 (3), pp. 265-268; Kaiser, A.B., Kernodle, D.S., Barg, N.L., Petracek, M.R., Influence of preoperative showers on staphylococcal skin colonization: A comparative trial of antiseptic skin cleansers (1988) Annals of Thoracic Surgery, 45 (1), pp. 35-38; Risk factors for deep sternal wound infection after sternotomy: A prospective, multicenter study (1996) J Thorac Cardiovasc Surg, 111, pp. 1200-1207; Bratzler, D.W., Hunt, D.R., The surgical infection prevention and Surgical Care Improvement Projects: National initiatives to improve outcomes for patients having surgery (2006) Clinical Infectious Diseases, 43 (3), pp. 322-330. , DOI 10.1086/505220; Ko, W., Lazenby, W.D., Zelano, J.A., Effects of shaving methods and intraoperative irrigation on suppurative mediastinitis after bypass operations (1992) Ann Thorac Surg, 53, pp. 301-305; Nishida, H., Grooters, R.K., Soltanzadeh, H., Discriminate use of electrocautery on the median sternotomy incision. A 0.16% wound infection rate (1991) J Thorac Cardiovasc Surg, 101, pp. 488-494; Tanner, J., Woodings, D., Moncaster, K., Preoperative hair removal to reduce surgical site infection (2006) Cochrane Database Syst Rev, pp. CD004122; Nelson, D.R., Buxton, T.B., Luu, Q.N., Rissing, J.P., The promotional effect of bone wax on experimental Staphylococcus aureus osteomyelitis (1990) Journal of Thoracic and Cardiovascular Surgery, 99 (6), pp. 977-980; Bennett, B., Duff, P., The effect of double gloving on frequency of glove perforations (1991) Obstet Gynecol, 78, pp. 1019-1022; Berridge, D.C., Starky, G., Jones, N.A.G., Chamberlain, J., A randomized controlled trial of double- versus single-gloving in vascular surgery (1998) Journal of the Royal College of Surgeons of Edinburgh, 43 (1), pp. 9-10; Gani, J.S., Anseline, P.F., Bissett, R.L., Efficacy of double versus single gloving in protecting the operating team (1990) Australian and New Zealand Journal of Surgery, 60 (3), pp. 171-175; Webb, J.M., Pentlow, B.D., Double gloving and surgical technique (1993) Annals of the Royal College of Surgeons of England, 75 (4), pp. 291-292; Wong, P.S., Young, V.K., Youhana, A., Wright, J.E., Surgical glove punctures during cardiac operations (1993) Annals of Thoracic Surgery, 56 (1), pp. 108-110; Crabtree, T.D., Codd, J.E., Fraser, V.J., Bailey, M.S., Olsen, M.A., Damiano Jr., R.J., Multivariate analysis of risk factors for deep and superficial sternal infection after coronary artery bypass grafting at a tertiary care medical center (2004) Seminars in Thoracic and Cardiovascular Surgery, 16 (1), pp. 53-61; Edwards, F.H., Engelman, R.M., Houck, P., Shahian, D.M., Bridges, C.R., The society of thoracic surgeons practice guideline series: Antibiotic prophylaxis in cardiac surgery, part I: Duration (2006) Annals of Thoracic Surgery, 81 (1), pp. 397-404. , DOI 10.1016/j.athoracsur.2005.06.034, PII S0003497505010398; Ridderstolpe, L., Gill, H., Granfeldt, H., Ahlfeldt, H., Rutberg, H., Superficial and deep sternal wound complications: Incidence, risk factors and mortality (2001) European Journal of Cardio-thoracic Surgery, 20 (6), pp. 1168-1175. , DOI 10.1016/S1010-7940(01)00991-5, PII S1010794001009915; Milano, C.A., Kesler, K., Archibald, N., Mediastinitis after coronary artery bypass graft surgery. Risk factors and long-term survival (1995) Circulation, 92, pp. 2245-2251; Abboud, C.S., Wey, S.B., Baltar, V.T., Risk factors for mediastinitis after cardiac surgery (2004) Annals of Thoracic Surgery, 77 (2), pp. 676-683. , DOI 10.1016/S0003-4975(03)01523-6; Loop, F.D., Lytle, B.W., Cosgrove, D.M., J. Maxwell Chamberlain memorial paper. Sternal wound complications after isolated coronary artery bypass grafting: Early and late mortality, morbidity, and cost of care (1990) Ann Thorac Surg, 49, pp. 179-186; Braxton, J.H., Marrin, C.A.S., McGrath, P.D., Morton, J.R., Norotsky, M., Charlesworth, D.C., Lahey, S.J., O'Connor, G.T., 10-Year follow-up of patients with and without mediastinitis (2004) Seminars in Thoracic and Cardiovascular Surgery, 16 (1), pp. 70-76; Ma, B., Rao, V., Weisel, R.D., Deep sternal wound infection: Risk factors and outcomes (1998) Ann Thorac Surg, 65, pp. 1050-1056; Stahle, E., Tammelin, A., Bergstrom, R., Hambreus, A., Nystrom, S.O., Hansson, H.E., Sternal wound complications - Incidence, microbiology and risk factors (1997) European Journal of Cardio-thoracic Surgery, 11 (6), pp. 1146-1153. , DOI 10.1016/S1010-7940(97)01210-4, PII S1010794097012104; Toumpoulis, I.K., Anagnostopoulos, C.E., DeRose Jr., J.J., Swistel, D.G., The impact of deep sternal wound infection on long-term survival after coronary artery bypass grafting (2005) Chest, 127 (2), pp. 464-471. , DOI 10.1378/chest.127.2.464; Filsoufi, F., Castillo, J.G., Rahmanian, P.B., Epidemiology of deep sternal wound infection in cardiac surgery (2009) J Cardiothorac Vasc Anesth, 23, pp. 488-494; Losanoff, J.E., Richman, B.W., Jones, J.W., Disruption and infection of median sternotomy: A comprehensive review (2002) European Journal of Cardio-thoracic Surgery, 21 (5), pp. 831-839. , DOI 10.1016/S1010-7940(02)00124-0, PII S1010794002001240; De Paulis, R., De Notaris, S., Scaffa, R., Nardella, S., Zeitani, J., Del Giudice, C., Penta De Peppo, A., Chiariello, L., The effect of bilateral internal thoracic artery harvesting on superficial and deep sternal infection: The role of skeletonization (2005) Journal of Thoracic and Cardiovascular Surgery, 129 (3), pp. 536-543. , DOI 10.1016/j.jtcvs.2004.07.059; Savage, E.B., Grab, J.D., O'Brien, S.M., Ali, A., Okum, E.J., Perez-Tamayo, R.A., Eiferman, D.S., Higgins, R.S.D., Use of Both Internal Thoracic Arteries in Diabetic Patients Increases Deep Sternal Wound Infection (2007) Annals of Thoracic Surgery, 83 (3), pp. 1002-1006. , DOI 10.1016/j.athoracsur.2006.09.094, PII S0003497506019205; Saso, S., James, D., Vecht, J.A., Effect of skeletonization of the internal thoracic artery for coronary revascularization on the incidence of sternal wound infection (2010) Ann Thorac Surg, 89, pp. 661-670; Murphy, G.J., Reeves, B.C., Rogers, C.A., Rizvi, S.I.A., Culliford, L., Angelini, G.D., Increased mortality, postoperative morbidity, and cost after red blood cell transfusion in patients having cardiac surgery (2007) Circulation, 116 (22), pp. 2544-2552. , DOI 10.1161/CIRCULATIONAHA.107.698977, PII 0000301720071127000009; Chelemer, S.B., Prato, B.S., Cox Jr., P.M., O'Connor, G.T., Morton, J.R., Association of bacterial infection and red blood cell transfusion after coronary artery bypass surgery (2002) Annals of Thoracic Surgery, 73 (1), pp. 138-142. , DOI 10.1016/S0003-4975(01)03308-2, PII S0003497501033082; Banbury, M.K., Brizzio, M.E., Rajeswaran, J., Lytle, B.W., Blackstone, E.H., Transfusion increases the risk of postoperative infection after cardiovascular surgery (2006) Journal of the American College of Surgeons, 202 (1), pp. 131-138. , DOI 10.1016/j.jamcollsurg.2005.08.028, PII S1072751505014304; Leal-Noval, S.R., Rincon-Ferrari, M.D., Garcia-Curiel, A., Herruzo-Aviles, A., Camacho-Larana, P., Garnacho-Montero, J., Amaya-Villar, R., Transfusion of blood components and postoperative infection in patients undergoing cardiac surgery (2001) Chest, 119 (5), pp. 1461-1468. , DOI 10.1378/chest.119.5.1461; Blanchard, A., Hurni, M., Ruchat, P., Incidence of deep and Superficial sternal infection after open heart surgery. A ten years retrospective study from 1981 to 1991 (1995) Eur J Cardiothorac Surg, 9, pp. 153-157; Risnes, I., Abdelnoor, M., Almd.

16. Epstein AE, Dimarco JP, Ellenbogen KA, Estes NAM, Freedman RA, Gettes LS, et al. 2012 ACCF/AHA/HRS focused update incorporated into the ACCF/AHA/HRS 2008 guidelines for device-based therapy of cardiac rhythm abnormalities: A report of the American college of cardiology foundation/american heart association task force on practice guidelines and the heart rhythm society. Circulation. 2013;127(3):e283-e352. doi: 10.1161/CIR.0b013e318276ce9b.

17. Tracy CM, Epstein AE, Darbar D, Dimarco JP, Dunbar SB, Estes Iii NAM, et al. 2012 ACCF/AHA/HRS focused update incorporated into the ACCF/AHA/HRS 2008 guidelines for device-based therapy of cardiac rhythm abnormalities: A report of the American college of cardiology foundation/American heart association task force on practice guidelines and the heart rhythm society. J Am Coll Cardiol. 2013;61(3):e6-e75. doi: 10.1016/j.jacc.2012.11.007.

18. Calkins H, Kuck KH, Cappato R, Brugada J, Camm AJ, Chen SA, et al. 2012 HRS/EHRA/ECAS expert consensus statement on catheter and surgical ablation of atrial fibrillation: Recommendations for patient selection, procedural techniques, patient management and follow-up, definitions, endpoints, and research trial design. Europace. 2012;14(4):528-606. doi: 10.1093/europace/eus027

10.1111/j.1540-8167.2011.02175.x; Gottlieb, I., Pinheiro, A., Brinker, J.A., Corretti, M.C., Mayer, S.A., Bluemke, D.A., Lima, J.A.C., Henrikson, C.A., Diagnostic accuracy of arterial phase 64-slice multidetector CT angiography for left atrial appendage thrombus in patients undergoing atrial fibrillation ablation (2008) Journal of Cardiovascular Electrophysiology, 19 (3), pp. 247-251. , DOI 10.1111/j.1540-8167.2007.01043.x; Saksena, S., Sra, J., Jordaens, L., A prospective comparison of cardiac imaging using intracardiac echocardiography with transesophageal echocardiography in patients with atrial fibrillation: The intracardiac echocardiography guided cardioversion helps interventional procedures study (2010) Circ Arrhythm Electrophysiol, 3 (6), pp. 571-577. , Dec 1; Patel, A., Au, E., Donegan, K., Multidetector row computed tomography for identification of left atrial appendage filling defects in patients undergoing pulmonary vein isolation for treatment of atrial fibrillation: Comparison with transesophageal echocardiography (2008) Heart Rhythm, 5 (2), pp. 253-260. , Feb; Asbach, S., Biermann, J., Bode, C., Faber, T.S., Early heparin administration reduces risk for left atrial thrombus formation during atrial fibrillation ablation procedures (2011) Cardiol Res Pract, 2011, p. 615087; Bruce, C.J., Friedman, P.A., Narayan, O., Early heparinization decreases the incidence of left atrial thrombi detected by intracardiac echocardiography during radiofrequency ablation for atrial fibrillation (2008) J Interv Card Electrophysiol, 22 (3), pp. 211-219. , Sep; Ren, J.F., Marchlinski, F.E., Callans, D.J., Increased intensity of anticoagulation may reduce risk of thrombus during atrial fibrillation ablation procedures in patients with spontaneous echo contrast (2005) J Cardiovasc Electrophysiol, 16 (5), pp. 474-477. , May; Chilukuri, K., Henrikson, C.A., Dalal, D., Incidence and outcomes of protamine reactions in patients undergoing catheter ablation of atrial fibrillation (2009) J Interv Card Electrophysiol, 25 (3), pp. 175-181. , Sep; Bunch, T.J., Crandall, B.G., Weiss, J.P., Warfarin is not needed in low-risk patients following atrial fibrillation ablation procedures (2009) J Cardiovasc Electrophysiol, 20 (9), pp. 988-993. , Sep; Bubien, R.S., Fisher, J.D., Gentzel, J.A., Murphy, E.K., Irwin, M.E., Shea, J.B., Dick II, M., MacGowan, J., NASPE expert consensus document: Use of IV (conscious) sedation/analgesia by nonanesthesia personnel in patients undergoing arrhythmia specific diagnostic, therapeutic, and surgical procedures (1998) PACE - Pacing and Clinical Electrophysiology, 21 (2), pp. 375-385. , DOI 10.1111/j.1540-8159.1998.tb00061.x; (2006) Statement on Granting Privileges for Administration of Moderate Sedation to Practitioners Who Are Not Anesthesia Professionals, , ASA. October; Kottkamp, H., Hindricks, G., Eitel, C., Deep sedation for catheter ablation of atrial fibrillation: A prospective study in 650 consecutive patients (2011) J Cardiovasc Electrophysiol, 22 (12), pp. 1339-1343. , Dec; Di Biase, L., Conti, S., Mohanty, P., General anesthesia reduces the prevalence of pulmonary vein reconnection during repeat ablation when compared with conscious sedation: Results from a randomized study (2011) Heart Rhythm, 8 (3), pp. 368-372. , Mar; Goode Jr., J.S., Taylor, R.L., Buffington, C.W., Klain, M.M., Schwartzman, D., High-frequency jet ventilation: Utility in posterior left atrial catheter ablation (2006) Heart Rhythm, 3 (1), pp. 13-19. , DOI 10.1016/j.hrthm.2005.09.013, PII S1547527105021053; Cummings, J.E., Schweikert, R.A., Saliba, W.I., Burkhardt, J.D., Kilikaslan, F., Saad, E., Natale, A., Brief communication: Atrial-esophageal fistulas after radiofrequency ablation (2006) Annals of Internal Medicine, 144 (8), pp. 572-574; Pappone, C., Oral, H., Santinelli, V., Vicedomini, G., Lang, C.C., Manguso, F., Torracca, L., Morady, F., Atrio-esophageal fistula as a complication of percutaneous transcatheter ablation of atrial fibrillation (2004) Circulation, 109 (22), pp. 2724-2726. , DOI 10.1161/01.CIR.0000131866.44650.46; Shah, D., Dumonceau, J.-M., Burri, H., Sunthorn, H., Schroft, A., Gentil-Baron, P., Yokoyama, Y., Takahashi, A., Acute pyloric spasm and gastric hypomotility: An extracardiac adverse effect of percutaneous radiofrequency ablation for atrial fibrillation (2005) Journal of the American College of Cardiology, 46 (2), pp. 327-330. , DOI 10.1016/j.jacc.2005.04.030, PII S0735109705009836; Ahmed, H., Neuzil, P., D'Avila, A., The esophageal effects of cryoenergy during cryoablation for atrial fibrillation (2009) Heart Rhythm, 6 (7), pp. 962-969. , Jul; Lemola, K., Sneider, M., Desjardins, B., Case, I., Han, J., Good, E., Tamirisa, K., Oral, H., Computed tomographic analysis of the anatomy of the left atrium and the esophagus: Implications for left atrial catheter ablation (2004) Circulation, 110 (24), pp. 3655-3660. , DOI 10.1161/01.CIR.0000149714.31471.FD; Kottkamp, H., Piorkowski, C., Tanner, H., Kobza, R., Dorszewski, A., Schirdewahn, P., Gerds-Li, J.-H., Hindricks, G., Topographic variability of the esophageal left atrial relation influencing ablation lines in patients with atrial fibrillation (2005) Journal of Cardiovascular Electrophysiology, 16 (2), pp. 146-150. , DOI 10.1046/j.1540-8167.2005.40604.x; Redfearn, D.P., Trim, G.M., Skanes, A.C., Petrellis, B., Krahn, A.D., Yee, R., Klein, G.J., Esophageal temperature monitoring during radiofrequency ablation of atrial fibrillation (2005) Journal of Cardiovascular Electrophysiology, 16 (6), pp. 589-593. , DOI 10.1111/j.1540-8167.2005.40825.x; Ruby, R.S., Wells, D., Sankaran, S., Prevalence of fever in patients undergoing left atrial ablation of atrial fibrillation guided by barium esophagraphy (2009) J Cardiovasc Electrophysiol, 20 (8), pp. 883-887. , Aug; Good, E., Oral, H., Lemola, K., Han, J., Tamirisa, K., Igic, P., Elmouchi, D., Morady, F., Movement of the esophagus during left atrial catheter ablation for atrial fibrillation (2005) Journal of the American College of Cardiology, 46 (11), pp. 2107-2110. , DOI 10.1016/j.jacc.2005.08.042, PII S0735109705021996; Cummings, J.E., Schweikert, R.A., Saliba, W.I., Burkhardt, J.D., Brachmann, J., Gunther, J., Schibgilla, V., Natale, A., Assessment of temperature, proximity, and course of the esophagus during radiofrequency ablation within the left atrium (2005) Circulation, 112 (4), pp. 459-464. , DOI 10.1161/CIRCULATIONAHA.104.509612; Ren, J.F., Marchlinski, F.E., Callans, D.J., Real-time intracardiac echocardiographic imaging of the posterior left atrial wall contiguous to anterior wall of the esophagus (2006) J Am Coll Cardiol, 48 (3), p. 594. , Aug 1 author reply 594-595; Kuwahara, T., Takahashi, A., Kobori, A., Safe and effective ablation of atrial fibrillation: Importance of esophageal temperature monitoring to avoid periesophageal nerve injury as a complication of pulmonary vein isolation (2009) J Cardiovasc Electrophysiol, 20 (1), pp. 1-6. , Jan; Leite, L.R., Santos, S.N., Maia, H., Luminal esophageal temperature monitoring with a deflectable esophageal temperature probe and intracardiac echocardiography may reduce esophageal injury during atrial fibrillation ablation procedures: Results of a pilot study (2011) Circ Arrhythm Electrophysiol, 4 (2), pp. 149-156. , Apr 1; Singh, S.M., D'Avila, A., Doshi, S.K., Esophageal injury and temperature monitoring during atrial fibrillation ablation (2008) Circ Arrhythm Electrophysiol, 1 (3), pp. 162-168. , Aug; Arruda, M.S., Armaganijan, L., Di Biase, L., Rashidi, R., Natale, A., Feasibility and safety of using an esophageal protective system to eliminate esophageal thermal injury: Implications on atrial-esophageal fistula following AF ablation (2009) J Cardiovasc Electrophysiol, 20 (11), pp. 1272-1278. , Nov; Chugh, A., Rubenstein, J., Good, E., Mechanical displacement of the esophagus in patients undergoing left atrial ablation of atrial fibrillation (2009) Heart Rhythm, 6 (3), pp. 319-322. , Mar; Tsuchiya, T., Ashikaga, K., Nakagawa, S., Hayashida, K., Kugimiya, H., Atrial fibrillation ablation with esophageal cooling with a cooled water-irrigated intraesophageal balloon: A pilot study (2007) Journal of Cardiovascular Electrophysiology, 18 (2), pp. 145-150. , DOI 10.1111/j.1540-8167.2006.00693.x; Gentlesk, P.J., Sauer, W.H., Gerstenfeld, E.P., Lin, D., Dixit, S., Pa-C, E.Z., Callans, D., Marchlinski, F.E., Reversal of left ventricular dysfunction following ablation of atrial fibrillation (2007) Journal of Cardiovascular Electrophysiology, 18 (1), pp. 9-14. , DOI 10.1111/j.1540-8167.2006.00653.x; Oral, H., Chugh, A., Good, E., Sankaran, S., Reich, S.S., Igic, P., Elmouchi, D., Morady, F., A tailored approach to catheter ablation of paroxysmal atrial fibrillation (2006) Circulation, 113 (15), pp. 1824-1831. , DOI 10.1161/CIRCULATIONAHA.105.601898, PII 0000301720060418000005; Bertaglia, E., Stabile, G., Senatore, G., Zoppo, F., Turco, P., Amellone, C., De Simone, A., Pascotto, P., Predictive value of early atrial tachyarrhythmias recurrence after circumferential anatomical pulmonary vein ablation (2005) PACE - Pacing and Clinical Electrophysiology, 28 (5), pp. 366-371. , DOI 10.1111/j.1540-8159.2005.09516.x; Vasamreddy, C.R., Lickfett, L., Jayam, V.K., Nasir, K., Bradley, D.J., Eldadah, Z., Dickfeld, T., Calkins, H., Predictors of recurrence following catheter ablation of atrial fibrillation using an irrigated-tip ablation catheter (2004) Journal of Cardiovascular Electrophysiology, 15 (6), pp. 692-697. , DOI 10.1046/j.1540-8167.2004.03538.x; Arya, A., Hindricks, G., Sommer, P., Long-term results and the predictors of outcome of catheter ablation of atrial fibrillation using steerable sheath catheter navigation after single procedure in 674 patients (2010) Europace, 12 (2), pp. 173-180. , Feb; Klemm, H.U., Ventura, R., Rostock, T., Brandstrup, B., Risius, T., Meinertz, T., Willems, S., Correlation of symptoms to ECG diagnosis following atrial fibrillation ablation (2006) Journal of Cardiovascular Electrophysiology, 17 (2), pp. 146-150. , DOI 10.1111/j.1540-8167.2005.00288.x; Vasamreddy, C.R., Dalal, D., Dong, J., Cheng, A., Spragg, D., Lamiy, S.Z., Meininger, G., Calkins, H., Symptomatic and asymptomatic atrial fibrillation in patients undergoing radiofrequency catheter ablation (2006) Journal of Cardiovascular Electrophysiology, 17 (2), pp. 134-139. , DOI 10.1111/j.1540-8167.2006.00359.x; Hindricks, G., Piorkowski, C., Tanner, H., Kobza, R., Gerds-Li, J.-H., Carbucicchio, C., Kottkamp, H., Perception of atrial fibrillation before and after radiofrequency catheter ablation: Relevance of asymptomatic arrhythmia recurrence (2005) Circulation, 112 (3), pp. 307-313. , DOI 10.1161/CIRCULATIONAHA.104.518837; Kottkamp, H., Tanner, H., Kobza, R., Schirdewahn, P., Dorszewski, A., Gerds-Li, J.-H., Carbucicchio, C., Hindricks, G., Time courses and quantitative analysis of atrial fibrillation episode number and duration after circular plus linear left atrial lesions: Trigger elimination or substrate modification: Early or delayed cure? (2004) Journal of the American College of Cardiology, 44 (4), pp. 869-877. , DOI 10.1016/j.jacc.2004.04.049, PII S0735109704010897; Oral, H., Veerareddy, S., Good, E., Hall, B., Cheung, P., Tamirisa, K., Han, J., Morady, F., Prevalence of asymptomatic recurrences of atrial fibrillation after successful radiofrequency catheter ablation (2004) Journal of Cardiovascular Electrophysiology, 15 (8), pp. 920-924. , DOI 10.1046/j.1540-8167.2004.04055.x; Senatore, G., Stabile, G., Bertaglia, E., Donnici, G., De Simone, A., Zoppo, F., Turco, P., Fazzari, M., Role of transtelephonic electrocardiographic monitoring in detecting short-term arrhythmia recurrences after radiofrequency ablation in patients with atrial fibrillation (2005) Journal of the American College of Cardiology, 45 (6), pp. 873-876. , DOI 10.1016/j.jacc.2004.11.050; Dagres, N., Kottkamp, H., Piorkowski, C., Influence of the duration of Holter monitoring on the detection of arrhythmia recurrences after catheter ablation of atrial fibrillation. Implications for patient follow-up (2010) Int J Cardiol, 139 (3), pp. 305-306. , Mar 18; Pokushalov, E., Romanov, A., Corbucci, G., Ablation of paroxysmal and persistent atrial fibrillation: 1-year follow-up through continuous subcutaneous monitoring (2011) J Cardiovasc Electrophysiol, 22 (4), pp. 369-375. , Apr; Ziegler, P.D., Koehler, J.L., Mehra, R., Comparison of continuous versus intermittent monitoring of atrial arrhythmias (2006) Heart Rhythm, 3 (12), pp. 1445-1452. , DOI 10.1016/j.hrthm.2006.07.030, PII S1547527106018418; Edgerton, J.R., Mahoney, C., Mack, M.J., Roper, K., Herbert, M.A., Long-term monitoring after surgical ablation for atrial fibrillation: How much is enough? (2011) J Thorac Cardiovasc Surg, 142 (1), pp. 162-165. , Jul; Purerfellner, H., Gillis, A.M., Holbrook, R., Hettrick, D.A., Accuracy of atrial tachyarrhythmia detection in implantable devices with arrhythmia therapies (2004) PACE - Pacing and Clinical Electrophysiology, 27 (7), pp. 983-992. , DOI 10.1111/j.1540-8159.2004.00569.x; Seidl, K., Meisel, E., VanAgt, E., Is the atrial high rate episode diagnostic feature reliable in detecting paroxysmal episodes of atrial tachyarrhythmias? (1998) Pacing Clin Electrophysiol, 21 (4 PART 1), pp. 694-700. , Apr; Eitel, C., Husser, D., Hindricks, G., Performance of an implantable automatic atrial fibrillation detection device: Impact of software adjustments and relevance of manual episode analysis (2011) Europace, 13 (4), pp. 480-485. , Apr; Hindricks, G., Pokushalov, E., Urban, L., Performance of a new leadless implantable cardiac monitor in detecting and quantifying atrial fibrillation: Results of the XPECT trial (2010) Circ Arrhythm Electrophysiol, 3 (2), pp. 141-147. , Apr 1; Schreieck, J., Ndrepepa, G., Zrenner, B., Schneider, M.A.E., Weyerbrock, S., Dong, J., Schmitt, C., Radiofrequency ablation of cardiac arrhythmias using a three-dimensional real-time position management and mapping system (2002) PACE - Pacing and Clinical Electrophysiology, 25 (12), pp. 1699-1707; Lee, S.H., Tai, C.T., Hsieh, M.H., Predictors of early and late recurrence of atrial fibrillation after catheter ablation of paroxysmal atrial fibrillation (2004) J Interv Card Electrophysiol, 10 (3), pp. 221-226. , Jun; Oral, H., Knight, B.P., Ozaydn, M., Tada, H., Chugh, A., Hassan, S., Scharf, C., Morady, F., Clinical significance of early recurrences of atrial fibrillation after pulmonary vein isolation (2002) Journal of the American College of Cardiology, 40 (1), pp. 100-104. , DOI 10.1016/S0735-1097(02)01939-3, PII S0735109702019393; Jiang, H., Lu, Z., Lei, H., Zhao, D., Yang, B., Huang, C., Predictors of early recurrence and delayed cure after segmental pulmonary vein isolation for paroxysmal atrial fibrillation without structural heart disease (2006) Journal of Interventional Cardiac Electrophysiology, 15 (3), pp. 157-163. , DOI 10.1007/s10840-006-9003-y; O'Donnell, D., Furniss, S.S., Dunuwille, A., Bourke, J.P., Delayed cure despite early recurrence after pulmonary vein isolation for atrial fibrillation (2003) American Journal of Cardiology, 91 (1), pp. 83-85. , DOI 10.1016/S0002-9149(02)03005-9, PII S0002914902030059; Joshi, S., Choi, A.D., Kamath, G.S., Prevalence, predictors, and prognosis of atrial fibrillation early after pulmonary vein isolation: Findings from 3 months of continuous automatic ECG loop recordings (2009) J Cardiovasc Electrophysiol, 20 (10), pp. 1089-1094. , Oct; Leong-Sit, P., Roux, J.F., Zado, E., Antiarrhythmics after ablation of atrial fibrillation (5A Study): Six-month follow-up study (2011) Circ Arrhythm Electrophysiol, 4 (1), pp. 11-14. , Feb; Baman, T.S., Gupta, S.K., Billakanty, S.R., Time to cardioversion of recurrent atrial arrhythmias after catheter ablation of atrial fibrillation and long-term clinical outcome (2009) J Cardiovasc Electrophysiol, 20 (12), pp. 1321-1325. , Dec; Chilukuri, K., Dukes, J., Dalal, D., Outcomes in patients requiring cardioversion following catheter ablation of atrial fibrillation (2010) J Cardiovasc Electrophysiol, 21 (1), pp. 27-32. , Jan; Choi, J.I., Pak, H.N., Park, J.S., Clinical significance of early recurrences of atrial tachycardia after atrial fibrillation ablation (2010) J Cardiovasc Electrophysiol, 21 (12), pp. 1331-1337. , Dec; Grubman, E., Pavri, B.B., Lyle, S., Reynolds, C., Denofrio, D., Kocovic, D.Z., Histopathologic effects of radiofrequency catheter ablation in previously infarcted human myocardium (1999) Journal of Cardiovascular Electrophysiology, 10 (3), pp. 336-342; Tanno, K., Kobayashi, Y., Kurano, K., Kikushima, S., Yazawa, T., Baba, T., Inoue, S., Katagiri, T., Histopathology of canine hearts subjected to catheter ablation using radiofrequency energy (1994) Japanese Circulation Journal, 58 (2), pp. 123-135; Hsieh, M.H., Chiou, C.W., Wen, Z.C., Alterations of heart rate variability after radiofrequency catheter ablation of focal atrial fibrillation originating from pulmonary veins (1999) Circulation, 100 (22), pp. 2237-2243. , Nov 30; Fenelon, G., Brugada, P., Delayed effects of radiofrequency energy: Mechanisms and clinical implications (1996) PACE - Pacing and Clinical Electrophysiology, 19 (4 I), pp. 484-489. , DOI 10.1111/j.1540-8159.1996.tb06520.x; Klein, L.S., Shih, H.T., Hackett, F.K., Zipes, D.P., Miles, W.M., Radiofrequency catheter ablation of ventricular tachycardia in patients without structural heart disease (1992) Circulation, 85 (5), pp. 1666-1674. , May; Langberg, J.J., Borganelli, S.M., Kalbfleisch, S.J., Strickberger, S.A., Calkins, H., Morady, F., Delayed effects of radiofrequency energy on accessory atrioventricular connections (1993) PACE - Pacing and Clinical Electrophysiology, 16 (5 I), pp. 1001-1005; Brooks, A.G., Stiles, M.K., Laborderie, J., Outcomes of long-standing persistent atrial fibrillation ablation: A systematic review (2010) Heart Rhythm, 7 (6), pp. 835-846. , Jun; Chugh, A., Oral, H., Lemola, K., Hall, B., Cheung, P., Good, E., Tamirisa, K., Morady, F., Prevalence, mechanisms, and clinical significance of macroreentrant atrial tachycardia during and following left atrial ablation for atrial fibrillation (2005) Heart Rhythm, 2 (5), pp. 464-471. , DOI 10.1016/j.hrthm.2005.01.027, PII S1547527105001980; Gerstenfeld, E.P., Marchlinski, F.E., Mapping and ablation of left atrial tachycardias occurring after atrial fibrillation ablation (2007) Heart Rhythm, 4 (3 SUPPL.), pp. S65-S72. , Mar; Villacastin, J., Perez-Castellano, N., Moreno, J., Gonzalez, R., Left atrial flutter after radiofrequency catheter ablation of focal atrial fibrillation (2003) Journal of Cardiovascular Electrophysiology, 14 (4), pp. 417-421. , DOI 10.1046/j.1540-8167.2003.02418.x; Anousheh, R., Sawhney, N.S., Panutich, M., Tate, C., Chen, W.C., Feld, G.K., Effect of mitral isthmus block on development of atrial tachycardia following ablation for atrial fibrillation (2010) Pacing Clin Electrophysiol, 33 (4), pp. 460-468. , Apr; Chugh, A., Oral, H., Good, E., Han, J., Tamirisa, K., Lemola, K., Elmouchi, D., Morady, F., Catheter ablation of atypical atrial flutter and atrial tachycardia within the coronary sinus after left atrial ablation for atrial fibrillation (2005) Journal of the American College of Cardiology, 46 (1), pp. 83-91. , DOI 10.1016/j.jacc.2005.03.053, PII S0735109705008648; Deisenhofer, I., Estner, H., Zrenner, B., Schreieck, J., Weyerbrock, S., Hessling, G., Scharf, K., Schmitt, C., Left atrial tachycardia after circumferential pulmonary vein ablation for atrial fibrillation: Incidence, electrophysiological characteristics, and results of radiofrequency ablation (2006) Europace, 8 (8), pp. 573-582. , DOI 10.1093/europace/eul077; Gerstenfeld, E.P., Callans, D.J., Sauer, W., Jacobson, J., Marchlinski, F.E., Reentrant and nonreentrant focal left atrial tachycardias occur after pulmonary vein isolation (2005) Heart Rhythm, 2 (11), pp. 1195-1202. , DOI 10.1016/j.hrthm.2005.08.020, PII S1547527105020126; Lim, T.W., Koay, C.H., McCall, R., See, V.A., Ross, D.L., Thomas, S.P., Atrial arrhythmias after single-ring isolation of the posterior left atrium and pulmonary veins for atrial fibrillation: Mechanisms and management (2008) Circ Arrhythm Electrophysiol, 1 (2), pp. 120-126. , Jun 1; Oral, H., Knight, B.P., Morady, F., Left atrial flutter after segmental ostial radiofrequency catheter ablation for pulmonary vein isolation (2003) PACE - Pacing and Clinical Electrophysiology, 26 (6), pp. 1417-1419. , DOI 10.1046/j.1460-9592.2003.t01-1-00202.x; Thomas, S.P., Wallace, E.M., Ross, D.L., The effect of a residual isthmus of surviving tissue on conduction after linear ablation in atrial myocardium (2000) Journal of Interventional Cardiac Electrophysiology, 4 (1), pp. 273-281. , DOI 10.1023/A:1009838201448; Jais, P., Sanders, P., Hsu, L.-F., Hocini, M., Sacher, F., Takahashi, Y., Rotter, M., Haissaguerre, M., Flutter localized to the anterior left atrium after catheter ablation of atrial fibrillation (2006) Journal of Cardiovascular Electrophysiology, 17 (3), pp. 279-285. , DOI 10.1111/j.1540-8167.2005.00292.x; Patel, A.M., D'Avila, A., Neuzil, P., Atrial tachycardia after ablation of persistent atrial fibrillation: Identification of the critical isthmus with a combination of multielectrode activation mapping and targeted entrainment mapping (2008) Circ Arrhythm Electrophysiol, 1 (1), pp. 14-22. , Apr; Marchlinski, F.E., Callans, D., Dixit, S., Gerstenfeld, E.P., Rho, R., Ren, J.-F., Zado, E., Efficacy and safety of targeted focal ablation versus PV isolation assisted by magnetic electroanatomic mapping (2003) Journal of Cardiovascular Electrophysiology, 14 (4), pp. 358-365. , DOI 10.1046/j.1540-8167.2003.02468.x; Roux, J.F., Zado, E., Callans, D.J., Antiarrhythmics after Ablation of Atrial Fibrillation (5A Study) (2009) Circulation, 120 (12), pp. 1036-1040. , Sep 22; Tayebjee, M.H., Creta, A., Moder, S., Impact of angiotensin-converting enzyme-inhibitors and angiotensin receptor blockers on long-term outcome of catheter ablation for atrial fibrillation (2010) Europace, 12 (11), pp. 1537-1542. , Nov; Bauer, A.C., Imig, C.J., Blood flow through human forearm following different types, intensities, and durations of exercise (1959) Am J Phys Med, 38 (2), pp. 48-52. , Apr; Gerstenfeld, E.P., Callans, D.J., Dixit, S., Zado, E., Marchlinski, F.E., Incidence and location of focal atrial fibrillation triggers in patients undergoing repeat pulmonary vein isolation: Implications for ablation strategies (2003) Journal of Cardiovascular Electrophysiology, 14 (7), pp. 685-690; Nanthakumar, K., Plumb, V.J., Epstein, A.E., Veenhuyzen, G.D., Link, D., Kay, G.N., Resumption of Electrical Conduction in Previously Isolated Pulmonary Veins: Rationale for a Different Strategy? (2004) Circulation, 109 (10), pp. 1226-1229. , DOI 10.1161/01.CIR.0000121423.78120.49; Bauer, A., Deisenhofer, I., Schneider, R., Zrenner, B., Barthel, P., Karch, M., Wagenpfeil, S., Schmidt, G., Effects of circumferential or segmental pulmonary vein ablation for paroxysmal atrial fibrillation on cardiac autonomic function (2006) Heart Rhythm, 3 (12), pp. 1428-1435. , DOI 10.1016/j.hrthm.2006.08.025, PII S1547527106019357; Scanavacca, M., Pisani, C.F., Hachul, D., Lara, S., Hardy, C., Darrieux, F., Trombetta, I., Sosa, E., Selective atrial vagal denervation guided by evoked vagal reflex to treat patients with paroxysmal atrial fibrillation (2006) Circulation, 114 (9), pp. 876-885. , DOI 10.1161/CIRCULATIONAHA.106.633560, PII 0000301720060829000005; Bhargava, M., Di Biase, L., Mohanty, P., Impact of type of atrial fibrillation and repeat catheter ablation on long-term freedom from atrial fibrillation: Results from a multicenter study (2009) Heart Rhythm, 6 (10), pp. 1403-1412. , Oct; Gaita, F., Caponi, D., Scaglione, M., Long-term clinical results of 2 different ablation strategies in patients with paroxysmal and persistent atrial fibrillation (2008) Circ Arrhythm Electrophysiol, 1 (4), pp. 269-275. , Oct; Katritsis, D., Wood, M.A., Giazitzoglou, E., Shepard, R.K., Kourlaba, G., Ellenbogen, K.A., Long-term follow-up after radiofrequency catheter ablation for atrial fibrillation (2008) Europace, 10 (4), pp. 419-424. , DOI 10.1093/europace/eun018; Medi, C., Sparks, P.B., Morton, J.B., Pulmonary vein antral isolation for paroxysmal atrial fibrillation: Results from long-term follow-up (2011) J Cardiovasc Electrophysiol, 22 (2), pp. 137-141. , Feb; Shah, A.N., Mittal, S., Sichrovsky, T.C., Long-term outcome following successful pulmonary vein isolation: Pattern and prediction of very late recurrence (2008) J Cardiovasc Electrophysiol, 19 (7), pp. 661-667. , Jul; Tzou, W.S., Marchlinski, F.E., Zado, E.S., Long-term outcome after successful catheter ablation of atrial fibrillation (2010) Circ Arrhythm Electrophysiol, 3 (3), pp. 237-242. , Jun 1; Connolly, S.J., Ezekowitz, M.D., Yusuf, S., Dabigatran versus warfarin in patients with atrial fibrillation (2009) N Engl J Med, 361 (12), pp. 1139-1151. , Sep 17; Daoud, E.G., Glotzer, T.V., Wyse, D.G., Temporal relationship of atrial tachyarrhythmias, cerebrovascular events, and systemic emboli based on stored device data: A subgroup analysis of TRENDS (2011) Heart Rhythm, 8 (9), pp. 1416-1423. , Sep; Wokhlu, A., Monahan, K.H., Hodge, D.O., Long-term quality of life after ablation of atrial fibrillation the impact of recurrence, symptom relief, and placebo effect (2010) J Am Coll Cardiol, 55 (21), pp. 2308-2316. , May 25; Chao, T.F., Lin, Y.J., Tsao, H.M., CHADS(2) and CHA(2)DS(2)-VASc scores in the prediction of clinical outcomes in patients with atrial fibrillation after catheter ablation (2011) J Am Coll Cardiol, 58 (23), pp. 2380-2385. , Nov 29; Krittayaphong, R., Raungrattanaamporn, O., Bhuripanyo, K., A randomized clinical trial of the efficacy of radiofrequency catheter ablation and amiodarone in the treatment of symptomatic atrial fibrillation (2003) J Med Assoc Thai, 86 (SUPPL. 1), pp. S8-S16. , May; Noheria, A., Kumar, A., Wylie Jr., J.V., Josephson, M.E., Catheter ablation vs antiarrhythmic drug therapy for atrial fibrillation: A systematic review (2008) Archives of Internal Medicine, 168 (6), pp. 581-586. , http://archinte.ama-assn.org/cgi/reprint/168/6/581, DOI 10.1001/archinte.168.6.581; Stabile, G., Bertaglia, E., Senatore, G., De Simone, A., Zoppo, F., Donnici, G., Turco, P., Vitale, D.F., Catheter ablation treatment in patients with drug-refractory atrial fibrillation: A prospective, multi-centre, randomized, controlled study (Catheter Ablation for the Cure of Atrial Fibrillation Study) (2006) European Heart Journal, 27 (2), pp. 216-221. , DOI 10.1093/eurheartj/ehi583; Wilber, D.J., Pappone, C., Neuzil, P., Comparison of antiarrhythmic drug therapy and radiofrequency catheter ablation in patients with paroxysmal atrial fibrillation: A randomized controlled trial (2010) Jama, 303 (4), pp. 333-340. , Jan 27; Cappato, R., Calkins, H., Chen, S.A., Updated worldwide survey on the methods, efficacy, and safety of catheter ablation for human atrial fibrillation (2010) Circ Arrhythm Electrophysiol, 3 (1), pp. 32-38. , Feb 1; Reynolds, M.R., Walczak, J., White, S.A., Cohen, D.J., Wilber, D.J., Improvements in symptoms and quality of life in patients with paroxysmal atrial fibrillation treated with radiofrequency catheter ablation versus antiarrhythmic drugs (2010) Circ Cardiovasc Qual Outcomes, 3 (6), pp. 615-623. , Nov 1; Bonanno, C., Paccanaro, M., La Vecchia, L., Ometto, R., Fontanelli, A., Efficacy and safety of catheter ablation versus antiarrhythmic drugs for atrial fibrillation: A meta-analysis of randomized trials (2010) J Cardiovasc Med (Hagerstown), 11 (6), pp. 408-418. , Jun; Nair, G.M., Nery, P.B., Diwakaramenon, S., Healey, J.S., Connolly, S.J., Morillo, C.A., A systematic review of randomized trials comparing radiofrequency ablation with antiarrhythmic medications in patients with atrial fibrillation (2009) J Cardiovasc Electrophysiol, 20 (2), pp. 138-144. , Feb; Parkash, R., Tang, A.S., Sapp, J.L., Wells, G., Approach to the catheter ablation technique of paroxysmal and persistent atrial fibrillation: A meta-analysis of the randomized controlled trials (2011) J Cardiovasc Electrophysiol, , Feb 18; Piccini, J.P., Lopes, R.D., Kong, M.H., Hasselblad, V., Jackson, K., Al-Khatib, S.M., Pulmonary vein isolation for the maintenance of sinus rhythm in patients with atrial fibrillation: A meta-analysis of randomized, controlled trials (2009) Circ Arrhythm Electrophysiol, 2 (6), pp. 626-633. , Dec; Calkins, H., Reynolds, M.R., Spector, P., Treatment of atrial fibrillation with antiarrhythmic drugs or radiofrequency ablation: Two systematic literature reviews and meta-analyses (2009) Circ Arrhythm Electrophysiol, 2 (4), pp. 349-361. , Aug; Hayward, R.M., Upadhyay, G.A., Mela, T., Pulmonary vein isolation with complex fractionated atrial electrogram ablation for paroxysmal and nonparoxysmal atrial fibrillation: A meta-analysis (2011) Heart Rhythm, 8 (7), pp. 994-1000. , Jul; Kong, M.H., Piccini, J.P., Bahnson, T.D., Efficacy of adjunctive ablation of complex fractionated atrial electrograms and pulmonary vein isolation for the treatment of atrial fibrillation: A meta-analysis of randomized controlled trials (2011) Europace, 13 (2), pp. 193-204. , Feb; Li, W.J., Bai, Y.Y., Zhang, H.Y., Additional ablation of complex fractionated atrial electrograms after pulmonary vein isolation in patients with atrial fibrillation: A meta-analysis (2011) Circ Arrhythm Electrophysiol, 4 (2), pp. 143-148. , Apr 1; Hoyt, H., Nazarian, S., Alhumaid, F., Demographic profile of patients undergoing catheter ablation of atrial fibrillation (2011) J Cardiovasc Electrophysiol, 22 (9), pp. 994-998. , Sep; Tilz, R.R., Chun, K.R., Schmidt, B., Catheter ablation of long-standing persistent atrial fibrillation: A lesson from circumferential pulmonary vein isolation (2010) J Cardiovasc Electrophysiol, 21 (10), pp. 1085-1093. , Oct; Corrado, A., Patel, D., Riedlbauchova, L., Fahmy, T.S., Themistoclakis, S., Bonso, A., Rossillo, A., Natale, A., Efficacy, safety, and outcome of atrial fibrillation ablation in septuagenarians (2008) Journal of Cardiovascular Electrophysiology, 19 (8), pp. 807-811. , DOI 10.1111/j.1540-8167.2008.01124.x; Hsu, L.F., Jais, P., Sanders, P., Catheter ablation for atrial fibrillation in congestive heart failure (2004) N Engl J Med, 351 (23), pp. 2373-2383. , Dec 2; Khan, M.N., Jais, P., Cummings, J., Pulmonary-vein isolation for atrial fibrillation in patients with heart failure (2008) N Engl J Med, 359 (17), pp. 1778-1785. , Oct 23; De Potter, T., Berruezo, A., Mont, L., Left ventricular systolic dysfunction by itself does not influence outcome of atrial fibrillation ablation (2010) Europace, 12 (1), pp. 24-29. , Jan; Wilton, S.B., Fundytus, A., Ghali, W.A., Meta-analysis of the effectiveness and safety of catheter ablation of atrial fibrillation in patients with versus without left ventricular systolic dysfunction (2010) Am J Cardiol, 106 (9), pp. 1284-1291. , Nov 1; Kojodjojo, P., O'Neill, M.D., Lim, P.B., Pulmonary venous isolation by antral ablation with a large cryoballoon for treatment of paroxysmal and persistent atrial fibrillation: Medium-term outcomes and non-randomised comparison with pulmonary venous isolation by radiofrequency ablation (2010) Heart, 96 (17), pp. 1379-1384. , Sep; Kuhne, M., Suter, Y., Altmann, D., Cryoballoon versus radiofrequency catheter ablation of paroxysmal atrial fibrillation: Biomarkers of myocardial injury, recurrence rates, and pulmonary vein reconnection patterns (2010) Heart Rhythm, 7 (12), pp. 1770-1776. , Dec; Linhart, M., Bellmann, B., Mittmann-Braun, E., Comparison of cryoballoon and radiofrequency ablation of pulmonary veins in 40 patients with paroxysmal atrial fibrillation: A case-control study (2009) J Cardiovasc Electrophysiol, 20 (12), pp. 1343-1348. , Dec; Dorian, P., Jung, W., Newman, D., The impairment of health-related quality of life in patients with intermittent atrial fibrillation: Implications for the assessment of investigational therapy (2000) J Am Coll Cardiol, 36 (4), pp. 1303-1309. , Oct; Reynolds, M.R., Ellis, E., Zimetbaum, P., Quality of life in atrial fibrillation: Measurement tools and impact of interventions (2008) J Cardiovasc Electrophysiol, 19 (7), pp. 762-768. , Jul; Ware, J., Snow, K., Kosinski, M., Gandek, B., (1993) SF-36 Health Survey: Manual, Interpretation Guide, , Boston: The Health Institute, New England Medical Center; Bubien, R.S., Knotts-Dolson, S.M., Plumb, V.J., Kay, G.N., Effect of radiofrequency catheter ablation on health-related quality of life and activities of daily living in patients with recurrent arrhythmias (1996) Circulation, 94 (7), pp. 1585-1591; Thrall, G., Lane, D., Carroll, D., Lip, G.Y., Quality of life in patients with atrial fibrillation: A systematic review (2006) Am J Med, 119 (5), pp. 448e441-448e1419. , May; Berkowitsch, A., Neumann, T., Kurzidim, K., Reiner, C., Kuniss, M., Siemon, G., Sperzel, J., Pitschner, H.F., Comparison of generic health survey SF-36 and arrhythmia related symptom severity check list in relation to post-therapy AF recurrence (2003) Europace, 5 (4), pp. 351-355. , DOI 10.1016/S1099-5129(03)00089-8; Kirchhof, P., Auricchio, A., Bax, J., Outcome parameters for trials in atrial fibrillation: Executive summary (2007) Eur Heart J, 28 (22), pp. 2803-2817. , Nov; Spertus, J., Dorian, P., Bubien, R., Development and validation of the Atrial Fibrillation Effect on QualiTy-of-Life (AFEQT) Questionnaire in patients with atrial fibrillation (2011) Circ Arrhythm Electrophysiol, 4 (1), pp. 15-25. , Feb; Fichtner, S., Deisenhofer, I., Kindsmuller, S., Prospective assessment of short and long-term quality of life after ablation for atrial fibrillation (2012) J Cardiovasc Electrophysiol, 23 (2), pp. 121-127. , Feb; Ausma, J., Litjens, N., Lenders, M.-H., Duimel, H., Mast, F., Wouters, L., Ramaekers, F., Borgers, M., Time course of atrial fibrillation-induced cellular structural remodeling in atria of the goat (2001) Journal of Molecular and Cellular Cardiology, 33 (12), pp. 2083-2094. , DOI 10.1006/jmcc.2001.1472; Wijffels, M.C.E.F., Kirchhof, C.J.H.J., Dorland, R., Power, J., Allessie, M.A., Electrical remodeling due to atrial fibrillation in chronically instrumented conscious goats: Roles of neurohumoral changes, ischemia, atrial stretch, and high rate of electrical activation (1997) Circulation, 96 (10), pp. 3710-3720; Jayam, V.K., Dong, J., Vasamreddy, C.R., Lickfett, L., Kato, R., Dickfeld, T., Eldadah, Z., Calkins, H., Atrial volume reduction following catheter ablation of atrial fibrillation and relation to reduction in pulmonary vein size: An evaluation using magnetic resonance angiography (2005) Journal of Interventional Cardiac Electrophysiology, 13 (2), pp. 107-114. , DOI 10.1007/s10840-005-0215-3; Scharf, C., Sneider, M., Case, I., Chugh, A., Lai, S.W.K., Pelosi Jr., F., Knight, B.P., Oral, H., Anatomy of the pulmonary veins in patients with atrial fibrillation and effects of segmental ostial ablation analyzed by computed tomography (2003) Journal of Cardiovascular Electrophysiology, 14 (2), pp. 150-155; Tsao, H.M., Wu, M.H., Huang, B.H., Morphologic remodeling of pulmonary veins and left atrium after catheter ablation of atrial fibrillation: Insight from long-term follow-up of three-dimensional magnetic resonance imaging (2005) J Cardiovasc Electrophysiol, 16 (1), pp. 7-12. , Jan; Lemola, K., Desjardins, B., Sneider, M., Case, I., Chugh, A., Good, E., Han, J., Oral, H., Effect of left atrial circum ferential ablation for atrial fibrillation on left atrial transport function (2005) Heart Rhythm, 2 (9), pp. 923-928. , DOI 10.1016/j.hrthm.2005.06.026, PII S1547527105017741; Verma, A., Kilicaslan, F., Adams, J.R., Extensive ablation during pulmonary vein antrum isolation has no adverse impact on left atrial function: An echocardiography and cine computed tomography analysis (2006) J Cardiovasc Electrophysiol, 17 (7), pp. 741-746. , Jul; Gibson, D.N., Di Biase, L., Mohanty, P., Stiff left atrial syndrome after catheter ablation for atrial fibrillation: Clinical characterization, prevalence, and predictors (2011) Heart Rhythm, 8 (9), pp. 1364-1371. , Sep; Balk, E.M., Garlitski, A.C., Alsheikh-Ali, A.A., Terasawa, T., Chung, M., Ip, S., Predictors of atrial fibrillation recurrence after radiofrequency catheter ablation: A systematic review (2010) J Cardiovasc Electrophysiol, 21 (11), pp. 1208-1216. , Nov; Berruezo, A., Tamborero, D., Mont, L., Benito, B., Tolosana, J.M., Sitges, M., Vidal, B., Brugada, J., Pre-procedural predictors of atrial fibrillation recurrence after circumferential pulmonary vein ablation (2007) European Heart Journal, 28 (7), pp. 836-841. , DOI 10.1093/eurheartj/ehm027; McCready, J.W., Smedley, T., Lambiase, P.D., Predictors of recurrence following radiofrequency ablation for persistent atrial fibrillation (2011) Europace, 13 (3), pp. 355-361. , Mar; Goldberg, A., Menen, M., Mickelsen, S., MacIndoe, C., Binder, M., Nawman, R., West, G., Kusumoto, F.M., Atrial fibrillation ablation leads to long-term improvement of quality of life and reduced utilization of healthcare resources (2003) Journal of Interventional Cardiac Electrophysiology, 8 (1), pp. 59-64. , DOI 10.1023/A:1022348216072; Weerasooriya, R., Jais, P., Le, H.J.-Y., Scavee, C., Choi, K.-J., Macle, L., Raybaud, F., Haissaguerre, M., Cost analysis of catheter ablation for paroxysmal atrial fibrillation (2003) PACE - Pacing and Clinical Electrophysiology, 26 (1 II), pp. 292-294; Chan, P.S., Vijan, S., Morady, F., Oral, H., Cost-Effectiveness of Radiofrequency Catheter Ablation for Atrial Fibrillation (2006) Journal of the American College of Cardiology, 47 (12), pp. 2513-2520. , DOI 10.1016/j.jacc.2006.01.070, PII S0735109706007509; Cappato, R., Calkins, H., Chen, S.A., Prevalence and causes of fatal outcome in catheter ablation of atrial fibrillation (2009) J Am Coll Cardiol, 53 (19), pp. 1798-1803. , May 12; Bunch, T.J., Asirvatham, S.J., Friedman, P.A., Outcomes after cardiac perforation during radiofrequency ablation of the atrium (2005) J Cardiovasc Electrophysiol, 16 (11), pp. 1172-1179. , Nov; Eick, O.J., Gerritse, B., Schumacher, B., Popping phenomena in temperature-controlled radiofrequency ablation: When and why do they occur? (2000) PACE - Pacing and Clinical Electrophysiology, 23 (2), pp. 253-258; Fisher, J.D., Kim, S.G., Ferrick, K.J., Gross, J.N., Goldberger, M.H., Nanna, M., Internal transcardiac pericardiocentesis for acute tamponade (2000) Am J Cardiol, 86 (12), pp. 1388-1389+A1386. , Dec 15; Hsu, L.-F., Jais, P., Hocini, M., Sanders, P., Scavee, C., Sacher, F., Takahashi, Y., Haissaguerre, M., Incidence and prevention of cardiac tamponade complicating ablation for atrial fibrillation (2005) PACE - Pacing and Clinical Electrophysiology, 28 (SUPPL. 1), pp. S106-S109. , DOI 10.1111/j.1540-8159.2005.00062.x; Hsu, L.-F., Scavee, C., Jais, P., Hocini, M., Haissaguerre, M., Transcardiac pericardiocentesis: An emergency life-saving technique for cardiac tamponade (2003) Journal of Cardiovascular Electrophysiology, 14 (9), pp. 1001-1003. , DOI 10.1046/j.1540-8167.2003.03153.x; Capatto, R., Calkins, H., Chen, S.-A., Delayed cardiac tamponade after radiofrequency catheter ablation of atrial fibrillation: A worldwide report (2011) J Am Coll Card, 58 (25), pp. 2696-2697. , Dec 13; Tsang, T.S.M., Enriquez-Sarano, M., Freeman, W.K., Barnes, M.E., Sinak, L.J., Gersh, B.J., Bailey, K.R., Seward, J.B., Consecutive 1127 therapeutic echocardiographically guided pericardiocenteses: Clinical profile, practice patterns, and outcomes spanning 21 years (2002) Mayo Clinic Proceedings, 77 (5), pp. 429-436; O'Neill, M.D., Jais, P., Derval, N., Hocini, M., Haissaguerre, M., Two techniques to avoid surgery for cardiac tamponade occurring during catheter ablation of atrial fibrillation (2008) Journal of Cardiovascular Electrophysiology, 19 (3), pp. 323-325. , DOI 10.1111/j.1540-8167.2007.00973.x; Latchamsetty, R., Gautam, S., Bhakta, D., Management and outcomes of cardiac tamponade during atrial fibrillation ablation in the presence of therapeutic anticoagulation with warfarin (2011) Heart Rhythm, 8 (6), pp. 805-808. , Jun; Ernst, S., Ouyang, F., Goya, M., Lober, F., Schneider, C., Hoffmann-Riem, M., Schwarz, S., Kuck, K.-H., Total pulmonary vein occlusion as a consequence of catheter ablation for atrial fibrillation mimicking primary lung disease (2003) Journal of Cardiovascular Electrophysiology, 14 (4), pp. 366-370. , DOI 10.1046/j.1540-8167.2003.02334.x; Katz, E.S., Tsiamtsiouris, T., Applebaum, R.M., Schwartzbard, A., Tunick, P.A., Kronzon, I., Surgical left atrial appendage ligation is frequently incomplete: A transesophageal echocardiographic study (2000) Journal of the American College of Cardiology, 36 (2), pp. 468-471. , DOI 10.1016/S0735-1097(00)00765-8, PII S0735109700007658; Packer, D.L., Keelan, P., Munger, T.M., Breen, J.F., Asirvatham, S., Peterson, L.A., Monahan, K.H., Holmes Jr., D.R., Clinical presentation, investigation, and management of pulmonary vein stenosis complicating ablation for atrial fibrillation (2005) Circulation, 111 (5), pp. 546-554. , DOI 10.1161/01.CIR.0000154541.58478.36; Taylor, G.W., Kay, G.N., Zheng, X., Bishop, S., Ideker, R.E., Pathological effects of extensive radiofrequency energy applications in the pulmonary veins in dogs (2000) Circulation, 101 (14), pp. 1736-1742; Thomas, D., Katus, H.A., Voss, F., Asymptomatic pulmonary vein stenosis after cryoballoon catheter ablation of paroxysmal atrial fibrillation (2011) J Electrocardiol, 44 (4), pp. 473-476. , Jul-Aug; Dill, T., Neumann, T., Ekinci, O., Breidenbach, C., John, A., Erdogan, A., Bachmann, G., Pitschner, H.-F., Pulmonary vein diameter reduction after radiofrequency catheter ablation for paroxysmal atrial fibrillation evaluated by contrast-enhanced three-dimensional magnetic resonance imaging (2003) Circulation, 107 (6), pp. 845-850. , DOI 10.1161/01.CIR.0000048146.81336.1D; Jin, Y., Ross, D.L., Thomas, S.P., Pulmonary vein stenosis and remodeling after electrical isolation for treatment of atrial fibrillation: Short- and medium-term follow-up (2004) PACE - Pacing and Clinical Electrophysiology, 27 (10), pp. 1362-1370. , DOI 10.1111/j.1540-8159.2004.00640.x; Baranowski, B., Saliba, W., Our approach to management of patients with pulmonary vein stenosis following AF ablation (2011) J Cardiovasc Electrophysiol, 22 (3), pp. 364-367. , Mar; Neumann, T., Kuniss, M., Conradi, G., Pulmonary vein stenting for the treatment of acquired severe pulmonary vein stenosis after pulmonary vein isolation: Clinical implications after long-term follow-up of 4 years (2009) J Cardiovasc Electrophysiol, 20 (3), pp. 251-257. , Mar; Ho, S.Y., Cabrera, J.A., Sanchez-Quintana, D., Vagaries of the vagus nerve: Relevance to ablationists (2006) Journal of Cardiovascular Electrophysiology, 17 (3), pp. 330-331. , DOI 10.1111/j.1540-8167.2006.00364.x; Tsao, H.-M., Wu, M.-H., Higa, S., Lee, K.-T., Tai, C.-T., Hsu, N.-W., Chang, C.-Y., Chen, S.-A., Anatomic relationship of the esophagus and left atrium: Implication for catheter ablation of atrial fibrillation (2005) Chest, 128 (4), pp. 2581-2587. , DOI 10.1378/chest.128.4.2581; Ueno, T., Uemura, K., Harris, M.B., Pappas, T.N., Takahashi, T., Role of vagus nerve in postprandial antropyloric coordination in conscious dogs (2005) American Journal of Physiology - Gastrointestinal and Liver Physiology, 288 (3), pp. G487-G495. , DOI 10.1152/ajpgi.00195.2004; Cury, R.C., Abbara, S., Schmidt, S., Malchano, Z.J., Neuzil, P., Weichet, J., Ferencik, M., Reddy, V.Y., Relationship of the esophagus and aorta to the left atrium and pulmonary veins: Implications for catheter ablation of atrial fibrillation (2005) Heart Rhythm, 2 (12), pp. 1317-1323. , DOI 10.1016/j.hrthm.2005.09.012, PII S1547527105021041; Borchert, B., Lawrenz, T., Hansky, B., Stellbrink, C., Lethal atrioesophageal fistula after pulmonary vein isolation using high-intensity focused ultrasound (HIFU) (2008) Heart Rhythm, 5 (1), pp. 145-148. , Jan; Doll, N., Borger, M.A., Fabricius, A., Stephan, S., Gummert, J., Mohr, F.W., Hauss, J., Hindricks, G., Esophageal perforation during left atrial radiofrequency ablation: Is the risk too high? (2003) Journal of Thoracic and Cardiovascular Surgery, 125 (4), pp. 836-842. , DOI 10.1067/mtc.2003.165; Gilcrease, G.W., Stein, J.B., A delayed case of fatal atrioesophageal fistula following radiofrequency ablation for atrial fibrillation (2010) J Cardiovasc Electrophysiol, 21 (6), pp. 708-711. , Jun 1; Gillinov, A.M., Pettersson, G., Rice, T.W., Esophageal injury during radiofrequency ablation for atrial fibrillation (2001) J Thorac Cardiovasc Surg, 122 (6), pp. 1239-1240. , Dec; Mohr, F.W., Fabricius, A.M., Falk, V., Curative treatment of atrial fibrillation with intraoperative radiofrequency ablation: Short-term and midterm results (2002) J Thorac Cardiovasc Surg, 123 (5), pp. 919-927. , May; Scanavacca, M.I., D'Avila, A., Parga, J., Sosa, E., Left atrial-esophageal fistula following radiofrequency catheter ablation of atrial fibrillation (2004) Journal of Cardiovascular Electrophysiology, 15 (8), pp. 960-962. , DOI 10.1046/j.1540-8167.2004.04083.x; Sonmez, B., Demirsoy, E., Yagan, N., Unal, M., Arbatli, H., Sener, D., Baran, T., Ilkova, F., A fatal complication due to radiofrequency ablation for atrial fibrillation: Atrio-esophageal fistula (2003) Annals of Thoracic Surgery, 76 (1), pp. 281-283. , DOI 10.1016/S0003-4975(03)00006-7, PII S0003497503000067; Ghia, K.K., Chugh, A., Good, E., A nationwide survey on the prevalence of atrioesophageal fistula after left atrial radiofrequency catheter ablation (2009) J Interv Card Electrophysiol, 24 (1), pp. 33-36. , Jan; D'Avila, A., Dukkipati, S., Esophageal damage during catheter ablation of atrial fibrillation: Is cryo safer than RF? (2009) Pacing Clin Electrophysiol, 32 (6), pp. 709-710. , Jun; Herweg, B., Ali, R., Khan, N., Ilercil, A., Barold, S.S., Esophageal contour changes during cryoablation of atrial fibrillation (2009) Pacing Clin Electrophysiol, 32 (6), pp. 711-716. , Jun; Marrouche, N.F., Guenther, J., Segerson, N.M., Daccarett, M., Rittger, H., Marschang, H., Schibgilla, V., Brachmann, J., Randomized comparison between open irrigation technology and intracardiac-echo-guided energy delivery for pulmonary vein antrum isolation: Procedural parameters, outcomes, and the effect on esophageal injury (2007) Journal of Cardiovascular Electrophysiology, 18 (6), pp. 583-588. , DOI 10.1111/j.1540-8167.2007.00879.x; Nakagawa, H., Seres, K.A., Jackman, W.M., Limitations of esophageal temperature-monitoring to prevent esophageal injury during atrial fibrillation ablation (2008) Circ Arrhythm Electrophysiol, 1 (3), pp. 150-152. , Aug; Schmidt, B., Metzner, A., Chun, K.R., Feasibility of circumferential pulmonary vein isolation using a novel endoscopic ablation system (2010) Circ Arrhythm Electrophysiol, 3 (5), pp. 481-488. , Oct 1; Tilz, R.R., Chun, K.R., Metzner, A., Unexpected high incidence of esophageal injury following pulmonary vein isolation using robotic navigation (2010) J Cardiovasc Electrophysiol, 21 (8), pp. 853-858. , Aug 1; Yokoyama, K., Nakagawa, H., Seres, K.A., Canine model of esophageal injury and atrial-esophageal fistula after applications of forward-firing high-intensity focused ultrasound and side-firing unfocused ultrasound in the left atrium and inside the pulmonary vein (2009) Circ Arrhythm Electrophysiol, 2 (1), pp. 41-49. , Feb; Zellerhoff, S., Ullerich, H., Lenze, F., Damage to the esophagus after atrial fibrillation ablation: Just the tip of the iceberg? High prevalence of mediastinal changes diagnosed by endosonography (2010) Circ Arrhythm Electrophysiol, 3 (2), pp. 155-159. , Apr 1; Di Biase, L., Saenz, L.C., Burkhardt, D.J., Esophageal capsule endoscopy after radiofrequency catheter ablation for atrial fibrillation: Documented higher risk of luminal esophageal damage with general anesthesia as compared with conscious sedation (2009) Circ Arrhythm Electrophysiol, 2 (2), pp. 108-112. , Apr; Martinek, M., Meyer, C., Hassanein, S., Identification of a high-risk population for esophageal injury during radiofrequency catheter ablation of atrial fibrillation: Procedural and anatomical considerations (2010) Heart Rhythm, 7 (9), pp. 1224-1230. , Sep; Bunch, T.J., Nelson, J., Foley, T., Temporary esophageal stenting allows healing of esophageal perforations following atrial fibrillation ablation procedures (2006) J Cardiovasc Electrophysiol, 17 (4), pp. 435-439. , Apr; Ajaj, W., Goehde, S.C., Papanikolaou, N., Holtmann, G., Ruehm, S.G., Debatin, J.F., Lauenstein, T.C., Real time high resolution magnetic resonance imaging for the assessment of gastric motility disorders (2004) Gut, 53 (9), pp. 1256-1261. , DOI 10.1136/gut.2003.038588; Bunch, T.J., Ellenbogen, K.A., Packer, D.L., Asirvatham, S.J., Vagus nerve injury after posterior atrial radiofrequency ablation (2008) Heart Rhythm, 5 (9), pp. 1327-1330. , Sep; Pisani, C.F., Hachul, D., Sosa, E., Scanavacca, M., Gastric hypomotility following epicardial vagal denervation ablation to treat atrial fibrillation (2008) Journal of Cardiovascular Electrophysiology, 19 (2), pp. 211-213. , DOI 10.1111/j.1540-8167.2007.00937.x; Schwartz, T.W., Rehfeld, J.F., Stadil, F., Larson, L.I., Chance, R.E., Moon, N., Pancreaticpolypeptide response to food in duodenal-ulcer patients before and after vagotomy (1976) Lancet, 1 (7969), pp. 1102-1105. , May 22; Dumonceau, J.M., Giostra, E., Bech, C., Spahr, L., Schroft, A., Shah, D., Acute delayed gastric emptying after ablation of atrial fibrillation: Treatment with botulinum toxin injection (2006) Endoscopy, 38 (5), p. 543. , DOI 10.1055/s-2006-925323; Jones, M.P., Maganti, K., A Systematic Review of Surgical Therapy for Gastroparesis (2003) American Journal of Gastroenterology, 98 (10), pp. 2122-2129. , DOI 10.1111/j.1572-0241.2003.07721.x; Bai, R., Patel, D., Di Biase, L., Phrenic nerve injury after catheter ablation: Should we worry about this complication? (2006) J Cardiovasc Electrophysiol, 17 (9), pp. 944-948. , Sep; Bunch, T.J., Bruce, G.K., Mahapatra, S., Mechanisms of phrenic nerve injury during radiofrequency ablation at the pulmonary vein orifice (2005) J Cardiovasc Electrophysiol, 16 (12), pp. 1318-1325. , Dec; Durante-Mangoni, E., Del, V.D., Ruggiero, G., Right diaphragm paralysis following cardiac radiofrequency catheter ablation for inappropriate sinus tachycardia (2003) PACE - Pacing and Clinical Electrophysiology, 26 (3), pp. 783-784. , DOI 10.1046/j.1460-9592.2003.00136.x; Lee, B.-K., Choi, K.-J., Kim, J., Rhee, K.-S., Nam, G.-B., Kim, Y.-H., Right phrenic nerve injury following electrical disconnection of the right superior pulmonary vein (2004) PACE - Pacing and Clinical Electrophysiology, 27 (10), pp. 1444-1446. , DOI 10.1111/j.1540-8159.2004.00652.x; Natale, A., Pisano, E., Shewchik, J., First human experience with pulmonary vein isolation using a through-the-balloon circumferential ultrasound ablation system for recurrent atrial fibrillation (2000) Circulation, 102 (16), pp. 1879-1882. , Oct 17; Sanchez-Quintana, D., Cabrera, J.A., Climent, V., Farre, J., Weiglein, A., Ho, S.Y., How close are the phrenic nerves to cardiac structures? Implications for cardiac interventionalists (2005) Journal of Cardiovascular Electrophysiology, 16 (3), pp. 309-313. , DOI 10.1046/j.1540-8167.2005.40759.x; Kuck, K.H., Furnkranz, A., Cryoballoon ablation of atrial fibrillation (2010) J Cardiovasc Electrophysiol, 21 (12), pp. 1427-1431. , Dec; Franceschi, F., Dubuc, M., Guerra, P.G., Khairy, P., Phrenic nerve monitoring with diaphragmatic electromyography during cryoballoon ablation for atrial fibrillation: The first human application (2011) Heart Rhythm, 8 (7), pp. 1068-1071. , Jul; Marrouche, N.F., Dresing, T., Cole, C., Bash, D., Saad, E., Balaban, K., Pavia, S.V., Natale, A., Circular mapping and ablation of the pulmonary vein for treatment of atrial fibrillation: Impact of different catheter technologies (2002) Journal of the American College of Cardiology, 40 (3), pp. 464-474. , DOI 10.1016/S0735-1097(02)01972-1, PII S0735109702019721; Patel, D., Bailey, S.M., Furlan, A.J., Long-term functional and neurocognitive recovery in patients who had an acute cerebrovascular event secondary to catheter ablation for atrial fibrillation (2010) J Cardiovasc Electrophysiol, 21 (4), pp. 412-417. , Apr; Gaita, F., Caponi, D., Pianelli, M., Radiofrequency catheter ablation of atrial fibrillation: A cause of silent thromboembolism? Magnetic resonance imaging assessment of cerebral thromboembolism in patients undergoing ablation of atrial fibrillation (2010) Circulation, 122 (17), pp. 1667-1673. , Oct 26; Lickfett, L., Hackenbroch, M., Lewalter, T., Cerebral diffusion-weighted magnetic resonance imaging: A tool to monitor the thrombogenicity of left atrial catheter ablation (2006) J Cardiovasc Electrophysiol, 17 (1), pp. 1-7. , Jan; Schrickel, J.W., Lickfett, L., Lewalter, T., Incidence and predictors of silent cerebral embolism during pulmonary vein catheter ablation for atrial fibrillation (2010) Europace, 12 (1), pp. 52-57. , Jan; Deneke, T., Shin, D.I., Balta, O., Post-ablation asymptomatic cerebral lesions-long-term follow-up using magnetic resonance imaging (2011) Heart Rhythm, 8 (11), pp. 1705-1711. , Nov; Sauren, L.D., Van Belle, Y., De Roy, L., Transcranial measurement of cerebral microembolic signals during endocardial pulmonary vein isolation: Comparison of three different ablation techniques (2009) J Cardiovasc Electrophysiol, 20 (10), pp. 1102-1107. , Oct; Bendszus, M., Stoll, G., Silent cerebral ischaemia: Hidden fingerprints of invasive medical procedures (2006) Lancet Neurol, 5 (4), pp. 364-372. , Apr; Kruis, R.W., Vlasveld, F.A., Van Dijk, D., The (un)importance of cerebral microemboli (2010) Semin Cardiothorac Vasc Anesth, 14 (2), pp. 111-118. , Jun; Michaud, G.F., Silent cerebral embolism during catheter ablation of atrial fibrillation: How concerned should we be? (2010) Circulation, 122 (17), pp. 1662-1663. , Oct 26; Cauchemez, B., Extramiana, F., Cauchemez, S., Cosson, S., Zouzou, H., Meddane, M., D'Allonnes, L.R., Houdart, E., High-flow perfusion of sheaths for prevention of thromboembolic complications during complex catheter ablation in the left atrium (2004) Journal of Cardiovascular Electrophysiology, 15 (3), pp. 276-283; Helps, S.C., Parsons, D.W., Reilly, P.L., Gorman, D.F., The effect of gas emboli on rabbit cerebral blood flow (1990) Stroke, 21 (1), pp. 94-99; Krivonyak, G.S., Warren, S.G., Cerebral arterial air embolism treated by a vertical head-down maneuver (2000) Catheterization and Cardiovascular Interventions, 49 (2), pp. 185-187. , DOI 10.1002/(SICI)1522-726X(200002)49:2<185::AID-CCD15>3.0.CO;2-C; Franzen, O.W., Klemm, H., Hamann, F., Koschyk, D., Von Kodolitsch, Y., Weil, J., Meinertz, T., Baldus, S., Mechanisms underlying air aspiration in patients undergoing left atrial catheterization (2008) Catheterization and Cardiovascular Interventions, 71 (4), pp. 553-558. , DOI 10.1002/ccd.21445; Ryu, K.H., Hindman, B.J., Reasoner, D.K., Dexter, F., Heparin reduces neurological impairment after cerebral arterial air embolism in the rabbit (1996) Stroke, 27 (2), pp. 303-309. , Feb discussion 310; Waigand, J., Uhlich, F., Gross, C.M., Thalhammer, C., Dietz, R., Percutaneous treatment of pseudoaneurysms and arteriovenous fistulas after invasive vascular procedures (1999) Catheter Cardiovasc Interv, 47 (2), pp. 157-164. , Jun; Ghaye, B., Szapiro, D., Dacher, J.N., Percutaneous ablation for atrial fibrillation: The role of cross-sectional imaging (2003) Radiographics, 23 (SPEC NO), pp. S19-S33. , Oct discussion S48-50; Augello, G., Vicedomini, G., Saviano, M., Pulmonary vein isolation after circumferential pulmonary vein ablation: Comparison between Lasso and threedimensional electroanatomical assessment of complete electrical disconnection (2009) Heart Rhythm, 6 (12), pp. 1706-1713. , Dec; Hayes, C.R., Keane, D., Safety of atrial fibrillation ablation with novel multielectrode array catheters on uninterrupted anticoagulation-a single-center experience (2010) J Interv Card Electrophysiol, 27 (2), pp. 117-122. , Mar; Hussein, A.A., Martin, D.O., Saliba, W., Radiofrequency ablation of atrial fibrillation under therapeutic international normalized ratio: A safe and efficacious periprocedural anticoagulation strategy (2009) Heart Rhythm, 6 (10), pp. 1425-1429. , Oct; Takahashi, Y., Jais, P., Hocini, M., Acute occlusion of the left circumflex coronary artery during mitral isthmus linear ablation (2005) J Cardiovasc Electrophysiol, 16 (10), pp. 1104-1107. , Oct; Calkins, H., Radiation exposure during radiofrequency catheter ablation procedures (2000) Radiofrequency Catheter Ablation of Cardiac Arrhythmias: Basic Concepts and Clinical Applications, pp. 793-803. , Huang S, ed. Armonk, NY: Futura Publishing Co; Calkins, H., Niklason, L., Sousa, J., El-Atassi, R., Langberg, J., Morady, F., Radiation exposure during radiofrequency catheter ablation of accessory atrioventricular connections (1991) Circulation, 84 (6), pp. 2376-2382. , Dec; Kovoor, P., Ricciardello, M., Collins, L., Uther, J.B., Ross, D.L., Risk to patients from radiation associated with radiofrequency ablation for supraventricular tachycardia (1998) Circulation, 98 (15), pp. 1534-1540; Lindsay, B.D., Eichling, J.O., Ambos, H.D., Cain, M.E., Radiation exposure to patients and medical personnel during radiofrequency catheter ablation for supraventricular tachycardia (1992) Am J Cardiol, 70 (2), pp. 218-223. , Jul 15; Mahesh, M., TFluoroscopy: Patient radiation exposure issues (2001) Radiographics, 21 (4), pp. 1033-1045. , Jul-Aug; Nahass, G.T., Fluoroscopy and the skin: Implications for radiofrequency catheter ablation (1995) Am J Cardiol, 76 (3), pp. 174-176. , Jul 15; Nahass, G.T., Acute radiodermatitis after radiofrequency catheter ablation (1997) Journal of the American Academy of Dermatology, 36 (5 II SUPPL.), pp. 881-884; Perisinakis, K., Damilakis, J., Theocharopoulos, N., Manios, E., Vardas, P., Gourtsoyiannis, N., Accurate assessment of patient effective radiation dose and associated detriment risk from radiofrequency catheter ablation procedures (2001) Circulation, 104 (1), pp. 58-62; Rosenthal, L.S., Beck, T.J., Williams, J., Mahesh, M., Herman, M.G., Dinerman, J.L., Calkins, H., Lawrence, J.H., Acute radiation dermatitis following radiofrequency catheter ablation of atrioventricular nodal reentrant tachycardia (1997) PACE - Pacing and Clinical Electrophysiology, 20 (7), pp. 1834-1839. , DOI 10.1111/j.1540-8159.1997.tb03574.x; Rosenthal, L.S., Mahesh, M., Beck, T.J., Saul, J.P., Miller, J.M., Kay, N., Klein, L.S., Calkins, H., Predictors of fluoroscopy time and estimated radiation exposure during radiofrequency catheter ablation procedures (1998) American Journal of Cardiology, 82 (4), pp. 451-458. , DOI 10.1016/S0002-9149(98)00356-7, PII S0002914998003567; Ector, J., Dragusin, O., Adriaenssens, B., Huybrechts, W., Willems, R., Ector, H., Heidbuchel, H., Obesity Is a Major Determinant of Radiation Dose in Patients Undergoing Pulmonary Vein Isolation for Atrial Fibrillation (2007) Journal of the American College of Cardiology, 50 (3), pp. 234-242. , DOI 10.1016/j.jacc.2007.03.040, PII S0735109707014064; Groot, N.M., Bootsma, M., Van Der Velde, E.T., Schalij, M.J., Three-dimensional catheter positioning during radiofrequency ablation in patients: First application of a real-time position management system (2000) J Cardiovasc Electrophysiol, 11 (11), pp. 1183-1192. , Nov; Macle, L., Jais, P., Scavee, C., Weerasooriya, R., Hocini, M., Shah, D.C., Raybaud, F., Haissaguerre, M., Pulmonary vein disconnection using the LocaLisa three-dimensional nonfluoroscopic catheter imaging system (2003) Journal of Cardiovascular Electrophysiology, 14 (7), pp. 693-697; Schmidt, B., Tilz, R.R., Neven, K., Julian Chun, K.R., Furnkranz, A., Ouyang, F., Remote robotic navigation and electroanatomical mapping for ablation of atrial fibrillation: Considerations for navigation and impact on procedural outcome (2009) Circ Arrhythm Electrophysiol, 2 (2), pp. 120-128. , Apr; Steven, D., Servatius, H., Rostock, T., Reduced fluoroscopy during atrial fibrillation ablation: Benefits of robotic guided navigation (2010) J Cardiovasc Electrophysiol, 21 (1), pp. 6-12. , Jan; Wittkampf, F.H.M., Wever, E.F.D., Derksen, R., Wilde, A.A.M., Ramanna, H., Hauer, R.N.W., Robles, D.M.E.O., LocaLisa: New technique for real-time 3-dimensional localization of regular intracardiac electrodes (1999) Circulation, 99 (10), pp. 1312-1317; Dragusin, O., Weerasooriya, R., Jais, P., Evaluation of a radiation protection cabin for invasive electrophysiological procedures (2007) Eur Heart J, 28 (2), pp. 183-189. , Jan; Luckie, M., Jenkins, N.P., Davidson, N.C., Chauhan, A., Dressler's syndrome following pulmonary vein isolation for atrial fibrillation (2008) Acute Card Care, 10 (4), pp. 234-235; Lambert, T., Steinwender, C., Leisch, F., Hofmann, R., Cardiac tamponade following pericarditis 18 days after catheter ablation of atrial fibrillation (2010) Clin Res Cardiol, 99 (9), pp. 595-597. , Sep; Ahsan, S.Y., Moon, J.C., Hayward, M.P., Chow, A.W., Lambiase, P.D., Constrictive pericarditis after catheter ablation for atrial fibrillation (2008) Circulation, 118 (24), pp. e834-e835. , Dec 9; Koyama, T., Sekiguchi, Y., Tada, H., Comparison of characteristics and significance of immediate versus early versus no recurrence of atrial fibrillation after catheter ablation (2009) Am J Cardiol, 103 (9), pp. 1249-1254. , May 1; Kesek, M., Englund, A., Jensen, S.M., Jensen-Urstad, M., Entrapment of circular mapping catheter in the mitral valve (2007) Heart Rhythm, 4 (1), pp. 17-19. , DOI 10.1016/j.hrthm.2006.09.016, PII S1547527106019667; Mansour, M., Mela, T., Ruskin, J., Keane, D., Successful release of entrapped circumferential mapping catheters in patients undergoing pulmonary vein isolation for atrial fibrillation (2004) Heart Rhythm, 1 (5), pp. 558-561. , DOI 10.1016/j.hrthm.2004.07.004, PII S1547527104003911; Wu, R.C., Brinker, J.A., Yuh, D.D., Berger, R.D., Calkins, H.G., Circular mapping catheter entrapment in the mitral valve apparatus: A previously unrecognized complication of focal atrial fibrillation ablation (2002) Journal of Cardiovascular Electrophysiology, 13 (8), pp. 819-821; Grove, R., Kranig, W., Coppoolse, R., Demand for open heart surgery due to entrapment of a circular mapping catheter in the mitral valve in a patient undergoing atrial fibrillation ablation (2008) Clin Res Cardiol, 97 (9), pp. 628-629. , Sep; Tavernier, R., Duytschaever, M., Taeymans, Y., Fracture of a circular mapping catheter after entrapment in the mitral valve apparatus during segmental pulmonary vein isolation (2003) PACE - Pacing and Clinical Electrophysiology, 26 (8), pp. 1774-1775. , DOI 10.1046/j.1460-9592.2003.t01-1-00268.x; Zeljko, H.M., Mont, L., Sitges, M., Entrapment of the circular mapping catheter in the mitral valve in two patients undergoing atrial fibrillation ablation (2011) Europace, 13 (1), pp. 132-133. , Jan; Naccarelli, G.V., Conti, J.B., DiMarco, J.P., Tracy, C.M., Task force 6: Training in specialized electrophysiology, cardiac pacing, and arrhythmia management endorsed by the Heart Rhythm Society (2008) J Am Coll Cardiol, 51 (3), pp. 374-380. , Jan 22; Green, M.S., Guerra, P.G., Krahn, A.D., 2010 Canadian Cardiovascular Society/Canadian Heart Rhythm Society training standards and maintenance of competency in adult clinical cardiac electrophysiology (2011) Can J Cardiol, 27 (6), pp. 859-861. , Nov; Cox, J.L., The surgical treatment of atrial fibrillation. IV. Surgical technique (1991) J Thorac Cardiovasc Surg, 101 (4), pp. 584-592. , Apr; Cox, J.L., Schuessler, R.B., D'Agostino Jr., H.J., The surgical treatment of atrial fibrillation. III. Development of a definitive surgical procedure (1991) J Thorac Cardiovasc Surg, 101 (4), pp. 569-583. , Apr; Smith, P.K., Holman, W.L., Cox, J.L., Surgical treatment of supraventricular tachyarrhythmias (1985) Surgical Clinics of North America, 65 (3), pp. 553-570; Guiraudon, G., Campbell, C., Jones, D., Combined sinoatrial note atrioventricular node isolation: A surgical alternative to His bundle ablation in patients with atrial fibrillation (1985) Circulation, 72 (SUPPL. 3), p. 220; Cox, J.L., Ad, N., Palazzo, T., Schaff, H.V., Impact of the maze procedure on the stroke rate in patients with atrial fibrillation (1999) Journal of Thoracic and Cardiovascular Surgery, 118 (5), pp. 833-840. , DOI 10.1016/S0022-5223(99)70052-8; Feinberg, M.S., Waggoner, A.D., Kater, K.M., Cox, J.L., Lindsay, B.D., Perez, J.E., Restoration of atrial function after the maze procedure for patients with atrial fibrillation: Assessment by Doppler echocardiography (1994) Circulation, 90 (5 II), pp. II285-II292; McCarthy, P.M., Gillinov, A.M., Castle, L., Chung, M., Cosgrove III, D., The Cox-Maze procedure: The Cleveland Clinic experience (2000) Semin Thorac Cardiovasc Surg, 12 (1), pp. 25-29. , Jan; Prasad, S.M., Maniar, H.S., Camillo, C.J., Schuessler, R.B., Boineau, J.P., Sundt III, T.M., Cox, J.L., Miller, D.C., The Cox maze III procedure for atrial fibrillation: Long-term efficacy in patients undergoing lone versus concomitant procedures (

19. Calkins H, Kuck KH, Cappato R, Brugada J, Camm AJ, Chen SA, et al. 2012 HRS/EHRA/ECAS Expert Consensus Statement on Catheter and Surgical Ablation of Atrial Fibrillation: Recommendations for Patient Selection, Procedural Techniques, Patient Management and Follow-up, Definitions, Endpoints, and Research Trial Design. Heart Rhythm. 2012;9(4):632-96.e21. doi: 10.1016/j.hrthm.2011.12.016.

20. Calkins H, Kuck KH, Cappato R, Brugada J, John Camm A, Chen SA, et al. 2012 HRS/EHRA/ECAS expert consensus statement on catheter and surgical ablation of atrial fibrillation: Recommendations for patient selection, procedural techniques, patient management and follow-up, definitions, endpoints, and research trial design. J Intervent Card Electrophysiol. 2012;33(2):171-257. doi: 10.1007/s10840-012-9672-7.

21. 2013 Air Medical Transport Conference AbstractsScientific Assembly, Monday, October 21, 2013. Air Medical Journal. 2013;32(5):251-60. doi: http://doi.org/10.1016/S1067-991X(13)00161-2.

22. 2013 Critical Care Transport Medicine Conference Scientific Forum. Air Medical Journal. 2013;32(4):194-9. doi: http://doi.org/10.1016/j.amj.2013.04.010.

23. Mancia G, Fagard R, Narkiewicz K, Redon J, Zanchetti A, Böhm M, et al. 2013 ESH/ESC guidelines for the management of arterial hypertension: The Task Force for the management of arterial hypertension of the European Society of Hypertension (ESH) and of the European Society of Cardiology (ESC). Eur Heart J. 2013;34(28):2159-219. doi: 10.1093/eurheartj/eht151

10.1136/bmj; Shufelt, C.L., Bairey Merz, C.N., Contraceptive hormone use and cardiovascular disease (2009) J Am Coll Cardiol, 53, pp. 221-231; (2004) Medical Eligibility Criteria for Contraceptive Use, , World Health Organization. 3rd ed: Geneva: World Health Organization; Lubianca, J.N., Moreira, L.B., Gus, M., Fuchs, F.D., Stopping oral contraceptives: An effective blood pressure-lowering intervention in women with hypertension (2005) J Hum Hypertens, 19, pp. 451-455; No. 73: Use of hormonal contraception in women with coexisting medical conditions (2006) Obstet Gynecol, 107, pp. 1453-1472. , ACOGCommittee on practice bulletin - Gynecology ACOGpractice bulletin; Mosca, L., Benjamin, E.J., Berra, K., Bezanson, J.L., Dolor, R.J., Lloyd-Jones, D.M., Newby, L.K., Wenger, N.K., Effectiveness-based guidelines for the prevention of cardiovascular disease in women: 2011 update: A guideline from the American Heart Association (2011) J Am Coll Cardiol, 57, pp. 1404-1423; Collins, P., Rosano, G., Casey, C., Daly, C., Gambacciani, M., Hadji, P., Kaaja, R., Stramba-Badiale, M., Management of cardiovascular risk in the peri-menopausal woman: A consensus statement of European cardiologists and gynaecologists (2007) Eur Heart J, 28, pp. 2028-2040; Mueck, A.O., Seeger, H., Effect of hormone therapy on BP in normotensive and hypertensive postmenopausal women (2004) Maturitas, 49, pp. 189-203; Regitz-Zagrosek, V., Blomstrom, L.C., Borghi, C., Cifkova, R., Ferreira, R., Foidart, J.M., Gibbs, J.S., Baumgartner, H., ESC Guidelines on the management of cardiovascular diseases during pregnancy: The Task Force on the Management of Cardiovascular Diseases during Pregnancy of the European Society of Cardiology (ESC) (2011) Eur Heart J, 32, pp. 3147-3197; The management of hypertensive disorders during pregnancy (2010) NICE Clinical Guidelines. No. 107, , Hypertension in pregnancy. National Collaborating Centre for Women's and Children's Health (UK). London: RCOG Press, August; Abalos, E., Duley, L., Steyn, D.W., Henderson-Smart, D.J., Antihypertensive drug therapy for mild to moderate hypertension during pregnancy (2001) Cochrane Database Syst Rev, pp. CD002252; Kuklina, E.V., Tong, X., Bansil, P., George, M.G., Callaghan, W.M., Trends in pregnancy hospitalizations that included a stroke in the United States from 1994 to 2007: Reasons for concern? (2011) Stroke, 42, pp. 2564-2570; Martin Jr., J.N., Thigpen, B.D., Moore, R.C., Rose, C.H., Cushman, J., May, W., Stroke and severe preeclampsia and eclampsia: A paradigm shift focusing on systolic blood pressure (2005) Obstet Gynecol, 105, pp. 246-254; Duley, L., Henderson-Smart, D., Knight, M., King, J., Antiplatelet drugs for prevention of pre-eclampsia and its consequences: Systematic review (2001) BMJ, 322, pp. 329-333; Rossi, A.C., Mullin, P.M., Prevention of pre-eclampsia with low-dose aspirin or vitamins C and e in women at high or low risk: A systematic review with meta-analysis (2011) Eur J Obstet Gynecol Reprod Biol, 158, pp. 9-16; Bujold, E., Roberge, S., Lacasse, Y., Bureau, M., Audibert, F., Marcoux, S., Forest, J.C., Giguere, Y., Prevention of preeclampsia and intrauterine growth restriction with aspirin started in early pregnancy: A meta-analysis (2010) Obstet Gynecol, 116, pp. 402-414; Bellamy, L., Casas, J.P., Hingorani, A.D., Williams, D.J., Pre-eclampsia and risk of cardiovascular disease and cancer in later life: Systematic review and meta-analysis (2007) BMJ, 335, p. 974; McDonald, S.D., Malinowski, A., Zhou, Q., Yusuf, S., Devereaux, P.J., Cardiovascular sequelae of preeclampsia/eclampsia: A systematic review and meta-analyses (2008) Am Heart J, 156, pp. 918-930; Beulens, J.W., Patel, A., Vingerling, J.R., Cruickshank, J.K., Hughes, A.D., Stanton, A., Lu, J., Stolk, R.P., Effects of blood pressure lowering and intensive glucose control on the incidence and progression of retinopathy in patients with type 2 diabetes mellitus: A randomised controlled trial (2009) Diabetologia, 52, pp. 2027-2036; Chaturvedi, N., Porta, M., Klein, R., Orchard, T., Fuller, J., Parving, H.H., Bilous, R., Sjolie, A.K., Effect of candesartan on prevention (DIRECT-Prevent 1) and progression (DIRECT-Protect 1) of retinopathy in type 1 diabetes: Randomised, placebocontrolled trials (2008) Lancet, 372, pp. 1394-1402; Watkins, P.J., Edmonds, M.E., (1999) Diabetic Autonomic Failure, , Oxford: University Press; Cederholm, J., Gudbjornsdottir, S., Eliasson, B., Zethelius, B., Eeg-Olofsson, K., Nilsson, P.M., Blood pressure and risk of cardiovascular disease in type 2 diabetes: Further findings from the Swedish National Diabetes Register (NDR-BP-II) (2012) J Hypertens, 30, pp. 2020-2030; Cooper-De Hoff, R.M., Gong, Y., Handberg, E.M., Bavry, A.A., Denardo, S.J., Bakris, G.L., Pepine, C.J., Tight blood pressure control and cardiovascular outcomes among hypertensives patients with diabetes and coronary artery disease (2010) JAMA, 304, pp. 61-68; Schmieder, R.E., Hilgers, K.F., Schlaich, M.P., Schmidt, B.M., Renin-angiotensin system and cardiovascular risk (2007) Lancet, 369, pp. 1208-1219; Alberti, K.G., Eckel, R.H., Grundy, S.M., Zimmet, P.Z., Cleeman, J.I., Donato, K.A., Fruchart, J.C., Smith Jr., S.C., Harmonizing the metabolic syndrome: A joint interim statement of the International Diabetes Federation Task Force on Epidemiology and Prevention; National Heart, Lung and Blood Institute; American Heart Association;World Heart Federation; International Atherosclerosis Society; And International Association for the Study of Obesity (2009) Circulation, 120, pp. 1640-1645; Benetos, A., Thomas, F., Pannier, B., Bean, K., Jego, B., Guize, L., All-cause and cardiovascular mortality using the different definitions of metabolic syndrome (2008) Am J Cardiol, 102, pp. 188-191; Nilsson, P.M., Engstrom, G., Hedblad, B., The metabolic syndromeand incidence of cardiovascular disease in non-diabetic subjects: A population-based study comparing three different definitions (2007) Diabet Med, 24, pp. 464-472; Mancia, G., Bombelli, M., Corrao, G., Facchetti, R., Madotto, F., Giannattasio, C., Trevano, F.Q., Sega, R., Metabolic syndrome in the Pressioni Arteriose Monitorate e Loro Associazioni (PAMELA) study: Daily life blood pressure, cardiac damage and prognosis (2007) Hypertension, 49, pp. 40-47; Shafi, T., Appel, L.J., Miller III, E.R., Klag, M.J., Parekh, R.S., Changes in serum potassium mediate thiazide-induced diabetes (2008) Hypertension, 52, pp. 1022-1029; Tuomilehto, J., Lindstrom, J., Eriksson, J.G., Valle, T.T., Hamalainen, H., Ilanne-Parikka, P., Keinanen-Kiukaanniemi, S., Uusitupa, M., Prevention of type 2 diabetes mellitus by changes in lifestyle among subjects with impaired glucose tolerance (2001) N Engl J Med, 344, pp. 1343-1350; Knowler, W.C., Barrett-Connor, E., Fowler, S.E., Hamman, R.F., Lachin, J.M., Walker, E.A., Nathan, D.M., Reduction in the incidence of type 2 diabetes with lifestyle intervention or metformin (2002) N Engl J Med, 346, pp. 393-403; Parati, G., Lombardi, C., Hedner, J., Bonsignore, M.R., Grote, L., Tkacova, R., Levy, P., McNicholas, W.T., Position paper on the management of patients with obstructive sleep apnea and hypertension: Joint recommendations by the European Society of Hypertension, by the European Respiratory Society and by the members of European COST (Co-operation in Scientific and Technological research) ACTION B26 on obstructive sleep apnea (2012) J Hypertens, 30, pp. 633-646; Bazzano, L.A., Khan, Z., Reynolds, K., He, J., Effect of nocturnal nasal continuous positive airway pressure on blood pressure in obstructive sleep apnea (2007) Hypertension, 50, pp. 417-423; Alajmi, M., Mulgrew, A.T., Fox, J., Davidson, W., Schulzer, M., Mak, E., Ryan, C.F., Ayas, N.T., Impact of continuous positive airway pressure therapy on blood pressure (2007) Lung, 185, pp. 67-72; Mo, L., He, Q.Y., Effect of long-term continuous positive airway pressure ventilation on blood pressure in patients with obstructive sleep apnea hypopnea syndrome: A meta-analysis of clinical trials (2007) Zhonghua Yi Xue Za Zhi, 87, pp. 1177-1180; Haentjens, P., Van Meerhaeghe, A., Moscariello, A., Deweerdt, S., Poppe, K., Dupont, A., Velkeniers, B., The impact of continuous positive airway pressure on blood pressure in patients with obstructive sleep apnea syndrome: Evidence from a meta-analysis of placebo-controlled randomized trials (2007) Arch Intern Med, 167, pp. 757-764; Kasiakogias, A., Tsoufis, C., Thomopoulos, C., Aragiannis, D., Alchanatis, M., Tousoulis, D., Papademetriou, V., Stefanadis, C., Effects of continuous positive airway pressure in hypertensive patients with obstructive sleep apnea: A 3-year follow-up (2013) J Hypertens, 31, pp. 352-360; Barbe, F., Duran-Cantolla, J., Sanchez-De-La-Torre, M., Martinez-Alonso, M., Carmona, C., Barcelo, A., Chiner, E., Montserrat, J.M., Effect of continuous positive airway pressure on the incidence of hypertension and cardiovascular events in nonsleepy patients with obstructive sleep apnea: A randomized controlled trial (2012) JAMA, 307, pp. 2161-2168; Marin, J.M., Agusti, A., Villar, I., Forner, M., Nieto, D., Carrizo, S.J., Barbe, F., Jelic, S., Association between treated and untreated obstructive sleep apnea and risk of hypertension (2012) JAMA, 307, pp. 2169-2176; Zanchetti, A., What should be learnt about the management of obstructive sleep apnea in hypertension? (2012) J Hypertens, 30, pp. 669-670; Klag, M.J., Whelton, P.K., Randall, B.L., Neaton, J.D., Brancati, F.L., Stamler, J., End-stage renal disease in African-American and white men. 16-year MRFIT findings (1997) JAMA, 277, pp. 1293-1298; Yano, Y., Fujimoto, S., Sato, Y., Konta, T., Iseki, K., Moriyama, T., Yamagata, K., Watanabe, T., Association between prehypertension and chronic kidney disease in the Japanese general population (2012) Kidney Int, 81, pp. 293-299; Jafar, T.H., Stark, P.C., Schmid, C.H., Landa, M., Maschio, G., De Jong, P.E., De Zeeuw, D., Levey, A.S., Progression of chronic kidney disease: The role of blood pressure control, proteinuria and angiotensin-converting enzyme inhibition: A patient-level meta-analysis (2003) Ann Intern Med, 139, pp. 244-252; Heerspink, H.J., Ninomiya, T., Zoungas, S., De Zeeuw, D., Grobbee, D.E., Jardine, M.J., Gallagher, M., Perkovic, V., Effect of lowering blood pressure on cardiovascular events and mortality in patients on dialysis: A systematic review and meta-analysis of randomised controlled trials (2009) Lancet, 373, pp. 1009-1015; Lea, J., Greene, T., Hebert, L., Lipkowitz, M., Massry, S., Middleton, J., Rostand, S.G., Bakris, G.L., The relationship between magnitude of proteinuria reduction and risk of end-stage renal disease: Results of the African American study of kidney disease and hypertension (2005) Arch Intern Med, 165, pp. 947-953; De Zeeuw, D., Remuzzi, G., Parving, H.H., Keane, W.F., Zhang, Z., Shahinfar, S., Snapinn, S., Brenner, B.M., Albuminuria, a therapeutic target for cardiovascular protection in type 2 diabetic patients with nephropathy (2004) Circulation, 110, pp. 921-927; Schmieder, R.E., Mann, J.F., Schumacher, H., Gao, P., Mancia, G., Weber, M.A., McQueen, M., Yusuf, S., Changes in albuminuria predict mortality and morbidity in patients with vascular disease (2011) J Am Soc Nephrol, 22, pp. 1353-1364; Kunz, R., Friedrich, C., Wolbers, M., Mann, J.F., Meta-analysis: Effect of monotherapy and combination therapy with inhibitors of the renin angiotensin system on proteinuria in renal disease (2008) Ann Intern Med, 148, pp. 30-48; Ruggenenti, P., Fassi, A., Ilieva, A.P., Iliev, I.P., Chiurchiu, C., Rubis, N., Gherardi, G., Remuzzi, G., Effects of verapamil added-on trandolapril therapy in hypertensive type 2 diabetes patients with microalbuminuria: The BENEDICT-B randomized trial (2011) J Hypertens, 29, pp. 207-216; Bakris, G.L., Serafidis, P.A., Weir, M.R., Dalhof, B., Pitt, B., Jamerson, K., Velazquez, E.J., Weber, M.A., Renal outcomes with different fixed-dose combination therapies in patients with hypertension at high risk for cardiovascular events (ACCOMPLISH): A prespecified secondary analysis of randomised controlled trial (2010) Lancet, 375, pp. 1173-1181. , ACCOMPLISHTrial Investigators; Pisoni, R., Acelajado, M.C., Cartmill, F.R., Dudenbostel, T., Dell'Italia, L.J., Cofield, S.S., Oparil, S., Calhoun, D.A., Long-term effects of aldosterone blockade in resistant hypertension associated with chronic kidney disease (2012) J Hum Hypertens, 26, pp. 502-506; Levin, N.W., Kotanko, P., Eckardt, K.U., Kasiske, B.L., Chazot, C., Cheung, A.K., Redon, J., London, G.M., Blood pressure in chronic kidney disease stage 5D-report from a Kidney Disease: Improving Global Outcomes controversies conference (2010) Kidney Int, 77, pp. 273-284; Potter, J.F., Robinson, T.G., Ford, G.A., Mistri, A., James, M., Chernova, J., Jagger, C., Controlling hypertension and hypotension immediately post-stroke (CHHIPS): A randomised, placebo-controlled, double-blind pilot trial (2009) Lancet Neurology, 8, pp. 48-56; Schrader, J., Luders, S., Kulschewski, A., Berger, J., Zidek, W., Treib, J., Einhaupl, K., Dominiak, P., The ACCESS study: Evaluation of acute candesartan cilexetil therapy in stroke survivors (2003) Stroke, 34, pp. 1699-1703; Sandset, E.C., Bath, P.M., Boysen, G., Jatuzis, D., Korv, J., Luders, S., Murray, G.D., Berge, E., The angiotensin-receptor blocker candesartan for treatment of acute stroke (SCAST): A randomised, placebo-controlled, double-blind trial (2011) Lancet, 377, pp. 741-750; Fuentes Patarroyo, S.X., Anderson, C., Blood pressure lowering in acute phase of stroke, latest evidence and clinical implication (2012) Ther Adv Chronic Dis, 3, pp. 163-171; Gueyffier, F., Boissel, J.P., Boutitie, F., Pocock, S., Coope, J., Cutler, J., Ekbom, T., Schron, E., Effect of antihypertensive treatment in patients having already suffered from stroke. Gathering the evidence. The INDANA (INdividual Data ANalysis of Antihypertensive intervention trials) Project Collaborators (1997) Stroke, 28, pp. 2557-2562; Schrader, J., Luders, S., Kulschewski, A., Hammersen, F., Plate, K., Berger, J., Zidek, W., Diener, H.C., Morbidity and mortality after stroke, eprosartan compared with nitrendipine for secondary prevention: Principal results of a prospective randomized controlled study (MOSES) (2005) Stroke, 36, pp. 1218-1226. , MOSES Study Group; Reboldi, G., Angeli, F., Cavallini, C., Gentile, G., Mancia, G., Verdecchia, P., Comparison between angiotensin-converting enzyme inhibitors and angiotensin receptor blockers on the risk of myocardial infarction, stroke and death: A meta-analysis (2008) J Hypertens, 26, pp. 1282-1289; Ninomiya, T., Ohara, T., Hirakawa, Y., Yoshida, D., Doi, Y., Hata, J., Kanba, S., Kiyohara, Y., Midlife and late-life blood pressure and dementia in Japanese elderly: The Hisayama study (2011) Hypertension, 58, pp. 22-28; Peters, R., Beckett, N., Forette, F., Tuomilehto, J., Clarke, R., Ritchie, C., Waldman, A., Bulpitt, C., Incident dementia and blood pressure lowering in the Hypertension in the Very Elderly Trial cognitive function assessment (HYVET-COG): A double-blind, placebo controlled trial (2008) Lancet Neurology, 7, pp. 683-689; Dufouil, C., Godin, O., Chalmers, J., Coskun, O., McMahon, S., Tzourio-Mazoyer, N., Bousser, M.G., Tzourio, C., Severe cerebral white matter hypersensities predict severe cognitive decline in patients with cerebrovascular disease history (2009) Stroke, 40, pp. 2219-2221; Godin, O., Tsourio, C., Maillard, P., Mazoyer, B., Dufouil, C., Antihypertensive treatment and change in blood pressure are associated with the progression of white matter lesion volumes: The Three-City (3C)-Dijon Magnetic Resonance Imaging Study (2011) Circulation, 123, pp. 266-273; Yusuf, S., Hawken, S., Ounpuu, S., Dans, T., Avezum, A., Lanas, F., McQueen, M., Liu, L., INTERHEART Study Investigators. Effect of potentially modifiable risk factors associated with myocardial infarction in 52 countries (the INTERHEART study): Case-control study (2004) Lancet, 364, pp. 937-952; Body-mass index and cause-specific mortality in 900 000 adults: Collaborative analyses of 57 prospective studies (2009) Lancet, 373, pp. 1083-1096. , Prospective Study Collaboration; Borghi, C., Bacchelli, S., Degli Esposti, D., Bignamini, A., Magnani, B., Ambrosioni, E., Effects of the administration of an angiotensin converting enzyme inhibitor during the acute phase of myocardial infarction in patients with arterial hypertension. SMILE Study Investigators. Survival of Myocardial Infarction Long Term Evaluation (1999) Am J Hypertens, 12, pp. 665-672; Gustafsson, F., Kober, L., Torp-Pedersen, C., Hildebrand, P., Ottesen, M.M., Sonne, B., Carlsen, J., Long-term prognosis after acute myocardial infarction in patients with a history of arterial hypertension (1998) Eur Heart J, 4, pp. 588-594; Tocci, G., Sciarretta, S., Volpe, M., Development of heart failure in recent hypertension trials (2008) J. Hypertens, 26, pp. 1477-1486; Effects of the angiotensin-receptor blocker telmisartan on cardiovascular events in high-risk patients intolerant to angiotensin-converting enzyme inhibitors: A randomised controlled trial (2008) Lancet, 372, pp. 1174-1183. , Telmisartan Randomized Assessment Study in ACE intolerant subjects with cardiovascular disease (TRANSCEND) Investigators. ; Raphael, C.E., Whinnett, Z.I., Davies, J.E., Fontana, M., Ferenczi, E.A., Manisty, C.H., Mayet, J., Francis, D.P., Quantifying the paradoxical effect of higher systolic blood pressure on mortality in chronic heart failure (2009) Heart, 95, pp. 56-62; Massie, B.M., Carson, P.E., McMurray, J.J., Komajda, M., McKelvie, R., Zile, M.R., Anderson, S., Ptaszynska, A., Irbesartan in patients with heart failure and preserved ejection fraction (2008) N Engl J Med, 359, pp. 2456-2467; Camm, A.J., Kirchhof, P., Lip, G.Y., Schotten, U., Savelieva, I., Ernst, S., Van Gelder, I.C., Rutten, F.H., Guidelines for the management of atrial fibrillation: The Task Force for the Management of Atrial Fibrillation of the European Society of Cardiology (ESC) (2010) Eur Heart J, 31, pp. 2369-2429; Grundvold, I., Skretteberg, P.T., Liestol, K., Erikssen, G., Kjeldsen, S.E., Arnesen, H., Erikssen, J., Bodegard, J., Upper normal blood pressures predict incident atrial fibrillation in healthy middle-aged men: A 35-year follow-up study (2012) Hypertension, 59, pp. 198-204; Manolis, A.J., Rosei, E.A., Coca, A., Cifkova, R., Erdine, S.E., Kjeldsen, S., Lip, G.Y., Mancia, G., Hypertension and atrial fibrillation: Diagnostic approach, prevention and treatment. Position paper of the Working Group 'Hypertension Arrhythmias and Thrombosis' of the European Society of Hypertension (2012) J Hypertens, 30, pp. 239-252; Hart, R.G., Pearce, L.A., Aquilar, M.I., Meta-analysis: Antithrombotic therapy to prevent stroke in patients who have nonvalvular atrial fibrillation (2007) Ann Intern Med, 146, pp. 857-867; Camm, A.J., Lip, G.Y., De Caterina, R., Savelieva, I., Atar, D., Hohnloser, S.H., Hindricks, G., Kirchhof, P., 2012 focused update of the ESC Guidelines for the management of atrial fibrillation: An update of the 2010 ESC Guidelines for the management of atrial fibrillation (2012) Eur Heart J, 33, pp. 2719-3274; Arima, H., Anderson, C., Omae, T., Woodward, M., MacMahon, S., Mancia, G., Bousser, M.G., Chalmers, J., Effects of blood pressure lowering on intracranial and extracranial bleeding in patients on antithrombotic therapy: The PROGRESS trial (2012) Stroke, 43, pp. 1675-1677; Wachtell, K., Lehto, M., Gerdts, E., Olsen, M.H., Hornestam, B., Dahlof, B., Ibsen, H., Devereux, R.B., Angiotensin II receptor blockade reduces new-onset atrial fibrillation and subsequent stroke compared with atenolol: The Losartan Intervention for End Point Reduction in Hypertension (LIFE) study (2005) J Am Coll Cardiol, 45, pp. 712-719; Schmieder, R.E., Kjeldsen, S.E., Julius, S., McInnes, G.T., Zanchetti, A., Hua, T.A., Reduced incidence of new-onset atrial fibrillation with angiotensin II receptor blockade: The VALUE trial (2008) J Hypertens, 26, pp. 403-411; Cohn, J.N., Tognoni, G., A randomized trial of the angiotensin-receptor blocker valsartan in chronic heart failure (2001) N Engl J Med, 345, pp. 1667-1675; Vermes, E., Tardif, J.C., Bourassa, M.G., Racine, N., Levesque, S., White, M., Guerra, P.G., Ducharme, A., Enalapril decreases the incidence of atrial fibrillation in patients with left ventricular dysfunction: Insight from the Studies of Left Ventricular Dysfunction (SOLVD) trials (2003) Circulation, 107, pp. 2926-2931; Ducharme, A., Swedberg, K., Pfeffer, M.A., Cohen-Solal, A., Granger, C.B., Maggioni, A.P., Michelson, E.L., Yusuf, S., Prevention of atrial fibrillation in patients with symptomatic chronic heart failure by candesartan in the Candesartan in Heart failure: Assessment of Reduction in Mortality and morbidity (CHARM) program (2006) Am Heart J, 152, pp. 86-92; Irbesartan in patients with atrial fibrillation (2011) N Engl J Med, 364, pp. 928-938. , The Active I Investigators; Tveit, A., Grundvold, I., Olufsen, M., Seljeflot, I., Abdelnoor, M., Arnesen, H., Smith, P., Candesartan in the prevention of relapsing atrial fibrillation (2007) Int J Cardiol, 120, pp. 85-91; Valsartan for prevention of recurrent atrial fibrillation (2009) N Engl J Med, 360, pp. 1606-1617; Goette, A., Schon, N., Kirchhof, P., Breithardt, G., Fetsch, T., Hausler, K.G., Klein, H.U., Meinertz, T., Angiotensin II-antagonist in paroxysmal atrial fibrillation(ANTIPAF) trial (2012) Circulation Arrhythmia and Electrophysiology, 5, pp. 43-51; Schneider, M.P., Hua, T.A., Bohm, M., Wachtell, K., Kjeldsen, S.E., Schmieder, R.E., Prevention of atrial fibrillation by renin-angiotensin system inhibition: A meta-analysis (2010) J Am Coll Cardiol, 55, pp. 2299-2307; Nasr, I.A., Bouzamondo, A., Hulot, J.S., Dubourg, O., Le Heuzey, J.Y., Lechat, P., Prevention of atrial fibrillation onset by beta-blocker treatment in heart failure: A meta-analysis (2007) Eur Heart J, 28, pp. 457-462; Swedberg, K., Zannad, F., McMurray, J.J., Krum, H., Van Veldhuisen, D.J., Shi, H., Vincent, J., Pitt, B., EMPHASIS-HF Study Investigators. Eplerenone and atrial fibrillation in mild systolic heart failure: Results from the EMPHASIS-HF (Eplerenone in Mild Patients Hospitalization and Surv Ival Study in Heart Failure) study (2012) J Am Coll Cardiol, 59, pp. 1598-1603; Schaer, B.A., Schneider, C., Jick, S.S., Conen, D., Osswald, S., Meier, C.R., Risk for incident atrial fibrillation in patients who receive antihypertensive drugs: A nested casecontrol study (2010) Ann Intern Med, 152, pp. 78-84; Fagard, R.H., Celis, H., Thijs, L., Wouters, S., Regression of left ventricular mass by antihypertensive treatment: A meta-analysis of randomized comparative studies (2009) Hypertension, 54, pp. 1084-1091; Zanchetti, A., Crepaldi, G., Bond, M.G., Gallus, G., Veglia, F., Mancia, G., Ventura, A., Magni, A., Different effects of antihypertensive regimens based on fosinopril or hydrochlorothiazide with or without lipid lowering by pravastatin on progression of asymptomatic carotid atherosclerosis: Principal results of PHYLLIS: A randomized double-blind trial (2004) Stroke, 35, pp. 2807-2812; Ong, K.T., Delerme, S., Pannier, B., Safar, M.E., Benetos, A., Laurent, S., Boutouyrie, P., Aortic stiffness is reduced beyond blood pressure lowering by short-term and long-term antihypertensive treatment: A meta-analysis of individual data in 294 patients (2011) J Hypertens, 29, pp. 1034-1042; Shahin, Y., Khan, J.A., Chetter, I., Angiotensin converting enzyme inhibitors effect on arterial stiffness and wave reflections: A meta-analysis and meta-regression of randomised controlled trials (2012) Atherosclerosis, 221, pp. 18-33; Karalliedde, J., Smith, A., Deangelis, L., Mirenda, V., Kandra, A., Botha, J., Ferber, P., Viberti, G., Valsartan improves arterial stiffness in type 2 diabetes independently of blood pressure lowering (2008) Hypertension, 51, pp. 1617-1623; Ait Oufella, H., Collin, C., Bozec, E., Ong, K.T., Laloux, B., Boutouyrie, P., Laurent, S., Longterm reduction in aortic stiffness: A 5.3 year follow-up in routine clinical practice (2010) J Hypertens, 28, pp. 2336-2340; Guerin, A.P., Blacher, J., Pannier, B., Marchais, S.J., Safar, M.E., London, G.M., Impact of aortic stiffness attenuation on survival of patients in end-stage renal failure (2001) Circulation, 103, pp. 987-992; Singer, D.R., Kite, A., Management of hypertension in peripheral arterial disease: Does the choice of drugs matter? (2008) Eur J Vasc Endovasc Surg, 35, pp. 701-708; Effects of an angiotensin-converting-enzyme inhibitor, ramipril, on cardiovascular events in high-risk patients (2000) N Engl J Med, 342, pp. 145-153. , The Heart Outcomes Prevention Evaluation Study Investigators; Paravastu, S.C., Mendonca, D.A., Da Silva, A., Beta blockers for peripheral arterial disease (2009) Eur J Vasc Endovasc Surg, 38, pp. 66-70; Radack, K., Deck, C., Beta-adrenergic blocker therapy does not worsen intermittent claudication in subjects with peripheral arterial disease. A meta-analysis of randomized controlled trials (1991) Arch Intern Med, 151, pp. 1769-1776; Dong, J.Y., Zhang, Y.H., Qin, L.Q., Erectile dysfunction and risk of cardiovascular disease: Meta-analysis of prospective cohort studies (2011) J Am Coll Cardiol, 58, pp. 1378-1385; Gupta, B.P., Murad, M.H., Clifton, M.M., Prokop, L., Nehra, A., Kopecky, S.L., The effect of lifestyle modification and cardiovascular risk factor reduction on erectile dysfunction: A systematic review and meta-analysis (2011) Arch Intern Med, 171, pp. 1797-1803; Manolis, A., Doumas, M., Sexual dysfunction: The 'prima ballerina' of hypertensionrelated quality-of-life complications (2008) J Hypertens, 26, pp. 2074-2084; Pickering, T.G., Shepherd, A.M., Puddey, I., Glasser, D.B., Orazem, J., Sherman, N., Mancia, G., Sildenafil citrate for erectile dysfunction in men receiving multiple antihypertensive agents: A randomized controlled trial (2004) Am J Hypertens, 17, pp. 1135-1142; Scranton, R.E., Lawler, E., Botteman, M., Chittamooru, S., Gagnon, D., Lew, R., Harnett, J., Gaziano, J.M., Effect of treating erectile dysfunction on management of systolic hypertension (2007) Am J Cardiol, 100, pp. 459-463; Ma, R., Yu, J., Xu, D., Yang, L., Lin, X., Zhao, F., Bai, F., Effect of felodipine with irbesartan or metoprolol on sexual function and oxidative stress in women with essential hypertension (2012) J Hypertens, 30, pp. 210-216; Fagard, R.H., Resistant hypertension (2012) Heart, 98, pp. 254-261; Dela Sierra, A., Segura, J., Banegas, J.R., Gorostidi, M., De La Cruz, J.J., Armario, P., Oliveras, A., Ruilope, L.M., Clinical features of 8295 patients with resistant hypertension classified on the basis of ambulatory blood pressure monitoring (2011) Hypertension, 57, pp. 171-174; Daugherty, S.L., Powers, J.D., Magid, D.J., Tavel, H.M., Masoudi, F.A., Maragolis, K.L., O'Connor, P.J., Ho, P.M., Incidence and prognosis of resistant hypertension in hypertensive patients (2012) Circulation, 125, pp. 1635-1642; Persell, S.D., Prevalence of resistant hypertension in the United States, 2003-2008 (2011) Hypertension, 57, pp. 1076-1080; Mantero, F., Mattarello, M.J., Albiger, N.M., Detecting and treating primary aldosteronism: Primary aldosteronism (2007) Exp Clin Endocrinol Diabetes, 115, pp. 171-174; Redon, J., Campos, C., Narciso, M.L., Rodicio, J.L., Pascual, J.M., Ruilope, L.M., Prognostic value of ambulatory blood pressure monitoring in refractory hypertension: A prospective study (1998) Hypertension, 31, pp. 712-718; Yakovlevitch, M., Black, H.R., Resistant hypertension in a tertiary care clinic (1991) Arch Intern Med, 151, pp. 1786-1792; Zannad, F., Aldosterone antagonist therapy in resistant hypertension (2007) J Hypertens, 25, pp. 747-750; Lane, D.A., Shah, S., Beevers, D.G., Low-dose spironolactone in the management of resistant hypertension: A surveillance study (2007) J Hypertens, 25, pp. 891-894; Vaclavik, J., Sedlak, R., Plachy, M., Navratil, K., Plasek, J., Jarkovsky, J., Vaclavik, T., Taborsky, M., Addition of spironolactone in patients with resistant arterial hypertension (ASPIRANT): A randomized, double-blind, placebo-controlled trial (2011) Hypertension, 57, pp. 1069-1075; Chapman, N., Chang, C.L., Dahlof, B., Sever, P.S., Wedel, H., Poulter, N.R., Effect of doxazosin gastrointestinal therapeutic system as third-line antihypertensive therapy on blood pressure and lipids in the Anglo-Scandinavian Cardiac Outcomes Trial (2008) Circulation, 118, pp. 42-48; Bobrie, G., Frank, M., Azizi, M., Peyrard, S., Boutouyrie, P., Chatellier, G., Laurent, S., Plouin, P.F., Sequential nephron blockade vs. Sequential renin-angiotensin system blockade in resistant hypertension: A prospective, randomized, open blinded endpoint study (2012) J Hypertens, 30, pp. 1656-1664; Gaddam, K.K., Nishizaka, M.K., Pratt-Ubunama, M.N., Pimenta, E., Aban, I., Oparil, S., Calhoun, D.A., Characterization of resistant hypertension: Association between resistant hypertension, aldosterone and persistent intravascular volume expansion (2008) Arch Intern Med, 168, pp. 1159-1164; Lijnen, P., Staessen, J., Fagard, R., Amery, A., Increase in plasma aldosterone during prolonged captopril treatment (1982) Am J Cardiol, 49, pp. 1561-1563; Weber, M.A., Black, H., Bakris, G., Krum, H., Linas, S., Weiss, R., Linseman, J.V., Lindholm, L.H., A selective endothelin-receptor antagonist to reduce blood pressure in patients with treatment-resistant hypertension: A randomised, double-blind, placebo-controlled trial (2009) Lancet, 374, pp. 1423-1431; Bakris, G.L., Lindholm, L.H., Black, H.R., Krum, H., Linas, S., Linseman, J.V., Arterburn, S., Weber, M., Divergent results using clinic and ambulatory blood pressures: Report of a darusentan-resistant hypertension trial (2010) Hypertension, 56, pp. 824-830; Laurent, S., Schlaich, M., Esler, M., New drugs procedures and devices for hypertension (2012) Lancet, 380, pp. 591-600; Bisognano, J.D., Bakris, G., Nadim, M.K., Sanchez, L., Kroon, A.A., Schafer, J., De Leeuw, P.W., Sica, D.A., Baroreflex activation therapy lowers blood pressure in patients with resistant hypertension: Results from the double-blind, randomized, placebocontrolled rheos pivotal trial (2011) J Am Coll Cardiol, 58, pp. 765-773; Bakris, G.L., Nadim, M.K., Haller, H., Lovett, E.G., Schafer, J.E., Bisognano, J.D., Baroreflex activation therapy provides durable benefit in patients with resistant hypertension: Results of long-termfollow-up in the Rheos Pivotal Trial (2012) J Am Soc Hypertens, 6, pp. 152-158; Hoppe, U.C., Brandt, M.C., Wachter, R., Beige, J., Rump, L.C., Kroon, A.A., Cates, A.W., Haller, H., Minimally invasive system for baroreflex activation therapy chronically lowers blood pressure with pacemaker-like safety profile: Results from the Barostim Neo trial (2012) J Am Soc Hypertens, 6, pp. 270-276; Krum, H., Schlaich, M., Whitbourn, R., Sobotka, P.A., Sadowski, J., Bartus, K., Kapelak, B., Esler, M., Catheter-based renal sympathetic denervation for resistant hypertension: A multicentre safety and proof-of-principle cohort study (2009) Lancet, 373, pp. 1275-1281; (2011) Hypertension, 57, pp. 911-917. , Simplicity HTN-1 Investigators Catheter-based renal sympathetic denervation for resistant hypertension: durability of blood pressure reduction out to 24 months; Renal sympathetic denervation in patients with treatment-resistant hypertension (The Symplicity HTN-2 Trial): A randomised controlled trial (2010) Lancet, 376, pp. 1903-1909. , Simplicity HTN-Investigators; Krum, H., Barman, N., Schlaich, M., Sobotka, P., Esler, M., Mahfoud, F., Böhm, M., Straley, C., Long-term follow up of catheterbased renal sympathetic denervation for resistant hypertension confirms durable blood pressure reduction (2012) J Am Coll Cardiol, 59 (13), pp. E1704-E1704. , doi: 10.1016/S0735-1097(12)61705-7; Geisler, B.P., Egan, B.M., Cohen, J.T., Garner, A.M., Akehurst, R.L., Esler, M.D., Pietsch, J.B., Cost-effectiveness and clinical effectiveness of catheter-based renal denervation for resistant hypertension (2012) J Am Coll Cardiol, 60, pp. 1271-1277; Esler, M., Lambert, G., Jenningis, G., Regional norepinephrine turnover in human hypertension (1989) Clin Exp Hypertens, 11 (SUPPL. 1), pp. 75-89; Grassi, G., Cattaneo, B.M., Seravalle, G., Lanfranchi, A., Mancia, G., Baroreflex control of sympathetic nerve activity in essential and secondary hypertension (1998) Hypertension, 31, pp. 68-72; Grassi, G., Seravalle, G., Dell'Oro, R., Turri, C., Bolla, G.B., Mancia, G., Adrenergic and reflex abnormalities in obesity-related hypertension (2000) Hypertension, 36, pp. 538-542; Stella, A., Zanchetti, A., Functional role of renal afferents (1991) Physiol Rev, 71, pp. 659-682; Dibona, G.F., Kopp, U.C., Neural control of renal function (1997) Physiol Rev, 77, pp. 75-197; Doumas, M., Anyfanti, P., Bakris, G., Should ambulatory blood pressure monitoring be mandatory for future studies in resistant hypertension: A perspective (2012) Hypertension, 30, pp. 874-876; Brandt, M.C., Mahfoud, F., Reda, S., Schirmer, S.H., Erdmann, E., Böhm, M., Hoppe, U.C., Renal sympathetic denervation reduces left ventricular hypertrophy and improves cardiac function in patients with resistant hypertension (2012) J Am Coll Cardiol, 59, pp. 901-909; Mahfoud, F., Schlaich, M., Kindermann, I., Ukena, C., Cremers, B., Brandt, M.C., Hoppe, U.C., Böhm, M., Effect of renal sympathetic denervation on glucose metabolism in patients with resistant hypertension: A pilot study (2011) Circulation, 123, pp. 1940-1946; Mahfoud, F., Cremers, B., Janker, J., Link, B., Vonend, O., Ukena, C., Linz, D., Böhm, M., Renal haemodynamics and renal function after catheter-based renal sympathetic denervation in patients with resistant hypertension (2012) Hypertension, 60, pp. 419-424; Schmieder, R.E., Redon, J., Grassi, G., Kjeldsen, S.E., Mancia, G., Narkiewicz, K., Parati, G., Tsioufis, C., ESH position paper: Renal denervation: An interventional therapy of resistant hypertension (2012) J Hypertens, 30, pp. 837-841; Frank, H., Heusser, K., Geiger, H., Fahlbuscg, R., Naraghi, R., Schobel, H.P., Temporary reduction of blood pressure and sympathetic nerve activity in hypertensive patients after microvascular decompression (2009) Stroke, 40, pp. 47-51; Zhang, Y., Zhang, X., Liu, L., Wang, Y., Tang, X., Zanchetti, A., Higher cardiovascular risk and impaired benefit of antihypertensve treatment in hypertensive patients requiring additional drugs on top of randomized therapy: Is adding drugs always beneficial? (2012) J Hypertens, 30, pp. 2202-2212. , FEVER Study Group; Weber, M.A., Julius, S., Kjeldsen, S.E., Jia, Y., Brunner, H.R., Zappe, D.H., Hua, T.A., Zanchetti, A., Cardiovascular outcomes in hypertensive patients: Comparing single-agent therapy with combination therapy (2012) J Hypertens, 30, pp. 2213-2222; Lane, D.A., Lip, G.Y., Beevers, D.G., Improving survival of malignant hypertension patients over 40 years (2009) Am J Hypertens, 22, pp. 1199-1204; Gosse, P., Coulon, P., Papaioannou, G., Litalien, J., Lemetayer, P., Impact of malignant arterial hypertension on the heart (2011) J Hypertens, 29, pp. 798-802; Gonzalez, R., Morales, E., Segura, J., Ruilope, L.M., Praga, M., Long-term renal survival in malignant hypertension (2010) Nephrol Dial Transplant, 25, pp. 3266-3272; Casadei, B., Abuzeid, H., Is there a strong rationale for deferring elective surgery in patients with poorly controlled hypertension? (2005) J Hypertens, 23, pp. 19-22; Manolis, A.J., Erdine, S., Borghi, C., Tsioufis, K., Perioperative screening and management of hypertensive patients (2010) European Society of Hypertension Scientific Newsletter, 11, p. 2; Pearce, J.D., Craven, B.L., Craven, T.E., Piercy, K.T., Stafford, J.M., Edwards, M.S., Hansen, K.J., Progression of atherosclerotic renovascular disease: A prospective populationbased study (2006) J Vasc Surg, 44, pp. 955-962; Safian, R.D., Textor, S.C., Renal-artery stenosis (2001) N Engl J Med, 344, pp. 431-442; Gray, B.H., Olin, J.W., Childs, M.B., Sullivan, T.M., Bacharach, J.M., Clinical benefit of renal artery angioplasty with stenting for the control of recurrent and refractory congestive heart failure (2002) Vasc Med, 7, pp. 275-279; Wheatley, K., Ives, N., Gray, R., Kalra, P.A., Moss, J.G., Baigent, C., Carr, S., Scoble, J., Revascularization vs. Medical therapy for renal-artery stenosis (2009) N Engl J Med, 361, pp. 1953-1962; Funder, J.W., Carey, R.M., Fardella, C., Gomez-Sanchez, C.E., Mantero, F., Stowasser, M., Young Jr., W.F., Montori, V.M., Case detection, diagnosis and treatment of patients with primary aldosteronism: An endocrine society clinical practice guideline (2008) J Clin Endocrinol Metab, 93, pp. 3266-3281; Sawka, A.M., Young, W.F., Thompson, G.B., Grant, C.S., Farley, D.R., Leibson, C., Van Heerden, J.A., Primary aldosteronism: Factors associated with normalization of blood pressure after surgery (2001) Ann Intern Med, 135, pp. 258-261; Rossi, G.P., Bolognesi, M., Rizzoni, D., Seccia, T.M., Piva, A., Porteri, E., Tiberio, G.A., Pessina, A.C., Vascular remodeling and duration of hypertension predict outcome of adrenalectomy in primary aldosteronism patients (2008) Hypertension, 51, pp. 1366-1371; Parthasarathy, H.K., Menard, J., White, W.B., Young Jr., W.F., Williams, G.H., Williams, B., Ruilope, L.M., MacDonald, T.M., Adouble-blind, randomized study comparing the antihypertensive effect of eplerenone and spironolactone in patients with hypertension and evidence of primary aldosteronism (2011) J Hypertens, 29, pp. 980-990; Chapman, M.J., Ginsberg, H.N., Amarenco, P., Andreotti, F., Borén, J., Catapano, A.L., Descamps, O.S., Watts, G.F., Triglyceride-rich lipoproteins and high-density lipoprotein cholesterol in patients at high risk of cardiovascular disease: Evidence and guidance for management (2011) Eur Heart J, 32, pp. 1345-1361. , and for the European Atherosclerosis Society Consensus Panel; Sever, P.S., Dahlof, B., Poulter, N.R., Wedel, H., Beevers, G., Caulfield, M., Collins, R., Ostergren, J., ASCOT Investigators. Prevention of coronary and stroke events with atorvastatin in hypertensive patients who have average or lower-than-average cholesterol concentrations, in the Anglo-Scandinavian Cardiac Outcomes Trial: Lipid Lowering Arm (ASCOT-LLA): A multicentre randomised controlled trial (2003) Lancet, 361, pp. 1149-1158; The antihypertensive and lipid lowering treatment to prevent heart attack trial. Major outcomes in moderately hypercholesterolemic, hypertensive patients randomized to pravastatin vs usual care: The Antihypertensive and Lipid-Lowering Treatment to Prevent Heart Attack Trial (ALLHAT-LLT) (2002) JAMA, 288, pp. 2998-3007. , ALLHATofficers and co-ordinators for the ALLHAT collaborative research group; Sever, P.S., Poulter, N.R., Dahlof, B., Wedel, H., ASCOT Investigators. Antihypertensive therapy and the benefits of atorvastatin in the Anglo-Scandinavian Cardiac Outcomes Trial: Lipid-lowering arm extension (2009) J Hypertens, 27, pp. 947-954; Ridker, P.M., Danielson, E., Fonseca, F.A., Genest, J., Gotto Jr., A.M., Kastelein, J.J., Koenig, W., Glynn, R.J., JUPITER Study Group. Rosuvastatin to prevent vascular events in men and women with elevated C-reactive protein (2008) N Engl J Med, 359, pp. 2195-2207; Reiner, Z., Catapano, A.L., De Backer, G., Graham, I., Taskinen, M.-R., Wiklund, O., Agewall, S., Wood, D., ESC/EAS Guidelines for the management of dyslipidaemias: The Task Force for the management of dyslipidaemias of the European Society of Cardiology ESC) and the European Atherosclerosis Society (EAS) (2011) Eur Heart J, 32, pp. 1769-1818; Baigent, C., Blackwell, L., Emberson, J., Holland, L.E., Reith, C., Bhala, N., Peto, R., Collins, R., Efficacy and safety of more intensive lowering of LDL cholesterol: A meta-analysis of data from 170, 000 participants in 26 randomised trials (2010) Lancet, 376, pp. 1670-1681; Amarenco, P., Bogousslavsky, J., Callahan III, A., Goldstein, L.B., Hennerici, M., Rudolph, A.E., Sillesen, H., Zivin, J.A., Stroke Prevention by Aggressive Reduction in Cholesterol Levels (SPARCL) investigators. Highdose atorvastatin after stroke or transient ischemic attack (2006) N Engl J Med, 355, pp. 549-559; Taylor, F., Ward, K., Moore, T.H., Burke, M., Davey Smith, G., Casas, J.P., Ebrahim, S., Statins for the primary prevention of cardiovascular disease (2011) Cochrane Database Syst Rev, 19, pp. CD004816. , Jan; Baigent, C., Blackwell, L., Collins, R., Emberson, J., Godwin, J., Peto, R., Buring, J., Zanchetti, A., Aspirin in the primary and secondary prevention of vascular disease: Collaborative meta-analysis of individual participant data from randomised trials (2009) Lancet, 373, pp. 1849-1860; Jardine, M.J., Ninomiya, T., Perkovic, V., Cass, A., Turnbull, F., Gallagher, M.P., Zoungas, S., Zanchetti, A., Aspirin is beneficial in hypertensive patients with chronic kidney disease: A post-hoc subgroup analysis of a randomized controlled trial (2010) J Am Coll Cardiol, 56, pp. 956-965; Rothwell, P.M., Price, J.F., Fowkes, F.G., Zanchetti, A., Roncaglioni, M.C., Tognoni, G., Lee, R., Meade, T.W., Short-term effects of daily aspirin on cancer incidence, mortality and non-vascular death: Analysis of the time course of risks and benefits in 51 randomised controlled trials (2012) Lancet, 379, pp. 1602-1612; Nathan, D.M., Cleary, P.A., Backlund, J.Y., Genuth, S.M., Lachin, J.M., Orchard, T.J., Raskin, P., Zinman, B., Intensive diabetes treatment and cardiovascular disease in patients with type 1 diabetes (2005) N Engl J Med, 353, pp. 2643-2653; Polak, J.F., Backlund, J.Y., Cleary, P.A., Harrington, A.P., O'Leary, D.H., Lachin, J.M., Nathan, D.M., Progression of carotid artery intimamedia thickness during 12 years in the Diabetes Control and Complications Trial/Epidemiology of Diabetes Interventions and Complications (DCCT/EDIC) study (2011) Diabetes, 60, pp. 607-613. , DCCT/EDIC Research Group; Intensive blood-glucose control with sulphonylureas or insulin compared with conventional treatment and risk of complications in patients with type 2 diabetes (UKPDS 33) (1998) Lancet, 352, pp. 837-853. , UK Prospective Diabetes Study (UKPDS ) Group; Effect of intensive blood-glucose control with metformin on complications in overweight patients with type 2 diabetes (UKPDS 34) (1998) Lancet, 352, pp. 854-865. , UK Prospective Diabetes Study (UKPDS) Group; Intensive blood glucose control and vascular outcomes in patients with type 2 diabetes (2008) N Engl J Med, 358, pp. 2560-2572. , ADVANCE Collaborative Group; Gerstein, H.C., Miller, M.E., Genuth, S., Ismail-Beigi, F., Buse, J.B., Goff Jr., D.C., Probstfield, J.L., Friedewald, W.T., Long-term effects of intensive glucose lowering on cardiovascular outcomes (2011) N Engl J Med, 364, pp. 818-828; Duckworth, W., Abraira, C., Moritz, T., Reda, D., Emanuele, N., Reaven, P.D., Zieve, F.J., Huang, G.D., Glucose control and vascular complications in veterans with type 2 diabetes (2009) N Engl J Med, 360, pp. 129-139; Ray, K.K., Seshasai, S.R., Wijesuriya, S., Sivakumaran, R., Nethercott, S., Preiss, D., Erqou, S., Sattar, N., Effect of intensive control of glucose on cardiovascular outcomes and death in patients with diabetes mellitus: A meta-analysis of randomised controlled trials (2009) Lancet, 373, pp. 1765-1772; Turnbull, F.M., Abraira, C., Anderson, R.J., Byington, R.P., Chalmers, J.P., Duckworth, W.C., Evans, G.W., Woodward, M., Intensive glucose control and macrovascular outcomes in type 2 diabetes (2009) Diabetologia, 52, pp. 2288-2298; Hemmingsen, B., Lund, S.S., Gluud, C., Vaag, A., Almdal, T., Hemmingsen, C., Wetterslev, J., Intensive glycaemic control for patients with type 2 diabetes: Systematic review with meta-analysis and trial sequential analysis of randomised clinical trials (2011) BMJ, 343, pp. d6898; Inzucchi, S.E., Bergenstal, R.M., Buse, J.B., Diamant, M., Ferrannini, E., Nauck, M., Peters, A.L., Matthews, D.R., Management of hyperglycaemia in type 2 diabetes: A patient-centreed approach. Position statement of the American Diabetes Association (ADA) and the European Association for the Study of Diabetes (EASD) (2012) Diabetologia, 55, pp. 1577-1596; Ferrannini, E., Solini, A., SGLT2 inhibition in diabetes mellitus: Rationale and clinical prospects (2012) Nature Rev Endocrinol, 8, pp. 495-502; ESC/EASD Guidelines on diabetes, pre-diabetes and cardiovascular diseases (2013) Eur Heart J, , doi: 10.1093/eurheartj/eht108; Birtwhistle, R.V., Godwin, M.S., Delva, M.D., Casson, R.I., Lam, M., MacDonald, S.E., Seguin, R., Ruhland, L., Randomised equivalence trial comparing three month and six month follow up of patients with hypertension by family practitioners (2004) BMJ, 328, p. 204; Clark, C.E., Smith, L.F., Taylor, R.S., Campbell, J.L., Nurse led interventions to improve control of blood pressure in people with hypertension: Systematic review and meta-analysis (2010) BMJ, 341, pp. c3995; Niiranen, T.J., Hanninen, M.R., Johansson, J., Reunanen, A., Jula, A.M., Home-measured blood pressure is a stronger predictor of cardiovascular risk than office blood pressure: The Finn-Home study (2010) Hypertension, 55, pp. 1346-1351; Bray, E.P., Holder, R., Mant, J., McManus, R.J., Does self-monitoring reduce blood pressure? Meta-analysis with meta-regression of randomized controlled trials (2010) Ann Med, 42, pp. 371-386; McManus, R.J., Mant, J., Bray, E.P., Holder, R., Jones, M.I., Greenfield, S., Kaambwa, B., Hobbs, F.D., Telemonitoring and selfmanagement in the control of hypertension (TASMINH2): A randomised controlled trial (2010) Lancet, 376, pp. 163-172; Gupta, A.K., McGlone, M., Greenway, F.L., Johnson, W.D., Prehypertension in diseasefree adults: A marker for an adverse cardiometabolic risk profile (2010) Hypertens Res, 33, pp. 905-910; Thompson, A.M., Hu, T., Eshelbrenner, C.L., Reynolds, K., He, J., Bazzano, L.A., Antihypertensive treatment and secondary prevention of cardiovascular disease events among persons without hypertension: A meta-analysis (2011) JAMA, 305, pp. 913-922; Sacks, F.M., Svetkey, L.P., Vollmer, W.M., Appel, L.J., Bray, G.A., Harsha, D., Obarzanek, E., Lin, P.H., Effects on blood pressure of reduced dietary sodium and the Dietary Approaches to Stop Hypertension (DASH) diet. DASH-Sodium Collaborative Research Group (2001) N Engl J Med, 344, pp. 3-10; Viera, A.J., Bangura, F., Mitchell, C.M., Cerna, A., Sloane, P., Dophysicians tell patients they have prehypertension? (2011) J Am Board Family Med, 24, pp. 117-118; Faria, C., Wenzel, M., Lee, K.W., Coderre, K., Nichols, J., Belletti, D.A., Anarrative reviewof clinical inertia: Focus on hypertension (2009) J Am Soc Hypert, 3, pp. 267-276; Kearney, P.M., Whelton, M., Reynolds, K., Whelton, P.K., He, J., Worldwide prevalence of hypertension: A systematic review (2004) J Hypertens, 22, pp. 11-19; Muiesan, M.L., Salvetti, M., Paini, A., Monteduro, C., Galbassini, G., Bonzi, B., Poisa, P., Agabiti Rosei, E., Inappropriate left ventricular mass changes during treatment adversely affects cardiovascular prognosis in hypertensive patients (2007) Hypertension, 49, pp. 1077-1083; Okin, P.M., Oikarinen, L., Viitasalo, M., Toivonen, L., Kjeldsen, S.E., Nieminen, M.S., Edelman, J.M., Devereux, R.B., Serial assessment of the electrocardiographic strain pattern for prediction of new-onset heart failure during antihypertensive treatment: The LIFE study (2011) Eur J Heart Fail, 13, pp. 384-391; Gerdts, E., Wachtell, K., Omvik, P., Otterstad, J.E., Oikarinen, L., Boman, K., Dahlof, B., Devereux, R.B., Left atrial size and risk of major cardiovascular events during antihypertensive treatment: Losartan intervention for endpoint reduction in hypertension trial (2007) Hypertension, 49, pp. 311-316; Olsen, M.H., Wachtell, K., Ibsen, H., Lindholm, L.H., Dahlof, B., Devereux, R.B., Kjeldsen, S.E., Okin, P.M., Reductions in albuminuria and in electrocardiographic left ventricular hypertrophy independently improve prognosis in hypertension: The LIFE study (2006) J Hypertens, 24, pp. 775-781; Atkins, R.C., Briganti, E.M., Lewis, J.B., Hunsicker, L.G., Braden, G., Champion De Crespigny, P.J., Deferrari, G., Lewis, E.J., Proteinuria reduction and progression to renal failure in patients with type 2 diabetes mellitus and overt nephropathy (2005) Am J Kidney Dis, 45, pp. 281-287; Costanzo, P., Perrone-Filardi, P., Vassallo, E., Paolillo, S., Cesarano, P., Brevetti, G., Chiariello, M., Does carotid intima-media thickness regression predict reduction of cardiovascular events? A meta-analysis of 41 randomized trials (2010) J Am Coll Cardiol, 56, pp. 2006-2020; Goldberger, Z.D., Valle, J.A., Dandekar, V.K., Chan, P.S., Ko, D.T., Nallamothu, B.K., Are changes in carotid intima-media thickness related to risk of nonfatal myocardial infarction? A critical review and meta-regression analysis (2010) Am Heart J, 160, pp. 701-714; Lorenz, M.W., Polak, J.F., Kavousi, M., Mathiesen, E.B., Voelzke, H., Tuomainen, T.P., Sander, D., Thompson, S.G., Carotid intima-media tickness progression to predict cardiovascular events in the general population (the PROG-IMT collaborative project): A meta-analysis of individuial participant data Lancet, 379, pp. 2053-2062. , PROG-IMT Study Group; Bots, M.L., Taylor, A.J., Kastelein, J.J., Peters, S.A., Den Ruijter, H.M., Tegeler, C.H., Baldassarre, D., Grobbee, D.E., Rate of exchange in carotid intima-media thickness and vascular events: Meta-analyses can not solve all the issues. A point of view (2012) J Hypertens, 30, pp. 1690-1696; Burt, V.L., Cutler, J.A., Higgins, M., Horan, M.J., Labarthe, D., Whelton, P., Brown, C., Roccella, E.J., Trends in the prevalence, awareness, treatment and control of hypertension in the adult US population. Data from the Health Examination Surveys, 1960 to 1991 (1995) Hypertension, 26, pp. 60-69; Reiner, Z., Sonicki, Z., Tedeschi-Reiner, E., Physicians' perception. Knowledge and awareness of cardiovacsulr risk factors and adherence to prevention guidelines: The PERCRO-DOC survey (2010) Atherosclerosis, 213, pp. 598-603; Amar, J., Chamontin, B., Genes, N., Cantet, C., Salvador, M., Cambou, J.P., Why is hypertension so frequently uncontrolled in secondary prevention? (2003) J Hypertens, 21, pp. 1199-1205; Mancia, G., Ambrosioni, E., Agabiti Rosei, E., Leonetti, G., Trimarco, B., Volpe, M., Blood pressure control and risk of stroke in untreated and treated hypertensive patients screened from clinical practice: Results of the for Life study (2005) J Hypertens, 23, pp. 1575-1581; Benetos, A., Thomas, F., Bean, K.E., Guize, L., Why cardiovascular mortality is higher in treated hypertensives vs. Subjects of the same age, in the general population (2003) J Hypertens, 21, pp. 1635-1640; Redon, J., Cea-Calvo, L., Lozano, J.V., Marti-Canales, J.C., Llisterri, J.L., Aznar, J., Gonzalez-Esteban, J., Differences in blood pressure control and stroke mortality across Spain: The Prevencion de Riesgo de Ictus (PREV-ICTUS) study (2007) Hypertension, 49, pp. 799-805; Kotseva, K., Wood, D., Debacker, G., Debacquer, D., Pyorala, K., Keil, U., Cardiovascular prevention guidelines in daily practice: A comparison of EUROASPIRE I, II and III surveys in eight European countries (2009) Lancet, 373, pp. 929-940; Bhatt, D.L., Steg, P.G., Ohman, E.M., Hirsch, A.T., Ikeda, Y., Mas, J.L., Goto, S., Wilson, P.W., International prevalence, recognition and treatment of cardiovascular risk factors in outpatients with atherothrombosis (2006) JAMA, 295, pp. 180-189; Cooper-De Hoff, R.M., Handberg, E.M., Mancia, G., Zhou, Q., Champion, A., Legler, U.F., Pepine, C.J., INVEST revisited: Review of findings from the International Verapamil SR-Trandolapril Study (2009) Expert Rev Cardiovasc Ther, 7, pp. 1329-1340; Ezzati, M., Lopez, A.D., Rodgers, A., Vander Hoorn, S., Murray, C.J., Selected major risk factors and global and regional burden of disease (2002) Lancet, 360, pp. 1347-1360; Banegas, J.R., Segura, J., Ruilope, L.M., Luque, M., Garcia-Robles, R., Campo, C., Rodriguez-Artalejo, F., Tamargo, J., Blood pressure control and physician management of hypertension in hospital hypertension units in Spain (2004) Hypertension, 43, pp. 1338-1344; Corrao, G., Zambon, A., Parodi, A., Poluzzi, E., Baldi, I., Merlino, L., Cesana, G., Mancia, G., Discontinuation of and changes in drug therapy for hypertension among newlytreated patients: A population-based study in Italy (2008) J Hypertens, 26, pp. 819-824; Lee, J.K., Grace, K.A., Taylor, A.J., Effect of a pharmacy care program on medication adherence and persistence, blood pressure and low-density lipoproteincholesterol: A randomized controlled trial (2006) JAMA, 296, pp. 2563-2571; Gale, N.K., Greenfield, S., Gill, P., Gutridge, K., Marshall, T., Patient and general practitioner attitudes to taking medication to prevent cardiovascular disease after receiving detailed information on risks and benefits of treatment: A qualitative study (2011) BMC Family Practice, 12, p. 59; Shanti, M., Maribel, S., (2003) Hypertension, pp. 98-104. , World Health Organization; Krousel-Wood, M., Joyce, C., Holt, E., Muntner, P., Webber, L.S., Morisky, D.E., Frohlich, E.D., Re, R.N., Predictors of decline in medication adherence: Results from the cohort study of medication adherence among older adults (2011) Hypertension, 58, pp. 804-810; Corrao, G., Parodi, A., Nicotra, F., Zambon, A., Merlino, L., Cesana, G., Mancia, G., Better compliance to antihypertensive medications reduces cardiovascular risk (2011) J Hypertens, 29, pp. 610-618; Mazzaglia, G., Ambrosioni, E., Alacqua, M., Filippi, A., Sessa, E., Immordino, V., Borghi, C., Mantovani, L.G., Adherence to antihypertensive medications and cardiovascular morbidity among newly diagnosed hypertensive patients (2009) Circulation, 120, pp. 1598-1605; Vrijens, B., Vincze, G., Kristanto, P., Urquhart, J., Burnier, M., Adherence to prescribed antihypertensive drug treatments: Longitudinal study of electronically compiled dosing histories (2008) BMJ, 336, pp. 1114-1117; Redon, J., Coca, A., Lazaro, P., Aguilar, M.D., Cabanas, M., Gil, N., Sanchez-Zamorano, M.A., Aranda, P., Factors associated with therapeutic inertia in hypertension: Validation of a predictive model (2010) J Hypertens, 28, pp. 1770-1777; Luders, S., Schrader, J., Schmieder, R.E., Smolka, W., Wegscheider, K., Bestehorn, K., Improvement of hypertension management by structured physician education and feedback system: Cluster randomized trial (2010) Eur J Cardiovasc Prev Rehabil, 17, pp. 271-279; De Rivas, B., Barrios, V., Redon, J., Calderon, A., Effectiveness of an Interventional Program to Improve Blood Pressure Control in Hypertensive Patients at High Risk for Developing Heart Failure: HEROIC study (2010) J Clin Hypertens (Greenwich), 12, pp. 335-344; Guthrie, B., Inkster, M., Fahey, T., Tackling therapeutic inertia: Role of treatment data in quality indicators (2007) BMJ, 335, pp. 542-544; Claxton, A.J., Cramer, J., Pierce, C., A systematic review of the associations between dose regimens and medication compliance (2001) Clin Ther, 23, pp. 1296-1310; Ashworth, M., Medina, J., Morgan, M., Effect of social deprivation on blood pressure monitoring and control in England: A survey of data from the quality and outcomes framework (2008) BMJ, 337, pp. a2030; Serumaga, B., Ross-Degnan, D., Avery, A.J., Elliott, R.A., Majumdar, S.R., Zhang, F., Soumerai, S.B., Effect of pay for performance on the management and outcomes of hypertension in the United Kingdom: Interrupted time series study (2011) BMJ, 342, pp. d108; Campbell, S.M., Reeves, D., Kontopantelis, E., Sibbald, B., Roland, M., Effects of pay for performance on the quality of primary care in England (2009) N Engl J Med, 361, pp. 368-378; Fahey, T., Schroeder, K., Ebrahim, S., Educational and organisational interventions used to improve the management of hypertension in primary care: A systematic review (2005) Br J Gen Pract, 55, pp. 875-882; Weingarten, S.R., Henning, J.M., Badamgarav, E., Knight, K., Hasselblad, V., Gano Jr., A., Ofman, J.J., Interventions used in disease management programmes for patients with chronic illness-which ones work? Meta-analysis of published reports (2002) BMJ, 325, p. 925; Carter, B.L., Bosworth, H.B., Green, B.B., The hypertension team: The role of the pharmacist, nurse and teamwork in hypertension therapy (2012) J Clin Hypertens (Greenwich), 14, pp. 51-65; Chodosh, J., Morton, S.C., Mojica, W., Maglione, M., Suttorp, M.J., Hilton, L., Rhodes, S., Shekelle, P., Meta-analysis: Chronic disease self-management programs for older adults (2005) Ann Intern Med, 143, pp. 427-438; Carter, B.L., Rogers, M., Daly, J., Zheng, S., James, P.A., The potency of team-based care interventions for hypertension: A meta-analysis (2009) Arch Intern Med, 169, pp. 1748-1755; Walsh, J.M., McDonald, K.M., Shojania, K.G., Sundaram, V., Nayak, S., Lewis, R., Owens, D.K., Goldstein, M.K., Quality improvement strategies for hypertension management: A systematic review (2006) Med Care, 44, pp. 646-657; Glynn, L.G., Murphy, A.W., Smith, S.M., Schroeder, K., Fahey, T., Interventions used to improve control of blood pressure in patients with hypertension (2010) Cochrane Database Syst Rev, pp. CD005182; MacHado, M., Bajcar, J., Guzzo, G.C., Einarson, T.R., Sensitivity of patient outcomes to pharmacist interventions. Part II: Systematic review and meta-analysis in hypertension management (2007) Ann Pharmacother, 41, pp. 1770-1781; Morak, J., Kumpusch, H., Hayn, D., Modre-Osprian, R., Schreier, G., Design and evaluation of a telemonitoring concept based on NFC-enabled mobile phones and sensor devices (2012) IEEE Transactions on Information Technology in Biomedicine: A Publication of the IEEE Engineering in Medicine and Biology Society, 16, pp. 17-23; Canzanello, V.J., Jensen, P.L., Schwartz, L.L., Wona, J.B., Klein, L.K., Inferred blood pressure control with a physician-nurse team and home BP measurement (2005) Mayo Clin Proc, 80, pp. 31-36; Stergiou, G., Myers, M.G., Reid, J.L., Burnier, M., Narkiewicz, K., Viigimaa, M., Mancia, G., Setting-up a blood pressure and vascular protection clinic: Requirements of the European Society of Hypertension (2010) J Hypertens, 28, pp. 1780-1781; Shea, K., Chamoff, B., Telehomecare communication and self-care in chronic conditions: Moving toward a shared understanding (2012) Worldviews on Evidence-based Nursing/Sigma Theta Tau International, Honor Society of Nursing, 9, pp. 109-116; Parati, G., Omboni, S., Albini, F., Piantoni, L., Giuliano, A., Revera, M., Illyes, M., Mancia, G., Home blood pressure telemonitoring improves hypertension control in general practice. The Tele BPCare study (2009) J Hypertens, 27, pp. 198-203; Neumann, C.L., Menne, J., Rieken, E.M., Fischer, N., Weber, M.H., Haller, H., Schulz, E.G., Blood pressure telemonitoring is useful to achieve blood pressure control in inadequately treated patients with arterial hypertension (2011) J Hum Hypertens, 25, pp. 732-738; Omboni, S., Guarda, A., Impact of home blood pressure telemonitoring and blood pressure control: A meta-analysis of randomized controlled studies (2011) Am J Hypertens, 24, pp. 989-998; Russell, M., Roe, B., Beech, R., Russell, W., Service developments for managing people with long-term conditions using case management approaches, an example from the UK (2009) International J Integrated Care, 9, pp. e02.

24. Zipes DP, Calkins H, Daubert JP, Ellenbogen KA, Field ME, Fisher JD, et al. 2015 ACC/AHA/HRS Advanced Training Statement on Clinical Cardiac Electrophysiology (A Revision of the ACC/AHA 2006 Update of the Clinical Competence Statement on Invasive Electrophysiology Studies, Catheter Ablation, and Cardioversion). Circ Arrhythmia Electrophysiol. 2015;8(6):1522-51. doi: 10.1161/HAE.0000000000000014.

25. Zipes DP, Calkins H, Daubert JP, Ellenbogen KA, Field ME, Fisher JD, et al. 2015 ACC/AHA/HRS advanced training statement on clinical cardiac electrophysiology (A revision of the ACC/AHA 2006 update of the clinical competence statement on invasive electrophysiology studies, catheter ablation, and cardioversion). Heart Rhythm. 2016;13(1):e3-e37. doi: 10.1016/j.hrthm.2015.09.014.

26. 2016 ASE 27th Annual Scientific Sessions. Journal of the American Society of Echocardiography. 2016;29(6):B1-B130. doi: http://doi.org/10.1016/j.echo.2016.04.008.

27. Syeda-Mahmood T, Wang F, Beymer D, Amir A, Richmond M, Hashmi SN, editors. AALIM: Multimodal mining for cardiac decision support. Computers in Cardiology 2007, CAR 2007; 2007; Durham, NC.

28. Pfeifer B, Seger M, Hintermüller C, Fischer G, Mühlthaler H, Modre-Osprian R, et al. AAM-based segmentation for imaging cardiac electrophysiology. METHODS INF MED. 2007;46(1):36-42.

29. Price RR. The AAPM/RSNA physics tutorial for residents: MR imaging safety considerations. Radiographics. 1999;19(6):1641-51.

30. Mahesh M, Cody DD. AAPM/RSNA physics tutorial for residents: Physics of cardiac imaging with multiple-row detector CT. Radiographics. 2007;27(5):1495-509. doi: 10.1148/rg.275075045.

31. Cody DD, Mahesh M. AAPM/RSNA physics tutorial for residents: Technologic advances in multidetector CT with a focus on cardiac imaging. Radiographics. 2007;27(6):1829-37. doi: 10.1148/rg.276075120.

32. Price RR. The AAPM/RSNA physics tutorial for residents. MR imaging safety considerations. Radiological Society of North America. Radiographics. 1999;19(6):1641-51. Epub 1999/11/11. doi: 10.1148/radiographics.19.6.g99no331641. PubMed PMID: 10555679.

33. Abstract Session A ASE Young Investigator Research Award. Journal of the American Society of Echocardiography. 1993;6(3, Part 2):S7-S40. doi: http://doi.org/10.1016/S0894-7317(14)80408-2.

34. Schiel U. Abstractions in semantic networks: axiom schemata for generalization, aggregation and grouping. SIGART Bull. 1989;(107):25-6. doi: 10.1145/65751.65752.

35. Abstracts of the 5th International Conference for Emergency Nurses, 13-15 October 2005, Coogee Beach, Sydney, Australia. Australasian Emergency Nursing Journal. 2005;8(3):85-130. doi: http://doi.org/10.1016/j.aenj.2005.08.004.

36. Abstracts of the 26th congress of ESPEN, the European society for clinical nutrition ; metabolism. Clinical Nutrition. 2004;23(4):757-944. doi: http://doi.org/10.1016/j.clnu.2004.06.003.

37. Abtracts Diagnosis, assessment, and reviews. The Journal of Pain. 2003;4(2, Supplement):1-104. doi: http://doi.org/.

38. Jortberg B, Myers E, Gigliotti L, Ivens BJ, Lebre M, Burke March S, et al. Academy of Nutrition and Dietetics: Standards of Practice and Standards of Professional Performance for Registered Dietitian Nutritionists (Competent, Proficient, and Expert) in Adult Weight Management. Journal of the Academy of Nutrition and Dietetics. 2015;115(4):609-18.e40. doi: http://doi.org/10.1016/j.jand.2014.12.018.

39. Epstein AE, DiMarco JP, Ellenbogen KA, Estes Iii NAM, Freedman RA, Gettes LS, et al. ACC/AHA/HRS 2008 Guidelines for Device-Based Therapy of Cardiac Rhythm Abnormalities. Heart Rhythm. 2008;5(6):e1-e62. doi: 10.1016/j.hrthm.2008.04.014.

40. Epstein AE, DiMarco JP, Ellenbogen KA, Estes Iii NAM, Freedman RA, Gettes LS, et al. ACC/AHA/HRS 2008 Guidelines for Device-Based Therapy of Cardiac Rhythm Abnormalities. A Report of the American College of Cardiology/American Heart Association Task Force on Practice Guidelines (Writing Committee to Revise the ACC/AHA/NASPE 2002 Guideline Update for Implantation of Cardiac Pacemakers and Antiarrhythmia Devices). J Am Coll Cardiol. 2008;51(21):e1-e62. doi: 10.1016/j.jacc.2008.02.032.

41. Spartano NL, Heffernan KS, Dumas AK, Gump BB. Accelerometer-determined physical activity and the cardiovascular response to mental stress in children. Journal of Science and Medicine in Sport. 2017;20(1):60-5. doi: http://doi.org/10.1016/j.jsams.2016.05.008.

42. Häggman H. Accident and Emergency in a shipping line. Accident and Emergency Nursing. 1993;1(2):104-7. doi: http://doi.org/10.1016/0965-2302(93)90057-7.

43. Hoyle RJ, Walker KJ, Thomson G, Bailey M. Accuracy of electrocardiogram interpretation improves with emergency medicine training. EMA Emerg Med Australas. 2007;19(2):143-50. doi: 10.1111/j.1742-6723.2007.00946.x.

44. Brown LH, Gough JE, Hawley CR. Accuracy of rural EMS provider interpretation of three-lead ECG rhythm strips. Prehospital Emergency Care. 1997;1(4):259-62. doi: 10.1080/10903129708958820.

45. Henpraserttae A, Thiemjarus S, Marukatat S, editors. Accurate activity recognition using a mobile phone regardless of device orientation and location. 8th International Conference on Body Sensor Networks, BSN 2011; 2011; Dallas, TX.

46. Egbogah EE, Fapojuwo AO, editors. Achieving energy efficient transmission in wireless body area networks for the physiological monitoring of military soldiers. 2013 IEEE Military Communications Conference, MILCOM 2013; 2013; San Diego, CA.

47. Bruining N, Caiani E, Chronaki C, Guzik P, Van Der Velde E. Acquisition and analysis of cardiovascular signals on smartphones: Potential, pitfalls and perspectives: By the Task Force of the e-Cardiology Working Group of European Society of Cardiology. Eur J Prev Cardiol. 2014;21:4-13. doi: 10.1177/2047487314552604.

48. Rolskov BS, Räder S, Holst A, Kayser L, Ringsted C, Hastrup SJ, et al. The acquisition and retention of ECG interpretation skills after a standardized web-based ECG tutorial-a randomised study. BMC medical education [Internet]. 2016; 15:[36 p.]. Available from: http://onlinelibrary.wiley.com/o/cochrane/clcentral/articles/631/CN-01109631/frame.html.

49. Botchen RP, Bachthaler S, Schick F, Chen M, Mori G, Weiskopf D, et al. Action-based multifield video visualization. IEEE Trans Visual Comput Graphics. 2008;14(4):885-99. doi: 10.1109/TVCG.2008.40.

50. Inamdar RS, Ramdasi DS, editors. Active appearance models for segmentation of cardiac MRI data. 2nd International Conference on Communication and Signal Processing, ICCSP 2013; 2013; Melmaruvathur, Tamilnadu.

51. Tan F, Polglaze T, Dawson B. Activity profiles and physical demands of elite women's water polo match play. J SPORTS SCI. 2009;27(10):1095-104. doi: 10.1080/02640410903207416.

52. Almalki M, Gray K, Martin-Sanchez F. Activity theory as a theoretical framework for health self-quantification: A systematic review of empirical studies. J Med Internet Res. 2016;18(5). doi: 10.2196/jmir.5000.

53. Sriram JC, Shin M, Choudhury T, Kotz D, editors. Activity-aware ECG-based patient authentication for remote health monitoring. International Conference on Multimodal Interfaces and the Workshop on Machine Learning for Multimodal Interfaces, ICMI-MLMI'09; 2009; Cambridge, MA.

54. Noble KA. Acute Coronary Syndrome: Evidence-Based Practice in Action. Journal of PeriAnesthesia Nursing. 2011;26(4):284-9. doi: http://doi.org/10.1016/j.jopan.2011.05.005.

55. Acute renal failure of obscure etiology. The American Journal of Medicine. 1961;30(3):464-71. doi: http://doi.org/10.1016/0002-9343(61)90054-7.

56. Khriesat I, Najada AH. Acute rheumatic fever without early carditis: An atypical clinical presentation. Eur J Pediatr. 2003;162(12):868-71. doi: 10.1007/s00431-003-1320-x.

57. Aziz ZA, Lee YYL, Ngah BA, Sidek NN, Looi I, Hanip MR, et al. Acute Stroke Registry Malaysia, 2010-2014: Results from the National Neurology Registry. Journal of Stroke and Cerebrovascular Diseases. 2015;24(12):2701-9. doi: http://doi.org/10.1016/j.jstrokecerebrovasdis.2015.07.025.

58. Novak G, Carlson D, Jarzabek S, editors. An adaptable and extensible mobile sensing framework for patient monitoring. 9th IEEE International Conference on Intelligent Sensors, Sensor Networks and Information Processing, IEEE ISSNIP 2014; 2014; Singapore: IEEE Computer Society.

59. Augustyniak P, editor Adaptive architecture for assisted living systems. 2013 6th International Conference on Human System Interactions, HSI 2013; 2013; Gdansk, Sopot.

60. Sussman MS, Robert N, Wright GA. Adaptive averaging for improved SNR in real-time coronary artery MRI. IEEE Trans Med Imaging. 2004;23(8):1034-45. doi: 10.1109/TMI.2004.828677.

61. Laguna P, Jané R, Masgrau E, Caminal P. The adaptive linear combiner with a periodic-impulse reference input as a linear comb filter. Signal Process. 1996;48(3):193-203. doi: 10.1016/0165-1684(95)00135-2.

62. Shuai J, Shen C, Zhu Z. Adaptive Morphological Feature Extraction and Support Vector Regressive Classification for Bearing Fault Diagnosis. Int J Rotating Machinery. 2017;2017. doi: 10.1155/2017/2384184.

63. Widrow B, Williams CS, Glover JR, Jr., McCool JM, Hearn RH, Zeidler JR, et al. Adaptive Noise Cancelling: Principles and Applications. Proc IEEE. 1975;63(12):1692-716. doi: 10.1109/PROC.1975.10036.

64. Li X, Wang Y. Adaptive online monitoring for ICU patients by combining just-in-time learning and principal component analysis. J Clin Monit Comput. 2016;30(6):807-20. doi: 10.1007/s10877-015-9778-4.

65. Winkley J, Jiang P. Adaptive probability scheme for behaviour monitoring of the elderly using a specialised ambient device. Intl J Mach Learn Cybern. 2014;5(2):293-307. doi: 10.1007/s13042-012-0134-4.

66. Kwok H, Coult J, Drton M, Rea TD, Sherman L. Adaptive rhythm sequencing: A method for dynamic rhythm classification during CPR. Resuscitation. 2015;91:26-31. doi: 10.1016/j.resuscitation.2015.02.031.

67. Saleh M, editor Adaptive ubiquitous mobile gaming system for youth obesity rehabilitation. 14th Annual Wireless Telecommunications Symposium, WTS 2015; 2015: IEEE Computer Society.

68. Wang Y, Qin L, Shi X, Zeng Y, Jing H, Schoepf UJ, et al. Adenosine-stress dynamic myocardial perfusion imaging with second-generation dual-source CT: Comparison with conventional catheter coronary angiography and SPECT nuclear myocardial perfusion imaging. Am J Roentgenol. 2012;198(3):521-9. doi: 10.2214/AJR.11.7830.

69. Popek C, Schaeffer R. An adult male with facial swelling, erythema, and sensation of arm tightness. Journal of Emergency Nursing. 2000;26(6):633-4. doi: http://doi.org/10.1067/men.2000.110197.

70. Advance Program for 2005 Emergency Nurses. Journal of Emergency Nursing. 2005;31(3):A27-A54. doi: http://doi.org/10.1016/j.jen.2005.05.002.

71. Abdul Rahman H, Ge D, Le Faucheur A, Prioux J, Carrault G. Advanced classification of ambulatory activities using spectral density distances and heart rate. Biomed Signal Process Control. 2017;34:9-15. doi: 10.1016/j.bspc.2016.12.018.

72. Kelley FJ, Kopac CA, Rosselli J. Advanced Health Assessment in Nurse Practitioner Programs: Follow-Up Study. Journal of Professional Nursing. 2007;23(3):137-43. doi: http://doi.org/10.1016/j.profnurs.2006.12.005.

73. Martinez MW. Advanced Imaging of Athletes: Added Value of Coronary Computed Tomography and Cardiac Magnetic Resonance Imaging. Clin Sports Med. 2015;34(3):433-48. doi: 10.1016/j.csm.2015.02.005.

74. Walton-Moss B, O’Neill S, Holland W, Hull R, Marineau L. Advanced practice nursing students: Pilot test of a simulation scenario. Collegian. 2012;19(3):171-6. doi: http://doi.org/10.1016/j.colegn.2012.06.002.

75. Lee JD. Advanced practice provider utilization in the neurocritical care unit. CONTINUUM Lifelong Learn Neurol. 2015;21(5):1451-4.

76. Strauss JM, Jehle DVK, Berlioz BE. Advancements at the bedside: Diagnostic and therapeutic tools. Clin Pract. 2014;11(6):689-97. doi: 10.2217/cpr.14.73.

77. Lin Z, De Chen JZ. Advances in time-frequency analysis of biomedical signals. CRIT REV BIOMED ENG. 1997;24(1):1-72.

78. Craft J, Christensen M, Bakon S, Wirihana L. Advancing student nurse knowledge of the biomedical sciences: A mixed methods study. Nurse Education Today. 2017;48:114-9. doi: http://doi.org/10.1016/j.nedt.2016.10.003.

79. Chang IB, Cho HW, Jeong SH, Yang SJ, editors. AED training algorithm for the CPR simulator. 2011 International Conference on Advanced Mechatronic Systems, ICAMechS 2011; 2011; Zhengzhou.

80. Rotstein A, Sagiv M, Ben-Sira D, Werber G, Hutzler J, Annenburg H. Aerobic capacity and anaerobic threshold of wheelchair basketball players. Paraplegia. 1994;32(3):196-201. doi: 10.1038/sc.1994.36.

81. McArdle WD, Magel JR, Kyvallos LC. Aerobic capacity, heart rate and estimated energy cost during women’s competitive basketball. Res Q Am Assoc Health, Phys Educ Recreat. 1971;42(2):178-86. doi: 10.1080/10671188.1971.10615055.

82. Casajus JA, Castagna C. Aerobic fitness and field test performance in elite Spanish soccer referees of different ages. Journal of Science and Medicine in Sport. 2007;10(6):382-9. doi: http://doi.org/10.1016/j.jsams.2006.08.004.

83. French WJ. AFib in special populations. Am J Med. 2014;127(4):e17-8. Epub 2014/03/25. doi: 10.1016/j.amjmed.2013.06.005. PubMed PMID: 24655745.

84. Cohen LB, DeLegge MH, Aisenberg J, Brill JV, Inadomi JM, Kochman ML, et al. AGA Institute Review of Endoscopic Sedation. Gastroenterology. 2007;133(2):675-701. doi: 10.1053/j.gastro.2007.06.002.

85. Epstein JH, Levin M, Jowell MS, editors. Agent based simulation for training and assessing students in the field of anesthesiology. 26th IEEE International Symposium on Computer-Based Medical Systems, CBMS 2013; 2013; Porto.

86. Natarajan P, Gold NB, Bick AG, McLaughlin H, Kraft P, Rehm HL, et al. Aggregate penetrance of genomic variants for actionable disorders in European and African Americans. Sci Transl Med. 2016;8(364). doi: 10.1126/scitranslmed.aag2367.

87. Hiemke C, Baumann P, Bergemann N, Conca A, Dietmaier O, Egberts K, et al. AGNP consensus guidelines for therapeutic drug monitoring in psychiatry: Update 2011. Pharmacopsychiatry. 2011;44(6):195-235. doi: 10.1055/s-0031-1286287.

88. Hinchey PR, De Maio VJ, Patel A, Cabañas JG. Air medical providers' physiological response to a simulated trauma scenario. Air Medical Journal. 2011;30(2):86-90+2. doi: 10.1016/j.amj.2010.11.002.

89. Greenleaf JE, Rehrer NJ, Mohler SR, Quach DT, Evans DG. Airline chair-rest deconditioning: Induction of immobilisation thromboemboli? SPORTS MED. 2004;34(11):705-25. doi: 10.2165/00007256-200434110-00002.

90. Airmed conference abstract 2000. Air Medical Journal. 2001;20(2):23-31. doi: http://doi.org/10.1016/S1067-991X(01)70090-9.

91. Merzougui R, Feham M, editors. Algorithm of remote monitoring ECG using mobile phone: Conception and implementation. 3rd International Conference on Broadband Communications, Informatics and Biomedical Applications, BroadCom 2008; 2008; Pretoria, Gauteng.

92. Fang F, Sanderson JE, Yu CM. All Heart Block Patients with a Pacemaker Indication Should Receive Biventricular Pacing: One Move, Double the Gains? Circ Arrhythmia Electrophysiol. 2015;8(3):722-8. doi: 10.1161/CIRCEP.114.000626.

93. Neubert FR. An allopath at sea. British Homoeopathic journal. 1945;35(1):47-51. doi: http://doi.org/10.1016/S0007-0785(45)80030-3.

94. Whyte G, Sharma S, George K, McKenna WJ. Alterations in cardiac morphology and function in elite multi-disciplinary athletes. INT J SPORTS MED. 1999;20(4):222-6. doi: 10.1055/s-2007-971121.

95. Tesarz J, Gerhardt A, Schommer K, Treede R-D, Eich W. Alterations in endogenous pain modulation in endurance athletes: An experimental study using quantitative sensory testing and the cold-pressor task. PAIN®. 2013;154(7):1022-9. doi: http://doi.org/10.1016/j.pain.2013.03.014.

96. Perez RP, Perez JJ, Betancur MJ, Bustamante J, editors. An alternative light based signal apparatus for generating biological patterns: Oximetry. 8th Pan American Health Care Exchanges Conference, PAHCE 2013; 2013; Medellin.

97. Roy N, Misra A, Cook D. Ambient and smartphone sensor assisted ADL recognition in multi-inhabitant smart environments. J Ambient Intell Humanized Comput. 2016;7(1):1-19. doi: 10.1007/s12652-015-0294-7.

98. Wac K, Tsiourti C. Ambulatory assessment of affect: Survey of sensor systems for monitoring of autonomic nervous systems activation in emotion. IEEE Trans Affective Comput. 2014;5(3):251-72. doi: 10.1109/TAFFC.2014.2332157.

99. Hashimoto F, Appenzeller O, Abrams J, Qualls C. Ambulatory electrocardiographic monitoring at high altitude. Journal of Wilderness Medicine. 1992;3(4):358-66. doi: http://doi.org/10.1580/0953-9859-3.4.358.

100. Mulvagh SL, Rakowski H, Vannan MA, Abdelmoneim SS, Becher H, Bierig SM, et al. American Society of Echocardiography Consensus Statement on the Clinical Applications of Ultrasonic Contrast Agents in Echocardiography. Journal of the American Society of Echocardiography. 2008;21(11):1179-201. doi: http://doi.org/10.1016/j.echo.2008.09.009.

101. Ainon RN, Bulgiba AM, Lahsasna A. AMI screening using linguistic fuzzy rules. J Med Syst. 2012;36(2):463-73. doi: 10.1007/s10916-010-9491-2.

102. Sorber J, Shin M, Peterson R, Cornelius C, Mare S, Prasad A, et al., editors. An Amulet for trustworthy wearable mHealth. 13th Workshop on Mobile Computing Systems and Applications, HotMobile 2012; 2012; San Diego, CA.

103. Meek T. Anaesthetic simulators: Making the most of your purchase. Current Anaesthesia & Critical Care. 2008;19(5–6):354-60. doi: http://doi.org/10.1016/j.cacc.2008.07.010.

104. Li Y, Poon CCY, Zhang YT. Analog integrated circuits design for processing physiological signals. IEEE Rev Biomed Eng. 2010;3:93-105. doi: 10.1109/RBME.2010.2082521.

105. Światowiec A, Król W, Kuch M, Braksator W, Krysztofiak H, Dłuzniewski M, et al. Analysis of 12-lead electrocardiogram in top competitive professional athletes in the light of recent guidelines. Kardiol Pol. 2009;67(10):1095-102.

106. Rasooli M, Foomany FH, Balasundaram K, Masse S, Zamiri N, Ramadeen A, et al. Analysis of electrocardiogram pre-shock waveforms during ventricular fibrillation. Biomed Signal Process Control. 2015;21:26-33. doi: 10.1016/j.bspc.2015.05.003.

107. Yassin IM, Abidin HZ, Baharom R, Mat Saat EH, Zabidi A, editors. Analysis of genetic data in for implementation of compression algorithm in Alzheimer's disease database. 2010 IEEE EMBS Conference on Biomedical Engineering and Sciences, IECBES 2010; 2010; Kuala Lumpur.

108. Li X, Chen Z, Liang Q, Yang Y. Analysis of mental stress recognition and rating based on Hidden Markov Model. J Comput Inf Syst. 2014;10(18):7911-9. doi: 10.12733/jcis11559.

109. Vachiratamporn V, Legaspi R, Moriyama K, Fukui KI, Numao M. An analysis of player affect transitions in survival horror games. J Multimodal User Interfaces. 2015;9(1):43-54. doi: 10.1007/s12193-014-0153-4

10.1145/1836135.1836143; Fanselow, M.S., Neural organization of the defensive behavior system responsible for fear (1994) Psychon Bull Rev, 1 (4), pp. 429-438; Garner, T., Grimshaw, M., A climate of fear: considerations for designing a virtual acoustic ecology of fear. In: Proceedings of the 6th audio mostly—a conference on interaction with sound, pp 31–38 (2011) doi:10.1145/2095667.2095672; Garner, T., Grimshaw, M., Abdel Nabi, D., A preliminary experiment to assess the fear value of preselected sound parameters in a survival horror game. In: Proceedings of the 5th audio mostly—a conference on interaction with sound (2010) doi:10.1145/1859799.1859809; Giakoumis, D., Tzovaras, D., Moustakas, K., Hassapis, G., Automatic recognition of boredom in video games using novel biosignal moment-based features (2011) IEEE Trans Affect Comput, 2 (3), pp. 119-133; Gilleade, K.M., Dix, A., Allanson, J., Affective videogames and modes of affective gaming: assist me, challenge me, emote me. In: Proceedings of digital games research association (DiGRA) 2005 conference (2005) pp 547–554; Hall, M., Frank, E., Holmes, G., Pfahringer, B., Reutemann, P., Witten, I.H., The weka data mining software: an update (2009) SIGKDD Explor Newsl, 11 (1), pp. 10-18; Higuchi, T., Approach to an irregular time series on the basis of the fractal theory (1988) Phys D, 31 (2), pp. 277-283; Hudlicka, E., Affective game engines: motivation and requirements. In: Proceedings of the 4th international conference on foundations of digital games, pp 299–306 (2009) doi:10.1145/1536513.1536565; Jennett, C., Cox, A.L., Cairns, P., Dhoparee, S., Epps, A., Tijs, T., Walton, A., Measuring and defining the experience of immersion in games (2008) Int J Hum Comput Stud, 66 (9), pp. 641-661; Khanna, P., Sasikumar, M., Recognising emotions from keyboard stroke pattern (2010) Int J Comput Appl, 11 (9), pp. 1-5; Krzywinska, T., Hands-on horror (2002) Screenplay: cinema/videogames/interfaces, pp. 206-223. , Wallflower Press, London:; Landwehr, N., Hall, M., Frank, E., Logistic model trees (2005) Mach Learn, 59 (1-2), pp. 161-205; Lane, J.S., The effect of performance experience on vocal music major’s perception of musical tension (2012) J Res Music Perform. ISSN, pp. 1722-2326; Laurans, G., Desmet, P.M., Hekkert, P., Assessing emotion in human–product interaction: an overview of available methods and a new approach (2012) Int J Prod Dev, 16 (3-4), pp. 225-242; Mandryk, R.L., Atkins, M.S., A fuzzy physiological approach for continuously modeling emotion during interaction with play technologies (2007) Int J Hum Comput Stud, 65 (4), pp. 329-347; Martinez, H.P., Garbarino, M., Yannakakis, G.N., Generic physiological features as predictors of player experience (2011) Proceedings of the 4th international conference on affective computing and intelligent interaction, part, pp. 267-276; Metallinou, A., Narayanan, S., Annotation and processing of continuous emotional attributes: challenges and opportunities. In: Proceedings of the 10th IEEE international conference and workshops on automatic face and gesture recognition (2013) doi:10.1109/FG.2013.6553804; Nacke, L., Lindley, C.A., Flow and immersion in first-person shooters: measuring the player’s gameplay experience. In: Proceedings of the 2008 conference on future play, pp 81–88 (2008) doi:10.1145/1496984.1496998; Nacke, L.E., Kalyn, M., Lough, C., Mandryk, R.L., Biofeedback game design: using direct and indirect physiological control to enhance game interaction. In: Proceedings of the SIGCHI conference on human factors in computing systems, pp 103–112 (2011) doi:10.1145/1978942.1978958; Öhman, A., Anxiety (2007) Encyclopedia of stress, pp. 236-239. , Academic Press, New York:; http://www.ign.com/articles/2012/07/07/slender-is-pure-horror, Onyett C (2012) Slender is pure horror. Accessed 22 Jan 2014Parker, J.R., Heerema, J., Audio interaction in computer mediated games (2008) Int J Comput Games Technol, 2008, p. 178923; Perron, B., Sign of a threat: the effects of warning systems in survival horror games. In: Proceedings of the 4th international conference on computational semiotics (2004) Split; Prieto-Pablos, J.A., The paradox of suspense (1998) Poetics, 26 (2), pp. 99-113; Quinlan, J.R., (1993) C4.5: programs for machine learning, , Morgan Kaufmann, San Francisco:; Roux-Girard, G., Grimshaw, M., Listening to fear: a study of sound in horror computer games (2011) Game sound technology and player interaction, pp. 192-212. , IGI Global, Hershey:; Schröder, M., Cowie, R., Douglas-Cowie, E., Savvidou, S., McMahon, E., Sawey, M., ’FEELTRACE’: an instrument for recording perceived emotion in real time (2000) Proceedings of the ISCA workshop on speech and emotion, pp. 19-24. , Textflow, Belfast:; Sourina, O., Liu, Y., Nguyen, M.K., Real-time EEG-based emotion recognition for music therapy (2012) J Multimodal User Interfaces, 5 (1-2), pp. 27-35; Heart rate variability: standards of measurement, physiological interpretation, and clinical use (1996) Circulation, 93 (5), pp. 1043-1065; Toprac, P., Abdel-Meguid, A., Causing fear, suspense, and anxiety using sound design in computer games (2011) Game sound technology and player interaction, pp. 176-191. , Grimshaw M, (ed), IGI Global, Hershey:; Truong, K.P., Neerincx, M.A., van Leeuwen, D.A., Assessing agreement of observer- and self-annotations in spontaneous multimodal emotion data. In: Proceedings of the 9th Annual Conference of the International Speech Communication Association (2008) pp 318–321; Tsui, W.H., Lee, P., Hsiao, T.C., The effect of emotion on keystroke: an experimental study using facial feedback hypothesis. In: Proceedings of the 35th annual international conference of the IEEE engineering in medicine and biology society, pp 2870–2873 (2013) doi:10.1109/EMBC.2013.6610139; Wang, Q., Sourina, O., Nguyen, M.K., Fractal dimension based neurofeedback in serious games (2011) Vis Comput, 27 (4), pp. 299-309; Weber, R., Behr, K.M., Tamborini, R., Ritterfeld, U., Mathiak, K., What do we really know about first-person-shooter games? An event-related, high-resolution content analysis (2009) J Comput Mediat Commun, 14 (4), pp. 1016-1037; Witten, I.H., Frank, E., Hall, M.A., (2011) Data mining: practical machine learning tools and techniques, , Morgan Kaufmann, Amsterdam:; Yannakakis, G.N., Hallam, J., Entertainment modeling through physiology in physical play (2008) Int J Hum Comput Stud, 66 (10), pp. 741-755.

110. Gevins AS. Analysis of the Electromagnetic Signals of the Human Brain: Milestones, Obstacles, and Goals. IEEE TRANS BIOMED ENG. 1984;BME-31(12):833-50. doi: 10.1109/TBME.1984.325246.

111. Cong F, Kalyakin I, Chang Z, Ristaniemi T. Analysis on subtracting projection of extracted independent components from EEG recordings. Biomed Tech (Berl). 2011;56(4):223-34. doi: 10.1515/BMT.2011.102.

112. Gomes MM, Higgins AL, Butler R, Farzaneh JR. Anatomy of a Staged Orientation Process. Journal of Emergency Nursing. 2009;35(6):575-9. doi: http://doi.org/10.1016/j.jen.2009.09.014.

113. Kraidin J, Ginsberg SH, Solina A. Anesthesia apps: Overview of current technology and intelligent search techniques. J Cardiothorac Vasc Anesth. 2012;26(2):322-6. doi: 10.1053/j.jvca.2011.11.010.

114. Wetchler BV. Anesthesia for outpatient surgery. AORN Journal. 1981;34(2):282-96. doi: http://doi.org/10.1016/S0001-2092(07)62243-1.

115. Devi G, Sarma KK, Datta P, Mahanta AK. ANN based multi classifier system for prediction of high energy shower primary energy and core location. World Acad Sci Eng Technol. 2009;39:890-9.

116. Bursa M, Huptych M, Lhotska L, editors. Ant colony inspired metaheuristics in biological signal processing - Hybrid ant colony and evolutionary approach. BIOSIGNALS 2008 - 1st International Conference on Bio-inspired Systems and Signal Processing; 2008; Funchal, Madeira.

117. Rani P, Sarkar N, Smith CA, Kirby LD. Anxiety detecting robotic system - Towards implicit human-robot collaboration. Robotica. 2004;22(1):85-95. doi: 10.1017/S0263574703005319.

118. AORN Proceedings. AORN Journal. 1967;5(1):82-91. doi: http://doi.org/10.1016/S0001-2092(08)71360-7.

119. AORN proceedings. AORN Journal. 1972;16(1):129-36. doi: http://doi.org/10.1016/S0001-2092(07)61974-7.

120. APhA2005 Abstracts of Contributed Papers. Journal of the American Pharmacists Association. 2005;45(2):219-94. doi: http://doi.org/10.1331/1544345053623500.

121. APhA2012 abstracts of contributed papers. Journal of the American Pharmacists Association. 2012;52(2):200-84. doi: http://doi.org/10.1331/JAPhA.2012.12510.

122. APhA2015 abstracts of contributed papers. Journal of the American Pharmacists Association. 2015;55(2):e113-e263. doi: http://doi.org/10.1331/JAPhA.2015.15515.

123. Shi C, Nourani M, Gupta G, Tamil L, editors. Apnea MedAssist II: A smart phone based system for sleep apnea assessment. 2013 IEEE International Conference on Bioinformatics and Biomedicine, IEEE BIBM 2013; 2013; Shanghai.

124. Shi H, Liu J, editors. Application for fault diagnosis of loopers based on evolutionary KPCA-LSSVM. 2010 8th World Congress on Intelligent Control and Automation, WCICA 2010; 2010; Jinan.

125. Liu L, Ge Y, Liu M, Cao H, Liu Y, Zhang G, et al., editors. The application of an apparatus of motion measurement and analysis in freestyle aerials. 2010 2nd WRI Global Congress on Intelligent Systems, GCIS 2010; 2010; Wuhan.

126. Barbosa D, Santos CP, Martins M. The Application of Cycling and Cycling Combined with Feedback in the Rehabilitation of Stroke Patients: A Review. Journal of Stroke and Cerebrovascular Diseases. 2015;24(2):253-73. doi: http://doi.org/10.1016/j.jstrokecerebrovasdis.2014.09.006.

127. Chua KC, Chandran V, Acharya UR, Lim CM. Application of higher order spectra to identify epileptic EEG. J Med Syst. 2011;35(6):1563-71. doi: 10.1007/s10916-010-9433-z.

128. Martis RJ, Acharya UR, Prasad H, Chua CK, Lim CM, Suri JS. Application of higher order statistics for atrial arrhythmia classification. Biomed Signal Process Control. 2013;8(6):888-900. doi: 10.1016/j.bspc.2013.08.008

10.1037/a0016973.supp, (last accessed 8.08.13); Bishop, C.M., (1995) Neural Networks for Pattern Recognition, , Oxford University Press Walton Street, Oxford; Cerutti, S., Mainardi, L.T., Porta, A., Bianchi, A.M., Analysis of the dynamics of RR interval series for the detection of atrial fibrillation episodes (1997) Computers in Cardiology, 24, pp. 77-80; Slocum, J., Sahakian, A., Swiryn, S., Diagnosis of atrial fibrillation from surface electrocardiograms based on computer-detected atrial activity (1992) Journal of Electrocardiology, 25, pp. 1-8; Yang, W., Yi-Sheng, Z., Thakor, N.V., Yu-Hong, X., A short-time multifractal approach for arrhythmia detection based on fuzzy neural network (2001) IEEE Transactions on Biomedical Engineering, 48, pp. 989-995; Sarkar, S., Ritscher, D., Mehra, R., A detector for a chronic implantable atrial tachyarrhythmia (2008) IEEE Transactions on Monitor, Biomedical Engineering, 55, pp. 1219-1224; Chao, H., Shuming, Y., Hang, C., Dingli, L., Fangtian, H., Yuewen, T., A novel method for detection of the transition between atrial fibrillation and sinus rhythm (2011) IEEE Transactions on Biomedical Engineering, 58, pp. 1113-1119; Fahim, S., Khalil, I., Diagnosis of cardiovascular abnormalities from compressed ECG: A data mining-based approach (2011) IEEE Transactions on Information Technology in Biomedicine, 15, pp. 33-39; Logan, B., Healey, J., Robust detection of atrial fibrillation for a long term telemonitoring system (2005) Computers in Cardiology, 2005, pp. 619-622; Tateno, K., Glass, L., Automatic detection of atrial fibrillation using the coefficient of variation and density histograms of RR and ΔrR intervals (2001) Medical and Biological Engineering and Computing, 39, pp. 664-671; Jovic, A., Bogunovic, N., Evaluating and comparing performance of feature combinations of heart rate variability measures for cardiac rhythm classification (2012) Biomedical Signal Processing and Control, 7 (3), pp. 245-255.

129. Acharya UR, Sudarshan VK, Koh JEW, Martis RJ, Tan JH, Oh SL, et al. Application of higher-order spectra for the characterization of Coronary artery disease using electrocardiogram signals. Biomed Signal Process Control. 2017;31:31-43. doi: 10.1016/j.bspc.2016.07.003.

130. Jiang M, Zhu L, Wang Y, Xia L, Shou G, Liu F, et al. Application of kernel principal component analysis and support vector regression for reconstruction of cardiac transmembrane potentials. Physics in Medicine and Biology. 2011;56(6):1727-42. doi: 10.1088/0031-9155/56/6/013.

131. Mohd Zahid MS, Abdullah AH, Supriyanto E, editors. Application of Mobile Cloud Computing in Care pathways. 2014 IEEE Canada International Humanitarian Technology Conference, IHTC 2014; 2014: Institute of Electrical and Electronics Engineers Inc.

132. Wai KL, Bing NL, Ming CD, Bin BF, editors. An application of morphological feature extraction and support vector machines in computerized ECG interpretation. 2007 6th Mexican International Conference on Artificial Intelligence, Special Session, MICAI 2007; 2008; Aguascalientes.

133. Martis RJ, Acharya UR, Mandana KM, Ray AK, Chakraborty C. Application of principal component analysis to ECG signals for automated diagnosis of cardiac health. Expert Sys Appl. 2012;39(14):11792-800. doi: 10.1016/j.eswa.2012.04.072.

134. Alavi-Moghaddam M, Forouzanfar R, Alamdari S, Shahrami A, Kariman H, Amini A, et al. Application of Queuing Analytic Theory to Decrease Waiting Times in Emergency Department: Does it Make Sense? Arch Trauma Res. 2012;1(3):101-7. Epub 2012/10/01. doi: 10.5812/atr.7177. PubMed PMID: 24396756; PubMed Central PMCID: PMCPMC3876544.

135. Du BX, Liu HJ. The application of recurrence plot in DC tracking test of gamma-ray irradiated polycarbonate. IEEE Trans Dielectr Electr Insul. 2009;16(1):17-23. doi: 10.1109/TDEI.2009.4784547.

136. Wasan PS, Uttamchandani M, Moochhala S, Yap VB, Yap PH. Application of statistics and machine learning for risk stratification of heritable cardiac arrhythmias. Expert Sys Appl. 2013;40(7):2476-86. doi: 10.1016/j.eswa.2012.10.054

10.1109/SSDM.2004.1311241 In Presented at the IEEE; Anderson, C.A., Pettersson, F.H., Clarke, G.M., Cardon, L.R., Morris, A.P., Zondervan, K.T., Data quality control in genetic case-control association studies (2010) Nature Protocols, 5 (9), pp. 1564-1573. , 10.1038/nprot.2010.116; Arking, D.E., Pfeufer, A., Post, W., Kao, W.H.L., Newton-Cheh, C., Ikeda, M., A common genetic variant in the NOS1 regulator NOS1AP modulates cardiac repolarization (2006) Nature Genetics, 38 (6), pp. 644-651. , 10.1038/ng1790; Ashby, D., Smith, A.F.M., Evidence-based medicine as Bayesian decision-making (2000) Statistics in Medicine, 19 (23), pp. 3291-3305; Babaee Bigi, M.A., Aslani, A., Aslani, A., Significance of cardiac autonomic neuropathy in risk stratification of Brugada syndrome (2008) Europace, 10 (7), pp. 821-824. , 10.1093/europace/eum272; Bailey, J.J., Berson, A.S., Handelsman, H., Hodges, M., Utility of current risk stratification tests for predicting major arrhythmic events after myocardial infarction (2001) Journal of the American College of Cardiology, 38 (7), pp. 1902-1911; Balding, D., A tutorial on statistical methods for population association studies (2006) Nature Reviews Genetics, , (Abstract); Becker, M.L., Visser, L.E., Newton-Cheh, C., Hofman, A., Uitterlinden, A.G., Witteman, J.C.M., A common NOS1AP genetic polymorphism is associated with increased cardiovascular mortality in users of dihydropyridine calcium channel blockers (2009) British Journal of Clinical Pharmacology, 67 (1), pp. 61-67. , 10.1111/j.1365-2125.2008.03325.x; Behr, E.R., Dalageorgou, C., Christiansen, M., Syrris, P., Hughes, S., Tome Esteban, M.T., Sudden arrhythmic death syndrome: Familial evaluation identifies inheritable heart disease in the majority of families (2008) European Heart Journal, 29 (13), pp. 1670-1680. , 10.1093/eurheartj/ehn219; Benito, B., Brugada, R., Brugada, J., Brugada, P., Brugada syndrome (2008) Progress in Cardiovascular Diseases, 51 (1), pp. 1-22; Benito, B., Sarkozy, A., Mont, L., Henkens, S., Berruezo, A., Tamborero, D., Gender differences in clinical manifestations of Brugada syndrome (2008) Journal of the American College of Cardiology, 52 (19), pp. 1567-1573. , 10.1016/j.jacc.2008.07.052; Berthold, M.R., Cebron, N., Dill, F., Gabriel, T.R., Kötter, T., Meinl, T., KNIME: The Konstanz information miner (2008) Data Analysis, Machine Learning and Applications, pp. 319-326. , http://dx.doi.org/10.1007/978-3-540-78246-9_38, C. Preisach, H. Burkhardt, L. Schmidt-Thieme, & R. Decker (Eds.) Berlin, Heidelberg: Springer Berlin Heidelberg; Bigi, R., Gregori, D., Cortigiani, L., Desideri, A., Artificial neural networks and robust Bayesian classifiers for risk stratification following uncomplicated myocardial infarction (2005) International Journal of Cardiology, 101 (3), pp. 481-487; Breiman, L., Bagging predictors (1996) Machine Learning, 24 (2), pp. 123-140; Breiman, L., Random forests (2001) Machine Learning, 45 (1), pp. 5-32; Breiman, L., Friedman, J., Stone, C.J., Olshen, R.A., (1984) Classification and Regression Trees, , (1st ed.). Chapman and Hall/CRC; Brugada, J., Determinants of sudden cardiac death in individuals with the electrocardiographic pattern of Brugada syndrome and no previous cardiac arrest (2003) Circulation, 108 (25), pp. 3092-3096. , 10.1161/01.CIR.0000104568.13957.4F; Brugada, R., Campuzano, O., (2009) Clinical Approach to Sudden Cardiac Death Syndromes, pp. 121-129. , R. Brugada, Springer London London 10.1007/978-1-84882-927-5-9; Calabrese, R., Capriotti, E., Fariselli, P., Martelli, P.L., Casadio, R., Functional annotations improve the predictive score of human disease-related mutations in proteins (2009) Human Mutation, 30 (8), pp. 1237-1244. , 10.1002/humu.21047; Chen, J.-Z., Xie, X.-D., Wang, X.-X., Tao, M., Shang, Y.-P., Guo, X.-G., Single nucleotide polymorphisms of the SCN5A gene in Han Chinese and their relation with Brugada syndrome (2004) Chinese Medical Journal, 117 (5), pp. 652-656; Chinushi, M., Komura, S., Izumi, D., Furushima, H., Tanabe, Y., Washizuka, T., Incidence and initial characteristics of pilsicainide-induced ventricular arrhythmias in patients with Brugada syndrome (2007) Pacing and Clinical Electrophysiology, 30 (5), pp. 662-671; Çinar, M., Engin, M., Engin, E.Z., Ziya Ateşçi, Y., Early prostate cancer diagnosis by using artificial neural networks and support vector machines (2009) Expert Systems with Applications, 36 (3), pp. 6357-6361. , 10.1016/j.eswa.2008.08.010; Clayton, D.G., Prediction and interaction in complex disease genetics: Experience in type 1 diabetes (2009) PLoS Genetics, 5 (7), p. 1000540. , M.I. McCarthy, 10.1371/journal.pgen.1000540.t001; Cordell, H.J., Epistasis: What it means, what it does not mean, and statistical methods to detect it in humans (2002) Human Molecular Genetics, 11 (20), p. 2463; Cordell, H.J., Estimation and testing of gene-environment interactions in family-based association studies (2009) Genomics, 93 (1), pp. 5-9. , 10.1016/j.ygeno.2008.05.002; Cosgun, E., Limdi, N.A., Duarte, C.W., High-dimensional pharmacogenetic prediction of a continuous trait using machine learning techniques with application to warfarin dose prediction in African Americans (2011) Bioinformatics, 27 (10), pp. 1384-1389. , 10.1093/bioinformatics/btr159; Delise, P., Allocca, G., Marras, E., Giustetto, C., Gaita, F., Sciarra, L., Risk stratification in individuals with the Brugada type 1 ECG pattern without previous cardiac arrest: Usefulness of a combined clinical and electrophysiologic approach (2011) European Heart Journal, 32 (2), pp. 169-176. , 10.1093/eurheartj/ehq381; Demšar, J., Zupan, B., Leban, G., Curk, T., Orange: From experimental machine learning to interactive data mining (2004) Knowledge Discovery in Databases: PKDD, pp. 537-539; Dietterich, T., (2000) Multiple Classifier Systems; Doi, A., Takagi, M., Maeda, K., Tatsumi, H., Shimeno, K., Yoshiyama, M., Conduction delay in right ventricle as a marker for identifying high-risk patients with Brugada syndrome (2009) Journal of Cardiovascular Electrophysiology, 21 (6), pp. 688-696. , 10.1111/j.1540-8167.2009.01677.x; Eijgelsheim, M., Aarnoudse, A.L.H.J., Rivadeneira, F., Kors, J.A., Witteman, J.C.M., Hofman, A., Identification of a common variant at the NOS1AP locus strongly associated to QT-interval duration (2009) Human Molecular Genetics, 18 (2), pp. 347-357. , 10.1093/hmg/ddn341; Eijgelsheim, M., Newton-Cheh, C., Aarnoudse, A.L.H.J., Van Noord, C., Witteman, J.C.M., Hofman, A., Genetic variation in NOS1AP is associated with sudden cardiac death: Evidence from the Rotterdam study (2009) Human Molecular Genetics, 18 (21), pp. 4213-4218. , 10.1093/hmg/ddp356; Elliott, P.M., Poloniecki, J., Dickie, S., Sharma, S., Monserrat, L., Varnava, A., Sudden death in hypertrophic cardiomyopathy: Identification of high risk patients (2000) Journal of the American College of Cardiology, 36 (7), pp. 2212-2218. , 10.1016/S0735-1097(00)01003-2; Exarchos, T.P., Tzallas, A.T., Baga, D., Chaloglou, D., Fotiadis, D.I., Tsouli, S., Using partial decision trees to predict Parkinson"s symptoms: A new approach for diagnosis and therapy in patients suffering from Parkinson"s disease (2012) Computers in Biology and Medicine, 42 (2), pp. 195-204. , 10.1016/j.compbiomed.2011.11.008; Garcia-Alvarez, A., Arzamendi, D., Loma-Osorio, P., Kiamco, R., Masotti, M., Sionis, A., Early risk stratification of patients with cardiogenic shock complicating acute myocardial infarction who undergo percutaneous coronary intervention (2009) The American Journal of Cardiology, 103 (8), pp. 1073-1077. , 10.1016/j.amjcard.2008.12.033; García-Magariños, M., López-De-Ullibarri, I., Cao, R., Salas, A., Evaluating the ability of tree-based methods and logistic regression for the detection of SNP-SNP interaction (2009) Annals of Human Genetics, 73 (3), pp. 360-369. , 10.1111/j.1469-1809.2009.00511.x; Gashler, M., Giraud-Carrier, C., Martinez, T., Decision tree ensemble: Small heterogeneous is better than large homogeneous (2008) Seventh International Conference on Machine Learning and Applications, ICMLA'08, pp. 900-905; Gehi, A.K., Duong, T.D., Metz, L.D., Gomes, J.A., Mehta, D., Risk stratification of individuals with the Brugada electrocardiogram: A meta-analysis (2006) Journal of Cardiovascular Electrophysiology, 17 (6), pp. 577-583. , 10.1111/j.1540-8167.2006.00455.x; Gilliam, T., Tanzi, R., Haines, J., Bonner, T., Localization of the Huntington's disease gene to a small segment of chromosome 4 flanked by D4S10 and the telomere (1987) Cell; Giustetto, C., Drago, S., Demarchi, P.G., Dalmasso, P., Bianchi, F., Masi, A.S., Risk stratification of the patients with Brugada type electrocardiogram: A community-based prospective study (2008) Europace, 11 (4), pp. 507-513. , 10.1093/europace/eup006; Goecks, J., Nekrutenko, A., Taylor, J., Galaxy: A comprehensive approach for supporting accessible, reproducible, and transparent computational research in the life sciences (2010) Genome Biology; Goldberger, J.J., Buxton, A.E., Cain, M., Costantini, O., Exner, D.V., Knight, B.P., Risk stratification for arrhythmic sudden cardiac death: Identifying the roadblocks (2011) Circulation, 123 (21), pp. 2423-2430. , 10.1161/CIRCULATIONAHA.110.959734; Goldenberg, I., Horr, S., Moss, A.J., Lopes, C.M., Barsheshet, A., McNitt, S., Risk for life-threatening cardiac events in patients with genotype-confirmed long-QT syndrome and normal-range corrected QT intervals (2011) Journal of the American College of Cardiology, 57 (1), pp. 51-59. , 10.1016/j.jacc.2010.07.038; Goldenberg, I., Moss, A.J., Peterson, D.R., McNitt, S., Zareba, W., Andrews, M.L., Risk factors for aborted cardiac arrest and sudden cardiac death in children with the congenital long-QT syndrome (2008) Circulation, 117 (17), pp. 2184-2191. , 10.1161/CIRCULATIONAHA.107.701243; Green, C.L., Kligfield, P., George, S., Gussak, I., Vajdic, B., Sager, P., Detection of QT prolongation using a novel electrocardiographic analysis algorithm applying intelligent automation: Prospective blinded evaluation using the cardiac safety research consortium electrocardiographic database (2012) American Heart Journal, 163 (3), pp. 365-371. , 10.1016/j.ahj.2011.11.009; Han, J., Kamber, M., Pei, J., (2011) Data Mining: Concepts and Techniques. Morgan Kaufmann; Haseena, H.H., Mathew, A.T., Paul, J.K., Fuzzy clustered probabilistic and multi layered feed forward neural networks for electrocardiogram arrhythmia classification (2009) Journal of Medical Systems, 35 (2), pp. 179-188. , 10.1007/s10916-009-9355-9; Hirschhorn, J., Genome-wide association studies for common diseases and complex traits (2005) Nature Reviews Genetics, , (Abstract); Hobbs, J.B., Peterson, D.R., Moss, A.J., McNitt, S., Zareba, W., Goldenberg, I., Risk of aborted cardiac arrest or sudden cardiac death during adolescence in the long-QT syndrome (2006) JAMA: The Journal of the American Medical Association, 296 (10), p. 1249; Holmes, G., Donkin, A., Witten, I.H., (1994) Proceedings of ANZIIS '94 - Australian New Zealand Intelligent Information Systems Conference. Presented at the ANZIIS '94 - Australian New Zealnd Intelligent Information Systems Conference, pp. 357-361. , IEEE 10.1109/ANZIIS.1994.396988; Ikeda, T., Takami, M., Sugi, K., Mizusawa, Y., Sakurada, H., Yoshino, H., Noninvasive risk stratification of subjects with a Brugada-type electrocardiogram and no history of cardiac arrest (2005) Annals of Noninvasive Electrocardiology, 10 (4), pp. 396-403; Jiang, R., Yang, H., Zhou, L., Kuo, C.C.J., Sun, F., Chen, T., Sequence-based prioritization of nonsynonymous single-nucleotide polymorphisms for the study of disease mutations (2007) The American Journal of Human Genetics, 81 (2), pp. 346-360. , 10.1086/519747; Jouven, X., Desnos, M., Guerot, C., Ducimetière, P., Predicting sudden death in the population: The Paris prospective study i (1999) Circulation, 99 (15), pp. 1978-1983; Jouven, X., Zureik, M., Desnos, M., Guerot, C., Ducimetière, P., Resting heart rate as a predictive risk factor for sudden death in middle-aged men (2001) Cardiovascular Research, 50 (2), p. 373; Kapplinger, J.D., Tester, D.J., Alders, M., Benito, B., Berthet, M., Brugada, J., An international compendium of mutations in the SCN5A-encoded cardiac sodium channel in patients referred for Brugada syndrome genetic testing (2010) Heart Rhythm: The Official Journal of the Heart Rhythm Society, 7 (1), pp. 33-46. , 10.1016/j.hrthm.2009.09.069; Kattygnarath, D., Maugenre, S., Neyroud, N., Balse, E., Ichai, C., Denjoy, I., MOG1: A new susceptibility gene for Brugada syndrome (2011) Circulation. Cardiovascular Genetics, 4 (3), pp. 261-268. , 10.1161/CIRCGENETICS.110.959130; Kerem, B., Rommens, J., Buchanan, J., Markiewicz, D., Cox, T., Chakravarti, A., Identification of the cystic fibrosis gene: Genetic analysis (1989) Science, 245 (4922), pp. 1073-1080. , 10.1126/science.2570460; Koeppel, F., Labarre, D., Zitoun, P., Quickly finding a needle in a haystack: A new automated cardiac arrhythmia detection software for preclinical studies (2012) Journal of Pharmacological and Toxicological Methods, , 10.1016/j.vascn.2012.04.008; Kohonen, T., Kohonen network (2007) Scholarpedia; Kotta, C.-M., Anastasakis, A., Gatzoulis, K., Manolis, A.S., Stefanadis, C., Novel sodium channel SCN5A mutations in Brugada syndrome patients from Greece (2010) International Journal of Cardiology, 145 (1), pp. 45-48. , 10.1016/j.ijcard.2009.03.134; Krishnan, V.G., Westhead, D.R., A comparative study of machine-learning methods to predict the effects of single nucleotide polymorphisms on protein function (2003) Bioinformatics, 19 (17), pp. 2199-2209. , 10.1093/bioinformatics/btg297; Lander, E.S., Linton, L.M., Birren, B., Nusbaum, C., Zody, M.C., Baldwin, J., Initial sequencing and analysis of the human genome (2001) Nature, 409 (6822), pp. 860-921. , 10.1038/35057062; Latcu, G.D., Meste, O., Duparc, A., Mondoly, P., Rollin, A., Delay, M., Temporal and spectral analysis of ventricular fibrillation in humans (2011) Journal of Interventional Cardiac Electrophysiology, 30 (3), pp. 199-209. , 10.1007/s10840-010-9541-1; Leite, C.R.M., Martin, D.L., Sizilio, G.R.M.A., Santos Dos, K.E.A., De Araújo, B.G., De M Valentim, R.A., (2010) Annual International Conference of the IEEE Engineering in Medicine and Biology. Presented at the 2010 32nd Annual International Conference of the IEEE Engineering in Medicine and Biology Society (EMBC 2010), pp. 1386-1389. , IEEE 10.1109/IEMBS.2010.5626728; Letsas, K.P., Weber, R., Efremidis, M., Korantzopoulos, P., Astheimer, K., Charalampous, C., Long-term prognosis of asymptomatic individuals with spontaneous or drug-induced type 1 electrocardiographic phenotype of Brugada syndrome (2011) Journal of Electrocardiology, 44 (3), pp. 346-349. , 10.1016/j.jelectrocard.2010.12.007; London, B., Michalec, M., Mehdi, H., Zhu, X., Kerchner, L., Sanyal, S., (2007) Mutation in glycerol-3-phosphate Dehydrogenase 1-like Gene (GPD1-L) Decreases Cardiac Na+ Current and Causes Inherited Arrhythmias; Maroco, J., Silva, D., Rodrigues, A., Guerreiro, M., Santana, I., De Mendonça, A., Data mining methods in the prediction of dementia: A real-data comparison of the accuracy, sensitivity and specificity of linear discriminant analysis, logistic regression, neural networks, support vector machines, classification trees and random forests (2011) BMC Research Notes, 4, p. 299. , 10.1186/1756-0500-4-299; Martis, R.J., Krishnan, M.M.R., Chakraborty, C., Pal, S., Sarkar, D., Mandana, K.M., Automated screening of arrhythmia using wavelet based machine learning techniques (2012) Journal of Medical Systems, 36 (2), pp. 677-688. , 10.1007/s10916-010-9535-7; McKinney, B.A., Reif, D.M., Ritchie, M.D., Moore, J.H., Machine learning for detecting gene-gene interactions: A review (2006) Applied Bioinformatics, 5 (2), pp. 77-88; McPherson, J., Marra, M., Hillier, L., Waterston, R., A physical map of the human genome (2001) Nature, , (Abstract); Metzker, M.L., Sequencing technologies - The next generation (2009) Nature Reviews Genetics, 11 (1), pp. 31-46. , 10.1038/nrg2626; Milhorn, H.T., (2005) Electrocardiography for the Family Physician: The Essentials, , Brown Walker Press; Milpied, P., Dubois, R., Roussel, P., Henry, C., Dreyfus, G., Arrhythmia discrimination in implantable cardioverter defibrillators using support vector machines applied to a new representation of electrograms (2011) IEEE Transactions on Biomedical Engineering, 58 (6), pp. 1797-1803. , 10.1109/TBME.2011.2117424; Nakano, Y., Shimizu, W., Ogi, H., Suenari, K., Oda, N., Makita, Y., A spontaneous type 1 electrocardiogram pattern in lead V2 is an independent predictor of ventricular fibrillation in Brugada syndrome (2010) Europace, 12 (3), pp. 410-416. , 10.1093/europace/eup446; Napolitano, C., Antzelevitch, C., (2011) Phenotypical Manifestations of Mutations in the Genes Encoding Subunits of the Cardiac Voltage-dependent L-type Calcium Channel; Napolitano, C., Bloise, R., Monteforte, N., Priori, S.G., Sudden cardiac death and genetic ion channelopathies: Long QT, Brugada, short QT, catecholaminergic polymorphic ventricular tachycardia, and idiopathic ventricular fibrillation (2012) Circulation, 125 (16), pp. 2027-2034. , 10.1161/CIRCULATIONAHA.111.055947; Nunn, L.M., Bhar-Amato, J., Lambiase, P.D., Brugada syndrome: Controversies in risk stratification and management (2010) Indian Pacing and Electrophysiology Journal, 10 (9), p. 400; Oinn, T., Addis, M., Ferris, J., Marvin, D., Senger, M., Greenwood, M., Carver, T., (2004) Taverna: A Tool for the Composition and Enactment of Bioinformatics Workflows; Pfeufer, A., Sanna, S., Arking, D.E., Ller, M.M.U., Gateva, V., Fuchsberger, C., Common variants at ten loci modulate the QT interval duration in the QTSCD study (2009) Nature Genetics, 41 (4), pp. 407-414. , 10.1038/ng.362; Pirooznia, M., Yang, J.Y., Yang, M.Q., Deng, Y., A comparative study of different machine learning methods on microarray gene expression data (2008) BMC Genomics, 9 (SUPPL. 1), p. 13. , 10.1186/1471-2164-9-S1-S13; Priori, S.G., Natural history of Brugada syndrome: Insights for risk stratification and management (2002) Circulation, 105 (11), pp. 1342-1347. , 10.1161/hc1102.105288; Priori, S., Schwartz, P., Napolitano, C., Bloise, R., Risk stratification in the long-QT syndrome (2003) The New England Journal of Medicine; Probst, V., Veltmann, C., Eckardt, L., Meregalli, P.G., Gaita, F., Tan, H.L., Long-term prognosis of patients diagnosed with Brugada syndrome: Results from the FINGER Brugada syndrome registry (2010) Circulation, 121 (5), pp. 635-643. , 10.1161/CIRCULATIONAHA.109.887026; Raju, H., Papadakis, M., Govindan, M., Bastiaenen, R., Chandra, N., O'Sullivan, A., Low prevalence of risk markers in cases of sudden death due to Brugada syndrome (2011) Journal of the American College of Cardiology, 57 (23), pp. 2340-2345. , 10.1016/j.jacc.2010.11.067; Rokach, L., Maimon, O.Z., (2008) Data Mining with Decision Trees, , World Scientific Pub Co Inc; Rumelhart, D.E., Hintont, G.E., Williams, R.J., Learning representations by back-propagating errors (1986) Nature, 323 (6088), pp. 533-536; Schramm, A., Schulte, J.H., Klein-Hitpass, L., Havers, W., Sieverts, H., Berwanger, B., Prediction of clinical outcome and biological characterization of neuroblastoma by expression profiling (2005) Oncogene, 24 (53), pp. 7902-7912. , 10.1038/sj.onc.1208936; Schulte, J.H., Schowe, B., Mestdagh, P., Kaderali, L., Kalaghatgi, P., Schlierf, S., Accurate prediction of neuroblastoma outcome based on miRNA expression profiles (2010) International Journal of Cancer. Journal International du Cancer, 127 (10), pp. 2374-2385. , 10.1002/ijc.25436; Schwarz, D.F., Szymczak, S., Ziegler, A., König, I.R., Picking single-nucleotide polymorphisms in forests (2007) BMC Proceedings, 1 (SUPPL. 1), p. 59; Segal, M., (2004) Machine Learning Benchmarks and Random Forest Regression; Sekkal, M., Chikh, M.A., Settouti, N., Evolving neural networks using a genetic algorithm for heartbeat classification (2011) Journal of Medical Engineering and Technology, 35 (5), pp. 215-223. , 10.3109/03091902.2011.574778; Sepulveda-Sanchis, J., Camps-Valls, G., Soria-Olivas, E., Salcedo-Sanz, S., Bousono-Calzon, C., Sanz-Romero, G., (2002) Computers in Cardiology. Presented at the Computers in Cardiology, 29. , IEEE doi:0.1109/CIC.2002.1166797; Siemiatycki, J., Thomas, D.C., Biological models and statistical interactions: An example from multistage carcinogenesis (1981) International Journal of Epidemiology, 10 (4), pp. 383-387; Straus, S.M.J.M., Bleumink, G.S., Dieleman, J.P., Van Der Lei, J., Jong G W, T., Kingma, J.H., Antipsychotics and the risk of sudden cardiac death (2004) Archives of Internal Medicine, 164 (12), p. 1293. , doi:10.1001/archinte.164.12.1293; Sun, Y.V., Cai, Z., Desai, K., Lawrence, R., Leff, R., Jawaid, A., Classification of rheumatoid arthritis status with candidate gene and genome-wide single-nucleotide polymorphisms using random forests (2007) BMC Proceedings, 1 (1), p. 62; Sun, Y.V., Bielak, L.F., Peyser, P.A., Turner, S.T., Sheedy, I.I.P.F., Application of machine learning algorithms to predict coronary artery calcification with a sibship-based design (2008) Genetic Epidemiology, 32 (4), pp. 350-360. , 10.1002/gepi.20309; Sun, Y., Bielak, L., Peyser, P., Application of machine learning algorithms to predict coronary artery calcification with a sibship-based design-Sun-2008-Genetic Epidemiology-Wiley Online Library (2008) Genetic....; Sunay, A., Cunedioǧlu, U., Feasibility of probabilistic neural networks, Kohonen self-organizing maps and fuzzy clustering for source localization of ventricular focal arrhythmias from intravenous catheter measurements-Sunay-2009-Expert Systems-Wiley Online Library (2009) Expert Systems; Syed, T.F., Guttag, J.V., (2011) Unsupervised Similarity-based Risk Stratification for Cardiovascular Events Using Long-term Time-series Data; Szymczak, S., Biernacka, J.M., Cordell, H.J., González-Recio, O., König, I.R., Zhang, H., Sun, Y.V., Machine learning in genome-wide association studies (2009) Genetic Epidemiology, 33 (S1), pp. S51-S57. , doi:10.1002/gepi.20473 J. W. MacCluer, L. A. Cupples, & L. Almasy (Eds.); Takagi, M., Yokoyama, Y., Aonuma, K., Aihara, N., Hiraoka, M., Clinical characteristics and risk stratification in symptomatic and asymptomatic patients with Brugada syndrome: Multicenter study in Japan (2007) Journal of Cardiovascular Electrophysiology, 18 (12), pp. 1244-1251. , for the Japan Idiopathic Ventricular Fibrillation Study (J-IVFS) Investigators 10.1111/j.1540-8167.2007.00971.x; Tatsumi, H., Takagi, M., Nakagawa, E., Yamashita, H., Yoshiyama, M., Risk stratification in patients with Brugada syndrome: Analysis of daily fluctuations in 12-lead electrocardiogram (ECG) and signal-averaged electrocardiogram (SAECG) (2006) Journal of Cardiovascular Electrophysiology, 17 (7), pp. 705-711. , 10.1111/j.1540-8167.2006.00508.x; Tuana, G., Volpato, V., Ricciardi-Castagnoli, P., Zolezzi, F., Stella, F., Foti, M., Classification of dendritic cell phenotypes from gene expression data (2011) BMC Immunology, 12, p. 50. , 10.1186/1471-2172-12-50; Tufféry, S., (2011) Data Mining and Statistics for Decision Making, , Wiley; Ture, M., Tokatli, F., Using Kaplan-Meier analysis together with decision tree methods (C&RT, CHAID, QUEST, C4.5 and ID3) in determining recurrence-free survival of breast cancer patients (2009) Expert Systems with Applications, 23. , 10.1016/j.eswa.2007.12.002; Vapnik, V., (1995) Machine Learning, , Springer 20(3); Veltmann, C., Schimpf, R., Borggrefe, M., Wolpert, C., Risk stratification in electrical cardiomyopathies (2009) Herz, 34 (7), pp. 518-527. , 10.1007/s00059-009-3288-4; Venter, J.C., Adams, M.D., Myers, E.W., Li, P.W., Mural, R.J., Sutton, G.G., The sequence of the human genome (2001) Science Signaling, 291 (5507), p. 1304. , 10.1126/science.1058040; Wan, X., Yang, C., Yang, Q., Xue, H., Tang, N.L.S., Yu, W., MegaSNPHunter: A learning approach to detect disease predisposition SNPs and high level interactions in genome wide association study (2009) BMC Bioinformatics, 10, p. 13. , 10.1186/1471-2105-10-13; Watanabe, H., Darbar, D., Kaiser, D.W., Jiramongkolchai, K., Chopra, S., Donahue, B.S., Kannankeril, P.J., Mutations in sodium channel β1- and β2-subunits associated with atrial fibrillation (2009) Clinical Perspective; Yang, P., Hwa Yang, Y., Zhou, B.B., Zomaya, A.Y., (2010) A Review of Ensemble Methods in Bioinformatics; Yue, P., Moult, J., Identification and analysis of deleterious human SNPs (2006) Journal of Molecular Biology, 356 (5), pp. 1263-1274. , 10.1016/j.jmb.2005.12.025; Zadeh, A.E., Khazaee, A., Ranaee, V., Classification of the electrocardiogram signals using supervised classifiers and efficient features (2010) Computer Methods and Programs in Biomedicine, 99 (2), pp. 179-194. , 10.1016/j.cmpb.2010.04.013; Zondervan, K., Designing candidate gene and genome-wide case-control association studies (2007) Nature Protocols, , (Abstract); Zweig, M.H., Campbell, G., Receiver-operating characteristic (ROC) plots: A fundamental evaluation tool in clinical medicine (1993) Clinical Chemistry, 39 (4), pp. 561-577.

137. Kasamatsu T, Hashimoto J, Iyatomi H, Nakahara T, Bai J, Kitamura N, et al. Application of support vector machine classifiers to preoperative risk stratification with myocardial perfusion scintigraphy. Circ J. 2008;72(11):1829-35. doi: 10.1253/circj.CJ-08-0236.

138. Mehta SS, Lingayat NS. Application of support vector machine for the detection of P- and T-waves in 12-lead electrocardiogram. COMPUT METHODS PROGRAMS BIOMED. 2009;93(1):46-60. doi: 10.1016/j.cmpb.2008.07.014.

139. Hidalgo-Muñoz AR, López MM, Santos IM, Pereira AT, Vázquez-Marrufo M, Galvao-Carmona A, et al. Application of SVM-RFE on EEG signals for detecting the most relevant scalp regions linked to affective valence processing. Expert Sys Appl. 2013;40(6):2102-8. doi: 10.1016/j.eswa.2012.10.013.

140. Sudarshan VK, Mookiah MRK, Acharya UR, Chandran V, Molinari F, Fujita H, et al. Application of wavelet techniques for cancer diagnosis using ultrasound images: A Review. Comput Biol Med. 2016;69:97-111. doi: 10.1016/j.compbiomed.2015.12.006.

141. MacIntyre J. Applications of neural computing in the twenty-first century and 21 years of Neural Computing & Applications. Neural Comput Appl. 2013;23(3-4):657-65. doi: 10.1007/s00521-013-1471-2

10. 1007/978-94-009-0431-6_44; Caudill, M., Butler, C.T., (1992) Naturally Intelligent Systems, , Cambridge, MA: MIT Press; Hebb, D.O., (1949) The Organization of Behavior, , New York: Wiley; Carpenter, G., Grossberg, S., The ART of adaptive pattern recognition by a self-organizing neural network (1988) Computer, 21 (3), pp. 77-88; Hinton, G.E., How neural networks learn from experience (1992) Sci Am, 267 (3), pp. 144-151; Werbos, P.J., (1974) Beyond Regression: New Tools For Prediction and Analysis In the Behavioral Sciences, , PhD Thesis, Harvard University; Bounds, D., Howard, J., Satchwell, C., Editorials (1993) Neural Comput Appl, 1 (1), pp. 1-3; Minsky, M., Papert, S., (1969) Perceptron: An Introduction to Computational Geometry, p. 88. , 19th edn., Cambridge: The MIT Press; Frank, R., The perceptron: a probabilistic model for information storage and organization in the brain (1958) Psychol Rev, 65 (6), p. 386; Jain, A.K., Mao, J., Mohiuddin, K.M., Artificial neural networks: a tutorial (1996) Computer, 29 (3), pp. 31-44; Vasconcelos, G., Fairhurst, M.C., Bisset, D.L., Efficient detection of spurious inputs for improving the robustness of MLP networks in practical applications (1995) Neural Comput Appl, 3 (4), pp. 202-212; Zhou, P., Austin, J., Learning criteria for training neural network classifiers (1998) Neural Comput Appl, 7, pp. 334-342; Alejo, R., Garcia, V., Sotoca, J.M., Mollineda, R.A., Sánchez, J.S., Improving the Classification Accuracy of RBF and MLP Neural Networks Trained With Imbalanced Samples (2006) Intelligent Data Engineering and Automated Learning - IDEAL 2006. Lecture Notes In Computer Science, 4224, pp. 464-471. , In: Corchado E Et Al (eds), Springer, Heidelberg, doi: 10. 1007/11875581_56; Benoudjit, N., Verleysen, M., On the Kernel Widths in radial-basis function networks (2003) Neural Process Lett, 18 (2), pp. 139-154; Wunsch, I.I.D.C., Hasselmo, M.E., Venayagamoorthy, G.K., Wang, D., (2003) Advances In Neural Network Research: Based On the Proceedings of IJCNN 2003, , 1st edn. ISBN: 9780080443201; Bullinaria, J., Using evolution to improve neural network learning: pitfalls and solutions (2007) Neural Comput Appl, 16 (3), pp. 209-226; McCulloch, W.S., Pitts, W., A logical calculus of the ideas immanent in nervous activity (1943) The Bulletin of Mathematical Biophysics, 5 (4), pp. 115-133; Belew, R.K., McInerney, J., Schraudolph, N.N., (1990) Evolving Networks: Using the Genetic Algorithm With Connectionist Learning, , CSE Technical Report #CS90-174, June, 1990; Whitley, D., A genetic algorithm tutorial (1994) Statistics and Computing, 4, pp. 65-85; Medsker, L., Genetic algorithms and neural networks (1995) In: Hybrid Intelligent Systems, pp. 127-143. , Kluwer Academic Publishers; Wermter, S., Riloff, E., Scheler, G., Using hybrid connectionist learning for speech/language analysis (1996) Connectionist, Statistical and Symbolic Approaches to Learning For Natural Language Processing, pp. 87-101. , Springer, Berlin; Zadeh, L.A., Fuzzy logic, neural networks, and soft computing (1994) Commun ACM, 37 (3), pp. 77-84; Paul, R., Macredie, R.M., Baldwin, L.P., Special issue: interdisciplinary approaches to neural computing (1997) Neural Comput Appl, 6 (4), pp. 187-192; Hunter, A., Hare, G., Brown, K., Genetic design of real-time neural network controllers (1997) Neural Comput Appl, 6 (1), pp. 12-18; Jewajinda, Y., Chongstitvatana, P., A parallel genetic algorithm for adaptive hardware and its application to ECG signal classification (2013) Neural Comput Appl, 22 (7-8), pp. 1609-1626; Dorigo, M., Di Caro, G., Gambardella, L.M., Ant algorithms for discrete optimization (1999) Artificial Life, 5 (2), pp. 137-172; Socha, K., Blum, C., An ant colony optimization algorithm for continuous optimization: application to feed-forward neural network training (2007) Neural Comput Appl, 16 (3), pp. 235-247; Reynolds, C., Flocks, herds and schools: A distributed behavioral model (1987) ACM SIGGRAPH Comput Graph, 21 (4), pp. 25-34; Cui, Z., Gao, X., Theory and applications of swarm intelligence (2012) Neural Comput Appl, 21 (2), pp. 205-206; Yang, X.-S., Deb, S., Cuckoo search via Lévy flights (2009) Proceedings of World Congress On Nature & Biologically Inspired Computing (NaBIC 2009), December 2009, India, pp. 210-214. , IEEE Publications, USA; Yang, X.-S., Deb, S., Cuckoo search: Recent advances and applications (2013) Neural Comput Appl, pp. 1-6. , doi: 10. 1007/s00521-013-1367-1; Yang, X.-S., Bat algorithm for multi-objective optimisation (2011) Int J Bio-Inspired Comput, 3 (5), pp. 267-274; Gandomi, A.H., Yang, X.-S., Alavi, A.H., Talatahari, S., Bat algorithm for constrained optimization tasks (2013) Neural Comput Appl, 22 (6), pp. 1239-1255; Greczy, P., Big data-big challenges? (2013) In: Proceedings of the 9th International Conference On Data Mining (DMIN13), Part of WorldComp13, , Las Vegas, July 2013; Kim, K.-J., Lee, W.B., Stock market prediction using artificial neural networks with optimal feature transformation (2004) Neural Comput Appl, 13 (3), pp. 255-260.

142. Redmond SJ, Lee QY, Xie Y, Lovell NH, editors. Applications of supervised learning to biological signals: ECG signal quality and systemic vascular resistance. 34th Annual International Conference of the IEEE Engineering in Medicine and Biology Society, EMBS 2012; 2012; San Diego, CA.

143. Riva G. Applications of Virtual Environments in Medicine. METHODS INF MED. 2003;42(5):524-34.

144. De Groot G, Hollander AP, Sargeant AJ, Van Ingen Schenau GJ, De Boer RW. Applied physiology of speed skating. J SPORTS SCI. 1987;5(3):249-59. doi: 10.1080/02640418708729780.

145. Montpetit RR. Applied Physiology of Squash. SPORTS MED. 1990;10(1):31-41. doi: 10.2165/00007256-199010010-00004.

146. Smith HK. Applied physiology of water polo. SPORTS MED. 1998;26(5):317-34.

147. Liu CH, Lee CS, Wang MH, Tseng YY, Kuo YL, Lin YC. Apply fuzzy ontology and FML to knowledge extraction for university governance and management. J Ambient Intell Humanized Comput. 2013;4(4):493-513. doi: 10.1007/s12652-012-0139-6

10.1007/s00500-011-0785-1; Acampora, G., Loia, V., Using FML and fuzzy technology in adaptive ambient intelligence environments (2005) Int J Comput Intell Res, 1 (2), pp. 171-182; Acampora, G., Loia, V., Fuzzy control interoperability and scalability for adaptive domotic framework (2005) IEEE Trans Ind Inform, 1 (2), pp. 97-111; Afacan, Y., Demirkan, H., An ontology-based universal design knowledge support system (2011) Knowledge-Based Syst, 24 (4), pp. 530-541; Angelo, T.A., (1999) Doing assessment as if learning matters most, , http://www.che.org.il/download/files/angelo.pdf, Accessed 16 Apr 2012; Askling, B., Kristensen, B., Toward the learning organizations: implications for institutional governance and leadership (2000) High Educ Manag, 12 (2), pp. 17-41; Beckwitt, E., Silverstone, S., Bean, D., Creating a culture of academic assessment and excellence via shared governance (2010) Contemp Issues Educ Res, 3 (2), pp. 35-48; Bennett, D., (2008) Templates galore: New approaches to public disclosure, pp. 37-41. , Change Nov/Dec; Bobillo, F., Sraccia, U., Fuzzy ontology representation using OWL2 (2011) Int J Approx Reason, 52 (7), pp. 1073-1094; Buzzigoli, L., Giusti, A., Vivian, A., The evaluation of university departments: a case study for Firenze (2010) Int Adv Econ Res, 16 (1), pp. 24-38; Castiglia, B., Turi, D., The impact of voluntary accountability of the design of higher education assessment (2011) Acad Educ Leadersh J, 15 (3), pp. 119-130; Damiani, E., Ceravolo, P., Leida, M., A toward framework for generic uncertainty management (2009) Proceedings of the Joint 2009 International Fuzzy Systems Association World Congress and 2009 European Society of Fuzzy Logic and Technology Conference (IFSA-EUSFLAT 2009), pp. 1169-1176. , Lisbon, Portugal, Jul 20-24; Chen, R.S., Chen, D.K., Apply ontology and agent technology to construct virtual observatory (2008) Expert Syst Appl, 34 (3), pp. 2019-2028; Durand, J., Pujadas, C., Self-assessment of governance teams in an argentine private university: adapting to difficult times (2004) Tert Educ Manag, 10 (1), pp. 27-44; Elton, L., Task differentiation in universities: toward a new collegiality (1996) Tert Educ Manag, 2 (2), pp. 138-145; Fjortoft, N., Smart, J.C., Enhancing organizational effectiveness: the importance of culture type and mission agreement (1994) High Educ, 27, pp. 429-447; Gaeta, M., Orciuoli, F., Ritrovato, P., Advanced ontology management system for personalized e-Learning (2009) Knowledge-Based Syst, 22 (4), pp. 292-301; Gerber, L.G., Inextricably linked: shared governance and academic freedom (2001) Academe, 87 (3); (2011) Higher Education Evaluation and Accreditation Council of Taiwan (HEEACT), , http://www.heeact.edu.tw/mp.asp?mp=4, Available via; Hoock, J.B., Lee, C.S., Rimmel, A., Teytaud, F., Wang, M.H., Teytaud, O., Intelligent agents for the game of Go (2010) IEEE Comput Intell Mag, 5 (4), pp. 28-42; Ismail, N.A., Activity-based management system implementation in higher education institution (2010) Campus-Wide Inf Sys, 27 (1), pp. 40-52; Kogan, M., Academic and administrative Interface (1999) Changing Relationship between Higher Education and the State, pp. 263-279. , M. Henkel and B. Little (Eds.), London: Jessica Kingsley Publishers; Kovac, V., Ledic, J., Rafajac, B., Academic staff participation in university governance: internal responses to external quality demand (2003) Tert Educ Manag, 9 (3), pp. 215-232; Laskey, K., Laskey, K., Costa, P., (2007) Uncertainty reasoning for the world wide web incubator group charter, , http://www.w3.org/2005/Incubator/urw3/charter, Accessed 16 Apr 2012; Lau, R.Y.K., Song, D., Li, Y., Cheung, T.C.H., Hao, J.X., Toward a fuzzy domain ontology extraction method for adaptive e-leaning (2009) IEEE Trans Knowl Data Eng, 21 (6), pp. 800-813; Lee, C.S., Wang, M.H., A fuzzy expert system for diabetes decision support application (2011) IEEE Trans Syst Man Cybern B Cybern, 41 (1), pp. 139-153; Lee, C.S., Jian, Z.W., Huang, L.K., A fuzzy ontology and its application to news summarization (2005) IEEE Trans Syst Man Cybern B Cybern, 35 (5), pp. 859-880; Lee, C.S., Kao, Y.F., Kuo, Y.H., Wang, M.H., Automated ontology construction for unstructured text documents (2007) Data Knowl Eng, 60 (3), pp. 547-566; Lee, C.S., Wang, M.H., Hagras, H., A type-2 fuzzy ontology and its application to personal diabetic-diet recommendation (2010) IEEE Trans Fuzzy Syst, 18 (2), pp. 374-395; Lee, C.S., Wang, M.H., Acampora, G., Hsu, C.Y., Hagras, H., Diet assessment based on type-2 fuzzy ontology and fuzzy markup language (2010) Int J Intell Syst, 25 (2), pp. 1187-1216; Lee, C.S., Wang, M.H., Su, M.K., Wu, N.Y., Liu, C.H., Tseng, Y.Y., Wang, H.M., Fuzzy markup language for university assessment (2011) Proceedings of the 2011 IEEE International Conference on Systems, Man, and Cybernetics (IEEE SMC 2011), pp. 954-959. , Anchorage, Alaska, USA, Oct 9-12; Maio, C.D., Fenza, G., Loia, V., Senatore, S., Towards an automatic fuzzy ontology generation (2009) Proceedings of the 2009 IEEE International Conference on Fuzzy System (FUZZ-IEEE 2009), pp. 1044-1049. , Jeju Island, Korea, Aug 19-14; Maio, C.D., Fenza, G., Loia, V., Senatore, S., Knowledge structuring to support facet-based ontology visualization (2010) Int J Intell Syst, 25 (12), pp. 1249-1264; Smith, K.D., Taylor, W.G.K., The learning organizational ideal in civil service organizations: deriving a measure (2000) Learn Organ, 7 (4), pp. 194-206; Srikanthan, G., Dalrymple, J., A synthesis of a quality management model for education in universities (2004) Int J Educ Manag, 18 (4-5), pp. 266-279; Sukboonyasatit, K., Thanapaisarn, C., Manmar, L., Key performance indicators of public universities based on quality assessment criteria in Thailand (2011) Contemp Issues Educ Res, 4 (9), pp. 9-18; Villarreal, E., Innovation, organization and governance in Spanish universities (2001) Tert Educ Manag, 7 (2), p. 181; Wang, M.H., Lee, C.S., Hsieh, K.L., Hsu, C.Y., Acampora, G., Chang, C.C., Ontology-based multi-agents for intelligent healthcare applications (2010) J Ambient Intell Humaniz Comput, 1 (2), pp. 111-131; Wang, M.H., Lee, C.S., Acampora, G., Loia, V., Electrocardiogram application based on heart rate variability ontology and fuzzy markup language (2011) ECG Signal Processing, Classification and Interpretation: A Comprehensive Framework of Computational Intelligence, pp. 155-178. , A. Gacek and W. Pedrycz (Eds.), Germany: Springer-Verlag.

148. Coast DA, Stern RM, Cano GG, Briller SA. An Approach to Cardiac Arrhythmia Analysis using Hidden Markov Models. IEEE TRANS BIOMED ENG. 1990;37(9):826-36. doi: 10.1109/10.58593.

149. Bhattacharjee S, Das Z, Das AK, Roy S, Neogi B, editors. An approach towards error less ECG signal equation based on computational simulation aspect with modeling of cardiovascular disorder diagnosis. 2014 International Conference on Control, Instrumentation, Energy and Communication, CIEC 2014; 2014: Institute of Electrical and Electronics Engineers Inc.

150. Fazel R, Gerber TC, Balter S, Brenner DJ, Carr JJ, Cerqueira MD, et al. Approaches to enhancing radiation safety in cardiovascular imaging a scientific statement from the American Heart Association. Circulation. 2014;130(19):1730-48. doi: 10.1161/CIR.0000000000000048.

151. Pien LC. Appropriate use of second-generation antihistamines. Clevel Clin J Med. 2000;67(5):372-80.

152. Oh S, Cha J, Ji M, Kang H, Kim S, Heo E, et al. Architecture design of healthcare software-as-a-service platform for cloud-based clinical decision support service. Healthc Informatics Res. 2015;21(2):102-10. doi: 10.4258/hir.2015.21.2.102.

153. Chimiak WJ, Rainer RO, Chimiak JM, Martinez R. An architecture for naval telemedicine. IEEE Trans Inf Technol Biomed. 1997;1(1):73-9.

154. Wellard SJ, Heggen KM. Are laboratories useful fiction? A comparison of Norwegian and Australian undergraduate nursing skills laboratories. Nurs Health Sci. 2010;12(1):39-44. doi: 10.1111/j.1442-2018.2009.00481.x.

155. Pelliccia A, Adami PE, Quattrini F, Squeo MR, Caselli S, Verdile L, et al. Are Olympic athletes free from cardiovascular diseases? Systematic investigation in 2352 participants from Athens 2004 to Sochi 2014. Br J Sports Med. 2017;51(4):238-43. doi: 10.1136/bjsports-2016-096961.

156. Murphy EK. Are perioperative nurses ‘borrowed servants’? Are surgeons ‘captains of the ship’? AORN Journal. 1994;60(3):474-7. doi: http://doi.org/10.1016/S0001-2092(07)62783-5.

157. Parenti C, Lurie N. Are things different in the light of day? A time study of internal medicine house staff days. The American Journal of Medicine. 1993;94(6):654-8. doi: http://doi.org/10.1016/0002-9343(93)90220-J.

158. Iwane N, editor Arm movement recognition for flag signaling with Kinect sensor. 2012 10th IEEE International Conference on Virtual Environments, Human-Computer Interfaces, and Measurement Systems, VECIMS 2012; 2012; Tianjin.

159. Suberbiola A, Zulueta E, Lopez-Guede JM, Etxeberria-Agiriano I, Graña M. Arm orthosis/prosthesis movement control based on surface EMG signal extraction. Int J Neural Syst. 2015;25(3). doi: 10.1142/S0129065715500094.

160. Mustacchi P. Arterial hypertension and the work environment: Some considerations affecting its compensability. J Occup Med. 1976;18(8):561-6.

161. Lim HW, Hau YW, Lim CW, Othman MA. Artificial intelligence classification methods of atrial fibrillation with implementation technology. Comput Aided Surg. 2016;21:155-62. doi: 10.1080/24699322.2016.1240303.

162. Krenek J, Kuca K, Krejcar O, Maresova P, Sobeslav V, Blazek P, editors. Artificial neural network tools for computerised data modeling and processing. 15th IEEE International Symposium on Computational Intelligence and Informatics, CINTI 2014; 2014: Institute of Electrical and Electronics Engineers Inc.

163. Daponte P, Grimaldi D. Artificial neural networks in measurements. Meas J Int Meas Confed. 1998;23(2):93-115.

164. Schlüter T, Kißels T, Conrad S, editors. AS3: A framework for automatic sleep stage scoring. IADIS Int Conf Intelligent Systems and Agents 2010,ISA, IADIS European Conference on Data Mining 2010,DM, Part of the MCCSIS 2010; 2010; Freiburg.

165. Jozwiak L, Lindwer M, Corvino R, Meloni P, Micconi L, Madsen J, et al. ASAM: Automatic architecture synthesis and application mapping. Microprocessors Microsyst. 2013;37(8 PARTC):1002-19. doi: 10.1016/j.micpro.2013.08.006.

166. Stratos C, Stefanadis C, Kallikazaros I, Boudoulas H, Toutouzas P. Ascending aorta distensibility abnormalities in hypertensive patients and response to nifedipine administration. The American Journal of Medicine. 1992;93(5):505-12. doi: http://doi.org/10.1016/0002-9343(92)90577-X.

167. Blackshear JL, Brott TG. Ascertainment of any and all neurologic and myocardial damage in carotid revascularization: The key to optimization? Exp Rev Cardiovasc Ther. 2013;11(4):469-84. doi: 10.1586/erc.13.25.

168. Tsai IC, Choi BW, Chan C, Jinzaki M, Kitagawa K, Yong HS, et al. ASCI 2010 appropriateness criteria for cardiac computed tomography: A report of the Asian Society of Cardiovascular Imaging cardiac computed tomography and cardiac magnetic resonance imaging guideline Working Group. Int J Card Imaging. 2010;26(SUPPL. 1):1-15. doi: 10.1007/s10554-009-9577-4

10.1016/j.ejrad.2009.06.003; Jinzaki, M., Sato, K., Tanami, Y., Diagnostic accuracy of angiographic view image for the detection of coronary artery stenoses by 64-detector row CT: A pilot study comparison with conventional post-processing methods and axial images alone (2009) Circ J, 73 (4), pp. 691-698. , 10.1253/circj.CJ-08-0798 19225204; (2009) Asia. Wikipedia, the Free Encyclopedia, , http://en.wikipedia.org/wiki/AsiaAccessedon14Sep2009, Wikipedia contributors 14 Sep 2009. Available at.

169. ASE 22nd Annual Scientific Sessions Abstracts. Journal of the American Society of Echocardiography. 2011;24(5):B2-B71. doi: http://doi.org/10.1016/j.echo.2011.03.005.

170. Jones NB, Spurgeon SK, Pont MJ, Twiddle JA, Lim CL, Parikh CR, et al. Aspects of diagnostic schemes for biomedical and engineering systems. IEE Proc Sci Meas Technol. 2000;147(6):357-62. doi: 10.1049/ip-smt:20000859.

171. Gomez BT. Assessing Competency With the Use of Human Patient Simulation in the Emergency Department. Journal of Emergency Nursing. 2009;35(5):476-8. doi: http://doi.org/10.1016/j.jen.2009.06.012.

172. Bond RR, Zhu T, Finlay DD, Drew B, Kligfield PD, Guldenring D, et al. Assessing computerized eye tracking technology for gaining insight into expert interpretation of the 12-lead electrocardiogram: An objective quantitative approach. J Electrocardiol. 2014;47(6):895-906. doi: 10.1016/j.jelectrocard.2014.07.011.

173. Ilgen JS, Humbert AJ, Kuhn G, Hansen ML, Norman GR, Eva KW, et al. Assessing diagnostic reasoning: A consensus statement summarizing theory, practice, and future needs. Acad Emerg Med. 2012;19(12):1454-61. doi: 10.1111/acem.12034.

174. Peleg M, Shahar Y, Quaglini S, Broens T, Budasu R, Fung N, et al. Assessment of a personalized and distributed patient guidance system. International Journal of Medical Informatics. 2017;101:108-30. doi: 10.1016/j.ijmedinf.2017.02.010.

175. Thomsen CE, Rosenfalck A, Christensen KN. Assessment of anaesthetic depth by clustering analysis and autoregressive modelling of electroencephalograms. COMPUT METHODS PROGRAMS BIOMED. 1991;34(2-3):125-38. doi: 10.1016/0169-2607(91)90038-U.

176. Kocabas O, Soyata T, Couderc JP, Aktas M, Xia J, Huang M, editors. Assessment of cloud-based health monitoring using homomorphic encryption. 2013 IEEE 31st International Conference on Computer Design, ICCD 2013; 2013; Asheville, NC: IEEE Computer Society.

177. Ribas Ripoll VJ, Wojdel A, Ramos P, Romero E, Brugada J, editors. Assessment of electrocardiograms with pretraining and shallow networks. 41st Computing in Cardiology Conference, CinC 2014; 2014: IEEE Computer Society.

178. Wang R, Zhang J, Zhang Y, Wang X. Assessment of human operator functional state using a novel differential evolution optimization based adaptive fuzzy model. Biomed Signal Process Control. 2012;7(5):490-8. doi: 10.1016/j.bspc.2011.09.004.

179. Parsinejad P, Sipahi R, editors. Assessment of human vulnerability in a touch-screen game; Metrics and analysis. ASME 2015 Dynamic Systems and Control Conference, DSCC 2015; 2015: American Society of Mechanical Engineers.

180. Bouchoucha S, Wikander L, Wilkin C. Assessment of simulated clinical skills and distance students: Can we do it better? Nurse Education Today. 2013;33(9):944-8. doi: http://doi.org/10.1016/j.nedt.2012.11.008.

181. Schiecke K, Schmidt C, Piper D, Putsche P, Feucht M, Witte H, et al. Assignment of empirical mode decomposition components and its application to biomedical signals. METHODS INF MED. 2015;54(5):461-73. doi: 10.3414/ME14-02-0024.

182. Subhani AR, Likun X, Saeed Malik A, editors. Association of autonomic nervous system and EEG scalp potential during playing 2D grand turismo 5. 34th Annual International Conference of the IEEE Engineering in Medicine and Biology Society, EMBS 2012; 2012; San Diego, CA.

183. Subahni AR, Xia L, Malik AS, editors. Association of mental stress with video games. 2012 4th International Conference on Intelligent and Advanced Systems, ICIAS 2012; 2012; Kuala Lumpur.

184. Cowperthwaite L, Holm R, Kostka J, Reno D. Attendees earn contact hours and take home valuable information from Congress education sessions: Saturday, April 2, to Thursday, April 7, 2005. AORN Journal. 2005;81(6):1209-22. doi: http://doi.org/10.1016/S0001-2092(06)60384-0.

185. Castiglia PT. Attention deficit/hyperactivity disorder. Journal of Pediatric Health Care. 1997;11(3):130-3. doi: http://doi.org/10.1016/S0891-5245(97)90065-1.

186. Cormier E. Attention Deficit/Hyperactivity Disorder: A Review and Update. Journal of Pediatric Nursing. 2008;23(5):345-57. doi: http://doi.org/10.1016/j.pedn.2008.01.003.

187. Vierhile A, Robb A, Ryan-Krause P. Attention-Deficit/Hyperactivity Disorder in Children and Adolescents: Closing Diagnostic, Communication, and Treatment Gaps. Journal of Pediatric Health Care. 2009;23(1, Supplement):S5-S21. doi: http://doi.org/10.1016/j.pedhc.2008.10.009.

188. Mackenzie CF, Hu PF, Horst RL, Group L. An audio-video system for automated data acquisition in the clinical environment. J Clin Monitor Comput. 1995;11(5):335-41. doi: 10.1007/BF01616993.

189. Antonijevic M, Sucic S, Keserica H, editors. Augmented reality for substation automation by utilizing IEC 61850 communication. 39th International Convention on Information and Communication Technology, Electronics and Microelectronics, MIPRO 2016; 2016: Institute of Electrical and Electronics Engineers Inc.

190. Gucciardo A. Augmented Vector Right Lead: A Game Changer in Acute Coronary Syndrome. The Journal for Nurse Practitioners. 2017;13(1):e43-e5. doi: http://doi.org/10.1016/j.nurpra.2016.08.007.

191. Author Index. Journal of Emergency Nursing. 2007;33(6):e19-e22. doi: http://doi.org/10.1016/S0099-1767(07)00637-X.

192. Author Index. Journal of Emergency Nursing. 2010;36(6):e3-e7. doi: http://doi.org/10.1016/S0099-1767(10)00523-4.

193. Author Index. Journal of the American Society of Echocardiography. 2013;26(6):B129-B52. doi: http://doi.org/10.1016/S0894-7317(13)00354-4.

194. Author Index. Journal of the American Society of Echocardiography. 2014;27(6):B122-B44. doi: http://doi.org/10.1016/S0894-7317(14)00345-9.

195. Sieczkowski K, Sondej T, Dobrowolski A, Olszewski R, editors. Autocorrelation algorithm for determining a pulse wave delay. 2016 Signal Processing: Algorithms, Architectures, Arrangements, and Applications, SPA 2016; 2016: IEEE Computer Society.

196. Noronha KP, Acharya UR, Nayak KP, Martis RJ, Bhandary SV. Automated classification of glaucoma stages using higher order cumulant features. Biomed Signal Process Control. 2014;10(1):174-83. doi: 10.1016/j.bspc.2013.11.006.

197. Jenny NZN, Faust O, Yu W. Automated classification of normal and premature ventricular contractions in electrocardiogram signals. J Med Imaging Health Informatics. 2014;4(6):886-92. doi: 10.1166/jmihi.2014.1336.

198. Acharya UR, Sree SV, Muthu Rama Krishnan M, Krishnananda N, Ranjan S, Umesh P, et al. Automated classification of patients with coronary artery disease using grayscale features from left ventricle echocardiographic images. COMPUT METHODS PROGRAMS BIOMED. 2013;112(3):624-32. doi: 10.1016/j.cmpb.2013.07.012.

199. Epstein RH, Dexter F, Piotrowski E. Automated correction of room location errors in anesthesia information management systems. ANESTH ANALG. 2008;107(3):965-71. doi: 10.1213/ane.0b013e31817e7b99.

200. Gilani M, Eklund JM, Makrehchi M, editors. Automated detection of atrial fibrillation episode using novel heart rate variability features. 38th Annual International Conference of the IEEE Engineering in Medicine and Biology Society, EMBC 2016; 2016: Institute of Electrical and Electronics Engineers Inc.

201. Martis RJ, Prasad H, Chakraborty C, Ray AK. Automated detection of atrial flutter and fibrillation using ECG signals in wavelet framework. J Mech Med Biol. 2012;12(5). doi: 10.1142/S0219519412400234.

202. Davari Dolatabadi A, Khadem SEZ, Asl BM. Automated diagnosis of coronary artery disease (CAD) patients using optimized SVM. COMPUT METHODS PROGRAMS BIOMED. 2017;138:117-26. doi: 10.1016/j.cmpb.2016.10.011.

203. Poddar MG, Kumar V, Sharma YP. Automated diagnosis of coronary artery diseased patients by heart rate variability analysis using linear and non-linear methods. J Med Eng Technol. 2015;39(6):331-41. doi: 10.3109/03091902.2015.1063721.

204. Acharya UR, Yanti R, Swapna G, Sree VS, Martis RJ, Suri JS. Automated diagnosis of epileptic electroencephalogram using independent component analysis and discrete wavelet transform for different electroencephalogram durations. Proc Inst Mech Eng Part H J Eng Med. 2013;227(3):234-44. doi: 10.1177/0954411912467883.

205. Andre AD, Jorgenson DB, Froman JA, Snyder DE, Poole JE. Automated external defibrillator use by untrained bystanders:: Can the public-use model work? Prehospital Emergency Care. 2004;8(3):284-91. doi: http://doi.org/10.1016/j.prehos.2004.02.004.

206. Strachan IGD, Hughes NP, Poonawala MH, Mason JW, Tarassenko L. Automated QT analysis that learns from cardiologist annotations. Ann Noninvasive Electrocardiol. 2009;14(SUPPL. 1):S9-S21. doi: 10.1111/j.1542-474X.2008.00259.x.

207. Khandoker AH, Karmakar CK, Palaniswami M. Automated recognition of patients with obstructive sleep apnoea using wavelet-based features of electrocardiogram recordings. Comput Biol Med. 2009;39(1):88-96. doi: 10.1016/j.compbiomed.2008.11.003.

208. Saidatul A, Paulraj MP, Yaacob S, Mohamad Nasir NF, editors. Automated system for stress evaluation based on EEG signal: A prospective review. 2011 IEEE 7th International Colloquium on Signal Processing and Its Applications, CSPA 2011; 2011; Penang.

209. Tsipouras MG, Fotiadis DI. Automatic arrhythmia detection based on time and time-frequency analysis of heart rate variability. COMPUT METHODS PROGRAMS BIOMED. 2004;74(2):95-108. doi: 10.1016/S0169-2607(03)00079-8.

210. Tobon-Gomez C, Butakoff C, Aguade S, Sukno F, Moragas G, Frangi AF. Automatic construction of 3D-ASM intensity models by simulating image acquisition: Application to myocardial gated SPECT studies. IEEE Trans Med Imaging. 2008;27(11):1655-67. doi: 10.1109/TMI.2008.2004819.

211. Rogal Jr SR, Neto AB, Figueredo MVM, Paraiso EC, Kaestner CAA, editors. Automatic detection of arrhythmias using wavelets and self-organized artificial neural networks. 9th International Conference on Intelligent Systems Design and Applications, ISDA 2009; 2009; Pisa.

212. Lalande A, Van Kien PK, Salvé N, Salem DB, Legrand L, Walker PM, et al. Automatic determination of aortic compliance with cinemagnetic resonance Imaging: An application of fuzzy logic theory. Invest Radiol. 2002;37(12):685-91. doi: 10.1097/00004424-200212000-00008.

213. Lei WK, Dong MC, Shi J, Fu BB, editors. Automatic ECG interpretation via morphological feature extraction and SVM inference nets. APCCAS 2008 - 2008 IEEE Asia Pacific Conference on Circuits and Systems; 2008; Macao.

214. Maaoui C, Bousefsaf F, Pruski A. AUTOMATIC HUMAN STRESS DETECTION BASED on WEBCAM PHOTOPLETHYSMOGRAPHIC SIGNALS. J Mech Med Biol. 2016;16(4). doi: 10.1142/S0219519416500391.

215. Griffiths D, Cunningham S, Weinel J, editors. Automatic music playlist generation using affective computing technologies. 5th International Conference on Internet Technologies and Applications, ITA 2013; 2013: Glyndwr University.

216. Miaou SG, Chen ST. Automatic quality control for wavelet-based compression of volumetric medical images using distortion-constrained adaptive vector quantization. IEEE Trans Med Imaging. 2004;23(11):1417-29. doi: 10.1109/TMI.2004.835312.

217. Giakoumis D, Tzovaras D, Moustakas K, Hassapis G. Automatic recognition of boredom in video games using novel biosignal moment-based features. IEEE Trans Affective Comput. 2011;2(3):119-33. doi: 10.1109/T-AFFC.2011.4.

218. Al-Ani T, Karmakar CK, Khandoker AH, Palaniswami M, editors. Automatic recognition of obstructive sleep apnoea syndrome using power spectral analysis of electrocardiogram and hidden markov models. 2008 International Conference on Intelligent Sensors, Sensor Networks and Information Processing, ISSNIP 2008; 2008; Sydney, NSW.

219. Micó P, Mora M, Cuesta-Frau D, Aboy M. Automatic segmentation of long-term ECG signals corrupted with broadband noise based on sample entropy. COMPUT METHODS PROGRAMS BIOMED. 2010;98(2):118-29. doi: 10.1016/j.cmpb.2009.08.010

http://doi.ieeecomputersociety.org/10.1109/SSAP.1996.534872; Raskinis, A., Raskinis, G., Application of symbolic machine learning to audio signal segmentatin (2005) Nonlinear Speech Modeling and Applications, 3445, pp. 397-403; Richman, J.S., Moorman, J.R., Physiological time-series analysis using approximate entropy and sample entropy (2000) American Journal of Physiology-Heart and Circulatory Physiology, 278 (6), pp. H2039-H2049; Clifford, G., Zapanta, L., Janz, B., Mietus, J., Youn, C., Mark, R., (2005), pp. 595-598. , doi:10.1109/CIC.2005.1588171, Segmentation of 24-hour cardiovascular activity using ecg-based sleep/sedation and noise metrics, in: Computers in CardiologyMahmoodi, S., Sharif, B.S., Signal segmentation and denoising algorithm based on energy optimisation (2005) Signal Processing, 85 (9), pp. 1845-1851; Morales, R.O., Sanchez, M.A.P., Ginori, J.V.L., Abalo, R.G., Ramirez, R.R., Evaluation of qrs morphological classifiers in the presence of noise (1997) Computers and Biomedical Research, 30 (3), pp. 200-210; Aboy, M., Cuesta-Frau, D., Austin, D., Mico-Tormos, P., Characterization of sample entropy in the context of biomedical signal analysis (2007) IEEE 29th Annual International Engineering in Medicine and Biology Society Conference (EMBC-07), pp. 5942-5945; Goldberger, A.L., Amaral, L.A.N., Glass, L., Hausdorff, J.M., Ivanov, P.C., Mark, R.G., Mietus, J.E., Stanley, H.E., PhysioBank, PhysioToolkit, and PhysioNet: components of a new research resource for complex physiologic signals (2000) Circulation, 101 (23), pp. e215-e220; Moody, G., Muldrow, W., Mark, R., A noise stress test for arrhythmia detectors (1984) Computers in Cardiology, vol. 11, pp. 381-384; Lake, D., Richman, J.S., Griffin, M.P., Moorman, J.R., Sample entropy analysis of neonatal heart rate variability (2002) American Journal of Physiology. Regulatory, Integrative and Comparative Physiology, 283 (3), pp. 789-797; Laurent, H., Doncarli, C., Stationarity index for abrupt changes detection in the time-frequency plane (1998) IEEE Signal Processing Letters, 5 (2), pp. 43-45.

220. Patil UG, Shirbahadurkar SD, Paithane AN, editors. Automatic speech recognition models: A characteristic and performance review. 2nd International Conference on Computing, Communication, Control and Automation, ICCUBEA 2016; 2016: Institute of Electrical and Electronics Engineers Inc.

221. Iellamo F, Pigozzi F, Spataro A, Di Salvo V, Fagnani F, Roselli A, et al. Autonomic and psychological adaptations in Olympic rowers. J SPORTS MED PHYS FITNESS. 2006;46(4):598-604.

222. Pandey S, Voorsluys W, Niu S, Khandoker A, Buyya R. An autonomic cloud environment for hosting ECG data analysis services. Future Gener Comput Syst. 2012;28(1):147-54. doi: 10.1016/j.future.2011.04.022.

223. Deschamps A, Kaufman I, Backman SB, Plourde G. Autonomic nervous system response to epidural analgesia in laboring patients by wavelet transform of heart rate and blood pressure variability. ANESTHESIOLOGY. 2004;101(1):21-7. doi: 10.1097/00000542-200407000-00006.

224. Steiner S, Abbruzzese E, La Marca R, Ehlert U. Autonomic stress responses elicited by watching a live broadcast soccer game: A pilot study. Gazz Med Ital Arch Sci Med. 2013;172(6):443-8.

225. Riganello F, Cortese MD, Dolce G, Lucca LF, Sannita WG. The Autonomic System Functional State Predicts Responsiveness in Disorder of Consciousness. J Neurotrauma. 2015;32(14):1071-7. doi: 10.1089/neu.2014.3539.

226. Yazdanian H, Nomani A, Yazdchi MR, editors. Autonomous detection of heartbeats and categorizing them by using Support Vector Machines. 2013 20th Iranian Conference on Biomedical Engineering, ICBME 2013; 2013; Tehran: IEEE Computer Society.

227. Paiss O, Inbar GF. Autoregressive Modeling of Surface EMG and Its Spectrum with Application to Fatigue. IEEE TRANS BIOMED ENG. 1987;BME-34(10):761-70. doi: 10.1109/TBME.1987.325918.

228. Kotas M, Pander T, Leski JM. Averaging of nonlinearly aligned signal cycles for noise suppression. Biomed Signal Process Control. 2015;21:157-68. doi: 10.1016/j.bspc.2015.06.003.

229. Rix H, Meste O, Muhammad W. Averaging Signals with Random Time Shift and Time Scale Fluctuations. METHODS INF MED. 2004;43(1):13-6.

230. Jordan CH. Awareness for action. AORN Journal. 1977;25(7):1317-36. doi: http://doi.org/10.1016/S0001-2092(07)67797-7.

231. Casida J, Shpakoff L. Baccalaureate Student Perceptions of Integrating Simulation as a Teaching Strategy in an Acute and Critical Care Nursing Course. Clinical Simulation in Nursing. 2012;8(8):e347-e52. doi: http://doi.org/10.1016/j.ecns.2011.01.008.

232. Geurts E, Haesen M, Dendale P, Luyten K, Coninx K, editors. Back on bike: The BoB mobile cycling app for secondary prevention in cardiac patients. 18th International Conference on Human-Computer Interaction with Mobile Devices and Services, MobileHCI 2016; 2016: Association for Computing Machinery, Inc.

233. Spruce L. Back to Basics: Inhaled Anesthesia. AORN Journal. 2015;102(4):389-95. doi: http://doi.org/10.1016/j.aorn.2015.07.006.

234. Staykova MP, Stewart DV, Staykov DI. Back to the Basics and Beyond: Comparing Traditional and Innovative Strategies for Teaching in Nursing Skills Laboratories. Teaching and Learning in Nursing. 2017;12(2):152-7. doi: http://doi.org/10.1016/j.teln.2016.12.001.

235. Han T, Xiao X, Shi L, Canny J, Wang J, editors. Balancing accuracy and fun: Designing camera based mobile games for implicit heart rate monitoring. 33rd Annual CHI Conference on Human Factors in Computing Systems, CHI 2015; 2015: Association for Computing Machinery.

236. Pérez GM, Swart W, Munyenyembe JK, Saranchuk P. Barriers to pilot mobile teleophthalmology in a rural hospital in Southern Malawi. Pan Afr Med J. 2014;19. doi: 10.11604/pamj.2014.19.136.5196.

237. Santiano N, Daffurn K, Lee A. The Basic Knowledge Assessment Tool: Is It Useful? Australian Critical Care. 1994;7(4):18-23. doi: http://doi.org/10.1016/S1036-7314(94)70698-6.

238. Agezo S, Zhang Y, Ye Z, Chopra S, Vora S, Kurzweg T, editors. Battery-free RFID heart rate monitoring system. 2016 IEEE Wireless Health, WH 2016; 2016: Institute of Electrical and Electronics Engineers Inc.

239. Fan X, Wang J, editors. Bayesheart: A probabilistic approach for robust, low-latency heart rate monitoring on camera phones. 20th ACM International Conference on Intelligent User Interfaces, IUI 2015; 2015: Association for Computing Machinery.

240. Grunkemeier GL, Payne N. Bayesian analysis: A new statistical paradigm for new technology. Ann Thorac Surg. 2002;74(6):1901-8. doi: 10.1016/S0003-4975(02)04535-6.

241. Chu CM, Chien WC, Lai CH, Bludau HB, Tschai HJ, Pai L, et al. A Bayesian expert system for clinical detecting coronary artery disease. J Med Sci(Taiwan). 2009;29(4):187-94.

242. Behar J, Andreotti F, Oster J, Clifford GD, editors. A Bayesian filtering framework for accurate extracting of the non-invasive FECG morphology. 41st Computing in Cardiology Conference, CinC 2014; 2014: IEEE Computer Society.

243. Sutton AJ, Abrams KR. Bayesian methods in meta-analysis and evidence synthesis. Stat Methods Med Res. 2001;10(4):277-303. doi: 10.1191/096228001678227794.

244. Forkan ARM, Khalil I, Ibaida A, Member ZT. BDCaM: Big Data for Context-Aware monitoring-a personalized knowledge discovery framework for assisted healthcare. IEEE Trans Cloud Comput. 2015;PP(99). doi: 10.1109/TCC.2015.2440269.

245. Stone BT, Correa KA, Brown TL, Spurgin AL, Stikic M, Johnson RR, et al. Behavioral and Neurophysiological Signatures of Benzodiazepine-Related Driving Impairments. Front Psychol. 2015;6:1799. Epub 2015/12/05. doi: 10.3389/fpsyg.2015.01799. PubMed PMID: 26635697; PubMed Central PMCID: PMCPMC4659917.

246. Cant RP, Cooper SJ. The benefits of debriefing as formative feedback in nurse education. Aus J Adv Nurs. 2011;29(1):37-47. doi: 10.1111/j.1365-2648.2009.05240.x; Chen, R.P., Norman, D., Huang, L., The use of high-fidelity simulation to implement a weight-based (Broselow) pediatric resuscitation cart system in the pediatric intensive care unit (2007) Pediatric Intensive Care Nursing, 8 (1), pp. 11-14; Cooper, S., Cant, R., Porter, J., Sellick, K., Somers, G., Kinsman, L., Nestel, D., Rating medical emergency team performance: Development of the Team Emergency Assessment Measure (TEAM) (2010) Resuscitation, 81 (4), pp. 446-452. , doi: 10.1016/j.resuscitation.2009.11.027.x; Cooper, S., Kinsman, L., Buykx, P., McConnell-Henry, T., Endacott, R., Scholes, J., Ma naging the deteriorating patient in a simulated environment: Nursing students' knowledge, skill and situational awareness (2010) Journal of Clinical Nursing, 19 (15-16), pp. 2309-2319; Crowe, M., O'Malley, J., Teaching critical refection skills for advanced mental health nursing practice: A deconstructive & reconstructive approach (2006) Journal of Advanced Nursing, 56 (1), pp. 79-87; Cziraki, K., Lucas, J., Rogers, T., Page, L., Zimmerman, R., Hauer, L.A., Daniels, C., Gregoroff, S., Communication and relationship skills for rapid response teams at Hamilton Health Sciences (2008) Healthcare Quarterly (Toronto), 11 (3), pp. 66-71; Decker, S., Sportsman, S., Puetz, L., Billings, L., The evolution of simulation and its contribution to competency (2008) Journal of Continuing Education In Nursing, 39 (2), pp. 74-80; Deering, S., Poggi, S., Macedonia, C., Gherman, R., Satin, A.J., Improving resident competency in the management of shoulder dystocia with simulation training (2004) Obstetrics and Gynecology, 103 (6), pp. 1224-1228; Dine, C., Gersh, R., Leary, M., Riegel, B., Bellini, L., Abella, B., Improving cardiopulmonary resuscitation quality and resuscitation training by combining audiovisual feedback and debriefing (2008) Critical Care Medicine, 36 (10), pp. 2817-2822; Draycott, T.J., Crofts, J.F., Ash, J.P., Wilson, L.V., Yard, E., Sibanda, T., Whitelaw, A., Improving neonatal outcome through practica shoulder dystocia training (2008) Obstetrics & Gynecology, 112 (1), pp. 14-20; Edelson, D., Litzinger, B., Arora, V., Walsh, D., Kim, S., Lauderdale, D., van den Hock, T., Abella, B., Improving in-hospita cardiac arrest and outcomes with performance debriefing (2008) Archives of Internal Medicine, 168 (10), pp. 1063-1069; Fanning, R., Gaba, D., The role of debriefing in simulation-based learning (2007) Simulation In Healthcare, 2 (1), pp. 115-125; Glynn, L., Macfarlane, A., Kelly, M., Cantillon, P., Murphy, A., Helping each other to learn- a process of evaluation of peer assisted earning (2006) BMC Medical Education, 6 (18). , doi: 10.1186/1472-6290/6/18; Hargreaves, J., So how do you feel about that? Assessing refective practice (2004) Nurse Education Today, 24 (3), pp. 196-201; Hogg, G., Pirie, E.S., Ker, J., The use of simulated learning to promote safe blood transfusion practice (2006) Nurse Education In Practice, 6 (4), pp. 214-223; Reland, S., Gilchrist, J., Maconochie, I., Debriefing after failed paediatric resuscitation: A survey of current UK practice (2008) Emergency Medicine Journal, 25 (6), pp. 328-330; Kolb, D., (1984) Experiential Learning: Experiential As Source of Learning and Development, , Prentice Hall, New Jersey; Kuiper, R., Heinrich, C., Matthias, A., Graham, M.J., Bell-Kotwall, L., Debriefing with the OPT model of clinical reasoning during high fdelity patient simulation (2008) International Journal of Nursing Education Scholarship, 5. , Article17; Lambert, V., Glacken, M., Clinical education facilitators: A literature review (2005) Journal of Clinical Nursing, 16 (6), pp. 664-673. , doi 10.1111/j.1365-2702.2005.01136.x; Loyd, G.E., Koenig, H.M., Assessment for learning: Formative evaluations (2008) International Anesthesiology Clinics, 46 (4), pp. 85-96; Mackway-Jones, K., Walker, M., (1999) Pocket Guide For Medical Instructors, , BMJ Books, London; Mikkelsen, J., Reime, M.H., Harris, A.K., Nursing students' learning of managing cross-infections: Scenario-based simulation training versus study groups (2008) Nurse Education Today, 28 (6), pp. 664-671; Minardi, H., Ritter, S., Recording skills practice on videotape can enhance learning- a comparative study between nurse lecturers and nursing students (1999) Journal of Advanced Nursing, 29 (6), pp. 1318-1325; Mitchell, J., When disaster strikes: The critical incident stress debriefing process (1983) Journal of Emergency Medical Services, 8, pp. 36-39; Morgan, P., Tarshis, J., le Blanc, V., Cleave-Hogg, D., Desousa, S., Haley, M.F., Herold-McIlroy, J., Law, J.A., Efficacy of high-fidelitysimulation debriefing on the performance of practicing anaesthetists in simulated scenarios (2009) British Journal of Anaesthesia, 103 (4), pp. 531-537; Neuhauser, C., Learning style and effectiveness of online and face-to-face instruction (2002) American Journal of Distance Education, 16 (2), pp. 99-113; Norris, G., The midwifery curriculum: Introducing obstetric emergency simulation (2008) British Journal of Midwifery, 16 (4), pp. 232-235; Papaspyros, S.C., Javangula, K.C., Adluri, R.K., O'Regan, D.J., Briefing and debriefing in the cardiac operating room. Analysis of impact on theatre team attitude and patient safety (2010) Interactive Cardiovascular & Thoracic Surgery, 10 (1), pp. 43-47; Parker, B., Myrick, F., Transformative learning as context for human patient simulation (2010) Journal of Nursing Education, 10, pp. 1-8; Perera, J., Lee, N., Win, K., Wijesuriya, L., Formative feedback to students: The mismatch between faculty perceptions and student expectations (2008) Medical Teacher, 30 (4), pp. 395-399; Prescott, S., Garside, J., An evaluation of simulated clinical practice for adult branch students (2009) Nursing Standard, 23 (22), pp. 35-40; (2006) Code of Practice For the Assurance of Academic Quality and Standards In Higher Education, , (2nd Ed), Quality Assurance Agency for Higher Education, QAA, Gloucester UK; Rentschler, D., Eaton, J., Cappiello, J., McNally, S., McWilliam, P., Evaluation of undergraduate students using Objective Structured Clinical Evaluation (2007) Journal of Nurse Education, 45 (3), pp. 135-139; Richardson, G., Maltby, H., Refection-on-practice: Enhancing student learning (1995) Journal of Advanced Nursing, 22, pp. 235-242; Rose, S., Bisson, J., Churchill, R., Wessely, S., (2002) Psychological Debriefing For Preventing Post Traumatic Stress Disorder, , (PTSD) Cochrane Database of Systematic Reviews 2: CD000560. doi: 10.1002/14654858. CD00560); Rosenzweig, M., Hravnak, M., Magdic, K., Beech, M., Clifton, M., Arnold, R., Patient communication simulation laboratory for students in an acute care nurse practitioner program (2008) American Journal of Critical Care, 17 (4), pp. 364-372; Salas, E., Wilson, K.A., Burke, C.S., Priest, H.A., Using simulation-based training to improve patient safety: What does it take? (2005) Joint Commission Journal On Quality & Patient Safety, 31 (7), pp. 363-371; Scherer, Y.K., Bruce, S.A., Runkawatt, V., A comparison of clinical simulation and case study presentation on nurse practitioner students' knowledge and confdence in managing a cardiac event (2007) International Journal of Nursing Education Scholarship, 4 (1); Shute, V., (2007) Focus On Formative Feedback, , Educational Testing Service, Princeton, New Jersey; Tiwari, A., Lam, D., Yuen, K., Chan, R., Fung, T., Chan, S., Student learning in clinical nursing education: Perceptions of the relationship between assessment and learning (2005) Nurse Education Today, 25, pp. 299-308; van Emmerik, A., Kamphuis, J., Hulsbosch, A., Emmelkamop, P., Single session debriefing after psychological trauma: A meta-analysis (2002) Lancet, 360, pp. 766-771; Vaughn, L., Baker, R., Teaching in the medical setting: Balancing teaching styles,learning styles and teaching methods (2001) Medical Teacher, 23 (6), pp. 610-612; Weinstock, P.H., Kappus, L.J., Kleinman, M.E., Grenier, B., Hickey, P., Burns, J.P., Toward a new paradigm in hospital-based pediatric education: The development of an onsite simulator program (2005) Pediatric Critical Care Medicine, 6 (6), pp. 635-641; Wisborg, T., Brattebø, G., Brattebø, J., Brinchmann-Hansen, A., Training multi professional trauma teams in Norwegian hospitals using simple and low cost local simulations (2006) Education For Health: Change In Learning and Practice, 19 (1), pp. 85-95.

247. Green M, Ohlsson M, Lundager Forberg J, Björk J, Edenbrandt L, Ekelund U. Best leads in the standard electrocardiogram for the emergency detection of acute coronary syndrome. J Electrocardiol. 2007;40(3):251-6. doi: 10.1016/j.jelectrocard.2006.12.011.

248. Tung A. Best practices for central line insertion. Int Anesthesiol Clin. 2013;51(1):62-78. doi: 10.1097/AIA.0b013e31827da437.

249. Porter R, Tadic V, Achim A, editors. Better than ℓ0 recovery via blind identification. IEEE Global Conference on Signal and Information Processing, GlobalSIP 2015; 2015: Institute of Electrical and Electronics Engineers Inc.

250. Zhou D, He J, Cao Y, Seo JS, editors. Bi-Level rare temporal pattern detection. 16th IEEE International Conference on Data Mining, ICDM 2016; 2017: Institute of Electrical and Electronics Engineers Inc.

251. Swami A, Giannakis GB, Zhou G. Bibliography on higher-order statistics. Signal Process. 1997;60(1):65-126. doi: 10.1016/S0165-1684(97)00065-0.

252. Mughal YM, Le Moullec Y, Annus P, Krivoshei A, editors. A bio-impedance signal simulator (BISS) for research and training purposes. 26th Irish Signals and Systems Conference, ISSC 2015; 2015: Institute of Electrical and Electronics Engineers Inc.

253. Kang SK, Chung KY, Ryu JK, Rim KW, Lee JH. Bio-interactive healthcare service system using lifelog based context computing. Wireless Pers Commun. 2013;73(2):341-51. doi: 10.1007/s11277-013-1242-5

10.1007/s11042-013-1355-6; Chung K., .Y., Yoo, J., Kim K., .J., Recent trends on mobile computing and future networks (2013) Personal and Ubiquitous Computing, , doi: 10.1007/s00779-013-0682-y; Goldberg, D., (1989) Genetic Algorithm in Search, Optimization, and Machine Learning, , 1 Addison-Wesley Boston; Gomez, A., Fernandez, M., Corch, O., (2004) Ontological Engineering, , (2nd ed.). New York, Berlin, Heidelberg; Gonzalez, R.C., Woods, R.E., (1993) Digital Image Processing, , Addison Wesley Reading; Jung, Y.G., Han, M.S., Chung, K.Y., Lee, S.J., A study of a valid frequency range using correlation analysis of throat signal (2011) Information-An International Interdisciplinary Journal, 14 (11), pp. 3791-3799; Kang S., .K., Kim J., .H., Chung K., .Y., Ryu J., .K., Rim K., .W., Lee J., .H., Evolutionary bio-interaction knowledge accumulation for smart healthcare (2012) Proceedings of the 2th International Conference IT Convergence and Security 2012, pp. 425-432. , LNEE 215 2012; Kim S., .H., Chung K., .Y., 3D simulator for stability analysis of finite slope causing plane activity (2013) Multimedia Tools and Applications, , doi: 10.1007/s11042-013-1356-5; Kim J., .H., Chung K., .Y., Ontology-based healthcare context information model to implement ubiquitous environment (2013) Multimedia Tools and Applications, , doi: 10.1007/s11042-011-0919-6; Kim J., .H., Lee, D., Chung K., .Y., Item recommendation based on context-aware model for personalized u-healthcare service (2013) Multimedia Tools and Applications, , doi: 10.1007/s11042-011-0920-0; Kuncheva, L.I., Jain, L.C., Designing classifier fusion systems by genetic algorithms (2000) IEEE Transactions on Evolutionary Computation, 4 (4), pp. 327-336. , 10.1109/4235.887233; Lee, M.S., Relationship of the relative risks of the metabolic syndrome and dietary habits of middle-aged in seoul (2004) Korean J Community Nutr, 9 (6), pp. 695-705; Lee, K.D., Nam, M.Y., Chung, K.Y., Lee, Y.H., Kang, U.G., Context and profile based cascade classifier for efficient people detection and safety care system (2013) Multimedia Tools and Applications, 63 (1), pp. 27-44. , 10.1007/s11042-012-1020-5; Liu, C., Wechsler, H., Evolutionary pursuit and its application to face recognition (2000) IEEE Transactions on Pattern Analysis and Machine Intelligence, 22 (6), pp. 570-582. , 10.1109/34.862196; http://ecg.mit.edu/, MIT-BIH Database DistributionMoghaddam, B., Nastar, C., Pentland, A., A Bayesian similarity measure for direct image matching (1996) Proceedings of the 13th International Conference on Pattern Recognition; Moody, G., Mark, R., The impact of the MIT-BIH Arrhythmia database (2001) IEEE Engineering in Medicine and Biology, pp. 45-50; Mori, N., Kude, T., Matsumoto, K., Adaptation to a dynamic environment by means of the environment identifying genetic algorithm (2000) Industrial Electronics Society IECON 2000, 26th Annual Conference of the IEEE, 4, pp. 2953-2958; Ong, K.G., Dreschel, W.R., Grimes, C.A., Detection of human respiration using square-wave modulated electromagnetic impulses (2003) Microwave and Optical Technology Letters, 35, pp. 339-343. , 10.1002/mop.10759; Pancer, T.P., A suppression of an impulsive noise in ECG signal processing (2004) Proceedings of the 26th Annual International Conference of the IEEE, Engineering in Medicine and Biology Society, pp. 596-599; http://www.physionet.org/physiobank/database/#ecg, PhysioBank Archive IndexRoot, M., Smith, T., Prescribe by risk: The utility of a biomarker-based risk calculation in disease management to prevent heart disease (2005) Disease Management, 8 (2), pp. 106-113. , 10.1089/dis.2005.8.106; Slay, H., Thomas, B., Vernik, R., Piekarski, W., A rapidly adaptive collaborative ubiquitous computing environment to allow passive detection of marked objects (2004) Lecture Notes in Computer Science, 3101, pp. 420-430. , 10.1007/978-3-540-27795-8-42; Song C., .W., Lee, D., Chung K., .Y., Rim K., .W., Lee J., .H., Interactive middleware architecture for lifelog based context awareness (2013) Multimedia Tools and Applications, , doi: 10.1007/s11042-013-1362-7; Song, C.W., Chung, K.Y., Jung, J.J., Rim, K.W., Lee, J.H., Localized approximation method using inertial compensation in WSNs (2011) Information-An International Interdisciplinary Journal, 14 (11), pp. 3591-3600; Turk, M., Pentland, A., Eigenfaces for recognition (1991) Journal of Cognitive Neuroscience, 13 (1), pp. 71-86. , 10.1162/jocn.1991.3.1.71; Yau, S., Wang, Y., Karim, F., Development of situation-aware application software for ubiquitous computing environments (2002) Proceedings of the 26th International Computer Software and Applications Conference, pp. 233-238; Yau S., .S., Wang, Y., Huang, D., In H., .P., A middleware situation-aware contract specification language for ubiquitous computing (2003) Proceedings of the 9th International Workshop on Future Trends of Distributed Computing Systems, pp. 93-99. , Puerto Rico, USA.

254. Johnson DA, Roethig-Johnston K, Richards D. Biochemical and physiological parameters of recovery in acute severe head injury: Responses to multisensory stimulation. Brain Inj. 1993;7(6):491-9. doi: 10.3109/02699059309008176.

255. Murakami A, Kobayashi D, Kubota T, Zukeyama N, Mukae H, Furusyo N, et al. Bioelectrical Impedance Analysis (BIA) of the association of the Japanese Kampo concept "Suidoku" (fluid disturbance) and the body composition of women. BMC Complement Altern Med. 2016;16(1). doi: 10.1186/s12906-016-1373-9.

256. Mei CJ, Thomas CM, Eid M, editors. A biofeedback interactive boxing system for optimal performance. 2014 IEEE International Instrumentation and Measurement Technology Conference: Instrumentation and Measurement for Sustainable Development, I2MTC 2014; 2014; Montevideo: Institute of Electrical and Electronics Engineers Inc.

257. Revill SM, Morgan MDL. Biological quality control for exercise testing. Thorax. 2000;55(1):63-6. doi: 10.1136/thorax.55.1.63.

258. Liu L, Liu J. Biomedical sensor technologies on the platform of mobile phones. Front Mech Eng. 2011;6(2):160-75. doi: 10.1007/s11465-011-0216-0.

259. Zhang Y, Zhang Q, Wu S, editors. Biomedical signal detection based on fractional fourier transform. 5th International Conference on Information Technology and Applications in Biomedicine, ITAB 2008 in conjunction with 2nd International Symposium and Summer School on Biomedical and Health Engineering, IS3BHE 2008; 2008; Shenzhen.

260. Baselli G, Caiani E, Porta A, Montano N, Signorini MG, Cerutti S. Biomedical signal processing and modeling in cardiovascular systems. CRIT REV BIOMED ENG. 2002;30(1-3):55-84. doi: 10.1615/CritRevBiomedEng.v30.i123.40.

261. Mehta SS, Lingayat NS, editors. Biomedical signal processing using SVM. IET-UK International Conference on Information and Communication Technology in Electrical Sciences, ICTES 2007; 2007; Tamil Nadu.

262. Wang J, Liu P, F.H.She M, Nahavandi S, Kouzani A. Biomedical time series clustering based on non-negative sparse coding and probabilistic topic model. COMPUT METHODS PROGRAMS BIOMED. 2013;111(3):629-41. doi: 10.1016/j.cmpb.2013.05.022.

263. Clerico A, Chamberland C, Parent M, Michon PE, Tremblay S, Falk TH, et al., editors. Biometrics and classifier fusion to predict the fun-factor in video gaming. 2016 IEEE Conference on Computational Intelligence and Games, CIG 2016; 2017: IEEE Computer Society.

264. Ciaccio EJ, Dunn SM, Akay M. Biosignal Pattern Recognition And Interpretation Systems. IEEE Eng Med Biol Mag. 1993;12(4):106-13. doi: 10.1109/51.248173.

265. Yu SN, Lee MY. Bispectral analysis and genetic algorithm for congestive heart failure recognition based on heart rate variability. Comput Biol Med. 2012;42(8):816-25. doi: 10.1016/j.compbiomed.2012.06.005.

266. Nikias CL, Raghuveer MR. Bispectrum Estimation: A Digital Signal Processing Framework. Proc IEEE. 1987;75(7):869-91. doi: 10.1109/PROC.1987.13824.

267. Da Silva HP, Lourenço A, Fred A, Martins R. BIT: Biosignal Igniter Toolkit. COMPUT METHODS PROGRAMS BIOMED. 2014;115(1):20-32. doi: 10.1016/j.cmpb.2014.03.002.

268. Arends LR, Hamza TH, Van Houwelingen JC, Heijenbrok-Kal MH, Hunink MGM, Stijnen T. Bivariate random effects meta-analysis of ROC curves. Med Decis Mak. 2008;28(5):621-38. doi: 10.1177/0272989X08319957.

269. Romo Vázquez R, Vélez-Pérez H, Ranta R, Louis Dorr V, Maquin D, Maillard L. Blind source separation, wavelet denoising and discriminant analysis for EEG artefacts and noise cancelling. Biomed Signal Process Control. 2012;7(4):389-400. doi: 10.1016/j.bspc.2011.06.005.

270. San PP, Ling SH, Nguyen HT, editors. Block based neural network for hypoglycemia detection. 33rd Annual International Conference of the IEEE Engineering in Medicine and Biology Society, EMBS 2011; 2011; Boston, MA.

271. Razi A, Afghah F, Belle A, Ward K, Najarian K, editors. Blood loss severity prediction using game theoretic based feature selection. 2014 IEEE-EMBS International Conference on Biomedical and Health Informatics, BHI 2014; 2014; Valencia: IEEE Computer Society.

272. Anuar SHB, Elamvazuthi I, Hanif NHHBM, editors. Blood pressure measuring device embedded with SMS capabilities. 2009 IEEE Student Conference on Research and Development, SCOReD2009; 2009; Serdang.

273. Marenzi B. Body piercing: a patient safety issue. Journal of PeriAnesthesia Nursing. 2004;19(1):4-10. doi: http://doi.org/10.1016/j.jopan.2003.11.002.

274. Huaming L, Jindong T, editors. Body sensor network based ECG segmentation and analysis. 29th Annual International Conference of IEEE-EMBS, Engineering in Medicine and Biology Society, EMBC'07; 2007; Lyon.

275. Poon CCY, Lo BPL, Yuce MR, Alomainy A, Hao Y. Body Sensor Networks: In the Era of Big Data and beyond. IEEE Rev Biomed Eng. 2015;8:4-16. doi: 10.1109/RBME.2015.2427254.

276. Samosky JT, Nelson DA, Wang B, Bregman R, Hosmer A, Mikulis B, et al., editors. BodyExplorerAR: Enhancing a mannequin medical simulator with sensing and projective augmented reality for exploring dynamic anatomy and physiology. 6th International Conference on Tangible, Embedded and Embodied Interaction, TEI 2012; 2012; Kingston, ON.

277. Volpicello C. Bone Cyst of the Acetabulum: A Case Study. AORN Journal. 1991;54(2):291-9. doi: http://doi.org/10.1016/S0001-2092(07)69291-6.

278. Booth Descriptions. AORN Journal. 1993;57(1):290-308. doi: http://doi.org/10.1016/S0001-2092(07)68426-9.

279. Dimoulas C, Kalliris G, Papanikolaou G, Petridis V, Kalampakas A. Bowel-sound pattern analysis using wavelets and neural networks with application to long-term, unsupervised, gastrointestinal motility monitoring. Expert Sys Appl. 2008;34(1):26-41. doi: 10.1016/j.eswa.2006.08.014.

280. Sall H, Timperley J. Bradycardia in anorexia nervosa. BMJ Case Rep. 2015;2015. doi: 10.1136/bcr-2015-211273.

281. Gentili RJ, Rietschel JC, Jaquess KJ, Lo LC, Prevost CM, Miller MW, et al., editors. Brain biomarkers based assessment of cognitive workload in pilots under various task demands. 2014 36th Annual International Conference of the IEEE Engineering in Medicine and Biology Society, EMBC 2014; 2014: Institute of Electrical and Electronics Engineers Inc.

282. Mishra P, Singla SK. Brain biometric: Non-linear analysis of electroencephalogram waveform during imaginary task. J Med Imaging Health Informatics. 2015;5(6):1188-93. doi: 10.1166/jmihi.2015.1512.

283. Xie SY, Guo R, Li NF, Wang G, Zhao HT, editors. Brain fMRI processing and classification based on combination of PCA and SVM. 2009 International Joint Conference on Neural Networks, IJCNN 2009; 2009; Atlanta, GA.

284. Selvam VS, Shenbagadevi S, editors. Brain tumor detection using scalp EEG with modified Wavelet-ICA and multi layer feed forward neural network. 33rd Annual International Conference of the IEEE Engineering in Medicine and Biology Society, EMBS 2011; 2011; Boston, MA.

285. Kim D, Cho SB, editors. A brain-computer interface for shared vehicle control on TORCS car racing game. 2014 10th International Conference on Natural Computation, ICNC 2014; 2014: Institute of Electrical and Electronics Engineers Inc.

286. Carabalona R, Castiglioni P, Gramatica F, editors. Brain-computer interfaces and neurorehabilitation. Stud Health Technol Informatics; 2009 19592793.

287. Visu P, Varunkumar KA, Srinivasan R, Vinoth Kumar R. Brainwave based accident avoidance system for drowsy drivers. Indian J Sci Technol. 2016;9(3):1-5. doi: 10.17485/ijst/2016/v9i3/86381.

288. Rochitte CE, Pinto IMF, Fernandes JL, Azevedo Filho CF, Jatene A, Carvalho ACDC, et al. The Brazilian Society of Cardiology (SBC) guidelines for resonance and cardiovascular tomography: Study group in resonance and cardiovascular tomography (GERT). Arq Bras Cardiol. 2006;87(3):e60-e100.

289. Marshall J, Rowland D, Egglestone SR, Benford S, Walker B, McAuley D, editors. Breath control of amusement rides. 29th Annual CHI Conference on Human Factors in Computing Systems, CHI 2011; 2011; Vancouver, BC.

290. Li X, Yang D, Liu X, Wu XM. Bridging time series dynamics and complex network theory with application to electrocardiogram analysis. IEEE Circuits Syst Mag. 2012;12(4):33-46. doi: 10.1109/MCAS.2012.2221521.

291. Henriquez CS. A brief history of tissue models for cardiac electrophysiology. IEEE TRANS BIOMED ENG. 2014;61(5):1457-65. doi: 10.1109/TBME.2014.2310515.

292. Marshall LS. Broken Heart Syndrome. Journal of Radiology Nursing. 2016;35(2):133-7. doi: http://doi.org/10.1016/j.jradnu.2016.04.002.

293. Marisa T, Niederhauser T, Haeberlin A, Wildhaber RA, Vogel R, Jacomet M, et al. Bufferless Compression of Asynchronously Sampled ECG Signals in Cubic Hermitian Vector Space. IEEE TRANS BIOMED ENG. 2015;62(12):2878-87. doi: 10.1109/TBME.2015.2449901.

294. Jalali L, Jain R, editors. Building health persona from personal data streams. 1st ACM International Workshop on Personal Data Meets Distributed Multimedia, PDM 2013 - Co-located with ACM Multimedia 2013; 2013; Barcelona.

295. He J, Zarei R, Cao J, Taraporewalla K, Steyn M, Van Zundert A, et al., editors. Building the Computational Virtual Reality Environment for Anaesthetists' Training and Practice. IEEE International Conference on Services Computing, SCC 2015; 2015: Institute of Electrical and Electronics Engineers Inc.

296. Hidefjäll P, Titkova D, editors. Business model design for a wearable biofeedback system. 12th International Conference on Wearable Micro and Nano Technologies for Personalized Health, pHealth 2015; 2015: IOS Press.

297. Calendar. Air Medical Journal. 1994;13(10):449-53. doi: http://doi.org/10.1016/S1067-991X(05)80067-7.

298. Calendar of events. The American Journal of Medicine. 1988;85(6):A19-A48. doi: http://doi.org/10.1016/S0002-9343(88)80015-9.

299. Rainwater JA, Romano PS, Antonius DM. The California Hospital Outcomes Project: How Useful Is California’s Report Card for Quality Improvement? The Joint Commission Journal on Quality Improvement. 1998;24(1):31-9. doi: http://doi.org/10.1016/S1070-3241(16)30357-1.

300. Kremser AK, Lyneham J. Can Australian Nurses Safely Assess for Thrombolysis on EKG Criteria? Journal of Emergency Nursing. 2007;33(2):102-9. doi: http://doi.org/10.1016/j.jen.2006.10.015.

301. Husain I, Spence D. Can healthy people benefit from health apps? BMJ (Online). 2015;350. doi: 10.1136/bmj.h1887.

302. Smith D, Miller DG, Cukor J. Can simulation measure differences in Task-Switching ability between junior and senior emergency medicine residents? West J Emerg Med. 2016;17(2):149-52. doi: 10.5811/westjem.2015.12.28269.

303. Kumar ATK, Asamoah D, Sharda R, editors. Can social media support public health? Demonstrating disease surveillance using big data analytics. 21st Americas Conference on Information Systems, AMCIS 2015; 2015: Americas Conference on Information Systems.

304. Badgett RG, Lucey CR, Mulrow CD. Can the clinical examination diagnose left-sided heart failure in adults? J AM MED ASSOC. 1997;277(21):1712-9.

305. Tasch C, Larcher L. Can triggers be cumulative in inducing heart attack in soccer game spectators? Wien Med Wochenschr. 2012;162(15-16):337-9. doi: 10.1007/s10354-012-0137-5.

306. Tillinghast SJ. Can Western Quality Improvement Methods Transform the Russian Health Care System? The Joint Commission Journal on Quality Improvement. 1998;24(5):280-98. doi: http://doi.org/10.1016/S1070-3241(16)30381-9.

307. Canadian Cardiovascular Society (CCS) CCS825 Oral: Treatment and Outcomes in Acute Coronary Syndromes (ACS) Tuesday, October 26, 2010. Canadian Journal of Cardiology. 2010;26, Supplement D:110D-1D. doi: http://doi.org/10.1016/S0828-282X(10)71139-9.

308. Howlett JG, Chan M, Ezekowitz JA, Harkness K, Heckman GA, Kouz S, et al. The Canadian Cardiovascular Society Heart Failure Companion: Bridging Guidelines to Your Practice. Canadian Journal of Cardiology. 2016;32(3):296-310. doi: http://doi.org/10.1016/j.cjca.2015.06.019.

309. Shimazaki T, Hara S, Okuhata H, Nakamura H, Kawabata T, editors. Cancellation of motion artifact induced by exercise for PPG-based heart rate sensing. 2014 36th Annual International Conference of the IEEE Engineering in Medicine and Biology Society, EMBC 2014; 2014: Institute of Electrical and Electronics Engineers Inc.

310. Hall EJ, Brenner DJ. Cancer risks from diagnostic radiology. Br J Radiol. 2008;81(965):362-78. doi: 10.1259/bjr/01948454.

311. Greydanus DE, Merrick J. Cannabis or marijuana: A review. J Pain Manage. 2016;9(4):347-73. doi: 10:1136/bmj.e536; Li, M.C., Brady, J.E., DiMaggio, C.J., Lusardi, A.R., Tzong, K.Y., Li, G., Marijuana use and motor vehicle crashes (2012) Epidemiol Rev, 34 (1), pp. 65-72; Drabek, M., Andysz, A., Effects of marijuana and amphetamine (and its derivatives) on driving performance based on the driving simulator studies (2011) Med Pr, 62 (5), pp. 551-563; Johnson, M.B., Kelley-Baker, G., Voas, R.B., Lacey, J.H., The prevalence of cannabis-involved driving in California (2012) Drug Alcohol Depend, 123 (1-3), pp. 105-109; Bosker, W.M., Kuypers, K.P., Thenunissen, E.L., Surinx, A., Blankenspoor, R.J., Skopp, G., Medicinal Δ(9)- tetrahydrocannabinol (dronabinol) impairs on-the-road driving performance of occasional and heavy cannabis users but is not detected in Standard Field Sobriety Tests (2012) Addiction, 107 (10), pp. 1837-1844; Barrio, G., Jimenez-Mejias, E., Pulido, J., Lardelli-Claret, P., Bravo, M.J., de la Fuenta, L., Association between cannabis use and non-traffic injuries (2012) Accid Anal Prev, 47, pp. 172-176; Campos, D.R., Yonamine, M., de Morases Moreau, R.L., Marijuana as doping in sports (2003) Sports Med, 33 (6), pp. 395-399; Saugy, M., Avois, L., Saudan, C., Robinson, N., Giroud, C., Mangin, P., Cannabis and sports (2006) Br J Sports Med, 40, pp. 13-15; Pope, H.G., Jr., Gruber, A.J., Yurgelun-Todd, D., The residual neuropsychological effects of cannabis: The current status of research (1995) Drug Alcohol Depend, 38 (1), pp. 25-34; Martin-Santos, F., Fagundo, A.B., Crippa, J.A., Atakan, Z., Bhattacharyya, S., Allen, P., Neuroimaging in cannabis use: A systemic review of the literature (2010) Psychol Med, 40 (3), pp. 383-398; Crippa, J.A., Lacerda, A.L., Amaro, E., Busatto Filho, G., Zuardi, A.W., Bressan, R.A., Brain effects of cannabis- neuroimaging findings (2005) Rev Bras Psiquiatr, 27 (1), pp. 70-78; Schreiner, A.A., Dunn, M.E., Residual effects of cannabis use on neurocognitive performance after prolonged abstinence: A meta-analysis (2012) Exp Clin Psychopharmacol, , Jun 25; Montgomery, C., Seddon, A.L., Fisk, J.E., Murphy, P.N., Jansari, A., Cannabis-related deficits in real-world memory (2012) Hum Psychopharmacol, 27 (2), pp. 217-225; Marmorstein, N.R., Iacono, W.G., McGue, M., Associations between substance use disorders and major depression in parents and late adolescent-emerging adult offspring:An adoption study (2012) Addiction, 107 (11), pp. 1965-1973; Harvey, M.A., Sellman, J.D., Porter, R.J., Frampton, C.M., The relationship between non-acute adolescent cannabis use and cognition (2007) Drug Alcohol Res, 26 (3), pp. 309-319; Sundram, S., Cannabis and neurodevelopment:Implications for psychiatric disorders (2006) Hum Psychopharmacol, 21 (4), pp. 245-254; Schneider, M., Puberty as a highly vulnerable developmental period for the consequences of cannabis exposure (2008) Addict Biol, 13 (2), pp. 253-263; Dinieri, J.A., Hurd, Y.L., Rat models of prenatal and adolescent cannabis exposure (2012) Methods Mol Biol, 829, pp. 231-242; Degenhardt, L., Coffey, C., Romaniuk, H., Swift, W., Carlin, J.B., Hall, W.D., The persistence of the association between adolescent cannabis use and common mental disorders into young adulthood (2013) Addiction, 108 (1), pp. 124-133; Urban, N.B., Slifstein, M., Thompson, J.L., Xu, X., Grigis, R.R., Raheja, S., Dopamine release in chronic cannabis uers: A [(11) C] raclopride positron emission tomography study (2012) Biol Psychiatry, 71 (8), pp. 677-683; Zalesky, A., Solowij, N., Yücel, M., Lubman, D.I., Takagi, M., Harding, I.H., Effect of long-term cannabis use on axonal fibre connectivity (2012) Brain, 135, pp. 2245-2255; Leach, L.S., Butterworth, P., The effect of early onset common mental disorders on educational attainment in Australia (2012) Psychiatry Res, 199 (1), pp. 51-57; Yücel, M., Solowij, N., Respondek, C., Whittle, S., Fornito, A., Pantelis, C., Regional brain abnormalities associated with long-term heavy cannabis use (2008) Arch Gen Psychiatry, 65 (6), pp. 694-701; Abushi, H., Akirav, I., Short-and long-term cognitive effects of chronic cannabinoids administration in lateadolescence rats (2012) PLoS One, 7 (2); Gordon, S.M., Tulak, F., Troncale, J., Prevalence and characteristics of adolescent patients with co-occurring ADHD and substance dependence (2004) J Addict Dis, 23, pp. 31-40; Dennis, M., Godley, S.H., Diamond, G., Tims, F.M., Babor, T., Donaldson, J., The Cannais Youth Treatment (CYT) Study: Main findings from two randomized trials (2004) J Subst Abuse Treat, 27 (3), pp. 197-213; Han, S., Yang, B.Z., Kranzler, H.R., Oslin, D., Anton, R., Farrer, L.A., Linkage analysis followed by association shown NRG1 associated with cannabis dependence in African Americans (2012) Biol Psychiatry, 72 (8), pp. 637-644; Hürlimann, F., Kupferschmid, S., Simon, A.E., Cannabisinduced depersonalization disorder in adolescence. Cannabis-induced depersonalization disorder in adolescents (2012) Neuropsychobiology, 65 (3), pp. 141-146; Horwood, L.J., Fergusson, D.M., Coffey, C., Patton, G.C., Tait, R., Smart, D., Cannabis and depression: An integrative data analysis of four Australasian cohorts (2012) Drug Alcohol Depend, 126 (3), pp. 369-378; Van Dam, N.T., Bedi, G., Earleywine, M., Characteristics of clinically anxious versus non- anxious regular, heavy marijuana users (2012) Addict Behav, 37 (11), pp. 1217-1223; Budney, A.J., Should cannabis withdrawal disorder be included in DSM-5? (2011) Psychiatric Times, 28 (2), pp. 48-50; Budney, A.J., Hughes, J.R., Moore, B.A., Vandrey, R., Review of the validity and significance of cannabis withdrawal syndrome (2004) Am J Psychiatry, 161, pp. 1967-1977; Budney, A.J., Hughes, J.R., The cannabis withdrawal syndrome (2006) Curr Opin Psychiatry, 19, pp. 233-238; Crowley, T.J., MacDonald, M.J., Whitmore, E.A., Cannabis dependence, withdrawal, and reinforcing effects among adolescents with conduct symptoms and substance use disorders (1998) Drug Alcohol Depend, 50, pp. 27-37; Vandrey, R.G., Budney, A.J., Hughes, J.R., Liguoria, A., A within-subject comparison of withdrawal symptoms during abstinence from cannabis, tobacco, and both substances (2008) Drug Alcohol Depend, 92 (1-3), pp. 48-54; Arendt, M., Rosenberg, R., Fjordback, L., Brandholdt, J., Foldager, L., Sher, L., Testing the self-medication hypothesis of depression and aggression in cannabisdependent subjects (2007) Psychol Med, 37 (7), pp. 935-945; Calles Jr, J.L., Nazeer, A., Aggressive and violent behavior (2011) Clinical aspects of psychopharmacology in childhood and adolescence., p. 106. , Greydanus DE, Calles JL Jr, Patel DR, Nazeer A, Merrick J, eds, New York: Nova Science; Schacht, J.P., Hutchinson, K.E., Filbey, F.M., Associations between cannabinoid-receptor-1 (CNR1) variation and hippocampus and amygdale volumes in heavy cannabis users (2012) Neuropsychopharmacology, 37 (11), pp. 2368-2376; Haney, M., Hart, C.L., Vosburg, K.D., Marijuana withdrawal in humans: Effects of oral THC or divalproex (2004) Neuropsychopharmacology, 29, pp. 158-170; Milin, R., Manion, I., Dare, G., Walker, S., Prospective assessment of cannabis withdrawal in adolescents with cannabis dependence: A pilot study (2008) J Am Acad Child Adolesc Psychiatry, 47, pp. 174-178; Cornelius, J.R., Chung, T., Martin, C., Cannabis withdrawal is common among treatment seeking adolescents with cannabis dependence and major depression, and is associated with rapid relapse to dependence (2008) Addict Behav, 103, pp. 787-799; Gorelick, D.A., Levin, K.H., Copersino, M.L., Heishman, S.J., Liu, F., Boggs, D.L., Diagnostic criteria for cannabis withdrawal syndrome (2012) Drug Alcohol Depend, 123 (1-3), pp. 141-147; Gardner, E.L., Addictive potential of cannabinoids: The underlying neurobiology (2005) Chem Phys Lipids, 31 (1-2), pp. 267-290. , 121; Cooper, Z.D., Haney, M., Cannabis reinforcement and dependence: Role of cannabinoid CB1 receptor (2008) Addict Biol, 13 (2), pp. 188-195; Cooper, Z.D., Haney, M., Actions of delta-9- tetrahydrocannabinol in cannabis: Relation to use, abuse, dependence (2009) Int Rev Psychiatry, 2192, pp. 104-112; Gardner, E.L., Endocannabinoid signaling system and brain reward: Emphasis on dopamine (2005) Pharmacol Biochem Behav, 81 (2), pp. 263-284; Gardner, E.L., Addiction and brain reward and antireward pathways (2011) Adv Psychosom Med, 30, pp. 22-60; Bossong, M.G., van Berckel, B.N., Boellaard, R., Zuurman, L., Schuit, R.C., Windhorst, A.D., Delta 9- tetrahydrocannabinol induces dopamine release in the human striatum (2009) Neuropsychopharmacology, 34 (3), pp. 759-766; Horey, J.T., Mariani, J.J., Cheng, W.Y., Bisaqa, A., Sullivan, M., Nunes, E., Comparison of substance use milestones in cannabis-and cocaine-dependent patients (2012) J Addict Dis, 31 (1), pp. 60-66; Goldstein, R.Z., Volkow, N.D., Dysfunction of the prefrontal cortex in addiction: Neuroimaging findings and clinical implications (2011) Nat Rev Neurosci, 12 (11), pp. 652-669; Goldstein, R.Z., Volkow, N.D., Drug addiction and its underlying neurobiological basis: Neuroimaging evidence for the involvement of the frontal cortex (2002) Am J Psychiatry, 159 (10), pp. 1642-1652; Feil, J., Sheppard, D., Fitzgerald, P.B., Yücel, M., Lubman, D.I., Bradshaw, J.L., Addiction, compulsive drug seeking, and the role of frontostriatal mechanisms in regulating inhibitory control (2010) Neurosci Biobehav Rev, 35 (2), pp. 248-275; Rotter, A., Bayerlein, K., Hansbauer, M., Weiland, J., Sperling, W., Kornhuber, Orexin A expression and promoter methylation in patients with cannabis dependence in comparison to nicotine-dependent cigarette smokers and nonsmokers (2012) Neuropsychobiology, 66 (2), pp. 126-133; Schafer, G., Feilding, A., Morgan, C.G., Agathangelou, M., Freeman, T.P., Valerie Curran, H., Investigating the interaction between schizotypy, divergent thinking, and cannabis use (2002) Conscious Cogn, 21 (1), pp. 292-298; Galvez-Buccollini, J.A., Proal, A.C., Tomaselli, V., Trachtenberg, M., Coconcea, C., Chun, J., Association between age at onset of psychosis and age at onset of cannabis use in non-affective psychosis (2012) Schizophr Res, 139 (1-3), pp. 157-160; Giovanni, M., Giuseppe, D.I., Gianna, S., Domenico, D.B., Luisa, D.R., Massimo, D.G., Cannabis use and psychosis:Theme introduction (2012) Curr Pharm Des, 18 (32), pp. 4991-4998; Leweke, F.M., Koethe, D., Cannabis and psychiatric disorders: It is not only addiction (2008) Addict Biol, 13 (2), pp. 264-275; Nazeer, A., Calles Jr, J.L., Schizophrenia in children and adolescents (2011) Clinical Aspects of Psychopharmacology in Childhood and Adolescence., p. 152. , Greydanus DE, Calles Jr JL, Patel DR, Nazeer A, Merrick J, eds, New York: Nova Science; Van Dijk, D., Koeter, M.W., Hijman, R., Kahn, R.S., van den Bring, W., Effect of cannabis use on the course of schizophrenia in male patients: A prospective cohort study (2012) Schizophr Res, 137 (1-3), pp. 50-57; Bossong, M.G., Niesink, R.J., Adolescent brain maturation, the endogenous cannabinoid system, and the neurobiology of cannabis-induced schizophrenia (2010) Prog Neurobiol, 92 (3), pp. 370-385; Rapp, C., Bugra, H., Riecher-Rössler, A., Borgwardt, S., Effects of cannabis use on human brain structure in psychosis: A systematic review combining in vivo structural neuroimaging and post-mortem studies (2012) Curr Pharm Des, 18 (32), pp. 5070-5080; Khan, M.K., Usmani, M.A., Hanif, S.A., A case of self amputation of penis by cannabis induced psychosis (2012) J Forensic Leg Med, 19 (6), pp. 355-357; Barrowclough, C., Emsley, R., Eisner, E., Beardmore, R., Wykes, T., Does change in cannabis use in established psychosis affect clinical outcome? (2013) Schizophr Bull, 39 (2), pp. 339-348; Leweke, F.M., Anandamide dysfunction in prodromal and established psychosis (2012) Curr Pharm Des, 18 (32), pp. 5188-5193; Decoster, J., van Os, J., Myin-Germeys, I., De Hert, M., van Winkel, R., Genetic variation underlying psychosisinducing effects of cannabis: Critical review and future directions (2012) Curr Pharm Des, 18 (32), pp. 5015-5023; Serafini, G., Pompili, M., Innamorati, M., Rihmer, Z., Sher, L., Girardi, P., Can cannabis increase the suicide risk in psychosis? A critical review (2012) Curr Pharm Des, 18 (32), pp. 5165-5187; Hermann, D., Schneider, M., Potential protective effects of cannabidol on neuroanatomical alterations in cannabis users and psychosis: A critical review (2012) Curr Pharm Des, 18 (32), pp. 4897-4905; Lev-Ran, S., Aviram, A., Braw, Y., Nitzan, U., Ratzoni, G., Fennig, S., Clinical correlates of cannabis use among adolescent psychiatric inpatients (2012) Eur Psychiatr, 27 (6), pp. 470-475; Auther, A.M., McLaughlin, D., Carrión, R.E., Naqachandran, P., Correll, C.U., Cornblatt, B.A., Prospective study of cannabis use in adolescents at clinical high risk for psychosis: Impact of conversion to psychosis and functional outcome (2012) Psychol Med, 30, pp. 1-13; Zuardi, A.W., Crippa, J.A., Bhattacharyya, S., Atakan, Z., Martin-Santos, R., A critical review of the antipsychotic effects of Cannabidiol: 30 years of translational investigation (2012) Curr Phar Des, 18 (32), pp. 5131-5140; Bhattacharyya, S., Crippa, J.A., Allen, P., Martin-Santos, R., Borgwardt, S., Fusar-Poli, P., Induction of psychosis byδ9-tetrahydrocannabinol reflects modulation of prefrontal and striatal function during attentional salience processing (2012) Arch Gen Psychiatry, 69 (1), pp. 7-36; Blum, K., Gardner, E., Oscar-Berman, M., Gold, M., "Liking" and "wanting" linked to reward deficiency syndrome (RDS): Hypothesizing differential responsivity in brain reward circuitry (2012) Curr Pharm Des, 18 (1), pp. 113-118; Bailén, A.J.R., Quesada, P.S., Valladares, M.A., Decision making in cannabis users (2012) Adicciones, 24 (2), pp. 161-172; Van Ours, J.C., Williams, J., The effects of cannabis use on physical and mental health (2012) J Health Econ, 31 (4), pp. 564-577; Schifano, F., Martinotti, G., Cunniff, A., Reissner, V., Scherbaum, N., Ghodse, H., Impact of an 18- month, NHS-based, treatment exposure for heroin dependence:Results from the London Area Treat 2000 Study (2012) Am J Addict, 21 (3), pp. 268-273; Porath-Waller, A.J., Beasley, E., Beirness, D.J., A metaanalytic review of school-based prevention for cannabis use (2010) Health Educ Behav, 37 (5), pp. 709-723; Teesson, M., Newton, N.C., Barrett, E.L., Australian schoolbased prevention programs for alcohol and other drugs:A systemic review (2012) Drug Alcohol Rev, 31 (6), pp. 731-736. , doi:10.1111/j.1465-3362.2012.00420.x; De Looze, M., Harakeh, Z., van Dorsselaer, S.A., Raaijmakers, Q.A., Vollebergh, W.H., Bogt, T.F., Explaining educational differences in adolescent substance use and early sexual debut: The role of parents and peers (2012) J Adolesc, 35 (4), pp. 1035-1044; Yap, M.B., Reavley, N.J., Jorm, A.F., Young people’s beliefs about the harmfulness of alcohol, cannabis, and tobacco for mental disorders: Findings from two Australian national youth surveys (2012) Addiction, 107 (4), pp. 838-847; Gillespie, N.A., Lubke, G.H., Gardner, C.O., Neale, M.C., Keanler, K.S., Two-part random effects growth modeling to identify risks associated with alcohol and cannabis initiation, initial average use, and changes in drug consumption in s sample of adult, male twins (2012) Drug Alcohol Depend, 123 (1-3), pp. 220-228; Abdoul, H., Le Faou, A.L., Bouchez, J., Touzeau, D., Lagrue, G., Cannabis cessation Interventions offered to young French users: Predictors of follow-up (2012) Encaphale, 38 (2), pp. 141-148; Scott, L.A., Roxburgh, A., Bruno, R., Matthews, A., Burns, L., The impact of comorbid cannabis and methamphetamine use on mental health among regular ecstasy users (2012) Addict Behav, 37 (9), pp. 1058-1062; Friedmann, P.D., Green, T.C., Taxman, F.S., Harrington, M., Rhodes, A.G., Katz, E., Collaborative behavioral management among parolees: Drug use, crime & rearrest in the Stepŉ Out randomized trial (2012) Addiction, 107 (6), pp. 1099-1108; Baker, A.L., Thornton, L.K., Hides, L., Dunlop, A., Treatment of cannabis use among people with psychotic disorders:A critical review of randomised control trials (2012) Curr Pharm Des, 18 (32), pp. 4923-4937; Buckner, J.D., Zvolensky, M.J., Schmidt, N.B., Cannabisrelated impairment and social anxiety: The roles of gender and cannabis use motives (2012) Addict Behav, 37 (11), pp. 1294-1297; Carroll, K.M., Nich, C., Lapaglia, D.M., Peters, E.N., Easton, C.J., Petry, N.M., Combining cognitive behavioral therapy and contingency management to enhance their effects in treating cannabis dependence: Less can be more, more or less (2012) Addiction, 107 (9), pp. 1650-1659; Hendriks, V., van der Schee, E., Blanken, P., Matching adolescents with a cannabis use disorder to multidimensional family therapy or cognitive behavioral therapy: Treatment effect moderators in a randomized controlled trial (2012) Drug Alcohol Depend, 125 (1-2), pp. 119-126; Baker, A.L., Hides, L., Lubman, D.I., Treatment of cannabis use among people with psychotic or depressive disorders: A systemic review (2010) J Clin Psychiatry, 71 (3), pp. 247-254; Gates, P.J., Norberg, M.M., Copeland, J., Digiusto, E., Randomised controlled trial of a noval cannabis use intervention delivered by telephone (2012) Addiction, 107 (12), pp. 2149-2158; Lindsey, W.T., Stewart, D., Childress, D., Drug interactions between common illicit drugs and prescription therapies (2012) Am J Drug Alcohol Abuse, 38 (4), pp. 334-343; Vandrey, R., Haney, M., Pharmacotherapy for cannabis dependence: How close are we? (2009) CNS Drugs, 23, pp. 543-553; Hjorthøj, C., Fohlmann, A., Nordentoft, M., Treatment of cannabis use disorders in people with schizophrenia spectrum disorders-a systemic review (2009) Addict Behav, 34 (6-7), pp. 520-525; Agrawal, A., Verweil, K.J., Gillespie, N.A., Health, A.C., Lessov-Schlaggar, C.N., Martin, C.N., The genetics of addiction-a translational perspective (2012) Transl Psychiatry, 2; Crippa, J.A., Derenusson, G.N., Chagas, M.H., Atakan, Z., Matin-Santos, R., Zuardi, A.W., Pharmacologic interventions in the treatment of the acute effects of cannabis: A systemic review of literature (2012) Harm Reduct J, 9 (1), p. 7; Crippa, J.A., Hallak, J.E., Machado-de-Sousa, J.P., Queiroz, R.H., Bergamaschi, M., Chagus, M.H., Cannabidol for the treatment of cannabis withdrawal syndrome: A case report (2013) J Clin Pharm Ther, 38 (2), pp. 162-164. , Doi:10.1111/jcpt.12018; Vandrey, R., Stitzer, M.L., Mintzer, M.Z., Huestis, M.A., Murray, J.A., Lee, D., The dose effects of short-term dronabinol (oral THC) maintenance in daily cannabis users (2013) Drug Alcohol Depend, 128 (1-2), pp. 64-70; Haney, M., Hart, C.L., Vosburg, K.D., Effects of THC and lofexidine in a human laboratory model of marijuana withdrawal and relapse (2008) Psychopharmacology (Berl), 197, pp. 157-168; Bedi, G., Cooper, Z.D., Haney, M., Subjective, cognitive and cardiovascular dose-effect profile of nabilone and dronabinol in marijuana smokers (2013) Addict Biol, 18 (5), pp. 872-881. , doi:10.1111/j.1369-1600.2011.00427.x; Kleinloog, D., Liem-Moolenaar, M., Jacobs, G., Klaassen, E., de Kam, M., Hijman, R., Does olanzapine inhibit the psychomimetic effects of {Delta} 9- tetrahydrocannabinol? (2012) J Psychopharmacol, 26 (10), pp. 1307-1316; Sarnyai, Z., Oxytocin and neuroadaptation to cocaine (1998) Prog Brain Res, 119, pp. 449-466; Baskerville, T.A., Douglas, A.J., Dopamine and oxytocin interactions underlying behaviors: Potential contributions to behavioral disorders (2010) CNS Neurosci Ther, 16 (3), pp. e92-e123; McGregor, I.S., Bowen, M.T., Breaking the loop: Oxytocin as a potential treatment for drug addiction (2012) Horm Behav, 61 (3), pp. 331-339; Gray, K.M., Carpenter, M.J., Baker, N.L., Desantis, S.M., Kryway, E., Hartwell, K.J., A double- blind randomized controlled trail of N-acetylcysteine in cannabis-dependent adolescents (2012) Am J Psychiatry, 169 (8), pp. 805-812; Weinstein, A.M., Gorelicki, D.A., Pharmacologic treatment of cannabis dependence (2011) Curr Pharm Des, 17 (14), pp. 1351-1358; Pertwee, R.G., Cannabis and cannabinoids: Pharmacology and rationale for clinical use (1999) Foresch Komplementarmed, 6, pp. 12-15; Svrakic, D.M., Lustman, P.J., Mallya, A., Lynn, T.A., Finney, R., Svrakic, N.M., Legalization, decriminalization & medicinal use of cannabis: A scientific and public health perspective (2012) Mo Med, 109 (2), pp. 90-98; Beaulieu, P., Rice, A.S., The pharmacology of cannabinoids derivatives: Are there applications to treatment of pain? (2002) Ann Fr Anesth Rhanim, 21 (6), pp. 493-508; Lee, M.C., Ploner, M., Wiech, K., Bingel, U., Wanigasekera, V., Brooks, J., Amygdala activity contributes to the dissociative effect of cannabis on pain perception (2013) Pain, 154 (1), pp. 124-134; Richardson, G.A., Larkby, C., Goldschmidt, L., Day, N.L., Adolescent initiation of drug use effects of prenatal cocaine exposure (2013) J Am Acad Child Adolesc Psychiatry, 52 (1), pp. 37-46; Mercolini, L., Mandrioli, R., Sorella, V., Somaini, L., Giocondi, D., Serpelloni, G., Dried blood spots:Liquid chromatography-mass spectrometry analysis of delta-9-tetrahydrocannabinol and its main metabolites (2013) J Chromatogr A, 1271 (1), pp. 33-40; Danovitch, I., Gorelick, D.A., State of the art treatments for cannabis dependence (2012) Psychiatr Clin North Am, 35 (2), pp. 309-326; Forray, A., Sofuoglu, M., Future pharmacologic treatments for substance use disorders (2014) Br J Clin Pharmacol, 77 (2), pp. 382-400. , doi.10.1111/j.1365-2125.2012.04474.x; Greydanus, D.E., Feucht, C., Hawver, E.K., Substance abuse and adolescence (2012) Int J Child Health Hum Dev, 5 (2), pp. 149-179; Beaulieu, P., Boulanger, A., Desroches, J., Clark, A.J., Medical cannabis: Considerations for the anesthesiologist and pain physician (2016) Can J Anaesth, , Feb 5. [Epub ahead of print]; Yarnell, S., The use of medicinal marijuana for posttraumatic stress disorder: A review of the current literature (2015) Prim Care Companion CNS Disord, 17 (3); Volz, M.S., Siegmund, B., Häuser, W., [Efficacy, tolerability, and safety of cannabinoids in gastroenterology: A systematic review] (2016) Schmerz, 30 (1), pp. 37-46. , German; Koppel, B.S., Brust, J.C., Fife, T., Bronstein, J., Youssof, S., Gronseth, G., Gloss, D., Systematic review: Efficacy and safety of medical marijuana in selected neurologic disorders: Report of the Guideline Development Subcommittee of the American Academy of Neurology (2014) Neurology, 82 (17), pp. 1556-1563; Mücke, M., Carter, C., Cuhls, H., Prüß, M., Radbruch, L., Häuser, W., [Cannabinoids in palliative care : Systematic review and meta-analysis of efficacy, tolerability and safety] (2016) Schmerz, 30 (1), pp. 25-36. , German.

312. Greydanus DE, Hawver EK, Greydanus MM, Merrick J. Cannabis: Effective and safe analgesic? J Pain Manage. 2014;7(3):209-33.

313. Brast S, Bland E, Jones-Hooker C, Long M, Green K. Capnography for the Radiology and Imaging Nurse: A Primer. Journal of Radiology Nursing. 2016;35(3):173-90. doi: http://doi.org/10.1016/j.jradnu.2016.07.002.

314. Fairclough SH, Gilleade K, Ewing KC, Roberts J. Capturing user engagement via psychophysiology: Measures and mechanisms for biocybernetic adaptation. Int J Auton Adapt Commun Syst. 2013;6(1):63-79. doi: 10.1504/IJAACS.2013.050694.

315. Potse M, Dubé B, Vinet A. Cardiac anisotropy in boundary-element models for the electrocardiogram. Med Biol Eng Comput. 2009;47(7):719-29. doi: 10.1007/s11517-009-0472-x.

316. Tofield A. Cardiac arrhythmia challenge: a new App. Eur Heart J. 2013;34(44):3392.

317. Lima CS, Cardoso MJ, editors. Cardiac arrhythmia detection by parameters sharing and MMIE training of hidden markov models. 29th Annual International Conference of IEEE-EMBS, Engineering in Medicine and Biology Society, EMBC'07; 2007; Lyon.

318. Chizner MA. Cardiac Auscultation: Rediscovering the Lost Art. Curr Probl Cardiol. 2008;33(7):326-408. doi: 10.1016/j.cpcardiol.2008.03.003.

319. Al-Trad BA, Faris MAIE, Al-Smadi M, Bashir A, Mansi M, Alaraj M, et al. Cardiac autonomic dysfunction in young obese males is not associated with disturbances in pituitary-thyroid axis hormones. Eur Rev Med Pharmacol Sci. 2015;19(9):1689-95.

320. Tiller WA, McCraty R, Atkinson M. Cardiac coherence: A new, noninvasive measure of autonomic nervous system order. Altern Ther Health Med. 1996;2(1):52-65.

321. Earls JP, Leipsic J. Cardiac Computed Tomography Technology and Dose-reduction Strategies. Radiol Clin North Am. 2010;48(4):657-74. doi: 10.1016/j.rcl.2010.04.003.

322. Paterick TE, Jan MF, Paterick ZR, Umland MM, Kramer C, Lake P, et al. Cardiac Evaluation of Collegiate Student Athletes: A Medical and Legal Perspective. The American Journal of Medicine. 2012;125(8):742-52. doi: http://doi.org/10.1016/j.amjmed.2012.02.017.

323. Sahlén A, Rubulis A, Winter R, Jacobsen PH, Ståhlberg M, Tornvall P, et al. Cardiac fatigue in long-distance runners is associated with ventricular repolarization abnormalities. Heart Rhythm. 2009;6(4):512-9. doi: 10.1016/j.hrthm.2008.12.020.

324. Bardo DME, Brown P. Cardiac multidetector computed tomography: Basic physics of image acquisition and clinical applications. Curr Cardiol Rev. 2008;4(3):231-43. doi: 10.2174/157340308785160615.

325. Libonati JR. Cardiac remodeling and exercise training in hypertension. Curr Hypertens Rev. 2011;7(1):20-8. doi: 10.2174/157340211795909016.

326. Montgomery H. Cardiac reserve: Linking physiology and genetics. Intensive Care Med Suppl. 2000;26(1):S137-S44.

327. Jarmon RG. Cardiac telemetry exercise program. Journal of the American College of Emergency Physicians. 1977;6(2):50-2. doi: 10.1016/S0361-1124(77)80032-4.

328. Craven M, Newman S, Fletcher M, Silvera B, Coore D, Forbes N, et al., editors. Cardiac training simulator using pump with electronic pressure sensor to trigger ventricular fibrillation. Proceedings IEEE SoutheastCon 2003 "Bridging the Digital Divide"; 2003; Ocho Rios, St. Ann.

329. Vainoras A, Marozas V, Korsakas S, Gargasas L, Siupsinskas L, Miskinis V. Cardiological telemonitoring in rehabilitation and sports medicine. Stud Health Technol Informatics. 2004;105:121-30.

330. Gordon MS. Cardiology patient simulator. Development of an animated manikin to teach cardiovascular disease. Am J Cardiol. 1974;34(3):350-5. doi: 10.1016/0002-9149(74)90038-1.

331. Turdi S, Guo R, Huff AF, Wolf EM, Culver B, Ren J. Cardiomyocyte contractile dysfunction in the APPswe/PS1dE9 mouse model of Alzheimer's disease. PLoS One. 2009;4(6):e6033. Epub 2009/06/25. doi: 10.1371/journal.pone.0006033. PubMed PMID: 19551139; PubMed Central PMCID: PMCPMC2696039.

332. Myers J, Arena R, Dewey F, Bensimhon D, Abella J, Hsu L, et al. A cardiopulmonary exercise testing score for predicting outcomes in patients with heart failure. Am Heart J. 2008;156(6):1177-83. doi: 10.1016/j.ahj.2008.07.010.

333. Stringer WW. Cardiopulmonary exercise testing: Current applications. Expert Rev Respir Med. 2010;4(2):179-88. doi: 10.1586/ers.10.8.

334. Jones S, Elliott PM, Sharma S, McKenna WJ, Whipp BJ. Cardiopulmonary responses to exercise in patients with hypertrophic cardiomyopathy. Heart. 1998;80(1):60-7.

335. Gabbott D, Smith G, Mitchell S, Colquhoun M, Nolan J, Soar J, et al. Cardiopulmonary resuscitation standards for clinical practice and training in the UK. Accident and Emergency Nursing. 2005;13(3):171-9. doi: http://doi.org/10.1016/j.aaen.2005.04.004.

336. Jamšek J, Stefanovska A, editors. The cardiorespiratory couplings observed in the LDF signal using wavelet bispectrum. 29th Annual International Conference of IEEE-EMBS, Engineering in Medicine and Biology Society, EMBC'07; 2007; Lyon.

337. Lean T. Cardiothoracic intensive care introductory program. Australian Critical Care. 1999;12(2):80. doi: http://doi.org/10.1016/S1036-7314(99)70569-2.

338. Mogensen UM, Jensen T, Køber L, Kelbæk H, Mathiesen AS, Dixen U, et al. Cardiovascular autonomic neuropathy and subclinical cardiovascular disease in normoalbuminuric type 1 diabetic patients. Diabetes. 2012;61(7):1822-30. doi: 10.2337/db11-1235.

339. Koerner DR. Cardiovascular benefits from an industrial physical fitness program. J Occup Med. 1973;15(9):700-7.

340. Brown PP, Houser F, Kugelmass AD, Anderson AL, Tarkington LG, Simon AW, et al. Cardiovascular Centers of Excellence Program: A System Approach for Improving the Care and Outcomes of Cardiovascular Patients at HCA Hospitals. The Joint Commission Journal on Quality and Patient Safety. 2007;33(11):647-59. doi: http://doi.org/10.1016/S1553-7250(07)33074-2.

341. Poderys J, Grunovas A, Poderiene K, Buliuolis A, Šilinskas V, Trinkunas E. Cardiovascular changes during the performance by nonathletes of Bosco repeated jumps anaerobic test. Medicina. 2015;51(3):187-92. doi: 10.1016/j.medici.2015.06.003.

342. Al-Thani H, Shabana A, Hussein A, Sadek A, Sharaf A, Koshy V, et al. Cardiovascular complications in diabetic patients undergoing regular hemodialysis: A 5-Year observational study. Angiology. 2014;66(3):225-30. doi: 10.1177/0003319714523672.

343. Keates AK, Mocumbi AO, Ntsekhe M, Sliwa K, Stewart S. Cardiovascular disease in Africa: Epidemiological profile and challenges. Nat Rev Cardiol. 2017;14(5):273-93. doi: 10.1038/nrcardio.2017.19.

344. Pelliccia A, Quattrini FM, Squeo MR, Caselli S, Culasso F, Link MS, et al. Cardiovascular diseases in Paralympic athletes. Br J Sports Med. 2016;50(17):1075-80. doi: 10.1136/bjsports-2015-095867.

345. Atwal S, Porter J, MacDonald P. Cardiovascular effects of strenuous exercise in adult recreational hockey: The hockey heart study. CMAJ. 2002;166(3):303-7.

346. Murayama M, Kuroda Y. Cardiovascular future of athletes. Jpn J Phys Fitness Sports Med. 1980;29(2):117-23. doi: 10.7600/jspfsm1949.29.117.

347. Biederman RWW. Cardiovascular magnetic resonance imaging as applied to patients with pulmonary arterial hypertension. Int J Clin Pract. 2009;63(SUPPL. 162):20-35. doi: 10.1111/j.1742-1241.2009.02109.x.

348. Biglands JD, Radjenovic A, Ridgway JP. Cardiovascular magnetic resonance physics for clinicians: Part II. J Cardiovasc Magn Reson. 2012;14(1). doi: 10.1186/1532-429X-14-66.

349. Galanti G, Pizzi A, Lucarelli M, Stefani L, Gianassi M, Di Tante V, et al. The cardiovascular profile of soccer referees: An echocardiographic study. Cardiovasc Ultrasound. 2008;6. doi: 10.1186/1476-7120-6-8.

350. Duncan HW, Barnard RJ, Grimditch GK, Vinten-Johansen J, Buckberg GD. Cardiovascular response to sudden strenuous exercise. Basic Res Cardiol. 1987;82(3):226-32. doi: 10.1007/BF01906853.

351. Wegmann M, Steffen A, Pütz K, Würtz N, Such U, Faude O, et al. Cardiovascular risk and fitness in veteran football players. J SPORTS SCI. 2016;34(6):576-83. doi: 10.1080/02640414.2015.1118525.

352. Felici F, Rodio A, Madaffari A, Ercolani L, Marchetti M. The cardiovascular work of competitive dinghy sailing. J SPORTS MED PHYS FITNESS. 1999;39(4):309-14.

353. Lowenstein SR, Crescenzi CA, Kern DC, Steel K. Care of the elderly in the emergency department. Ann Emerg Med. 1986;15(5):528-35. doi: 10.1016/S0196-0644(86)80987-8.

354. Gatch G. Caring for children needing anesthesia. AORN Journal. 1982;35(2):218-26. doi: http://doi.org/10.1016/S0001-2092(07)68783-3.

355. Selamet Tierney ES, Gauvreau K, Jaff MR, Gal D, Nourse SE, Trevey S, et al. Carotid Artery Intima-Media Thickness Measurements in the Youth: Reproducibility and Technical Considerations. Journal of the American Society of Echocardiography. 2015;28(3):309-16. doi: http://doi.org/10.1016/j.echo.2014.10.004.

356. Gaitan BD, Ramakrishna H, DiNardo JA, Cannesson M. Case 1-2010 Pulmonary Thrombectomy in an Adult With Fontan Circulation. J Cardiothorac Vasc Anesth. 2010;24(1):173-82. doi: 10.1053/j.jvca.2009.11.003.

357. Sousa JP, Cabri J, Donaghy M. Case research in sports physiotherapy: A review of studies. Phys Ther Sport. 2007;8(4):197-206. doi: 10.1016/j.ptsp.2007.02.003.

358. Bouder F. A case study of long QT regulation: A regulatory tennis game across the Atlantic. J Risk Res. 2007;10(3):385-412. doi: 10.1080/13669870701270903.

359. Althoff KD, Bergmann R, Wess S, Manago M, Auriol E, Larichev OI, et al. Case-based reasoning for medical decision support tasks: The Inreca approach. Artif Intell Med. 1998;12(1):25-41. doi: 10.1016/S0933-3657(97)00038-9.

360. Bloice MD, Simonic KM, Holzinger A. Casebook: A virtual patient iPad application for teaching decision-making through the use of electronic health records. BMC Med Informatics Decis Mak. 2014;14(1). doi: 10.1186/1472-6947-14-66.

361. Chou KT, Yu SN, editors. Categorizing heartbeats by independent component analysis and support vector machines. 8th International Conference on Intelligent Systems Design and Applications, ISDA 2008; 2008; Kaohsiung.

362. Chen X, Ho CT, Lim ET, Kyaw TZ, editors. Cellular phone based online ECG processing for ambulatory and continuous detection. Computers in Cardiology 2007, CAR 2007; 2007; Durham, NC.

363. Tri JL, Severson RP, Firl AR, Hayes DL, Abenstein JP. Cellular telephone interference with medical equipment. Mayo Clin Proc. 2005;80(10):1286-90.

364. Rengier F, Weber TF, Giesel FL, Böckler D, Kauczor HU, Von Tengg-Kobligk H. Centerline analysis of aortic CT angiographic examinations: Benefits and limitations. Am J Roentgenol. 2009;192(5):W255-W63. doi: 10.2214/AJR.08.1488.

365. Illies T, Säring D, Kinoshita M, Fujinaka T, Bester M, Fiehler J, et al. Cerebral aneurysm pulsation: Do iterative reconstruction methods improve measurement accuracy in vivo? Am J Neuroradiol. 2014;35(11):2159-63. doi: 10.3174/ajnr.A4000.

366. De Jesus CR, García Peña BM, Lozano JM, Maniaci V. Cervical Spine Motion during Airway Management Using Two Manual In-line Immobilization Techniques: A Human Simulator Model Study. Pediatr Emerg Care. 2015;31(9):627-32. doi: 10.1097/PEC.0000000000000245.

367. CEU Test. Journal of Emergency Nursing. 1999;25(5):432-6. doi: http://doi.org/10.1016/S0099-1767(99)70108-X.

368. Moghaddam RF, Moghaddam FF, Cheriet M, Dandres T, Samson R, Lemieux Y, editors. Challenges and complexities in application of LCA approaches in the case of ICT for a sustainable future. 2nd International Conference on ICT for Sustainability, ICT4S 2014; 2014: Atlantis Press.

369. Nielsen T, Paquette T, Solomonova E, Lara-Carrasco J, Colombo R, Lanfranchi P. Changes in cardiac variability after REM sleep deprivation in recurrent nightmares. Sleep. 2010;33(1):113-22.

370. Oinuma M, Hirayanagi K, Yajima K, Igarashi M, Arakawa Y. Changes in cardio-respiratory function, heart rate variability, and electrogastrogram preceding motion sickness-like symptoms induced by virtual reality stimulus. Jpn J Aerosp Environ Med. 2004;41(3):99-109.

371. Veerabhadrappa ST, Vyas AL, Anand S. Changes in heart rate variability and pulse wave characteristics during normal pregnancy and postpartum. Int J Biomed Eng Technol. 2015;17(2):99-114. doi: 10.1504/IJBET.2015.068045.

372. Ghobadi S, Rahati S, Golmakani A, Fadardi JS, editors. The chaotic behavior of EEG signals during Reiki. 2011 19th Iranian Conference on Electrical Engineering, ICEE 2011; 2011; Tehran.

373. Boey RA, Wuyts FL, Van de Heyning PH, De Bodt MS, Heylen L. Characteristics of stuttering-like disfluencies in Dutch-speaking children. Journal of Fluency Disorders. 2007;32(4):310-29. doi: http://doi.org/10.1016/j.jfludis.2007.07.003.

374. Wahed MA. Characterization of ECG signals based on Zernike moments and moment invariants. J Eng Appl Sci. 2007;54(2):205-21.

375. Barold SS, Cantens F. Characterization of the 16 blanking periods of the Medtronic GEM DR dual chamber defibrillators. J Intervent Card Electrophysiol. 2001;5(3):319-25. doi: 10.1023/A:1011477002235.

376. Coia LR. Chemoradiation: A superior alternative for the primary management of esophageal carcinoma. Seminars in Radiation Oncology. 1994;4(3):157-64. doi: http://doi.org/10.1016/S1053-4296(05)80063-3.

377. Castellanos A, Myerburg RJ. The Chicago school of arrhythmology: Revisited. Card Electrophysiol Rev. 2003;7(1):96-8. doi: 10.1023/A:1023615828855.

378. Coussement K, Van den Poel D. Churn prediction in subscription services: An application of support vector machines while comparing two parameter-selection techniques. Expert Sys Appl. 2008;34(1):313-27. doi: 10.1016/j.eswa.2006.09.038.

379. Rabe-Hesketh S, Skrondal A. Classical latent variable models for medical research. Stat Methods Med Res. 2008;17(1):5-32. doi: 10.1177/0962280207081236.

380. Içer S, Gengeç Ş. Classification and analysis of non-stationary characteristics of crackle and rhonchus lung adventitious sounds. Digital Signal Process Rev J. 2014;28(1):18-27. doi: 10.1016/j.dsp.2014.02.001.

381. Dobrowolski A, Suchocki M, Tomczykiewicz K, Majda-Zdancewicz E. Classification of auditory brainstem response using wavelet decomposition and SVM network. Biocybern Biomed Eng. 2016;36(2):427-36. doi: 10.1016/j.bbe.2016.01.003.

382. Sotto LFDP, Coelho RC, De Melo VV, editors. Classification of cardiac arrhythmia by random forests with features constructed by Kaizen programming with linear genetic programming. 2016 Genetic and Evolutionary Computation Conference, GECCO 2016; 2016: Association for Computing Machinery, Inc.

383. Kumari VSR, Kumar PR. Classification of cardiac arrhythmia using hybrid genetic algorithm optimisation for multi-layer perceptron neural network. Int J Biomed Eng Technol. 2016;20(2):132-49. doi: 10.1504/IJBET.2016.074199.

384. Subasi A, Erçelebi E. Classification of EEG signals using neural network and logistic regression. COMPUT METHODS PROGRAMS BIOMED. 2005;78(2):87-99. doi: 10.1016/j.cmpb.2004.10.009.

385. Khazaee A, Ebrahimzadeh A. Classification of electrocardiogram signals with support vector machines and genetic algorithms using power spectral features. Biomed Signal Process Control. 2010;5(4):252-63. doi: 10.1016/j.bspc.2010.07.006.

386. Subasi A. Classification of EMG signals using PSO optimized SVM for diagnosis of neuromuscular disorders. Comput Biol Med. 2013;43(5):576-86. doi: 10.1016/j.compbiomed.2013.01.020.

387. Jahidin AH, Megat Ali MSA, Taib MN, Tahir N, Yassin IM, Lias S. Classification of intelligence quotient via brainwave sub-band power ratio features and artificial neural network. COMPUT METHODS PROGRAMS BIOMED. 2014;114(1):50-9. doi: 10.1016/j.cmpb.2014.01.016.

388. Moslem B, Diab MO, Marque C, Khalil M, editors. Classification of multichannel uterine EMG signals. 33rd Annual International Conference of the IEEE Engineering in Medicine and Biology Society, EMBS 2011; 2011; Boston, MA.

389. Kadous MW, Sammut C. Classification of multivariate time series and structured data using constructive induction. Mach Learn. 2005;58(2-3):179-216. doi: 10.1007/s10994-005-5826-5.

390. Gubbi J, Khandoker A, Palaniswami M, editors. Classification of obstructive and central sleep apnea using wavelet packet analysis of ECG signals. 36th Annual Conference of Computers in Cardiology, CinC 2009; 2009; Park City, UT.

391. Wieben O, Afonso VX, Tompkins WJ. Classification of premature ventricular complexes using filter bank features, induction of decision trees and a fuzzy rule-based system. Med Biol Eng Comput. 1999;37(5):560-5.

392. Gubbi J, Khandoker A, Palaniswami M. Classification of sleep apnea types using wavelet packet analysis of short-term ECG signals. J Clin Monit Comput. 2012;26(1):1-11. doi: 10.1007/s10877-011-9323-z.

393. Zadeh AE, Khazaee A, Ranaee V. Classification of the electrocardiogram signals using supervised classifiers and efficient features. COMPUT METHODS PROGRAMS BIOMED. 2010;99(2):179-94. doi: 10.1016/j.cmpb.2010.04.013

10.1016/j.na.2008.10.015Osowski, S., Linh, T.H., ECG beat recognition using fuzzy hybrid neural network (2001) IEEE Trans. Biomed. Eng., 48, pp. 1265-1271; Chazal, P., O'Dwyer, M., Reilly, R.B., Automatic classification of heartbeats using ECG morphology and heartbeat interval features (2004) IEEE Trans. Biomed. Eng., 51, pp. 1196-1206; Lagerholm, M., Clustering ECG complexes using Hermite functions and self-organizing maps (2000) IEEE Trans. Biomed. Eng., 47, pp. 839-847; Khadra, L., Al-Fahoum, A.S., Binajjaj, S., A quantitative analysis approach for cardiac arrhythmia classification using higher order spectral techniques (2005) IEEE Trans. Biomed. Eng., 52, pp. 1840-1845. , November; Andreao, R.V., Dorizzi, B., Boudy, J., ECG signal analysis through hidden Markov models (2006) IEEE Trans. Biomed. Eng., 53, pp. 1541-1549. , August; Mitra, S., Mitra, M., Chaudhuri, B.B., A rough set-based inference engine for ECG classification (2006) IEEE Trans. Instrum. Meas., 55, pp. 2198-2206. , December; de Chazal, F., Reilly, R.B., A patient adapting heart beat classifier using ECG morphology and heartbeat interval features (2006) IEEE Trans. Biomed. Eng., 53, pp. 2535-2543. , December; Kania, M., Fereniec, M., Maniewski, R., (2007), Wavelet denoising for multi-lead high resolution ECG signals, Measur. Sci. Rev. 7 (Section 2, no. 4)Donoho, D., Johnstone, I., Adapting to unknown smoothness via wavelet shrinkage (1995) J. ASA, 90, pp. 1200-1223; Donoho, D., De-noising by soft-thresholding (1995) IEEE Trans. Inform. Theory, 41, pp. 613-627; Al-Alaoui, M.A., A unified analog and digital design to peak and valley detector, window peak and valley detectors, and zero crossing detectors (1986) IEEE Trans. Instrum. Meas., 35, pp. 304-307; Haykin, S., (1999) Neural Networks: A Comprehensive Foundation, , Prentice-Hall, NJ, USA; Rumelhart, D.E., McClelland, J.L., (1986) Parallel Distributed Processing: Explorations in the Microstructure of Cognition, , MIT Press, Cambridge, MA; Riedmiller, M., Braun, H., (1993), pp. 586-591. , A direct adaptive method for faster backpropagation learning: the rprop algorithm, Proc. I CN NBattiti, R., First and second order methods for learning (1992) Neural Comput., 4, pp. 141-166; Hagan, M.T., Menhaj, M., Training feed-forward networks with the Marquardt algorithm (1994) IEEE Trans. Neural Networks, 5 (6), pp. 989-993; Specht, D.F., Probabilistic neural networks (1990) Neural Networks, 3, pp. 109-118; Burges, C., A tutorial on support vector machines for pattern recognition (1998) Data Mining Knowledge Discov., 2, pp. 121-167; Mark, R.G., Moody, G.B., http://ecg.mit.edu/dbinfo.html, MIT-BIH Arrhythmia Database 1997 [online]. Available at: Moody, G.B., Mark, R.G., The impact of the MIT/BIH arrhythmia database (2001) IEEE Eng. Med. Biol. Mag., 20 (3), pp. 45-50. , May-June.

394. Al-Mardini M, Aloul F, Sagahyroon A, Al-Husseini L. Classifying obstructive sleep apnea using smartphones. J Biomed Informatics. 2014;52:251-9. doi: 10.1016/j.jbi.2014.07.004.

395. Duff WR, Mannes JH, Breitmeyer MO. Clinical device note: a versatile ECG simulator for laboratory and teaching applications. Med Instrum. 1973;7(4):235-6. Epub 1973/09/01. PubMed PMID: 4746838.

396. Hollis C, Pennant M, Cuenca J, Glazebrook C, Kendall T, Whittington C, et al. Clinical effectiveness and patient perspectives of different treatment strategies for tics in children and adolescents with tourette syndrome: A systematic review and qualitative analysis. Health Technol Assess. 2016;20(4):1-289, xiii-xxxvii. doi: 10.3310/hta20040.

397. Aldridge MD, Waddington WW, Dickson JC, Prakash V, Ell PJ, Bomanji JB. Clinical evaluation of reducing acquisition time on single-photon emission computed tomography image quality using proprietary resolution recovery software. Nucl Med Commun. 2013;34(11):1116-23. doi: 10.1097/MNM.0b013e3283658328.

398. Frykholm P, Pikwer A, Hammarskjöld F, Larsson AT, Lindgren S, Lindwall R, et al. Clinical guidelines on central venous catheterisation. Acta Anaesthesiol Scand. 2014;58(5):508-24. doi: 10.1111/aas.12295.

399. Evans SM, Murray A, Patrick I, Fitzgerald M, Smith S, Cameron P. Clinical handover in the trauma setting: A qualitative study of paramedics and trauma team members. Qual Saf Health Care. 2010;19(6). doi: 10.1136/qshc.2009.039073.

400. Harbison J. Clinical judgement in the interpretation of evidence: A Bayesian approach. J Clin Nurs. 2006;15(12):1489-97. doi: 10.1111/j.1365-2702.2005.01487.x.

401. Flavell EM, Stacey MR, Hall JE. The clinical management of airway obstruction. Current Anaesthesia & Critical Care. 2009;20(3):102-12. doi: http://doi.org/10.1016/j.cacc.2009.02.004.

402. Lewis CK. The clinical nurse specialist's role as coach in a clinical practice development model. Journal of Vascular Nursing. 1996;14(2):48-52. doi: http://doi.org/10.1016/S1062-0303(96)80016-1.

403. Calder S. Clinical Pearls and Pitfalls of Electrocardiogram Interpretation in Acute Myocardial Infarction. Journal of Emergency Nursing. 2008;34(4):324-9. doi: 10.1016/j.jen.2007.08.003.

404. Bain CA, Bucknall T, Weir-Phyland J, editors. A clinical quality feedback loop supported by mobile point-of-care (POC) data collection. 3rd International Workshop on Information Management for Mobile Applications, IMMoA 2013 - In Conjunction with VLDB 2013; 2013: CEUR-WS.

405. Califf RM, Mehta RH, Peterson ED. Clinical Quality in Non–ST-Elevation Acute Coronary Syndromes. The American Journal of Medicine. 2007;120(11):930-5. doi: http://doi.org/10.1016/j.amjmed.2006.10.016.

406. Stayt LC. Clinical simulation: A sine qua non of nurse education or a white elephant? Nurse Education Today. 2012;32(5):e23-e7. doi: http://doi.org/10.1016/j.nedt.2011.06.003.

407. Ogden PE, Cobbs LS, Howell MR, Sibbitt SJB, DiPette DJ. Clinical Simulation: Importance to the Internal Medicine Educational Mission. The American Journal of Medicine. 2007;120(9):820-4. doi: http://doi.org/10.1016/j.amjmed.2007.06.017.

408. Tolsgaard MG. Clinical skills training in undergraduate medical education using a student-centered approach. Dan Med J. 2013;60(8):14.

409. Childs JC, Sepples S. Clinical teaching by simulation: Lessons learned from a complex patient care scenario. Nurs Educ Persp. 2006;27(3):154-8.

410. Brohet C. Clinical value of vectorcardiography, Holter monitoring and quantitative electrocardiology. Acta Cardiol. 2000;55(3):157-62.

411. Gilroy J, Meyer JS, Bauer RB, Vulpe M, Greenwood D. Clinical, biochemical and neurophysiological studies of chronic interstitial hypertrophic polyneuropathy. The American Journal of Medicine. 1966;40(3):368-83. doi: http://doi.org/10.1016/0002-9343(66)90132-X.

412. Auricchio F, Conti M, Ferrara A, Lanzarone E. A clinically applicable stochastic approach for noninvasive estimation of aortic stiffness using computed tomography data. IEEE TRANS BIOMED ENG. 2015;62(1):176-87. doi: 10.1109/TBME.2014.2343673.

413. Kelly N, Esteve R, Papadimos TJ, Sharpe RP, Keeney SA, DeQuevedo R, et al. Clinician-performed ultrasound in hemodynamic and cardiac assessment: a synopsis of current indications and limitations. Eur J Trauma Emerg Surg. 2015;41(5):469-80. doi: 10.1007/s00068-014-0492-6.

414. Balady GJ, Arena R, Sietsema K, Myers J, Coke L, Fletcher GF, et al. Clinician's guide to cardiopulmonary exercise testing in adults: A scientific statement from the American heart association. Circulation. 2010;122(2):191-225. doi: 10.1161/CIR.0b013e3181e52e69

10.1016/j.ijcard.2008. 12.143 Accessed May 27; Guazzi, M., Myers, J., Arena, R., Cardiopulmonary exercise testing in the clinical and prognostic assessment of diastolic heart failure (2005) Journal of the American College of Cardiology, 46 (10), pp. 1883-1890. , DOI 10.1016/j.jacc.2005.07.051, PII S0735109705019984; Moore, B., Brubaker, P.H., Stewart, K.P., Kitzman, D.W., VE/VCO2 slope in older heart failure patients with normal versus reduced ejection fraction compared with age-matched healthy controls (2007) J Card Fail, 13, pp. 259-262; Arena, R., Owens, D.S., Arevalo, J., Smith, K., Mohiddin, S.A., McAreavey, D., Ulisney, K.L., Plehn, J.F., Ventilatory efficiency and resting hemodynamics in hypertrophic cardiomyopathy (2008) Med Sci Sports Exerc, 40, pp. 799-805; Guazzi, M., Myers, J., Peberdy, M.A., Bensimhon, D., Chase, P., Arena, R., Exercise oscillatory breathing in diastolic heart failure: Prevalence and prognostic insights (2008) Eur Heart J, 29, pp. 2751-2759; Waraich, S., Sietsema, K.E., Clinical cardiopulmonary exercise testing: Patient and referral characteristics (2007) J Cardiopulm Rehabil Prev, 27, pp. 400-406; Janicki, J.S., Weber, K.T., Likoff, M.J., Fishman, A.P., Exercise testing to evaluate patients with pulmonary vascular disease (1984) Am Rev Respir Dis, 129, pp. S93-S95; Martinez, F.J., Stanopoulos, I., Acero, R., Becker, F.S., Pickering, R., Beamis, J.F., Graded comprehensive cardiopulmonary exercise testing in the evaluation of dyspnea unexplained by routine evaluation (1994) Chest, 105, pp. 168-174; Depaso, W.J., Winterbauer, R.H., Lusk, J.A., Dreis, D.F., Springmeyer, S.C., Chronic dyspnea unexplained by history, physical examination, chest roentgenogram, and spirometry: Analysis of a seven-year experience (1991) Chest, 100, pp. 1293-1299; Pratter, M.R., Curley, F.J., Dubois, J., Irwin, R.S., Cause and evaluation of chronic dyspnea in a pulmonary disease clinic (1989) Arch Intern Med, 149, pp. 2277-2282; Wasserman, K., Hansen, J.E., Sue, D.Y., Stringer, W.W., Whipp, B.J., (2004) Principles of Exercise Testing and Interpretation: Including Pathophysiology and Clinical Applications, , 4th ed. Philadelphia Pa: Lippincott Williams & Wilkins; Weisman, I.M., Zeballos, R.J., An integrated approach to the interpretation of cardiopulmonary exercise testing (1994) Clin Chest Med, 15, pp. 421-445; Palange, P., Carlone, S., Forte, S., Galassetti, P., Serra, P., Cardiopulmonary exercise testing in the evaluation of patients with ventilatory vs circulatory causes of reduced exercise tolerance (1994) Chest, 105, pp. 1122-1126; Palange, P., Ward, S.A., Carlsen, K.-H., Casaburi, R., Gallagher, C.G., Gosselink, R., O'Donnell, D.E., Whipp, B.J., Recommendations on the use of exercise testing in clinical practice (2007) European Respiratory Journal, 29 (1), pp. 185-209. , DOI 10.1183/09031936.00046906; Hansen, J.E., Sue, D.Y., Oren, A., Wasserman, K., Relation of oxygen uptake to work rate in normal men and men with circulatory disorders (1987) Am J Cardiol, 59, pp. 669-674; Haller, R.G., Lewis, S.F., Pathophysiology of exercise performance in muscle disease (1984) Med Sci Sports Exerc, 16, pp. 456-459; Flaherty, K.R., Wald, J., Weisman, I.M., Zeballos, R.J., Schork, M.A., Blaivas, M., Rubenfire, M., Martinez, F.J., Unexplained exertional limitation: Characterization of patients with a mitochondrial myopathy (2001) Am J Respir Crit Care Med, 164, pp. 425-432; Tanabe, Y., Nakagawa, I., Ito, E., Suzuki, K., Hemodynamic basis of the reduced oxygen uptake relative to work rate during incremental exercise in patients with chronic heart failure (2002) Int J Cardiol, 83, pp. 57-62; Duscha, B.D., Kraus, W.E., Keteyian, S.J., Sullivan, M.J., Green, H.J., Schachat, F.H., Pippen, A.M., Annex, B.H., Capillary density of skeletal muscle: A contributing mechanism for exercise intolerance in class II-III chronic heart failure independent of other peripheral alterations (1999) J Am Coll Cardiol, 33, pp. 1956-1963; Hambrecht, R., Fiehn, E., Yu, J., Niebauer, J., Weigl, C., Hilbrich, L., Adams, V., Schuler, G., Effects of endurance training on mitochondrial ultra-structure and fiber type distribution in skeletal muscle of patients with stable chronic heart failure (1997) J Am Coll Cardiol, 29, pp. 1067-1073; Mettauer, B., Zoll, J., Garnier, A., Ventura-Clapier, R., Heart failure: A model of cardiac and skeletal muscle energetic failure (2006) Pflugers Arch, 452, pp. 653-666; Sullivan, M.J., Knight, J.D., Higginbotham, M.B., Cobb, F.R., Relation between central and peripheral hemodynamics during exercise in patients with chronic heart failure: Muscle blood flow is reduced with maintenance of arterial perfusion pressure (1989) Circulation, 80, pp. 769-781; Papazachou, O., Anastasiou-Nana, M., Sakellariou, D., Tassiou, A., Dimopoulos, S., Venetsanakos, J., Maroulidis, G., Nanas, S., Pulmonary function at peak exercise in patients with chronic heart failure (2007) International Journal of Cardiology, 118 (1), pp. 28-35. , DOI 10.1016/j.ijcard.2006.04.091, PII S0167527306006103; Agostoni, P., Bussotti, M., Cattadori, G., Margutti, E., Contini, M., Muratori, M., Marenzi, G., Fiorentini, C., Gas diffusion and alveolar-capillary unit in chronic heart failure (2006) Eur Heart J, 27, pp. 2538-2543; Marin-García, J., Goldenthal, M.J., Moe, G.W., Abnormal cardiac and skeletal muscle mitochondrial function in pacing-induced cardiac failure (2001) Cardiovasc Res, 52, pp. 103-110; Duscha, B.D., Schulze, P.C., Robbins, J.L., Forman, D.E., Implications of chronic heart failure on peripheral vasculature and skeletal muscle before and after exercise training (2008) Heart Fail Rev, 13, pp. 21-37; Gielen, S., Adams, V., Möbius-Winkler, S., Linke, A., Erbs, S., Yu, J., Kempf, W., Hambrecht, R., Anti-inflammatory effects of exercise training in the skeletal muscle of patients with chronic heart failure (2003) J Am Coll Cardiol, 42, pp. 861-868; Siciliano, G., Volpi, L., Piazza, S., Ricci, G., Mancuso, M., Murri, L., Functional diagnostics in mitochondrial diseases (2007) Biosci Rep, 27, pp. 53-67; Jeppesen, T.D., Schwartz, M., Olsen, D.B., Vissing, J., Oxidative capacity correlates with muscle mutation load in mitochondrial myopathy (2003) Ann Neurol, 54, pp. 86-92; Taivassalo, T., Jensen, T.D., Kennaway, N., Dimauro, S., Vissing, J., Haller, R.G., The spectrum of exercise tolerance in mitochondrial myopathies: A study of 40 patients (2003) Brain, 126, pp. 413-423; Taivassalo, T., Haller, R.G., Exercise and training in mitochondrial myop-athies (2005) Med Sci Sports Exerc, 37, pp. 2094-2101; Arena, R., Myers, J., Guazzi, M., The clinical significance of aerobic exercise testing and prescription: From apparently healthy to confirmed cardiovascular disease (2008) Am J Lifestyle Med, 2, pp. 519-536; MacKo, R.F., Ivey, F.M., Forrester, L.W., Task-oriented aerobic exercise in chronic hemiparetic stroke: Training protocols and treatment effects (2005) Top Stroke Rehabil, 12, pp. 45-57; MacKo, R.F., Benvenuti, F., Stanhope, S., MacEllari, V., Taviani, A., Nesi, B., Weinrich, M., Stuart, M., Adaptive physical activity improves mobility function and quality of life in chronic hemiparesis (2008) J Rehabil Res Dev, 45, pp. 323-328; Ivey, F.M., MacKo, R.F., Prevention of deconditioning after stroke (2009) Stroke Recovery and Rehabilitation Textbook, pp. 387-404. , Stein J, Harvey RL, Macko RF, Winstein CJ, Zorowitz RD, eds New York, NY: Demos Medical; Fletcher, B.J., Dunbar, S.B., Felner, J.M., Jensen, B.E., Almon, L., Cotsonis, G., Fletcher, G.F., Exercise testing and training in physically disabled men with clinical evidence of coronary artery disease (1994) Am J Cardiol, 73, pp. 170-174; Luft, A.R., MacKo, R.F., Forrester, L.W., Villagra, F., Ivey, F., Sorkin, J.D., Whitall, J., Hanley, D.F., Treadmill exercise activates subcortical neural networks and improves walking after stroke: A randomized controlled trial (2008) Stroke, 39, pp. 3341-3350; Ivey, F.M., Ryan, A.S., Hafer-Macko, C.E., Goldberg, A.P., MacKo, R.F., Treadmill aerobic training improves glucose tolerance and indices of insulin sensitivity in disabled stroke survivors: A preliminary report (2007) Stroke, 38, pp. 2752-2758; The pulmonary system (2007) Guides to the Evaluation of Permanent Impairment, , American Medical Association. 6th ed. Chicago Ill: American Medical Association; Oren, A., Sue, D.Y., Hansen, J.E., Torrance, D.J., Wasserman, K., The role of exercise testing in impairment evaluation (1987) Am Rev Respir Dis, 135, pp. 230-235; Agostoni, P., Smith, D.D., Schoene, R.B., Robertson, H.T., Butler, J., Evaluation of breathlessness in asbestos workers: Results of exercise testing (1987) Am Rev Respir Dis, 135, pp. 812-816; Fredriksen, P.M., Veldtman, G., Hechter, S., Therrien, J., Chen, A., Warsi, M.A., Freeman, M., Webb, G., Aerobic capacity in adults with various congenital heart diseases (2001) Am J Cardiol, 87, pp. 310-314; Dimopoulos, K., Okonko, D.O., Diller, G.-P., Broberg, C.S., Salukhe, T.V., Babu-Narayan, S.V., Li, W., Gatzoulis, M.A., Abnormal ventilatory response to exercise in adults with congenital heart disease relates to cyanosis and predicts survival (2006) Circulation, 113 (24), pp. 2796-2802. , DOI 10.1161/CIRCULATIONAHA.105.594218, PII 0000301720060620000006; Diller, G.P., Dimopoulos, K., Okonko, D., Li, W., Babu-Narayan, S.V., Broberg, C.S., Johansson, B., Gatzoulis, M.A., Exercise intolerance in adult congenital heart disease: Comparative severity, correlates, and prognostic implication (2005) Circulation, 112, pp. 828-835; Gratz, A., Hess, J., Hager, A., Self-estimated physical functioning poorly predicts actual exercise capacity in adolescents and adults with congenital heart disease (2009) Eur Heart J, 30, pp. 497-504; Giardini, A., Specchia, S., Berton, E., Sangiorgi, D., Coutsoumbas, G., Gargiulo, G., Oppido, G., Picchio, F.M., Strong and independent prognostic value of peak circulatory power in adults with congenital heart disease (2007) Am Heart J, 154, pp. 441-447; Colice, G.L., Shafazand, S., Griffin, J.P., Keenan, R., Bolliger, C.T., Physiologic evaluation of the patient with lung cancer being considered for resectional surgery: ACCP evidenced-based clinical practice guidelines (2nd edition) (2007) Chest, 132 (3 SUPPL.), pp. 161S-177S. , DOI 10.1378/chest.07-1359; Loewen, G.M., Watson, D., Kohman, L., Herndon Je, I.I., Shennib, H., Kernstine, K., Olak, J., Green, M., Preoperative exercise VO2 measurement for lung resection candidates: Results of Cancer and Leukemia Group B Protocol 9238 (2007) J Thorac Oncol, 2, pp. 619-625. , Cancer and Leukemia Group B; DeCamp Jr., M.M., Lipson, D., Krasna, M., Minai, O.A., McKenna Jr., R.J., Thomashow, B.M., The evaluation and preparation of the patient for lung volume reduction surgery (2008) Proceedings of the American Thoracic Society, 5 (4), pp. 427-431. , http://pats.atsjournals.org/cgi/reprint/5/4/427, DOI 10.1513/pats.200707-087ET; Fishman, A., Martinez, F., Naunheim, K., Piantadosi, S., Wise, R., Ries, A., Weinmann, G., Wood, D.E., A randomized trial comparing lung-volume-reduction surgery with medical therapy for severe emphysema (2003) N Engl J Med, 348, pp. 2059-2073. , National Emphysema Treatment Trial Research Group; Rich, S., Rabinovitch, M., Diagnosis and treatment of secondary (non-category 1) pulmonary hypertension (2008) Circulation, 118, pp. 2190-2199; Sun, X.G., Hansen, J.E., Oudiz, R.J., Wasserman, K., Gas exchange detection of exercise-induced right-to-left shunt in patients with primary pulmonary hypertension (2002) Circulation, 105, pp. 54-60; Ting, H., Sun, X.G., Chuang, M.L., Lewis, D.A., Hansen, J.E., Wasserman, K., A noninvasive assessment of pulmonary perfusion abnormality in patients with primary pulmonary hypertension (2001) Chest, 119, pp. 824-832; Miyamoto, S., Nagaya, N., Satoh, T., Kyotani, S., Sakamaki, F., Fujita, M., Nakanishi, N., Miyatake, K., Clinical correlates and prognostic significance of six-minute walk test in patients with primary pulmonary hypertension: Comparison with cardiopulmonary exercise testing (2000) Am J Respir Crit Care Med, 161, pp. 487-492; Kavanagh, T., Mertens, D.J., Hamm, L.F., Beyene, J., Kennedy, J., Corey, P., Shephard, R.J., Prediction of long-term prognosis in 12 169 men referred for cardiac rehabilitation (2002) Circulation, 106, pp. 666-671; Kavanagh, T., Mertens, D.J., Hamm, L.F., Beyene, J., Kennedy, J., Corey, P., Shephard, R.J., Peak oxygen intake and cardiac mortality in women referred for cardiac rehabilitation (2003) J Am Coll Cardiol, 42, pp. 2139-2143; Chaundhry, S., Arena, R., Wasserman, K., Hansen, J.E., Lewis, G.D., Myers, J., Chronos, N., Boden, W.E., Exercise-induced myocardial ischemia detected by cardiopulmonary exercise testing (2009) Am Heart J, 103, pp. 615-619; Belardinelli, R., Lacalaprice, F., Carle, F., Minnucci, A., Cianci, G., Perna, G., D'Eusanio, G., Exercise-induced myocardial ischaemia detected by car-diopulmonary exercise testing (2003) Eur Heart J, 24, pp. 1304-1313; Bussotti, M., Apostolo, A., Andreini, D., Palermo, P., Contini, M., Agostoni, P., Cardiopulmonary evidence of exercise-induced silent ischaemia (2006) Eur J Cardiovasc Prev Rehabil, 13, pp. 249-253; Greco, E.M., Guardini, S., Ferrario, M., Romano, S., How to program rate responsive pacemakers (2000) Pacing Clin Electrophysiol, 23, pp. 165-173; Duru, F., Cho, Y., Wilkoff, B.L., Cole, C.R., Adler, S., Jensen, D.N., Strobel, U., Candinas, R., Rate responsive pacing using transthoracic impedance minute ventilation sensors: A multicenter study on calibration stability (2002) Pacing Clin Electrophysiol, 25, pp. 1679-1684; Capucci, A., Boriani, G., Specchia, S., Marinelli, M., Santarelli, A., Magnani, B., Evaluation by cardiopulmonary exercise test of DDDR versus DDD pacing (1992) Pacing Clin Electrophysiol, 15, pp. 1908-1913; Lemke, B., Dryander, S.V., Jäger, D., MacHraoui, A., MacCarter, D., Barmeyer, J., Aerobic capacity in rate modulated pacing (1992) Pacing Clin Electrophysiol, 15, pp. 1914-1918; Lewalter, T., Rickli, H., MacCarter, D., Schwartze, P., Schimpf, R., Schumacher, B., Jung, W., Lüderitz, B., Oxygen uptake to work rate relation throughout peak exercise in normal subjects: Relevance for rate adaptive pacemaker programming (1999) Pacing Clin Electrophysiol, 22, pp. 769-775; Mathony, U., Schmidt, H., Gröger, C., Francis, D.P., Konzag, I., Müller-Werdan, U., Werdan, K., Syska, J., Optimal maximum tracking rate of dual-chamber pacemakers required by children and young adults for a maximal cardiorespiratory performance (2005) Pacing Clin Electrophysiol, 28, pp. 378-383; Alt, E.U., Schlegl, M.J., Matula, M.M., Intrinsic heart rate response as a predictor of rate-adaptive pacing benefit (1995) Chest, 107, pp. 925-930; Meine, M., Achtelik, M., Hexamer, M., Kloppe, A., Werner, J., Trappe, H.J., Assessment of the chronotropic response at the anaerobic threshold: An objective measure of chronotropic function (2000) Pacing Clin Electrophysiol, 23, pp. 1457-1467; Page, E., Defaye, P., Bonnet, J.L., Durand, C., Amblard, A., Comparison of the cardiopulmonary response to exercise in recipients of dual sensor DDDR pacemakers versus a healthy control group (2003) Pacing Clin Electrophysiol, 26, pp. 239-243; Madaric, J., Vanderheyden, M., Van Laethem, C., Verhamme, K., Feys, A., Goethals, M., Verstreken, S., Bartunek, J., Early and late effects of cardiac resynchronization therapy on exercise-induced mitral regurgitation: Relationship with left ventricular dyssynchrony, remodelling and cardiopulmonary performance (2007) Eur Heart J, 28, pp. 2134-2141; Strickberger, S.A., Conti, J., Daoud, E.G., Havranek, E., Mehra, M.R., Piña, I.L., Young, J., Patient selection for cardiac resynchronization therapy: From the Council on Clinical Cardiology Subcommittee on Electrocardiogra-phy and Arrhythmias and the Quality of Care and Outcomes Research Interdisciplinary Working Group, in collaboration with the Heart Rhythm Society (2005) Circulation, 111, pp. 2146-2150; Pardaens, K., Van Cleemput, J., Vanhaecke, J., Fagard, R.H., Atrial fibrillation is associated with a lower exercise capacity in male chronic heart failure patients (1997) Heart, 78, pp. 564-568; Agostoni, P., Emdin, M., Corrà, U., Veglia, F., Magrì, D., Tedesco, C.C., Berton, E., Guazzi, M., Permanent atrial fibrillation affects exercise capacity in chronic heart failure patients (2008) Eur Heart J, 29, pp. 2367-2372; Guazzi, M., Belletti, S., Bianco, E., Lenatti, L., Guazzi, M.D., Endothelial dysfunction and exercise performance in lone atrial fibrillation or associated with hypertension or diabetes: Different results with cardio-version (2006) Am J Physiol Heart Circ Physiol, 291, pp. H921-H928; Guazzi, M., Belletti, S., Tumminello, G., Fiorentini, C., Guazzi, M.D., Exercise hyperventilation, dyspnea sensation, and ergoreflex activation in lone atrial fibrillation (2004) Am J Physiol Heart Circ Physiol, 287, pp. H2899-H2905; Wozakowska-Kaplon, B., Opolski, G., Effects of sinus rhythm restoration in patients with persistent atrial fibrillation: A clinical, echocardiographic and hormonal study (2004) Int J Cardiol, 96, pp. 171-176; Lok, N.S., Lau, C.P., Oxygen uptake kinetics and cardiopulmonary performance in lone atrial fibrillation and the effects of sotalol (1997) Chest, 111, pp. 934-940; McCullough, P.A., Gallagher, M.J., Dejong, A.T., Sandberg, K.R., Trivax, J.E., Alexander, D., Kasturi, G., Franklin, B.A., Cardiorespiratory fitness and short-term complications after bariatric surgery (2006) Chest, 130, pp. 517-525; Santry, H.P., Gillen, D.L., Lauderdale, D.S., Trends in bariatric surgical procedures (2005) JAMA, 294, pp. 1909-1917; Eagle, K.A., Berger, P.B., Calkins, H., Chaitman, B.R., Ewy, G.A., Fleischmann, K.E., Fleisher, L.A., Smith Jr., S.C., ACC/AHA guideline update for perioperative cardiovascular evaluation for noncardiac surgery: Executive summary: A report of the American College of Cardiology/American Heart Association Task Force on Practice Guidelines (2002) Circulation, 105, pp. 1257-1267. , Committee to Update the 1996 Guidelines on Perioperative Cardiovascular Evaluation for Noncardiac Surgery [published correction appears in Circulation. 2006;113:e846]; Ainsworth, B.E., Haskell, W.L., Whitt, M.C., Irwin, M.L., Swartz, A.M., Strath, S.J., O'Brien, W.L., Leon, A.S., Compendium of physical activities: An update of activity codes and MET intensities (2000) Med Sci Sports Exerc, 32, pp. S498-S504; Jones, N., (1997) Clinical Exercise Testing, , Philadelphia Pa: Saunders; Morris, C.K., Myers, J., Froelicher, V.F., Kawaguchi, T., Ueshima, K., Hideg, A., Nomogram based on metabolic equivalents and age for assessing aerobic exercise capacity in men (1993) J Am Coll Cardiol, 22, pp. 175-182; Hansen, J.E., Sue, D.Y., Wasserman, K., Predicted values for clinical exercise testing (1984) Am Rev Respir Dis, 129, pp. S49-S55; Hsich, E., Gorodeski, E.Z., Starling, R.C., Blackstone, E.H., Ishwaran, H., Lauer, M.S., Importance of treadmill exercise time as an initial prognostic screening tool in patients with systolic left ventricular dysfunction (2009) Circulation, 119, pp. 3189-3197; Baba, R., Nagashima, M., Goto, M., Nagano, Y., Yokota, M., Tauchi, N., Nishibata, K., Oxygen intake efficiency slope: A new index of cardiore-spiratory functional reserve derived from the relationship between oxygen consumption and minute ventilation during incremental exercise (1996) Nagoya J Med Sci, 59, pp. 55-62; Van Laethem, C., Bartunek, J., Goethals, M., Nellens, P., Andries, E., Vander-Heyden, M., Oxygen uptake efficiency slope, a new submaximal parameter in evaluating exercise capacity in chronic heart failure patients (2005) Am Heart J, 149, pp. 175-180; Hollenberg, M., Tager, I.B., Oxygen uptake efficiency slope: An index of exercise performance and cardiopulmonary reserve requiring only sub-maximal exercise (2000) J Am Coll Cardiol, 36, pp. 194-201; Davies, L.C., Wensel, R., Georgiadou, P., Cicoira, M., Coats, A.J., Piepoli, M.F., Francis, D.P., Enhanced prognostic value from cardiopulmonary exercise testing in chronic heart failure by non-linear analysis: Oxygen uptake efficiency slope (2006) Eur Heart J, 27, pp. 684-690; Baba, R., Kubo, N., Morotome, Y., Iwagaki, S., Reproducibility of the oxygen uptake efficiency slope in normal healthy subjects (1999) Journal of Sports Medicine and Physical Fitness, 39 (3), pp. 202-206; Pogliaghi, S., Dussin, E., Tarperi, C., Cevese, A., Schena, F., Calculation of oxygen uptake efficiency slope based on heart rate reserve end-points in healthy elderly subjects (2007) Eur J Appl Physiol, 101, pp. 691-696; Arena, R., Myers, J., Hsu, L., Peberdy, M.A., Pinkstaff, S., Bensimhon, D., Chase, P., Guazzi, M., The minute ventilation/carbon dioxide production slope is prognostically superior to the oxygen uptake efficiency slope (2007) J Card Fail, 13, pp. 462-469; Myers, J., Arena, R., Dewey, F., Bensimhon, D., Abella, J., Hsu, L., Chase, P., Peberdy, M.A., A cardiopulmonary exercise testing score for predicting outcomes in patients with heart failure (2008) Am Heart J, 156, pp. 1177-1183; Arena, R., Peberdy, M.A., Reliability of resting end-tidal carbon dioxide in chronic heart failure (2005) J Cardiopulm Rehabil, 25, pp. 177-180; Jin, X., Weil, M.H., Tang, W., Povoas, H., Pernat, A., Xie, J., Bisera, J., End-tidal carbon dioxide as a noninvasive indicator of cardiac index during circulatory shock (2000) Crit Care Med, 28, pp. 2415-2419; Isserles, S.A., Breen, P.H., Can changes in end-tidal PCO2 measure changes in cardiac output? (1991) Anesth Analg, 73, pp. 808-814; Matsumoto, A., Itoh, H., Eto, Y., Kobayashi, T., Kato, M., Omata, M., Watanabe, H., Momomura, S., End-tidal CO2 pressure decreases during exercise in cardiac patients: Association with severity of heart failure and cardiac output reserve (2000) J Am Coll Cardiol, 36, pp. 242-249; Arena, R., Guazzi, M., Myers, J., Prognostic value of end-tidal carbon dioxide during exercise testing in heart failure (2007) International Journal of Cardiology, 117 (1), pp. 103-108. , DOI 10.1016/j.ijcard.2006.04.058, PII S0167527306005092; Arena, R., Myers, J., Abella, J., Pinkstaff, S., Brubaker, P., Moore, B., Kitzman, D., Guazzi, M., The partial pressure of resting end-tidal carbon dioxide predicts major cardiac events in patients with systolic heart failure (2008) Am Heart J, 156, pp. 982-988; Bradley, T.D., The ups and downs of periodic breathing: Implications for mortality in heart failure (2003) J Am Coll Cardiol, 41, pp. 2182-2184; Leite, J.J., Mansur, A.J., De Freitas, H.F., Chizola, P.R., Bocchi, E.A., Terra-Filho, M., Neder, J.A., Lorenzi-Filho, G., Periodic breathing during incremental exercise predicts mortality in patients with chronic heart failure evaluated for cardiac transplantation (2003) J Am Coll Cardiol, 41, pp. 2175-2181; Guazzi, M., Arena, R., Ascione, A., Piepoli, M., Guazzi, M.D., Exercise oscillatory breathing and increased ventilation to carbon dioxide production slope in heart failure: An unfavorable combination with high prognostic value (2007) Am Heart J, 153, pp. 859-867; Ribeiro, J.P., Periodic breathing in heart failure: Bridging the gap between the sleep laboratory and the exercise laboratory (2006) Circulation, 113, pp. 9-10; Somers, V.K., Sleep: A new cardiovascular frontier (2005) N Engl J Med., 353, pp. 2070-2073. , [published correction appears in N Engl J Med 2005;353:2523]; Hanly, P., Zuberi, N., Gray, R., Pathogenesis of Cheyne-Stokes respiration in patients with congestive heart failure: Relationship to arterial PCO2 (1993) Chest, 104, pp. 1079-1084; Ben-Dov, I., Sietsema, K.E., Casaburi, R., Wasserman, K., Evidence that circulatory oscillations accompany ventilatory oscillations during exercise in patients with heart failure (1992) Am Rev Respir Dis, 145, pp. 776-781; Francis, D.P., Willson, K., Davies, L.C., Coats, A.J., Piepoli, M., Quantitative general theory for periodic breathing in chronic heart failure and its clinical implications (2000) Circulation, 102, pp. 2214-2221; Ponikowski, P., Anker, S.D., Chua, T.P., Francis, D., Banasiak, W., Poole-Wilson, P.A., Coats, A.J., Piepoli, M., Oscillatory breathing patterns during wakefulness in patients with chronic heart failure: Clinical implications and role of augmented peripheral chemosensitivity (1999) Circulation, 100, pp. 2418-2424; Guazzi, M., Raimondo, R., Vicenzi, M., Arena, R., Proserpio, C., Sarzi Braga, S., Pedretti, R., Exercise oscillatory ventilation may predict sudden cardiac death in heart failure patients (2007) J Am Coll Cardiol, 50, pp. 299-308; Myers, J., Salleh, A., Buchanan, N., Smith, D., Neutel, J., Bowes, E., Froelicher, V.F., Ventilatory mechanisms of exercise intolerance in chronic heart failure (1992) Am Heart J, 124, pp. 710-719; Guazzi, M., Marenzi, G., Assanelli, E., Perego, G.B., Cattadori, G., Doria, E., Agostoni, P.G., Evaluation of the dead space/tidal volume ratio in patients with chronic congestive heart failure (1995) J Card Fail, 1, pp. 401-408; Jones, N.L., Robertson, D.G., Kane, J.W., Difference between end-tidal and arterial PCO2 in exercise (1979) J Appl Physiol, 47, pp. 954-960; Lewis, D.A., Sietsema, K.E., Casaburi, R., Sue, D.Y., Inaccuracy of nonin-vasive estimates of VD/VT in clinical exercise testing (1994) Chest, 106, pp. 1476-1480; Koike, A., Itoh, H., Kato, M., Sawada, H., Aizawa, T., Fu, L.T., Watanabe, H., Prognostic power of ventilatory responses during submaximal exercise in patients with chronic heart disease (2002) Chest, 121, pp. 1581-1588; Barstow, T.J., Casaburi, R., Wasserman, K., O2 uptake kinetics and the O2 deficit as related to exercise intensity and blood lactate (1993) J Appl Physiol, 75, pp. 755-762; Hickson, R.C., Bomze, H.A., Hollozy, J.O., Faster adjustment of O2 uptake to the energy requirement of exercise in the trained state (1978) J Appl Physiol, 44, pp. 877-881; Powers, S.K., Dodd, S., Beadle, R.E., Oxygen uptake kinetics in trained athletes differing in V? O 2max (1985) Eur J Appl Physiol Occup Physiol, 54, pp. 306-308; Sietsema, K.E., Ben-Dov, I., Zhang, Y.Y., Sullivan, C., Wasserman, K., Dynamics of oxygen uptake for submaximal exercise and recovery in patients with chronic heart failure (1994) Chest, 105, pp. 1693-1700; Sietsema, K.E., Cooper, D.M., Perloff, J.K., Rosove, M.H., Child, J.S., Canobbio, M.M., Whipp, B.J., Wasserman, K., Dynamics of oxygen uptake during exercise in adults with cyanotic congenital heart disease (1986) Circulation, 73, pp. 1137-1144; Koike, A., Yajima, T., Adachi, H., Shimizu, N., Kano, H., Sugimoto, K., Niwa, A., Hiroe, M., Evaluation of exercise capacity using submaximal exercise at a constant work rate in patients with cardiovascular disease (1995) Circulation, 91, pp. 1719-1724; Harris, R.C., Edwards, R.H., Hultman, E., Nordesjö, L.O., Nylind, B., Sahlin, K., The time course of phosphorylcreatine resynthesis during recovery of the quadriceps muscle in man (1976) Pflugers Arch, 367, pp. 137-142; Barstow, T.J., Lamarra, N., Whipp, B.J., Modulation of muscle and pulmonary O2 uptakes by circulatory dynamics during exercise (1990) J Appl Physiol, 68, pp. 979-989; Guazzi, M., Tumminello, G., Di Marco, F., Fiorentini, C., Guazzi, M.D., The effects of phosphodiesterase-5 inhibition with sildenafil on pulmonary hemodynamics and diffusion capacity, exercise ventilatory efficiency, and oxygen uptake kinetics in chronic heart failure (2004) J Am Coll Cardiol, 44, pp. 2339-2348; De Groote, P., Millaire, A., Decoulx, E., Nugue, O., Guimier, P., Ducloux, G., Kinetics of oxygen consumption during and after exercise in patients with dilated cardiomyopathy: New markers of exercise intolerance with clinical implications (1996) J Am Coll Cardiol, 28, pp. 168-175; Wilson, J.R., Rayos, G., Yeoh, T.K., Gothard, P., Dissociation between peak exercise oxygen consumption and hemodynamic dysfunction in potential heart transplant candidates (1995) J Am Coll Cardiol, 26, pp. 429-435; Chomsky, D.B., Lang, C.C., Rayos, G.H., Shyr, Y., Yeoh, T.K., Pierson III, R.N., Davis, S.F., Wilson, J.R., Hemodynamic exercise testing: A valuable tool in the selection of cardiac transplantation candidates (1996) Circulation, 94, pp. 3176-3183; Metra, M., Faggiano, P., D'Aloia, A., Nodari, S., Gualeni, A., Raccagni, D., Dei Cas, L., Use of cardiopulmonary exercise testing with hemodynamic monitoring in the prognostic assessment of ambulatory patients with chronic heart failure (1999) J Am Coll Cardiol, 33, pp. 943-950; Lang, C.C., Agostoni, P., Mancini, D.M., Prognostic significance and measurement of exercise-derived hemodynamic variables in patients with heart failure (2007) J Card Fail, 13, pp. 672-679; Stringer, W.W., Hansen, J.E., Wasserman, K., Cardiac output estimated noninvasively from oxygen uptake during exercise (1997) J Appl Physiol, 82, pp. 908-912; Lang, C.C., Karlin, P., Haythe, J., Tsao, L., Mancini, D.M., Ease of noninvasive measurement of cardiac output coupled with peak V? O2 determination at rest and during exercise in patients with heart failure (2007) Am J Cardiol, 99, pp. 404-405; Grossman, W., Blood flow measurement: Cardiac output and vascular resistance (2006) Grossman's Cardiac Catheterization, Angiography, and Intervention, pp. 148-162. , Baim D, ed Philadelphia, Pa: Lippincott Williams & Wilkins; Sun, X.-G., Hansen, J.E., Stringer, W.W., Ting, H., Wasserman, K., Carbon dioxide pressure-concentration relationship in arterial and mixed venous blood during exercise (2001) Journal of Applied Physiology, 90 (5), pp. 1798-1810; Agostoni, P., Cattadori, G., Apostolo, A., Contini, M., Palermo, P., Marenzi, G., Wasserman, K., Noninvasive measurement of cardiac output during exercise by inert gas rebreathing technique: A new tool for heart failure evaluation (2005) J Am Coll Cardiol, 46, pp. 1779-1781; Baum, M.M., Moss, J.A., Kumar, S., Wagner, P.D., Non-invasive measurement of cardiac output: Evaluation of new infrared absorption spectrometer (2006) Respir Physiol Neurobiol, 153, pp. 191-201; Johnson, B.D., Weisman, I.M., Zeballos, R.J., Beck, K.C., Emerging concepts in the evaluation of ventilatory limitation during exercise: The exercise tidal flow-volume loop (1999) Chest, 116, pp. 488-503; O'Donnell, D.E., Hyperinflation, dyspnea, and exercise intolerance in chronic obstructive pulmonary disease (2006) Proc Am Thorac Soc, 3, pp. 180-184; Johnson, B.D., Beck, K.C., Olson, L.J., O'Malley, K.A., Allison, T.G., Squires, R.W., Gau, G.T., Ventilatory constraints during exercise in patients with chronic heart failure (2000) Chest, 117, pp. 321-332; Dempsey, J.A., McKenzie, D.C., Haverkamp, H.C., Eldridge, M.W., Update in the understanding of respiratory limitations to exercise performance in fit, active adults (2008) Chest, 134, pp. 613-622.

415. Abd SK, Al-Haddad S, Hashim F, Abdullah AB, Yussof S. Cloud computing concept, classifications, applications and challenges. Int J Control Theory Appl. 2016;9(30):261-88.

416. Hossain MS, Muhammad G, editors. Cloud-assisted framework for health monitoring. 2015 28th IEEE Canadian Conference on Electrical and Computer Engineering, CCECE 2015; 2015: Institute of Electrical and Electronics Engineers Inc.

417. Zhou B, Ma Q, Song Y, Bian C, editors. Cloud-based dynamic electrocardiogram monitoring and analysis system. 9th International Congress on Image and Signal Processing, BioMedical Engineering and Informatics, CISP-BMEI 2016; 2016: Institute of Electrical and Electronics Engineers Inc.

418. Al-Rakhami M, Alhamed A, editors. Cloud-based graphical simulation tool of ECG for educational purpose. International Conference on Internet of Things and Cloud Computing, ICC 2016; 2016: Association for Computing Machinery.

419. Bagnall A, Janacek G. Clustering time series with clipped data. Mach Learn. 2005;58(2-3):151-78. doi: 10.1007/s10994-005-5825-6.

420. Kakkasageri MS, Manvi SS, Pitt J. Cognitive agent based critical information gathering and dissemination in vehicular Ad hoc networks. Wireless Pers Commun. 2013;69(4):1107-29. doi: 10.1007/s11277-012-0623-5
[truncated: 1,126,413 more chars]
