# Supplementary material for: Educational Software Applied in Teaching Electrocardiogram: A Systematic Review
Source: Biomed Res Int. 2018 Mar 15;2018:8203875. doi: 10.1155/2018/8203875 (PMC5875041; doi:10.1155/2018/8203875)
Supplement: Supplementary 3 — S3 Appendix: eligibility—full-text articles assessed for eligibility (DOCX). [file 8203875.f3.docx]

**S3 Appendix - ELIGIBILITY - Full-text articles assessed for eligibility**

The following 59 Full-text articles assessed for eligibility.

1. Akgun T, Karabay CY, Kocabay G, Kalayci A, Oduncu V, Guler A, et al. Learning electrocardiogram on YouTube: How useful is it? J Electrocardiol. 2014;47(1):113-7. doi: 10.1016/j.jelectrocard.2013.09.004.

2. Al-Rakhami M, Alhamed A, editors. Cloud-based graphical simulation tool of ECG for educational purpose. International Conference on Internet of Things and Cloud Computing, ICC 2016; 2016: Association for Computing Machinery.

3. Bailey M, Kirchen G, Bonaventura B, Rosborough K, Abdel-Rasoul M, Dzwonczyk R. Intraoperative MRI electrical noise and monitor ECG filters affect arrhythmia detection and identification. J Clin Monit Comput. 2012;26(3):157-61. doi: 10.1007/s10877-012-9346-0. PubMed PMID: 22389138.

4. Bond RR, Finlay DD, Nugent CD, Moore G, Guldenring D. A simulation tool for visualizing and studying the effects of electrode misplacement on the 12-lead electrocardiogram. J Electrocardiol. 2011;44(4):439-44. doi: 10.1016/j.jelectrocard.2011.03.006. PubMed PMID: 21704221.

5. Bond RR, Van Dam E, Van Dam P, Finlay DD, Guldenring D, editors. Evaluating the human-computer interaction of 'ECGSim': A virtual simulator to aid learning in electrocardiology. 42nd Computing in Cardiology Conference, CinC 2015; 2015: IEEE Computer Society.

6. Bond RR, Zhu T, Finlay DD, Drew B, Kligfield PD, Guldenring D, et al. Assessing computerized eye tracking technology for gaining insight into expert interpretation of the 12-lead electrocardiogram: an objective quantitative approach. J Electrocardiol. 2014;47(6):895-906. doi: 10.1016/j.jelectrocard.2014.07.011. PubMed PMID: 25110276.

7. Brown D, Chronister C. The Effect of Simulation Learning on Critical Thinking and Self-confidence When Incorporated Into an Electrocardiogram Nursing Course. Clinical Simulation in Nursing. 2009;5(1):e45-e52. doi: 10.1016/j.ecns.2008.11.001.

8. Burke JF, Gnall E, Umrudden Z, Kyaw M, Schick PK. Critical analysis of a computer-assisted tutorial on ECG interpretation and its ability to determine competency. Med Teach. 2008;30(2):e41-8. Epub 2008/05/09. doi: 10.1080/01421590801972471. PubMed PMID: 18464131.

9. Cairns AW, Bond RR, Finlay DD, Breen CJ, Guldenring D, Gaffney R, et al., editors. Interactive progressive-based approach to aid the human interpretation of the 12-lead Electrocardiogram. 42nd Computing in Cardiology Conference, CinC 2015; 2015: IEEE Computer Society.

10. Cardoso Martins A, Dias Costa P, Miguel Marques J, Cruz Correia R, editors. Electrocardiogram rhythm simulation in open source environment - A contribution to training in biomedical sciences. 3rd International Conference on Health Informatics, HEALTHINF 2010; 2010; Valencia.

11. Clavario P, Copello F, Giugliano M, Martinengo E, Biagini A, Damanti D, et al., editors. Use of an arrhythmia simulator for the evaluation of nurses' knowledge and as a teaching tool. Proceedings of the 1995 Conference on Computers in Cardiology; 1995; Los Alamitos, CA, United States

Vienna, Austria: IEEE.

12. Davies A, Macleod R, Bennett-Britton I, McElnay P, Bakhbakhi D, Sansom J. E-learning and near-peer teaching in electrocardiogram education: a randomised trial. Clin Teach. 2016;13(3):227-30. doi: 10.1111/tct.12421. PubMed PMID: 26135499.

13. Devitt P, Worthley S, Palmer E, Cehic D. Evaluation of a computer based package on electrocardiography. Aust N Z J Med. 1998;28(4):432-5. PubMed PMID: 9777109.

14. Fent G, Gosai J, Purva M. A randomized control trial comparing use of a novel electrocardiogram simulator with traditional teaching in the acquisition of electrocardiogram interpretation skill. J Electrocardiol. 2016;49(2):112-6. doi: 10.1016/j.jelectrocard.2015.11.005. PubMed PMID: 26709105.

15. Fukushima M, Inoue M, Fukunami M, Ishikawa K, Inada H, Abe H. Computer-assisted education system for arrhythmia (CAESAR). Comput Biomed Res. 1984;17(4):376-88. doi: 10.1016/0010-4809(84)90047-8. PubMed PMID: 6383704.

16. Granero-Molina J, Fernandez-Sola C, Lopez-Domene E, Hernandez-Padilla JM, Preto LS, Castro-Sanchez AM. Effects of web-based electrocardiography simulation on strategies and learning styles. Rev Esc Enferm USP. 2015;49(4):650-6. doi: 10.1590/S0080-623420150000400016. PubMed PMID: 26353103.

17. Güney E, Ekşi Z, Çakiroǧlu M. WebECG: A novel ECG simulator based on MATLAB Web Figure. Adv Eng Software. 2012;45(1):167-74. doi: 10.1016/j.advengsoft.2011.09.005

10.1007/s10916-009-9304-7; Tan, T., Chang, C.S., Huang, Y.F., Chen, Y.F., Lee, C., Development of a portable Linux-based ECG measurement and monitoring system (2009) J Med Syst, , doi:10.1007/s10916-009-9392-4; Oefinger, M.B., Mark, R.G., A web-based tool for visualization and collaborative annotation of physiological databases (2005) Computers in Cardiology, 32, pp. 163-165. , DOI 10.1109/CIC.2005.1588060, 1588060, Computers in Cardiology, 2005; Karthik, R., (2007) ECG Simulation Using MATLAB, B.E. Dissertation, , Anna University, Chennai; The Six Second ECG (Cardiac Rhythm Simulator), , http://www.skillstat.com/ecg_sim_demo.html, [accessed 28.04.11]; Martins, A.C., Costa, P.D., Marques, J.M., ECG Simulator, , http://simecg.sourceforge.net/, [accessed 28.04.11]; Lu, W., Wei, D., Zhu, X., Chen, W., A computer model based on real anatomy for electrophysiology study (2011) Adv Eng Softw, 42, pp. 463-476; Markiewicz, T., Using MATLAB software with Tomcat server and Java platform for remote image analysis in Pathology (2011) Diagn Pathol, 6 (SUPPL. 1), p. 18. , 10.1186/1746-1596-6-S1-S18; Kirba, I., Bayilmis, C., HealthFace: A web-based remote monitoring interface for medical healthcare systems based on wireless body area sensor network Turkish J Electr Eng Comput Sci, , doi:10.3906/elk-1011-934; Petropoulakis, L., Stephen, B., WebClass: Software to web-enable MATLAB for collaborative use (2005) Advances in Engineering Software, 36 (8), pp. 497-503. , DOI 10.1016/j.advengsoft.2005.02.006, PII S0965997805000463; Bayilmis, C., Development of A Web-based Educational Interface Using MATLAB Builder NE with Web Figure for Digital Modulation Techniques, , doi:2010;10.1002/cae.20427; Rosello, E.G., Lado, M.J., Mendez, A.J., Dacosta, J.G., Cota, M.P., A component framework for reusing a proprietary computer-aided engineering environment (2007) Advances in Engineering Software, 38 (4), pp. 256-266. , DOI 10.1016/j.advengsoft.2006.08.014, PII S0965997806001402; http://www.mathworks.com/help/toolbox/dotnetbuilder/, MATLAB Builder NE [accessed 28.04.11]UR - https://www.scopus.com/inward/record.uri?eid=2-s2.0-83555174612&doi=10.1016%2fj.advengsoft.2011.09.005&partnerID=40&md5=2b705db4e6c6dba4b0a09b9160491d4e.

18. Lessard Y, Sinteff JP, Siregar P, Julen N, Hannouche F, Rio S, et al., editors. An ECG analysis interactive training system for understanding arrhythmias. 22nd International Conference on Medical Informatics Europe, MIE 2009; 2009; Sarajevo19745450.

19. Mueller MP, Christ T, Dobrev D, Nitsche I, Stehr SN, Ravens U, et al. Teaching antiarrhythmic therapy and ECG in simulator-based interdisciplinary undergraduate medical education. Br J Anaesth. 2005;95(3):300-4. doi: 10.1093/bja/aei174. PubMed PMID: 15964889.

20. Muqri MR, Chng SE, Muqri F, Muqri A, editors. Normal and abnormal EKGs and heart sounds: Development of a diagnostic tool. 119th ASEE Annual Conference and Exposition; 2012; San Antonio, TX.

21. Rolskov BS, Räder S, Holst A, Kayser L, Ringsted C, Hastrup SJ, et al. The acquisition and retention of ECG interpretation skills after a standardized web-based ECG tutorial-a randomised study. BMC medical education [Internet]. 2016; 15:[36 p.]. Available from: http://onlinelibrary.wiley.com/o/cochrane/clcentral/articles/631/CN-01109631/frame.html

http://download.springer.com/static/pdf/806/art%253A10.1186%252Fs12909-015-0319-0.pdf?originUrl=http%3A%2F%2Fbmcmededuc.biomedcentral.com%2Farticle%2F10.1186%2Fs12909-015-0319-0&token2=exp=1493044776~acl=%2Fstatic%2Fpdf%2F806%2Fart%25253A10.1186%25252Fs12909-015-0319-0.pdf*~hmac=31f1e1c5e3137c7abaad62de2946cc4e88b9d79d2d00a977e804474731358147.

22. Schlindwein M, von Wagner G, Kirst M, Rajewicz M, Karl F, Schochlin J, et al. Mobile patient simulator for resuscitation training with automatic external defibrillators. Biomed Tech (Berl). 2002;47 Suppl 1 Pt 2:559-60. Epub 2002/12/06. PubMed PMID: 12465236.

23. Sprick C, Ruthenbeck GS, Owen H, Reynolds KJ, editors. Virtual patient monitors for new user familiarization. Medicine Meets Virtual Reality 16 - Parallel, Combinatorial, Convergent: NextMed by Design, MMVR 2008; 2008; Long Beach, CA18391350.

24. Stasiu RK, De Britto J, Da Silva Dias J, Scalabrin E. Teaching of electrocardiogram interpretation guided by a tutorial expert. Proc IEEE Symp Comput Based Med Syst. 2001:487-92. doi: 10.1109/cbms.2001.941766.

25. Takeuchi A, Hirose M, Hamada A, Ikeda N. Simulation system of arrhythmia using ActiveX control. Comput Methods Programs Biomed. 2005;79(1):49-57. doi: 10.1016/j.cmpb.2005.03.011. PubMed PMID: 15925427.

26. Tubaishat A, Tawalbeh LI. Effect of Cardiac Arrhythmia Simulation on Nursing Students' Knowledge Acquisition and Retention. West J Nurs Res. 2015;37(9):1160-74. doi: 10.1177/0193945914545134. PubMed PMID: 25082710.

27. Web scan. Emerg Nurse. 2009;16(10):4. Epub 2009/03/10. doi: 10.7748/en.16.10.4.s9. PubMed PMID: 27644355.

28. Alinier G, Gordon R, Harwood C, Hunt WB. 12-lead ECG training: the way forward. Nurse Educ Today. 2006;26(1):87-92. doi: 10.1016/j.nedt.2005.08.004. PubMed PMID: 16182413.

29. Baquero GA, Banchs JE, Ahmed S, Naccarelli GV, Luck JC. Surface 12 lead electrocardiogram recordings using smart phone technology. J Electrocardiol. 2015;48(1):1-7. doi: 10.1016/j.jelectrocard.2014.09.006. PubMed PMID: 25283739.

30. Baxter R. 12 lead ECG interpretation: The self-assessment Approach. Intensive Care Nursing. 1990;6(4):213-4. doi: 10.1016/0266-612x(90)90033-4.

31. Bond RR, Finlay DD, McLaughlin J, Guldenring D, Cairns A, Kennedy A, et al. Human factors analysis of the CardioQuick Patch(R): A novel engineering solution to the problem of electrode misplacement during 12-lead electrocardiogram acquisition. J Electrocardiol. 2016;49(6):911-8. doi: 10.1016/j.jelectrocard.2016.08.009. PubMed PMID: 27662775.

32. Brown LH, Gough JE, Hawley CR. Accuracy of rural EMS provider interpretation of three-lead ECG rhythm strips. Prehosp Emerg Care. 1997;1(4):259-62. doi: 10.1080/10903129708958820. PubMed PMID: 9709367.

33. Buttussi F, Pellis T, Cabas Vidani A, Pausler D, Carchietti E, Chittaro L. Evaluation of a 3D serious game for advanced life support retraining. Int J Med Inform. 2013;82(9):798-809. doi: 10.1016/j.ijmedinf.2013.05.007. PubMed PMID: 23763908.

34. Caner C, Engin M, Engin EZ. The programmable ECG simulator. J Med Syst. 2008;32(4):355-9. doi: 10.1007/s10916-008-9140-1. PubMed PMID: 18619099.

35. Chiang P, Zheng J, Yu Y, Mak KH, Chui CK, Cai Y. A VR simulator for intracardiac intervention. IEEE Comput Graph Appl. 2013;33(1):44-57. doi: 10.1109/MCG.2012.47. PubMed PMID: 24807881.

36. Cotin S, Dawson SL, Meglan D, Shaffer DW, Ferrell MA, Bardsley RS, et al. ICTS, an interventional cardiology training system. Stud Health Technol Inform. 2000;70:59-65. Epub 2000/09/08. PubMed PMID: 10977584.

37. Dong J, Zhang S, Wan Y, editors. A hybrid framework for ECG interpretation by computer and its evaluation platform. BioMedical Engineering and Informatics: New Development and the Future - 1st International Conference on BioMedical Engineering and Informatics, BMEI 2008; 2008; Sanya, Hainan.

38. Drǎghiciu N, Creţiu P. Ecg simulator. J Electr Electron Eng. 2013;6(1):33-6.

39. Dubin S, Butler A, Hanes D. EKGenius: a computer-interactive electrophysiology learning tool. Biomed Sci Instrum. 1994;30:133-40. PubMed PMID: 7948625.

40. Fordham Z, Devereaux R, Edwards A. Teaching emergency medicine residents transvenous cardiac pacing: Simulation technology versus traditional methods. Acad Emerg Med [Internet]. 2010; 17:[S196 p.]. Available from: http://onlinelibrary.wiley.com/o/cochrane/clcentral/articles/712/CN-01055712/frame.html

http://onlinelibrary.wiley.com/store/10.1111/j.1553-2712.2010.00743.x/asset/j.1553-2712.2010.00743.x.pdf?v=1&t=j1w88f24&s=c4466cf04f53f29a4b88de0a336c2e428b024838.

41. Howlett PJ, Pearson SA. Simple e.c.g. arrhythmia simulator. Med Biol Eng Comput. 1978;16(2):217-8. doi: 10.1007/BF02451926. PubMed PMID: 309047.

42. Johnson F, Sawle GV, Tomlinson DR. An e.c.g. vector simulator to facilitate learning of the basic principles of electrocardiography [proceedings]. J Physiol. 1980;298:1P. Epub 1980/01/01. PubMed PMID: 7359376; PubMed Central PMCID: PMCPMC1279024.

43. Kim JH, Kim WO, Min KT, Yang JY, Nam YT. Learning by computer simulation does not lead to better test performance than textbook study in the diagnosis and treatment of dysrhythmias. J Clin Anesth. 2002;14(5):395-400. doi: 10.1016/s0952-8180(02)00384-7.

44. Koebele J, Heinke M, Ismer B. Soundcard based Multichannel Live-ECG Simulator for Research, Development and Education. Biomed Tech (Berl). 2012;57 Suppl 1(SUPPL. 1 TRACK-O):331-2. doi: 10.1515/bmt-2012-4024. PubMed PMID: 23096314.

45. Kontodimopoulos N, Pallikarakis N, Christov I, Daskalov I. In-house development of test equipment for quality control and training. Case study: a prototype ECG simulator-tester. Med Eng Phys. 1998;20(10):717-21. Epub 1999/05/01. PubMed PMID: 10223639.

46. Larnard DJ. Simulation of cardiac arrhythmia haemodynamics with a real-time computer model. Med Biol Eng Comput. 1986;24(5):465-70. doi: 10.1007/BF02443960. PubMed PMID: 3821202.

47. Lavranos G, Koliaki C, Briasoulis A, Nikolaou A, Stefanadis C. Effectiveness of current teaching methods in Cardiology: the SKILLS (medical Students Knowledge Integration of Lower Level clinical Skills) study. Hippokratia. 2013;17(1):34-7. PubMed PMID: 23935341; PubMed Central PMCID: PMCPMC3738274.

48. Le-Huy P, Yvroud E, Dion J-L, editors. VERSATILE CARDIAC ARRHYTHMIA SIMULATOR. Conference Proceedings - IEEE Instrumentation and Measurement Technology Conference; 1987; New York, NY, USA

Boston, MA, USA: IEEE.

49. Lessard Y, Sinteff JP, Siregar P, Julen N, Hannouche F, Rio S, et al., editors. Oaat, a new, simple and powerfull interactive tool for ecg analysis learning. e-Learning 2008, MCCSIS'08 - IADIS Multi Conference on Computer Science and Information Systems; 2008; Amsterdam.

50. Omar HM, Ibrahim R, Jaafar A, editors. Methodology to evaluate interface of educational computer game. 2011 International Conference on Pattern Analysis and Intelligent Robotics - Special Track on Human Centered Multimedia Analysis, ICPAIR 2011; 2011; Putrajaya.

51. Omar Mohamed H, Yusoff R, Jaafar A, editors. Quantitive analysis in a heuristic evaluation for usability of educational computer game (UsaECG). 2012 International Conference on Information Retrieval and Knowledge Management, CAMP'12; 2012; Kuala Lumpur.

52. Raupach T, Harendza S, Anders S, Schuelper N, Brown J. How can we improve teaching of ECG interpretation skills? Findings from a prospective randomised trial. J Electrocardiol. 2016;49(1):7-12. doi: 10.1016/j.jelectrocard.2015.10.004. PubMed PMID: 26615874.

53. Reisman SS, Friedman KJ, editors. EKG CHALLENGER - A SELF TEACHING EKG COMPUTER PROGRAM. Proceedings of the Ninth Annual Conference of the IEEE Engineering in Medicine and Biology Conference; 1987; New York, NY, USA

Boston, MA, USA: IEEE.

54. Sandige RS, Ferris CD, Bhaskaran A. Electronic ECG simulator. Biomed Sci Instrum. 1992;28:21-5. PubMed PMID: 1643225.

55. Shahein HI. Computers in health-sciences education. An application to electrocardiography. Comput Programs Biomed. 1983;17(3):213-23. doi: 10.1016/0010-468X(83)90042-9. PubMed PMID: 6370580.

56. Siregar P, Chahine M, Lemoulec F, Le Beux P. An interactive qualitative model in cardiology. Comput Biomed Res. 1995;28(6):443-78. doi: 10.1006/cbmr.1995.1029. PubMed PMID: 8770534.

57. Takashina T, Shimizu M, Katayama H. A new cardiology patient simulator. CARDIOLOGY. 1997;88(5):408-13. PubMed PMID: 9286501.

58. Tofield A. Cardiac arrhythmia challenge: a new App. Eur Heart J. 2013;34(44):3392. PubMed PMID: 24400366.

59. Xu K. Design of Heart Sounds and ECG Real-time Auscultation System Based on LabVIEW. Int J Control Autom. 2015;8(5):117-26. doi: 10.14257/ijca.2015.8.5.11.
