# Supplementary material for: Educational Software Applied in Teaching Electrocardiogram: A Systematic Review
Source: Biomed Res Int. 2018 Mar 15;2018:8203875. doi: 10.1155/2018/8203875 (PMC5875041; doi:10.1155/2018/8203875)
Supplement: Supplementary 4 — S4 Appendix: complete overview of the extracted data (DOCX). [file 8203875.f4.docx]

**Complete overview of the extracted data**

| **ID** | **Year** | **Type of Software** | **Form of Availability** | **Subjects** | **Subjects** |
| --- | --- | --- | --- | --- | --- |
| S1 | 1983 | Tutorial Software | Computer (locally) | Teaching of electrophysiology and/or physiology of the heart/ Normal ECG / Arrhythmias | Nonexperimental / level 1 KirkPatrick (reaction) / level 2 KirkPatrick  (learning) |
| S2 | 1984 | Simulator | Computer (locally) | Teaching of electrophysiology and/or physiology of the heart/ Normal ECG/ Arrhythmias | Nonexperimental / level 1 KirkPatrick (reaction) / level 2 KirkPatrick  (learning) |
| S3 | 1986 | Tutorial Software | Computer (locally) | Teaching of electrophysiology and/or physiology of the heart/ Normal ECG/ Arrhythmias | Nonexperimental / level 1 KirkPatrick (reaction) |
| S4 | 1998 | Tutorial Software | Computer (locally) | Teaching of electrophysiology and/or physiology of the heart/ Normal ECG/ Arrhythmias | Experimental / level 2 KirkPatrick (learning) |
| S5 | 1998 | Tutorial Software | Computer (locally) | Teaching of electrophysiology and/or physiology of the heart/ Arrhythmias | Nonexperimental / level 1 KirkPatrick (reaction) / level 2 KirkPatrick  (learning) |
| S6 | 2005 | Simulator | Computer (locally) | Teaching of electrophysiology and/or physiology of the heart/ Arrhythmias | Nonexperimental / level 1 KirkPatrick (reaction) |
| S7 | 2006 | Problem Solving | Internet (Web) | Normal ECG/ Arrhythmias | Nonexperimental / level 1 KirkPatrick (reaction) |
| S8 | 2007 | Simulator | Computer (locally) | Teaching of electrophysiology and/or physiology of the heart/ Normal ECG | Quasi Experimental / level 2 KirkPatrick (learning) |
| S9 | 2008 | Tutorial Software | Computer (locally) | Teaching of electrophysiology and/or physiology of the heart/ Arrhythmias | Nonexperimental / level 2 KirkPatrick (learning) |
| S10 | 2008 | Tutorial Software | Internet (Web) | Teaching of electrophysiology and/or physiology of the heart/ Normal ECG/ Arrhythmias | Quasi Experimental / level 1 KirkPatrick (reaction) / level 2 KirkPatrick  (learning) |
| S11 | 2009 | Tutorial Software | Computer (locally) | Teaching of electrophysiology and/or physiology of the heart/ Normal ECG/ Arrhythmias | Nonexperimental / level 1 KirkPatrick (reaction) / level 2 KirkPatrick (learning) |
| S12 | 2011 | Simulator | Internet (Web) | ECG Examination/ Normal ECG | Nonexperimental / level 1 KirkPatrick (reaction) |
| S13 | 2012 | Simulator | Internet (Web) | Teaching of electrophysiology and/or physiology of the heart/ Normal ECG/ Arrhythmias | Nonexperimental / level 1 KirkPatrick (reaction) |
| S14 | 2015 | Tutorial Software | Internet (Web) | Teaching of electrophysiology and/or physiology of the heart/ Normal ECG/ Arrhythmias | Nonexperimental / Level 1 KirkPatrick (reaction) / Level 2 KirkPatrick (learning) |
| S15 | 2015 | Problem Solving | Computer (locally) | Teaching of electrophysiology and/or physiology of the heart/ Normal ECG/ Arrhythmias | Experimental / level 2 KirkPatrick (aprendizagem) |
| S16 | 2016 | Tutorial Software | Internet (Web) | Teaching of electrophysiology and/or physiology of the heart/ Normal ECG/ Arrhythmias | Experimental / level 1 KirkPatrick (reação) / level 2 KirkPatrick  (aprendizagem) |
| S17 | 2016 | Simulator | Internet (Web) | Teaching of electrophysiology and/or physiology of the heart/ Normal ECG/ Arrhythmias | Quasi Experimental / level 1 KirkPatrick (reaction) |

**ID Evaluation (which types of study**

**Representation (X=treatment; O=measures/evidence; R=random**

**Assignment)**

**Classification Evaluation Level**

**(Kirkpatric)**

**Instrument**

**(through what)**

**Detailing (learning task)**

**Main findings**

**(conclusions)**

**S1** Use case: one Shot posttest only

X O

**S2** One-shot

pre-test – post-test

O X O

**S3** Use case: one Shot posttest only

X O

Nonexperimental 1 e 2 Questionnaires

Nonexperimental 1 e 2 Questionnaires

Nonexperimental 1

• •The student uses the software to learn electrocardiography through readings on the teaching of ECG based on use case on normal and abnormal ECG, and

subsequently answer •

the exercises.

• •Students must acquire the ability to diagnose logically complex arrhythmias. They can view the patterns of 18 types of arrhythmias or enter parameters in the model to simulate a particular arrhythmia, • in a trial and error approach.

• •Students learn heart rhythms and arrhythmias by navigating through the menus in the software that displays text that covers the most important information about 22

cardiac arrhythmias •

in a step-by-step approach, illustrating all the points for learning with high resolution graphics. In addition, a questionnaire and random access to each individual arrhythmia is available.

•Indicates a useful and effective method for learning

•Useful to consolidate the knowledge.

•The majority of students would like to see it applied in other courses

•Learning of 2 (two) hours with this system, significantly higher than those prior to use.

•Learned with interest

•System is useful and

effective for training the interpretation of arrhythmias using ECG.

•The results indicated an improvement in knowledge;

•Students found it easy to use and a valuable addition to the teaching curriculum

•Teachers and the educational institution also found it useful and complementary tool.

**S4** Randomized pretest - posttest

control group

R O X1 O R O X2 O R O X3 O

experimental 2 -

• • The group that used computers was the only one to achieve a significant increase in performance in their knowledge of electrocardiography and in its ability to interpret ECG

**S5** Use case: one Shot posttest only

Nonexperimental 1 e 2 survey

• • • This system has proved to be useful and

effective for the training of X O medical students in ECG interpretation of arrhythmias

• Simple to use

| **S6** | Use case: one | Shot | Nonexperimental | 1 | Questionnaires | Subsequently use a | • The students |
| --- | --- | --- | --- | --- | --- | --- | --- |
|  | posttest only |  |  |  |  | self assessment | were interested in the ECG |

software through the

X O analysis of 140 ECG

and then perform a

waves that appear on the

screen due to its real-time

simulation.

series of exercises for • Although no formal survey

resolution of

problems in

cardiology.

has been yet, we are given favorable comments, written and oral, in a favorable manner from the students.

**S7** Use case: one Shot

Nonexperimental 1

• Users were taught to • In a survey conducted

posttest only

X O

study cardiac arrhythmia through the selection of 35 arrhythmias

contained in the software. Explanations and pictures were presented. The arrhythmias were drawn in real time on the ECG. In addition to solving exercises. ECG could be analyzed and the speed of using commands of the software could be controlled

anonymously by trainees exposed to the program, 20 of 21 agreed strongly that the ECGViewer was a very useful teaching tool, more than any other, and easy to use.

**S8** Static group comparison group

X O O

Quasi 2 experimental

• • • Demonstrated greater accuracy in the calculation of the mean QRS axis than those who are instructed solely by the classical approach

• The study shows that teach electrocardiogram using ECGSIM is superior to traditional methods alone

**S9** Use case: one Shot posttest only

X O

Nonexperimental 2 Questionnaire •

• The post-test revealed that the tutorial has contributed to a significant improvement in the recognition of resources. The competency testing distinguished among the residents with pending grades and those who needed remediation.

| **S10** | Static | group | Quasi | 1 e 2 | • The participants | • On average, the |
| --- | --- | --- | --- | --- | --- | --- |
|  | comparison group |  | experimental |  | analyze digitized | usefulness of the program |
|  | X O |  |  |  | ECG, through the  manipulation of | and the quality of the  software of the program |

O virtual tools (calipers,

ruler and

magnification) through the mouse for crucial measurements

were rated as very good.

• The group that used the educational software obtained better assessment grades;

and comparisons of • The results suggest benefits

intervals.

in the use of a program of learning through an educational software.

**S11** Use case: one Shot posttest only

X O

nonexperimental 1 e 2

• The students used the interactive software to assist in the calculation of important parameters for ECG

• Although useful for distance education, OAAT is not intended to be a substitute for the traditional forms of teaching;

interpretation, in • However, an initial

**S12** one Shot postest only

particular the mean QRS axis. The ECG were selected from a

database.

Nonexperimental 1 Oral and written • The participants had

examination of the scores of a group of 18 students have already showed that, after an average of two attempts, all students have achieved a score of 100% and the correct diagnosis of five cases of arrhythmia. The student’s satisfaction is linked to performance.

• It has the

comments

X O generally

favorable.

to recognize all the details and abnormal characteristics of the

potential to be used to support to detect poor electrode placement.

ECG and then had to • Could be used to help the

**S13** one Shot posttest only

X O

Nonexperimental 1 survey

diagnose the underlying disorder of a list of possible

diagnoses. The interactivity was facilitated by the provision of a quick and thorough reasoning. The active learning was promoted by asking questions before the discussion of ECGs.

• • Users can access the ECG simulator using only the web browser without a need for any program. It can set the parameters of heart rate and amplitude / period of

students in education on the effects of poor placement of the electrodes

• 70% of the students consider that the WebECG facilitates learning of various types of ECG signals

• It is easy to use

• The simulator is a bit slow

P, Q, R, S, T waves. • 75% Simulator is very

**S14** One-shot

pre-test – post-test

O X O

Nonexperimental 1 e 2

After you can choose

the diagram of nine predefined

arrhythmia types.

• • The student learns through access to a theoretical module on ECG and its components, sinus rhythm, causes of arrhythmias,

including a detailed

review of different arrhythmias and theory in cardiac

successful

• The mean score of the test significantly improved from the pre-test to the post-test;

• In general, it has been shown to have positive effects on the results, such as the knowledge and skills, in comparison with the absence of intervention;

blocks, bundle branch blocks, hypertrophy patterns, heart axis , low tension, and ischemia. He then accesses a training module with the opportunity to interpret 15 different ECGs (including clinical scenarios) with feedback.

• New students and experienced professionals experienced results of comparable score, suggesting that both groups benefited equally from the intervention;

• Therefore, WBL can be an effective means of teaching skills of ECG interpretation for medical students from a wide range of semesters;

• The newly acquired skills were quickly lost as the gain of initial score was reduced to half within 2-4 weeks, but we couldn't find any additional decrease in interpreting ECG skills in

10-12 weeks or 18-20 weeks.

R O X1 O R O X2 O

| **S15** | Pre test  Posttest 3 months later | experimental | 2 | Questionnaires | • •• Students observed scenarios in a virtual | • The results showed that students in the experimental |
| --- | --- | --- | --- | --- | --- | --- |
|  |  |  |  |  | simulator (with many | group subject to education |
|  | Control and |  |  |  | features such as blood  pressure | based on simulation showed  significant improvement in |
|  | Experimental group |  |  |  | measurement, pulse | the score of knowledge of |
| Randomized pretest - posttest  Control group | | | | | palpation, ECG, chest | arrhythmia in both the first |
|  |  |  |  |  | expansion and | and second post-test, |
|  |  |  |  |  | simulator voice); | compared with the control |
|  |  |  |  |  | • • Students received | group who were taught by |

normal ECG rhythms, which were dynamic and displayed on a monitor and representing patient conditions. All waves, intervals and segments were illustrated for students;

• • Scenarios (set of scenarios) showed some cardiac arrhythmias.

• Students had to identify arrhythmias and suggest appropriate interventions, including drugs or the use of a defibrillator or pacemaker;

• Thus, the students saw the results appear as if they were in a real patient

means of a traditional method lecture.

**S16** Randomized pre-test –

Experimental 1 e 2 Questionnaires

• Students are led • The group of almost-pairs

post-test

R O X O R O O

through a series of sections that illustrate the basic principles of ECG interpretation. And then they should perform small multiple-choice summative assessments to consolidate knowledge

(84%) presented a performance significantly higher than the group e- learning (74.5%)

| **S17** | Static  comparison group | group | Quasi  experimental | 1 | Questionnaires | • • Participants used an interactive simulator | • There were no differences in scores on the tests of ECG |
| --- | --- | --- | --- | --- | --- | --- | --- |
|  | X O |  |  |  |  | for 45 min. They  learned aspects about | interpretation immediately  after or 3 months after the |

|  | X O |  |  |  | ECG interpretation,  which consisted of examples of ECGs with 12 leads, containing  diagnostics and an interactive 3D  animation of the conductive tissue of the heart and explanations of text for each diagnosis. | teaching in groups of  lectures or simulators. |
| --- | --- | --- | --- | --- | --- | --- |
